# Identification, Characterization, and Transcriptional Reprogramming of Epithelial Stem Cells and Intestinal Enteroids in Simian Immunodeficiency Virus Infected Rhesus Macaques

**DOI:** 10.3389/fimmu.2021.769990

**Published:** 2021-11-23

**Authors:** Nongthombam Boby, Xuewei Cao, Alyssa Ransom, Barcley T. Pace, Christopher Mabee, Monica N. Shroyer, Arpita Das, Peter J. Didier, Sudesh K. Srivastav, Edith Porter, Qiuying Sha, Bapi Pahar

**Affiliations:** ^1^ Division of Comparative Pathology, Tulane National Primate Research Center, Covington, LA, United States; ^2^ Department of Mathematical Sciences, Michigan Technological University, Houghton, MI, United States; ^3^ Division of Veterinary Medicine, Tulane National Primate Research Center, Covington, LA, United States; ^4^ Division of Microbiology, Tulane National Primate Research Center, Covington, LA, United States; ^5^ Department of Biostatistics, Tulane University, New Orleans, LA, United States; ^6^ Department of Biological Sciences, California State University, Los Angeles, Los Angeles, CA, United States; ^7^ Department of Microbiology and Immunology, Tulane University School of Medicine, New Orleans, LA, United States; ^8^ Department of Tropical Medicine, Tulane School of Public Health and Tropical Medicine, New Orleans, LA, United States

**Keywords:** intestinal stem cell (ISC), SIV, epithelial cells, LGR5, alpha defensin

## Abstract

Epithelial cell injury and impaired epithelial regeneration are considered key features in HIV pathogenesis and contribute to HIV-induced generalized immune activation. Understanding the molecular mechanisms underlying the disrupted epithelial regeneration might provide an alternative approach for the treatment of HIV-mediated enteropathy and immune activation. We have observed a significant increased presence of α defensin5+ (HD5) Paneth cells and proliferating Ki67+ epithelial cells as well as decreased expression of E-cadherin expression in epithelial cells during SIV infection. SIV infection did not significantly influence the frequency of LGR5+ stem cells, but the frequency of HD5+ cells was significantly higher compared to uninfected controls in jejunum. Our global transcriptomics analysis of enteroids provided novel information about highly significant changes in several important pathways like metabolic, TCA cycle, and oxidative phosphorylation, where the majority of the differentially expressed genes were downregulated in enteroids grown from chronically SIV-infected macaques compared to the SIV-uninfected controls. Despite the lack of significant reduction in LGR5+ stem cell population, the dysregulation of several intestinal stem cell niche factors including Notch, mTOR, AMPK and Wnt pathways as well as persistence of inflammatory cytokines and chemokines and loss of epithelial barrier function in enteroids further supports that SIV infection impacts on epithelial cell proliferation and intestinal homeostasis.

## Introduction

Human immunodeficiency virus (HIV) continues to be a cause of major global life-threatening disease, with 37.7 million people are living with HIV/AIDS worldwide in 2020. Around 36.3 million people have died from AIDS-related illnesses since the start of the AIDS epidemic in 1981. In 2020 alone, an estimated 1.5 million people were newly infected with HIV, and over 680,000 people died from AIDS-related illnesses (1). Loss of intestinal barrier function and subsequent translocation of luminal bacteria are now thought to be the major cause of the chronic systemic immune activation that perpetuates HIV replication and progression to AIDS (2–4). The simian immunodeficiency virus (SIV) infected Rhesus Macaque (*RhM*) model is a well-accepted model for the study of HIV-associated enteropathy (5, 6), and we have discovered that intestinal epithelial cell (iEC) apoptosis and loss of tight junction (TJ) proteins occur soon after SIV infection (7, 8). Internalization of IL-10R with the resultant impact on IL-10 signaling and dysregulation of the IL-10 and TGF-β-mediated anti-inflammatory responses also play a crucial role in iEC damage and subsequent SIV-mediated pathology (7, 9) and may contribute to diminished iEC regeneration. Each intestinal villus in the small intestine is encircled by at least six crypts of Lieberkühn, which provide populations of stem cells that self-renew and give rise to the various differentiated iECs (10). LGR5 (leucine-rich repeat G-protein-coupled receptor 5) is one of the best characterized markers for crypt base columnar (CBC) cells located at the base of intestinal crypts, also referred to as intestinal stem cells (ISCs) (10, 11). An additional set of reliable surface markers (CD44+CD166+CD24^lo^) was used to characterize human ISCs (12). Genetic analysis of LGR5+ ISCs revealed that a single intestinal stem cell can maintain crypt homeostasis in adult intestines by clonal expansion (13). Stem cells residing at the crypt base proliferate into transiently amplifying (TA) cells, which rapidly migrate upward and differentiate into several other cell types, namely enteroendocrine cells, goblet cells, tuft cells, M cells, enterocytes, and Paneth cells (PCs) (14). Unlike the other cell types, PCs move downward and take residence at the bottom of the crypt (15). As soon as there is damage in the intestinal lining, the intestinal epithelium undergoes a repair process to restore the structural and functional integrity of the intestine (16, 17). The repair process encompasses epithelial cell restitution, proliferation, and differentiation. Epithelial restitution is a rapid process during which injured regions of the epithelial lining are covered by the surrounding epithelial cells. The cells at the edge of the damaged area extend over the denuded basal lamina, reform cell contacts, and reestablish barrier function, thereby limiting fluid and electrolyte losses and preventing direct exposure of submucosa to foreign antigens (18, 19). After epithelial restitution, ISCs proliferate and differentiate to replenish the decreased cell population and to reinstate the epithelial cell functions, respectively. The study of ISCs has been greatly advanced through the introduction of enteroids, which were first derived from mouse intestine (20, 21).

In HIV/SIV-induced enteropathy, the intestinal architecture is altered (22); however, the impact on ISCs and PCs in SIV infection is not well understood. Moreover, the dynamics of intestinal LGR5+ cells and molecular changes in enteroids from normal and SIV-infected *RhMs* have not been well defined. In this study we utilized a *RhM* model of SIV infection to identify and characterize LGR5+ ISCs and intestinal enteroids and to address the impact of SIV on LGR5+ ISCs and PCs within the intestinal structure. We have also delineated the transcriptomic changes detected in enteroids obtained from SIV-infected *RhMs*.

## Materials And Methods

### Animals, Housing, and Ethics Statement

A total of 40 *RhMs* (*Macaca mulatta*) of both sexes between 4.2 and 14.1 years of age were used. At the beginning of this study, all animals were antibody and virus-negative for HIV-2, SIV, STLV-1, simian retrovirus type D, and Macacine herpesvirus 1. All animals were housed at the Tulane National Primate Research Center (TNPRC, full accreditation by AAALAC) and were under the care of TNPRC veterinarians for all sample collection and surgical procedures. The study was approved by the TNPRC Institutional Animal Care and Use Committee (IACUC) and was conducted within the guidelines of the United States Public Health Service Policy and the Guide for the Care and Use of Laboratory Animals (23). All *RhMs* were grouped into uninfected controls (n=17), acute SIV infection (8–21 days after SIV infection, n=10), and chronic SIV infection (150–422 days after SIV infection, n=13) based on days after SIV_MAC_251 infection (**Table 1**). All acute and chronic SIV-infected *RhMs* were inoculated with 100-1000 TCID_50_ pathogenic SIV_MAC_251 using either intravenous (IV) or intravaginal (IVAG) routes (**Table 1**). In our previous studies, we have not detected any association between viral dosage, CD4 depletion, and viral loads in *RhMs* (7, 24, 25). We have also used IV and IVAG routes of SIV inoculation to mimic the major routes of HIV transmission in humans. At necropsy, peripheral blood and jejunum tissues were collected. Samples were processed for subsequent cellular and molecular assays.

**Table 1:** Cumulative List of Adult Rhesus Macaques Examined.

Category	Animal Number	Age (Year)	Sex^a^	Virus	Days of Infection	Dosage (TCID_50_)	Route^b^	Terminal Plasma Viral Load (RNA copies/mL)
UNINFECTED CONTROL	GI92	4.2	M	Nil	–	–	–	–
	JG16	4.3	M	Nil	–	–	–	–
	GT20	4.6	M	Nil	–	–	–	–
	FK25	5.8	M	Nil	–	–	–	–
	EV39	6.2	M	Nil	–	–	–	–
	HG11	7.1	M	Nil	–	–	–	–
	JM76	6.4	M	Nil	–	–	–	–
	JV66	7.2	M	Nil	–	–	–	–
	JB65	7.6	M	Nil	–	–	–	–
	JT62	8.0	M	Nil	–	–	–	–
	DJ78	8.1	F	Nil	–	–	–	–
	JG77	8.9	F	Nil	–	–	–	–
	JD56	9.1	F	Nil	–	–	–	–
	GN70	10.1	F	Nil	–	–	–	–
	HR97	10.8	F	Nil	–	–	–	–
	AG71	11.1	F	Nil	–	–	–	–
	GN74	13.3	F	Nil	–	–	–	–
ACUTE SIV	AV91	14.1	M	SIV_MAC_251	10	500	IV	157,190,000
	GI28	5.9	F	SIV_MAC_251	21	500	IVAG	5,830,000
	HI53	6.6	F	SIV_MAC_251	8	100	IV	3,555,700
	FT35	6.7	F	SIV_MAC_251	21	500	IVAG	3,540,000
	EK98	8.7	F	SIV_MAC_251	21	500	IVAG	26,800,000
	EM64	8.9	F	SIV_MAC_251	21	500	IVAG	3,840,000
	CF65	12.3	F	SIV_MAC_251	21	500	IVAG	10,100,000
	HN29	12.6	F	SIV_MAC_251	10	100	IV	110,000,000
	BA57	14	F	SIV_MAC_251	8	500	IV	14,288,200
	M992	16	F	SIV_MAC_251	13	500	IV	34,949,800
CHRONIC SIV	JK56	8.2	M	SIV_MAC_251	180	100	IV	6,480,000
	GN91	4.9	F	SIV_MAC_251	401	500	IVAG	4,190
	EJ26	6.2	F	SIV_MAC_251	309	100	IV	397,806
	DR59	6.3	F	SIV_MAC_251	250	1000	IVAG	288,441
	EB09	6.3	F	SIV_MAC_251	250	100	IVAG	750,720
	FK88	6.5	F	SIV_MAC_251	226	500	IVAG	2,314,583
	KP54	6.8	F	SIV_MAC_251	183	100	IV	3,060,000
	KA42	7.8	F	SIV_MAC_251	181	100	IV	23,800,000
	HG58	9.1	F	SIV_MAC_251	288	300	IVAG	7,074
	DE50	9.7	F	SIV_MAC_251	150	500	IVAG	304,000
	CL86	11.1	F	SIV_MAC_251	281	500	IVAG	972,889
	BC35	11.9	F	SIV_MAC_251	422	300	IVAG	428,298
	BD03	12.8	F	SIV_MAC_251	167	500	IVAG	14,788,890

a F and M denote female and male, respectively.

b IV and IVAG denote intravenous and intravaginal route, respectively.

TCID_50_ represents tissue culture infectivity dose at 50%.

Macaques were housed indoors in Animal Biosafety Level 2 housing with 1:1 light/dark hour cycle, relative humidity of 30-70%, and climate-controlled conditions. Veterinarians monitored animals on a regular basis to ensure their welfare, and animals were fed commercially available monkey chow twice daily. The animals participated in the TNPRC environmental enrichment program, which is reviewed and approved by the IACUC semiannually. All clinical procedures, including administration of anesthesia and analgesics to minimize pain and distress during sampling procedures, were carried out under the supervision of a laboratory animal veterinarian. Animal surgeries and tissue collections were performed by pre-anesthetizing animals with ketamine hydrochloride, acepromazine, and glycopyrrolate, followed by intubation and maintenance of anesthesia with a mixture of isoflurane and oxygen. Buprenorphine was given intraoperatively and postoperatively for analgesia following the veterinarian’s recommendation.

### Plasma Viral Load Quantification

Plasma viral RNA was quantified by either a bDNA signal amplification assay (Siemens Diagnostics, USA) or quantitative reverse transcription-PCR (RT-PCR) with a lower detection limit of 125 and 60 SIV RNA copies/mL of plasma, respectively (7).

### Crypt Isolation From *RhM* Jejunum for Enteroid Culture

Crypts from jejunum were isolated using a low-temperature method standardized in our laboratory and described previously (26). In brief, 3–5 cm of jejunum was collected in a 50 mL tube and thoroughly cleaned with sterile PBS by shaking the tube gently, discarding the fluid and repeating the washes until the fluid was clear. The tissue was chopped into pieces of 2–3 mm length in a Petri dish. The tissue pieces were transferred into a sterile 50 mL tube with 25 mL of 1 x HBSS (Thermo Fisher, USA) containing 1 mM Dithiothreitol (DTT, Thermo Fisher Scientific, USA) and 5 mM EDTA (Thermo Fisher) to remove jejunum villi. The tube was kept in ice and constantly stirred at 200 rpm for 5 min. The undigested tissues were settled down for a minute and the supernatant (which mostly contains villi) was discarded. The undigested tissues were treated with chelating buffer (containing 27 mM Na_3_C_6_H_5_O_7_ (G-Biosciences, USA), 5 mM Na_2_HPO_4_ (USB corporation, USA), 96 mM NaCl (Sigma-Aldrich, USA), 8 mM KH_2_PO_4_ (VWR, USA), 1.5 mM KCl (Thermo Fisher), 0.5 mM DTT, 55mM D-sorbitol (VWR), 44 mM sucrose (VWR) with pH 7.3) which predominantly removes villi enterocytes. The tube was inverted slowly by hand at least 60 times. The tissues were allowed to settle for a minute and the supernatant was discarded. The treatment of tissues with EDTA and chelating buffer were repeated alternately four times. Finally, the crypts were allowed to separate from the tissues by tapping or shaking the tube vigorously in chelating buffer. The detached crypts were filtered through 100 µm cell strainer and 1% BSA (Sigma-Aldrich) in DMEM (Thermo Fisher Scientific) was added to reach 40 mL volume. The filtrate containing crypts was centrifuged at 4°C and 200 × *g* for 10 min. The isolated crypts were resuspended in DMEM + 1% BSA and the number of viable crypts was determined using trypan blue in a Glasstic™ Slide 10 with Grids (KOVA International, USA). A total of 20,000 crypts were aliquoted for the enteroid culture as described below. When possible, the remaining crypts were digested for flow cytometry staining by shaking in 10 mL of 1 x HBSS containing 6 U of Dispase (Gibco, USA) and 0.1 mg/mL of DNAse I (Sigma-Aldrich) at 37°C and 210 rpm using an orbital shaking incubator for 20 min. The isolated single cell suspension was stained with Viastain AOPI staining solution and counted using Cellometer (Nexcelom, Lawrence, MA, USA), and used for flow cytometry analysis when cells were >90% viable.

### Flow Cytometry Analysis of *RhM* Crypts

**Figure 1:**
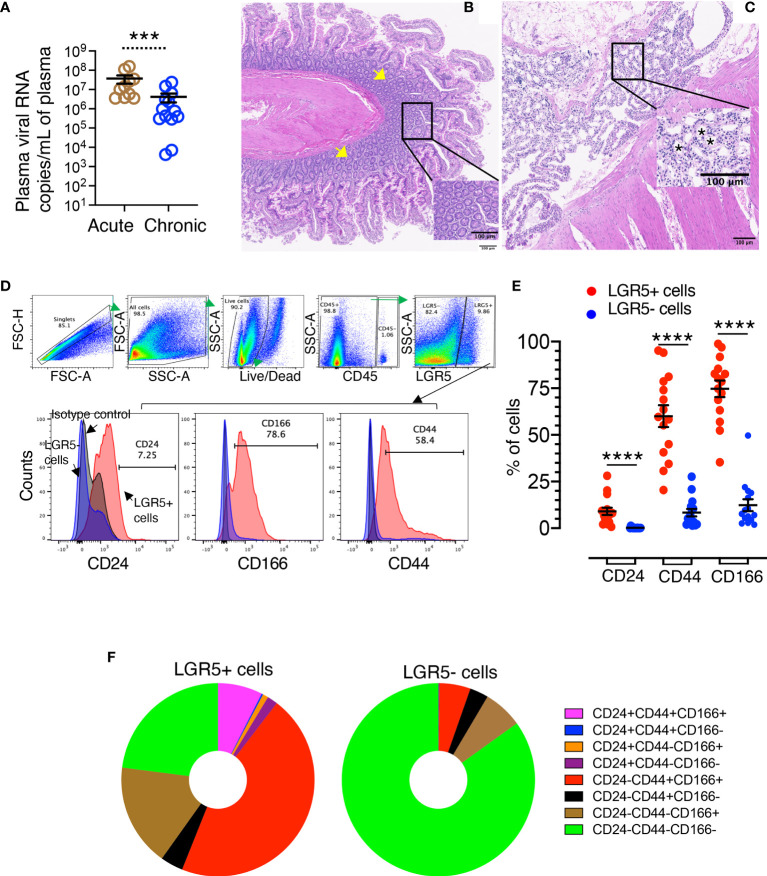
Isolation of jejunum crypts and phenotypic characterization of LGR5+ intestinal stem cells. **(A)** Plasma viral loads with mean (± standard errors) in macaques during acute (n=10) and chronic (n=13) phases of SIV_MAC_251 infection. Statistically significant differences between groups as analyzed with Mann-Whitney t-test are indicated with asterisks (***p = 0.001). **(B)** Jejunum crypts were isolated using a low temperature isolation method as discussed in the Materials and Methods section. Pre-processing normal jejunum tissue where the tissue architecture with abundant crypts and villi lined by enterocytes (yellow arrows) are maintained (JT62). Inset: Higher magnification of normal crypts. **(C)** Post processing jejunum tissue with a complete absence of enterocytes and a loss of crypts (JT62). Inset: Higher magnification showing empty spaces where crypts are missing (asterisks). **(D)** A representative gating strategy of LGR5+ cells from a normal healthy uninfected rhesus macaque is shown. Crypts were further enzymatically digested and single-cell suspension was further stained for different cell phenotypic markers. Cells were gated first on singlets, all cells, followed by live cells, and then gated on CD45- cells. All CD45- cells were further gated for LGR5+ and LGR5- cells. Both LGR5+ and LGR5- cells were further gated on different cell surface markers like CD24, CD44, and CD166. The percentages of either CD24, CD44 or CD166 for LGR5+ cells (in red color) are shown in the left bottom row, where the positive responses for those surface markers were compared to the isotype (grey color) and LGR5- cells (blue color) using histograms. **(E)** Nine SIV-uninfected normal RMs, of which six were run in duplicate, were examined for their LGR5 expression. Frequency of CD24, CD44, and CD166 surface markers were compared between LGR5+ and LGR5- cells. The horizontal line denotes the mean frequencies (± standard errors) of each category. Statistically significant differences between cells as analyzed with Mann-Whitney T-test are indicated with asterisks (****p < 0.0001). **(F)** LGR5+ and LGR5- cells were further analyzed based on their CD24, CD44, and CD166 surface expression. The majority of the LGR5+ cells were found to be positive for CD44 and CD166 surface molecule expression, whereas about 85% of LGR5- cells were found negative for CD24, CD44, and CD166 markers. One-way ANOVA with Tukey’s multiple comparison test was performed to calculate significant differences among different cell subsets. A *p*-value < 0.05 was considered significant.

Single cell suspension obtained from crypts were stained for flow cytometry following the protocol described previously (7, 27). Briefly, 1–1.5 X 10^6^ cells resuspended in 100 µL of flow wash buffer (0.5% BSA in PBS) were first stained with live/dead dye (Invitrogen, USA), and subsequently with fluorochrome-conjugated monoclonal antibody (MAb) cocktail for 25 min at room temperature. The MAbs used in the studies were anti-CD24 FITC, anti-LGR5 PE, anti-CD166 BV421, anti-CD45 BV605 and anti-CD44 BV711 (**Supplementary Table 1**). CD166 (ALCAM) plays an important role in ISC maintenance and proliferative capacity (28). CD44 is a prominent Wnt signaling target in the intestine and is highly expressed in the regenerating ISCs (29). CD24 and CD44 are differentially expressed in LGR5+ stem cells (30). Anti-LGR5 antibody was custom conjugated with PE fluorochrome using lightning link R-PE conjugation kit (Novus Biologicals, USA) following the manufacturer’s protocol. The cells were washed with wash buffer and fixed in BD stabilizing and fixative buffer (BD Biosciences, USA). At least 50,000 cells were acquired either using BD LSRII or Fortessa instruments (BD Biosciences), and the data were analyzed using FlowJo software (version 10.7.1., BD Biosciences). Singlets and live cell populations were subjected to downstream analysis for quantifying all the cell surface markers (**Figure 1**).

### Immunohistochemistry Staining

To quantify LGR5+ cells in jejunal tissues, formalin-fixed tissue sections were stained with anti- LGR5 antibodies using the Mach3 Rabbit AP-Polymer Detection Kit (Biocare Medical, USA) as performed earlier (31). The paraffin-embedded tissue sections were deparaffinized and epitope retrieved by heating the tissue sections in low pH citrate buffer (Vector Laboratories, USA) using a microwave. After blocking with a serum-free protein blocker (Agilent Dako, USA) for 30 min, the tissue sections were incubated with anti-LGR5 primary antibodies (**Supplementary Table 1**). The negative control slide consisted of rabbit Ig fractions (R&D Systems, USA) used at the same isotype and concentration as LGR5 antibodies to determine the background intensity and staining. The tissue sections were subsequently treated with the kit’s probe and polymer and finally developed using BCIP/NBT (Agilent Dako) chromogen system. The slides were then mounted with Vecta Mount AQ (Vector Laboratories). An average of 19–20 view fields (0.116mm^2^/view field under a 400 x magnification) were enumerated in each of the slides to quantify LGR5+ cells manually. The tissue sites for this evaluation were selected randomly. Tissues were analyzed by two different blinded individuals to avoid bias and averaged prior to the statistical analysis.

### Histopathological Analysis

Paraffin-embedded jejunum specimens from uninfected control *RhMs* before and after the crypt isolation process were stained with hematoxylin and eosin (H&E) and evaluated according to standard pathology scores.

### Immunofluorescence Staining

Formalin-fixed tissue sections were processed for immunofluorescence with one or a combination of primary antibodies as described earlier (7, 8, 24). In brief, deparaffinized tissue sections were subjected to antigen retrieval and successively incubated with one or two different fluorescence antibodies including anti-cytokeratin, anti-E-cadherin, anti-HD5 [human α defensin 5 for PCs (6)], anti-Ki67, and/or anti-LGR5 primary antibodies (**Supplementary Table 1**). Alexa Fluor 488 or Alexa Fluor 568 conjugated secondary antibodies (1:1000 dilution, Invitrogen, USA) were used for staining. Anti-nuclear ToPro-3 antibodies (1 µM, Life Technologies, USA) or DAPI (1:5000 dilution, EMD Millipore, USA) were used for nuclear counterstaining. Labeled tissue sections were mounted using Prolong Gold antifade medium (Invitrogen). Images were captured using a TCS SP2 confocal laser scanning microscope (Leica, Germany) equipped with an argon-krypton laser at 488 nm (green), a krypton laser at 568 nm (red), and a helium-neon laser at 633 nm (blue). Negative controls consisted of either omitting the primary antibody or using isotype IgG1 and IgG (H+L) controls. ImageJ (version 1.53a; National Institutes of Health, USA) and Adobe Photoshop (USA) were used to assign pseudocolors to the channels collected. Quantitative fluorescence densitometry was performed using ImageJ software to quantify E-cadherin and Ki67 expression in the jejunum. An average of 19–20 view fields chosen at random (0.232mm^2^/view field under a 200 x magnification) were quantified. For determining Ki67 expression in epithelial cells, region of interest (ROI) was drawn to limit analysis to epithelium only and fluorescence intensity was quantified within that region. The intensity of E-cadherin and Ki67 protein expression is represented as fluorescence pixel values. For quantification of HD5+ cells, positive cells were counted in a minimum of 20 fields using a Nuance FX multispectral imaging system with colors assigned using Nuance version 2.10 software (Cri, USA). The data is presented as positive cells/mm^2^ of the tissue. For immunofluorescence staining of enteroids (see below), enteroids grown in 8-chambered polystyrene vessel tissue culture-treated glass slides (Falcon, USA) for 7 days were fixed with 2% paraformaldehyde for 30 minutes at room temperature and washed before staining with primary antibodies.

### *RhM* Enteroid Culture and Its Subculture

Isolated jejunum crypts were seeded and cultured *in vitro* using the protocols described for human ISCs with modifications (12, 32). In brief, 1000 crypts suspended in 25 μL DMEM + 1% BSA media were mixed with 25 μL of BD Matrigel basement membrane matrix so that 50 μL of the crypt suspension could be cultured per well in a pre-warmed 24-well cell culture plate (Corning, USA). After carefully dispensing the crypt and matrigel mixture on the plate well, the plate was kept at 37°C for 10 min to allow full gel polymerization. After polymerization, 750 μL of prewarmed complete seeding media was added to each well and the plate was placed into a 37°C incubator under 5% CO_2_. Complete seeding media consisted of advanced DMEM/F12 medium (Life Technologies) with 2 mM glutamine (Life Technologies), 10 mM HEPES (Life Technologies), 100 U/mL penicillin (Life Technologies), 100 μg/mL streptomycin (Life Technologies), 1 x N2 supplement (Life Technologies), 1 x B27 supplement (Life Technologies), 1% BSA (Sigma-Aldrich), 50% Wnt-3A-conditioned medium (prepared in-house using L Wnt-3A cell line (ATCC, USA), 1 µg/mL R-Spondin 1 (R&D Systems), 1 mM N-acetylcysteine (Sigma-Aldrich), 500nM A-83-01 (Peprotech, USA), 10 nM [Leu]15-Gastrin (Sigma-Aldrich), 10 mM Nicotinamide (Peprotech), 50 ng/mL EGF (Sigma-Aldrich), 100 ng/mL Noggin 1 (R&D Systems), and 10 μM SB202190 (Sigma-Aldrich). During the first two days of culture, the culture medium was also supplemented with 2.5 µM Thiazovivin (Stemgent, USA) and 2.5 µM CHIR99021 (Stemgent). The culture was monitored regularly under an inverted brightfield microscope for cell division and development, and the medium was replaced with 750 μL of fresh prewarmed complete seeding medium every 2 days.

Fully grown enteroids with multiple budding and thickened and darkened epithelial membranes, typically 10-12 days after primary culture, were used for subculturing. Dissociation of enteroids was performed using gentle cell dissociation agent (Stemcell Technologies Inc., USA) and trypsin-EDTA (Gibco) treatment. In brief, enteroids were harvested after 11 min treatment in gentle cell dissociation reagent at room temperature. Harvested cells were washed with DMEM/F-12 media. The cells were further treated with trypsin-EDTA for 10 minutes at 37°C. Cells were disrupted by gently pipetting with wide orifice pipet tips followed by washing with DMEM/F-12 medium. Cells were examined under an inverted bright field microscope for sufficient disruption and achievement of a single cell suspension. Cells were then counted, and 1000 cells were used for subculturing after mixing with BD Matrigel basement membrane matrix as discussed initially to determine its efficiency to redevelop enteroids and maintenance of phenotypic characteristics by immunohistochemistry assays.

### RNA Isolation, cDNA Synthesis, and PCR

For gene expression analysis, jejunal crypts and enteroids prepared as described above from uninfected normal and chronically SIV-infected RhMs were resuspended in RLT buffer (Qiagen, Germany) and vortexed at high speed for 1–2 min. The lysate was loaded onto a QIAshredder spin column (Qiagen) which was then spun for 2 min at 11,000 rpm. The flow-through was carefully transferred into a new microcentrifuge tube and mixed with one volume of 70% ethanol. The cell lysate preparation was subsequently used for RNA isolation including DNAse treatment for 15 min at room temperature as per the manufacturer’s instructions (RNAeasy mini kit, Qiagen). The quantity and quality of the total RNA was assessed using RNA 6000 Pico kit of the Bioanalyzer system (Agilent Technologies, USA). cDNA from the isolated RNA was synthesized using the Superscript IV first-strand synthesis system (Thermo Scientific) using the manufacturer’s protocol. To remove residual RNA from the final product, 1 μL of *E. coli* RNAse H was added to 20 μL of the reaction mixture and incubated at 37°C for 20 min. The cDNA products were stored at -20°C for further use. PCR was performed using the DreamTaq green PCR master mix (Thermo Fisher Scientific). Each 25 µL PCR reaction mixture contained 1 x DreamTaq green PCR master mix, 10 µM forward primer, 10 µM reverse primer, and 1 µL of synthesized cDNA. The thermal cycling comprised an initial denaturation at 95°C for 5 min; 36 cycles of denaturation at 95°C for 30 s, annealing at 56–60°C (**Supplementary Table 2**) for 40 s and extension at 72°C for 45 s; and a final extension at 72°C for 10 min (T1000 Thermal Cycler, Bio-Rad, USA). The primers in the study were designed using an online primer designing tool (Integrated DNA Technologies, USA) and analyzed with OligoAnalyzer (Integrated DNA Technologies). Proper negative control with no template and a positive control targeting GAPDH housekeeping gene were included for each reaction. The amplified products were separated in 1.5% low electroendosmosis agarose prepared in 1 x TAE buffer and visualized under UV gel documentation system.

### Generation of RNA-Seq Data

To assess SIV-mediated changes in the enteroid transcriptome, enteroids derived from 4 SIV-uninfected normal and 3 chronically SIV-infected *RhMs* were subjected to next-generation sequencing. One µg of the extracted RNA from primary enteroids grown for 12–13 days was used for cDNA library construction at Novogene using an NEBNext^®^ Ultra RNA Library Prep Kit for Illumina^®^ (cat# E7420S, New England Biolabs, USA) according to the manufacturer’s protocol. Briefly, mRNA was first enriched by removing ribosomal RNA using the Ribo-Zero kit. The mRNA was fragmented randomly using divalent cation under elevated temperature in the NEBNext first-strand synthesis reaction buffer, then the first-strand cDNA was synthesized using the fragmented mRNA as template and random hexamers primer. Second-strand cDNA was subsequently generated using dNTPs, RNase H and DNA polymerase I. After a series of terminal repair, a ligation and sequencing adaptor ligation, the double-stranded cDNA library was prepared. cDNA libraries of 250–350 bp were preferentially selected and enriched with PCR using Phusion High-Fidelity DNA polymerase. The quantity and quality of the resulting cDNA was assessed by using a Qubit 2.0 fluorometer (Thermo Fisher Scientific) and an Agilent 2100 Bioanalyzer (Agilent Technologies), respectively. Qualified libraries were sequenced on an Illumina Nova Seq 6000 Platform (Illumina, USA) using a paired-end 150 run (2 x 150 bases). Twenty million raw reads were generated from each library. These raw reads were stored in the FASTQ format using (bcl2fastq2) conversion software (v2.17).

### Transcriptome Assembly

Raw reads (in FASTQ format) were subjected to sequence quality control using FastQC (v0.11.9 released: http://www.bioinformatics.babraham.ac.uk/projects/fastqc/) (33). FastQC performed a series of analysis modules on raw reads and created a report with statistics for the data analyzed. For each library, FastQC showed high per base/tile sequence quality, exceeding 34 on Phred scale (less than 1/2000 chance of a base being wrong) and few overrepresented sequences. We used TopHat2 (34) to map the raw reads to the reference sequences and annotations of *RhM* from iGenomes (https://support.illumina.com/sequencing/sequencing_software/igenome.html), which is a collection of reference sequences and annotation files for commonly analyzed organisms. Reads with multiple alignments were discarded. The gene expression counts were calculated using Htseq-count in Galaxy platform (35) (https://usegalaxy.org/).

### Differential Gene Expression Analysis

Transcriptome profiling and data analysis were performed using DESeq2 (36) in R/Bioconductor (https://bioconductor.org/packages/release/bioc/html/DESeq2.html). At first, genes with a read count smaller than 10 were excluded from further analysis. In order to remove the dependence of the variance on the mean, we used regularized-logarithm transformation (rlog) to transform the read counts per gene to log2 scale. Then, principal component analysis (PCA) was applied to obtain an impression on the similarity of SIV-infected and uninfected controls using “DESeq2” so we could identify subgroups or outliers. Differentially expressed genes (DEGs) were identified by comparisons of the counts of SIV-infected and uninfected controls with false discovery rate (FDR) < 0.05 and the absolute value of log2 fold change > 1, where the FDR is the adjusted *P* value obtained by applying Benjamini and Hochberg’s (BH) FDR correction on the original *P* value, and fold change is a measure describing how much a quantity changes between different group of *RhMs*.

We ranked the DEGs based on the FDR from smallest to largest and created hierarchical clustering trees based on the Pearson correlation coefficient for each animal with the top 40 DEGs and the following top 60 DEGs, respectively. The heat maps were generated using the “*pHeatmap*” R package (https://cran.r-project.org/web/packages/pheatmap/index.html). The heat maps also directly reflected the clustering of groups of samples, enabling the detection of the differential expression patterns of the genes in the SIV-infected and uninfected *RhMs*.

### Pathway Enrichment Analysis

In order to better understand the biological meaning behind the variant DEGs, we identified the molecular functions or pathways in which DEGs were involved. All DEGs were mapped to the Kyoto Encyclopedia of Genes and Genomes (KEGG) pathways using a functional annotation tool named Database for Annotation, Visualization, and Integrated Discovery Bioinformatics Resource (DAVID: https://david.ncifcrf.gov/) (37, 38) for pathway enrichment analysis. KEGG pathways predominantly harvest pathway clusters covering our knowledge on the molecular interaction and reaction networks in DEGs, which are more focused on the metabolic pathways (39). In this study, significantly enriched pathways were identified by DEGs if FDR < 0.05. Moreover, genes weighted by length and categories with FDR < 0.05 were identified as being significantly enriched in the corresponding pathways.

### Gene Ontology (GO) Functional Analysis

The GO functional analysis mainly covers domains of biological processes, molecular function, and cellular components. We performed GO functional analysis of DEGs that showed significant transcriptional changes in enteroids between SIV-infected and uninfected *RhMs* by the Gene Ontology Resource (http://geneontology.org/). We used the same criteria as for the pathway enrichment analysis; we claimed significantly enriched GO terms by DEGs if FDR < 0.05, and significantly enriched genes in the corresponding GO terms with FDR < 0.05.

### Statistical Analysis

Statistical analysis and graphical representation of the data were performed using GraphPad Prism version 9.1.1 (GraphPad Software, USA). One-way ANOVA was applied to determine any statistically significant differences between the group means. Scatter plots are presented as a graphical method for comparing the frequency of different subsets of LGR5+, LGR5-, and HD5+ cells as well as fluorescence intensity of E-cadherin and Ki67 expression in SIV-uninfected and infected *RhMs*. To compare the expression of CD24, CD44, and CD166 between LGR5+ and LGR5- cells, Mann-Whitney T-test was applied. Analysis of Variance (ANOVA) followed by Tukey-Kramer multiple comparison as *post hoc* analysis was applied for LGR5, HD5, E-cadherin, and Ki67 expression analysis to observe statistical significance between different groups. Correlation analyses were performed with a two-tailed Pearson correlation method. A *p* value of < 0.05 was considered statistically significant.

## Results

### Terminal Plasma Viral Loads in SIV Infected *RhMs*

All SIV-infected *RhMs* had detectable plasma viral loads ranging from 4.2 x 10^3^ to 1.6 x 10^8^ copies of RNA/mL of plasma. Acute SIV-infected *RhMs* had significantly higher levels of plasma viral load (ranging from 3.5 x 10^6^ to 1.6 x 10^8^ copies of RNA/mL of plasma) compared to chronically infected macaques (ranging from 4.2 x 10^3^ to 2.4 x 10^7^copies of RNA/mL of plasma) (**Figure 1A**, *p* = 0.001). No significant difference in plasma viral load was detected between IV and IVAG inoculated *RhMs* during either acute or chronic SIV infection (**Supplementary Figures 1A, B**).

### Isolation of Crypts From Normal *RhM* Jejunum Tissues

To confirm that the low-temperature crypt isolation technique provides well-defined crypts, we performed H&E staining on both pre- and post-processing tissues (**Figures 1B, C**). Preprocessing jejunum tissue showed normal architecture with abundant crypts and villi which were lined by enterocytes. However, after crypt isolation using a low-temperature crypt isolation procedure, the overall intestinal architecture was maintained but a loss of enterocytes and crypts was noticeable, suggesting that this protocol was able to remove a majority of crypts from tissues without introducing major alterations of the remaining tissue (**Figures 1B, C**). Our isolation protocol yielded an average 0.6–0.8 x 10^6^ crypts/3 cm^2^ of jejunum tissue from normal *RhMs*.

### Characterization of LGR5+ and LGR5− Intestinal Epithelial Cells

The phenotype of LGR5+ cells in jejunum was determined by flow cytometry assay using enzymatically digested crypts. Cells were analyzed by gating on singlets followed by all cells, live cells, and finally gated on CD45− cells. All CD45− cells were further gated on LGR5+ and LGR5− cells. Both LGR5+ and LGR5− cells were further analyzed for their CD24, CD44, and CD166 surface expression (**Figure 1D**). We detected significantly increased expression of CD24 (mean ± the standard error, 9.1 ± 2.0%), CD44 (mean ± the standard error, 60.0 ± 5.8%), and CD166 (mean ± the standard error, 74.7 ± 4.5%) in LGR5+ cells compared to LGR5− cells (mean ± the standard error, 0.2 ± 0.1%, 8.4 ± 2.0%, and 12.3 ± 3.2%, for CD24, CD44, and CD166 markers, respectively, *p* < 0.0001) (**Figure 1E**). Using combination gating strategy, we further analyzed those markers and found that the largest proportion of LGR5+ cells were CD24-CD44+CD166+ (mean ± the standard error, 45.6 ± 5.7%) followed by CD24-CD44-CD166- (mean ± the standard error, 22.9 ± 4.0%), CD24-CD44-CD166+ (mean ± the standard error, 17.2 ± 3.6%), and CD24+CD44+CD166+ cells (mean ± the standard error, 7.4 ± 1.8%) (**Figure 1F**). In contrast, the majority of the LGR5− cells were negative for CD24, CD44, and CD166 surface expression (mean ± the standard error, 84.9 ± 3.3%), followed by CD24-CD44-CD166+ (mean ± the standard error, 6.6 ± 1.6%) and CD24-CD44+CD166+ cells (mean ± the standard error, 5.2 ± 1.5%) (**Figure 1F**). CD24-CD44-CD166- cells were significantly higher compared to any other subpopulation in the LGR5− cell population (**Figure 1F**). We also measured the purity of the epithelial cells by performing flow cytometry on the digesting crypts. Digested crypts had little to no leukocyte contamination as detected by CD45 marker (pan leukocyte markers with mean ± the standard deviation, 4.3 ± 3.7%).

### *RhM* Enteroid Characterization

**Figure 2:**
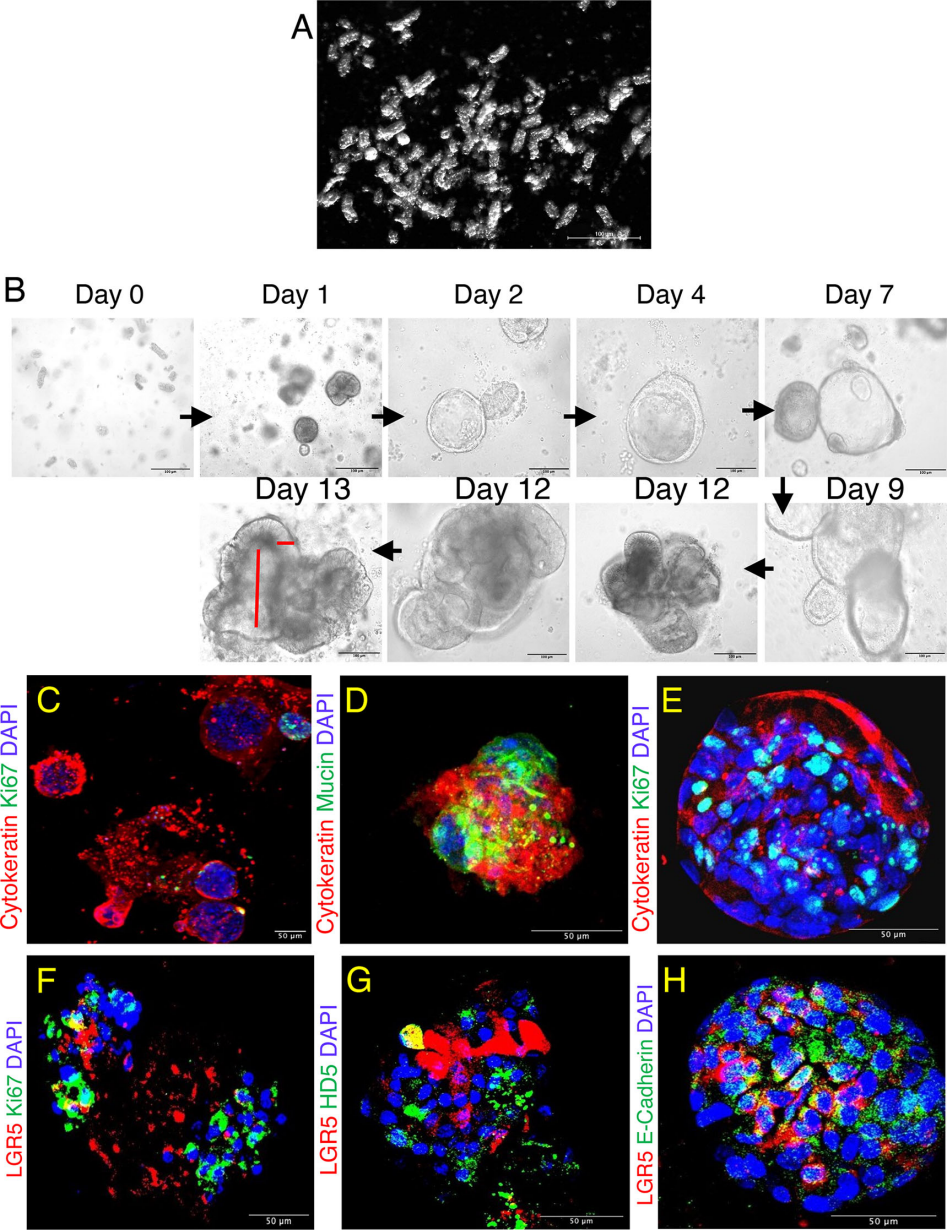
Jejunum enteroids and its phenotypic characterization in normal SIV infected rhesus macaque. **(A)** Isolated short tubular shaped glandular crypts show the purity of the crypt suspension detected by dark field microscopy. **(B)** Typical images of enteroids grown from single jejunum crypt. Lumen-like cystic structures started to form on days 2-4, budding events started on days 2-7, and maximum budding occurred at day 9 and was maintained onwards. The longitudinal and vertical red lines shown for day 13 represent enteroid lumen and epithelial cell lining, respectively. **(C)** Confocal images of enteroids showing the presence of epithelial cells (cytokeratin+) and proliferating (Ki67+) cells. Note that the proliferating cells were present at the tip of the budding structure. **(D–F)** Confocal images of subcultured enteroids showing goblet cells (mucin+, **D**) and proliferating cells (Ki67+, **E, F**) along with LGR5+ and epithelial markers after 7 days of culture. **(G, H)** First subculture of enteroids also shows expression of HD5 (Paneth cells+, **G**), and cell adhesion molecule (E-cadherin+, **H**) along with continued presence of LGR5+ stem cells after 7 days of culture. DAPI represents nuclear staining.

Enriched suspensions of intact crypts with classical tubular shape were obtained (**Figure 2A**). On average, more than 90% of the isolated crypts were viable as detected by trypan blue dye exclusion staining method (data not shown). Isolated crypts were cultured in basement membrane matrix, and from day 1, the crypts started rounding up to form enterospheres, with some of them starting their first budding at day 2. The majority of the crypt budding occurred by day 7, and then from day 9 onwards full enteroids were formed as indicated by the appearance of a lumen and epithelial lining (**Figure 2B**, see also red lines at day 13**)**. Enteroid cultures were maintained for a total of 12–13 days. Thereafter, enteroids began to disintegrate (data not shown). Most of the proliferating cells were detected in the bud of the tips along with the epithelial cells (Cytokeratin+, **Figure 2C**). For subculturing, 10–12-day enteroids were used, as they are at their peak of differentiation and budding at this time. Enteroids were characterized based on their morphology and their ability to differentiate into intestinal epithelial lineages by measuring the expression of cytokeratin, E-cadherin [adherens junction (AJ) protein/differentiation marker for epithelial cells], HD5 (for Paneth cells), mucin-2 (for goblet cells), and Ki67 (for proliferating cells) in subcultured enteroids (**Figures 2D–H**). We detected expression of cytokeratin and Ki67 throughout the subcultured enteroids, suggesting ongoing epithelial cell proliferation in those cultures. LGR5+ and HD5+ cells were also detected in subcultured enteroids, suggesting their continued regeneration and differentiation (**Figures 2E–H**). The presence of cytokeratin and mucin-2 expression in the original and subcultured enteroids indicated presence of absorptive enterocytes and goblet cells, respectively (**Figures 2D, E, H**), further supporting differentiation of enteroid ISC into functional cells.

### Molecular Characterization of Jejunal Crypts and Enteroids

To confirm the stemness properties of crypts and enteroids, RNA isolated from freshly isolated crypts as well as fully grown primary enteroids (between 10–12 days of culture) of healthy normal *RhMs* were used to determine the expression of LGR5, OLFM4, VIL1, MUC2, LGR4, FOXA2, and SPON1 genes by RT-PCR (**Supplementary Figure 2**). The reference gene IDs for these genes are given in **Supplementary Table 2**. The amplicon size (base pairs) of our target genes LGR5, OLFM4, VIL1, MUC2, LGR4, FOXA2, SPON1, and GAPDH were 232, 143, 120, 113, 202, 270, 231, and 120, respectively (**Supplementary Figure 2** and **Supplementary Table 2**).

### Localization of LGR5+ and HD5+ Cells and Their Frequency in Normal and SIV-Infected *RhMs*

**Figure 3:**
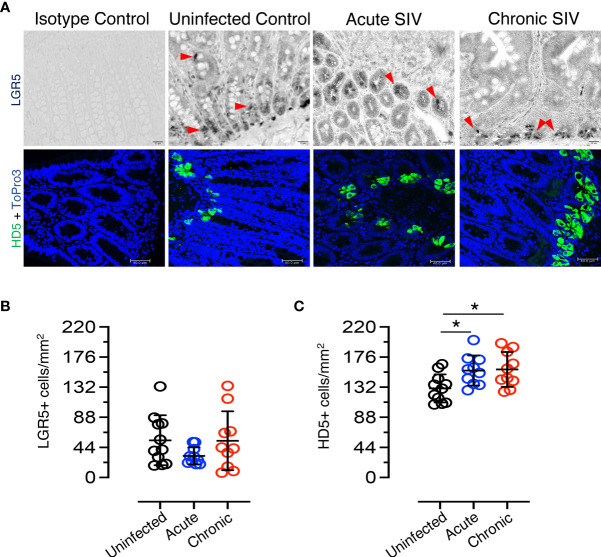
Dynamics of LGR5+ intestinal stem cells and HD5+ Paneth cells in SIV infection. **(A)** Top panels: LGR5+ staining; bottom panels: HD5 staining. Representative isotype control for LGR5 showing the absence of nonspecific background staining (macaque number FK25). LGR5+ cells from uninfected (macaque FK25), acute SIV (macaque EM64 at 21 days post infection), and chronic SIV (macaque FK88, 226 days post infection) *RhMs* are shown. The red arrows are representative of selected LGR5+ cells. HD5+ cells increase in SIV-infected *RhM* jejunum as detected by immunofluorescence staining. Representative isotype control for HD5 showing the absence of nonspecific background staining from an SIV-uninfected *RhMs* (macaque number AG71). Representative images of HD5+ cells in SIV-uninfected (macaque AG71), SIV acute (macaque EK98 at 21 days post infection), and SIV chronically (macaque FK88 at 226 days post infection) infected *RhMs* are shown. ToPro3 stains cell nucleus. Scatter plots of LGR5+ **(B)** and HD5+ **(C)** cells are shown for jejunum in normal (n=11), acutely (n=10) and chronically (n=10) SIV-infected *RhMs*. An average 19-20 fields was randomly selected for each animal. The number of positive LGR5+ or HD5+ cells in each field was counted. Each point in the scatter plot represents the average number of positive cells from all the fields of each animal. The larger horizontal line denotes the mean frequencies (± standard deviation) of each category. Statistically significant differences of LGR5+ or HD5+ cell frequency among normal and SIV infected *RhMs* determined with one way ANOVA followed by Tukey-Kramer multiple comparison analysis as indicated with asterisks (*p < 0.05).

LGR5+ cells were localized to the crypt base columnar cells in normal formalin-fixed paraffin-embedded jejunum tissue (**Figures 3A** and **Supplementary Figures 3A–C**). Both LGR5+ and HD5+ cells were localized side by side where HD5 cells intersperse with LGR5 cells at the base of the crypts (**Supplementary Figure 3A**). Colocalization of LGR5+ cells with cytokeratin (cytokeratin+LGR5+ cells in yellow) was also detected as well as cell-to-cell adhesion molecule (E-cadherin+LGR5+ cells in yellow) (**Supplementary Figures 3B, C**), consistent with the epithelial cell lineage of ISCs.

To determine the impact of SIV infection on ISCs, the frequency of LGR5+ cells in the jejunum of SIV-infected and uninfected *RhMs* was determined (**Figure 3B**). No significant differences were detected in the number of LGR5+ stem cells due to the effect of SIV infection. A slight decrease in LGR5+ stem cell population was detected during acute infection (mean ± the standard deviation, 54.4 ± 36.5 cells/mm^2^ and 31.6 ± 12.4 cells/mm^2^ for uninfected-normal and acute SIV *RhMs*, respectively; *p* = 0.244). However, the frequency of LGR5+ cells was similar to normal levels during chronic SIV infection (mean ± the standard deviation, 53.7 ± 42.9 cells/mm^2^). There was no significant increase in LGR5+ cells during chronic infection compared to acute infection (*p* = 0.343), and there was no significant difference in the frequency of LGR5+ cells between normal SIV-uninfected and chronically SIV-infected *RhMs* (*p* = 0.962). HD5+ cells (PCs) were detected in the crypt base in normal as well as SIV-infected jejunum (**Figures 3A** and **Supplementary Figure 3D**). To determine the impact of SIV on PCs, HD5+ cells were quantified in both SIV-infected and uninfected *RhMs*. A significant increase in HD5+ cells was detected during acute SIV infection (mean ± the standard deviation, 130.3 ± 20.5 cells/mm^2^ and 156.3 ± 22.0 cells/mm^2^ for uninfected-normal and acute SIV *RhMs*, respectively; *p* = 0.026). In addition, HD5+ cells were also increased in chronic SIV infection (mean ± the standard deviation, 157.9 ± 25.5 cells/mm^2^; *p* = 0.018) when compared to the values from uninfected normal jejunum tissues (**Figure 3C**). There were no significant differences in the HD5+ cells between acute and chronically SIV-infected *RhMs*. No significant positive or negative correlation between LGR5+ cells and HD5+ cells was detected (*p* = 0.96). More male animals were represented in the uninfected group (10/17) than in the acute (1/10) and chronic (1/13) SIV-infected group, and we compared LGR5+ and HD5+ cells on jejunum tissues from male and female SIV-uninfected control animals to determine whether there are any differences in LGR5 and HD5 expression between male and female *RhMs* (**Table 1**). However, we did not observe any significant differences in cell population/mm^2^ for either LGR5 (*p* = 0.354) or HD5+ (*p* = 0.251) cells (**Supplementary Figures 4A, B**).

### Reduced Cell Adhesion Molecule E-Cadherin and Increased Proliferation Detected in SIV Infection

**Figure 4:**
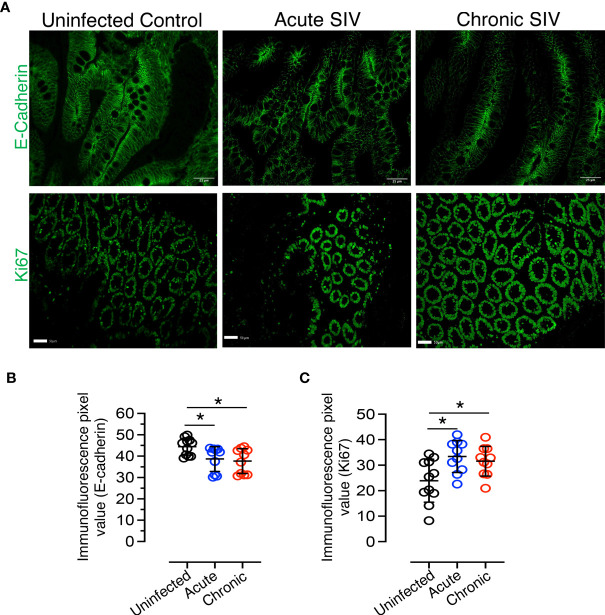
Reduced E-cadherin and increased proliferation markers detected during SIV infection in *RhM* jejunum tissue. **(A)** Top panel: Representative E-cadherin expression in normal uninfected (macaque number GT20), acutely (macaque M992), and chronically (DE50) SIV-infected *RhMs*. Bottom panel: Representative Ki67+ staining is shown for normal (macaque GT20), acutely (M992), and chronically (DE50) SIV-infected *RhMs*. Scatter plots of E-cadherin **(B)** and Ki67+ **(C)** immunofluorescence pixel values for normal (n=11), acutely (n=10), and chronically (n=10) SIV-infected *RhMs*. The expression intensity of E-cadherin and Ki67 protein was quantified in 19-20 randomly selected fields/region of interest (ROI) for each animal and the intensity in each selected field/ROI was quantified. Each point in the scatter plot represents the average of quantified intensities for relevant cellular markers of each animal. The larger horizontal line denotes the mean frequencies (± standard deviation) of each category. Statistically significant differences of E-cadherin+ or Ki67+ staining among SIV-infected and uninfected *RhMs* are shown as analyzed with ANOVA followed by Tukey-Kramer multiple comparison (*p < 0.05). A *p*-value < 0.05 was considered significant.

E-cadherin, the core membrane protein of the AJ protein family in jejunum tissues, is responsible for regulating intestinal homeostasis and barrier function. To understand the impact of SIV infection on E-cadherin expression in epithelial region, we analyzed the expression of E-cadherin in jejunum tissues and found it was significantly reduced in both acutely (*p* = 0.032, mean of MFI ± standard deviation, 38.7 ± 5.8) and chronically (*p* = 0.012; mean of MFI ± standard deviation, 37.7 ± 5.8) SIV-infected *RhMs* compared to uninfected controls (mean of MFI ± standard deviation, 44.4 ± 3.9; **Figures 4A**, **B**). Ki67, a marker of cell proliferation, was significantly increased in jejunum from both acute (mean of MFI ± the standard deviation, 33.4 ± 6.3) and chronic (mean of MFI ± standard deviation, 31.6 ± 5.8) SIV infection compared to the uninfected controls (mean of MFI ± the standard deviation, 23.9 ± 8.4; *p* = 0.012 and *p* = 0.047, respectively) (**Figures 4A, C**). Since no epithelial marker was used along with Ki67 to measure epithelial cell induced cell proliferation, ROIs were carefully drawn in the epithelial region in order to avoid the proliferation normally occurring in Peyer’s patches or other gut associated lymphatic tissue (**Supplementary Figure 5**). Pearson correlation coefficient analysis of E-cadherin and Ki67 MFI values demonstrated that no significant correlation existed between the expression levels of E-cadherin and Ki67+ proliferating cells in jejunum tissue (*p* = 0.965), indicating that despite enhanced cell proliferation the disrupted barrier function could not be repaired in SIV infection.

### Identification of Differentially Expressed Genes (DEGs) in Enteroids From Normal and Chronically SIV-Infected *RhMs*

**Figure 5:**
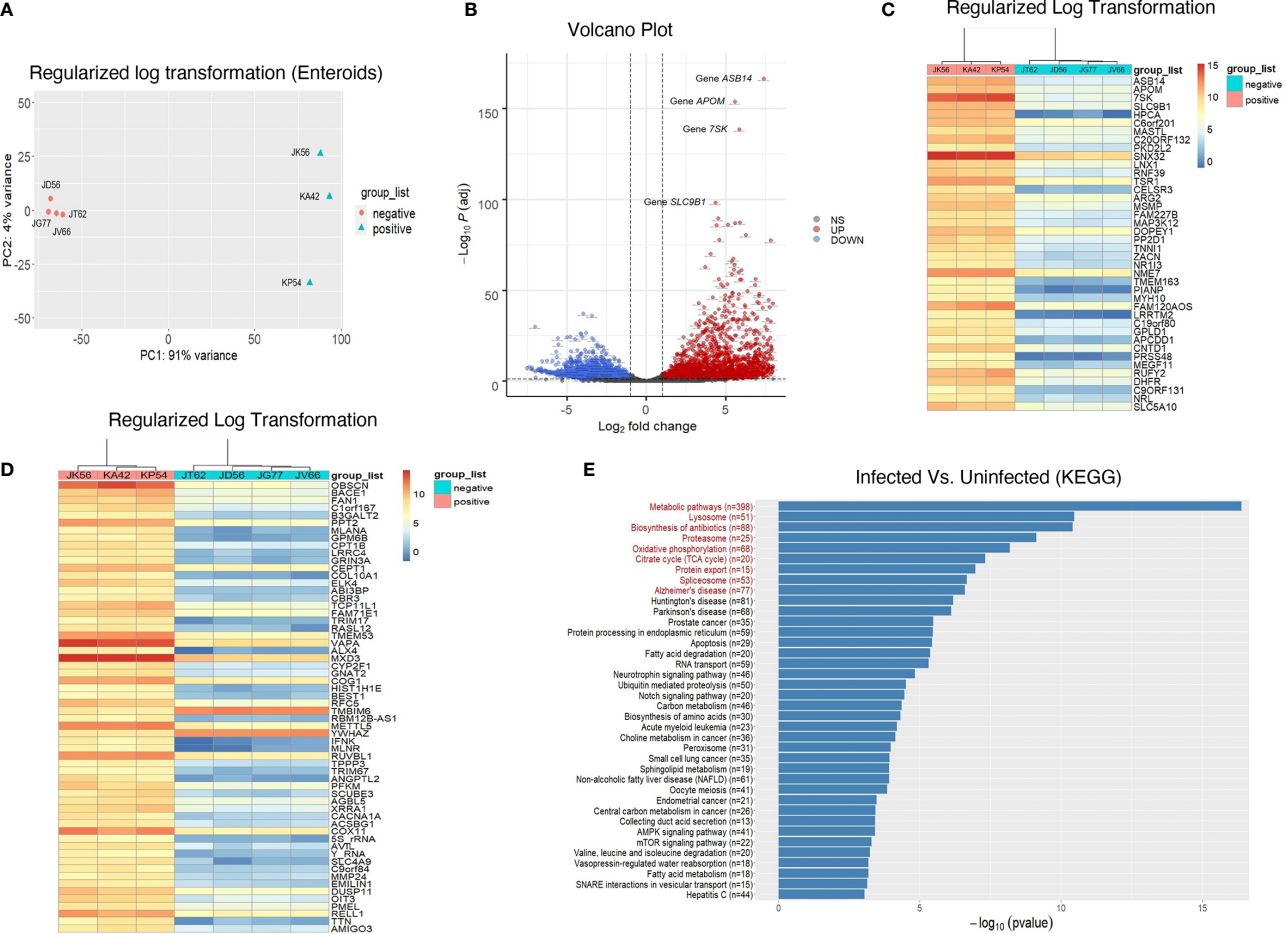
Transcriptomic profiling of rhesus enteroids isolated from chronically SIV-infected and SIV-uninfected *RhMs*. **(A)** PCA plot of 4 normal SIV-uninfected (JT62, JD56, JG77, and JV66) and 3 chronically SIV-infected (JK56, KA42, and KP54) *RhMs* show the characteristics of samples according to the gene expression levels. **(B)** Volcano plot of the upregulated and downregulated DEGs of SIV-infected and uninfected RhMs. The X-axis and Y-axis represent the log_2_ fold change and negative log_10_ adjusted p-values, respectively. Genes with an FDR of less than 0.05 found with DEseq2 were assigned as differentially expressed. Red and blue dots indicate significantly upregulated and downregulated genes, respectively. Heatmaps of read counts of the top 40 **(C)** and next 60 genes **(D)** from enteroids which have the smallest adjusted p-values and the regulation function using the shifted logarithm transformation. **(E)** The results for the pathway enrichment analysis based on KEGG database. There are 38 significantly enriched pathways whose p-values were <0.05 without the correction of multiple comparisons. The red enriched pathways indicate that those pathways are enriched with the p-value adjusted by the Benjamini-Hochberg (BH) FDR below the significance level of 0.05.

Despite there was significantly decreased E-cadherin expression, we were unable to detect any statistically significant difference in LGR5+ stem cell population in jejunum between uninfected and chronically SIV infected *RhMs*. To determine whether LGR5+ cells during chronic SIV infection was able to maintain intestinal homeostasis and iEC regeneration as observed in SIV-uninfected ISCs, we compared enteroids from chronically SIV-infected *RhMs* with enteroids derived from uninfected animals and studied the transcriptomic profiling of ISCs (SRA accession number PRJNA765696). On average about 15-20% of the crypts were able to generate well and developed mature enteroids. We monitored the development of enteroids isolated from both uninfected and chronically SIV infected *RhMs* microscopically on a regular basis and did observe any differences in budding stages in enteroids between those two groups under the same culture condition (**Supplementary Figure 6**). RNA was isolated from enteroid cultures grown from four SIV uninfected and three chronically SIV-infected *RhMs* and subjected to RNA-seq. In all our subsequent analyses we have compared primary culture enteroids from the uninfected control and chronically SIV-infected *RhMs*. PCA of the variable transcripts after DESeq2 regularized log transformation revealed a clear distinction between SIV-infected *RhMs* and uninfected controls along the first principal component (PC1) with 91% and the second principal component (PC2) with 4% of the total variance (**Figure 5A**).

The resulting number of significant DEGs with the official gene symbols (n=5293; **Supplementary Table 3**) was used for further downstream analysis. A majority of these DEGs were downregulated in response to SIV infection (2900, compared to 2393 that were upregulated). The volcano plot shows the upregulated (red) and downregulated (blue) DEGs of SIV-infected *RhMs* and uninfected controls (**Figure 5B**). We performed hierarchical clustering analysis of the regularized log transformed gene expression data of the top 40 DEGs (**Figure 5C**) and the next 60 DEGs (**Figure 5D**). Our top 100 DEGs denoted upregulation of some important genes associated with inflammatory diseases of the gut, like Crohn’s disease and ulcerative colitis, as well as carcinogenesis (**Supplementary Table 3**). These included ARG2, DHFR, ANGPTL2, SCUBE3, AVIL, EMILIN1 and DUSP11.

### Identification of KEGG-Enriched Pathways in SIV-Infected and Uninfected *RhM* Enteroids

There were 1789 DEGs enriched in 284 pathways in the KEGG database which contained at least two DEGs (**Supplementary Table 4**). Among all the pathways, 38 were significantly enriched (*p* < 0.05) without the correction of multiple comparisons, and 9 pathways (red) with the p-value adjusted by the BH FDR (*p* < 0.05) (**Figure 5E** and **Supplementary Table 4**). The latter included the metabolic pathway (mcc01100; *p* = 7.69E-08), mitochondrial pathway (namely, oxidative phosphorylation (OXPHOS)) (mcc00190; *p =* 2.79E-04), tri-carboxylic acid/TCA cycles (mcc00020; *p =* 6.68E-04), apoptosis (mcc04210; *p* = 0.004), Notch signaling pathway (mcc04330, *p =* 0.012), AMPK signaling pathway (mcc04152, *p*=0.033) and mTOR signaling pathway (mcc04150, *p* = 0.037) (**Supplementary Table 4**).

### Dysregulation of Various Metabolic Pathway Associated Genes in Enteroids From SIV-Infected *RhMs*

The balance between proliferation and differentiation of ISCs to maintain homeostasis is controlled by several external and internal factors. Availability of cytokines, growth factors, and different nutrients are considered as important external factors in regulating intestinal homeostasis. Hence, impairment of nutrient metabolism pathways could impact intestinal stemness. KEGG pathway analysis revealed the metabolic pathway in enteroids from SIV-infected *RhMs* as the highest significantly enriched pathway compared to enteroids from SIV-uninfected *RhMs*. A total of 398 DEGs were associated with metabolic pathways, out of which 298 and 100 DEGs were downregulated and upregulated, respectively (**Supplementary Tables 4**–**6**). In the glycolysis/gluconeogenesis pathway, we identified 29 DEGs, of which 23 genes were downregulated (ACSS2, PDHA1, ADH1A, PGAM1, PDHB, ENO2, HK1, ALDH3A2, MINPP1, ALDH1A3, LDHA, PFKL, PGK1, ALDOC, ALDOB, DLAT, ACSS1, ALDOA, DLD, GAPDH, PGM1, PCK2, ALDH9A1) and only 6 genes were upregulated (ADH4, GAPDHS, ADH7, PFKM, GCK, LDHAL6B). Sixteen DEGs in the pyruvate metabolism pathway were detected, where 15 genes (FH, ACSS2, PDHA1, MDH1, GLO1, PDHB, ALDH3A2, LDHA, LDHD, HAGH, DLAT, ACSS1, DLD, PCK2, ALDH9A1) were downregulated and only 1 gene (LDHAL6B) was upregulated. We noted 18 DEGs in fatty acid metabolism, of which 13 genes (PECR, ACAA2, OXSM, ACSL4, MCAT, ACADSB, HADHB, HADHA, PPT1, ACOX3, ACADM, HADH, ACADS) were downregulated and 5 genes (ELOVL5, ACSBG1, PPT2, CPT1B, ACSBG2) were upregulated. All DEGs (BDH2, HMGCS1, BDH1, OXCT1) involved in ketone body metabolism were downregulated. The AMPK signaling pathway, which regulates intestinal barrier function (40) and various energy metabolism pathways (41), was also significantly dysregulated in SIV-infected enteroids compared to uninfected enteroids. A significant downregulation of PRKAB1 and PRKAG1 genes, which encode β and γ subunits of master regulator enzyme AMPK, were detected in enteroids from SIV-infected *RhMs* (**Supplementary Table 4**).

### Downregulation of DEGs Encoding Proteins of OXPHOS and TCA Cycle in Enteroids From SIV-Infected *RhMs* Suggests Mitochondrial Stress

**Figure 6:**
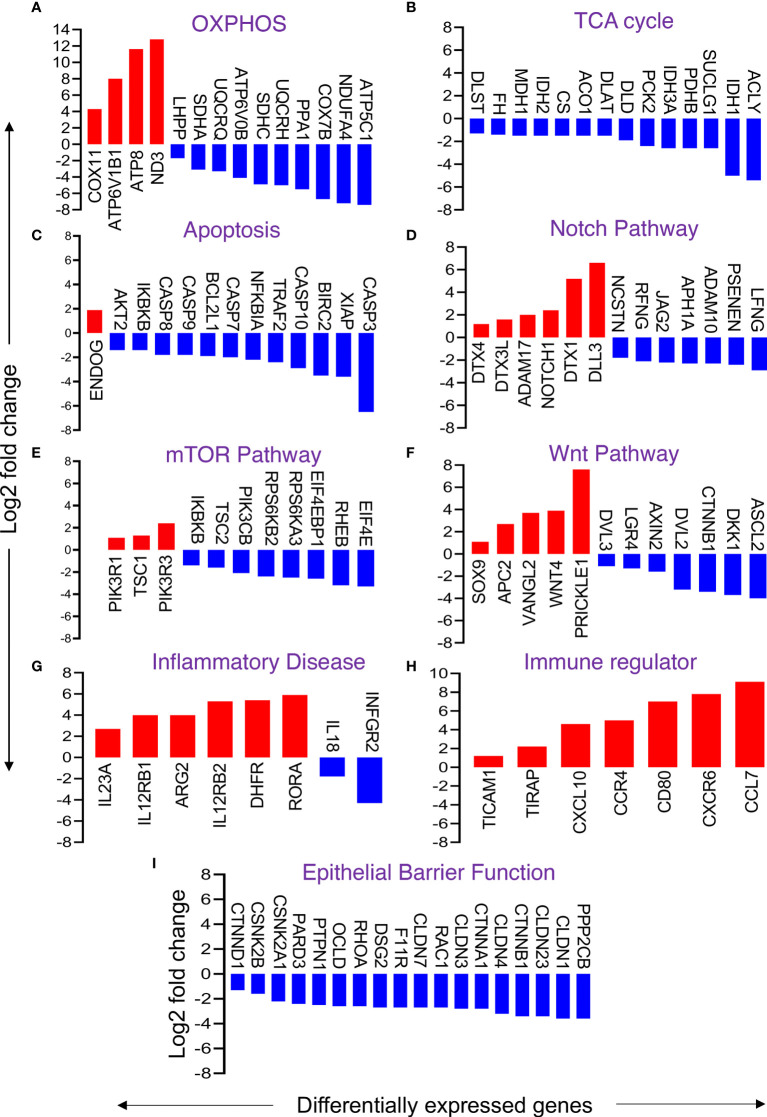
Changes in selective gene expression of primary enteroids isolated from chronically SIV-infected *RhMs* when compared to SIV-uninfected *RhMs*. Representative differentially expressed genes responsible for oxidative phosphorylation (OXPHOS) **(A)**, TCA cycle **(B)**, apoptosis **(C)**, Notch pathway **(D)**, mTOR pathway **(E)**, Wnt pathway **(F)**, inflammatory disease **(G)**, immune regulator **(H)**, and gut epithelial barrier function **(I)** are shown. Log(2) fold change in gene expression for upregulated and downregulated genes are shown by red and blue bars, respectively.

The metabolism of different nutrients is the sum of catabolic and anabolic reactions resulting in the production of byproducts that provide energy to drive various cellular processes, including ATP. Glycolysis, TCA cycle, and OXPHOS are among the ATP-generating pathways. KEGG analysis showed that the majority of DEGs associated with TCA and OXPHOS pathways were downregulated. Fifty-five out of 68 DEGs (nearly 81%) of OXPHOS were downregulated (**Figure 6A** and **Supplementary Table 4**). Notable downregulated genes included COX (-7B, 4I1, 8A, 6B1, 7A2L, etc.), NDUF (-A10, A12, C2, S8 etc.), ATP (-5C1, 5G3, 5G1, 5F1, etc.), ATP6V (-0B, 0E1, 0D1, 1G1, etc.), SDH (-A, B, C), UQCR, UQCR (-C1, C2, H etc.), LHPP, and PPA1. Upregulated genes in enteroids from SIV-infected *RhMs* included ND (-1 to 6), ATP (-4A, 4B, 6, 8), ATP6V1B1, and COX11. Interestingly, all DEGs (100%) related to TCA cycles were downregulated. Downregulated genes were FH, PDHA1, MDH1, IDH (-1, 2), DLST, PDHB, CS, ACLY, SUCL (-A2, G1, G2), ACO1, DLAT, DLD, PCK2, and IDH3A. (**Figure 6B** and **Supplementary Table 4**). Since mitochondrial metabolisms and signaling pathways play pivotal roles in maintaining the homeostasis, proliferation, differentiation and stemness of IECs, our observation may indicate loss of ISC functions due to mitochondrial metabolic stress in enteroids from chronically SIV-infected *RhMs*.

### Genes Encoding Apoptosis Inhibitors Were Downregulated in Enteroids From SIV-Infected *RhMs*

Apoptosis of iECs and loss of TJ proteins are characteristic of SIV infection (7). Transcriptomic analysis revealed 29 DEGs involved in apoptosis, among which 26 DEGs were downregulated and 6 DEGs were upregulated. Notable downregulated genes include XIAP, IKBKB, AKT2, TRAF2, NFKBIA, BIRC2, and BCL2L1 (**Figure 6C** and **Supplementary Table 4**). These genes encode proteins that negatively regulate the expression of apoptosis genes. Thus, downregulation of these genes suggests uninhibited apoptosis processes. Further, we also detected upregulation of the ENDOG gene, which induces DNA fragmentation and cell apoptosis. However, we also observed downregulation of genes encoding different caspase proteins like CASP9, CASP7, CASP8, CASP10, and CASP3 that might play a role in compensating excess cellular apoptosis and overall increased cell proliferation.

### Significant Changes in the Notch Signaling Pathway Detected in Enteroids From SIV-Infected *RhMs*

The Notch signaling pathway regulates the proliferation and differentiation of ISCs. We detected 20 DEGs relevant to the Notch signaling pathway in enteroids from SIV-infected *RhMs*. Important downregulated genes were NCSTN, RFNG, JAG2, APH1A, ADAM10, PSENEN, and LFNG, while upregulated genes were ADAM17, NOTCH1, DTX3L, DTX (1 and 4), and DLL3 (**Figure 6D** and **Supplementary Table 4**). The data suggest that SIV pathogenesis during chronic infection leads to dysregulation of the Notch pathway, which might have a significant impact on ISC regeneration, proliferation, and function.

### Disruption of mTOR Signaling Pathway in Enteroids From SIV-Infected Animals

The mTOR signaling pathway regulates a number of cellular processes like autophagy, protein biosynthesis, lipid biosynthesis, cell survival, and growth factor signaling. We identified several upregulated genes including TSC1, PIK3R1, and PIK3R3 that were involved in the mTOR pathway. Several downregulated genes were also detected including PIK3CB, TSC2, IKBKB, RPS6KA3, RPS6KB2, RHEB, EIF4E, and EIF4E2 (**Figure 6E** and **Supplementary Table 4**).

### Selected Genes Encoding Key Proteins of the Wnt Signaling Pathway Were Differentially Expressed in Enteroids From SIV-Infected *RhMs*

Some of the DEGs encoding key proteins of the Wnt signaling pathway were significantly downregulated or upregulated. Downregulation of some positive regulator encoding genes (e.g., CTNNB1, DVL (-2, 3), LGR4, ASCL2) and upregulation of negative regulator encoding genes (e.g., APC2 and SOX9) were detected (**Figure 6F** and **Supplementary Table 4**). We also noted upregulation of WNT4, VANGL2, and PRICKLE1 genes, which encode proteins that activate the Wnt signaling pathway, and downregulation of AXIN2 and DKK1 genes, which encode pathway inhibitory proteins. Upregulation of genes encoding activator proteins and downregulation of inhibitory proteins suggest ISCs are Wnt dependent and showed an effort to restore normal Wnt signaling pathway function during chronic SIV infection.

### Transcriptomic Changes in Intestinal Inflammation, Immune Regulator, and Gut Barrier Functions in Enteroids From SIV-Infected *RhMs*

Intestinal inflammatory disease-related DEGs were either upregulated (IL12R (B1, B2), IL23A, ARG2, DHFR and RORA) or downregulated (IFNGR2 and IL18) in enteroids derived from chronic SIV-infected *RhMs* compared to enteroids derived from SIV-uninfected control *RhMs* (**Figure 6G**). Several DEGs (TICAM1, TIRAP, CXCL10, CCR4, CCR7, CD80, and CXCR6) associated with immune regulation were also upregulated (**Figure 6H**). We noted 18 out of 25 DEGs encoding TJ proteins and regulatory proteins were downregulated (**Figure 6I** and **Supplementary Table 4**). Similarly, 15 of 22 DEGs encoding proteins of AJ were downregulated (**Supplementary Table 4**). Notable downregulated genes encoding epithelial barrier regulatory proteins CLDN (-1, 3, 4, 7, 23), OCLN, PARD3, CTNNB1, CTNND1, CTNNA1, F11R, RHOA, DSG2, PPP2CB, PTPN1, CSNK2A1, CSNK2B, CDX2, and RAC1 (**Figure 6I** and **Supplementary Table 3**).

### GO Functional Profiling Changes in Enteroids From SIV-Infected *RhMs*

Molecular function (MF), cellular components (CC), and biological processes (BP) of DEGs showed significant transcriptional changes in enteroids from SIV-infected *RhMs* compared to enteroids from uninfected *RhMs*. Under the MF category, 5293 DEGs were annotated, of which 2393 DEGs were upregulated and 2900 DEGs were downregulated. The results of molecular function GO analysis revealed significant upregulation of calcium ion binding GO terms and 11 significantly downregulated GO terms, namely, poly(A) RNA binding, GDP binding, GTP binding, translation initiation factor activity, structural constituent of ribosome, GTPase activity, mRNA binding, electro carrier activity, and fatty-acyl-CoA binding (**Table 2** and **Supplementary Tables 7**, **8**). The CC category also provided two significantly upregulated GO terms: integral component of plasma membrane and voltage-gated calcium channel complex (**Table 2**). Twenty-five significantly downregulated GO terms were detected under the CC category including extracellular exosome, mitochondrion, membrane, focal adhesion, mitochondrial inner membrane, lysosome, endoplasmic reticulum, lysosomal membrane, nucleolus, and intracellular ribonucleoprotein complex (**Table 2** and **Supplementary Tables 9**, **10**). Similarly, one significantly downregulated GO term “proteolysis involved in cellular protein catabolic process” in the BP category was detected (**Table 2** and **Supplementary Tables 11**, **12**).

**Table 2:** Significantly Enriched GO Terms For Differentially Expressed Protein Coding Genes.

Category	GO Term	Overlap DEG Count	FDR
**Molecular Function** **(MF)**	**Upregulated DEGs**		
	calcium ion binding	97	0.045704
	**Downregulated DEGs**		
	poly(A) RNA binding	259	1.36E-24
	GDP binding	21	4.71E-05
	GTP binding	83	6.78E-04
	translation initiation factor activity	30	0.001869
	structural constituent of ribosome	82	0.005375
	GTPase activity	45	0.005417
	cysteine-type endopeptidase activity	20	0.005417
	nucleotide binding	71	0.016379
	mRNA binding	29	0.018816
	electron carrier activity	19	0.027385
	fatty-acyl-CoA binding	12	0.027385
**Cellular Component (CC)**	**Upregulated DEGs**		
	integral component of plasma membrane	118	0.019303
	voltage-gated calcium channel complex	10	0.033808
	**Downregulated DEGs**		
	extracellular exosome	623	1.01E-54
	Mitochondrion	217	4.37E-12
	Membrane	230	6.39E-10
	focal adhesion	96	8.67E-10
	Cytosol	217	2.16E-09
	mitochondrial inner membrane	57	4.85E-07
	Ribosome	48	9.56E-07
	nucleoplasm	281	6.65E-06
	Lysosome	42	2.58E-05
	perinuclear region of cytoplasm	86	2.76E-05
	endoplasmic reticulum	116	7.85E-05
	catalytic step 2 spliceosome	31	3.98E-04
	lysosomal membrane	45	3.98E-04
	Nucleolus	131	6.26E-04
	intracellular ribonucleoprotein complex	30	0.001013
	endoplasmic reticulum membrane	62	0.001917
	lipid particle	20	0.002189
	myelin sheath	37	0.009125
	Arp2/3 protein complex	8	0.011275
	nuclear envelope	23	0.017746
	Midbody	28	0.021276
	eukaryotic translation initiation factor 3 complex	9	0.025019
	spliceosomal complex	17	0.034287
	viral nucleocapsid	21	0.040989
	cytosolic small ribosomal subunit	23	0.044681
**Biological Process (BP)**	**Downregulated DEGs**		
	proteolysis involved in cellular protein catabolic process	22	2.91E-04

DEG, Differentially expressed gene; FDR, False discovery rate; GO, Gene ontology.

## Discussion

HIV-associated gastroenteropathy is a major contributor to the symptomology of HIV (42). The impairment of the intestinal mucosal barrier with aberrant iEC regeneration starts very early during acute HIV/SIV infection (7, 43). Understanding the aberrant iEC regeneration in HIV/SIV might lead to an alternative approach for the treatment of HIV-mediated enteropathy based on enhancing iEC repair mechanisms. A continuous supply of LGR5+ crypt columnar intestinal stem cells is essential for the maintenance of healthy small intestine epithelium renewal and regeneration (44). However, the impact of HIV/SIV infection on LGR5+ stem cells is not well defined. We observed for the first time that the putative LGR5+ ISCs are not significantly depleted during SIV infection. However, SIV infection is accompanied by a loss of tight junction protein, increased epithelial cell proliferation, and increased numbers of HD5+ cells which suggests aberrant regeneration and differentiation of LGR5 positive cells. Despite the lack of significant reduction in LGR5+ stem cells, we have observed significantly increased iEC apoptosis and loss of ZO-1 expression during SIV infection as reported in our earlier study (7). The loss of iECs during SIV infection does not positively correlate with the crypt cell population. It may be possible that LGR5+ or TA cell proliferation represents an attempt to compensate for iEC apoptosis, but it failed to provide subsequent differentiation, reconstitution and regeneration of these cells that are necessary for iEC repair. It may also be possible that the LGR5+ cells have been replenished by the transit amplifying cells at +4 position or some other absorptive and secretory progenitor cells (45–47). We have previously shown that in response to infection in the small intestine, TA cells proliferate and more PCs can be found in a mouse model of *Salmonella* Typhimurium infection (48). Our study suggested that 46% of the LGR5+ cells were CD24-CD44+CD166+. However, around 54% of the LGR5+ cells were either negative or expressed different combinations of CD24, CD44, and CD166 surface markers. This might be representative of different functional subtypes that we will explore in the future. We are also interested to know whether SIV/HIV infection may have any preferential targets on some subset of the LGR5+ population, and these directions will be investigated in the future studies.

Intestinal PCs contribute to gastrointestinal innate immunity by secreting antimicrobial peptides including α-defensins such as HD5. HD5 protects the mucosal tissue against invading pathogens and regulates mucosal microbial communities (49). We have detected an increased expansion of small intestinal HD5+ cells in acute and chronically SIV-infected *RhMs*. Upregulation of HD5 in the colorectal mucosa and jejunum has also been reported in patients with HIV-1 and in SIV-infected and simian AIDS *RhMs* (6, 50). The increased production of HD5 during acute and chronic SIV infection might play a compensatory role in regulating mucosal immunity and effector functions to combat virus replication or immune dysregulation in the mucosal environment. Both *in vitro* and *in vivo* studies have demonstrated that PCs play an important role in mediating ISC renewal and regeneration following injury (51). Although HD5+ cells increased during the acute infection preceding the recovery of LGR5+ cells, we did not find any correlation between the frequency of PCs and LGR5+ cells in SIV-infected *RhMs*, suggesting that the expansion of the PC population does not necessarily aids ISCs to regenerate during acute SIV infection. A significant downregulation of some specific stem cell gene signatures (AXIN2 and ASCL2) in enteroids from chronic SIV-infected *RhMs* suggest a plausible loss of stemness of ISCs.

Our crypt isolation method has improved the viability and purity of crypts, enteroid cultures, and subcultured enteroids (32). It has been reported that enteroids retain their structural and functional properties from the original tissue (52, 53). We verified the expression of some important genes like FOXA2, LGR4, LGR5, MUC2, SPON1, OLFM4 and VIL1 in enteroids and compared the results to the gene expression profile of freshly isolated crypts. FOXA2 is a transcription factor that regulates a network of genes important for intestinal epithelial functions and is localized to ISCs and TA cells (54, 55). LGR4 and LGR5 are GPC-receptors expressed on the surface of ISCs. They interact with R-spondin and thus modulate the Wnt-signaling pathway to maintain stem cells (56–58). Recently, it was found that LGR5+ crypt base columnar cells in human small intestine are characterized by high expression of OLFM4; thus, OLFM4 has emerged as a robust marker for human ISCs (59). We included VIL1 and MUC2 genes as a marker for iECs (60) and goblet cells (61), respectively. The successful amplification of these genes from the cDNA generated from the enteroids indicated that both crypts and enteroids possess the characteristics of ISCs which can differentiate to other intestinal cell types. Thus, this model promises to be useful for further studies on the pathogenesis of SIV infection involving ISCs.

In a previous study, we detected iEC apoptosis and the loss of ZO-1 expression during acute and chronic SIV infection in the small intestine (7). Downregulation of apoptosis inhibitor DEGs like XIAP, IKBKB, AKT2, TRAF2, NFKBIA, BIRC2, and BCL2L1 in this study is suggestive of an impaired apoptosis regulatory mechanism in enteroids from chronically SIV-infected *RhMs* (62, 63). Upregulation of the ENDOG gene, which can modulate the caspase-independent apoptotic pathway after cleaving nucleic acids (64), was detected in enteroids from SIV-infected *RhMs*. This may indicate that apoptosis persists even in the absence of upregulated caspase genes. Decreased expression of apoptosis inhibitor genes was also documented in SIV-infected intestinal epithelium (65). The significant loss of E-cadherin expression during acute infection in our study suggests that the loss of E-cadherin expression is not associated with the LGR5+ stem cell population. However, the stable presence of LGR+ cells during chronic infection was not sufficient to restore E-cadherin expression, suggesting a prolonged failure of intestinal barrier function. We have recently shown an increased production of intestinal TGF-β during SIV infection (9), and this increased TGF-β production may have some direct inhibitory effect on E-cadherin expression. A direct *in vivo* and *in vitro* interaction between TGF-β and E-cadherin+ dendritic cells has been documented (66). Failure of intestinal barrier function was also detected from a transcriptomic profile in enteroids grown from chronic SIV-infected *RhMs*. CDX2, a key transcription factor that regulates epithelial differentiation, was also downregulated, and had a major impact in maintaining intestinal barrier function (40).

Transcriptomic analysis of enteroids obtained from SIV-infected *RhMs* revealed 7 upregulated genes that have been reported to be responsible for intestinal inflammatory diseases among the most significant 100 DEGs, namely ARG2, DHFR, ANGPTL2, SCUBE3, AVIL, EMILIN, and DUSP11. Increased ARG2 and DHFR seem to aggravate colitis by altering mucosal immunity (67, 68). On the other hand, upregulated ANGPTL2 has been shown to promote ISC niche and iEC regeneration (69). A few studies have reported the involvement of other genes (SCUBE3, AVIL, EMILIN1, and DUSP11) in intestinal inflammatory diseases (70–73); however, their role in SIV infection remains unclear. Not surprisingly, our transcriptomic analysis revealed downregulation of genes encoding for TJ and AJ proteins in enteroids grown from chronic SIV-infected *RhMs*. Downregulation of claudin-3, 4, and 7 was also reported in other intestinal inflammatory diseases like ulcerative colitis and Crohn’s disease (74, 75). Downregulation of CTNNB1-encoding β-catenin could interfere with the β-catenin-TCF/LEF signaling pathway and affect overall claudin expression (76). Here, we demonstrated downregulation of the majority of genes encoding epithelial barrier proteins, which suggests impairment in the restoration of these barrier proteins in enteroids from SIV-infected *RhMs*.

The role of various metabolic pathways including glycolysis, TCA cycle, OXPHOS, pyruvate metabolism, fatty acid oxidation, and ketone bodies in maintaining the balance between self-renewal and differentiation of ISCs is well documented (77–83). Each metabolic pathway has specific functions which directly or indirectly regulate stem cell proliferation and differentiation. We have detected a significant downregulation of several metabolic pathways (75% of DEGs) in enteroids grown from chronic SIV-infected *RhMs* suggesting impaired metabolic functions in ISCs and PCs during chronic SIV infection. Similarly, we have observed downregulation of DEGs that were responsible for OXPHOS (81%) and TCA cycles (100%), suggesting that mitochondrial stress leads to the failure of proper ISC function during chronic infection. OXPHOS, a critical process for generating ATP, is known to be essential for crypt formation and ISC differentiation by the MAPK p38 signaling pathway (77). Inhibition of OXPHOS in HSP60 knockout ISCs caused differentiation of ISCs into aberrant PCs (84, 85). Changes in mitochondrial genes, proteins, and metabolism have been reported in iECs of intestinal inflammatory diseases (86–88). Downregulation of the majority of the DEGs involved in metabolic pathways in enteroids indicated a massive impact of chronic SIV infection on the metabolism, regeneration, and homeostasis of ISCs and subsequently differentiated cell types. AMPK, a master regulator of cellular energy homeostasis, regulates various energy metabolism pathways, including glucose and fatty acid metabolism (41). Downregulation of regulatory β and γ subunits of AMPK observed in enteroids from chronic infection interfere with AMP binding and energy production (89, 90).

The importance of Notch signaling in regulating intestinal homeostasis has been well documented (91, 92). The presence or absence of Notch signaling determines the fate of ISC differentiation into enterocytes or secretory epithelial cells (93). Upregulation of ADAM17 and downregulation of ADAM10 and JAG2 genes indicate that ligand-independent Notch signaling pathway (LINP) might be the preferred pathway for Notch signaling. The upregulation of NOTCH1 also suggests adaptation of LINP in enteroids from SIV-infected *RhMs*, as ADAM17 can only process NOTCH1 signals (94). Upregulation of DTX1, DTX4, and DTX3L genes in enteroids from SIV-infected *RhMs* accelerates degradation of unbound Notch receptors (95). In contrast, upregulated DLL3 acts as an antagonist and attenuates Notch signaling (96). In the absence of effective Notch signaling, the undifferentiated cells developed into more secretory cells (e.g., PCs and goblet cells) (93, 97, 98). Collectively, our observations suggest a lack of effective Notch signaling, possibly due to upregulation of DLL3 and downregulation of several important genes, which may lead to the generation of increased PCs, lack of enterocyte differentiation, and the loss of gut homeostasis.

The mTOR signaling pathway regulates iEC differentiation (99) and ISC activities through the regulation of cellular metabolic pathways (100, 101). mTOR signaling seems to be ineffective in chronic SIV infection, as different genes (e.g., RPS6KB2, RPS6KA3, EIF4EBP1, and EIF4E) encoding downstream proteins of mTOR signaling were downregulated. Downregulation of the RHEB gene encoding Rheb protein, an important positive regulator of this pathway, also indicated inefficient mTOR signaling and promoted differentiation into secretory cells (PCs). The significant increase in Ki67+ cells in our study can also be correlated with TSC2 downregulation, in agreement with a previous study where increased Ki67+ proliferating cells were detected in the small intestine of TSC2 knockout mice but not wild type mice (102, 103).

Our transcriptomic analysis indicated that enteroids from SIV-infected *RhMs* favored the non-canonical Wnt signaling pathway over the canonical pathway, as we observed downregulation of CTNNB1 encoding β-catenin, a central component of the canonical pathway. Further, we also observed upregulation of APC2 gene encoding a subunit of the APC containing destruction complex which degrades CTNNB1, and downregulation of DVL gene encoding disheveled homolog proteins which counteract against the APC destruction complex (104). Further, upregulation of the non-canonical core protein genes WNT4, VANGL2, and PRICKLE1 indicated adaptation of the non-canonical pathway (105, 106). Upregulation of non-canonical Wnt genes also correlates with increased HD5+ PCs during chronic SIV infection, suggesting ISC priming towards PC differentiation. Our observation agrees with a recent study in which the non-canonical Wnt signaling pathway specifically activated ISCs to differentiate into PCs and enteroendocrine cells by the upregulation of Wnt/planar cell polarity genes such as VANGL2 (106). Upregulation of the SOX9 gene, which encodes Sox9 transcription factor, possibly inhibits ISC proliferation by regulating the Wnt/β-catenin signaling pathway and upregulating PC differentiation as we have observed during SIV infection (107–109). Collectively, the transcriptomic analysis of DEGs involved in signaling pathways and study of protein expression indicated enhanced PC differentiation. This is also supported by the finding that HD5 expression in PCs depends on TCF4, a Wnt-regulated transcription factor (110). Downregulation of genes encoding inhibitor proteins like DKK1 and AXIN2 suggested an attempt to conserve the Wnt signaling pathway and to maintain intestinal homeostasis.

Analysis of DEGs encoding immune and inflammation regulatory cytokines revealed dysregulation of inflammatory disease susceptible genes. IL-12 acts as key mediator of Th1 cell differentiation in human intestinal mucosa and promotes intestinal inflammation. IL-23, a heterodimeric cytokine in the IL-12 family, also promotes intestinal inflammation. Upregulation of IL23a, IL12RB1, and IL12RB2 suggests their role in fostering inflammatory responses during SIV infection. In contrast, downregulation of RORA, IL-18, and IFNGR2 genes seem to counterbalance the intestinal inflammation in enteroids grown from SIV-infected *RhMs*. Significant upregulation of CXCL10, CCR4, CCR7, CXCR6, CD80, TICAM1, and TIRAP has also been reported to be involved in intestinal inflammatory disease directly or indirectly (111, 112), indicating persistent expression of inflammatory cytokines/chemokines in chronic SIV-infected enteroids. Moreover, expression of CXCL10 (113), CCR4 (114), CCR7 (114) CXCR6 (115), CD80 (116), TICAM1 (TRIF (117), and TIRAP (118) in enteroids derived from SIV-infected *RhMs* suggests an attempt to recruit and activate mobile immune cells to establish a barrier against microbial invasion from the intestinal lumen. Significant functional changes were detected in the transcriptome affecting mitochondria, mitochondrial inner membrane, focal adhesion, cellular membrane, and electro-carrier activity, which are involved in metabolic and signaling pathways, apoptosis, barrier functions, absorption, and transportation of nutrients. Defects in molecular processes were also indicated by dysregulated poly(A) RNA binding, translation initiation factor activity, structural constituent of ribosome, and mRNA binding. Collectively, this indicated aberrant cellular components resulting in the loss of normal physiological processes in enteroids from chronic SIV-infected *RhMs*.

## Conclusion

**Figure 7:**
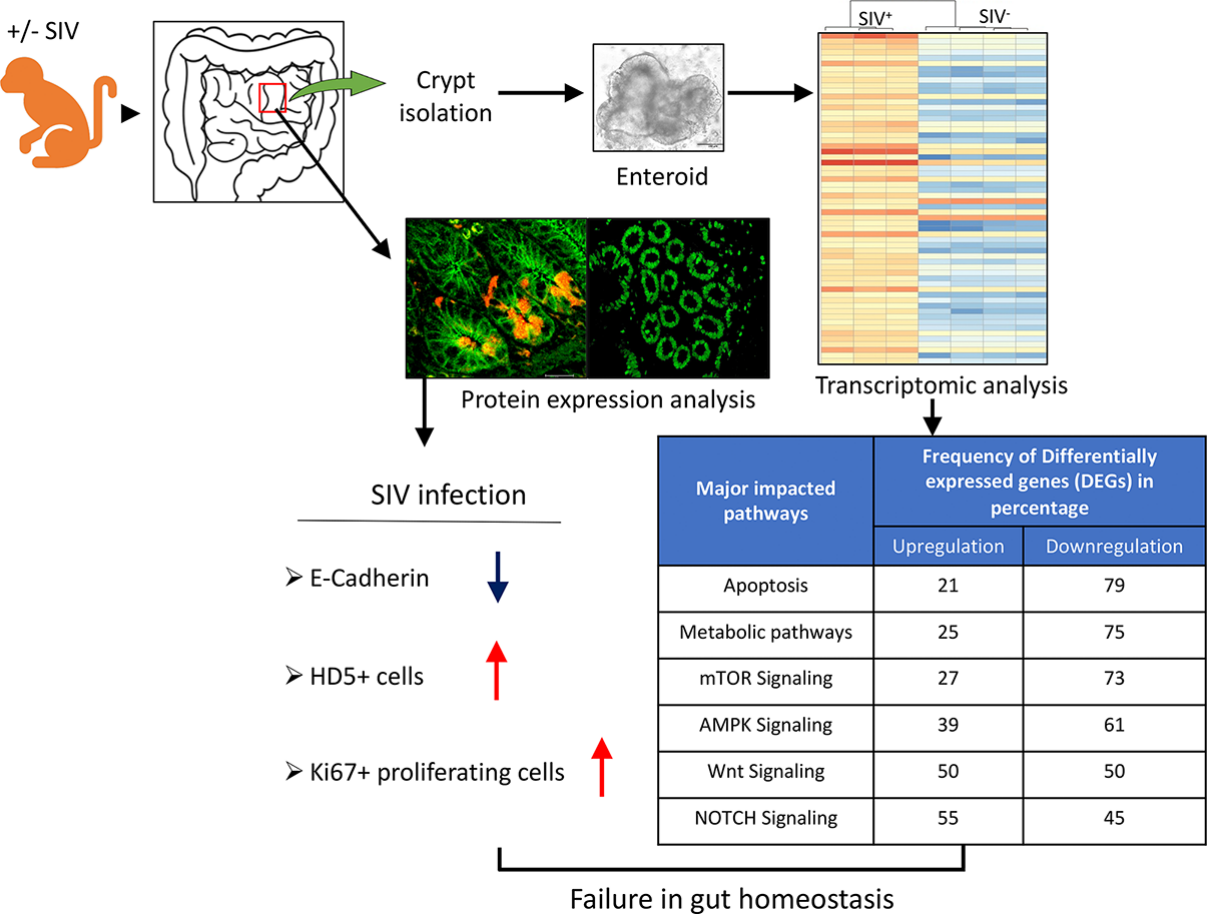
Schematic representation of the main findings. Jejunum tissues from SIV-infected and uninfected *RhMs* were collected. Tissues were either used for protein expression analysis by immunohistochemistry or immunofluorescence or used for crypt isolation for the growth of enteroids and their subsequent characterization and transcriptomic analysis. The protein expression analysis revealed significantly reduced E-cadherin expression indicative of the impairment of intestinal barrier function. In contrast, a significantly increased proliferation of epithelial cells was detected in both acute and chronic SIV-infected *RhMs*, possibly reflecting enhanced transit amplifying cell divisions. A significant increase in HD5+ Paneth cells during acute and chronic stages of SIV infection suggest a compensatory role of these cells in regulating mucosal immunity and effector mucosal function. Transcriptomic analysis showed DEGs involved in different biological pathways necessary for maintaining homeostasis, proliferation, and differentiation of intestinal epithelial cells in enteroids from SIV-infected *RhMs* when compared to SIV-uninfected control *RhMs*. Some of the important pathways and the frequency of upregulated and downregulated differentially expressed genes are shown for enteroids isolated from SIV-infected *RhMs*.

Understanding the molecular mechanisms underlying the disrupted iEC regeneration during SIV infection might lead to an alternative approach for the treatment of HIV-mediated enteropathy. We observed no significant reduction in LGR5+ ISCs in the small intestine during SIV infection compared to normal uninfected macaques. Decreased E-cadherin are thought to be the key features of intestinal enteropathy that is associated with increased gut permeability. Increased Ki67 expression and increased HD5+ cells during acute and chronic SIV infection may signify regenerative attempts to restore homeostasis and combat microbial invasion. Immunophenotyping of LGR5+ cells probing CD24, CD44, and CD166 revealed several subtypes of LGR5+ cells with CD24-CD44+CD166+ being the most frequent subtype. We were successful in developing a protocol to generate *RhM* intestinal enteroids and have characterized these by both cellular and molecular assays. The global transcriptomics of enteroids grown from normal and chronic SIV-infected *RhMs* provided novel information about highly significant changes in metabolic, OXPHOS, and TCA cycle pathways where a majority of the DEGs were downregulated in enteroids derived from SIV-infected *RhMs* compared to enteroids from uninfected control *RhMs* despite no significant changes in LGR5+ stem cell frequencies. This suggests that SIV infection disrupts ISC metabolism and the ATP generation pathway, leading to impaired regeneration capacity and failure to replenish the epithelial barrier during SIV infection. Dysregulation of several intestinal stem cell niche factors including Notch, mTOR, AMPK, and Wnt pathways, and DEGs encoding proteins that drive ISC functions such as ADAM10, ASCL2, AXIN2, CTNNB1, VANGL2, and SOX9, further supports that SIV infection negatively impacts ISCs and intestinal homeostasis. A schematic diagram presenting the cellular and transcriptomic changes in ISCs and enteroids during SIV infection is shown in **Figure 7**. Further studies are needed to understand the role of different phenotypes of LGR5+ cells as well as the molecular changes that occur in enteroids during acute SIV infection.

## Data Availability Statement

The datasets presented in this study can be found in online repositories. The data presented in the study are deposited in the SRA repository, accession number PRJNA765696 (https://www.ncbi.nlm.nih.gov/bioproject/PRJNA765696/).

## Ethics Statement

The animal study was reviewed and approved by Tulane University IACUC.

## Author Contributions

The overall planning, direction and design of the experiment were carried out by BP. NB, AR, BTP, CM, MNS, AD, and BP carried out animal experiments, sample processing and other experiments. BP designed the flow cytometry panels. NB and BP analyzed the flow data. PJD performed the immunohistochemistry data analysis. SKS performed the statistical analysis. XC and QS performed RNAseq data analysis and interpretation. EP provided scientific advice, HD5 polyclonal antibodies and manuscript editing. BP wrote the manuscript with inputs from all authors. All authors contributed to the article and approved the submitted version.

## Funding

The study was supported by National Institutes of Health grant R01DK109883 (BP) and TNPRC base grant P51OD011104.

## Conflict of Interest

The authors declare that the research was conducted in the absence of any commercial or financial relationships that could be construed as a potential conflict of interest.

## Publisher’s Note

All claims expressed in this article are solely those of the authors and do not necessarily represent those of their affiliated organizations, or those of the publisher, the editors and the reviewers. Any product that may be evaluated in this article, or claim that may be made by its manufacturer, is not guaranteed or endorsed by the publisher.

## Supplementary Material

Supplementary Figure 1 Plasma viral loads with means in RhMs infected with pathogenic SIVMAC251 using either intravenous (IV) or intravaginal (IVAG) routes during acute **(A)** and chronic **(B)** phases of infection as determined by real-time PCR. No statistically significant differences in plasma viral load between different routes of infection were detected as analyzed with Mann-Whitney t-test.

Supplementary Figure 2 Amplicons from RT-PCR using the RhM jejunum crypts cDNA (**A**, upper panel) and enteroids cDNA (**B**, lower panel). PCR products were separated by electrophoresis on 1.5% agarose gel and visualized with ethidium bromide staining. Each lane represents the different amplicons resulting from the reactions with gene-specific primers for LGR5 (232bp), OLFM4 (143bp), Vil1 (120bp), MUC2 (113bp), LGR4 (202bp), FOXA2 (270bp), SPON1 (231bp), no cDNA negative control (with GAPDH primers), and GAPDH positive control (120bp). A 50bp DNA ladder was used for reference.

Supplementary Figure 3 Representative immunofluorescence images of the jejunum showing the distribution of LGR5+ cells and HD5 (Paneth cells, **A**), epithelial cells (Cytokeratin, **B**) and adherens junction protein (E-cadherin, **C**) from a normal uninfected RhM. **(A)** The white lines show the orientation of crypts in this jejunum section. **(D)** Distribution of HD5+ cells are shown from a normal SIV-uninfected RhM where the HD5+ Paneth Cells are located at the base of the crypts. Note that the colocalization of LGR5+ cells and epithelial cells (detected by E-cadherin or Cytokeratin) was shown by yellow color (yellow arrows).

Supplementary Figure 4 Scatter plots of LGR5+ **(A)** and HD5+ **(B)** cells are shown for jejunum in male (n=6) and female (n=5) SIV-uninfected control RhMs. The number of LGR5+ and HD5+ cells was quantified from 19-20 randomly selected fields and the number of positive cells in each field was depicted as a single point in the scatter plot. The larger horizontal line denotes the mean frequencies (+/- standard deviation) of each category. No statistically significant differences of LGR5+ or HD5+ cells among male and female macaques were detected with Mann-Whitney T-test.

Supplementary Figure 5 The representative figure depicts the gating of region of interest (ROI) for Ki67 staining. The ROIs were manually drawn in the epithelium from randomly selected crypts (highlighted with yellow, and marked as 1, 2, and 3) for the quantification of Ki67 in epithelial cells.

Supplementary Figure 6 Development of fully grown enteroids isolated from single jejunum crypts on day 13th in both uninfected and chronic SIV infected rhesus macaques.
