# Supplementary figures and images for: Identification, Characterization, and Transcriptional Reprogramming of Epithelial Stem Cells and Intestinal Enteroids in Simian Immunodeficiency Virus Infected Rhesus Macaques
Source: Front Immunol. 2021 Nov 23;12:769990. doi: 10.3389/fimmu.2021.769990 (PMC8650114; doi:10.3389/fimmu.2021.769990)

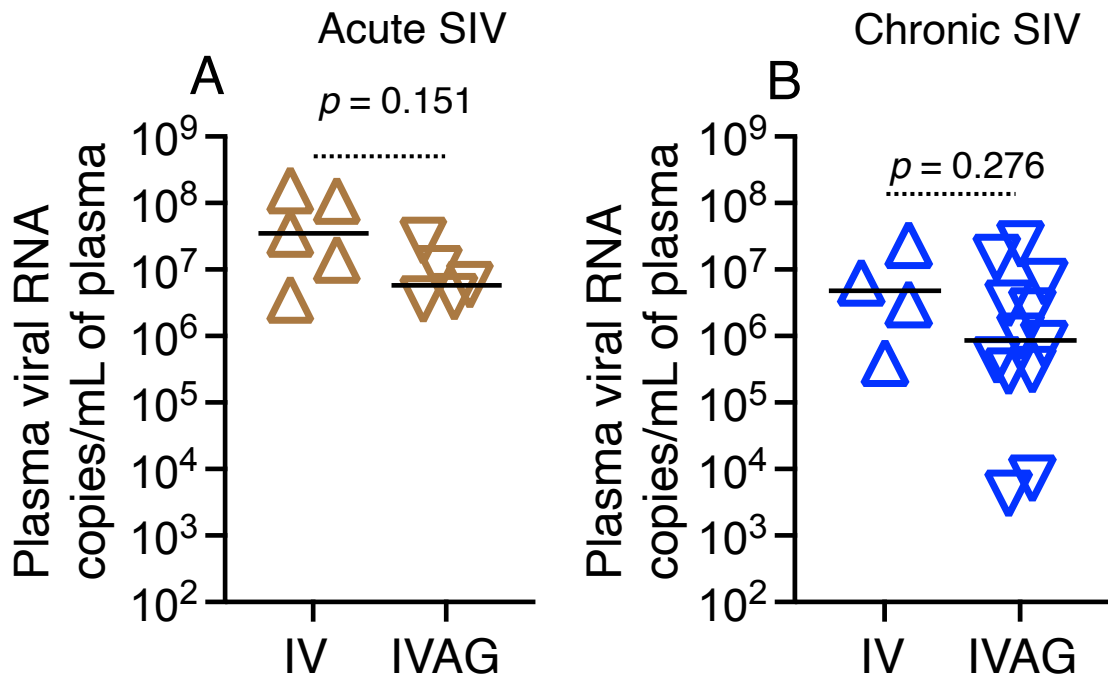

Supplement: Supplementary Figure 1 — Plasma viral loads with means in RhMs infected with pathogenic SIVMAC251 using either intravenous (IV) or intravaginal (IVAG) routes during acute (A) and chronic (B) phases of infection as determined by real-time PCR. No statistically significant differences in plasma viral load between different routes of infection were detected as analyzed with Mann-Whitney t-test. [file Image_1.pdf]

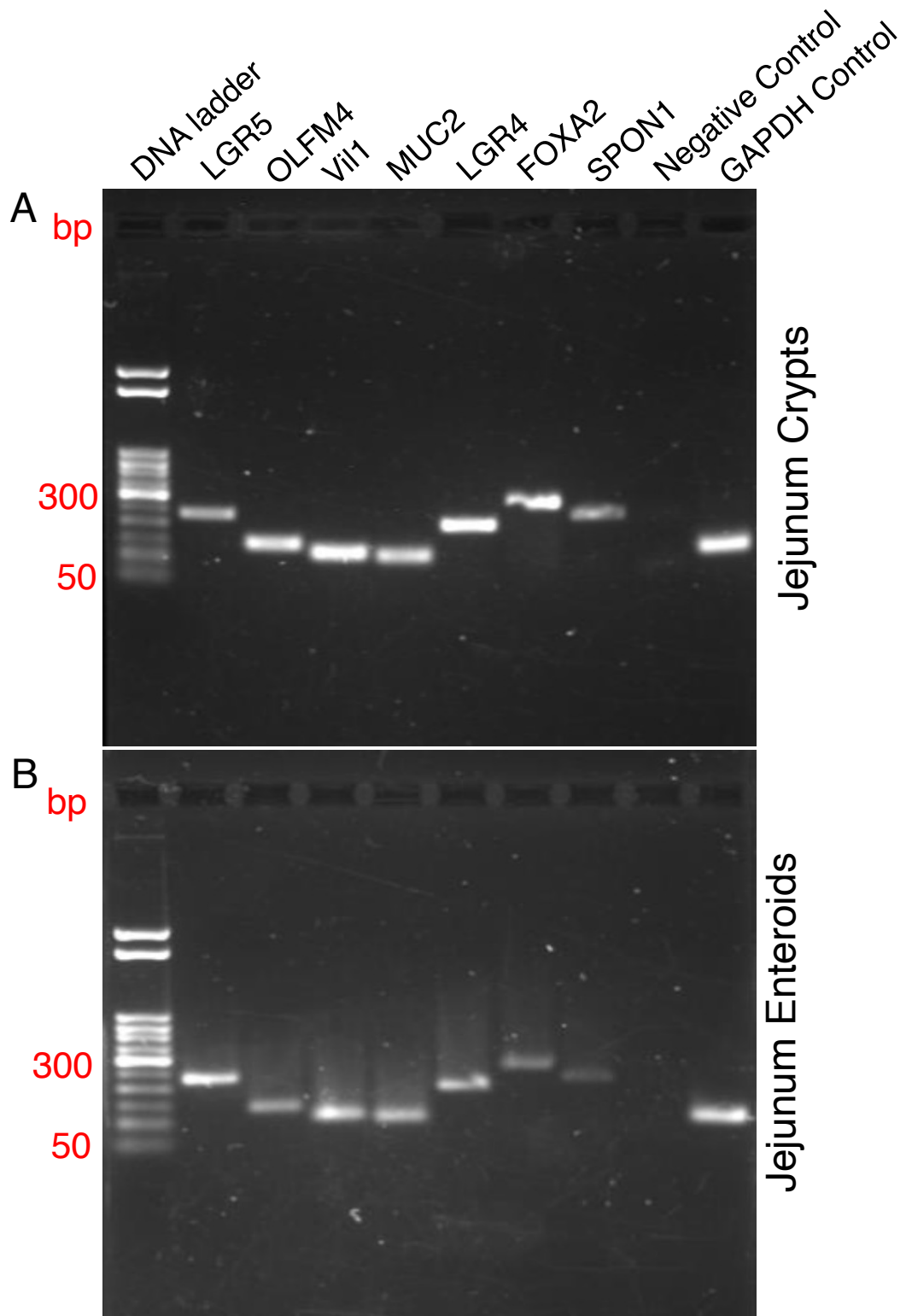

Supplement: Supplementary Figure 2 — Amplicons from RT-PCR using the RhM jejunum crypts cDNA (A, upper panel) and enteroids cDNA (B, lower panel). PCR products were separated by electrophoresis on 1.5% agarose gel and visualized with ethidium bromide staining. Each lane represents the different amplicons resulting from the reactions with gene-specific primers for LGR5 (232bp), OLFM4 (143bp), Vil1 (120bp), MUC2 (113bp), LGR4 (202bp), FOXA2 (270bp), SPON1 (231bp), no cDNA negative control (with GAPDH primers), and GAPDH positive control (120bp). A 50bp DNA ladder was used for reference. [file Image_2.pdf]

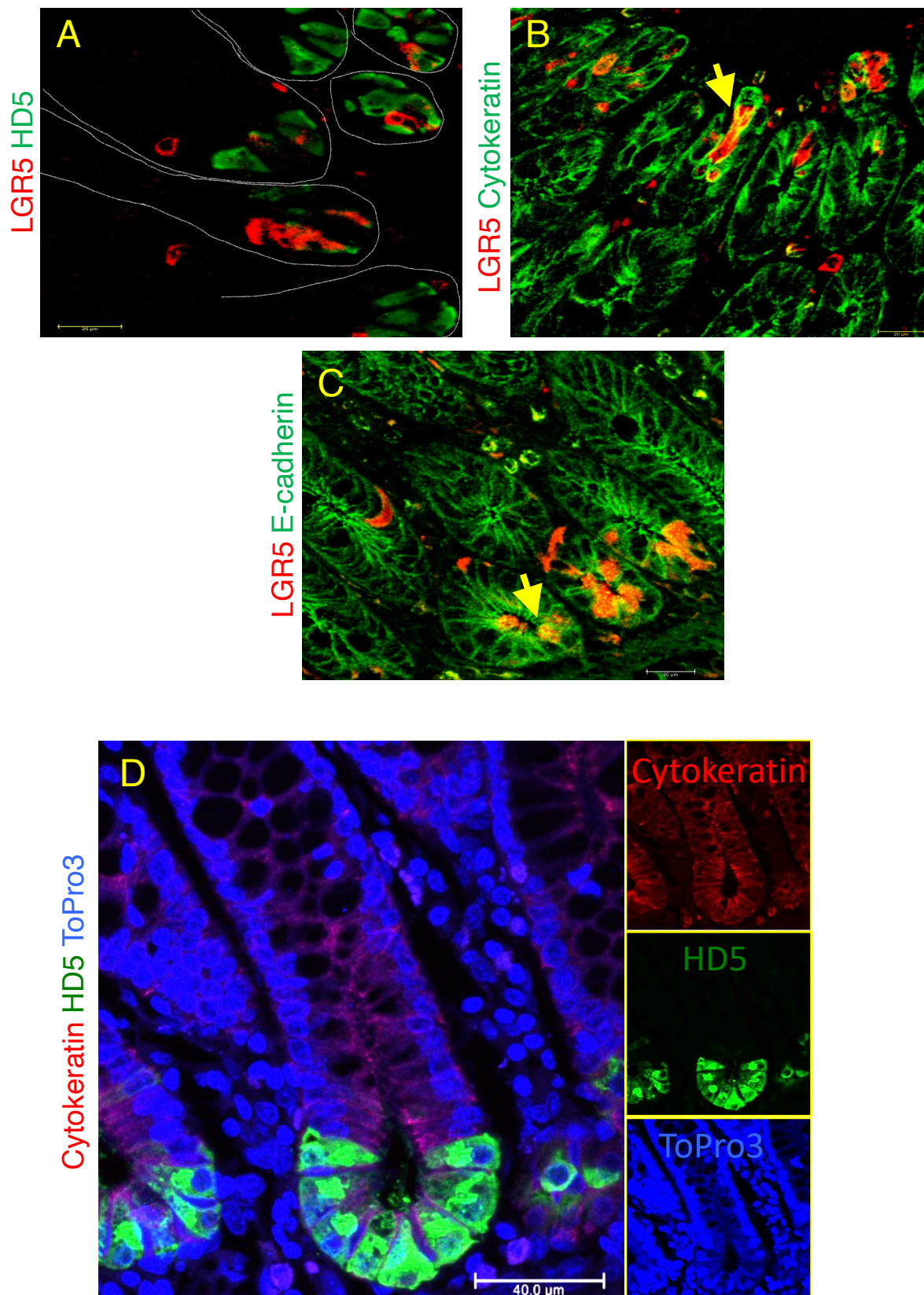

Supplement: Supplementary Figure 3 — Representative immunofluorescence images of the jejunum showing the distribution of LGR5+ cells and HD5 (Paneth cells, A), epithelial cells (Cytokeratin, B) and adherens junction protein (E-cadherin, C) from a normal uninfected RhM. (A) The white lines show the orientation of crypts in this jejunum section. (D) Distribution of HD5+ cells are shown from a normal SIV-uninfected RhM where the HD5+ Paneth Cells are located at the base of the crypts. Note that the colocalization of LGR5+ cells and epithelial cells (detected by E-cadherin or Cytokeratin) was shown by yellow color (yellow arrows). [file Image_3.pdf]

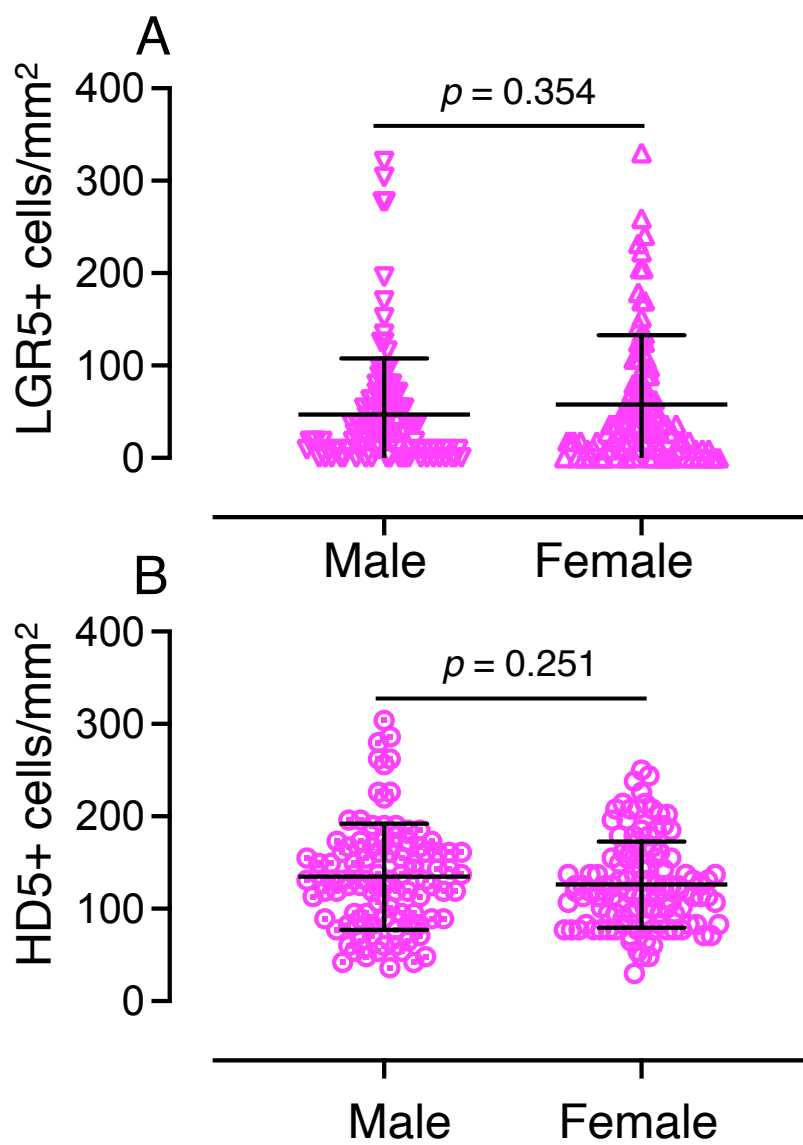

Supplement: Supplementary Figure 4 — Scatter plots of LGR5+ (A) and HD5+ (B) cells are shown for jejunum in male (n=6) and female (n=5) SIV-uninfected control RhMs. The number of LGR5+ and HD5+ cells was quantified from 19-20 randomly selected fields and the number of positive cells in each field was depicted as a single point in the scatter plot. The larger horizontal line denotes the mean frequencies (+/- standard deviation) of each category. No statistically significant differences of LGR5+ or HD5+ cells among male and female macaques were detected with Mann-Whitney T-test. [file Image_4.pdf]

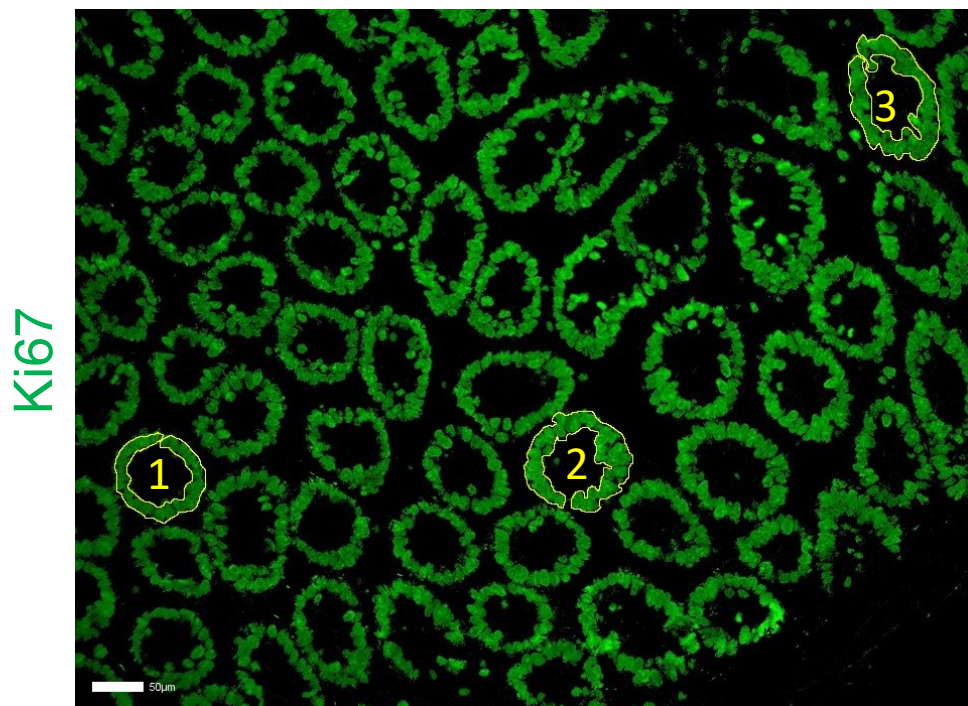

Supplement: Supplementary Figure 5 — The representative figure depicts the gating of region of interest (ROI) for Ki67 staining. The ROIs were manually drawn in the epithelium from randomly selected crypts (highlighted with yellow, and marked as 1, 2, and 3) for the quantification of Ki67 in epithelial cells. [file Image_5.pdf]

## Uninfected control

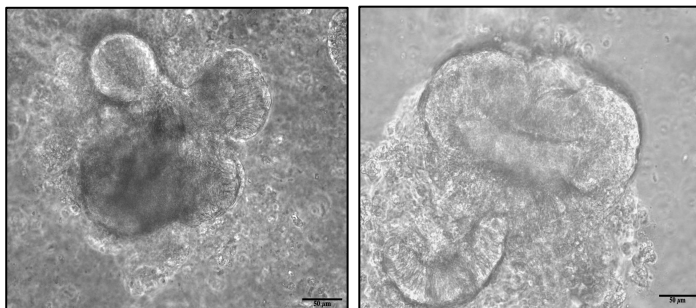

## Chronic SIV

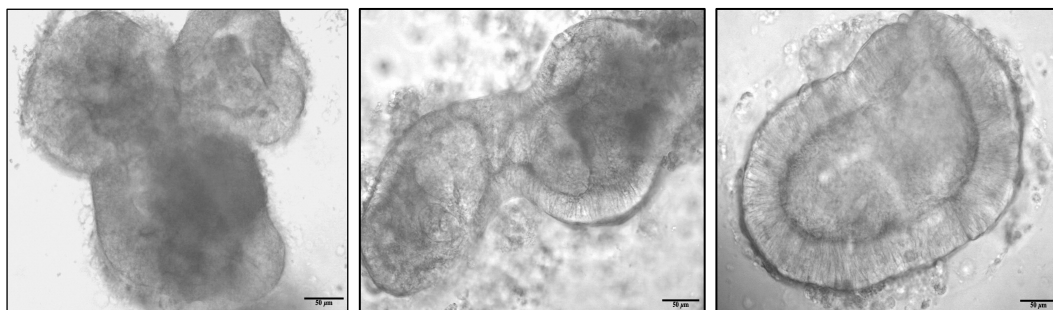

Supplement: Supplementary Figure 6 — Development of fully grown enteroids isolated from single jejunum crypts on day 13th in both uninfected and chronic SIV infected rhesus macaques. [file Image_6.pdf]
