# Supplementary material for: Identification, Characterization, and Transcriptional Reprogramming of Epithelial Stem Cells and Intestinal Enteroids in Simian Immunodeficiency Virus Infected Rhesus Macaques
Source: Front Immunol. 2021 Nov 23;12:769990. doi: 10.3389/fimmu.2021.769990 (PMC8650114; doi:10.3389/fimmu.2021.769990)
Supplement: Supplementary file 7 [file Table_1.pdf]

Supplementary Table 1: List of antibodies used for identifying mucosal mononuclear and epithelial cells in rhesus macaques

Antibody	Isotype	Clone	Assay	Amount (ul)/Dilution	Source
Anti-cow cytokeratin, Wide Spectrum Screening	Rabbit polyclonal IgG	-	IF	1:100	Dako
Anti-human CD24	Mouse IgG1	SN3	FC	20	Abcam
Anti-human CD44	Rat IgG2b	IM7	FC	5	Biolegend
Anti-NHP CD45	Mouse IgG1	D058-1283	FC	5	BD Biosciences
Anti-human CD166	Mouse IgG1	3A6	FC	5	BD Biosciences
Anti-human Mucin	Mouse IgG1	45M1	IF	1:50	SantaCruz Biotech, Inc.
Anti-human Ki67	Mouse IgG1	MIB-1	IF	1:150	Dako
Anti-human E-Cadherin	Mouse IgG1	NCH-38	IF	2□g/mL	Dako
Anti-human alpha Defensin5 (anti-HD5)	Rabbit polyclonal IgG	-	IF	1:2000	In house [1]
Anti-human LGR5	Rabbit polyclonal IgG	-	FC, IF	1.2/1:2000	Novus Biologicals
Mouse IgG1 kappa isotype control	Mouse IgG1		IF	2□g/mL	Invitrogen
Normal Rabbit IgG control	Rabbit IgG		IF	1:100	R&D Systems

Note: Before all staining the dilution of the respective antibodies was determined after serial dilution experiment.

FC and IF denote flow cytometry and immunofluorescence assay, respectively.

[1] Porter EM, Liu L, Oren A, Anton PA, Ganz T. Localization of human intestinal defensin 5 in Paneth cell granules. Infect Immun. 1997;65(6):2389-95. Epub 1997/06/01. doi: 10.1128/iai.65.6.2389-2395.1997. PubMed PMID: 9169779; PubMed Central PMCID: PMCPMC175331.
