# Supplementary material for: Identification, Characterization, and Transcriptional Reprogramming of Epithelial Stem Cells and Intestinal Enteroids in Simian Immunodeficiency Virus Infected Rhesus Macaques
Source: Front Immunol. 2021 Nov 23;12:769990. doi: 10.3389/fimmu.2021.769990 (PMC8650114; doi:10.3389/fimmu.2021.769990)
Supplement: Supplementary file 8 [file Table_2.pdf]

Supplementary Table 2: List of primers used

Target Gene	Function	Primer	Sequence (5' to 3')	Annealing Temp (0C)	Product Length (bp)	Gene ID/Reference
FOXA2	Regulator of intestinal epithelial cell function	Forward<br>Reverse	AATAACAACCCCACCCCAACAC<br>AGACTTCCCTGCAACAACAGC	60	270	XM_001094972.4
GAPDH	Housekeeping gene	Forward<br>Reverse	GAAATCCCATCACCATCTTCCAGG<br>GAGCCCCAGCCTTCTCCATG	57	120	Ahn et al, 2008 [2]
LGR4	Stem cell marker	Forward<br>Reverse	GCCTGAATGGGCTAAATCAA<br>CCTTTCTCCTGTGCCACACT	56	202	NM_001265665.1
LGR5	Intestinal stem cells	Forward<br>Reverse	CAGCGTCTTCACCTCCTACC<br>TTGAAGGCTTCGCAAGTTCT	56	232	XM_028829726.1
MUC2	Present in goblet cells	Forward<br>Reverse	ACACCTACACGCACATCGAG<br>AGTTCACCCACTCCCCAGAT	57	113	XM_028832923.1
OLFM4	Stem cells	Forward<br>Reverse	GGAACGCTTGGAATTCACAGC<br>CCTTCTCCATGATCTCAATTCGG	56	143	XM_001085453.4
SPON1	Extracellular matrix protein	Forward<br>Reverse	TCACCGTCATCAAAGCCAAAG<br>TGCCAGCATCCCAAGGAATC	60	231	NM_001266118.1
VIL1	Calcium-dependent actin binding cytoskeletal protein	Forward<br>Reverse	GGAACGTGGTAGCTGGAGAG<br>ACCTGAAAGTAACCGCATGG	57	120	XM_001090524.4

bp: basepairs

[2] Ahn K, Huh JW, Park SJ, Kim DS, Ha HS, Kim YJ, et al. Selection of internal reference genes for SYBR green qRT-PCR studies of rhesus monkey (Macaca mulatta) tissues. BMC Mol Biol. 2008;9:78. Epub 2008/09/11. doi: 10.1186/1471-2199-9-78. PubMed PMID: 18782457; PubMed Central PMCID: PMCPMC2561044.
