# Supplementary material for: Identification, Characterization, and Transcriptional Reprogramming of Epithelial Stem Cells and Intestinal Enteroids in Simian Immunodeficiency Virus Infected Rhesus Macaques
Source: Front Immunol. 2021 Nov 23;12:769990. doi: 10.3389/fimmu.2021.769990 (PMC8650114; doi:10.3389/fimmu.2021.769990)
Supplement: Supplementary file 9 [file Table_3.pdf]

Supplementary Table 3: The gene list of the 5293 DEGs with official gene symbols

Offical Gene Symbols	ENSEMBL Gene ID	baseMean	log2FoldChange	lfcSE	stat	pvalue	padj	up- or down-regulated	gene type
ASB14	ENSMMUG00000021008	1250.767247	7.393250807	0.265207	27.87733	5.03E-171	3.56E-167	up-regulated	protein_coding
APOM	ENSMMUG00000005303	761.7253291	5.574925765	0.208188	26.77828	5.79E-158	2.73E-154	up-regulated	protein_coding
7SK	ENSMMUG00000026568	8145.199781	9.369114199	0.360678	25.9764	9.15E-149	2.59E-145	up-regulated	misc_RNA
SLC9B1	ENSMMUG00000011206	1004.404116	5.855899261	0.230309	25.42624	1.29E-142	3.05E-139	up-regulated	protein_coding
HPCA	ENSMMUG00000017302	861.0915507	11.6499598	0.529745	21.99164	3.46E-107	4.08E-104	up-regulated	protein_coding
C6orf201	ENSMMUG00000014020	863.5418792	4.350616776	0.202983	21.43338	6.53E-102	5.77E-99	up-regulated	protein_coding
MASTL	ENSMMUG00000016407	396.1957738	4.542379344	0.221966	20.46427	4.48E-93	3.17E-90	up-regulated	protein_coding
C20ORF132	ENSMMUG00000014380	976.7324855	5.913637048	0.292522	20.21603	7.08E-91	4.55E-88	up-regulated	protein_coding
PKD2L2	ENSMMUG00000016357	226.7375403	5.580872915	0.276541	20.18101	1.44E-90	8.84E-88	up-regulated	protein_coding
SNX32	ENSMMUG00000019017	14869.04306	5.151564202	0.256578	20.07797	1.15E-89	6.78E-87	up-regulated	protein_coding
LNX1	ENSMMUG00000017401	557.9468322	4.431750665	0.221182	20.03665	2.64E-89	1.49E-86	up-regulated	protein_coding
RNF39	ENSMMUG00000012124	566.1375825	6.274667459	0.323482	19.39728	8.14E-84	4.11E-81	up-regulated	protein_coding
TSR1	ENSMMUG00000017519	1703.706062	4.60601553	0.241254	19.09199	2.94E-81	1.44E-78	up-regulated	protein_coding
CELSR3	ENSMMUG00000014707	250.2835424	7.835214911	0.411514	19.03996	7.96E-81	3.63E-78	up-regulated	protein_coding
ARG2	ENSMMUG00000015865	596.2847801	4.059494412	0.224218	18.10513	2.90E-73	1.24E-70	up-regulated	protein_coding
MSMP	ENSMMUG00000031859	772.7224943	5.503198348	0.309697	17.76963	1.21E-70	4.65E-68	up-regulated	protein_coding
FAM227B	ENSMMUG00000009840	302.0488356	5.374517679	0.305795	17.57558	3.79E-69	1.37E-66	up-regulated	protein_coding
MAP3K12	ENSMMUG00000006613	311.3162516	5.562761643	0.321245	17.31625	3.55E-67	1.25E-64	up-regulated	protein_coding
DOPEY1	ENSMMUG00000012580	850.1305008	3.698241574	0.215373	17.17132	4.35E-66	1.40E-63	up-regulated	protein_coding
PP2D1	ENSMMUG00000006993	485.3644331	5.532291274	0.326066	16.96677	1.45E-64	4.45E-62	up-regulated	protein_coding
TNNI1	ENSMMUG00000013509	418.264167	5.987042099	0.353311	16.94554	2.08E-64	6.25E-62	up-regulated	protein_coding
ZACN	ENSMMUG00000008883	211.5508505	5.844563387	0.348496	16.77082	3.99E-63	1.15E-60	up-regulated	protein_coding
NR1I3	ENSMMUG00000018128	185.5518009	5.401064395	0.324898	16.62386	4.68E-62	1.27E-59	up-regulated	protein_coding
NME7	ENSMMUG00000021079	2378.6212	4.563636154	0.280729	16.25637	2.01E-59	5.09E-57	up-regulated	protein_coding
TMEM163	ENSMMUG00000010564	121.4473343	6.877869403	0.423734	16.23157	3.02E-59	7.49E-57	up-regulated	protein_coding
PIANP	ENSMMUG00000016994	134.9888559	9.059757538	0.55842	16.22392	3.42E-59	8.34E-57	up-regulated	protein_coding
MYH10	ENSMMUG00000023803	142.4240338	5.984745801	0.36946	16.19861	5.16E-59	1.24E-56	up-regulated	protein_coding
FAM120AOS	ENSMMUG00000002470	1962.006487	5.888689097	0.363845	16.18461	6.48E-59	1.50E-56	up-regulated	protein_coding
LRRTM2	ENSMMUG00000017990	165.983354	9.852805068	0.617543	15.95484	2.64E-57	5.92E-55	up-regulated	protein_coding
C19orf80	ENSMMUG00000009337	201.0979089	4.691465299	0.296715	15.81136	2.60E-56	5.74E-54	up-regulated	protein_coding
GPLD1	ENSMMUG00000022119	345.0869135	5.109426496	0.32318	15.80985	2.66E-56	5.79E-54	up-regulated	protein_coding
APCDD1	ENSMMUG00000011772	131.3606802	6.858189799	0.434466	15.78532	3.93E-56	8.42E-54	up-regulated	protein_coding
CNTD1	ENSMMUG00000012641	617.6545696	4.843502174	0.309324	15.65836	2.91E-55	5.97E-53	up-regulated	protein_coding
PRSS48	ENSMMUG00000009680	105.8178145	8.88208892	0.581141	15.28388	9.79E-53	1.78E-50	up-regulated	protein_coding
MEGF11	ENSMMUG00000008007	180.5923755	6.388502275	0.418404	15.26874	1.24E-52	2.21E-50	up-regulated	protein_coding
RUFY2	ENSMMUG00000019703	1144.519459	5.405772945	0.35758	15.11767	1.24E-51	2.16E-49	up-regulated	protein_coding
DHFR	ENSMMUG00000018283	724.0930597	5.439431646	0.360289	15.09739	1.68E-51	2.91E-49	up-regulated	protein_coding
C9ORF131	ENSMMUG00000020188	168.4471666	7.13041782	0.473907	15.04602	3.67E-51	6.25E-49	up-regulated	protein_coding
NRL	ENSMMUG00000007361	204.9996397	5.828896341	0.388684	14.99647	7.74E-51	1.27E-48	up-regulated	protein_coding
SLC5A10	ENSMMUG00000005325	745.9555051	4.984055141	0.33421	14.91296	2.71E-50	4.36E-48	up-regulated	protein_coding
OBSCN	ENSMMUG00000016891	3345.814927	7.104074862	0.478108	14.85872	6.11E-50	9.60E-48	up-regulated	protein_coding
BACE1	ENSMMUG00000007785	459.6412763	5.392705142	0.363875	14.82022	1.08E-49	1.67E-47	up-regulated	protein_coding
FAN1	ENSMMUG00000009426	315.7593291	4.20492755	0.285673	14.71939	4.84E-49	7.22E-47	up-regulated	protein_coding
C1orf167	ENSMMUG00000020063	234.7156904	5.253912397	0.357429	14.69919	6.52E-49	9.52E-47	up-regulated	protein_coding
B3GALT2	ENSMMUG00000022648	77.05302878	5.80828865	0.398523	14.57455	4.08E-48	5.71E-46	up-regulated	protein_coding
PPT2	ENSMMUG00000018432	791.5881524	4.941387027	0.339674	14.54742	6.06E-48	8.41E-46	up-regulated	protein_coding
MLANA	ENSMMUG00000005476	101.9806171	7.69201149	0.531687	14.46718	1.95E-47	2.68E-45	up-regulated	protein_coding
GPM6B	ENSMMUG00000019415	107.3632924	8.106232326	0.562571	14.40927	4.52E-47	6.16E-45	up-regulated	protein_coding
CPT1B	ENSMMUG00000003121	175.6284229	5.205696251	0.363164	14.3343	1.34E-46	1.75E-44	up-regulated	protein_coding
LRRC4	ENSMMUG00000015436	112.6998499	6.981652346	0.488619	14.28853	2.58E-46	3.35E-44	up-regulated	protein_coding
GRIN3A	ENSMMUG00000016675	104.1455899	7.106228439	0.499939	14.21419	7.48E-46	9.62E-44	up-regulated	protein_coding
CEPT1	ENSMMUG00000002461	533.5220146	3.309153757	0.232865	14.21061	7.87E-46	1.00E-43	up-regulated	protein_coding
COL10A1	ENSMMUG00000014642	104.9751409	7.975702811	0.564028	14.14061	2.13E-45	2.67E-43	up-regulated	protein_coding
ELK4	ENSMMUG00000001024	311.0278426	5.476542294	0.390658	14.01876	1.20E-44	1.46E-42	up-regulated	protein_coding
ABI3BP	ENSMMUG00000002865	64.50860266	6.191759148	0.442153	14.00366	1.48E-44	1.79E-42	up-regulated	protein_coding
CBR3	ENSMMUG00000010338	96.27367416	6.232548922	0.449143	13.87654	8.79E-44	1.03E-41	up-regulated	protein_coding
TCP11L1	ENSMMUG00000002539	510.8255371	4.93933393	0.356232	13.8655	1.03E-43	1.18E-41	up-regulated	protein_coding
FAM71E1	ENSMMUG00000006717	329.8499704	3.644107689	0.263706	13.81884	1.96E-43	2.24E-41	up-regulated	protein_coding
TRIM17	ENSMMUG00000005307	101.1460925	7.781300514	0.563408	13.81113	2.18E-43	2.45E-41	up-regulated	protein_coding
RASL12	ENSMMUG00000004029	133.5075947	7.344452586	0.533344	13.77058	3.83E-43	4.24E-41	up-regulated	protein_coding
TMEM53	ENSMMUG00000014820	1084.196312	4.556486029	0.334754	13.61145	3.42E-42	3.64E-40	up-regulated	protein_coding
VAPA	ENSMMUG00000014418	4738.529718	5.061775802	0.374074	13.53147	1.02E-41	1.08E-39	up-regulated	protein_coding
ALX4	ENSMMUG00000013097	104.2109862	8.293567368	0.615087	13.48356	1.95E-41	2.05E-39	up-regulated	protein_coding
MXD3	ENSMMUG00000009047	7260.655101	4.897102163	0.365197	13.40948	5.32E-41	5.54E-39	up-regulated	protein_coding
CYP2F1	ENSMMUG00000028758	160.9031635	5.558212694	0.415032	13.39224	6.71E-41	6.93E-39	up-regulated	protein_coding
GNAT2	ENSMMUG00000005819	148.6837754	5.235403011	0.393469	13.30577	2.14E-40	2.18E-38	up-regulated	protein_coding
COG1	ENSMMUG00000001266	806.9985644	3.658566699	0.275235	13.29254	2.56E-40	2.58E-38	up-regulated	protein_coding
HIST1H1E	ENSMMUG00000023748	60.56208136	6.677750943	0.502585	13.2868	2.76E-40	2.77E-38	up-regulated	protein_coding
BEST1	ENSMMUG00000015147	64.27593107	6.2066346	0.467598	13.27343	3.30E-40	3.29E-38	up-regulated	protein_coding
RFC5	ENSMMUG00000010235	392.6970445	4.310721205	0.324935	13.26643	3.62E-40	3.59E-38	up-regulated	protein_coding
TMBIM6	ENSMMUG00000005960	1801.260325	-3.969887556	0.299684	-13.2469	4.70E-40	4.62E-38	down-regulated	protein_coding
RBM12B-AS1	ENSMMUG00000005559	79.8937076	6.799894669	0.515366	13.19429	9.46E-40	9.24E-38	up-regulated	protein_coding
METTL5	ENSMMUG00000023054	1465.633495	4.78446982	0.366957	13.03822	7.42E-39	7.09E-37	up-regulated	protein_coding
YWHAZ	ENSMMUG00000006489	1595.848191	-3.414007537	0.262027	-13.0292	8.35E-39	7.93E-37	down-regulated	protein_coding
IFNK	ENSMMUG00000016510	94.83191872	8.581922629	0.661432	12.97477	1.70E-38	1.58E-36	up-regulated	protein_coding
MLNR	ENSMMUG00000007691	82.70994333	8.387193823	0.64684	12.96642	1.90E-38	1.75E-36	up-regulated	protein_coding
RUVBL1	ENSMMUG00000018662	1227.093718	4.071514596	0.314212	12.95785	2.12E-38	1.95E-36	up-regulated	protein_coding
TPPP3	ENSMMUG00000009822	84.38377689	5.019567292	0.388302	12.92698	3.17E-38	2.87E-36	up-regulated	protein_coding
TRIM67	ENSMMUG00000017449	61.66039908	6.196680202	0.480375	12.89968	4.52E-38	4.05E-36	up-regulated	protein_coding
ANGPTL2	ENSMMUG00000012161	164.2192242	8.753702741	0.67959	12.88086	5.77E-38	5.13E-36	up-regulated	protein_coding
PFKM	ENSMMUG00000002045	275.3607727	4.157574545	0.32347	12.85303	8.27E-38	7.27E-36	up-regulated	protein_coding
SCUBE3	ENSMMUG00000023557	208.3041118	6.98939346	0.54551	12.81259	1.39E-37	1.22E-35	up-regulated	protein_coding
AGBL5	ENSMMUG00000016044	186.0135769	3.898827495	0.305733	12.75241	3.02E-37	2.62E-35	up-regulated	protein_coding
XRRA1	ENSMMUG00000021039	303.771399	5.448903944	0.427876	12.73477	3.79E-37	3.27E-35	up-regulated	protein_coding
CACNA1A	ENSMMUG00000003555	133.0654546	6.324906481	0.496831	12.73051	4.00E-37	3.43E-35	up-regulated	protein_coding
ACSBG1	ENSMMUG00000011839	121.8833818	5.486925124	0.431441	12.71767	4.72E-37	4.00E-35	up-regulated	protein_coding
COX11	ENSMMUG00000010548	1404.071791	4.353878094	0.342778	12.70176	5.78E-37	4.87E-35	up-regulated	protein_coding
5S_rRNA	ENSMMUG00000024636	57.06225231	6.87212163	0.542379	12.67033	8.63E-37	7.23E-35	up-regulated	rRNA
AVIL	ENSMMUG00000005319	161.2310404	5.782175982	0.45655	12.66493	9.25E-37	7.70E-35	up-regulated	protein_coding
Y_RNA	ENSMMUG00000027685	67.40216817	6.431181336	0.508255	12.65346	1.07E-36	8.76E-35	up-regulated	misc_RNA
SLC4A9	ENSMMUG00000003723	136.3020241	7.654510654	0.606196	12.62712	1.50E-36	1.22E-34	up-regulated	protein_coding
C9orf84	ENSMMUG00000023307	101.4852128	6.118982883	0.484823	12.62107	1.62E-36	1.31E-34	up-regulated	protein_coding
MMP24	ENSMMUG00000018206	93.77240832	5.423476355	0.429871	12.61652	1.71E-36	1.38E-34	up-regulated	protein_coding
EMILIN1	ENSMMUG00000016046	107.2144602	5.847602275	0.463698	12.61079	1.84E-36	1.46E-34	up-regulated	protein_coding
DUSP11	ENSMMUG00000021537	393.8232853	3.841605803	0.305767	12.56383	3.34E-36	2.64E-34	up-regulated	protein_coding
OIT3	ENSMMUG00000020404	257.3052871	5.647300117	0.449975	12.55026	3.96E-36	3.11E-34	up-regulated	protein_coding
PMEL	ENSMMUG00000018816	136.8143468	3.358563053	0.268494	12.50889	6.68E-36	5.19E-34	up-regulated	protein_coding
RELL1	ENSMMUG00000017630	821.9374025	3.985748273	0.320073	12.45261	1.35E-35	1.04E-33	up-regulated	protein_coding

TTN	ENSMMUG00000009038	119.6261127	7.857061921	0.631494	12.44202	1.55E-35	1.18E-33	up-regulated	protein_coding
AMIGO3	ENSMMUG00000014582	141.1457558	5.130456566	0.413753	12.3998	2.62E-35	1.99E-33	up-regulated	protein_coding
EMILIN3	ENSMMUG00000004252	125.4834837	4.077848151	0.329301	12.38334	3.22E-35	2.43E-33	up-regulated	protein_coding
SCARNA2	ENSMMUG00000035645	137.9613626	5.732359716	0.464799	12.33298	6.02E-35	4.48E-33	up-regulated	snoRNA
HTR2B	ENSMMUG00000011773	69.49209328	8.281236296	0.673343	12.29869	9.21E-35	6.82E-33	up-regulated	protein_coding
MED31	ENSMMUG00000003790	281.3796666	4.211313586	0.342889	12.28184	1.13E-34	8.36E-33	up-regulated	protein_coding
C1ORF84	ENSMMUG00000023674	1557.947635	3.658311558	0.298118	12.27135	1.29E-34	9.46E-33	up-regulated	protein_coding
CIDEB	ENSMMUG00000010048	604.5732815	3.145095609	0.257506	12.21369	2.63E-34	1.90E-32	up-regulated	protein_coding
ZNFX1	ENSMMUG00000023623	1257.264432	4.901317138	0.401382	12.21111	2.71E-34	1.95E-32	up-regulated	protein_coding
ZNF3	ENSMMUG00000004021	267.3217767	3.72262196	0.305324	12.19235	3.41E-34	2.44E-32	up-regulated	protein_coding
ABHD1	ENSMMUG00000002388	272.92653	6.052296346	0.496919	12.17965	3.99E-34	2.81E-32	up-regulated	protein_coding
GNRHR2	ENSMMUG00000016049	142.84912	4.497427412	0.37058	12.13617	6.79E-34	4.76E-32	up-regulated	protein_coding
SDR39U1	ENSMMUG00000009517	1303.496998	4.338841498	0.358299	12.10957	9.40E-34	6.52E-32	up-regulated	protein_coding
C7H14ORF119	ENSMMUG00000012978	361.1135528	3.32094954	0.274573	12.09498	1.12E-33	7.75E-32	up-regulated	protein_coding
EFCAB10	ENSMMUG00000004301	163.991609	4.314655172	0.35957	11.99949	3.58E-33	2.42E-31	up-regulated	protein_coding
FAM196A	ENSMMUG00000018550	271.0469238	8.997225694	0.754714	11.92138	9.16E-33	6.17E-31	up-regulated	protein_coding
SPACA4	ENSMMUG00000022157	134.3461318	4.820139941	0.404375	11.91997	9.31E-33	6.25E-31	up-regulated	protein_coding
HEATR1	ENSMMUG00000015135	375.5387584	3.301373189	0.277226	11.90862	1.07E-32	7.09E-31	up-regulated	protein_coding
TMSB4X	ENSMMUG00000014256	1732.882315	-7.013838481	0.589629	-11.8954	1.25E-32	8.27E-31	down-regulated	protein_coding
C1ORF192	ENSMMUG00000008251	321.677221	4.83054289	0.406504	11.88314	1.45E-32	9.49E-31	up-regulated	protein_coding
RYR3	ENSMMUG00000005270	50.63597429	7.501347674	0.63257	11.85852	1.94E-32	1.26E-30	up-regulated	protein_coding
FAM151A	ENSMMUG00000013680	780.9209666	4.812574771	0.405988	11.85398	2.05E-32	1.33E-30	up-regulated	protein_coding
HAS3	ENSMMUG00000010922	674.2994246	4.560690469	0.386546	11.79856	3.97E-32	2.54E-30	up-regulated	protein_coding
RAD9A	ENSMMUG00000017275	1284.764151	4.702388613	0.398754	11.7927	4.26E-32	2.70E-30	up-regulated	protein_coding
OAZ3	ENSMMUG00000009095	684.2392111	5.287194841	0.448362	11.79225	4.28E-32	2.70E-30	up-regulated	protein_coding
MASP2	ENSMMUG00000007459	160.1967677	5.813711075	0.49502	11.7444	7.55E-32	4.70E-30	up-regulated	protein_coding
TMED8	ENSMMUG00000001098	137.1332874	3.848486406	0.328241	11.72459	9.54E-32	5.92E-30	up-regulated	protein_coding
ND3	ENSMMUG00000028680	1062.584755	12.79683005	1.094526	11.69166	1.41E-31	8.61E-30	up-regulated	protein_coding
DENND1C	ENSMMUG00000005600	272.410531	4.174384747	0.358892	11.6313	2.86E-31	1.73E-29	up-regulated	protein_coding
CYP2D42	ENSMMUG00000017962	1236.864998	4.400831955	0.378826	11.61702	3.38E-31	2.03E-29	up-regulated	protein_coding
FUT2	ENSMMUG00000019738	102.8810334	6.548407471	0.564091	11.60877	3.72E-31	2.22E-29	up-regulated	protein_coding
THAP11	ENSMMUG00000013240	367.8710151	3.740413057	0.322374	11.60273	3.99E-31	2.37E-29	up-regulated	protein_coding
CHAD	ENSMMUG00000017238	139.2263975	5.116535542	0.442982	11.55021	7.36E-31	4.36E-29	up-regulated	protein_coding
C1H1ORF111	ENSMMUG00000004790	97.83890075	7.608220775	0.658991	11.54526	7.80E-31	4.60E-29	up-regulated	protein_coding
CASKIN1	ENSMMUG00000019226	731.3608673	4.519503758	0.391879	11.53289	9.01E-31	5.29E-29	up-regulated	protein_coding
SOX30	ENSMMUG00000021550	169.9402534	4.817350833	0.417748	11.53172	9.13E-31	5.34E-29	up-regulated	protein_coding
LIMS2	ENSMMUG00000004179	615.6431068	4.572575352	0.397044	11.51654	1.09E-30	6.31E-29	up-regulated	protein_coding
GPR82	ENSMMUG00000022584	50.92796319	6.883841476	0.598419	11.50337	1.27E-30	7.30E-29	up-regulated	protein_coding
SPON1	ENSMMUG00000021914	77.09971939	6.310745191	0.550709	11.45931	2.11E-30	1.20E-28	up-regulated	protein_coding
DPAGT1	ENSMMUG00000014872	674.2810874	3.849224995	0.336343	11.44434	2.51E-30	1.43E-28	up-regulated	protein_coding
VASN	ENSMMUG00000006500	70.67486876	6.785905124	0.593595	11.43188	2.90E-30	1.64E-28	up-regulated	protein_coding
MRPL43	ENSMMUG00000010679	1651.603028	3.709416364	0.325025	11.41271	3.61E-30	2.04E-28	up-regulated	protein_coding
7SK	ENSMMUG00000036617	55.76945851	6.979435814	0.615066	11.34746	7.64E-30	4.24E-28	up-regulated	misc_RNA
CHMP5	ENSMMUG00000020149	1606.790081	3.677345559	0.324775	11.32275	1.01E-29	5.60E-28	up-regulated	protein_coding
CREB3	ENSMMUG00000016328	851.2897861	4.038915485	0.356862	11.31787	1.07E-29	5.89E-28	up-regulated	protein_coding
MED11	ENSMMUG00000004892	388.8335131	3.525705283	0.31181	11.30721	1.21E-29	6.63E-28	up-regulated	protein_coding
OMG	ENSMMUG00000006824	93.28682612	9.245017098	0.817656	11.30673	1.22E-29	6.64E-28	up-regulated	protein_coding
ISM1	ENSMMUG00000015939	76.15620087	8.944980011	0.792007	11.29406	1.40E-29	7.64E-28	up-regulated	protein_coding
C1ORF124	ENSMMUG00000005244	164.1734594	3.593359516	0.318214	11.29228	1.43E-29	7.77E-28	up-regulated	protein_coding
EVL	ENSMMUG00000015434	466.0928285	4.171350228	0.371842	11.21807	3.32E-29	1.79E-27	up-regulated	protein_coding
CALM1	ENSMMUG00000018185	759.4207603	-3.809327994	0.340098	-11.2007	4.05E-29	2.17E-27	down-regulated	protein_coding
ZNF750	ENSMMUG00000005374	54.6542648	7.139055823	0.640133	11.15246	6.97E-29	3.69E-27	up-regulated	protein_coding
TCP1	ENSMMUG00000012846	2115.253478	3.579011891	0.321068	11.14721	7.39E-29	3.90E-27	up-regulated	protein_coding
HSPCA	ENSMMUG00000011073	1119.530887	-4.144475938	0.37184	-11.1458	7.50E-29	3.95E-27	down-regulated	protein_coding
GRIN2C	ENSMMUG00000005744	71.94040344	4.735658654	0.425287	11.13521	8.45E-29	4.41E-27	up-regulated	protein_coding
EML5	ENSMMUG00000004606	72.57415943	4.962627649	0.445893	11.12965	9.00E-29	4.68E-27	up-regulated	protein_coding
LDHAL6B	ENSMMUG00000012934	91.31402058	9.337968475	0.842484	11.08385	1.50E-28	7.73E-27	up-regulated	protein_coding
ARF1	ENSMMUG00000002792	680.9858903	-4.128277759	0.372791	-11.074	1.68E-28	8.60E-27	down-regulated	protein_coding
CNTNAP1	ENSMMUG00000006091	61.49069339	6.780709476	0.6136	11.0507	2.18E-28	1.10E-26	up-regulated	protein_coding
SLC4A8	ENSMMUG00000006607	58.85178908	6.322746563	0.573781	11.01944	3.08E-28	1.55E-26	up-regulated	protein_coding
KBTBD7	ENSMMUG00000004799	97.51308333	4.621343176	0.423132	10.92175	9.07E-28	4.56E-26	up-regulated	protein_coding
HOMEZ	ENSMMUG00000014744	198.3666874	2.858502534	0.261961	10.91194	1.01E-27	5.00E-26	up-regulated	protein_coding
RNaseP_nuc	ENSMMUG00000036452	1288.900097	10.35034593	0.951173	10.88167	1.41E-27	6.93E-26	up-regulated	misc_RNA
ZNF189	ENSMMUG00000023795	540.9121263	5.592602758	0.514311	10.87398	1.53E-27	7.51E-26	up-regulated	protein_coding
RECQL	ENSMMUG00000010857	333.8615423	3.41540164	0.314328	10.86574	1.68E-27	8.19E-26	up-regulated	protein_coding
KRT12	ENSMMUG00000001502	53.24615673	5.64914572	0.520662	10.84993	2.00E-27	9.70E-26	up-regulated	protein_coding
TIAM2	ENSMMUG00000011169	616.8756667	4.260562108	0.393001	10.84111	2.20E-27	1.07E-25	up-regulated	protein_coding
KANSL1L	ENSMMUG00000010515	195.6239404	3.964773324	0.365875	10.83642	2.31E-27	1.12E-25	up-regulated	protein_coding
NKIRAS2	ENSMMUG00000014275	356.3043846	3.197526632	0.295371	10.82546	2.61E-27	1.25E-25	up-regulated	protein_coding
IL17RD	ENSMMUG00000016602	116.5786969	5.068636331	0.468431	10.82044	2.75E-27	1.32E-25	up-regulated	protein_coding
GSTA3	ENSMMUG00000017137	3299.670098	-4.807893581	0.446709	-10.7629	5.15E-27	2.46E-25	down-regulated	protein_coding
RELL2	ENSMMUG00000012591	546.6921916	3.853735142	0.358793	10.74084	6.54E-27	3.12E-25	up-regulated	protein_coding
PHC1	ENSMMUG00000017843	808.1783589	4.119712051	0.384475	10.71515	8.64E-27	4.08E-25	up-regulated	protein_coding
GPR124	ENSMMUG00000003204	42.77205543	6.112161041	0.570641	10.71104	9.03E-27	4.25E-25	up-regulated	protein_coding
TTC19	ENSMMUG00000008949	239.7201551	2.344531083	0.2189	10.71049	9.09E-27	4.26E-25	up-regulated	protein_coding
SLC26A1	ENSMMUG00000017223	440.1582186	5.272848406	0.493641	10.68154	1.24E-26	5.76E-25	up-regulated	protein_coding
ALDH1A1	ENSMMUG00000013303	2996.496502	-4.04280857	0.378656	-10.6767	1.31E-26	6.05E-25	down-regulated	protein_coding
IDH1	ENSMMUG00000016077	676.422312	-5.004614552	0.469493	-10.6596	1.57E-26	7.25E-25	down-regulated	protein_coding
HIST2H3D	ENSMMUG00000031746	177.4921695	7.389524003	0.693626	10.65347	1.68E-26	7.72E-25	up-regulated	protein_coding
GPR18	ENSMMUG00000012336	61.8181135	7.805474262	0.735513	10.61228	2.61E-26	1.19E-24	up-regulated	protein_coding
JAZF1	ENSMMUG00000022456	42.08236548	5.243078615	0.495548	10.58037	3.67E-26	1.66E-24	up-regulated	protein_coding
CTNNB1	ENSMMUG00000014627	671.5295252	-3.414815508	0.32305	-10.5706	4.08E-26	1.84E-24	down-regulated	protein_coding
ITGA7	ENSMMUG00000013226	62.44728526	4.692132007	0.444071	10.56618	4.28E-26	1.92E-24	up-regulated	protein_coding
SC4MOL	ENSMMUG00000012994	726.6269332	-5.567075867	0.527633	-10.551	5.02E-26	2.24E-24	down-regulated	protein_coding
COCH	ENSMMUG00000011514	59.97532518	4.284796243	0.407522	10.51428	7.42E-26	3.30E-24	up-regulated	protein_coding
NID2	ENSMMUG00000018201	54.66275646	6.98256278	0.665999	10.48434	1.02E-25	4.51E-24	up-regulated	protein_coding
PRDM2	ENSMMUG00000017040	362.3174883	3.220939514	0.307667	10.46892	1.20E-25	5.27E-24	up-regulated	protein_coding
SNORD91	ENSMMUG00000032912	56.56950918	6.8824628	0.658858	10.44604	1.53E-25	6.69E-24	up-regulated	snoRNA
OAZ1	ENSMMUG00000017949	875.292211	-4.494354558	0.430556	-10.4385	1.65E-25	7.22E-24	down-regulated	protein_coding
Y_RNA	ENSMMUG00000036138	37.09638325	5.392882839	0.517156	10.42797	1.85E-25	8.05E-24	up-regulated	misc_RNA
RAD1	ENSMMUG00000030184	251.1881284	4.155279551	0.399625	10.39794	2.53E-25	1.09E-23	up-regulated	protein_coding
CSDE1	ENSMMUG00000001974	534.0028659	-3.322890149	0.319649	-10.3954	2.60E-25	1.12E-23	down-regulated	protein_coding
ACTA2	ENSMMUG00000009341	60.87254444	5.437722781	0.523128	10.39463	2.62E-25	1.12E-23	up-regulated	protein_coding
CENPN	ENSMMUG00000013082	292.1790552	3.825489367	0.369849	10.34339	4.48E-25	1.91E-23	up-regulated	protein_coding
SLC26A5	ENSMMUG00000003433	46.74540569	7.683512057	0.746691	10.29008	7.81E-25	3.26E-23	up-regulated	protein_coding
SNORA70	ENSMMUG00000024433	43.73383309	8.947390451	0.870013	10.2842	8.30E-25	3.46E-23	up-regulated	snoRNA
COMMD5	ENSMMUG00000011549	239.9144947	3.001876276	0.292461	10.26419	1.02E-24	4.24E-23	up-regulated	protein_coding
JMJD8	ENSMMUG00000001984	3383.264676	4.348015333	0.424253	10.24864	1.20E-24	4.94E-23	up-regulated	protein_coding
LOXL3	ENSMMUG00000002138	193.312921	4.906281544	0.47903	10.24212	1.28E-24	5.27E-23	up-regulated	protein_coding
THAP3	ENSMMUG00000002047	1067.891609	4.224824584	0.41286	10.23306	1.41E-24	5.75E-23	up-regulated	protein_coding

SKIV2L2	ENSMMUG00000010379	636.7859057	3.135689952	0.308491	10.16462	2.85E-24	1.15E-22	up-regulated	protein_coding
RAD17	ENSMMUG00000014881	154.1860025	2.758996041	0.271601	10.15827	3.04E-24	1.22E-22	up-regulated	protein_coding
MMACHC	ENSMMUG00000015950	362.6178028	4.445671391	0.446497	9.956788	2.36E-23	9.23E-22	up-regulated	protein_coding
SPATA21	ENSMMUG00000015898	34.84775229	6.003805213	0.603206	9.95316	2.44E-23	9.55E-22	up-regulated	protein_coding
BCL2L12	ENSMMUG00000003926	656.5606956	3.614066855	0.363522	9.941803	2.74E-23	1.07E-21	up-regulated	protein_coding
AGER	ENSMMUG00000018442	53.0814959	5.071550824	0.510588	9.932764	3.00E-23	1.17E-21	up-regulated	protein_coding
Y_RNA	ENSMMUG00000034769	41.60679409	6.426805201	0.647501	9.925553	3.22E-23	1.25E-21	up-regulated	misc_RNA
RFTN1	ENSMMUG00000022694	67.55323124	6.958421242	0.70152	9.919064	3.44E-23	1.33E-21	up-regulated	protein_coding
MTMR7	ENSMMUG00000001878	77.93550285	6.351664831	0.641611	9.899556	4.18E-23	1.61E-21	up-regulated	protein_coding
CATSPERD	ENSMMUG00000028918	64.60464541	8.923385973	0.902261	9.890033	4.60E-23	1.77E-21	up-regulated	protein_coding
ATF5	ENSMMUG00000011630	261.3103799	4.007056892	0.405701	9.876877	5.24E-23	2.01E-21	up-regulated	protein_coding
CPSF3L	ENSMMUG00000013866	602.7395095	2.868913021	0.291042	9.857377	6.37E-23	2.42E-21	up-regulated	protein_coding
GPR22	ENSMMUG00000015412	31.50959209	7.60219338	0.771498	9.853814	6.60E-23	2.50E-21	up-regulated	protein_coding
TUBB1	ENSMMUG00000013187	31.8000468	6.845748061	0.695411	9.844169	7.26E-23	2.74E-21	up-regulated	protein_coding
HIST1H2AH	ENSMMUG00000029907	36.8823852	6.887254417	0.700451	9.8326	8.15E-23	3.07E-21	up-regulated	protein_coding
GLS2	ENSMMUG00000000860	137.8291982	4.406149521	0.448876	9.815951	9.61E-23	3.60E-21	up-regulated	protein_coding
TNRC18	ENSMMUG00000008220	421.4791168	2.629689857	0.268054	9.810306	1.02E-22	3.80E-21	up-regulated	protein_coding
SLC25A5	ENSMMUG00000022663	935.7827738	-5.543126694	0.565412	-9.8037	1.09E-22	4.04E-21	down-regulated	protein_coding
EXTL1	ENSMMUG00000012329	59.05747554	6.856659136	0.69976	9.798585	1.14E-22	4.24E-21	up-regulated	protein_coding
NPAS3	ENSMMUG00000014839	49.76849507	9.096053101	0.929827	9.782523	1.34E-22	4.94E-21	up-regulated	protein_coding
FLRT1	ENSMMUG00000000482	29.17658533	6.568245152	0.673031	9.759206	1.68E-22	6.19E-21	up-regulated	protein_coding
LOXL4	ENSMMUG00000010285	78.78376697	10.18343808	1.0448	9.746779	1.90E-22	6.96E-21	up-regulated	protein_coding
ALOX12	ENSMMUG00000016264	306.1676115	4.258018807	0.437067	9.742253	1.99E-22	7.26E-21	up-regulated	protein_coding
SLC5A11	ENSMMUG00000001407	33.6599646	7.169174479	0.735953	9.741349	2.01E-22	7.31E-21	up-regulated	protein_coding
CENPP	ENSMMUG00000004630	94.23353412	4.101418607	0.422317	9.711711	2.69E-22	9.73E-21	up-regulated	protein_coding
ELP5	ENSMMUG00000010549	393.1265816	3.583011256	0.368936	9.711739	2.69E-22	9.73E-21	up-regulated	protein_coding
MAB21L2	ENSMMUG00000002220	83.87760094	9.089307771	0.937413	9.696163	3.13E-22	1.13E-20	up-regulated	protein_coding
PRICKLE1	ENSMMUG00000021733	44.14377943	7.627492689	0.787574	9.684797	3.50E-22	1.25E-20	up-regulated	protein_coding
SGSH	ENSMMUG00000002849	297.2206915	3.307149009	0.341732	9.677623	3.75E-22	1.34E-20	up-regulated	protein_coding
MYL12B	ENSMMUG00000013752	1005.566229	-3.037128013	0.313901	-9.67543	3.83E-22	1.36E-20	down-regulated	protein_coding
MMP14	ENSMMUG00000013665	415.2237753	-3.150069614	0.325682	-9.67223	3.96E-22	1.40E-20	down-regulated	protein_coding
PSORS1C2	ENSMMUG00000015677	30.47329915	6.452230408	0.669231	9.641256	5.35E-22	1.89E-20	up-regulated	protein_coding
5_8S_rRNA	ENSMMUG00000025378	8302.766355	14.17403656	1.471117	9.634881	5.70E-22	1.99E-20	up-regulated	rRNA
snoU89	ENSMMUG00000035606	41.58210582	6.746759749	0.701424	9.618654	6.67E-22	2.32E-20	up-regulated	snoRNA
HIGD1B	ENSMMUG00000013110	52.48495506	6.229914833	0.648098	9.612611	7.07E-22	2.46E-20	up-regulated	protein_coding
NFATC3	ENSMMUG00000021843	156.7721922	3.313735257	0.344769	9.611463	7.15E-22	2.48E-20	up-regulated	protein_coding
SNORA70	ENSMMUG00000035846	39.38333902	7.879238711	0.82187	9.586965	9.07E-22	3.14E-20	up-regulated	snoRNA
SLC5A2	ENSMMUG00000000897	373.2417274	4.42672214	0.461923	9.583255	9.40E-22	3.25E-20	up-regulated	protein_coding
COXII	ENSMMUG00000028686	3622.899603	10.62245752	1.111952	9.552983	1.26E-21	4.31E-20	up-regulated	protein_coding
CACNA2D2	ENSMMUG00000007022	35.57223351	5.028408304	0.52673	9.54646	1.34E-21	4.58E-20	up-regulated	protein_coding
ZNF710	ENSMMUG00000014831	303.5346892	3.034624805	0.31851	9.527568	1.61E-21	5.48E-20	up-regulated	protein_coding
EVI2B	ENSMMUG00000008603	65.50296375	6.178554861	0.649178	9.517512	1.77E-21	6.02E-20	up-regulated	protein_coding
CTNNA1	ENSMMUG00000017985	712.90928	-2.84501596	0.299785	-9.49017	2.31E-21	7.81E-20	down-regulated	protein_coding
H2AFY2	ENSMMUG00000006395	114.1198603	5.323785129	0.561519	9.481041	2.52E-21	8.48E-20	up-regulated	protein_coding
DONSON	ENSMMUG00000003701	426.3736853	3.466756245	0.365683	9.480231	2.54E-21	8.52E-20	up-regulated	protein_coding
ACSBG2	ENSMMUG00000018937	39.96661853	7.390375665	0.779574	9.480023	2.54E-21	8.52E-20	up-regulated	protein_coding
C18orf8	ENSMMUG00000021480	231.516587	2.271363354	0.239883	9.46863	2.84E-21	9.44E-20	up-regulated	protein_coding
NUDT13	ENSMMUG00000011276	48.22683466	3.728362754	0.39486	9.442251	3.65E-21	1.21E-19	up-regulated	protein_coding
ENDOU	ENSMMUG00000018399	43.99079631	6.622060072	0.701429	9.440818	3.70E-21	1.23E-19	up-regulated	protein_coding
CRYM	ENSMMUG00000016745	59.76294998	4.131665745	0.437817	9.436965	3.84E-21	1.27E-19	up-regulated	protein_coding
LEAP2	ENSMMUG00000015638	49.43887326	4.563592097	0.483711	9.434545	3.93E-21	1.30E-19	up-regulated	protein_coding
SPTB	ENSMMUG00000019172	113.2590618	4.870731274	0.516561	9.429151	4.13E-21	1.36E-19	up-regulated	protein_coding
U3	ENSMMUG00000027695	175.8907749	4.481189459	0.476394	9.406481	5.13E-21	1.68E-19	up-regulated	snoRNA
UBC	ENSMMUG00000002320	1711.02231	-3.056693125	0.325406	-9.39347	5.81E-21	1.89E-19	down-regulated	protein_coding
SYTL3	ENSMMUG00000019455	87.9121505	4.62118017	0.492275	9.387389	6.15E-21	2.00E-19	up-regulated	protein_coding
F11R	ENSMMUG00000001996	701.1824333	-2.701017161	0.287932	-9.38073	6.55E-21	2.12E-19	down-regulated	protein_coding
ND2	ENSMMUG00000028695	21389.12691	7.870738623	0.84233	9.34401	9.28E-21	2.96E-19	up-regulated	protein_coding
ST7L	ENSMMUG00000007766	152.1151455	2.828040987	0.303281	9.324834	1.11E-20	3.52E-19	up-regulated	protein_coding
CRB2	ENSMMUG00000006345	183.0935639	4.018374746	0.431621	9.309966	1.28E-20	4.04E-19	up-regulated	protein_coding
SERPIND1	ENSMMUG00000022169	66.97372102	8.402946319	0.903524	9.300188	1.40E-20	4.42E-19	up-regulated	protein_coding
DNAH2	ENSMMUG00000008166	26.7798723	6.322896293	0.680785	9.287657	1.58E-20	4.93E-19	up-regulated	protein_coding
ARHGEF39	ENSMMUG00000016323	1529.398509	5.027407076	0.54164	9.28183	1.67E-20	5.19E-19	up-regulated	protein_coding
FAM184B	ENSMMUG00000018067	164.9516507	4.928760807	0.532134	9.262254	2.00E-20	6.18E-19	up-regulated	protein_coding
SERINC4	ENSMMUG00000022770	112.2227704	5.064049261	0.546782	9.261543	2.01E-20	6.21E-19	up-regulated	protein_coding
ND5	ENSMMUG00000028673	28366.72754	6.585649921	0.711143	9.260657	2.03E-20	6.25E-19	up-regulated	protein_coding
U2	ENSMMUG00000034414	39.66887627	5.958508315	0.645835	9.226059	2.81E-20	8.62E-19	up-regulated	snRNA
MFSD11	ENSMMUG00000022414	2060.728324	4.255058592	0.464015	9.170085	4.73E-20	1.44E-18	up-regulated	protein_coding
Y_RNA	ENSMMUG00000026452	55.08421427	11.6572026	1.276545	9.131841	6.73E-20	2.04E-18	up-regulated	misc_RNA
HCN3	ENSMMUG00000023142	171.1761849	3.338699787	0.365843	9.126043	7.10E-20	2.14E-18	up-regulated	protein_coding
R3HDML	ENSMMUG00000022215	51.71524082	7.920504779	0.867993	9.125074	7.17E-20	2.15E-18	up-regulated	protein_coding
KCNAB1	ENSMMUG00000016968	38.07144399	7.078121323	0.776146	9.119579	7.54E-20	2.26E-18	up-regulated	protein_coding
TP53BP1	ENSMMUG00000017078	201.2253664	2.664390868	0.29301	9.093165	9.62E-20	2.87E-18	up-regulated	protein_coding
IFT172	ENSMMUG00000005096	234.3436489	3.770226711	0.414657	9.092408	9.69E-20	2.89E-18	up-regulated	protein_coding
SERHL2	ENSMMUG00000031009	92.29005508	4.445999732	0.489044	9.091208	9.79E-20	2.91E-18	up-regulated	protein_coding
RASGRP1	ENSMMUG00000018333	123.9583236	7.1096662	0.782142	9.089996	9.90E-20	2.94E-18	up-regulated	protein_coding
KIRREL3	ENSMMUG00000006931	30.51110486	5.212952822	0.574097	9.080264	1.08E-19	3.21E-18	up-regulated	protein_coding
AGR2	ENSMMUG00000017050	776.3570068	-3.888960739	0.4287	-9.07153	1.17E-19	3.46E-18	down-regulated	protein_coding
ATP2A1	ENSMMUG00000023526	40.99873594	4.568721702	0.504729	9.051824	1.41E-19	4.13E-18	up-regulated	protein_coding
SLC24A5	ENSMMUG00000018831	186.2471461	4.089423642	0.4519	9.049394	1.44E-19	4.21E-18	up-regulated	protein_coding
RANBP3L	ENSMMUG00000018820	34.9965538	5.824713696	0.64506	9.029725	1.72E-19	5.03E-18	up-regulated	protein_coding
TMOD4	ENSMMUG00000007903	83.3799466	4.572294703	0.506781	9.022237	1.84E-19	5.38E-18	up-regulated	protein_coding
FER1L5	ENSMMUG00000011482	96.49497095	3.912312609	0.434966	8.994534	2.37E-19	6.89E-18	up-regulated	protein_coding
MRAP	ENSMMUG00000010293	140.5876489	5.89758953	0.656194	8.987576	2.53E-19	7.33E-18	up-regulated	protein_coding
YLPM1	ENSMMUG00000004255	312.7601441	2.39512353	0.266705	8.98042	2.70E-19	7.80E-18	up-regulated	protein_coding
Y_RNA	ENSMMUG00000027354	41.44483899	9.240453784	1.030057	8.970819	2.94E-19	8.50E-18	up-regulated	misc_RNA
LIG3	ENSMMUG00000004794	146.5851968	2.93791466	0.327723	8.964632	3.11E-19	8.97E-18	up-regulated	protein_coding
YWHAQ	ENSMMUG00000000721	350.3937772	-4.352003851	0.486501	-8.94553	3.70E-19	1.06E-17	down-regulated	protein_coding
CAMK4	ENSMMUG00000011974	53.32562529	7.877263481	0.882376	8.927329	4.36E-19	1.25E-17	up-regulated	protein_coding
Y_RNA	ENSMMUG00000025492	26.57432839	10.62679853	1.193016	8.907507	5.22E-19	1.49E-17	up-regulated	misc_RNA
HMGCS1	ENSMMUG00000001226	535.2405815	-3.371087525	0.378774	-8.89999	5.59E-19	1.59E-17	down-regulated	protein_coding
DCAF13	ENSMMUG00000019405	348.9535328	3.446240025	0.387738	8.888059	6.22E-19	1.77E-17	up-regulated	protein_coding
DDX5	ENSMMUG00000009463	672.9830851	-2.661331489	0.299899	-8.87409	7.05E-19	2.00E-17	down-regulated	protein_coding
SLC10A1	ENSMMUG00000022409	40.40500873	7.705157687	0.868672	8.870041	7.31E-19	2.07E-17	up-regulated	protein_coding
KLHDC2	ENSMMUG00000029366	449.6765769	2.466564893	0.278178	8.866866	7.52E-19	2.12E-17	up-regulated	protein_coding
CFAP57	ENSMMUG00000002642	101.2195315	5.053924334	0.570127	8.864561	7.68E-19	2.16E-17	up-regulated	protein_coding
B2M	ENSMMUG00000003760	1070.363902	-4.773914201	0.540589	-8.83096	1.04E-18	2.90E-17	down-regulated	protein_coding
PDZD2	ENSMMUG00000013954	43.60898762	6.893361005	0.781112	8.825059	1.09E-18	3.05E-17	up-regulated	protein_coding
NFRKB	ENSMMUG00000017547	2022.252543	4.2460727	0.482789	8.79489	1.43E-18	3.99E-17	up-regulated	protein_coding
HTRA2	ENSMMUG00000002136	541.6225316	3.497722761	0.398598	8.775071	1.71E-18	4.75E-17	up-regulated	protein_coding
CD81	ENSMMUG00000002043	630.49448	-3.349421048	0.381938	-8.76954	1.79E-18	4.98E-17	down-regulated	protein_coding

Y_RNA	ENSMMUG00000026388	28.74076784	8.333056865	0.950534	8.766712	1.84E-18	5.09E-17	up-regulated	misc_RNA
GAPDH	ENSMMUG00000018679	4860.768124	-3.605355081	0.411641	-8.75849	1.98E-18	5.46E-17	down-regulated	protein_coding
RPS26	ENSMMUG00000017042	791.7153313	-5.665982801	0.649114	-8.7288	2.57E-18	7.07E-17	down-regulated	protein_coding
MPL	ENSMMUG00000003148	31.94095363	5.023808828	0.57702	8.706467	3.13E-18	8.56E-17	up-regulated	protein_coding
RBM4B	ENSMMUG00000010577	105.4417453	3.104131589	0.356736	8.70149	3.28E-18	8.93E-17	up-regulated	protein_coding
PDE7A	ENSMMUG00000011262	56.54236347	4.690583118	0.53995	8.687072	3.72E-18	1.01E-16	up-regulated	protein_coding
ANXA2	ENSMMUG00000012000	2085.252767	-3.78837317	0.436146	-8.68601	3.75E-18	1.02E-16	down-regulated	protein_coding
SDC1	ENSMMUG00000030922	896.7353365	-2.810286396	0.323754	-8.68033	3.95E-18	1.07E-16	down-regulated	protein_coding
CCDC28B	ENSMMUG00000015066	61.00861212	5.372765629	0.619136	8.67784	4.03E-18	1.09E-16	up-regulated	protein_coding
ANGPT2	ENSMMUG00000010139	36.49320304	7.206617262	0.830517	8.677264	4.05E-18	1.09E-16	up-regulated	protein_coding
GCNT7	ENSMMUG00000000076	33.10157673	6.855098299	0.791061	8.665699	4.49E-18	1.21E-16	up-regulated	protein_coding
HEXIM2	ENSMMUG00000000713	68.56720254	4.504468193	0.520134	8.660199	4.71E-18	1.26E-16	up-regulated	protein_coding
SRSF12	ENSMMUG00000014941	25.28431335	4.803435206	0.554949	8.655631	4.90E-18	1.31E-16	up-regulated	protein_coding
DHX32	ENSMMUG00000008542	292.1196775	2.371740953	0.27424	8.648421	5.22E-18	1.39E-16	up-regulated	protein_coding
FAM222A	ENSMMUG00000003941	79.90323965	4.157292998	0.481365	8.636459	5.80E-18	1.54E-16	up-regulated	protein_coding
SPTBN4	ENSMMUG00000020796	49.24351037	3.661266074	0.424388	8.627165	6.29E-18	1.67E-16	up-regulated	protein_coding
RUNX3	ENSMMUG00000002420	119.1310067	5.231810099	0.606541	8.62565	6.37E-18	1.69E-16	up-regulated	protein_coding
MEFV	ENSMMUG00000017969	78.0521747	4.685044645	0.543194	8.625	6.41E-18	1.70E-16	up-regulated	protein_coding
POLR3H	ENSMMUG00000001459	547.2277835	3.252836512	0.37749	8.617024	6.87E-18	1.81E-16	up-regulated	protein_coding
7SK	ENSMMUG00000035446	29.53471266	7.52981877	0.877231	8.583626	9.19E-18	2.41E-16	up-regulated	misc_RNA
PKHD1L1	ENSMMUG00000000975	20.45321009	7.051054468	0.824511	8.551804	1.21E-17	3.16E-16	up-regulated	protein_coding
SNAP47	ENSMMUG00000001869	336.4570043	3.555464286	0.416196	8.542769	1.31E-17	3.41E-16	up-regulated	protein_coding
WDFY2	ENSMMUG00000009421	89.90055622	3.829025319	0.448387	8.539563	1.35E-17	3.50E-16	up-regulated	protein_coding
ACTB	ENSMMUG00000009425	2009.80418	-4.0904873	0.479266	-8.53489	1.40E-17	3.64E-16	down-regulated	protein_coding
BCR	ENSMMUG00000023622	240.9238964	2.3542631	0.276481	8.51509	1.66E-17	4.31E-16	up-regulated	protein_coding
SLC12A5	ENSMMUG00000016553	23.07806398	7.453196273	0.875521	8.512867	1.70E-17	4.39E-16	up-regulated	protein_coding
OR52B6	ENSMMUG00000013488	93.14526452	10.44183071	1.226811	8.511357	1.72E-17	4.44E-16	up-regulated	protein_coding
LAPTM4A	ENSMMUG00000001582	333.0182185	-3.359973074	0.394817	-8.51019	1.74E-17	4.48E-16	down-regulated	protein_coding
MBP	ENSMMUG00000011333	57.88888698	3.342024784	0.393569	8.491596	2.04E-17	5.24E-16	up-regulated	protein_coding
SPINT2	ENSMMUG00000002158	1019.865234	-2.512729747	0.296333	-8.4794	2.26E-17	5.80E-16	down-regulated	protein_coding
CD52	ENSMMUG00000010240	104.3542248	5.021772335	0.592653	8.47338	2.38E-17	6.09E-16	up-regulated	protein_coding
GLIPR1	ENSMMUG00000009638	86.19049327	5.109306137	0.60401	8.458975	2.70E-17	6.85E-16	up-regulated	protein_coding
UBE2D3	ENSMMUG00000011201	1213.374751	1.918059514	0.226743	8.459164	2.69E-17	6.85E-16	up-regulated	protein_coding
FAM166A	ENSMMUG00000012162	12320.44341	4.846559867	0.572937	8.459156	2.69E-17	6.85E-16	up-regulated	protein_coding
ADAM12	ENSMMUG00000009661	23.5099294	7.731241303	0.914406	8.454936	2.79E-17	7.08E-16	up-regulated	protein_coding
PSMD5	ENSMMUG00000002213	142.1980872	4.448678365	0.528161	8.422951	3.67E-17	9.26E-16	up-regulated	protein_coding
FCGRT	ENSMMUG00000012273	426.1234747	-4.129185889	0.49095	-8.4106	4.08E-17	1.03E-15	down-regulated	protein_coding
TMEM194B	ENSMMUG00000001145	37.67616727	5.122166949	0.609355	8.405889	4.25E-17	1.06E-15	up-regulated	protein_coding
DBP	ENSMMUG00000022160	347.0174684	2.390360253	0.284901	8.390145	4.86E-17	1.21E-15	up-regulated	protein_coding
PPP3R1	ENSMMUG00000017460	135.2135324	2.191850854	0.261316	8.387752	4.96E-17	1.23E-15	up-regulated	protein_coding
F2RL2	ENSMMUG00000022988	32.58359779	7.249982575	0.865556	8.376096	5.47E-17	1.35E-15	up-regulated	protein_coding
GPR137	ENSMMUG00000010390	407.1611799	3.101798558	0.370679	8.36788	5.87E-17	1.44E-15	up-regulated	protein_coding
LSMEM2	ENSMMUG00000020118	285.5321133	4.551115134	0.54432	8.361105	6.21E-17	1.53E-15	up-regulated	protein_coding
SAT1	ENSMMUG00000008335	979.7434831	-2.560169136	0.306223	-8.36047	6.25E-17	1.53E-15	down-regulated	protein_coding
PSMC3IP	ENSMMUG00000009889	410.5640276	3.733764261	0.447004	8.352874	6.66E-17	1.63E-15	up-regulated	protein_coding
VTI1B	ENSMMUG00000015866	276.8182315	1.955972385	0.234453	8.342711	7.26E-17	1.77E-15	up-regulated	protein_coding
RPL39	ENSMMUG00000029299	43.65160907	7.117186123	0.854108	8.332887	7.89E-17	1.92E-15	up-regulated	protein_coding
PAQR3	ENSMMUG00000014501	434.9600314	4.336807427	0.520922	8.325248	8.42E-17	2.05E-15	up-regulated	protein_coding
FAM132B	ENSMMUG00000018907	34.13825411	4.620797868	0.55508	8.324561	8.46E-17	2.05E-15	up-regulated	protein_coding
PARP9	ENSMMUG00000016381	222.729973	3.659162421	0.439892	8.318327	8.92E-17	2.16E-15	up-regulated	protein_coding
TNFRSF17	ENSMMUG00000021520	61.73472741	6.451256279	0.77564	8.317335	9.00E-17	2.18E-15	up-regulated	protein_coding
ETNK2	ENSMMUG00000008462	48.06493379	3.289231482	0.396022	8.305681	9.92E-17	2.39E-15	up-regulated	protein_coding
RASA1	ENSMMUG00000003245	331.7866627	3.27961367	0.394891	8.305116	9.97E-17	2.40E-15	up-regulated	protein_coding
C13H2ORF40	ENSMMUG00000001697	30.65236269	6.843722197	0.825508	8.290318	1.13E-16	2.71E-15	up-regulated	protein_coding
Y_RNA	ENSMMUG00000026224	30.95637339	10.10899428	1.219485	8.289557	1.14E-16	2.73E-15	up-regulated	misc_RNA
TEX38	ENSMMUG00000004864	20.09995688	10.23382629	1.236208	8.278402	1.25E-16	2.98E-15	up-regulated	protein_coding
PPP2R5D	ENSMMUG00000004873	480.828606	1.940924047	0.234782	8.266918	1.37E-16	3.28E-15	up-regulated	protein_coding
NNAT	ENSMMUG00000019174	173.3059524	4.633628964	0.560799	8.262546	1.43E-16	3.38E-15	up-regulated	protein_coding
CREB3L4	ENSMMUG00000005008	91.50721703	3.697748212	0.448064	8.252725	1.55E-16	3.66E-15	up-regulated	protein_coding
ACKR4	ENSMMUG00000000526	64.55664413	4.770554867	0.578296	8.249329	1.59E-16	3.76E-15	up-regulated	protein_coding
TP53TG5	ENSMMUG00000003408	51.60574887	6.38198102	0.77472	8.23779	1.75E-16	4.12E-15	up-regulated	protein_coding
GREM1	ENSMMUG00000001810	29.64838005	6.881610382	0.835628	8.235256	1.79E-16	4.20E-15	up-regulated	protein_coding
MMP20	ENSMMUG00000011013	22.96089354	5.172672595	0.628442	8.230941	1.86E-16	4.35E-15	up-regulated	protein_coding
YIPF2	ENSMMUG00000004400	258.8052741	2.284349984	0.27762	8.228327	1.90E-16	4.43E-15	up-regulated	protein_coding
CYCS	ENSMMUG00000030295	330.7848734	-4.172377148	0.507408	-8.22293	1.99E-16	4.61E-15	down-regulated	protein_coding
CXCR6	ENSMMUG00000023587	90.85733241	7.807192975	0.950136	8.216918	2.09E-16	4.84E-15	up-regulated	protein_coding
AMBRA1	ENSMMUG00000011526	163.4885849	2.188036274	0.266646	8.205777	2.29E-16	5.28E-15	up-regulated	protein_coding
MTX1	ENSMMUG00000008506	531.1361577	3.740668782	0.458105	8.165532	3.20E-16	7.34E-15	up-regulated	protein_coding
OBSCN	ENSMMUG00000023096	266.9957503	4.966515316	0.608756	8.158461	3.39E-16	7.76E-15	up-regulated	protein_coding
U4	ENSMMUG00000034125	53.1249373	4.43163577	0.543616	8.152149	3.58E-16	8.15E-15	up-regulated	snRNA
TRAF3IP3	ENSMMUG00000022062	89.43181633	4.514828947	0.554175	8.14694	3.73E-16	8.49E-15	up-regulated	protein_coding
SRRM5	ENSMMUG00000028754	217.9414977	4.056505284	0.498185	8.142571	3.87E-16	8.79E-15	up-regulated	protein_coding
SNORD10	ENSMMUG00000037310	19.6724286	5.543237708	0.681538	8.133428	4.17E-16	9.45E-15	up-regulated	snoRNA
KCNIP4	ENSMMUG00000030380	46.23799928	4.607759378	0.56862	8.103409	5.34E-16	1.21E-14	up-regulated	protein_coding
ZNF38	ENSMMUG00000014082	95.06765864	2.533759446	0.31301	8.094813	5.74E-16	1.29E-14	up-regulated	protein_coding
CA14	ENSMMUG00000021385	29.02133489	7.226220481	0.893913	8.083805	6.28E-16	1.41E-14	up-regulated	protein_coding
MSTO1	ENSMMUG00000009981	302.1147411	3.424069779	0.423958	8.076445	6.67E-16	1.49E-14	up-regulated	protein_coding
CD3EAP	ENSMMUG00000000717	204.8109439	4.405256492	0.545795	8.071269	6.96E-16	1.55E-14	up-regulated	protein_coding
7SK	ENSMMUG00000036718	38.52178039	9.697662679	1.201566	8.070851	6.98E-16	1.55E-14	up-regulated	misc_RNA
FARP2	ENSMMUG00000001313	113.0916339	2.445966493	0.30357	8.057338	7.80E-16	1.73E-14	up-regulated	protein_coding
U4	ENSMMUG00000035316	40.97489747	6.968088123	0.865261	8.053159	8.07E-16	1.79E-14	up-regulated	snRNA
TMEM262	ENSMMUG00000018771	76.10501077	3.991047384	0.49607	8.045331	8.60E-16	1.90E-14	up-regulated	protein_coding
U6	ENSMMUG00000034815	18.27423938	10.09001396	1.254243	8.044701	8.65E-16	1.91E-14	up-regulated	snRNA
POU5F1	ENSMMUG00000015688	37.56647798	6.103540866	0.760882	8.021669	1.04E-15	2.29E-14	up-regulated	protein_coding
snoU13	ENSMMUG00000036923	21.53800186	10.36278752	1.294465	8.005459	1.19E-15	2.61E-14	up-regulated	snoRNA
APOBEC2	ENSMMUG00000021171	22.04750489	5.8246857	0.727735	8.00385	1.21E-15	2.64E-14	up-regulated	protein_coding
OPN1SW	ENSMMUG00000009620	36.48955041	5.707866669	0.713383	8.001121	1.23E-15	2.69E-14	up-regulated	protein_coding
C1orf145	ENSMMUG00000023088	34.30347952	7.076200895	0.884752	7.997951	1.27E-15	2.76E-14	up-regulated	protein_coding
ZFHX3	ENSMMUG00000021041	223.1749301	4.240730131	0.530969	7.986769	1.39E-15	3.02E-14	up-regulated	protein_coding
RAB1A	ENSMMUG00000021721	256.1056445	-4.308303471	0.539639	-7.98367	1.42E-15	3.09E-14	down-regulated	protein_coding
CKMT2	ENSMMUG00000006932	63.79941598	6.838167921	0.856551	7.983374	1.42E-15	3.09E-14	up-regulated	protein_coding
TAF5	ENSMMUG00000011523	165.7573634	4.238741308	0.531026	7.982173	1.44E-15	3.12E-14	up-regulated	protein_coding
ZBTB32	ENSMMUG00000002375	26.00750451	5.559738437	0.697854	7.966907	1.63E-15	3.52E-14	up-regulated	protein_coding
5S_rRNA	ENSMMUG00000026532	19.86737386	7.426837026	0.932571	7.963834	1.67E-15	3.60E-14	up-regulated	rRNA
PPP1CC	ENSMMUG00000013664	277.8122142	-4.79120022	0.601795	-7.96151	1.70E-15	3.67E-14	down-regulated	protein_coding
CSRNP2	ENSMMUG00000012457	204.3837046	2.730659144	0.343531	7.948801	1.88E-15	4.05E-14	up-regulated	protein_coding
CLDN15	ENSMMUG00000007836	353.2618618	-5.660991104	0.71254	-7.94481	1.94E-15	4.17E-14	down-regulated	protein_coding
BCAN	ENSMMUG00000017093	162.545139	5.198932552	0.654857	7.939032	2.04E-15	4.36E-14	up-regulated	protein_coding
MAK16	ENSMMUG00000010244	233.008925	3.560328228	0.448734	7.934163	2.12E-15	4.52E-14	up-regulated	protein_coding
ASTE1	ENSMMUG00000005142	108.9824831	3.127796415	0.394326	7.931997	2.16E-15	4.60E-14	up-regulated	protein_coding

SLC12A1	ENSMMUG00000016896	18.50172359	10.1037035	1.275637	7.920514	2.37E-15	5.03E-14	up-regulated	protein_coding
GNAS1	ENSMMUG00000010266	1126.243717	-2.800820788	0.353898	-7.91421	2.49E-15	5.28E-14	down-regulated	protein_coding
FTL	ENSMMUG00000003909	2739.148978	-3.165313229	0.400252	-7.9083	2.61E-15	5.52E-14	down-regulated	protein_coding
PGK1	ENSMMUG00000012874	804.4693683	-3.796721076	0.480719	-7.89801	2.83E-15	5.98E-14	down-regulated	protein_coding
KIAA2013	ENSMMUG00000012469	295.8972845	-2.872063675	0.363753	-7.89565	2.89E-15	6.09E-14	down-regulated	protein_coding
OR51G2	ENSMMUG00000013811	16.8248571	9.98579079	1.27343	7.841647	4.45E-15	9.31E-14	up-regulated	protein_coding
HSPA5	ENSMMUG00000005217	883.7894	-2.035717262	0.259782	-7.83627	4.64E-15	9.67E-14	down-regulated	protein_coding
NRIP2	ENSMMUG00000007424	44.60354796	6.431704857	0.821102	7.833017	4.76E-15	9.91E-14	up-regulated	protein_coding
CHRNG	ENSMMUG00000022149	64.28746885	6.208618712	0.793315	7.826173	5.03E-15	1.04E-13	up-regulated	protein_coding
RNMTL1	ENSMMUG00000011423	360.7911384	4.468600555	0.571227	7.822804	5.17E-15	1.07E-13	up-regulated	protein_coding
OR2H2	ENSMMUG00000021482	100.0228936	4.521620358	0.578678	7.813713	5.55E-15	1.15E-13	up-regulated	protein_coding
DBNDD1	ENSMMUG00000000398	53.07893113	5.180166057	0.663036	7.812793	5.59E-15	1.15E-13	up-regulated	protein_coding
ESR2	ENSMMUG00000022839	26.03858095	8.065480945	1.033499	7.80405	6.00E-15	1.23E-13	up-regulated	protein_coding
ANGPTL7	ENSMMUG00000006187	29.45252949	6.483305589	0.831005	7.801763	6.10E-15	1.25E-13	up-regulated	protein_coding
C14orf178	ENSMMUG00000005403	47.86803544	4.290729489	0.550214	7.798295	6.27E-15	1.28E-13	up-regulated	protein_coding
PORCN	ENSMMUG00000018895	34.10594444	3.756689888	0.481867	7.796114	6.38E-15	1.31E-13	up-regulated	protein_coding
LHX4	ENSMMUG00000012050	22.77279242	6.705154535	0.860335	7.793653	6.51E-15	1.33E-13	up-regulated	protein_coding
Y_RNA	ENSMMUG00000027954	20.57664812	7.459830617	0.957526	7.790737	6.66E-15	1.36E-13	up-regulated	misc_RNA
RPH3A	ENSMMUG00000012778	18.88939612	10.09730293	1.296471	7.788296	6.79E-15	1.38E-13	up-regulated	protein_coding
RTN4	ENSMMUG00000021299	288.1647201	-3.251396205	0.418374	-7.7715	7.76E-15	1.57E-13	down-regulated	protein_coding
PHLPP2	ENSMMUG00000012007	553.6928134	3.847618765	0.495132	7.770896	7.79E-15	1.58E-13	up-regulated	protein_coding
ZCCHC3	ENSMMUG00000005262	129.971976	2.906709077	0.374298	7.765763	8.12E-15	1.64E-13	up-regulated	protein_coding
PLCD4	ENSMMUG00000009626	58.15959757	4.967532534	0.641017	7.749457	9.23E-15	1.85E-13	up-regulated	protein_coding
REC114	ENSMMUG00000009867	26.65898832	6.790249558	0.876453	7.747423	9.38E-15	1.88E-13	up-regulated	protein_coding
EIF1	ENSMMUG00000019505	344.0604536	-3.522569533	0.454988	-7.74212	9.78E-15	1.95E-13	down-regulated	protein_coding
INO80D	ENSMMUG00000001053	72.78167231	3.837573376	0.495824	7.739794	9.96E-15	1.99E-13	up-regulated	protein_coding
TSPAN3	ENSMMUG00000010172	340.2698139	-2.726263079	0.352645	-7.73091	1.07E-14	2.13E-13	down-regulated	protein_coding
SNORA48	ENSMMUG00000035716	15.91199834	9.902320307	1.282741	7.719657	1.17E-14	2.31E-13	up-regulated	snoRNA
CRMP1	ENSMMUG00000019563	147.3729161	7.808749707	1.011894	7.716961	1.19E-14	2.36E-13	up-regulated	protein_coding
GHRL	ENSMMUG00000019935	23.81727591	7.353706934	0.953172	7.714987	1.21E-14	2.39E-13	up-regulated	protein_coding
7SK	ENSMMUG00000024734	28.95737774	5.761228796	0.747006	7.712426	1.23E-14	2.44E-13	up-regulated	misc_RNA
ANGPTL1	ENSMMUG00000017553	21.65434982	7.939486714	1.029954	7.708584	1.27E-14	2.51E-13	up-regulated	protein_coding
RALBP1	ENSMMUG00000019521	470.2918261	1.803478379	0.234027	7.706274	1.30E-14	2.55E-13	up-regulated	protein_coding
TMED2	ENSMMUG00000031491	233.7954026	-4.229105923	0.548782	-7.70635	1.29E-14	2.55E-13	down-regulated	protein_coding
SNORA17	ENSMMUG00000032744	21.88519356	6.780628313	0.879939	7.705794	1.30E-14	2.55E-13	up-regulated	snoRNA
CSMD1	ENSMMUG00000023475	22.1346443	7.678138451	0.997672	7.696053	1.40E-14	2.75E-13	up-regulated	protein_coding
ERMN	ENSMMUG00000003345	22.87033947	6.925736327	0.900131	7.694141	1.42E-14	2.78E-13	up-regulated	protein_coding
CCDC108	ENSMMUG00000000783	90.14455321	6.2619785	0.815674	7.67706	1.63E-14	3.17E-13	up-regulated	protein_coding
CALM1	ENSMMUG00000017466	259.7918277	-4.752157981	0.619569	-7.6701	1.72E-14	3.34E-13	down-regulated	protein_coding
ACAP1	ENSMMUG00000006211	40.95510851	5.265702887	0.689036	7.642134	2.14E-14	4.15E-13	up-regulated	protein_coding
HIST1H1B	ENSMMUG00000001442	132.6449884	8.855060288	1.158802	7.641564	2.15E-14	4.16E-13	up-regulated	protein_coding
CHRND	ENSMMUG00000022147	18.78895569	6.111107502	0.799938	7.639477	2.18E-14	4.22E-13	up-regulated	protein_coding
CCDC65	ENSMMUG00000006970	67.20301828	4.891345509	0.641089	7.629748	2.35E-14	4.55E-13	up-regulated	protein_coding
FAM69A	ENSMMUG00000005073	166.2928914	3.735513569	0.489981	7.623793	2.46E-14	4.75E-13	up-regulated	protein_coding
RAD52	ENSMMUG00000018654	69.71788624	3.635861082	0.47702	7.622024	2.50E-14	4.81E-13	up-regulated	protein_coding
TM9SF2	ENSMMUG00000019556	299.2882608	-3.402018555	0.446557	-7.61833	2.57E-14	4.95E-13	down-regulated	protein_coding
PRSS27	ENSMMUG00000015943	16.84078311	6.965853502	0.914536	7.616817	2.60E-14	5.00E-13	up-regulated	protein_coding
SERPINE3	ENSMMUG00000013190	25.54803955	5.683406527	0.74653	7.613099	2.68E-14	5.13E-13	up-regulated	protein_coding
BRWD3	ENSMMUG00000010763	274.3180249	3.692535829	0.485245	7.609638	2.75E-14	5.26E-13	up-regulated	protein_coding
GON4L	ENSMMUG00000009987	258.2589877	2.238460978	0.294479	7.601424	2.93E-14	5.59E-13	up-regulated	protein_coding
snoU13	ENSMMUG00000035951	25.39963897	8.125179581	1.069008	7.60067	2.95E-14	5.61E-13	up-regulated	snoRNA
AP4M1	ENSMMUG00000013447	183.7968804	2.979274127	0.392109	7.598068	3.01E-14	5.71E-13	up-regulated	protein_coding
snoU13	ENSMMUG00000036456	19.67692873	9.433996659	1.241635	7.598044	3.01E-14	5.71E-13	up-regulated	snoRNA
KCNK7	ENSMMUG00000007402	20.5237878	5.20337461	0.685609	7.589423	3.21E-14	6.09E-13	up-regulated	protein_coding
HIST1H2AJ	ENSMMUG00000029900	29.56306647	10.09772408	1.330633	7.588663	3.23E-14	6.11E-13	up-regulated	protein_coding
MYH15	ENSMMUG00000019977	235.6238651	5.998321472	0.79178	7.575745	3.57E-14	6.73E-13	up-regulated	protein_coding
MKRN1	ENSMMUG00000010420	266.9963951	-2.906249357	0.383762	-7.57305	3.65E-14	6.86E-13	down-regulated	protein_coding
MT1F	ENSMMUG00000010373	180.0717028	5.505171521	0.727202	7.570342	3.72E-14	6.99E-13	up-regulated	protein_coding
GPR182	ENSMMUG00000021114	21.01532092	8.334241861	1.103135	7.555049	4.19E-14	7.84E-13	up-regulated	protein_coding
DOCK3	ENSMMUG00000004273	20.80072919	6.178761286	0.818374	7.550044	4.35E-14	8.12E-13	up-regulated	protein_coding
TXNDC17	ENSMMUG00000003788	175.0735289	2.025552192	0.268869	7.533594	4.94E-14	9.15E-13	up-regulated	protein_coding
CRYBA1	ENSMMUG00000012855	26.34601536	5.976788152	0.794658	7.52121	5.43E-14	1.00E-12	up-regulated	protein_coding
C8ORF41	ENSMMUG00000010246	256.5778236	3.254195277	0.432844	7.518165	5.56E-14	1.02E-12	up-regulated	protein_coding
RAC1	ENSMMUG00000021075	443.0363872	-2.672118588	0.355441	-7.51775	5.57E-14	1.03E-12	down-regulated	protein_coding
C12H2ORF62	ENSMMUG00000031218	45.21822988	5.215064346	0.693904	7.51554	5.67E-14	1.04E-12	up-regulated	protein_coding
HEATR4	ENSMMUG00000000313	92.1072551	4.418160425	0.588097	7.512637	5.79E-14	1.06E-12	up-regulated	protein_coding
HS6ST1	ENSMMUG00000030412	40.21614492	8.846417406	1.17755	7.512561	5.80E-14	1.06E-12	up-regulated	protein_coding
FAM20A	ENSMMUG00000002195	57.25826949	7.009420974	0.934496	7.500751	6.35E-14	1.16E-12	up-regulated	protein_coding
KRT40	ENSMMUG00000001142	77.39043132	8.284256441	1.104899	7.497753	6.49E-14	1.19E-12	up-regulated	protein_coding
PRG4	ENSMMUG00000009076	18.1246868	5.714585302	0.762459	7.494938	6.63E-14	1.21E-12	up-regulated	protein_coding
PDIA3	ENSMMUG00000001682	653.3859418	-3.1649225	0.422309	-7.49432	6.66E-14	1.22E-12	down-regulated	protein_coding
LRRC71	ENSMMUG00000012431	17.09339753	7.607340537	1.015362	7.492245	6.77E-14	1.23E-12	up-regulated	protein_coding
SNORA12	ENSMMUG00000032988	24.42600991	9.055475971	1.209207	7.488775	6.95E-14	1.26E-12	up-regulated	snoRNA
KRT39	ENSMMUG00000001510	45.88606657	9.408238426	1.256776	7.48601	7.10E-14	1.29E-12	up-regulated	protein_coding
CTSW	ENSMMUG00000008649	25.37262972	6.192218766	0.827272	7.485102	7.15E-14	1.30E-12	up-regulated	protein_coding
FBXL16	ENSMMUG00000016906	71.21870936	3.95580729	0.528556	7.484183	7.20E-14	1.30E-12	up-regulated	protein_coding
EEF1A1	ENSMMUG00000004852	8263.45597	-3.132508243	0.418907	-7.47781	7.56E-14	1.36E-12	down-regulated	protein_coding
Telomerase-vert	ENSMMUG00000024619	19.11658048	8.16967704	1.093267	7.472716	7.86E-14	1.41E-12	up-regulated	misc_RNA
RHOA	ENSMMUG00000012962	294.0687197	-2.642257559	0.353596	-7.47253	7.87E-14	1.41E-12	down-regulated	protein_coding
TMOD1	ENSMMUG00000012278	106.3694886	3.722947037	0.498261	7.471886	7.91E-14	1.42E-12	up-regulated	protein_coding
PHLDB1	ENSMMUG00000002544	591.5802141	3.110064911	0.416794	7.461874	8.53E-14	1.53E-12	up-regulated	protein_coding
ADCY7	ENSMMUG00000003118	134.9493201	3.530146176	0.473531	7.454944	8.99E-14	1.61E-12	up-regulated	protein_coding
ST14	ENSMMUG00000018034	1158.476782	-2.002163525	0.268766	-7.44946	9.37E-14	1.67E-12	down-regulated	protein_coding
WBP2	ENSMMUG00000009381	248.2049338	-4.398202103	0.590793	-7.44458	9.73E-14	1.73E-12	down-regulated	protein_coding
APOBEC4	ENSMMUG00000031303	17.68472431	8.001038362	1.076696	7.431105	1.08E-13	1.92E-12	up-regulated	protein_coding
CAPN1	ENSMMUG00000013475	424.7623134	-2.309803365	0.311431	-7.41675	1.20E-13	2.13E-12	down-regulated	protein_coding
TMBIM1	ENSMMUG00000022817	552.5864711	-2.728511489	0.3679	-7.41645	1.20E-13	2.14E-12	down-regulated	protein_coding
ATP6	ENSMMUG00000028683	5648.225018	12.33608766	1.66365	7.415076	1.22E-13	2.16E-12	up-regulated	protein_coding
TMPRSS6	ENSMMUG00000000151	28.73105268	5.521311254	0.745045	7.41071	1.26E-13	2.22E-12	up-regulated	protein_coding
TM9SF3	ENSMMUG00000001874	417.5326249	-2.808299525	0.379182	-7.40621	1.30E-13	2.30E-12	down-regulated	protein_coding
DOK6	ENSMMUG00000018275	17.13950668	9.947973303	1.344794	7.397396	1.39E-13	2.45E-12	up-regulated	protein_coding
U6	ENSMMUG00000033251	18.00363652	9.321465472	1.261493	7.389235	1.48E-13	2.60E-12	up-regulated	snRNA
CLDN7	ENSMMUG00000010551	1033.092604	-2.673095706	0.361867	-7.38696	1.50E-13	2.64E-12	down-regulated	protein_coding
DUOXA1	ENSMMUG00000008748	266.9349348	5.120248547	0.693619	7.381935	1.56E-13	2.74E-12	up-regulated	protein_coding
PPIA	ENSMMUG00000016638	1389.555537	-4.714076132	0.63867	-7.38108	1.57E-13	2.76E-12	down-regulated	protein_coding
EIF4G2	ENSMMUG00000011436	1081.930929	-1.735329484	0.235335	-7.37388	1.66E-13	2.91E-12	down-regulated	protein_coding
GTF3A	ENSMMUG00000009029	180.5241395	2.781230744	0.37743	7.368863	1.72E-13	3.01E-12	up-regulated	protein_coding
7SK	ENSMMUG00000035181	14.95038423	6.756559168	0.917326	7.365495	1.76E-13	3.09E-12	up-regulated	misc_RNA
ANXA13	ENSMMUG00000018008	274.8908812	-4.757403038	0.646218	-7.36191	1.81E-13	3.16E-12	down-regulated	protein_coding
MTCH1	ENSMMUG00000021976	291.3135797	-3.298110053	0.448507	-7.35354	1.93E-13	3.36E-12	down-regulated	protein_coding

ABCB5	ENSMMUG00000007821	19.19843544	10.12168752	1.378768	7.341112	2.12E-13	3.67E-12	up-regulated	protein_coding
DMTF1	ENSMMUG00000010781	181.9212814	2.224603903	0.303418	7.331813	2.27E-13	3.92E-12	up-regulated	protein_coding
CLDN23	ENSMMUG00000003970	413.461443	-3.422309218	0.467378	-7.32236	2.44E-13	4.20E-12	down-regulated	protein_coding
mml-mir-147b	ENSMMUG00000028568	338.0900001	-3.743569727	0.511266	-7.32216	2.44E-13	4.21E-12	down-regulated	miRNA
TRPV2	ENSMMUG00000015614	113.6712299	4.87721972	0.666132	7.321699	2.45E-13	4.21E-12	up-regulated	protein_coding
GADD45A	ENSMMUG00000014808	356.5815551	-2.444694105	0.334519	-7.3081	2.71E-13	4.66E-12	down-regulated	protein_coding
5S_rRNA	ENSMMUG00000024765	17.08916136	9.307519643	1.274311	7.303964	2.79E-13	4.80E-12	up-regulated	rRNA
MZF1	ENSMMUG00000017267	90.88855667	3.263021017	0.44676	7.303753	2.80E-13	4.80E-12	up-regulated	protein_coding
GNMT	ENSMMUG00000001393	73.10383185	5.201129911	0.712367	7.301196	2.85E-13	4.88E-12	up-regulated	protein_coding
GAPDHS	ENSMMUG00000000837	242.1386255	5.55568692	0.76099	7.300607	2.86E-13	4.90E-12	up-regulated	protein_coding
MYL6	ENSMMUG00000031838	736.7955708	-2.798021746	0.383516	-7.29571	2.97E-13	5.07E-12	down-regulated	protein_coding
MYH7B	ENSMMUG00000000227	68.60972883	3.614297243	0.495604	7.292711	3.04E-13	5.17E-12	up-regulated	protein_coding
C8ORF40	ENSMMUG00000020187	220.5449574	2.393888639	0.328424	7.289026	3.12E-13	5.31E-12	up-regulated	protein_coding
C20ORF165	ENSMMUG00000009957	30.00442432	4.312450411	0.591732	7.287845	3.15E-13	5.35E-12	up-regulated	protein_coding
OAT	ENSMMUG00000010485	423.3331538	-6.262356984	0.859616	-7.28506	3.22E-13	5.45E-12	down-regulated	protein_coding
ABI3	ENSMMUG00000002437	99.25937002	5.720669615	0.785756	7.280469	3.33E-13	5.64E-12	up-regulated	protein_coding
TMEM59	ENSMMUG00000007183	394.3766953	-3.043845279	0.418188	-7.27865	3.37E-13	5.71E-12	down-regulated	protein_coding
TM4SF4	ENSMMUG00000018980	327.1651956	-4.140939983	0.569063	-7.27677	3.42E-13	5.77E-12	down-regulated	protein_coding
HSD17B3	ENSMMUG00000007990	23.21120484	7.069475824	0.97165	7.275745	3.45E-13	5.81E-12	up-regulated	protein_coding
ATP5F1	ENSMMUG00000003313	278.7190003	-3.677102473	0.505528	-7.27379	3.50E-13	5.89E-12	down-regulated	protein_coding
NAV1	ENSMMUG00000001908	21.92229846	5.569289252	0.766927	7.261823	3.82E-13	6.42E-12	up-regulated	protein_coding
CMAS	ENSMMUG00000020808	213.6933716	-4.690193579	0.646297	-7.25702	3.96E-13	6.63E-12	down-regulated	protein_coding
VASH2	ENSMMUG00000011733	31.70887042	7.027344471	0.968687	7.254505	4.03E-13	6.75E-12	up-regulated	protein_coding
MROH2B	ENSMMUG00000004774	16.21531063	9.921369194	1.368158	7.251624	4.12E-13	6.88E-12	up-regulated	protein_coding
CAMK1	ENSMMUG00000006120	67.81979866	4.677001326	0.645011	7.251041	4.14E-13	6.90E-12	up-regulated	protein_coding
BPIL2	ENSMMUG00000006726	49.04628847	4.525659983	0.624768	7.243739	4.36E-13	7.27E-12	up-regulated	protein_coding
THRA	ENSMMUG00000002931	374.2170747	3.832631413	0.529149	7.243009	4.39E-13	7.30E-12	up-regulated	protein_coding
YBX1	ENSMMUG00000013136	1064.152584	-2.729023904	0.376995	-7.23889	4.52E-13	7.52E-12	down-regulated	protein_coding
GABARAP	ENSMMUG00000010545	333.557134	-3.886867154	0.537007	-7.23801	4.55E-13	7.56E-12	down-regulated	protein_coding
PERM1	ENSMMUG00000022525	98.64889708	4.185558247	0.578323	7.237408	4.57E-13	7.58E-12	up-regulated	protein_coding
MT-ND4L	ENSMMUG00000028678	1553.958267	15.77734539	2.179977	7.237392	4.57E-13	7.58E-12	up-regulated	protein_coding
RPS20	ENSMMUG00000021314	684.0424419	-6.57602547	0.90877	-7.23618	4.61E-13	7.64E-12	down-regulated	protein_coding
ENO4	ENSMMUG00000007575	30.37830555	5.906776029	0.816306	7.235983	4.62E-13	7.64E-12	up-regulated	protein_coding
TACR2	ENSMMUG00000015310	28.19261295	4.243602772	0.586805	7.231712	4.77E-13	7.87E-12	up-regulated	protein_coding
CC2D2A	ENSMMUG00000009383	52.38282357	2.999653444	0.415026	7.227633	4.91E-13	8.10E-12	up-regulated	protein_coding
TRIM46	ENSMMUG00000012182	573.188364	5.364567794	0.742873	7.221376	5.15E-13	8.48E-12	up-regulated	protein_coding
IFITM1	ENSMMUG00000013257	328.4542846	-5.929216225	0.821117	-7.22091	5.16E-13	8.50E-12	down-regulated	protein_coding
OS9	ENSMMUG00000020710	345.5499979	-2.351823453	0.325862	-7.21723	5.31E-13	8.71E-12	down-regulated	protein_coding
PLAC8	ENSMMUG00000022311	280.9250418	-4.201196637	0.582325	-7.21452	5.41E-13	8.87E-12	down-regulated	protein_coding
C4H6ORF62	ENSMMUG00000002682	289.1743122	-3.470956575	0.481218	-7.21285	5.48E-13	8.97E-12	down-regulated	protein_coding
CSPP1	ENSMMUG00000014423	90.53134573	3.631276088	0.503476	7.212407	5.50E-13	8.99E-12	up-regulated	protein_coding
MGST3	ENSMMUG00000015948	275.724691	-3.431718602	0.475816	-7.21227	5.50E-13	8.99E-12	down-regulated	protein_coding
ZFAND5	ENSMMUG00000005230	194.0676013	-4.289556849	0.594796	-7.21181	5.52E-13	9.01E-12	down-regulated	protein_coding
SEC22C	ENSMMUG00000021237	85.70885574	2.766716999	0.383937	7.20618	5.75E-13	9.38E-12	up-regulated	protein_coding
HSPD1	ENSMMUG00000010489	339.9156881	-4.390598694	0.609407	-7.20471	5.82E-13	9.47E-12	down-regulated	protein_coding
SC5DL	ENSMMUG00000007832	274.7330329	-4.475941671	0.621654	-7.20005	6.02E-13	9.79E-12	down-regulated	protein_coding
FANCL	ENSMMUG00000002026	81.15671113	2.796550395	0.388458	7.199105	6.06E-13	9.85E-12	up-regulated	protein_coding
FLG2	ENSMMUG00000012805	29.52636319	10.80883773	1.50204	7.196105	6.20E-13	1.00E-11	up-regulated	protein_coding
RAB11A	ENSMMUG00000008006	308.9336931	-2.459077253	0.342111	-7.18794	6.58E-13	1.06E-11	down-regulated	protein_coding
SNORA76	ENSMMUG00000036111	15.10129766	9.098241455	1.269193	7.168523	7.58E-13	1.22E-11	up-regulated	snoRNA
IL16	ENSMMUG00000011555	89.5818969	5.177358062	0.722752	7.163398	7.87E-13	1.26E-11	up-regulated	protein_coding
FAM212B	ENSMMUG00000014488	16.45165267	4.949392088	0.691436	7.158135	8.18E-13	1.31E-11	up-regulated	protein_coding
IGSF6	ENSMMUG00000013876	31.92577561	6.427338415	0.898062	7.156899	8.25E-13	1.32E-11	up-regulated	protein_coding
CLVS1	ENSMMUG00000008774	131.185024	3.762389091	0.526171	7.150504	8.65E-13	1.38E-11	up-regulated	protein_coding
SEC61A2	ENSMMUG00000017139	220.3067264	3.759814505	0.525947	7.148662	8.76E-13	1.40E-11	up-regulated	protein_coding
DCLRE1C	ENSMMUG00000017176	44.65747319	4.332728359	0.606166	7.147755	8.82E-13	1.40E-11	up-regulated	protein_coding
EIF5A	ENSMMUG00000006206	348.1705254	-4.99091419	0.698939	-7.1407	9.29E-13	1.47E-11	down-regulated	protein_coding
5S_rRNA	ENSMMUG00000032850	20.76099137	6.546832783	0.916969	7.139646	9.36E-13	1.48E-11	up-regulated	rRNA
CD160	ENSMMUG00000007969	31.14426304	5.495563999	0.770773	7.129936	1.00E-12	1.59E-11	up-regulated	protein_coding
ADAMTS18	ENSMMUG00000000824	36.37183871	6.376179015	0.894356	7.129349	1.01E-12	1.60E-11	up-regulated	protein_coding
SCP2	ENSMMUG00000004952	262.2062112	-4.379706357	0.614541	-7.12679	1.03E-12	1.62E-11	down-regulated	protein_coding
FAM69B	ENSMMUG00000032136	26.2798015	6.761287164	0.949118	7.123757	1.05E-12	1.65E-11	up-regulated	protein_coding
CLDN3	ENSMMUG00000001143	646.8959338	-2.821909075	0.396223	-7.12203	1.06E-12	1.67E-11	down-regulated	protein_coding
PCYT2	ENSMMUG00000012889	324.4171059	-3.20174451	0.449642	-7.12066	1.07E-12	1.68E-11	down-regulated	protein_coding
ATP5G3	ENSMMUG00000003954	262.7505319	-4.738723234	0.665842	-7.11689	1.10E-12	1.73E-11	down-regulated	protein_coding
TM4SF5	ENSMMUG00000012996	303.5688907	-4.565013344	0.641551	-7.11559	1.11E-12	1.74E-11	down-regulated	protein_coding
RYR2	ENSMMUG00000001060	14.36012645	7.301200295	1.026219	7.114661	1.12E-12	1.75E-11	up-regulated	protein_coding
GHITM	ENSMMUG00000018572	279.4750167	-4.877637446	0.685567	-7.11475	1.12E-12	1.75E-11	down-regulated	protein_coding
MAPK3	ENSMMUG00000017758	543.1665352	-1.727783612	0.243015	-7.10979	1.16E-12	1.81E-11	down-regulated	protein_coding
SDCBP2	ENSMMUG00000018165	406.7763481	-3.138036753	0.441886	-7.10147	1.23E-12	1.92E-11	down-regulated	protein_coding
DALRD3	ENSMMUG00000005652	661.9357519	3.413241542	0.481499	7.088788	1.35E-12	2.09E-11	up-regulated	protein_coding
BEST2	ENSMMUG00000006278	25.77939922	7.046334906	0.994025	7.088693	1.35E-12	2.09E-11	up-regulated	protein_coding
ICAM3	ENSMMUG00000019718	176.1546463	3.659186495	0.516308	7.087211	1.37E-12	2.11E-11	up-regulated	protein_coding
LGALS3	ENSMMUG00000003565	2271.17101	-4.667594696	0.659325	-7.07935	1.45E-12	2.23E-11	down-regulated	protein_coding
OR4D11	ENSMMUG00000014112	19.62743065	8.730796461	1.23577	7.065068	1.61E-12	2.47E-11	up-regulated	protein_coding
SLIT1	ENSMMUG00000010428	35.94143145	3.827289078	0.541983	7.06164	1.65E-12	2.52E-11	up-regulated	protein_coding
RPS5	ENSMMUG00000008729	694.5386835	-4.671658108	0.662133	-7.05547	1.72E-12	2.63E-11	down-regulated	protein_coding
MCC	ENSMMUG00000013557	32.34545706	6.813464463	0.965824	7.054561	1.73E-12	2.65E-11	up-regulated	protein_coding
C2orf16	ENSMMUG00000030890	31.31216624	6.700284078	0.951869	7.03908	1.94E-12	2.95E-11	up-regulated	protein_coding
TTC28	ENSMMUG00000001058	70.96314195	5.275283032	0.749701	7.036517	1.97E-12	3.00E-11	up-regulated	protein_coding
ARR3	ENSMMUG00000013935	19.29954026	6.540457695	0.929699	7.03503	1.99E-12	3.03E-11	up-regulated	protein_coding
CYP2A24	ENSMMUG00000021068	36.89381652	5.323098842	0.756788	7.033808	2.01E-12	3.05E-11	up-regulated	protein_coding
AKT3	ENSMMUG00000019995	89.22848572	4.979847773	0.708469	7.029024	2.08E-12	3.16E-11	up-regulated	protein_coding
DCST2	ENSMMUG00000023336	44.75319763	5.78768506	0.823783	7.025736	2.13E-12	3.23E-11	up-regulated	protein_coding
ACMSD	ENSMMUG00000010195	21.51306512	7.416151007	1.056728	7.01803	2.25E-12	3.41E-11	up-regulated	protein_coding
FUOM	ENSMMUG00000006324	273.8464775	-4.821246444	0.687004	-7.01778	2.25E-12	3.41E-11	down-regulated	protein_coding
LMOD2	ENSMMUG00000011542	17.03193889	7.547260003	1.07553	7.01725	2.26E-12	3.42E-11	up-regulated	protein_coding
TGFB1I1	ENSMMUG00000000896	48.97312565	4.612426032	0.657928	7.010534	2.37E-12	3.58E-11	up-regulated	protein_coding
LAMA2	ENSMMUG00000022136	21.67550597	5.408007387	0.772337	7.002137	2.52E-12	3.80E-11	up-regulated	protein_coding
BRP44	ENSMMUG00000010387	500.8411387	3.095156278	0.442226	6.999038	2.58E-12	3.88E-11	up-regulated	protein_coding
SH3BP1	ENSMMUG00000003854	359.4073962	-2.589426187	0.370086	-6.99682	2.62E-12	3.94E-11	down-regulated	protein_coding
FARP1	ENSMMUG00000009890	124.8708676	3.654689584	0.522469	6.995039	2.65E-12	3.98E-11	up-regulated	protein_coding
ZSWIM4	ENSMMUG00000017645	155.7079078	2.576086668	0.368331	6.993952	2.67E-12	4.01E-11	up-regulated	protein_coding
RNF32	ENSMMUG00000011748	19.76998328	4.842767733	0.692622	6.991938	2.71E-12	4.06E-11	up-regulated	protein_coding
LCTL	ENSMMUG00000017447	124.0812379	5.25692672	0.751898	6.991543	2.72E-12	4.07E-11	up-regulated	protein_coding
HIPK4	ENSMMUG00000006072	21.82087011	9.567405111	1.369392	6.986608	2.82E-12	4.21E-11	up-regulated	protein_coding
DNM1L	ENSMMUG00000013655	313.6637369	1.815464475	0.260081	6.980385	2.94E-12	4.39E-11	up-regulated	protein_coding
HSPA12B	ENSMMUG00000022077	123.5682058	5.362198364	0.768173	6.98046	2.94E-12	4.39E-11	up-regulated	protein_coding
ADARB2	ENSMMUG00000017120	20.09893008	8.270514232	1.185581	6.975915	3.04E-12	4.53E-11	up-regulated	protein_coding
TMEM66	ENSMMUG00000014143	213.5089055	-3.298538848	0.473224	-6.97036	3.16E-12	4.70E-11	down-regulated	protein_coding

TSSK4	ENSMMUG00000012496	86.532738	5.068418033	0.72721	6.969677	3.18E-12	4.72E-11	up-regulated	protein_coding
SNX21	ENSMMUG00000023515	157.1746719	2.258891973	0.324175	6.968122	3.21E-12	4.76E-11	up-regulated	protein_coding
PROZ	ENSMMUG00000001039	95.21611107	5.013877919	0.719731	6.966327	3.25E-12	4.82E-11	up-regulated	protein_coding
ATP2A2	ENSMMUG00000001739	239.6940101	-3.524006102	0.505865	-6.96629	3.25E-12	4.82E-11	down-regulated	protein_coding
IP6K3	ENSMMUG00000021732	12.56387562	9.535720111	1.369762	6.961586	3.36E-12	4.97E-11	up-regulated	protein_coding
GBA3	ENSMMUG00000005569	249.5900487	-4.488201412	0.645465	-6.95344	3.56E-12	5.26E-11	down-regulated	protein_coding
IFNGR2	ENSMMUG00000005508	319.847175	-4.358869871	0.628073	-6.94007	3.92E-12	5.77E-11	down-regulated	protein_coding
C21ORF2	ENSMMUG00000004174	558.8573813	3.434171767	0.495432	6.931664	4.16E-12	6.12E-11	up-regulated	protein_coding
RPL27A	ENSMMUG00000021828	956.2624196	-4.453517902	0.642529	-6.93123	4.17E-12	6.13E-11	down-regulated	protein_coding
DEGS2	ENSMMUG00000015437	253.3345744	-5.540476504	0.799743	-6.92782	4.27E-12	6.27E-11	down-regulated	protein_coding
U3	ENSMMUG00000036297	13.3222731	9.662257373	1.395283	6.924947	4.36E-12	6.39E-11	up-regulated	snoRNA
CDIPT	ENSMMUG00000018985	209.7539068	-4.156091369	0.600181	-6.92473	4.37E-12	6.40E-11	down-regulated	protein_coding
ARPC3	ENSMMUG00000006492	268.4406084	-3.315070528	0.479856	-6.90847	4.90E-12	7.13E-11	down-regulated	protein_coding
C8ORF42	ENSMMUG00000011108	42.77554504	6.441447656	0.932416	6.908343	4.90E-12	7.13E-11	up-regulated	protein_coding
CSF3	ENSMMUG00000002923	23.05597714	6.707006294	0.971295	6.905221	5.01E-12	7.27E-11	up-regulated	protein_coding
C9orf117	ENSMMUG00000016423	451.4831495	4.093913274	0.593127	6.90225	5.12E-12	7.42E-11	up-regulated	protein_coding
ANO7	ENSMMUG00000001721	126.5945548	2.70310237	0.391819	6.898854	5.24E-12	7.59E-11	up-regulated	protein_coding
Y_RNA	ENSMMUG00000028038	16.25405218	9.134826935	1.324876	6.894854	5.39E-12	7.80E-11	up-regulated	misc_RNA
KAZALD1	ENSMMUG00000017668	32.85534026	3.318454108	0.481582	6.890737	5.55E-12	8.02E-11	up-regulated	protein_coding
ZBTB5	ENSMMUG00000017894	140.25011	3.05648031	0.443582	6.890449	5.56E-12	8.03E-11	up-regulated	protein_coding
ZFYVE19	ENSMMUG00000001723	64.16467062	2.927703786	0.425079	6.887442	5.68E-12	8.18E-11	up-regulated	protein_coding
HNRNPA2B1	ENSMMUG00000003619	658.9629476	-3.548601712	0.515268	-6.88691	5.70E-12	8.21E-11	down-regulated	protein_coding
JUND	ENSMMUG00000028838	228.8172643	-4.361620656	0.634108	-6.87835	6.05E-12	8.69E-11	down-regulated	protein_coding
SDHC	ENSMMUG00000012411	212.1267751	-4.951747045	0.720024	-6.8772	6.10E-12	8.75E-11	down-regulated	protein_coding
MMEL1	ENSMMUG00000012381	99.21401505	3.772120058	0.548753	6.873987	6.24E-12	8.94E-11	up-regulated	protein_coding
SPSB3	ENSMMUG00000018596	431.0715621	3.011291889	0.43842	6.868505	6.49E-12	9.27E-11	up-regulated	protein_coding
PNPLA2	ENSMMUG00000020625	390.1687845	-2.832632353	0.413345	-6.85296	7.23E-12	1.03E-10	down-regulated	protein_coding
U11	ENSMMUG00000034248	13.43081059	8.914077173	1.303013	6.841127	7.86E-12	1.12E-10	up-regulated	snRNA
CTSH	ENSMMUG00000010093	262.5547163	-3.089690517	0.451684	-6.84038	7.90E-12	1.12E-10	down-regulated	protein_coding
Y_RNA	ENSMMUG00000028086	23.08208429	9.733001733	1.424258	6.833737	8.27E-12	1.17E-10	up-regulated	misc_RNA
UBE2V1	ENSMMUG00000012228	195.7991952	-2.715722912	0.397481	-6.83234	8.35E-12	1.18E-10	down-regulated	protein_coding
CLCF1	ENSMMUG00000017274	46.31465751	4.738269814	0.694282	6.824706	8.81E-12	1.25E-10	up-regulated	protein_coding
TSSK3	ENSMMUG00000000352	58.45798114	5.404395282	0.792855	6.816376	9.34E-12	1.32E-10	up-regulated	protein_coding
SSPN	ENSMMUG00000018630	77.17483017	5.039345185	0.739285	6.816517	9.33E-12	1.32E-10	up-regulated	protein_coding
UPB1	ENSMMUG00000000262	17.14524365	7.204275241	1.057404	6.813175	9.55E-12	1.35E-10	up-regulated	protein_coding
PCBP1	ENSMMUG00000013756	18.46142231	7.080475403	1.039668	6.810325	9.74E-12	1.37E-10	up-regulated	protein_coding
SYNE4	ENSMMUG00000019699	19.47914708	4.94265373	0.727275	6.79613	1.07E-11	1.51E-10	up-regulated	protein_coding
HDC	ENSMMUG00000002877	16.93339459	9.242768061	1.360807	6.792121	1.10E-11	1.55E-10	up-regulated	protein_coding
TAF9B	ENSMMUG00000012875	70.82341746	2.615952556	0.385148	6.792069	1.11E-11	1.55E-10	up-regulated	protein_coding
Y_RNA	ENSMMUG00000035977	23.66506649	5.004953821	0.737358	6.787681	1.14E-11	1.59E-10	up-regulated	misc_RNA
SLC13A4	ENSMMUG00000016386	65.02397466	6.842200139	1.008219	6.786425	1.15E-11	1.61E-10	up-regulated	protein_coding
HIST1H4K	ENSMMUG00000009629	18.08351538	6.915264921	1.019096	6.785683	1.16E-11	1.61E-10	up-regulated	protein_coding
GRIK1	ENSMMUG00000004886	13.55256244	9.650535231	1.422755	6.782991	1.18E-11	1.64E-10	up-regulated	protein_coding
LDHA	ENSMMUG00000031024	1513.434404	-2.634574935	0.388741	-6.77719	1.23E-11	1.71E-10	down-regulated	protein_coding
CAPZB	ENSMMUG00000006894	270.2110573	-3.287403337	0.485316	-6.77374	1.25E-11	1.75E-10	down-regulated	protein_coding
NOP16	ENSMMUG00000009317	653.4267093	2.428956624	0.358802	6.769623	1.29E-11	1.79E-10	up-regulated	protein_coding
CD80	ENSMMUG00000016367	21.53541259	6.972868773	1.030216	6.768358	1.30E-11	1.81E-10	up-regulated	protein_coding
THUMPD3	ENSMMUG00000017307	153.1533752	2.601784063	0.384629	6.764398	1.34E-11	1.86E-10	up-regulated	protein_coding
SYNC	ENSMMUG00000007498	22.79245722	4.058151854	0.600395	6.759135	1.39E-11	1.92E-10	up-regulated	protein_coding
RPS19	ENSMMUG00000013429	1034.327987	-4.720440201	0.699116	-6.75201	1.46E-11	2.02E-10	down-regulated	protein_coding
CLDN4	ENSMMUG00000009633	1841.358119	-3.162271026	0.468644	-6.7477	1.50E-11	2.08E-10	down-regulated	protein_coding
COL14A1	ENSMMUG00000002595	25.86298757	7.511762499	1.113675	6.74502	1.53E-11	2.11E-10	up-regulated	protein_coding
NINJ2	ENSMMUG00000013897	24.44877324	6.062041126	0.898735	6.745086	1.53E-11	2.11E-10	up-regulated	protein_coding
GSTO1	ENSMMUG00000015334	235.3406382	-4.03218052	0.598564	-6.73642	1.62E-11	2.23E-10	down-regulated	protein_coding
PEBP1	ENSMMUG00000005098	869.782731	-2.640163825	0.392209	-6.73152	1.68E-11	2.31E-10	down-regulated	protein_coding
Y_RNA	ENSMMUG00000025074	12.02454695	9.532114565	1.417016	6.726893	1.73E-11	2.38E-10	up-regulated	misc_RNA
RPL35	ENSMMUG00000005260	709.7452974	-5.204734116	0.773875	-6.72555	1.75E-11	2.40E-10	down-regulated	protein_coding
ANXA5	ENSMMUG00000009194	256.55221	-3.72786276	0.554383	-6.72434	1.76E-11	2.42E-10	down-regulated	protein_coding
PKDREJ	ENSMMUG00000018665	33.15550504	6.530582822	0.971328	6.723358	1.78E-11	2.43E-10	up-regulated	protein_coding
GLT8D2	ENSMMUG00000021292	15.12320837	4.939882875	0.73706	6.702148	2.05E-11	2.80E-10	up-regulated	protein_coding
GUCD1	ENSMMUG00000000263	223.4288329	-3.231503035	0.482194	-6.70166	2.06E-11	2.81E-10	down-regulated	protein_coding
UQCRC2	ENSMMUG00000018287	320.6606095	-2.320325483	0.346232	-6.70166	2.06E-11	2.81E-10	down-regulated	protein_coding
DISP2	ENSMMUG00000012408	22.64936164	5.3095167	0.792583	6.699003	2.10E-11	2.86E-10	up-regulated	protein_coding
C2orf82	ENSMMUG00000012214	15.88399184	6.770924848	1.011415	6.694504	2.16E-11	2.94E-10	up-regulated	protein_coding
FAM71D	ENSMMUG00000029318	19.02003267	8.675540406	1.295933	6.694436	2.17E-11	2.94E-10	up-regulated	protein_coding
SLAMF8	ENSMMUG00000011338	16.30322394	6.448228902	0.963955	6.689348	2.24E-11	3.04E-10	up-regulated	protein_coding
MVP	ENSMMUG00000016422	1072.133998	-2.270083206	0.339468	-6.68717	2.28E-11	3.08E-10	down-regulated	protein_coding
TMPRSS9	ENSMMUG00000001190	90.82407878	4.197976015	0.62841	6.680315	2.38E-11	3.23E-10	up-regulated	protein_coding
PLEKHH1	ENSMMUG00000021326	106.8134339	2.199994714	0.329443	6.677925	2.42E-11	3.28E-10	up-regulated	protein_coding
ZSCAN23	ENSMMUG00000012396	22.73286558	4.506158665	0.674809	6.677676	2.43E-11	3.28E-10	up-regulated	protein_coding
PHF17	ENSMMUG00000002145	37.04242376	3.310053461	0.49603	6.673094	2.50E-11	3.38E-10	up-regulated	protein_coding
NUTM1	ENSMMUG00000020938	42.11964144	5.686996917	0.852979	6.667219	2.61E-11	3.50E-10	up-regulated	protein_coding
PPFIA2	ENSMMUG00000013804	14.18563378	9.034278105	1.355504	6.664887	2.65E-11	3.56E-10	up-regulated	protein_coding
C10H22ORF23	ENSMMUG00000021560	29.1041661	3.86236722	0.579743	6.662204	2.70E-11	3.62E-10	up-regulated	protein_coding
SLC47A2	ENSMMUG00000014134	23.32873526	4.602271491	0.690857	6.66168	2.71E-11	3.63E-10	up-regulated	protein_coding
GPHA2	ENSMMUG00000003976	23.49583938	6.455418691	0.970021	6.654924	2.83E-11	3.79E-10	up-regulated	protein_coding
AGR3	ENSMMUG00000016017	191.2732659	-3.651911377	0.548999	-6.65195	2.89E-11	3.87E-10	down-regulated	protein_coding
RSPH9	ENSMMUG00000029639	18.37923674	4.144657096	0.624164	6.640338	3.13E-11	4.17E-10	up-regulated	protein_coding
BZW1	ENSMMUG00000022202	295.0967639	-2.333868029	0.352041	-6.62953	3.37E-11	4.48E-10	down-regulated	protein_coding
LRPPRC	ENSMMUG00000019841	220.7932785	-3.360362419	0.506912	-6.62908	3.38E-11	4.49E-10	down-regulated	protein_coding
U4	ENSMMUG00000027338	13.24644503	9.651576121	1.456482	6.626637	3.43E-11	4.55E-10	up-regulated	snRNA
ASF1A	ENSMMUG00000008526	108.5458069	3.277566517	0.494782	6.624265	3.49E-11	4.62E-10	up-regulated	protein_coding
HEBP1	ENSMMUG00000017633	187.3181304	-5.0912911	0.768704	-6.62321	3.51E-11	4.65E-10	down-regulated	protein_coding
KATNAL2	ENSMMUG00000015952	17.61989508	7.668553146	1.157964	6.622447	3.53E-11	4.67E-10	up-regulated	protein_coding
SNORA44	ENSMMUG00000035742	19.75913622	9.440851433	1.426341	6.618929	3.62E-11	4.78E-10	up-regulated	snoRNA
TSSK2	ENSMMUG00000017385	13.81229682	4.959607374	0.749704	6.61542	3.70E-11	4.88E-10	up-regulated	protein_coding
CEP290	ENSMMUG00000015862	69.80471494	2.264680987	0.342611	6.610074	3.84E-11	5.06E-10	up-regulated	protein_coding
NCOA3	ENSMMUG00000012020	139.8287411	2.16146949	0.32726	6.604748	3.98E-11	5.24E-10	up-regulated	protein_coding
COLQ	ENSMMUG00000031486	23.27988864	6.70010006	1.014596	6.603712	4.01E-11	5.27E-10	up-regulated	protein_coding
C12orf71	ENSMMUG00000004106	15.81515069	8.460800259	1.281857	6.600422	4.10E-11	5.38E-10	up-regulated	protein_coding
NR2C2	ENSMMUG00000005156	43.14017012	2.67507175	0.405617	6.59507	4.25E-11	5.57E-10	up-regulated	protein_coding
LIMD2	ENSMMUG00000016755	79.74494766	3.35655961	0.510295	6.577686	4.78E-11	6.24E-10	up-regulated	protein_coding
ZNF541	ENSMMUG00000014729	15.0891101	9.743234252	1.48186	6.575003	4.87E-11	6.35E-10	up-regulated	protein_coding
FAR2	ENSMMUG00000015810	124.7476635	2.040626911	0.31049	6.572284	4.95E-11	6.46E-10	up-regulated	protein_coding
ANXA7	ENSMMUG00000015708	155.8542684	-3.783321206	0.576099	-6.56713	5.13E-11	6.68E-10	down-regulated	protein_coding
IL22RA1	ENSMMUG00000023055	169.5983603	2.257602258	0.343774	6.567106	5.13E-11	6.68E-10	up-regulated	protein_coding
SDHA	ENSMMUG00000014452	186.6525303	-3.11116955	0.473898	-6.56506	5.20E-11	6.76E-10	down-regulated	protein_coding
LRRN4	ENSMMUG00000013745	70.21979948	4.356230251	0.663769	6.562872	5.28E-11	6.85E-10	up-regulated	protein_coding
RNASE4	ENSMMUG00000005496	332.7597327	-3.427154976	0.522291	-6.56177	5.32E-11	6.90E-10	down-regulated	protein_coding
CCDC3	ENSMMUG00000007820	28.83942206	5.707675982	0.870372	6.557743	5.46E-11	7.08E-10	up-regulated	protein_coding

MFRP	ENSMMUG00000005485	18.58259561	7.336595886	1.118948	6.556691	5.50E-11	7.12E-10	up-regulated	protein_coding
MYL12A	ENSMMUG00000019893	300.036573	-4.021883081	0.613842	-6.55199	5.68E-11	7.34E-10	down-regulated	protein_coding
MYO18B	ENSMMUG00000003334	18.84270318	9.366999816	1.429754	6.551475	5.70E-11	7.36E-10	up-regulated	protein_coding
7SK	ENSMMUG00000035006	25.95120622	6.085800306	0.9296	6.546684	5.88E-11	7.59E-10	up-regulated	misc_RNA
RPS4Y2	ENSMMUG00000003295	863.9459561	-3.688311797	0.563482	-6.54557	5.93E-11	7.64E-10	down-regulated	protein_coding
RPL27	ENSMMUG00000008090	350.7355764	-5.728806723	0.875572	-6.54293	6.03E-11	7.77E-10	down-regulated	protein_coding
DOC2A	ENSMMUG00000020277	87.19938596	3.44124193	0.526019	6.542054	6.07E-11	7.81E-10	up-regulated	protein_coding
TMEM204	ENSMMUG00000000045	27.47327049	6.582407015	1.007272	6.534883	6.37E-11	8.17E-10	up-regulated	protein_coding
RUSC2	ENSMMUG00000016313	122.2064784	2.858176809	0.437687	6.530185	6.57E-11	8.43E-10	up-regulated	protein_coding
NAIP	ENSMMUG00000020215	43.43214442	4.779190064	0.731875	6.530062	6.57E-11	8.43E-10	up-regulated	protein_coding
UBB	ENSMMUG00000023316	735.9509529	-2.158563149	0.330656	-6.52812	6.66E-11	8.53E-10	down-regulated	protein_coding
ANXA4	ENSMMUG00000000857	741.0260517	-2.503172808	0.383594	-6.52559	6.77E-11	8.67E-10	down-regulated	protein_coding
C14ORF164	ENSMMUG00000014743	15.03575365	6.156947523	0.943688	6.524344	6.83E-11	8.73E-10	up-regulated	protein_coding
ASAH1	ENSMMUG00000015497	400.5610914	-3.77315939	0.578664	-6.52046	7.01E-11	8.95E-10	down-regulated	protein_coding
HAPLN2	ENSMMUG00000019821	65.667118	3.579470918	0.548973	6.520302	7.02E-11	8.95E-10	up-regulated	protein_coding
ZFP2	ENSMMUG00000008978	17.97159144	3.905189807	0.59975	6.511362	7.45E-11	9.48E-10	up-regulated	protein_coding
CHMP4C	ENSMMUG00000002625	202.1183977	2.117718735	0.325442	6.507217	7.66E-11	9.72E-10	up-regulated	protein_coding
GNB2	ENSMMUG00000004080	181.4499919	-4.679619423	0.719204	-6.50667	7.68E-11	9.75E-10	down-regulated	protein_coding
KIAA1549	ENSMMUG00000006548	175.3752751	2.632657366	0.404689	6.505381	7.75E-11	9.83E-10	up-regulated	protein_coding
MYBPC2	ENSMMUG00000006715	35.62994491	5.953316112	0.915427	6.503323	7.86E-11	9.95E-10	up-regulated	protein_coding
SMS	ENSMMUG00000020392	214.834726	-2.60221904	0.400621	-6.49546	8.28E-11	1.05E-09	down-regulated	protein_coding
WFDC1	ENSMMUG00000004442	45.18014605	3.063723207	0.471705	6.495001	8.30E-11	1.05E-09	up-regulated	protein_coding
RPS8	ENSMMUG00000015270	1175.245296	-4.371637869	0.673232	-6.49351	8.39E-11	1.06E-09	down-regulated	protein_coding
MKNK2	ENSMMUG00000011265	190.2735103	-3.704644071	0.571027	-6.48769	8.72E-11	1.10E-09	down-regulated	protein_coding
CCNI2	ENSMMUG00000010308	111.261934	2.805288864	0.432696	6.48328	8.97E-11	1.13E-09	up-regulated	protein_coding
YWHAG	ENSMMUG00000004125	505.9566851	2.085404834	0.321799	6.480457	9.14E-11	1.15E-09	up-regulated	protein_coding
HCFC1R1	ENSMMUG00000007665	178.6998272	2.557295895	0.394758	6.478128	9.29E-11	1.16E-09	up-regulated	protein_coding
ARHGDIA	ENSMMUG00000009949	819.1330606	-2.504083931	0.386709	-6.47537	9.46E-11	1.19E-09	down-regulated	protein_coding
PRR13	ENSMMUG00000017738	995.158472	-3.209791463	0.496017	-6.47114	9.73E-11	1.22E-09	down-regulated	protein_coding
UPK1B	ENSMMUG00000008079	46.09182317	4.231140003	0.654082	6.468822	9.88E-11	1.23E-09	up-regulated	protein_coding
CDK2AP2	ENSMMUG00000011827	273.2099205	-3.026797809	0.467963	-6.46803	9.93E-11	1.24E-09	down-regulated	protein_coding
AGAP1	ENSMMUG00000009346	131.2465142	1.863016833	0.288051	6.467672	9.95E-11	1.24E-09	up-regulated	protein_coding
FGD1	ENSMMUG00000012613	44.6478116	3.501327079	0.542732	6.451299	1.11E-10	1.38E-09	up-regulated	protein_coding
DCLK2	ENSMMUG00000002211	22.74211084	5.882875347	0.912328	6.448205	1.13E-10	1.41E-09	up-regulated	protein_coding
SPTBN5	ENSMMUG00000003844	37.42446358	3.360920669	0.521332	6.446795	1.14E-10	1.42E-09	up-regulated	protein_coding
CCL25	ENSMMUG00000010614	312.7077706	-2.991003499	0.464157	-6.44395	1.16E-10	1.44E-09	down-regulated	protein_coding
MID1IP1	ENSMMUG00000003201	212.1143316	-3.494323579	0.542343	-6.44301	1.17E-10	1.45E-09	down-regulated	protein_coding
VSIG2	ENSMMUG00000008482	18.75188242	10.06559915	1.563146	6.439319	1.20E-10	1.49E-09	up-regulated	protein_coding
CFL1	ENSMMUG00000019016	1842.709361	-2.152271366	0.334358	-6.43702	1.22E-10	1.51E-09	down-regulated	protein_coding
TACSTD1	ENSMMUG00000012390	2213.800704	-4.043390986	0.628486	-6.43355	1.25E-10	1.54E-09	down-regulated	protein_coding
MST1	ENSMMUG00000014547	71.2953778	2.900870597	0.451034	6.431607	1.26E-10	1.56E-09	up-regulated	protein_coding
5S_rRNA	ENSMMUG00000034797	10.98916719	8.644764466	1.344738	6.428585	1.29E-10	1.59E-09	up-regulated	rRNA
PICALM	ENSMMUG00000019618	259.454153	-3.415715357	0.531395	-6.42783	1.29E-10	1.60E-09	down-regulated	protein_coding
HIST1H2AH	ENSMMUG00000001444	35.76449929	8.308253741	1.292796	6.426579	1.31E-10	1.61E-09	up-regulated	protein_coding
MLF2	ENSMMUG00000012206	305.1830411	-3.241365037	0.50448	-6.42516	1.32E-10	1.62E-09	down-regulated	protein_coding
RNF167	ENSMMUG00000002769	290.5382639	2.017978993	0.314113	6.424381	1.32E-10	1.63E-09	up-regulated	protein_coding
ADAMTSL4	ENSMMUG00000008218	108.127574	3.357306267	0.522629	6.423878	1.33E-10	1.63E-09	up-regulated	protein_coding
RORA	ENSMMUG00000018192	32.04440768	5.932035103	0.924803	6.414379	1.41E-10	1.74E-09	up-regulated	protein_coding
ADCY8	ENSMMUG00000010440	12.03868939	9.416948083	1.469452	6.408477	1.47E-10	1.80E-09	up-regulated	protein_coding
MPPE1	ENSMMUG00000011467	217.2667889	2.440686229	0.380904	6.407612	1.48E-10	1.81E-09	up-regulated	protein_coding
SPATA9	ENSMMUG00000001213	15.203993	4.281731417	0.668267	6.40722	1.48E-10	1.81E-09	up-regulated	protein_coding
SLC30A8	ENSMMUG00000004969	36.65883544	7.696260007	1.202019	6.402776	1.53E-10	1.87E-09	up-regulated	protein_coding
DNPEP	ENSMMUG00000017929	198.1186659	-4.25802556	0.665057	-6.40249	1.53E-10	1.87E-09	down-regulated	protein_coding
SNRNP25	ENSMMUG00000000587	315.6508524	3.387664437	0.529731	6.395065	1.60E-10	1.96E-09	up-regulated	protein_coding
C3orf17	ENSMMUG00000014876	84.98876934	3.032187583	0.474376	6.391949	1.64E-10	2.00E-09	up-regulated	protein_coding
AK5	ENSMMUG00000020218	47.39414794	5.453629869	0.853735	6.387966	1.68E-10	2.05E-09	up-regulated	protein_coding
AMN1	ENSMMUG00000011180	73.64890374	2.374933811	0.372237	6.380169	1.77E-10	2.15E-09	up-regulated	protein_coding
CCT2	ENSMMUG00000010820	251.5465175	-3.145339491	0.493425	-6.37451	1.84E-10	2.23E-09	down-regulated	protein_coding
NEXN	ENSMMUG00000010352	92.75610837	3.117958439	0.489414	6.3708	1.88E-10	2.28E-09	up-regulated	protein_coding
SVEP1	ENSMMUG00000001221	14.43922896	6.57029734	1.031507	6.36961	1.90E-10	2.30E-09	up-regulated	protein_coding
INCA1	ENSMMUG00000009175	33.31570701	4.792575616	0.752612	6.367924	1.92E-10	2.32E-09	up-regulated	protein_coding
GBP1	ENSMMUG00000000662	22.73373239	3.800151256	0.597397	6.361187	2.00E-10	2.41E-09	up-regulated	protein_coding
TRPM3	ENSMMUG00000006156	20.05393439	7.185738737	1.12969	6.360809	2.01E-10	2.42E-09	up-regulated	protein_coding
ZRANB1	ENSMMUG00000022028	146.7931461	2.719260325	0.427702	6.357845	2.05E-10	2.46E-09	up-regulated	protein_coding
U2	ENSMMUG00000034628	12.08170349	7.071308421	1.112863	6.35416	2.10E-10	2.52E-09	up-regulated	snRNA
NRXN3	ENSMMUG00000013842	16.94721738	4.886434069	0.769733	6.348217	2.18E-10	2.62E-09	up-regulated	protein_coding
DNMT3L	ENSMMUG00000010648	259.3815988	4.507949248	0.710374	6.345886	2.21E-10	2.66E-09	up-regulated	protein_coding
CSRNP3	ENSMMUG00000008393	25.25547865	4.622442614	0.728491	6.345228	2.22E-10	2.67E-09	up-regulated	protein_coding
SLC25A1	ENSMMUG00000005431	535.5173728	-3.685781436	0.581131	-6.34243	2.26E-10	2.71E-09	down-regulated	protein_coding
STK19	ENSMMUG00000000747	187.1271672	2.722108513	0.429611	6.336212	2.35E-10	2.82E-09	up-regulated	protein_coding
COL4A1	ENSMMUG00000022280	15.35752227	5.35967909	0.845883	6.336197	2.36E-10	2.82E-09	up-regulated	protein_coding
FANCI	ENSMMUG00000011155	383.1294492	3.338608701	0.526931	6.335946	2.36E-10	2.82E-09	up-regulated	protein_coding
JUP	ENSMMUG00000013048	793.8865476	-2.539849753	0.400982	-6.33407	2.39E-10	2.85E-09	down-regulated	protein_coding
snoU13	ENSMMUG00000035704	13.37958163	8.227588735	1.299106	6.33327	2.40E-10	2.86E-09	up-regulated	snoRNA
IQCC	ENSMMUG00000017718	72.39474395	3.150646722	0.498249	6.323441	2.56E-10	3.05E-09	up-regulated	protein_coding
5S_rRNA	ENSMMUG00000026798	11.4589479	5.848432504	0.924942	6.323027	2.56E-10	3.05E-09	up-regulated	rRNA
CDH17	ENSMMUG00000020010	1007.31664	-2.666508652	0.421746	-6.32255	2.57E-10	3.06E-09	down-regulated	protein_coding
FIP1L1	ENSMMUG00000017395	206.0291151	1.929366036	0.305222	6.321199	2.60E-10	3.08E-09	up-regulated	protein_coding
RAP1B	ENSMMUG00000011442	162.1111311	-3.675304742	0.581616	-6.31912	2.63E-10	3.12E-09	down-regulated	protein_coding
FAT2	ENSMMUG00000003497	15.11263497	9.039329354	1.431169	6.316045	2.68E-10	3.18E-09	up-regulated	protein_coding
TOPORS	ENSMMUG00000002686	125.2477354	1.964522824	0.311447	6.307737	2.83E-10	3.36E-09	up-regulated	protein_coding
ATP6V0C	ENSMMUG00000019094	227.669214	-5.066873976	0.803357	-6.30712	2.84E-10	3.37E-09	down-regulated	protein_coding
SNORD65	ENSMMUG00000032815	31.25602788	5.374081215	0.852185	6.306237	2.86E-10	3.38E-09	up-regulated	snoRNA
UBA1	ENSMMUG00000017428	575.1740074	-1.691079145	0.268659	-6.29451	3.08E-10	3.64E-09	down-regulated	protein_coding
ZSWIM8	ENSMMUG00000004604	405.1543099	1.567088544	0.249315	6.285565	3.27E-10	3.85E-09	up-regulated	protein_coding
HIST2H2AB	ENSMMUG00000031743	22.80014229	8.259332926	1.315598	6.278008	3.43E-10	4.03E-09	up-regulated	protein_coding
MMP19	ENSMMUG00000006295	63.14135753	4.322416299	0.688547	6.277588	3.44E-10	4.04E-09	up-regulated	protein_coding
CLGN	ENSMMUG00000022355	60.81598549	3.615578521	0.576285	6.273945	3.52E-10	4.13E-09	up-regulated	protein_coding
DNAH11	ENSMMUG00000022764	85.15682453	4.461673019	0.711198	6.273465	3.53E-10	4.14E-09	up-regulated	protein_coding
OPA3	ENSMMUG00000022940	58.5325243	3.2989334	0.525918	6.272719	3.55E-10	4.16E-09	up-regulated	protein_coding
CBLN3	ENSMMUG00000009515	20.79264778	4.964013933	0.791841	6.268952	3.63E-10	4.25E-09	up-regulated	protein_coding
KCNJ9	ENSMMUG00000009928	19.01317769	5.889767024	0.939925	6.266212	3.70E-10	4.32E-09	up-regulated	protein_coding
ZBTB8OS	ENSMMUG00000009939	158.2216239	1.897243623	0.302906	6.263482	3.76E-10	4.40E-09	up-regulated	protein_coding
snoU13	ENSMMUG00000035764	24.6454553	5.429248921	0.866829	6.263341	3.77E-10	4.40E-09	up-regulated	snoRNA
RPS6	ENSMMUG00000003412	1134.047824	-4.113904933	0.657007	-6.26159	3.81E-10	4.44E-09	down-regulated	protein_coding
SMARCD3	ENSMMUG00000006687	24.44198857	4.164014388	0.665275	6.259083	3.87E-10	4.51E-09	up-regulated	protein_coding
OR2D3	ENSMMUG00000009263	22.96473027	6.774275075	1.082365	6.258773	3.88E-10	4.51E-09	up-regulated	protein_coding
GPR146	ENSMMUG00000007208	35.45708339	3.850929718	0.615337	6.25824	3.89E-10	4.53E-09	up-regulated	protein_coding
APOC3	ENSMMUG00000016126	1083.327867	-3.86491695	0.617749	-6.25645	3.94E-10	4.58E-09	down-regulated	protein_coding
CDKN1A	ENSMMUG00000003210	588.9794825	-2.976722439	0.476048	-6.25298	4.03E-10	4.67E-09	down-regulated	protein_coding

PHYHIP	ENSMMUG00000013032	35.7417007	6.63717253	1.061601	6.252039	4.05E-10	4.69E-09	up-regulated	protein_coding
SRSF5	ENSMMUG00000022407	308.6069496	-3.775860258	0.603963	-6.25181	4.06E-10	4.70E-09	down-regulated	protein_coding
BDNF	ENSMMUG00000008634	15.98429957	7.96696141	1.274501	6.251045	4.08E-10	4.72E-09	up-regulated	protein_coding
Y_RNA	ENSMMUG00000026815	11.79356928	8.020712275	1.283896	6.247166	4.18E-10	4.83E-09	up-regulated	misc_RNA
F5	ENSMMUG00000010245	12.45912402	6.262972914	1.006008	6.22557	4.80E-10	5.52E-09	up-regulated	protein_coding
NUP210L	ENSMMUG00000014952	16.59500222	6.260262157	1.005569	6.225591	4.80E-10	5.52E-09	up-regulated	protein_coding
HSD17B11	ENSMMUG00000019041	470.7479096	-3.764858626	0.605205	-6.2208	4.95E-10	5.69E-09	down-regulated	protein_coding
TRMT10B	ENSMMUG00000008662	79.95351389	2.568627891	0.412989	6.219605	4.98E-10	5.72E-09	up-regulated	protein_coding
PLSCR1	ENSMMUG00000021756	304.0221118	-3.428951175	0.551762	-6.21455	5.15E-10	5.91E-09	down-regulated	protein_coding
MAP1A	ENSMMUG00000007255	18.97994228	6.222916427	1.002401	6.208011	5.37E-10	6.15E-09	up-regulated	protein_coding
5S_rRNA	ENSMMUG00000024111	12.31960276	8.022151669	1.292333	6.207496	5.38E-10	6.17E-09	up-regulated	rRNA
NTN3	ENSMMUG00000000004	12.41623139	6.740464521	1.086092	6.206164	5.43E-10	6.22E-09	up-regulated	protein_coding
TGM4	ENSMMUG00000003652	12.651041	9.482459117	1.528629	6.203246	5.53E-10	6.32E-09	up-regulated	protein_coding
CALCOCO2	ENSMMUG00000012178	189.1847713	-2.480029607	0.399793	-6.20329	5.53E-10	6.32E-09	down-regulated	protein_coding
FTSJD2	ENSMMUG00000011573	131.3956946	1.862601141	0.300336	6.201723	5.58E-10	6.38E-09	up-regulated	protein_coding
SRPK3	ENSMMUG00000003372	24.59658757	3.379855392	0.545119	6.200216	5.64E-10	6.43E-09	up-regulated	protein_coding
PAX4	ENSMMUG00000015429	17.36054031	7.429768758	1.200331	6.189765	6.03E-10	6.86E-09	up-regulated	protein_coding
RECQL5	ENSMMUG00000021635	183.0263132	2.36857469	0.382696	6.189185	6.05E-10	6.88E-09	up-regulated	protein_coding
DNAJB5	ENSMMUG00000022895	77.40346709	2.323259792	0.375396	6.188826	6.06E-10	6.89E-09	up-regulated	protein_coding
PBX3	ENSMMUG00000013763	81.68419704	2.995752542	0.484158	6.187555	6.11E-10	6.93E-09	up-regulated	protein_coding
TMEM49	ENSMMUG00000001741	235.2554823	-2.209497515	0.357225	-6.18518	6.20E-10	7.03E-09	down-regulated	protein_coding
HIST1H2AH	ENSMMUG00000029896	16.16936837	6.032711434	0.975702	6.182944	6.29E-10	7.13E-09	up-regulated	protein_coding
TSPAN18	ENSMMUG00000000368	27.89560181	5.809245771	0.939663	6.182262	6.32E-10	7.15E-09	up-regulated	protein_coding
ZFAND6	ENSMMUG00000009361	142.1913965	-4.027651842	0.651535	-6.18179	6.34E-10	7.17E-09	down-regulated	protein_coding
AGXT2L2	ENSMMUG00000003997	92.68933618	3.485018909	0.563856	6.180685	6.38E-10	7.21E-09	up-regulated	protein_coding
PLCZ1	ENSMMUG00000022175	11.19885358	9.34114243	1.512834	6.174598	6.63E-10	7.48E-09	up-regulated	protein_coding
U6	ENSMMUG00000032869	11.19885358	9.34114243	1.512834	6.174598	6.63E-10	7.48E-09	up-regulated	snRNA
CCDC176	ENSMMUG00000012683	18.37505281	4.599752704	0.745161	6.172835	6.71E-10	7.56E-09	up-regulated	protein_coding
7SK	ENSMMUG00000035052	185.7042538	-5.090874055	0.826006	-6.16324	7.13E-10	8.02E-09	down-regulated	misc_RNA
GNPDA2	ENSMMUG00000009273	54.65464304	2.398262079	0.389381	6.159167	7.31E-10	8.22E-09	up-regulated	protein_coding
PIAS4	ENSMMUG00000011018	102.0026707	2.065175045	0.335594	6.153797	7.56E-10	8.49E-09	up-regulated	protein_coding
EIF3F	ENSMMUG00000015010	219.8526098	-4.779065298	0.776841	-6.15192	7.66E-10	8.59E-09	down-regulated	protein_coding
CPA5	ENSMMUG00000009494	10.76525727	8.620424017	1.402176	6.147892	7.85E-10	8.80E-09	up-regulated	protein_coding
RAD9B	ENSMMUG00000013155	20.75921731	5.240281671	0.852708	6.145461	7.97E-10	8.92E-09	up-regulated	protein_coding
NTM	ENSMMUG00000020198	20.96527244	10.23689415	1.666024	6.144506	8.02E-10	8.96E-09	up-regulated	protein_coding
PNLIPRP1	ENSMMUG00000002724	11.98919132	8.67740508	1.413047	6.140916	8.20E-10	9.15E-09	up-regulated	protein_coding
COPZ1	ENSMMUG00000005182	167.7444787	-3.422853406	0.557453	-6.14017	8.24E-10	9.18E-09	down-regulated	protein_coding
snoU13	ENSMMUG00000035874	12.11451746	9.434932546	1.537149	6.137944	8.36E-10	9.30E-09	up-regulated	snoRNA
DDR2	ENSMMUG00000009330	15.91616203	5.217632677	0.850397	6.135529	8.49E-10	9.43E-09	up-regulated	protein_coding
SNPH	ENSMMUG00000018159	59.96824336	3.426287034	0.558436	6.135501	8.49E-10	9.43E-09	up-regulated	protein_coding
7SK	ENSMMUG00000035167	12.90567705	6.434226989	1.048732	6.135244	8.50E-10	9.44E-09	up-regulated	misc_RNA
Y_RNA	ENSMMUG00000026740	9.285970698	9.157146683	1.492907	6.133769	8.58E-10	9.52E-09	up-regulated	misc_RNA
STAB2	ENSMMUG00000006347	12.10346578	6.207394695	1.012183	6.132678	8.64E-10	9.57E-09	up-regulated	protein_coding
RASEF	ENSMMUG00000005988	118.3369295	2.531104512	0.412897	6.130104	8.78E-10	9.72E-09	up-regulated	protein_coding
HMGCR	ENSMMUG00000006139	448.4903361	-1.740219489	0.283911	-6.12946	8.82E-10	9.75E-09	down-regulated	protein_coding
TRIM16	ENSMMUG00000019200	297.0892411	1.625352741	0.265403	6.124097	9.12E-10	1.01E-08	up-regulated	protein_coding
GPR44	ENSMMUG00000000020	51.0334358	5.372830102	0.877432	6.123361	9.16E-10	1.01E-08	up-regulated	protein_coding
CDKL1	ENSMMUG00000002616	23.23636753	4.981481157	0.813528	6.123303	9.17E-10	1.01E-08	up-regulated	protein_coding
GPR183	ENSMMUG00000012338	42.54896596	7.844143588	1.281358	6.121744	9.26E-10	1.02E-08	up-regulated	protein_coding
FAM205A	ENSMMUG00000004980	18.93241605	7.679319502	1.254825	6.119835	9.37E-10	1.03E-08	up-regulated	protein_coding
BRIX1	ENSMMUG00000000442	154.7056457	2.836016786	0.463817	6.114515	9.69E-10	1.06E-08	up-regulated	protein_coding
ATP5C1	ENSMMUG00000009207	256.3422026	-7.40423941	1.211452	-6.11187	9.85E-10	1.08E-08	down-regulated	protein_coding
C10H20ORF118	ENSMMUG00000002089	93.13519162	2.764050234	0.452343	6.110524	9.93E-10	1.09E-08	up-regulated	protein_coding
BMX	ENSMMUG00000014047	25.13179853	5.370292313	0.880012	6.102524	1.04E-09	1.14E-08	up-regulated	protein_coding
Y_RNA	ENSMMUG00000026458	17.51115972	8.496240533	1.392472	6.101553	1.05E-09	1.15E-08	up-regulated	misc_RNA
IHH	ENSMMUG00000000785	161.5183797	-3.121199141	0.511766	-6.09889	1.07E-09	1.17E-08	down-regulated	protein_coding
LRP2	ENSMMUG00000017143	22.25706136	7.352717136	1.205663	6.098483	1.07E-09	1.17E-08	up-regulated	protein_coding
mml-mir-199a-2	ENSMMUG00000026921	10.64552292	5.297907353	0.869011	6.096478	1.08E-09	1.18E-08	up-regulated	miRNA
MST1R	ENSMMUG00000003353	283.2509836	-2.690190087	0.441628	-6.09152	1.12E-09	1.22E-08	down-regulated	protein_coding
KCTD4	ENSMMUG00000012636	12.37886917	7.102934333	1.166729	6.087906	1.14E-09	1.25E-08	up-regulated	protein_coding
TRIM62	ENSMMUG00000015097	95.79359759	2.197852154	0.361133	6.08599	1.16E-09	1.26E-08	up-regulated	protein_coding
TINF2	ENSMMUG00000029407	146.8345966	2.062759024	0.339154	6.08207	1.19E-09	1.29E-08	up-regulated	protein_coding
EAF1	ENSMMUG00000020761	104.1108256	2.747449189	0.452042	6.077865	1.22E-09	1.32E-08	up-regulated	protein_coding
CTNS	ENSMMUG00000023389	271.0798327	2.404390452	0.395804	6.074706	1.24E-09	1.35E-08	up-regulated	protein_coding
SSTR2	ENSMMUG00000020947	22.900233	3.91328181	0.644341	6.073305	1.25E-09	1.36E-08	up-regulated	protein_coding
PHF10	ENSMMUG00000015335	263.1341008	2.399138175	0.395367	6.068123	1.29E-09	1.40E-08	up-regulated	protein_coding
RTN4RL2	ENSMMUG00000010982	42.17206649	6.065795981	1.000052	6.065478	1.32E-09	1.42E-08	up-regulated	protein_coding
LRRC29	ENSMMUG00000020260	56.33183768	2.398665124	0.395678	6.06217	1.34E-09	1.45E-08	up-regulated	protein_coding
TUBA4A	ENSMMUG00000019628	210.6845158	-4.924143404	0.812862	-6.05779	1.38E-09	1.49E-08	down-regulated	protein_coding
HNRNPR	ENSMMUG00000010970	139.0495597	-3.630263734	0.599345	-6.05705	1.39E-09	1.49E-08	down-regulated	protein_coding
PDPK1	ENSMMUG00000011459	183.1584698	2.815998157	0.465279	6.052285	1.43E-09	1.54E-08	up-regulated	protein_coding
TFIP11	ENSMMUG00000023446	171.7962126	2.422416249	0.400429	6.049553	1.45E-09	1.56E-08	up-regulated	protein_coding
GBP3	ENSMMUG00000019251	49.52628088	4.372566204	0.722969	6.048065	1.47E-09	1.57E-08	up-regulated	protein_coding
DUSP6	ENSMMUG00000006227	371.5402217	-2.419781387	0.400217	-6.04617	1.48E-09	1.59E-08	down-regulated	protein_coding
FSCN2	ENSMMUG00000001583	27.62560986	4.475916167	0.740325	6.045878	1.49E-09	1.59E-08	up-regulated	protein_coding
SCG5	ENSMMUG00000011751	25.98841886	4.219449441	0.698308	6.042391	1.52E-09	1.63E-08	up-regulated	protein_coding
ARL4C	ENSMMUG00000011516	211.5105628	-2.646521412	0.438288	-6.03831	1.56E-09	1.67E-08	down-regulated	protein_coding
ZDBF2	ENSMMUG00000005636	20.7346503	7.229711401	1.197723	6.036211	1.58E-09	1.69E-08	up-regulated	protein_coding
CEP68	ENSMMUG00000017019	72.59354563	2.736623584	0.453453	6.03508	1.59E-09	1.70E-08	up-regulated	protein_coding
PROCA1	ENSMMUG00000004710	23.13927081	4.690798864	0.777395	6.033998	1.60E-09	1.71E-08	up-regulated	protein_coding
TAGLN	ENSMMUG00000002058	85.99980815	3.051674106	0.505815	6.033184	1.61E-09	1.71E-08	up-regulated	protein_coding
RAP1A	ENSMMUG00000014486	143.4322079	-4.677103768	0.77538	-6.03202	1.62E-09	1.73E-08	down-regulated	protein_coding
CYSTM1	ENSMMUG00000018574	154.6935567	-4.402620285	0.730011	-6.0309	1.63E-09	1.74E-08	down-regulated	protein_coding
EIF3L	ENSMMUG00000019785	250.632768	-3.191710866	0.529263	-6.03048	1.63E-09	1.74E-08	down-regulated	protein_coding
NFIC	ENSMMUG00000003439	43.86057365	3.270602876	0.542459	6.029214	1.65E-09	1.75E-08	up-regulated	protein_coding
KIF24	ENSMMUG00000018726	124.8189723	3.828253069	0.635348	6.02544	1.69E-09	1.79E-08	up-regulated	protein_coding
TRPM1	ENSMMUG00000009430	11.81298352	7.428473707	1.23323	6.02359	1.71E-09	1.81E-08	up-regulated	protein_coding
RPS15A	ENSMMUG00000005593	1052.931338	3.313865195	0.550584	6.018813	1.76E-09	1.86E-08	up-regulated	protein_coding
Y_RNA	ENSMMUG00000026825	13.17085088	7.040362484	1.169846	6.018193	1.76E-09	1.87E-08	up-regulated	misc_RNA
Y_RNA	ENSMMUG00000027747	11.25704575	7.978098355	1.326038	6.016494	1.78E-09	1.89E-08	up-regulated	misc_RNA
H2AFY	ENSMMUG00000014465	549.2448057	-1.856274676	0.308645	-6.01427	1.81E-09	1.91E-08	down-regulated	protein_coding
mml-mir-181a-2	ENSMMUG00000026876	12.91423763	8.21736846	1.366339	6.014151	1.81E-09	1.91E-08	up-regulated	miRNA
SNORA11	ENSMMUG00000033737	11.93550453	8.030800906	1.335317	6.014154	1.81E-09	1.91E-08	up-regulated	snoRNA
CCNG1	ENSMMUG00000019550	175.2976041	-4.903277945	0.815731	-6.0109	1.84E-09	1.95E-08	down-regulated	protein_coding
PRELID1	ENSMMUG00000009046	445.6027955	-2.58627191	0.430478	-6.00791	1.88E-09	1.98E-08	down-regulated	protein_coding
SDCBP	ENSMMUG00000017296	254.0825713	-2.339857252	0.389637	-6.00523	1.91E-09	2.01E-08	down-regulated	protein_coding
DNAH17	ENSMMUG00000008239	28.94128581	5.06527007	0.843791	6.002989	1.94E-09	2.04E-08	up-regulated	protein_coding
FAM162A	ENSMMUG00000016377	173.2255533	-3.743993918	0.623861	-6.00133	1.96E-09	2.06E-08	down-regulated	protein_coding
ATP6V0B	ENSMMUG00000015154	152.1125322	-4.153353165	0.6947	-5.97863	2.25E-09	2.36E-08	down-regulated	protein_coding
PLCE1	ENSMMUG00000001608	39.35084237	3.714658995	0.621373	5.978144	2.26E-09	2.36E-08	up-regulated	protein_coding

S100A10	ENSMMUG00000011656	788.9866224	-2.913293155	0.487517	-5.97578	2.29E-09	2.39E-08	down-regulated	protein_coding
XRCC3	ENSMMUG00000029181	231.3554392	3.303686005	0.552885	5.975354	2.30E-09	2.40E-08	up-regulated	protein_coding
SLC22A18	ENSMMUG00000007751	148.0369681	-3.63773377	0.609208	-5.97125	2.35E-09	2.46E-08	down-regulated	protein_coding
MGST1	ENSMMUG00000002359	329.941869	-3.576337969	0.599495	-5.96559	2.44E-09	2.54E-08	down-regulated	protein_coding
SF3B1	ENSMMUG00000022430	377.3021267	-1.931951232	0.32397	-5.96337	2.47E-09	2.57E-08	down-regulated	protein_coding
TFF3	ENSMMUG00000018076	187.6783802	-5.084595436	0.853026	-5.96066	2.51E-09	2.61E-08	down-regulated	protein_coding
OVCH2	ENSMMUG00000019757	12.84055805	8.197366341	1.375249	5.96064	2.51E-09	2.61E-08	up-regulated	protein_coding
DSTN	ENSMMUG00000013786	447.5207147	-1.928719378	0.323771	-5.95704	2.57E-09	2.67E-08	down-regulated	protein_coding
TFG	ENSMMUG00000002863	114.2492836	-7.204970126	1.209998	-5.95453	2.61E-09	2.71E-08	down-regulated	protein_coding
CYB5D1	ENSMMUG00000008179	23.47947334	3.185904041	0.535371	5.95083	2.67E-09	2.76E-08	up-regulated	protein_coding
ATP6V1B1	ENSMMUG00000002090	16.67074836	8.017607286	1.347615	5.949481	2.69E-09	2.78E-08	up-regulated	protein_coding
LAPTM4B	ENSMMUG00000008926	149.8899051	-4.711863915	0.792211	-5.94774	2.72E-09	2.81E-08	down-regulated	protein_coding
ETFA	ENSMMUG00000011045	182.1641979	-3.419909422	0.575002	-5.94765	2.72E-09	2.81E-08	down-regulated	protein_coding
PFN1	ENSMMUG00000002772	893.9000539	-2.830714636	0.475993	-5.94697	2.73E-09	2.82E-08	down-regulated	protein_coding
ACY1	ENSMMUG00000003646	162.3164593	-4.464608387	0.750981	-5.94504	2.76E-09	2.85E-08	down-regulated	protein_coding
PAM	ENSMMUG00000007844	386.3514522	-4.284695858	0.720793	-5.94442	2.77E-09	2.86E-08	down-regulated	protein_coding
SLC4A1AP	ENSMMUG00000019748	97.79705597	1.787525146	0.300907	5.940458	2.84E-09	2.93E-08	up-regulated	protein_coding
SYPL1	ENSMMUG00000019593	127.5207642	-4.190836786	0.705759	-5.93806	2.88E-09	2.97E-08	down-regulated	protein_coding
mml-mir-762-2	ENSMMUG00000037167	136.9950867	3.65494801	0.615818	5.935109	2.94E-09	3.02E-08	up-regulated	miRNA
FAU	ENSMMUG00000018776	251.3765014	-4.199516205	0.708108	-5.93062	3.02E-09	3.10E-08	down-regulated	protein_coding
SCARNA15	ENSMMUG00000036951	14.72316039	6.897508868	1.163403	5.928737	3.05E-09	3.14E-08	up-regulated	snoRNA
SRP14	ENSMMUG00000016061	187.8954531	-4.195282231	0.707876	-5.92658	3.09E-09	3.17E-08	down-regulated	protein_coding
CLDN20	ENSMMUG00000011172	28.27297094	5.71996361	0.966752	5.916682	3.29E-09	3.37E-08	up-regulated	protein_coding
TACC2	ENSMMUG00000013033	349.3277017	1.588819589	0.268708	5.91281	3.36E-09	3.45E-08	up-regulated	protein_coding
PLCD1	ENSMMUG00000000985	130.3358367	2.463907357	0.416795	5.91155	3.39E-09	3.46E-08	up-regulated	protein_coding
CCL20	ENSMMUG00000013250	135.0912068	-4.611878099	0.780133	-5.91165	3.39E-09	3.46E-08	down-regulated	protein_coding
HIVEP3	ENSMMUG00000016215	10.8260091	5.194465033	0.878666	5.911765	3.38E-09	3.46E-08	up-regulated	protein_coding
TTC21A	ENSMMUG00000020145	48.5632073	2.852019574	0.482538	5.91045	3.41E-09	3.49E-08	up-regulated	protein_coding
CAPZA2	ENSMMUG00000006784	248.0757496	-3.056240829	0.51746	-5.90623	3.50E-09	3.57E-08	down-regulated	protein_coding
Y_RNA	ENSMMUG00000025480	9.559816309	6.757546216	1.144803	5.902805	3.57E-09	3.65E-08	up-regulated	misc_RNA
DNAH7	ENSMMUG00000005622	17.31281064	7.931577434	1.343961	5.90164	3.60E-09	3.67E-08	up-regulated	protein_coding
RAVER1	ENSMMUG00000014897	136.8252536	-3.270031124	0.554338	-5.89898	3.66E-09	3.72E-08	down-regulated	protein_coding
DHX9	ENSMMUG00000005635	142.4101698	-3.470346381	0.588476	-5.89718	3.70E-09	3.76E-08	down-regulated	protein_coding
ABI2	ENSMMUG00000000312	54.45806733	3.135728033	0.531859	5.895784	3.73E-09	3.79E-08	up-regulated	protein_coding
C9orf129	ENSMMUG00000031598	128.0581775	3.89903344	0.661585	5.893474	3.78E-09	3.84E-08	up-regulated	protein_coding
CLMP	ENSMMUG00000007798	14.83897653	6.431382048	1.091632	5.891529	3.83E-09	3.88E-08	up-regulated	protein_coding
CLDN22	ENSMMUG00000030008	19.53654283	3.989665591	0.677368	5.889955	3.86E-09	3.91E-08	up-regulated	protein_coding
snoU13	ENSMMUG00000036143	18.22090938	7.736289654	1.313469	5.889967	3.86E-09	3.91E-08	up-regulated	snoRNA
SUFU	ENSMMUG00000004396	115.6663192	1.734045924	0.294603	5.886044	3.96E-09	4.00E-08	up-regulated	protein_coding
ATP5D	ENSMMUG00000007091	382.6608655	-4.069178772	0.691333	-5.88599	3.96E-09	4.00E-08	down-regulated	protein_coding
FAM104A	ENSMMUG00000001268	207.0123104	1.994426183	0.339177	5.880189	4.10E-09	4.14E-08	up-regulated	protein_coding
PRSS2	ENSMMUG00000000272	467.0260189	4.408513327	0.750063	5.877524	4.16E-09	4.20E-08	up-regulated	protein_coding
ITGB1	ENSMMUG00000017767	242.7278136	-3.989245674	0.678938	-5.87571	4.21E-09	4.24E-08	down-regulated	protein_coding
EPS8L2	ENSMMUG00000017483	439.1404013	-1.739970883	0.296239	-5.87354	4.27E-09	4.29E-08	down-regulated	protein_coding
HIST2H2BF	ENSMMUG00000031747	58.49856774	4.717296461	0.803137	5.87359	4.26E-09	4.29E-08	up-regulated	protein_coding
COL4A2	ENSMMUG00000022282	58.16348293	3.791306347	0.646156	5.867481	4.42E-09	4.45E-08	up-regulated	protein_coding
CRYGC	ENSMMUG00000011728	11.09217525	6.899325465	1.176496	5.864298	4.51E-09	4.53E-08	up-regulated	protein_coding
TEX10	ENSMMUG00000001526	85.88192644	2.407557501	0.410554	5.864174	4.51E-09	4.53E-08	up-regulated	protein_coding
ADAM17	ENSMMUG00000012921	86.3205406	2.040169624	0.347934	5.86367	4.53E-09	4.54E-08	up-regulated	protein_coding
GLOD4	ENSMMUG00000011417	281.5879923	2.21350378	0.377614	5.861818	4.58E-09	4.59E-08	up-regulated	protein_coding
CLIC1	ENSMMUG00000029827	307.1264929	-3.31890466	0.566342	-5.86025	4.62E-09	4.63E-08	down-regulated	protein_coding
U1	ENSMMUG00000026128	16.05095275	6.248688514	1.066401	5.859607	4.64E-09	4.64E-08	up-regulated	snRNA
MSH5	ENSMMUG00000009702	48.59184059	3.159186625	0.539212	5.858892	4.66E-09	4.66E-08	up-regulated	protein_coding
E2F5	ENSMMUG00000006380	63.34323742	2.931188511	0.500399	5.857705	4.69E-09	4.69E-08	up-regulated	protein_coding
TMPRSS7	ENSMMUG00000016068	10.3664116	6.28660474	1.073227	5.857663	4.69E-09	4.69E-08	up-regulated	protein_coding
BRICD5	ENSMMUG00000019233	78.00725943	4.045191838	0.690835	5.855511	4.76E-09	4.75E-08	up-regulated	protein_coding
ND4	ENSMMUG00000028677	24282.47447	4.199767346	0.717688	5.851798	4.86E-09	4.85E-08	up-regulated	protein_coding
AK8	ENSMMUG00000009916	30.8476709	3.574137196	0.611046	5.849212	4.94E-09	4.92E-08	up-regulated	protein_coding
TMEM54	ENSMMUG00000017301	270.7525181	-6.634857857	1.134607	-5.84772	4.98E-09	4.96E-08	down-regulated	protein_coding
RARB	ENSMMUG00000007367	19.03809283	6.297061145	1.0778	5.842511	5.14E-09	5.12E-08	up-regulated	protein_coding
FHL2	ENSMMUG00000016594	249.7944209	-2.183798432	0.374517	-5.83097	5.51E-09	5.48E-08	down-regulated	protein_coding
WSB1	ENSMMUG00000020226	194.1541994	-5.069844688	0.869753	-5.82906	5.57E-09	5.54E-08	down-regulated	protein_coding
POU1F1	ENSMMUG00000016244	21.97745241	5.293422222	0.908446	5.826899	5.65E-09	5.60E-08	up-regulated	protein_coding
POMGNT1	ENSMMUG00000015078	371.2491788	1.855914551	0.318693	5.823511	5.76E-09	5.71E-08	up-regulated	protein_coding
HIST1H2AC	ENSMMUG00000029916	78.12812208	7.323077685	1.257826	5.822014	5.81E-09	5.76E-08	up-regulated	protein_coding
EIF3E	ENSMMUG00000011359	192.4480034	-4.306416301	0.739765	-5.82133	5.84E-09	5.77E-08	down-regulated	protein_coding
CTSC	ENSMMUG00000022139	142.1446873	-7.520575236	1.292561	-5.81835	5.94E-09	5.86E-08	down-regulated	protein_coding
SAMHD1	ENSMMUG00000002091	118.5817112	2.83123544	0.486847	5.815456	6.05E-09	5.95E-08	up-regulated	protein_coding
MFAP3L	ENSMMUG00000019472	19.98521698	5.486964904	0.943507	5.815499	6.05E-09	5.95E-08	up-regulated	protein_coding
HSPA4	ENSMMUG00000009396	130.8736415	-4.242409522	0.729648	-5.81433	6.09E-09	5.98E-08	down-regulated	protein_coding
GDI1	ENSMMUG00000005502	167.1541793	-2.553661351	0.439225	-5.81401	6.10E-09	5.99E-08	down-regulated	protein_coding
TSPO	ENSMMUG00000006058	251.150551	-3.968023437	0.682881	-5.81071	6.22E-09	6.10E-08	down-regulated	protein_coding
FKBP8	ENSMMUG00000009587	321.4853418	-2.700697213	0.465123	-5.80642	6.38E-09	6.26E-08	down-regulated	protein_coding
PABPN1	ENSMMUG00000014747	203.3512602	-4.078411825	0.70243	-5.80614	6.39E-09	6.26E-08	down-regulated	protein_coding
ALDH6A1	ENSMMUG00000012684	106.9624477	2.47481827	0.426301	5.805328	6.42E-09	6.29E-08	up-regulated	protein_coding
AK2	ENSMMUG00000006284	196.5584908	-2.479606016	0.427249	-5.80366	6.49E-09	6.35E-08	down-regulated	protein_coding
DAD1	ENSMMUG00000000830	175.8720313	-4.023460169	0.693294	-5.8034	6.50E-09	6.35E-08	down-regulated	protein_coding
ATP6V1G1	ENSMMUG00000004899	133.4874066	-3.898099258	0.672516	-5.7963	6.78E-09	6.62E-08	down-regulated	protein_coding
Y_RNA	ENSMMUG00000027531	20.74617325	8.884261004	1.534218	5.790744	7.01E-09	6.84E-08	up-regulated	misc_RNA
MUC6	ENSMMUG00000012779	19.12330155	5.750421552	0.993134	5.790176	7.03E-09	6.86E-08	up-regulated	protein_coding
ADGRG2	ENSMMUG00000020515	19.16305405	5.803870851	1.002462	5.789615	7.05E-09	6.87E-08	up-regulated	protein_coding
FHOD3	ENSMMUG00000006862	16.35069074	6.278371558	1.084596	5.788671	7.09E-09	6.91E-08	up-regulated	protein_coding
METTL3	ENSMMUG00000014997	151.4918295	2.45399776	0.42428	5.783906	7.30E-09	7.10E-08	up-regulated	protein_coding
XPO1	ENSMMUG00000006941	152.7048292	-2.704351175	0.467692	-5.78234	7.37E-09	7.15E-08	down-regulated	protein_coding
CRYBG3	ENSMMUG00000017871	71.52147982	3.322408652	0.574719	5.780929	7.43E-09	7.21E-08	up-regulated	protein_coding
PKMYT1	ENSMMUG00000004214	312.3180738	3.307626828	0.572245	5.780093	7.47E-09	7.24E-08	up-regulated	protein_coding
MMP21	ENSMMUG00000008537	22.20937851	3.847253284	0.665641	5.779772	7.48E-09	7.25E-08	up-regulated	protein_coding
5S_rRNA	ENSMMUG00000034402	23.27672198	6.440262737	1.114355	5.779361	7.50E-09	7.26E-08	up-regulated	rRNA
C19orf26	ENSMMUG00000015768	21.35613242	4.005392821	0.693472	5.775854	7.66E-09	7.41E-08	up-regulated	protein_coding
Y_RNA	ENSMMUG00000026446	13.80153554	9.012372385	1.561556	5.771406	7.86E-09	7.60E-08	up-regulated	misc_RNA
EIF2B1	ENSMMUG00000013181	132.6395975	1.901946898	0.329754	5.767779	8.03E-09	7.76E-08	up-regulated	protein_coding
WDR72	ENSMMUG00000001242	13.01042864	9.642946292	1.673152	5.763341	8.25E-09	7.96E-08	up-regulated	protein_coding
FRMD7	ENSMMUG00000003479	15.58766648	8.441260482	1.464666	5.763265	8.25E-09	7.96E-08	up-regulated	protein_coding
SSR2	ENSMMUG00000013495	211.7310412	-3.538243446	0.614636	-5.75665	8.58E-09	8.27E-08	down-regulated	protein_coding
MXRA8	ENSMMUG00000003582	14.31361459	5.179980116	0.900088	5.754969	8.67E-09	8.35E-08	up-regulated	protein_coding
SCN1A	ENSMMUG00000008407	9.190853932	7.665861236	1.333746	5.747615	9.05E-09	8.71E-08	up-regulated	protein_coding
BLNK	ENSMMUG00000008687	83.09207773	2.279857068	0.396679	5.747357	9.06E-09	8.71E-08	up-regulated	protein_coding
CDX2	ENSMMUG00000016137	505.8906556	-1.911212094	0.332822	-5.74244	9.33E-09	8.96E-08	down-regulated	protein_coding
FOXP3	ENSMMUG00000008624	16.35196237	4.74332897	0.82634	5.740165	9.46E-09	9.08E-08	up-regulated	protein_coding
MAGED2	ENSMMUG00000007345	149.197867	-3.047371952	0.530922	-5.73977	9.48E-09	9.09E-08	down-regulated	protein_coding

SNORA64	ENSMMUG00000032817	16.14775709	7.926874262	1.381034	5.739811	9.48E-09	9.09E-08	up-regulated	snoRNA
LRCOL1	ENSMMUG00000031455	31.57028802	5.957676334	1.038988	5.734115	9.80E-09	9.39E-08	up-regulated	protein_coding
HNF1A	ENSMMUG00000006435	90.99618793	2.736956264	0.477474	5.732157	9.92E-09	9.49E-08	up-regulated	protein_coding
QRFP	ENSMMUG00000012502	19.66682575	7.307005046	1.275003	5.730969	9.99E-09	9.55E-08	up-regulated	protein_coding
PKN1	ENSMMUG00000005727	543.7749779	2.455450313	0.428492	5.730448	1.00E-08	9.58E-08	up-regulated	protein_coding
ABHD12B	ENSMMUG00000014667	15.45236873	6.061520264	1.058171	5.7283	1.01E-08	9.68E-08	up-regulated	protein_coding
ZC3HAV1L	ENSMMUG00000006549	137.4561559	3.716018171	0.649048	5.725338	1.03E-08	9.85E-08	up-regulated	protein_coding
FUT11	ENSMMUG00000013695	128.7246078	2.542026267	0.444232	5.722295	1.05E-08	1.00E-07	up-regulated	protein_coding
MPDU1	ENSMMUG00000013503	132.9060849	-3.971269383	0.69403	-5.72204	1.05E-08	1.00E-07	down-regulated	protein_coding
GPRIN1	ENSMMUG00000013284	22.55330229	6.573650111	1.149964	5.716399	1.09E-08	1.03E-07	up-regulated	protein_coding
RHAG	ENSMMUG00000012079	18.47173962	6.082903309	1.064767	5.712898	1.11E-08	1.05E-07	up-regulated	protein_coding
Y_RNA	ENSMMUG00000026029	11.56764164	6.02366856	1.055896	5.704796	1.16E-08	1.11E-07	up-regulated	misc_RNA
CYR61	ENSMMUG00000013197	47.47977755	2.169951196	0.380541	5.702273	1.18E-08	1.12E-07	up-regulated	protein_coding
NFIA	ENSMMUG00000013840	103.5197787	2.565414762	0.450104	5.699606	1.20E-08	1.14E-07	up-regulated	protein_coding
HIST1H3A	ENSMMUG00000001441	33.17607808	6.976282307	1.224107	5.699078	1.20E-08	1.14E-07	up-regulated	protein_coding
GCSAM	ENSMMUG00000016073	12.24922603	9.556384097	1.676825	5.699095	1.20E-08	1.14E-07	up-regulated	protein_coding
PDE6A	ENSMMUG00000004752	17.64091564	7.359285145	1.293481	5.689518	1.27E-08	1.20E-07	up-regulated	protein_coding
SNORA72	ENSMMUG00000024707	13.03079851	8.061874679	1.418809	5.682141	1.33E-08	1.26E-07	up-regulated	snoRNA
GPAT2	ENSMMUG00000012863	16.02218112	4.386863746	0.772181	5.68113	1.34E-08	1.26E-07	up-regulated	protein_coding
MAMSTR	ENSMMUG00000018414	10.42426614	4.948026657	0.87121	5.67949	1.35E-08	1.27E-07	up-regulated	protein_coding
FAM13C	ENSMMUG00000003706	22.28209395	6.115475421	1.077315	5.676591	1.37E-08	1.30E-07	up-regulated	protein_coding
SCPEP1	ENSMMUG00000002269	168.7427867	-3.139890732	0.553411	-5.67371	1.40E-08	1.31E-07	down-regulated	protein_coding
ACOT13	ENSMMUG00000029939	100.2640856	2.127548891	0.375328	5.6685	1.44E-08	1.35E-07	up-regulated	protein_coding
PCNP	ENSMMUG00000017782	127.2969566	-3.461648393	0.610698	-5.66835	1.44E-08	1.35E-07	down-regulated	protein_coding
FBRS	ENSMMUG00000005625	116.4871024	-3.132150379	0.552852	-5.66544	1.47E-08	1.37E-07	down-regulated	protein_coding
TES	ENSMMUG00000007097	232.9759848	-1.934141431	0.341398	-5.66535	1.47E-08	1.37E-07	down-regulated	protein_coding
MAT2A	ENSMMUG00000004450	123.5226044	-3.656868527	0.645557	-5.66468	1.47E-08	1.38E-07	down-regulated	protein_coding
IDI1	ENSMMUG00000017115	142.1364494	-2.651063197	0.46828	-5.66128	1.50E-08	1.41E-07	down-regulated	protein_coding
HVCN1	ENSMMUG00000012840	33.81322223	5.465466773	0.965721	5.659465	1.52E-08	1.42E-07	up-regulated	protein_coding
AKR1C3	ENSMMUG00000023280	174.6626239	-4.366224811	0.771882	-5.6566	1.54E-08	1.44E-07	down-regulated	protein_coding
LASP1	ENSMMUG00000007799	985.5044572	-1.486192984	0.262761	-5.65607	1.55E-08	1.45E-07	down-regulated	protein_coding
FAM189B	ENSMMUG00000018309	27.49737287	4.324004325	0.764551	5.655614	1.55E-08	1.45E-07	up-regulated	protein_coding
Y_RNA	ENSMMUG00000025317	13.11819132	5.460102079	0.96555	5.654913	1.56E-08	1.45E-07	up-regulated	misc_RNA
YKT6	ENSMMUG00000002430	169.5379333	-2.634158754	0.465859	-5.65442	1.56E-08	1.46E-07	down-regulated	protein_coding
Y_RNA	ENSMMUG00000024931	9.460468609	6.180857193	1.093152	5.65416	1.57E-08	1.46E-07	up-regulated	misc_RNA
SMC3	ENSMMUG00000007982	108.2244559	-3.441854526	0.609219	-5.64962	1.61E-08	1.49E-07	down-regulated	protein_coding
THAP9	ENSMMUG00000008510	47.8281818	2.782708861	0.492696	5.647919	1.62E-08	1.51E-07	up-regulated	protein_coding
RPS12	ENSMMUG00000013237	637.1088854	-3.851777954	0.682015	-5.64765	1.63E-08	1.51E-07	down-regulated	protein_coding
BCL2L1	ENSMMUG00000005985	659.0095934	1.947012034	0.345079	5.642229	1.68E-08	1.56E-07	up-regulated	protein_coding
ARHGEF4	ENSMMUG00000011314	76.0338965	6.312844354	1.119719	5.63788	1.72E-08	1.60E-07	up-regulated	protein_coding
MT2A	ENSMMUG00000022243	335.575366	4.065368538	0.721237	5.636659	1.73E-08	1.61E-07	up-regulated	protein_coding
MYH11	ENSMMUG00000010367	58.80963756	4.576514491	0.812244	5.634406	1.76E-08	1.63E-07	up-regulated	protein_coding
SPHK2	ENSMMUG00000022159	489.4129976	2.650315642	0.470548	5.632403	1.78E-08	1.64E-07	up-regulated	protein_coding
CCDC37	ENSMMUG00000004192	21.61399878	5.955516089	1.057461	5.631903	1.78E-08	1.65E-07	up-regulated	protein_coding
RPS7	ENSMMUG00000011870	560.7392753	-3.552385593	0.630757	-5.63194	1.78E-08	1.65E-07	down-regulated	protein_coding
TRAF4	ENSMMUG00000021407	288.8756668	-2.719575664	0.483056	-5.62994	1.80E-08	1.66E-07	down-regulated	protein_coding
CORO1B	ENSMMUG00000005488	185.3290438	-2.898789856	0.514961	-5.62915	1.81E-08	1.67E-07	down-regulated	protein_coding
RXFP4	ENSMMUG00000005090	76.58201583	3.805849058	0.676692	5.624199	1.86E-08	1.72E-07	up-regulated	protein_coding
CYP20A1	ENSMMUG00000005971	47.21697865	2.960035969	0.526397	5.623196	1.87E-08	1.73E-07	up-regulated	protein_coding
Y_RNA	ENSMMUG00000025629	11.36377152	8.738478277	1.554174	5.622586	1.88E-08	1.73E-07	up-regulated	misc_RNA
PIK3IP1	ENSMMUG00000019661	112.2321169	2.508173532	0.446209	5.621069	1.90E-08	1.75E-07	up-regulated	protein_coding
SLC26A8	ENSMMUG00000008442	28.43672917	5.835030363	1.038114	5.620799	1.90E-08	1.75E-07	up-regulated	protein_coding
NME1-NME2	ENSMMUG00000001940	324.7862267	-4.550613162	0.810132	-5.61712	1.94E-08	1.78E-07	down-regulated	protein_coding
MAPT	ENSMMUG00000004122	13.08132382	5.879080597	1.04684	5.616028	1.95E-08	1.79E-07	up-regulated	protein_coding
GIF	ENSMMUG00000013523	12.09756989	9.539660211	1.698776	5.615607	1.96E-08	1.80E-07	up-regulated	protein_coding
BLCAP	ENSMMUG00000015940	115.2210582	-3.749692545	0.667724	-5.61563	1.96E-08	1.80E-07	down-regulated	protein_coding
ALDOC	ENSMMUG00000020322	444.9698419	-3.187160053	0.567909	-5.61209	2.00E-08	1.83E-07	down-regulated	protein_coding
SERBP1	ENSMMUG00000000652	303.4416439	-2.36025523	0.420763	-5.60946	2.03E-08	1.86E-07	down-regulated	protein_coding
PSMA1	ENSMMUG00000016446	131.883903	-3.989545289	0.711286	-5.60892	2.04E-08	1.86E-07	down-regulated	protein_coding
ZNF789	ENSMMUG00000020139	59.26352087	2.913541374	0.519617	5.607092	2.06E-08	1.88E-07	up-regulated	protein_coding
ANO3	ENSMMUG00000012924	21.98028926	9.590745368	1.710927	5.605585	2.08E-08	1.90E-07	up-regulated	protein_coding
SLC14A2	ENSMMUG00000004010	17.14423631	9.303176347	1.659843	5.604852	2.08E-08	1.90E-07	up-regulated	protein_coding
DUSP5	ENSMMUG00000009858	246.7562067	-3.010132803	0.537087	-5.60456	2.09E-08	1.90E-07	down-regulated	protein_coding
TAS1R3	ENSMMUG00000032291	17.1057858	5.109452898	0.911651	5.604614	2.09E-08	1.90E-07	up-regulated	protein_coding
SNORD49	ENSMMUG00000025672	25.48591086	4.226145476	0.755081	5.596945	2.18E-08	1.99E-07	up-regulated	snoRNA
GPX4	ENSMMUG00000028946	537.0050273	-2.771367428	0.495175	-5.59674	2.18E-08	1.99E-07	down-regulated	protein_coding
IFI27L2	ENSMMUG00000023782	117.5980027	-7.246529755	1.295105	-5.59532	2.20E-08	2.00E-07	down-regulated	protein_coding
U6	ENSMMUG00000033135	13.45998434	7.104568051	1.270881	5.590268	2.27E-08	2.06E-07	up-regulated	snRNA
DDX31	ENSMMUG00000004590	29.69558753	3.362976041	0.601854	5.587697	2.30E-08	2.09E-07	up-regulated	protein_coding
CALM1	ENSMMUG00000005928	86.51035039	-6.803950362	1.218849	-5.58228	2.37E-08	2.15E-07	down-regulated	protein_coding
UGDH	ENSMMUG00000001892	223.7837538	-2.47789159	0.444033	-5.58042	2.40E-08	2.17E-07	down-regulated	protein_coding
KLB	ENSMMUG00000008834	17.4217564	4.814023955	0.863275	5.576463	2.45E-08	2.22E-07	up-regulated	protein_coding
ELSPBP1	ENSMMUG00000006857	14.09541256	6.305032319	1.131098	5.574258	2.49E-08	2.25E-07	up-regulated	protein_coding
ANXA9	ENSMMUG00000023231	40.83206467	3.118875881	0.559582	5.573586	2.50E-08	2.25E-07	up-regulated	protein_coding
SYCE2	ENSMMUG00000020225	33.01952496	4.636241425	0.832037	5.572158	2.52E-08	2.27E-07	up-regulated	protein_coding
TXN	ENSMMUG00000010322	540.4817331	-3.81695242	0.685162	-5.57087	2.53E-08	2.29E-07	down-regulated	protein_coding
FBN1	ENSMMUG00000004239	19.49054383	5.037556803	0.904594	5.568859	2.56E-08	2.31E-07	up-regulated	protein_coding
RPAIN	ENSMMUG00000010468	179.8970908	3.064551882	0.550508	5.56677	2.60E-08	2.34E-07	up-regulated	protein_coding
RBP2	ENSMMUG00000003054	512.1143479	-3.038441239	0.545876	-5.56617	2.60E-08	2.34E-07	down-regulated	protein_coding
ARF3	ENSMMUG00000006968	147.8339067	-2.478401396	0.445345	-5.56512	2.62E-08	2.35E-07	down-regulated	protein_coding
CCDC7	ENSMMUG00000017765	18.54189478	6.640498118	1.193605	5.563398	2.65E-08	2.38E-07	up-regulated	protein_coding
DNAH5	ENSMMUG00000011026	17.12271803	8.511829856	1.530598	5.561113	2.68E-08	2.41E-07	up-regulated	protein_coding
RPL3	ENSMMUG00000010029	1758.012532	-3.613049379	0.649753	-5.56065	2.69E-08	2.41E-07	down-regulated	protein_coding
PIM1	ENSMMUG00000017316	188.4131629	-2.735226632	0.492019	-5.55919	2.71E-08	2.43E-07	down-regulated	protein_coding
ATP6V1E1	ENSMMUG00000021477	111.7188419	-3.652423479	0.6572	-5.55755	2.74E-08	2.45E-07	down-regulated	protein_coding
KAZ	ENSMMUG00000023070	33.08390071	2.827284454	0.509211	5.552282	2.82E-08	2.52E-07	up-regulated	protein_coding
ANXA6	ENSMMUG00000021053	210.1398953	-3.322031348	0.59863	-5.54939	2.87E-08	2.56E-07	down-regulated	protein_coding
NCSTN	ENSMMUG00000021856	225.7566764	-1.857888984	0.335009	-5.54578	2.93E-08	2.61E-07	down-regulated	protein_coding
TMEM14A	ENSMMUG00000023710	107.7065459	-3.671566739	0.66205	-5.54576	2.93E-08	2.61E-07	down-regulated	protein_coding
CST3	ENSMMUG00000009200	420.3647033	-2.351990577	0.424154	-5.54513	2.94E-08	2.62E-07	down-regulated	protein_coding
NDUFA4	ENSMMUG00000020894	112.2470988	-7.17909212	1.295039	-5.54353	2.96E-08	2.64E-07	down-regulated	protein_coding
LIPT2	ENSMMUG00000013094	94.15040572	2.891886692	0.521803	5.5421	2.99E-08	2.66E-07	up-regulated	protein_coding
BCORL1	ENSMMUG00000008781	101.919818	2.504131808	0.451937	5.540893	3.01E-08	2.68E-07	up-regulated	protein_coding
C17orf99	ENSMMUG00000021863	28.03119617	6.107973216	1.102342	5.540904	3.01E-08	2.68E-07	up-regulated	protein_coding
ENOSF1	ENSMMUG00000004051	121.8011817	2.417778696	0.436382	5.540509	3.02E-08	2.68E-07	up-regulated	protein_coding
BTF3	ENSMMUG00000022291	224.9504536	-3.810904346	0.687894	-5.53996	3.03E-08	2.69E-07	down-regulated	protein_coding
GSN	ENSMMUG00000013020	1067.837256	-2.60387319	0.470206	-5.53773	3.06E-08	2.72E-07	down-regulated	protein_coding
YWHAB	ENSMMUG00000013206	380.5308798	-2.042888863	0.368979	-5.5366	3.08E-08	2.74E-07	down-regulated	protein_coding
SEC62	ENSMMUG00000021899	130.2045008	-2.652361026	0.479327	-5.53351	3.14E-08	2.78E-07	down-regulated	protein_coding
SEC16A	ENSMMUG00000003106	472.9327579	2.006651636	0.362683	5.532802	3.15E-08	2.79E-07	up-regulated	protein_coding

U6	ENSMMUG00000027332	18.81742104	8.08763949	1.462429	5.530277	3.20E-08	2.83E-07	up-regulated	snRNA
PRIC285	ENSMMUG00000006070	175.2986109	-2.143073238	0.387846	-5.52558	3.28E-08	2.91E-07	down-regulated	protein_coding
B3GNT2	ENSMMUG00000014181	161.1013007	-3.491482875	0.631881	-5.52554	3.28E-08	2.91E-07	down-regulated	protein_coding
NPC2	ENSMMUG00000006563	159.3621298	-6.709355608	1.214417	-5.52476	3.30E-08	2.92E-07	down-regulated	protein_coding
DIMT1	ENSMMUG00000000400	111.7776649	2.602041338	0.471085	5.52351	3.32E-08	2.93E-07	up-regulated	protein_coding
IRF2	ENSMMUG00000015087	270.6074705	2.159240181	0.39102	5.522071	3.35E-08	2.96E-07	up-regulated	protein_coding
AUNIP	ENSMMUG00000007856	25.57899746	4.140475379	0.750286	5.518531	3.42E-08	3.02E-07	up-regulated	protein_coding
ABHD16B	ENSMMUG00000011854	44.71906268	4.680172441	0.84812	5.51829	3.42E-08	3.02E-07	up-regulated	protein_coding
ARPC2	ENSMMUG00000022808	778.1284219	-1.34199342	0.243204	-5.51797	3.43E-08	3.02E-07	down-regulated	protein_coding
RNF114	ENSMMUG00000000757	113.1108904	-3.140181776	0.569343	-5.51544	3.48E-08	3.06E-07	down-regulated	protein_coding
ATP5A1	ENSMMUG00000003570	978.0162644	-1.695053226	0.3074	-5.51416	3.50E-08	3.08E-07	down-regulated	protein_coding
PFKL	ENSMMUG00000004173	840.1706601	-2.268210861	0.411653	-5.51001	3.59E-08	3.15E-07	down-regulated	protein_coding
PDE7B	ENSMMUG00000003708	9.950982296	7.80304105	1.416843	5.507345	3.64E-08	3.20E-07	up-regulated	protein_coding
LIMCH1	ENSMMUG00000007805	14.07545925	6.975761684	1.266883	5.506241	3.67E-08	3.21E-07	up-regulated	protein_coding
RLTPR	ENSMMUG00000009799	46.38654717	4.00988734	0.728259	5.506131	3.67E-08	3.21E-07	up-regulated	protein_coding
ADH7	ENSMMUG00000011253	136.1682492	3.513596247	0.638214	5.505354	3.68E-08	3.23E-07	up-regulated	protein_coding
EDEM1	ENSMMUG00000008807	246.9324572	-3.304859416	0.60036	-5.5048	3.70E-08	3.23E-07	down-regulated	protein_coding
ASNS	ENSMMUG00000019157	109.948225	-3.093190573	0.561938	-5.5045	3.70E-08	3.24E-07	down-regulated	protein_coding
PAPOLA	ENSMMUG00000018610	144.4536335	-2.994507711	0.5441	-5.5036	3.72E-08	3.25E-07	down-regulated	protein_coding
ADAMTS14	ENSMMUG00000012330	17.35166984	5.434770221	0.987727	5.502301	3.75E-08	3.27E-07	up-regulated	protein_coding
CLIC4	ENSMMUG00000002416	124.068202	-3.374979868	0.613608	-5.50022	3.79E-08	3.31E-07	down-regulated	protein_coding
RPL7A	ENSMMUG00000022341	1604.477151	-2.679987578	0.487336	-5.49926	3.81E-08	3.32E-07	down-regulated	protein_coding
SLC25A3	ENSMMUG00000023036	818.1072449	-2.202430493	0.40058	-5.49811	3.84E-08	3.34E-07	down-regulated	protein_coding
ELOVL1	ENSMMUG00000003152	341.0460763	-2.005394488	0.364886	-5.49595	3.89E-08	3.38E-07	down-regulated	protein_coding
HNRPH1	ENSMMUG00000019580	115.0390237	-3.562514084	0.648348	-5.49475	3.91E-08	3.40E-07	down-regulated	protein_coding
SLC6A8	ENSMMUG00000012742	1463.537157	-3.309927526	0.60357	-5.48391	4.16E-08	3.62E-07	down-regulated	protein_coding
TRPC5	ENSMMUG00000010424	10.60256891	8.63646047	1.57597	5.480093	4.25E-08	3.69E-07	up-regulated	protein_coding
ATP6V0E1	ENSMMUG00000013825	111.2618586	-3.69170825	0.673935	-5.47784	4.31E-08	3.74E-07	down-regulated	protein_coding
RPL34	ENSMMUG00000011901	262.3167072	-4.092129042	0.747393	-5.4752	4.37E-08	3.79E-07	down-regulated	protein_coding
CXCL16	ENSMMUG00000004891	138.5950231	-3.040927112	0.555611	-5.47313	4.42E-08	3.83E-07	down-regulated	protein_coding
NDUFA10	ENSMMUG00000010382	133.1833885	-3.478651928	0.63584	-5.47095	4.48E-08	3.88E-07	down-regulated	protein_coding
TANC1	ENSMMUG00000006180	133.5355799	1.810813305	0.331023	5.470352	4.49E-08	3.89E-07	up-regulated	protein_coding
SUN1	ENSMMUG00000017056	325.67273	-3.022799704	0.552656	-5.46959	4.51E-08	3.90E-07	down-regulated	protein_coding
CRYL1	ENSMMUG00000004560	127.9845884	-2.580610221	0.471823	-5.46944	4.51E-08	3.90E-07	down-regulated	protein_coding
KIAA0895	ENSMMUG00000010703	117.3956364	4.98416789	0.912268	5.46349	4.67E-08	4.03E-07	up-regulated	protein_coding
TBCE	ENSMMUG00000008426	173.9793796	2.41028665	0.441216	5.462822	4.69E-08	4.04E-07	up-regulated	protein_coding
5S_rRNA	ENSMMUG00000034334	18.49100345	8.644103107	1.584677	5.454805	4.90E-08	4.22E-07	up-regulated	rRNA
Y_RNA	ENSMMUG00000025215	15.35053807	6.852690111	1.257529	5.449328	5.06E-08	4.35E-07	up-regulated	misc_RNA
CYB5A	ENSMMUG00000013607	174.1941305	-6.844523249	1.256101	-5.44902	5.06E-08	4.36E-07	down-regulated	protein_coding
ARL6IP5	ENSMMUG00000008627	113.0358622	-4.270085514	0.783972	-5.44673	5.13E-08	4.40E-07	down-regulated	protein_coding
SRP54	ENSMMUG00000015073	77.55166007	-6.645555305	1.2215	-5.44049	5.31E-08	4.56E-07	down-regulated	protein_coding
Y_RNA	ENSMMUG00000025754	11.18471114	9.427782865	1.735612	5.431965	5.57E-08	4.77E-07	up-regulated	misc_RNA
SQSTM1	ENSMMUG00000021055	454.4360895	-1.877108281	0.345888	-5.42692	5.73E-08	4.91E-07	down-regulated	protein_coding
C12orf42	ENSMMUG00000004953	10.57516467	9.34541366	1.72226	5.426251	5.76E-08	4.92E-07	up-regulated	protein_coding
HFE2	ENSMMUG00000016054	13.42178496	7.575888153	1.399923	5.411645	6.24E-08	5.34E-07	up-regulated	protein_coding
ALS2CR12	ENSMMUG00000018356	8.950050818	5.514433197	1.019234	5.410371	6.29E-08	5.37E-07	up-regulated	protein_coding
FAM70A	ENSMMUG00000011074	22.51754014	5.673329084	1.048716	5.409783	6.31E-08	5.39E-07	up-regulated	protein_coding
LAMC2	ENSMMUG00000001479	347.2107211	-2.940116761	0.543667	-5.40794	6.38E-08	5.44E-07	down-regulated	protein_coding
CCDC78	ENSMMUG00000006452	22.21468057	3.378576859	0.625242	5.403632	6.53E-08	5.57E-07	up-regulated	protein_coding
B3GAT2	ENSMMUG00000017557	22.1165575	3.83960223	0.710749	5.40219	6.58E-08	5.60E-07	up-regulated	protein_coding
RPS14	ENSMMUG00000002848	951.6430498	-3.189535781	0.59049	-5.40151	6.61E-08	5.62E-07	down-regulated	protein_coding
PRRT3	ENSMMUG00000018289	19.12324552	3.176785109	0.58855	5.397646	6.75E-08	5.73E-07	up-regulated	protein_coding
C15ORF63	ENSMMUG00000022768	516.6300602	-2.90414741	0.538064	-5.3974	6.76E-08	5.74E-07	down-regulated	protein_coding
PRSS54	ENSMMUG00000019451	15.96046962	8.462660282	1.568319	5.396007	6.81E-08	5.78E-07	up-regulated	protein_coding
MGAT4A	ENSMMUG00000022909	167.7762574	-4.911971176	0.910545	-5.39454	6.87E-08	5.82E-07	down-regulated	protein_coding
TIRAP	ENSMMUG00000007946	98.00021717	2.160445139	0.400666	5.392135	6.96E-08	5.90E-07	up-regulated	protein_coding
SCAMP2	ENSMMUG00000012974	134.1199618	-2.80501635	0.520232	-5.39186	6.97E-08	5.90E-07	down-regulated	protein_coding
U6	ENSMMUG00000036646	9.900339795	5.430750023	1.007947	5.387932	7.13E-08	6.03E-07	up-regulated	snRNA
SUCLG1	ENSMMUG00000000908	193.8255562	-2.621087397	0.486687	-5.38557	7.22E-08	6.10E-07	down-regulated	protein_coding
SCGB1C1	ENSMMUG00000011501	12.83917872	8.199304329	1.522768	5.384475	7.27E-08	6.14E-07	up-regulated	protein_coding
SULT1C2	ENSMMUG00000003696	13.06273588	5.302535915	0.985031	5.383118	7.32E-08	6.18E-07	up-regulated	protein_coding
CDK5R1	ENSMMUG00000009548	25.60972522	3.437874848	0.6387	5.382616	7.34E-08	6.19E-07	up-regulated	protein_coding
IMMP1L	ENSMMUG00000016806	71.03642269	2.555080156	0.474827	5.381081	7.40E-08	6.24E-07	up-regulated	protein_coding
LSR	ENSMMUG00000011294	313.7773176	-1.755795782	0.326463	-5.37823	7.52E-08	6.34E-07	down-regulated	protein_coding
SYDE1	ENSMMUG00000007726	10.56695564	6.634265947	1.233927	5.376548	7.59E-08	6.39E-07	up-regulated	protein_coding
SRPR	ENSMMUG00000007938	398.6363459	1.745548783	0.324671	5.376365	7.60E-08	6.40E-07	up-regulated	protein_coding
EPRS	ENSMMUG00000008722	165.8597233	-2.328201719	0.433175	-5.37473	7.67E-08	6.45E-07	down-regulated	protein_coding
CD37	ENSMMUG00000014789	80.29463023	4.20360069	0.782255	5.373695	7.71E-08	6.49E-07	up-regulated	protein_coding
LIPM	ENSMMUG00000000469	19.88835637	4.202053056	0.782649	5.369015	7.92E-08	6.65E-07	up-regulated	protein_coding
DERL1	ENSMMUG00000013516	144.6783038	-2.448264328	0.456104	-5.36777	7.97E-08	6.69E-07	down-regulated	protein_coding
Y_RNA	ENSMMUG00000026080	10.65034346	4.919404188	0.917015	5.364582	8.11E-08	6.81E-07	up-regulated	misc_RNA
FLOT1	ENSMMUG00000014071	163.9777238	-2.383177607	0.4443	-5.36389	8.14E-08	6.83E-07	down-regulated	protein_coding
DCXR	ENSMMUG00000010896	145.4126397	-5.143905195	0.959193	-5.36274	8.20E-08	6.87E-07	down-regulated	protein_coding
EIF5	ENSMMUG00000005630	111.1445671	-4.700027132	0.876705	-5.36101	8.28E-08	6.93E-07	down-regulated	protein_coding
WDR83	ENSMMUG00000016868	113.2667286	2.425241137	0.452469	5.360013	8.32E-08	6.96E-07	up-regulated	protein_coding
XKR8	ENSMMUG00000012964	61.41050762	2.970402754	0.554738	5.354602	8.57E-08	7.16E-07	up-regulated	protein_coding
RAB41	ENSMMUG00000013937	20.61394778	5.042418359	0.941791	5.354075	8.60E-08	7.18E-07	up-regulated	protein_coding
NTF4	ENSMMUG00000015794	16.23853564	7.606561219	1.420948	5.353159	8.64E-08	7.21E-07	up-regulated	protein_coding
OGN	ENSMMUG00000004634	13.78536406	6.905863139	1.290316	5.35207	8.70E-08	7.25E-07	up-regulated	protein_coding
LDB2	ENSMMUG00000023116	9.590747398	7.62479455	1.424839	5.351337	8.73E-08	7.28E-07	up-regulated	protein_coding
Y_RNA	ENSMMUG00000024638	9.407391009	6.693649122	1.251905	5.34677	8.95E-08	7.46E-07	up-regulated	misc_RNA
OR6J1	ENSMMUG00000009442	13.13310858	8.84809045	1.654884	5.346652	8.96E-08	7.46E-07	up-regulated	protein_coding
GPR143	ENSMMUG00000006192	10.65099274	9.352175809	1.750467	5.342675	9.16E-08	7.62E-07	up-regulated	protein_coding
HSPA1L	ENSMMUG00000029822	365.8299027	2.959357411	0.554169	5.340174	9.29E-08	7.72E-07	up-regulated	protein_coding
ATPIF1	ENSMMUG00000018678	217.4449416	-3.054652652	0.57231	-5.33741	9.43E-08	7.83E-07	down-regulated	protein_coding
FRAS1	ENSMMUG00000002564	25.47528192	5.02324954	0.941672	5.334394	9.59E-08	7.96E-07	up-regulated	protein_coding
PTCD1	ENSMMUG00000030408	346.3155599	2.691424	0.504776	5.331918	9.72E-08	8.06E-07	up-regulated	protein_coding
RPS6KA1	ENSMMUG00000022111	831.4998567	-1.663513369	0.31201	-5.3316	9.74E-08	8.06E-07	down-regulated	protein_coding
CCT8	ENSMMUG00000003023	233.3806021	-2.736743118	0.513635	-5.32818	9.92E-08	8.21E-07	down-regulated	protein_coding
OLA1	ENSMMUG00000007092	109.4968543	-3.699895338	0.694441	-5.32788	9.94E-08	8.22E-07	down-regulated	protein_coding
NDUFC2	ENSMMUG00000017471	126.0917465	-4.40230144	0.826605	-5.32576	1.01E-07	8.31E-07	down-regulated	protein_coding
LAP3	ENSMMUG00000005732	235.6331186	-2.561729273	0.481418	-5.32122	1.03E-07	8.51E-07	down-regulated	protein_coding
PPL	ENSMMUG00000017822	137.8888482	1.99040833	0.374044	5.321321	1.03E-07	8.51E-07	up-regulated	protein_coding
HIST1H3A	ENSMMUG00000023727	12.29832554	6.361919817	1.195914	5.319715	1.04E-07	8.58E-07	up-regulated	protein_coding
COX5B	ENSMMUG00000009730	162.3252208	-5.328170081	1.001683	-5.31922	1.04E-07	8.60E-07	down-regulated	protein_coding
MEA1	ENSMMUG00000004871	502.09084	2.356179629	0.443403	5.313859	1.07E-07	8.85E-07	up-regulated	protein_coding
CPNE9	ENSMMUG00000006115	8.756488467	7.090327835	1.334395	5.313516	1.08E-07	8.86E-07	up-regulated	protein_coding
RAB33B	ENSMMUG00000020692	110.8064386	2.765004351	0.5204	5.313226	1.08E-07	8.87E-07	up-regulated	protein_coding
PSMD12	ENSMMUG00000002132	100.4207446	-3.339781096	0.629032	-5.3094	1.10E-07	9.05E-07	down-regulated	protein_coding
PNPO	ENSMMUG00000003325	219.8254099	2.128481522	0.400965	5.308395	1.11E-07	9.10E-07	up-regulated	protein_coding

H2AFZ	ENSMMUG00000006834	195.6761185	-5.589427363	1.05321	-5.30704	1.11E-07	9.16E-07	down-regulated	protein_coding
M1AP	ENSMMUG00000002140	15.86034752	9.169525221	1.728198	5.305831	1.12E-07	9.22E-07	up-regulated	protein_coding
GJD3	ENSMMUG00000012489	91.53883083	7.367077051	1.388762	5.30478	1.13E-07	9.26E-07	up-regulated	protein_coding
RPS23	ENSMMUG00000003578	416.3126617	-3.734018657	0.704062	-5.30354	1.14E-07	9.32E-07	down-regulated	protein_coding
CD33	ENSMMUG00000009486	14.05092521	8.931979314	1.684194	5.303415	1.14E-07	9.32E-07	up-regulated	protein_coding
CACNA1E	ENSMMUG00000014498	14.35423749	8.962440504	1.690021	5.303155	1.14E-07	9.33E-07	up-regulated	protein_coding
SLC39A9	ENSMMUG00000021572	118.6199505	-4.385002096	0.8274	-5.29974	1.16E-07	9.50E-07	down-regulated	protein_coding
IQCK	ENSMMUG00000002708	80.07583659	4.137069645	0.780709	5.299121	1.16E-07	9.53E-07	up-regulated	protein_coding
NSUN5	ENSMMUG00000022776	359.2094233	2.527114903	0.477412	5.293368	1.20E-07	9.83E-07	up-regulated	protein_coding
CST6	ENSMMUG00000006095	121.2977436	2.615283483	0.494224	5.291699	1.21E-07	9.91E-07	up-regulated	protein_coding
AKTIP	ENSMMUG00000008657	143.3844266	1.487542869	0.281172	5.290509	1.22E-07	9.97E-07	up-regulated	protein_coding
RAD23B	ENSMMUG00000014944	274.9404675	-1.498839719	0.283329	-5.2901	1.22E-07	9.99E-07	down-regulated	protein_coding
UQCRB	ENSMMUG00000008804	120.9608318	-4.453945161	0.842192	-5.28852	1.23E-07	1.01E-06	down-regulated	protein_coding
LRRC14	ENSMMUG00000015593	146.0502641	2.432230108	0.459912	5.288465	1.23E-07	1.01E-06	up-regulated	protein_coding
HSD17B8	ENSMMUG00000003861	78.32010899	-6.659749709	1.259356	-5.28822	1.24E-07	1.01E-06	down-regulated	protein_coding
ACSM4	ENSMMUG00000022106	25.92261735	5.747522748	1.086991	5.287553	1.24E-07	1.01E-06	up-regulated	protein_coding
ZXDA	ENSMMUG00000019432	57.27029049	3.331086118	0.630055	5.286979	1.24E-07	1.01E-06	up-regulated	protein_coding
XPNPEP1	ENSMMUG00000015117	123.3795908	-2.473446884	0.468084	-5.2842	1.26E-07	1.03E-06	down-regulated	protein_coding
snoU13	ENSMMUG00000036634	15.99804442	5.705327487	1.079729	5.284038	1.26E-07	1.03E-06	up-regulated	snoRNA
PGRMC2	ENSMMUG00000014585	105.2118299	-2.990586914	0.566106	-5.28273	1.27E-07	1.03E-06	down-regulated	protein_coding
HIST1H3A	ENSMMUG00000023722	15.9868846	4.651546538	0.880511	5.28278	1.27E-07	1.03E-06	up-regulated	protein_coding
PLCB3	ENSMMUG00000012948	572.0092359	-1.482054244	0.280555	-5.28259	1.27E-07	1.03E-06	down-regulated	protein_coding
MYO16	ENSMMUG00000021231	11.78378855	6.137542419	1.162162	5.281141	1.28E-07	1.04E-06	up-regulated	protein_coding
SERINC1	ENSMMUG00000007911	182.1713276	-3.627767103	0.687303	-5.27826	1.30E-07	1.06E-06	down-regulated	protein_coding
PDGFRB	ENSMMUG00000011557	13.03104944	4.776607653	0.90658	5.268823	1.37E-07	1.11E-06	up-regulated	protein_coding
ZBTB12	ENSMMUG00000011683	47.59826335	2.945748275	0.55924	5.267416	1.38E-07	1.12E-06	up-regulated	protein_coding
CCDC38	ENSMMUG00000009303	12.17795302	6.128915398	1.163849	5.266075	1.39E-07	1.13E-06	up-regulated	protein_coding
LGALS13	ENSMMUG00000003527	32.2814806	7.183590208	1.364611	5.264202	1.41E-07	1.14E-06	up-regulated	protein_coding
ANXA1	ENSMMUG00000012667	113.24057	-4.780554156	0.909418	-5.25672	1.47E-07	1.18E-06	down-regulated	protein_coding
HADH	ENSMMUG00000023254	282.0614908	-2.563795617	0.487712	-5.25678	1.47E-07	1.18E-06	down-regulated	protein_coding
ALDOB	ENSMMUG00000023796	2509.463207	-3.270363888	0.62236	-5.25478	1.48E-07	1.20E-06	down-regulated	protein_coding
SH3D21	ENSMMUG00000011688	352.2550708	2.496656568	0.475174	5.254196	1.49E-07	1.20E-06	up-regulated	protein_coding
ERLIN1	ENSMMUG00000018470	133.2443715	-2.327918667	0.443507	-5.24889	1.53E-07	1.23E-06	down-regulated	protein_coding
AGPAT4	ENSMMUG00000006147	20.7980623	5.846790493	1.113992	5.248503	1.53E-07	1.23E-06	up-regulated	protein_coding
NSUN4	ENSMMUG00000002717	58.54083122	1.816057694	0.346043	5.248063	1.54E-07	1.24E-06	up-regulated	protein_coding
ETF1	ENSMMUG00000005871	156.5669374	-2.170566943	0.413655	-5.24729	1.54E-07	1.24E-06	down-regulated	protein_coding
BSG	ENSMMUG00000018740	288.8997778	-2.773172852	0.528507	-5.24718	1.54E-07	1.24E-06	down-regulated	protein_coding
HNRNPA3	ENSMMUG00000001858	211.9923776	-3.497416956	0.666582	-5.24679	1.55E-07	1.24E-06	down-regulated	protein_coding
SHISA5	ENSMMUG00000010062	129.8941371	-3.998071732	0.762092	-5.24618	1.55E-07	1.25E-06	down-regulated	protein_coding
TMEM14B	ENSMMUG00000018789	165.2465781	-6.768097646	1.291365	-5.24104	1.60E-07	1.28E-06	down-regulated	protein_coding
LRRC39	ENSMMUG00000004241	19.17394514	3.149542403	0.601053	5.240037	1.61E-07	1.29E-06	up-regulated	protein_coding
Y_RNA	ENSMMUG00000024867	9.738133987	9.226110256	1.760771	5.239812	1.61E-07	1.29E-06	up-regulated	misc_RNA
ALDH3A2	ENSMMUG00000014130	215.5837858	-4.337648262	0.828145	-5.23779	1.63E-07	1.30E-06	down-regulated	protein_coding
UQCR	ENSMMUG00000009016	180.8300215	-4.272212341	0.815822	-5.2367	1.63E-07	1.31E-06	down-regulated	protein_coding
HTATIP2	ENSMMUG00000010718	309.5107586	-2.506989993	0.478899	-5.23491	1.65E-07	1.32E-06	down-regulated	protein_coding
STK35	ENSMMUG00000004305	61.24235207	2.560921074	0.489301	5.23384	1.66E-07	1.33E-06	up-regulated	protein_coding
TM2D1	ENSMMUG00000010931	63.36621403	-6.355184682	1.214782	-5.23154	1.68E-07	1.34E-06	down-regulated	protein_coding
CD82	ENSMMUG00000019268	122.659192	-6.336206113	1.211891	-5.22836	1.71E-07	1.36E-06	down-regulated	protein_coding
PTPLAD2	ENSMMUG00000019068	11.59128098	5.929726494	1.134614	5.226207	1.73E-07	1.38E-06	up-regulated	protein_coding
NUDT21	ENSMMUG00000019906	72.97246516	-6.557739148	1.256021	-5.22104	1.78E-07	1.42E-06	down-regulated	protein_coding
MGAT1	ENSMMUG00000009137	90.87443292	-3.680758083	0.705231	-5.21922	1.80E-07	1.43E-06	down-regulated	protein_coding
COX7B	ENSMMUG00000015861	161.6276626	-6.735928672	1.290869	-5.21814	1.81E-07	1.44E-06	down-regulated	protein_coding
RAB18	ENSMMUG00000006928	115.0128686	-3.974830379	0.761776	-5.21785	1.81E-07	1.44E-06	down-regulated	protein_coding
SRRM1	ENSMMUG00000006645	347.3849618	1.426052592	0.27337	5.216562	1.82E-07	1.45E-06	up-regulated	protein_coding
ABCA4	ENSMMUG00000013858	215.7043442	2.416634869	0.46328	5.21636	1.82E-07	1.45E-06	up-regulated	protein_coding
SLC12A9	ENSMMUG00000021248	142.6908787	-2.845425432	0.54549	-5.21627	1.83E-07	1.45E-06	down-regulated	protein_coding
BPNT1	ENSMMUG00000017396	65.42319263	-6.400264542	1.227303	-5.2149	1.84E-07	1.46E-06	down-regulated	protein_coding
TIMP1	ENSMMUG00000011426	83.65364422	-6.755003474	1.295429	-5.21449	1.84E-07	1.46E-06	down-regulated	protein_coding
ACRV1	ENSMMUG00000014812	11.50939348	4.810836414	0.923139	5.211391	1.87E-07	1.48E-06	up-regulated	protein_coding
GSTCD	ENSMMUG00000018811	30.35782796	2.579464887	0.495012	5.210909	1.88E-07	1.49E-06	up-regulated	protein_coding
OCIAD1	ENSMMUG00000014868	98.61100191	-3.093719788	0.593784	-5.21018	1.89E-07	1.49E-06	down-regulated	protein_coding
SRR	ENSMMUG00000017518	243.0642073	2.480024434	0.476014	5.209979	1.89E-07	1.49E-06	up-regulated	protein_coding
PRSS53	ENSMMUG00000012862	57.46335008	3.414113531	0.655559	5.207942	1.91E-07	1.51E-06	up-regulated	protein_coding
RAB23	ENSMMUG00000018003	68.93835199	3.323145836	0.638786	5.20228	1.97E-07	1.55E-06	up-regulated	protein_coding
SNORA12	ENSMMUG00000033195	10.27185239	9.305648234	1.789355	5.200561	1.99E-07	1.57E-06	up-regulated	snoRNA
RPL15	ENSMMUG00000006353	452.1615414	-3.277960965	0.630398	-5.19983	1.99E-07	1.57E-06	down-regulated	protein_coding
5S_rRNA	ENSMMUG00000034828	11.90483108	8.732630472	1.6797	5.198924	2.00E-07	1.58E-06	up-regulated	rRNA
ST18	ENSMMUG00000008439	9.550248508	6.090368195	1.171558	5.198522	2.01E-07	1.58E-06	up-regulated	protein_coding
U6	ENSMMUG00000036752	9.733451804	7.682773774	1.478136	5.197609	2.02E-07	1.59E-06	up-regulated	snRNA
IL2RG	ENSMMUG00000017795	157.0160839	-4.564608777	0.878801	-5.19413	2.06E-07	1.62E-06	down-regulated	protein_coding
ECI2	ENSMMUG00000014021	90.88538802	-3.989659018	0.768445	-5.19186	2.08E-07	1.64E-06	down-regulated	protein_coding
FAM18B	ENSMMUG00000019203	64.59559865	-6.383132905	1.229902	-5.18995	2.10E-07	1.65E-06	down-regulated	protein_coding
CAND2	ENSMMUG00000001339	17.58415311	6.316106198	1.217245	5.188853	2.12E-07	1.66E-06	up-regulated	protein_coding
IMP3	ENSMMUG00000016103	250.4508553	1.944080228	0.374682	5.188612	2.12E-07	1.66E-06	up-regulated	protein_coding
KLHL29	ENSMMUG00000016030	51.90754537	4.147713806	0.799868	5.185498	2.15E-07	1.69E-06	up-regulated	protein_coding
RABEPK	ENSMMUG00000005215	34.84485832	2.515491364	0.485435	5.181932	2.20E-07	1.72E-06	up-regulated	protein_coding
SLC25A24	ENSMMUG00000002789	198.4761608	-2.796787715	0.539835	-5.18082	2.21E-07	1.73E-06	down-regulated	protein_coding
C2ORF29	ENSMMUG00000001147	108.3554935	1.60141777	0.30914	5.180241	2.22E-07	1.73E-06	up-regulated	protein_coding
BLOC1S1	ENSMMUG00000013227	79.9055981	-6.688648451	1.291315	-5.17972	2.22E-07	1.74E-06	down-regulated	protein_coding
RAP2A	ENSMMUG00000022747	64.67672319	-6.384834387	1.232896	-5.17873	2.23E-07	1.74E-06	down-regulated	protein_coding
KLHDC3	ENSMMUG00000000766	146.8431143	-2.830608719	0.546643	-5.17816	2.24E-07	1.75E-06	down-regulated	protein_coding
KPNA4	ENSMMUG00000018083	102.0094086	-4.103238882	0.792475	-5.17775	2.25E-07	1.75E-06	down-regulated	protein_coding
C11H12ORF29	ENSMMUG00000015860	74.65128087	2.734438908	0.528224	5.176662	2.26E-07	1.76E-06	up-regulated	protein_coding
UQCRH	ENSMMUG00000030221	135.0949438	-5.036947552	0.973435	-5.17441	2.29E-07	1.78E-06	down-regulated	protein_coding
CPNE3	ENSMMUG00000006308	115.9763935	-3.814788911	0.737416	-5.17319	2.30E-07	1.79E-06	down-regulated	protein_coding
KLHL31	ENSMMUG00000023789	18.17067609	5.185615367	1.002626	5.172035	2.32E-07	1.80E-06	up-regulated	protein_coding
SERPINB1	ENSMMUG00000011720	344.6592638	-2.67299827	0.516971	-5.1705	2.33E-07	1.82E-06	down-regulated	protein_coding
MAST4	ENSMMUG00000005357	73.98673373	2.003485977	0.38759	5.16909	2.35E-07	1.83E-06	up-regulated	protein_coding
TAX1BP1	ENSMMUG00000022454	195.5778786	-2.956331362	0.572296	-5.16574	2.39E-07	1.86E-06	down-regulated	protein_coding
ZNF233	ENSMMUG00000012797	27.52692292	3.864659967	0.748161	5.165548	2.40E-07	1.86E-06	up-regulated	protein_coding
ADGRL1	ENSMMUG00000014137	41.08191537	5.287874665	1.024293	5.162463	2.44E-07	1.89E-06	up-regulated	protein_coding
TMEM14C	ENSMMUG00000018788	125.8131003	-5.51394669	1.068244	-5.16169	2.45E-07	1.90E-06	down-regulated	protein_coding
MYOM2	ENSMMUG00000023408	12.47987332	4.914911476	0.952274	5.161238	2.45E-07	1.90E-06	up-regulated	protein_coding
SLCO2A1	ENSMMUG00000012605	34.15123839	3.413945057	0.661753	5.15894	2.48E-07	1.92E-06	up-regulated	protein_coding
RPL13A	ENSMMUG00000007551	1292.119542	-3.546875846	0.687732	-5.15735	2.50E-07	1.94E-06	down-regulated	protein_coding
DNAH9	ENSMMUG00000018539	7.552007274	5.792259602	1.123094	5.157412	2.50E-07	1.94E-06	up-regulated	protein_coding
BAG3	ENSMMUG00000000798	134.4343205	1.518743028	0.294609	5.155116	2.53E-07	1.96E-06	up-regulated	protein_coding
LIPE	ENSMMUG00000017916	166.4105757	1.76432177	0.342245	5.15515	2.53E-07	1.96E-06	up-regulated	protein_coding
TBRG4	ENSMMUG00000010823	133.2737166	-5.577274281	1.082029	-5.15446	2.54E-07	1.96E-06	down-regulated	protein_coding
U6	ENSMMUG00000036025	10.41152151	6.765579702	1.313032	5.152637	2.57E-07	1.98E-06	up-regulated	snRNA

NEDD8	ENSMMUG00000029409	78.92595748	-6.670804838	1.295066	-5.15094	2.59E-07	1.99E-06	down-regulated	protein_coding
AFF2	ENSMMUG00000000466	8.749447165	9.078436013	1.762727	5.150222	2.60E-07	2.00E-06	up-regulated	protein_coding
snoU13	ENSMMUG00000036252	8.749447165	9.078436013	1.762727	5.150222	2.60E-07	2.00E-06	up-regulated	snoRNA
TXNIP	ENSMMUG00000007338	767.0309623	-2.33666707	0.453835	-5.14872	2.62E-07	2.01E-06	down-regulated	protein_coding
CMYA5	ENSMMUG00000020733	8.739655108	6.52587467	1.268002	5.14658	2.65E-07	2.04E-06	up-regulated	protein_coding
GSTP1	ENSMMUG00000018524	606.9655797	-3.190959893	0.620505	-5.14252	2.71E-07	2.08E-06	down-regulated	protein_coding
PSMB1	ENSMMUG00000019919	140.8134387	-4.690740926	0.912335	-5.14147	2.73E-07	2.09E-06	down-regulated	protein_coding
SLC25A10	ENSMMUG00000004457	145.1738324	-3.010013908	0.585591	-5.14013	2.75E-07	2.10E-06	down-regulated	protein_coding
SRI	ENSMMUG00000022696	125.7934838	-6.372089533	1.239666	-5.14017	2.74E-07	2.10E-06	down-regulated	protein_coding
CALB1	ENSMMUG00000006664	14.30193526	7.022558238	1.366757	5.138117	2.78E-07	2.12E-06	up-regulated	protein_coding
SNX14	ENSMMUG00000005446	108.8449076	-3.659428097	0.712389	-5.13684	2.79E-07	2.14E-06	down-regulated	protein_coding
BNC2	ENSMMUG00000001141	8.815402347	7.666688706	1.492963	5.135216	2.82E-07	2.15E-06	up-regulated	protein_coding
BCAT2	ENSMMUG00000010079	193.7278195	3.344843555	0.651731	5.132247	2.86E-07	2.19E-06	up-regulated	protein_coding
YWHAH	ENSMMUG00000009945	291.2412077	-1.63826207	0.319279	-5.13114	2.88E-07	2.20E-06	down-regulated	protein_coding
TDRD7	ENSMMUG00000012275	119.6053284	-2.985817137	0.581897	-5.13117	2.88E-07	2.20E-06	down-regulated	protein_coding
SNORD24	ENSMMUG00000024743	11.32918505	7.941848035	1.548747	5.127918	2.93E-07	2.23E-06	up-regulated	snoRNA
HIST1H1C	ENSMMUG00000023732	96.26739447	3.235774542	0.631751	5.121913	3.02E-07	2.30E-06	up-regulated	protein_coding
GREB1	ENSMMUG00000020249	18.70390602	6.410633431	1.252388	5.118726	3.08E-07	2.34E-06	up-regulated	protein_coding
CD164	ENSMMUG00000007011	534.8063696	-2.242005599	0.438021	-5.11848	3.08E-07	2.34E-06	down-regulated	protein_coding
POLR2E	ENSMMUG00000013927	108.8587707	-3.670123859	0.717095	-5.11804	3.09E-07	2.35E-06	down-regulated	protein_coding
U6	ENSMMUG00000025663	8.825275235	9.087578804	1.777027	5.113923	3.16E-07	2.40E-06	up-regulated	snRNA
PROX2	ENSMMUG00000004259	8.673619094	9.067409504	1.773613	5.112393	3.18E-07	2.42E-06	up-regulated	protein_coding
FBXL13	ENSMMUG00000012427	16.02353715	4.300257235	0.841293	5.111486	3.20E-07	2.43E-06	up-regulated	protein_coding
KDR	ENSMMUG00000012669	7.431091611	6.326346168	1.23798	5.110218	3.22E-07	2.44E-06	up-regulated	protein_coding
SEC22B	ENSMMUG00000012707	65.50052698	-6.403160935	1.254149	-5.10558	3.30E-07	2.50E-06	down-regulated	protein_coding
CTRL	ENSMMUG00000021397	17.70953407	4.854021176	0.95158	5.101014	3.38E-07	2.56E-06	up-regulated	protein_coding
PRNP	ENSMMUG00000019949	88.74988431	-3.985918246	0.781498	-5.10036	3.39E-07	2.57E-06	down-regulated	protein_coding
RC3H1	ENSMMUG00000000630	65.06791677	2.256436493	0.442455	5.099812	3.40E-07	2.57E-06	up-regulated	protein_coding
CRBN	ENSMMUG00000017986	138.6420458	2.618586454	0.513502	5.099463	3.41E-07	2.58E-06	up-regulated	protein_coding
CADM4	ENSMMUG00000004615	41.94526913	2.493215592	0.489127	5.097274	3.45E-07	2.60E-06	up-regulated	protein_coding
MAL2	ENSMMUG00000004296	115.95207	-3.58867018	0.704539	-5.09364	3.51E-07	2.65E-06	down-regulated	protein_coding
NPEPPS	ENSMMUG00000005869	298.1088294	-2.196712977	0.431319	-5.09301	3.52E-07	2.66E-06	down-regulated	protein_coding
MORC4	ENSMMUG00000013150	152.2480844	3.2354246	0.635291	5.09282	3.53E-07	2.66E-06	up-regulated	protein_coding
ABCA13	ENSMMUG00000016191	11.30739107	8.61426408	1.692844	5.088633	3.61E-07	2.72E-06	up-regulated	protein_coding
TNS1	ENSMMUG00000013785	12.03540464	4.584267056	0.901452	5.085426	3.67E-07	2.76E-06	up-regulated	protein_coding
PIGB	ENSMMUG00000010797	58.38489489	1.86859483	0.367482	5.084865	3.68E-07	2.77E-06	up-regulated	protein_coding
USO1	ENSMMUG00000021835	136.2169363	-2.809934282	0.552662	-5.08436	3.69E-07	2.77E-06	down-regulated	protein_coding
PPA1	ENSMMUG00000006397	124.36074	-5.509650606	1.083809	-5.0836	3.70E-07	2.78E-06	down-regulated	protein_coding
KMT2C	ENSMMUG00000007212	345.6257835	1.901465315	0.374037	5.083624	3.70E-07	2.78E-06	up-regulated	protein_coding
5S_rRNA	ENSMMUG00000025702	8.627695314	8.348035088	1.642535	5.08241	3.73E-07	2.80E-06	up-regulated	rRNA
Y_RNA	ENSMMUG00000034413	8.627695314	8.348035088	1.642535	5.08241	3.73E-07	2.80E-06	up-regulated	misc_RNA
SAC3D1	ENSMMUG00000031168	105.2766446	3.148225606	0.619848	5.079032	3.79E-07	2.84E-06	up-regulated	protein_coding
PPM1N	ENSMMUG00000011715	11.23722881	5.474300637	1.078387	5.076377	3.85E-07	2.88E-06	up-regulated	protein_coding
C4orf17	ENSMMUG00000009089	7.721063996	6.761716005	1.332294	5.075243	3.87E-07	2.90E-06	up-regulated	protein_coding
TM4SF20	ENSMMUG00000013245	842.1424286	-3.278774847	0.646143	-5.07438	3.89E-07	2.91E-06	down-regulated	protein_coding
CASP3	ENSMMUG00000012053	72.16849464	-6.542983155	1.289855	-5.07265	3.92E-07	2.94E-06	down-regulated	protein_coding
CAND1	ENSMMUG00000008300	126.908454	-2.504295722	0.493757	-5.07192	3.94E-07	2.94E-06	down-regulated	protein_coding
VDAC2	ENSMMUG00000008691	678.6585614	-1.785627633	0.352063	-5.0719	3.94E-07	2.94E-06	down-regulated	protein_coding
BUB3	ENSMMUG00000003092	59.08004938	-6.253241487	1.233083	-5.07122	3.95E-07	2.95E-06	down-regulated	protein_coding
FZR1	ENSMMUG00000019870	107.093763	-2.889594731	0.569919	-5.07018	3.97E-07	2.97E-06	down-regulated	protein_coding
TM9SF4	ENSMMUG00000017416	185.705887	-2.043386684	0.403152	-5.06853	4.01E-07	2.99E-06	down-regulated	protein_coding
BSDC1	ENSMMUG00000001264	191.8237077	-1.945496223	0.383852	-5.06835	4.01E-07	2.99E-06	down-regulated	protein_coding
TAS1R1	ENSMMUG00000018183	31.86751871	6.011344052	1.187133	5.063751	4.11E-07	3.06E-06	up-regulated	protein_coding
ARL6IP1	ENSMMUG00000018491	122.5193014	-3.770421375	0.745187	-5.0597	4.20E-07	3.13E-06	down-regulated	protein_coding
TMEM219	ENSMMUG00000020273	119.6996629	-4.12919801	0.816237	-5.05882	4.22E-07	3.14E-06	down-regulated	protein_coding
MYO3B	ENSMMUG00000016256	9.579600703	7.651222145	1.512501	5.058657	4.22E-07	3.14E-06	up-regulated	protein_coding
PSMA2	ENSMMUG00000022451	119.8347995	-5.388808009	1.065855	-5.05586	4.28E-07	3.18E-06	down-regulated	protein_coding
IGSF9B	ENSMMUG00000012410	18.60611175	3.353965637	0.663614	5.054089	4.32E-07	3.21E-06	up-regulated	protein_coding
BBX	ENSMMUG00000008787	99.39453672	3.022095458	0.598147	5.052433	4.36E-07	3.24E-06	up-regulated	protein_coding
CCNL2	ENSMMUG00000006904	253.8817383	-2.24625335	0.444653	-5.0517	4.38E-07	3.25E-06	down-regulated	protein_coding
NEURL	ENSMMUG00000007064	86.69193695	2.625923892	0.519862	5.05119	4.39E-07	3.26E-06	up-regulated	protein_coding
SNX1	ENSMMUG00000014005	146.4184066	-2.276461921	0.450913	-5.04857	4.45E-07	3.30E-06	down-regulated	protein_coding
SLC16A4	ENSMMUG00000015022	86.80944361	3.248516383	0.643674	5.046838	4.49E-07	3.33E-06	up-regulated	protein_coding
KIAA2018	ENSMMUG00000011039	123.885451	2.902062884	0.575656	5.041312	4.62E-07	3.42E-06	up-regulated	protein_coding
FAM129B	ENSMMUG00000009961	419.317613	-2.13868117	0.424278	-5.04075	4.64E-07	3.43E-06	down-regulated	protein_coding
SLC35C1	ENSMMUG00000002452	108.0769954	-3.02730047	0.600778	-5.03897	4.68E-07	3.46E-06	down-regulated	protein_coding
TMEM123	ENSMMUG00000003403	312.9864962	-2.415704035	0.479771	-5.03512	4.78E-07	3.53E-06	down-regulated	protein_coding
APH1A	ENSMMUG00000021386	205.5874966	-2.316563171	0.460298	-5.03274	4.84E-07	3.57E-06	down-regulated	protein_coding
PNPLA6	ENSMMUG00000011392	176.3198906	-3.077016565	0.612197	-5.02619	5.00E-07	3.69E-06	down-regulated	protein_coding
LTBP3	ENSMMUG00000023479	55.1123243	3.158903464	0.628557	5.025646	5.02E-07	3.70E-06	up-regulated	protein_coding
ACSM3	ENSMMUG00000016745	88.01859246	4.210652193	0.838074	5.024199	5.06E-07	3.73E-06	up-regulated	protein_coding
PCGF5	ENSMMUG00000020001	119.4636977	-4.45163197	0.88725	-5.01734	5.24E-07	3.86E-06	down-regulated	protein_coding
PSTPIP1	ENSMMUG00000010171	24.57566715	5.390674891	1.074764	5.015683	5.28E-07	3.89E-06	up-regulated	protein_coding
EHMT2	ENSMMUG00000011679	355.2094804	1.966893548	0.39217	5.015413	5.29E-07	3.89E-06	up-regulated	protein_coding
GCNT1	ENSMMUG00000020406	99.99883258	-4.598348749	0.917111	-5.01395	5.33E-07	3.92E-06	down-regulated	protein_coding
PDE8B	ENSMMUG00000021358	24.91754133	4.125434391	0.822817	5.013795	5.34E-07	3.92E-06	up-regulated	protein_coding
U6	ENSMMUG00000034348	14.50966579	8.391449435	1.673883	5.013163	5.35E-07	3.93E-06	up-regulated	snRNA
USP7	ENSMMUG00000004059	216.4769389	-1.697145326	0.338597	-5.01229	5.38E-07	3.95E-06	down-regulated	protein_coding
NKPD1	ENSMMUG00000022931	96.55204011	4.183070379	0.834853	5.010548	5.43E-07	3.98E-06	up-regulated	protein_coding
RIC1	ENSMMUG00000009020	97.64226345	1.801195376	0.359648	5.008212	5.49E-07	4.03E-06	up-regulated	protein_coding
C17orf78	ENSMMUG00000030090	97.38811834	-6.975078296	1.393107	-5.00685	5.53E-07	4.05E-06	down-regulated	protein_coding
TAS2R40	ENSMMUG00000005967	8.901103305	9.095210138	1.817051	5.005479	5.57E-07	4.08E-06	up-regulated	protein_coding
CDH10	ENSMMUG00000012293	8.901103305	9.095210138	1.817051	5.005479	5.57E-07	4.08E-06	up-regulated	protein_coding
CNPY4	ENSMMUG00000013452	39.84845463	2.93954803	0.587362	5.00466	5.60E-07	4.09E-06	up-regulated	protein_coding
RPS25	ENSMMUG00000030153	139.6414131	-4.682030663	0.935649	-5.00405	5.61E-07	4.10E-06	down-regulated	protein_coding
LRP2BP	ENSMMUG00000003519	37.98782076	3.806382017	0.760721	5.003653	5.63E-07	4.11E-06	up-regulated	protein_coding
BAX	ENSMMUG00000003907	147.554116	-5.067232265	1.01273	-5.00354	5.63E-07	4.11E-06	down-regulated	protein_coding
C14ORF147	ENSMMUG00000006360	52.66499403	-6.087839333	1.216863	-5.0029	5.65E-07	4.12E-06	down-regulated	protein_coding
AMOTL2	ENSMMUG00000021694	267.246389	1.735444034	0.34694	5.002144	5.67E-07	4.14E-06	up-regulated	protein_coding
CD47	ENSMMUG00000008791	111.2716882	-5.284613421	1.056605	-5.0015	5.69E-07	4.15E-06	down-regulated	protein_coding
TLCD1	ENSMMUG00000019313	53.51436884	2.915445297	0.582954	5.001157	5.70E-07	4.15E-06	up-regulated	protein_coding
NDUFB4	ENSMMUG00000001592	124.6591969	-4.082598918	0.816767	-4.99849	5.78E-07	4.21E-06	down-regulated	protein_coding
TSTD1	ENSMMUG00000002001	124.5546945	-4.49842969	0.900099	-4.9977	5.80E-07	4.22E-06	down-regulated	protein_coding
SETD6	ENSMMUG00000014621	71.08365803	2.384544919	0.477219	4.996755	5.83E-07	4.24E-06	up-regulated	protein_coding
POMP	ENSMMUG00000000097	116.7188997	-6.26232748	1.253902	-4.99427	5.91E-07	4.29E-06	down-regulated	protein_coding
HNRNPU	ENSMMUG00000021098	379.300118	-1.336860117	0.267682	-4.9942	5.91E-07	4.29E-06	down-regulated	protein_coding
MGRN1	ENSMMUG00000001888	143.3702788	1.79882912	0.360561	4.988975	6.07E-07	4.41E-06	up-regulated	protein_coding
PEX6	ENSMMUG00000001394	128.0794309	-3.899386321	0.781748	-4.98804	6.10E-07	4.43E-06	down-regulated	protein_coding
YME1L1	ENSMMUG00000016406	119.6475046	-2.467819231	0.494901	-4.98649	6.15E-07	4.46E-06	down-regulated	protein_coding
GRID1	ENSMMUG00000023612	8.107050343	6.460318604	1.2958	4.985585	6.18E-07	4.48E-06	up-regulated	protein_coding

RTF1	ENSMMUG00000015482	98.15648772	-2.697150933	0.541568	-4.98026	6.35E-07	4.60E-06	down-regulated	protein_coding
TCEAL3	ENSMMUG00000003501	12.41446299	6.212426362	1.247772	4.978814	6.40E-07	4.63E-06	up-regulated	protein_coding
C4H6ORF89	ENSMMUG00000005004	85.37790097	-3.313817442	0.665604	-4.97866	6.40E-07	4.63E-06	down-regulated	protein_coding
AGK	ENSMMUG00000021017	94.87135629	2.029576125	0.407656	4.978645	6.40E-07	4.63E-06	up-regulated	protein_coding
CSDC2	ENSMMUG00000001852	15.06408871	5.433125683	1.091915	4.975779	6.50E-07	4.69E-06	up-regulated	protein_coding
SDC4	ENSMMUG00000003406	173.2003345	-2.370900073	0.476584	-4.97478	6.53E-07	4.71E-06	down-regulated	protein_coding
KLC4	ENSMMUG00000011668	221.8773297	-1.947684188	0.391516	-4.97472	6.53E-07	4.71E-06	down-regulated	protein_coding
C14H11ORF42	ENSMMUG00000016881	19.00312014	5.104647695	1.026503	4.972853	6.60E-07	4.76E-06	up-regulated	protein_coding
EIF3M	ENSMMUG00000009026	110.463972	-4.323327107	0.869421	-4.97265	6.60E-07	4.76E-06	down-regulated	protein_coding
SLC35A4	ENSMMUG00000001725	107.9873159	-2.559090903	0.514667	-4.97233	6.62E-07	4.77E-06	down-regulated	protein_coding
TSLP	ENSMMUG00000004669	11.77186137	6.314407156	1.270251	4.970992	6.66E-07	4.80E-06	up-regulated	protein_coding
SNORA81	ENSMMUG00000037227	7.586016958	6.393996644	1.286283	4.970911	6.66E-07	4.80E-06	up-regulated	snoRNA
PRKCQ	ENSMMUG00000020776	26.56975772	5.808700589	1.168617	4.970575	6.68E-07	4.80E-06	up-regulated	protein_coding
FDX1	ENSMMUG00000002110	228.5433204	-1.938214041	0.390513	-4.96325	6.93E-07	4.98E-06	down-regulated	protein_coding
ZG16	ENSMMUG00000009686	175.4980974	-4.887233624	0.984674	-4.9633	6.93E-07	4.98E-06	down-regulated	protein_coding
MDK	ENSMMUG00000021448	151.2133237	-2.369557618	0.477523	-4.96218	6.97E-07	5.01E-06	down-regulated	protein_coding
CABYR	ENSMMUG00000004346	43.21187548	3.247176654	0.654583	4.960677	7.02E-07	5.04E-06	up-regulated	protein_coding
TSHR	ENSMMUG00000019713	7.78709034	8.203935856	1.653852	4.960501	7.03E-07	5.04E-06	up-regulated	protein_coding
HMCN1	ENSMMUG00000010474	21.23684176	5.301144053	1.069026	4.958853	7.09E-07	5.09E-06	up-regulated	protein_coding
PREPL	ENSMMUG00000006311	664.1546776	3.245422852	0.654551	4.958245	7.11E-07	5.10E-06	up-regulated	protein_coding
USH1C	ENSMMUG00000023582	238.7204421	-1.703703658	0.34368	-4.95724	7.15E-07	5.12E-06	down-regulated	protein_coding
MED15	ENSMMUG00000023263	97.92449377	-2.747993494	0.554598	-4.95493	7.24E-07	5.18E-06	down-regulated	protein_coding
RPL11	ENSMMUG00000002445	573.6589902	-3.00602938	0.606719	-4.95456	7.25E-07	5.19E-06	down-regulated	protein_coding
FBLN5	ENSMMUG00000008447	62.36440881	4.237502812	0.855279	4.954527	7.25E-07	5.19E-06	up-regulated	protein_coding
UBE2K	ENSMMUG00000011325	51.51728205	-6.055697148	1.223877	-4.94796	7.50E-07	5.36E-06	down-regulated	protein_coding
PSMB4	ENSMMUG00000020924	192.3428246	-2.852288936	0.576565	-4.94704	7.54E-07	5.38E-06	down-regulated	protein_coding
GRN	ENSMMUG00000023751	624.1538223	-1.233305452	0.24933	-4.94648	7.56E-07	5.39E-06	down-regulated	protein_coding
PPP2CB	ENSMMUG00000020819	238.2226142	-3.561302658	0.720016	-4.94614	7.57E-07	5.40E-06	down-regulated	protein_coding
APOA4	ENSMMUG00000016125	7061.733892	-3.123860576	0.631792	-4.94445	7.64E-07	5.44E-06	down-regulated	protein_coding
UBE2B	ENSMMUG00000016641	84.61426269	-3.906426565	0.790199	-4.9436	7.67E-07	5.46E-06	down-regulated	protein_coding
BIRC2	ENSMMUG00000003400	93.70253254	-3.470734941	0.702267	-4.94219	7.73E-07	5.50E-06	down-regulated	protein_coding
ATP1B3	ENSMMUG00000012256	251.7859349	-3.195876919	0.646639	-4.94229	7.72E-07	5.50E-06	down-regulated	protein_coding
SEC31A	ENSMMUG00000008501	201.4262801	-2.392698527	0.484217	-4.94137	7.76E-07	5.52E-06	down-regulated	protein_coding
SUB1	ENSMMUG00000011470	106.2237774	-4.700957732	0.951636	-4.93987	7.82E-07	5.56E-06	down-regulated	protein_coding
BCL9L	ENSMMUG00000009318	231.8355799	1.904467179	0.385803	4.93637	7.96E-07	5.66E-06	up-regulated	protein_coding
ZBTB25	ENSMMUG00000004747	17.38921798	3.220611093	0.652701	4.93428	8.04E-07	5.71E-06	up-regulated	protein_coding
COL19A1	ENSMMUG00000005574	13.17657976	7.640037075	1.549116	4.93187	8.14E-07	5.78E-06	up-regulated	protein_coding
SETD3	ENSMMUG00000001199	114.0965481	-2.57713484	0.523327	-4.92452	8.46E-07	5.99E-06	down-regulated	protein_coding
IRF3	ENSMMUG00000022735	318.2556962	-2.251300183	0.457237	-4.9237	8.49E-07	6.01E-06	down-regulated	protein_coding
TSG101	ENSMMUG00000014613	115.6535201	-2.192422074	0.445328	-4.92317	8.52E-07	6.03E-06	down-regulated	protein_coding
PMPCB	ENSMMUG00000003429	94.90823812	-3.397864804	0.690324	-4.92213	8.56E-07	6.05E-06	down-regulated	protein_coding
ROGDI	ENSMMUG00000017946	306.0202631	1.839703405	0.373798	4.921655	8.58E-07	6.06E-06	up-regulated	protein_coding
SPATS2L	ENSMMUG00000008101	116.7995242	-3.394142413	0.689667	-4.92142	8.59E-07	6.07E-06	down-regulated	protein_coding
TRAM1	ENSMMUG00000001516	217.3276011	-1.785586729	0.362845	-4.92107	8.61E-07	6.08E-06	down-regulated	protein_coding
JMJD4	ENSMMUG00000001867	307.4538278	2.738392246	0.556487	4.920856	8.62E-07	6.08E-06	up-regulated	protein_coding
RPS9	ENSMMUG00000022552	838.9295125	-3.293926109	0.669562	-4.91952	8.68E-07	6.12E-06	down-regulated	protein_coding
NDUFB6	ENSMMUG00000002685	59.14741103	-6.254420521	1.271478	-4.91901	8.70E-07	6.13E-06	down-regulated	protein_coding
RSPO2	ENSMMUG00000010745	9.889790127	9.238475	1.878514	4.917971	8.74E-07	6.16E-06	up-regulated	protein_coding
PQLC3	ENSMMUG00000022785	56.9101373	3.481640289	0.70827	4.915698	8.85E-07	6.23E-06	up-regulated	protein_coding
FBN2	ENSMMUG00000010682	22.17402697	3.457315465	0.70336	4.915428	8.86E-07	6.24E-06	up-regulated	protein_coding
RPL7	ENSMMUG00000005141	735.6194256	-3.448914019	0.701665	-4.91533	8.86E-07	6.24E-06	down-regulated	protein_coding
TSPAN4	ENSMMUG00000031282	25.02325997	4.195686669	0.853809	4.914082	8.92E-07	6.27E-06	up-regulated	protein_coding
DNAJA1	ENSMMUG00000007740	110.8427925	-2.404587023	0.489781	-4.90952	9.13E-07	6.42E-06	down-regulated	protein_coding
EYA3	ENSMMUG00000012965	47.10257579	2.928669053	0.596594	4.908978	9.16E-07	6.43E-06	up-regulated	protein_coding
ROR1	ENSMMUG00000004668	9.10898632	4.75821483	0.969392	4.908452	9.18E-07	6.45E-06	up-regulated	protein_coding
CMTM8	ENSMMUG00000012675	35.65374056	3.00689668	0.612896	4.906046	9.29E-07	6.52E-06	up-regulated	protein_coding
GDAP1L1	ENSMMUG00000002839	9.004335619	8.390911668	1.711147	4.903676	9.41E-07	6.60E-06	up-regulated	protein_coding
WDR45L	ENSMMUG00000021803	114.4298764	-3.562382113	0.72652	-4.90335	9.42E-07	6.61E-06	down-regulated	protein_coding
TF	ENSMMUG00000008916	99.44271008	-2.940668953	0.599969	-4.90137	9.52E-07	6.67E-06	down-regulated	protein_coding
OMD	ENSMMUG00000004636	9.358993635	9.170982264	1.871248	4.900998	9.54E-07	6.68E-06	up-regulated	protein_coding
MAZ	ENSMMUG00000019826	150.8659884	-3.301086644	0.673735	-4.89968	9.60E-07	6.72E-06	down-regulated	protein_coding
EMP1	ENSMMUG00000017638	381.8771621	-1.579969684	0.322519	-4.89885	9.64E-07	6.75E-06	down-regulated	protein_coding
HSD17B12	ENSMMUG00000018465	96.10424008	-4.111368368	0.839807	-4.89561	9.80E-07	6.85E-06	down-regulated	protein_coding
DNASE1L2	ENSMMUG00000019236	24.41226927	4.606934313	0.941562	4.892861	9.94E-07	6.95E-06	up-regulated	protein_coding
RHEB	ENSMMUG00000006250	100.4524707	-3.175723321	0.649329	-4.89078	1.00E-06	7.02E-06	down-regulated	protein_coding
TVP23A	ENSMMUG00000002823	7.084861224	4.933659338	1.008949	4.889901	1.01E-06	7.05E-06	up-regulated	protein_coding
KCNA10	ENSMMUG00000016056	16.17327845	6.861879455	1.403686	4.88847	1.02E-06	7.09E-06	up-regulated	protein_coding
MMU.251	ENSMMUG00000015563	648.2522837	-3.050610804	0.624131	-4.88777	1.02E-06	7.12E-06	down-regulated	protein_coding
SYNE1	ENSMMUG00000019048	36.71906864	2.925732583	0.598678	4.886986	1.02E-06	7.14E-06	up-regulated	protein_coding
PCMTD1	ENSMMUG00000003347	56.17264773	-6.181546183	1.265146	-4.88603	1.03E-06	7.17E-06	down-regulated	protein_coding
CEBPA	ENSMMUG00000006124	87.31401279	-4.382539382	0.897045	-4.88553	1.03E-06	7.18E-06	down-regulated	protein_coding
C17orf104	ENSMMUG00000015047	9.128381097	4.109852319	0.841265	4.885322	1.03E-06	7.19E-06	up-regulated	protein_coding
MYBPC3	ENSMMUG00000015604	15.38471205	6.900654629	1.413293	4.882679	1.05E-06	7.28E-06	up-regulated	protein_coding
GLO1	ENSMMUG00000001638	163.1953158	-3.342550895	0.684927	-4.88016	1.06E-06	7.36E-06	down-regulated	protein_coding
ATG14	ENSMMUG00000003761	69.08549138	2.477567417	0.507685	4.880126	1.06E-06	7.36E-06	up-regulated	protein_coding
G3BP1	ENSMMUG00000014701	146.5625545	-2.511653331	0.514671	-4.88012	1.06E-06	7.36E-06	down-regulated	protein_coding
PTPN2	ENSMMUG00000017550	12.74798988	5.659284487	1.160009	4.878656	1.07E-06	7.41E-06	up-regulated	protein_coding
DUSP16	ENSMMUG00000018580	595.4375559	2.084928775	0.427574	4.87618	1.08E-06	7.50E-06	up-regulated	protein_coding
STXBP5L	ENSMMUG00000011161	11.85849493	6.788221891	1.392404	4.87518	1.09E-06	7.54E-06	up-regulated	protein_coding
THOC6	ENSMMUG00000007666	241.0176387	2.400130843	0.492343	4.874921	1.09E-06	7.54E-06	up-regulated	protein_coding
LDHD	ENSMMUG00000022037	160.2262534	-1.944004835	0.398872	-4.87375	1.09E-06	7.58E-06	down-regulated	protein_coding
PKP3	ENSMMUG00000003525	432.5365673	-2.316368471	0.475455	-4.8719	1.11E-06	7.65E-06	down-regulated	protein_coding
ITFG1	ENSMMUG00000004717	177.0477621	-2.790775563	0.572864	-4.87162	1.11E-06	7.66E-06	down-regulated	protein_coding
KRT83	ENSMMUG00000031925	9.635658183	5.558755479	1.141852	4.868192	1.13E-06	7.79E-06	up-regulated	protein_coding
RPS13	ENSMMUG00000022547	388.0223256	-3.808385016	0.782459	-4.8672	1.13E-06	7.82E-06	down-regulated	protein_coding
RPLP0	ENSMMUG00000009157	1970.244551	-3.1734085	0.652391	-4.86427	1.15E-06	7.93E-06	down-regulated	protein_coding
EIF2S3	ENSMMUG00000006979	170.6075863	-1.704758181	0.350533	-4.86333	1.15E-06	7.97E-06	down-regulated	protein_coding
FAM217A	ENSMMUG00000014019	10.96324836	5.502712504	1.131951	4.861265	1.17E-06	8.04E-06	up-regulated	protein_coding
PSIP1	ENSMMUG00000002224	85.87956919	2.204623415	0.453526	4.861074	1.17E-06	8.05E-06	up-regulated	protein_coding
KCNJ14	ENSMMUG00000001792	36.34118956	3.722534857	0.766029	4.859521	1.18E-06	8.11E-06	up-regulated	protein_coding
HIST1H2AC	ENSMMUG00000023728	9.74666616	9.189945787	1.89297	4.854775	1.21E-06	8.30E-06	up-regulated	protein_coding
NAE1	ENSMMUG00000006752	96.32139922	-3.504873753	0.722051	-4.85405	1.21E-06	8.32E-06	down-regulated	protein_coding
NTRK3	ENSMMUG00000001386	16.21566316	7.95614069	1.639542	4.852659	1.22E-06	8.38E-06	up-regulated	protein_coding
TAGLN2	ENSMMUG00000004705	1013.976402	-2.223299305	0.458185	-4.8524	1.22E-06	8.38E-06	down-regulated	protein_coding
ACAP3	ENSMMUG00000013862	114.5563224	-4.068477704	0.83879	-4.85041	1.23E-06	8.46E-06	down-regulated	protein_coding
ARSA	ENSMMUG00000016274	626.774731	1.487284575	0.306819	4.847437	1.25E-06	8.58E-06	up-regulated	protein_coding
TM7SF2	ENSMMUG00000018773	111.9483301	-4.373786391	0.902305	-4.84735	1.25E-06	8.58E-06	down-regulated	protein_coding
SF3B3	ENSMMUG00000017812	190.9246563	-2.147425926	0.443023	-4.84721	1.25E-06	8.58E-06	down-regulated	protein_coding
IGSF10	ENSMMUG00000001107	9.466761969	8.409055188	1.735087	4.846474	1.26E-06	8.61E-06	up-regulated	protein_coding
WNK3	ENSMMUG00000005433	7.778220537	5.525260194	1.140165	4.846016	1.26E-06	8.63E-06	up-regulated	protein_coding

QRICH2	ENSMMUG00000003539	11.49113322	5.101950799	1.052848	4.845856	1.26E-06	8.63E-06	up-regulated	protein_coding
CXCL17	ENSMMUG00000017919	14.34757786	4.532428342	0.935715	4.843815	1.27E-06	8.71E-06	up-regulated	protein_coding
PPM1A	ENSMMUG00000010464	120.9081252	-3.429283237	0.708054	-4.84325	1.28E-06	8.73E-06	down-regulated	protein_coding
FAM98A	ENSMMUG00000010462	46.66864802	-5.91289777	1.221071	-4.84239	1.28E-06	8.76E-06	down-regulated	protein_coding
RAB2A	ENSMMUG00000017038	95.46845851	-5.090851922	1.051393	-4.84201	1.29E-06	8.78E-06	down-regulated	protein_coding
SF3B14	ENSMMUG00000001282	51.53155193	-6.055593193	1.251028	-4.84049	1.30E-06	8.84E-06	down-regulated	protein_coding
FN1	ENSMMUG00000012321	53.33968814	2.651943158	0.548132	4.838146	1.31E-06	8.94E-06	up-regulated	protein_coding
PSME4	ENSMMUG00000008104	93.70057181	-2.993597746	0.618907	-4.83691	1.32E-06	8.99E-06	down-regulated	protein_coding
IGFBP7	ENSMMUG00000014011	32.42201241	3.61591239	0.747906	4.834712	1.33E-06	9.09E-06	up-regulated	protein_coding
ITGB7	ENSMMUG00000005245	66.4633434	3.265971198	0.675639	4.833902	1.34E-06	9.12E-06	up-regulated	protein_coding
FAM96B	ENSMMUG00000010919	100.1512058	-4.511309772	0.933327	-4.83358	1.34E-06	9.13E-06	down-regulated	protein_coding
TMEM198	ENSMMUG00000013241	109.4182972	4.125131117	0.853652	4.832334	1.35E-06	9.18E-06	up-regulated	protein_coding
HADHA	ENSMMUG00000004380	279.1205456	-2.045360905	0.42329	-4.83205	1.35E-06	9.19E-06	down-regulated	protein_coding
CDK10	ENSMMUG00000002503	65.51930319	2.202489443	0.455826	4.831867	1.35E-06	9.20E-06	up-regulated	protein_coding
PRSS50	ENSMMUG00000021250	12.15578279	5.488459712	1.13655	4.829053	1.37E-06	9.32E-06	up-regulated	protein_coding
RNF150	ENSMMUG00000010444	9.604937286	6.245843915	1.29409	4.826439	1.39E-06	9.43E-06	up-regulated	protein_coding
ITFG3	ENSMMUG00000000599	1048.64704	-1.415401744	0.293279	-4.82613	1.39E-06	9.44E-06	down-regulated	protein_coding
ANKRD23	ENSMMUG00000007591	19.55407145	3.89211757	0.807	4.822944	1.41E-06	9.58E-06	up-regulated	protein_coding
MAPRE1	ENSMMUG00000001816	75.11558812	-3.369230811	0.698627	-4.82265	1.42E-06	9.59E-06	down-regulated	protein_coding
CD151	ENSMMUG00000020623	394.0654315	-1.827227156	0.378889	-4.8226	1.42E-06	9.59E-06	down-regulated	protein_coding
ACSS1	ENSMMUG00000020597	201.2299393	-1.897950367	0.393954	-4.81769	1.45E-06	9.82E-06	down-regulated	protein_coding
ZBTB37	ENSMMUG00000022801	12.37826005	3.692253458	0.766638	4.816162	1.46E-06	9.89E-06	up-regulated	protein_coding
BCAP31	ENSMMUG00000012744	247.1211835	-2.242815271	0.465734	-4.81566	1.47E-06	9.91E-06	down-regulated	protein_coding
CSNK2A1	ENSMMUG00000031224	183.9296333	-2.184195357	0.45363	-4.81492	1.47E-06	9.95E-06	down-regulated	protein_coding
CCNE2	ENSMMUG00000005358	59.56797203	3.685274024	0.766218	4.809696	1.51E-06	1.02E-05	up-regulated	protein_coding
U4	ENSMMUG00000034826	10.28323714	7.315048949	1.52117	4.80883	1.52E-06	1.02E-05	up-regulated	snRNA
EMILIN2	ENSMMUG00000017546	77.083757	4.607450874	0.958226	4.808312	1.52E-06	1.03E-05	up-regulated	protein_coding
S100A11	ENSMMUG00000004441	544.8504653	-2.691664235	0.559819	-4.8081	1.52E-06	1.03E-05	down-regulated	protein_coding
RICH2	ENSMMUG00000022515	91.04522782	2.27316003	0.4728	4.807869	1.53E-06	1.03E-05	up-regulated	protein_coding
SLC18A2	ENSMMUG00000014817	22.82342654	4.287459449	0.891952	4.80683	1.53E-06	1.03E-05	up-regulated	protein_coding
BLVRB	ENSMMUG00000015603	213.4804222	-3.947352602	0.821662	-4.80411	1.55E-06	1.05E-05	down-regulated	protein_coding
GSKIP	ENSMMUG00000003395	96.18576455	-5.069465943	1.055258	-4.804	1.56E-06	1.05E-05	down-regulated	protein_coding
LPP	ENSMMUG00000016823	172.3072071	2.640199151	0.549745	4.802589	1.57E-06	1.05E-05	up-regulated	protein_coding
DNASE1L1	ENSMMUG00000005495	217.5084024	1.678993276	0.349679	4.801523	1.57E-06	1.06E-05	up-regulated	protein_coding
snoU13	ENSMMUG00000036650	8.521962953	9.038761388	1.882597	4.80122	1.58E-06	1.06E-05	up-regulated	snoRNA
RRAS2	ENSMMUG00000000664	82.89818341	-4.316070814	0.89921	-4.79985	1.59E-06	1.07E-05	down-regulated	protein_coding
CA10	ENSMMUG00000013737	9.143086746	7.096753181	1.478542	4.799832	1.59E-06	1.07E-05	up-regulated	protein_coding
APBB3	ENSMMUG00000001707	42.62148561	2.335980551	0.486907	4.797591	1.61E-06	1.08E-05	up-regulated	protein_coding
SYNE3	ENSMMUG00000004931	71.85663264	2.928432262	0.610449	4.797175	1.61E-06	1.08E-05	up-regulated	protein_coding
RNF181	ENSMMUG00000017824	55.75882529	-6.169251829	1.286153	-4.79667	1.61E-06	1.08E-05	down-regulated	protein_coding
CDK5RAP3	ENSMMUG00000004760	126.1601815	-2.51619826	0.524672	-4.79575	1.62E-06	1.09E-05	down-regulated	protein_coding
NEK9	ENSMMUG00000015166	172.9802306	-1.873219885	0.390967	-4.79125	1.66E-06	1.11E-05	down-regulated	protein_coding
PROB1	ENSMMUG00000029825	13.49168974	3.687586013	0.77044	4.786338	1.70E-06	1.14E-05	up-regulated	protein_coding
TOP2B	ENSMMUG00000007371	159.33851	-1.842133407	0.385019	-4.78452	1.71E-06	1.15E-05	down-regulated	protein_coding
TOR1A	ENSMMUG00000003794	115.3751229	1.777829165	0.371632	4.783847	1.72E-06	1.15E-05	up-regulated	protein_coding
DGKA	ENSMMUG00000018814	193.5857653	-1.828747118	0.382334	-4.78311	1.73E-06	1.15E-05	down-regulated	protein_coding
FAM3D	ENSMMUG00000011947	210.4865112	-2.349979279	0.49158	-4.78046	1.75E-06	1.17E-05	down-regulated	protein_coding
SIGMAR1	ENSMMUG00000020774	134.5512478	-2.874067079	0.601523	-4.77799	1.77E-06	1.18E-05	down-regulated	protein_coding
TMEM150A	ENSMMUG00000017825	39.8251441	2.42071495	0.506968	4.774888	1.80E-06	1.20E-05	up-regulated	protein_coding
REC8	ENSMMUG00000012480	33.62117496	4.585252468	0.960374	4.774445	1.80E-06	1.20E-05	up-regulated	protein_coding
PDGFA	ENSMMUG00000004949	245.1740835	1.285841271	0.269633	4.768861	1.85E-06	1.23E-05	up-regulated	protein_coding
TMEM87B	ENSMMUG00000002231	129.2504583	-2.61963426	0.549479	-4.76749	1.87E-06	1.24E-05	down-regulated	protein_coding
PDE6C	ENSMMUG00000014713	10.63773247	4.984157645	1.045607	4.76676	1.87E-06	1.25E-05	up-regulated	protein_coding
FAM210B	ENSMMUG00000000069	88.34921262	-2.54897107	0.535218	-4.76249	1.91E-06	1.27E-05	down-regulated	protein_coding
AMHR2	ENSMMUG00000020037	9.79314319	4.407183555	0.926158	4.758564	1.95E-06	1.29E-05	up-regulated	protein_coding
GGA1	ENSMMUG00000003853	245.1244123	1.618257905	0.340083	4.758427	1.95E-06	1.29E-05	up-regulated	protein_coding
MEX3A	ENSMMUG00000022307	17.95549734	3.851724103	0.809505	4.75812	1.95E-06	1.29E-05	up-regulated	protein_coding
ARPC1B	ENSMMUG00000001458	689.8284019	-1.969302602	0.414063	-4.75605	1.97E-06	1.31E-05	down-regulated	protein_coding
SCOC	ENSMMUG00000022351	82.58666928	-5.758642717	1.210822	-4.75598	1.97E-06	1.31E-05	down-regulated	protein_coding
SNX2	ENSMMUG00000023598	89.38933735	-3.407460924	0.716433	-4.75615	1.97E-06	1.31E-05	down-regulated	protein_coding
ATXN7L1	ENSMMUG00000000383	28.0584038	2.293561757	0.482507	4.753432	2.00E-06	1.32E-05	up-regulated	protein_coding
SLC25A4	ENSMMUG00000015472	93.75497583	-5.061233133	1.064876	-4.75289	2.01E-06	1.33E-05	down-regulated	protein_coding
DUOX1	ENSMMUG00000008752	18.21369079	2.987083978	0.628638	4.751678	2.02E-06	1.33E-05	up-regulated	protein_coding
VPS29	ENSMMUG00000009255	88.88599264	-5.86686272	1.23478	-4.75134	2.02E-06	1.33E-05	down-regulated	protein_coding
MAMDC4	ENSMMUG00000005048	257.3967207	2.604559932	0.548216	4.750978	2.02E-06	1.34E-05	up-regulated	protein_coding
BACE2	ENSMMUG00000004257	185.4413994	-3.795915336	0.799031	-4.75065	2.03E-06	1.34E-05	down-regulated	protein_coding
U6	ENSMMUG00000027859	9.388826763	6.260115472	1.317879	4.750145	2.03E-06	1.34E-05	up-regulated	snRNA
UBE2V2	ENSMMUG00000008223	72.23960822	-3.672918301	0.773367	-4.74926	2.04E-06	1.35E-05	down-regulated	protein_coding
RPL22L1	ENSMMUG00000022881	75.63812869	-6.609513541	1.391738	-4.74911	2.04E-06	1.35E-05	down-regulated	protein_coding
FAM158A	ENSMMUG00000012471	161.7070562	2.545896994	0.536148	4.748493	2.05E-06	1.35E-05	up-regulated	protein_coding
HMGB1	ENSMMUG00000023399	274.9270666	-3.628671434	0.76423	-4.74814	2.05E-06	1.35E-05	down-regulated	protein_coding
CARNS1	ENSMMUG00000005480	27.13114884	3.65111634	0.769029	4.747697	2.06E-06	1.35E-05	up-regulated	protein_coding
ARL13A	ENSMMUG00000019971	9.901152685	5.764740131	1.214363	4.747132	2.06E-06	1.36E-05	up-regulated	protein_coding
ACTR3	ENSMMUG00000015032	273.9994782	-2.01547169	0.424601	-4.74674	2.07E-06	1.36E-05	down-regulated	protein_coding
C7H14ORF166	ENSMMUG00000018200	101.5623794	-6.051099218	1.274837	-4.74657	2.07E-06	1.36E-05	down-regulated	protein_coding
USP22	ENSMMUG00000005377	123.4865971	-2.332493342	0.491606	-4.74464	2.09E-06	1.37E-05	down-regulated	protein_coding
SUMO1	ENSMMUG00000005240	43.5547728	-5.813125012	1.225726	-4.7426	2.11E-06	1.39E-05	down-regulated	protein_coding
WDR88	ENSMMUG00000022871	7.74116656	7.486897641	1.57949	4.740073	2.14E-06	1.40E-05	up-regulated	protein_coding
SSR1	ENSMMUG00000002147	160.5418093	-1.67506077	0.353413	-4.73967	2.14E-06	1.40E-05	down-regulated	protein_coding
VAPB	ENSMMUG00000004986	151.1073188	-2.053710735	0.43331	-4.73958	2.14E-06	1.40E-05	down-regulated	protein_coding
IMPAD1	ENSMMUG00000014650	118.5121864	1.562092111	0.329599	4.73937	2.14E-06	1.40E-05	up-regulated	protein_coding
Y_RNA	ENSMMUG00000026406	13.03459848	5.41058023	1.141983	4.737882	2.16E-06	1.41E-05	up-regulated	misc_RNA
APOA5	ENSMMUG00000020288	18.40387991	3.355502337	0.708348	4.737084	2.17E-06	1.42E-05	up-regulated	protein_coding
ZC2HC1C	ENSMMUG00000015165	22.76501995	4.172798344	0.881846	4.731888	2.22E-06	1.45E-05	up-regulated	protein_coding
ARRDC4	ENSMMUG00000006488	138.7179282	-2.269574629	0.479723	-4.73101	2.23E-06	1.46E-05	down-regulated	protein_coding
TMBIM4	ENSMMUG00000016621	106.5981459	-2.306067362	0.487554	-4.72987	2.25E-06	1.47E-05	down-regulated	protein_coding
SLC35B3	ENSMMUG00000010672	44.10153325	-5.831692417	1.232977	-4.72976	2.25E-06	1.47E-05	down-regulated	protein_coding
TRIL	ENSMMUG00000020183	18.05347376	6.559674899	1.387028	4.729304	2.25E-06	1.47E-05	up-regulated	protein_coding
UCHL5	ENSMMUG00000022641	75.76750174	-4.152596868	0.878203	-4.72851	2.26E-06	1.47E-05	down-regulated	protein_coding
SLA2	ENSMMUG00000008200	10.71966383	5.566270516	1.177219	4.72832	2.26E-06	1.47E-05	up-regulated	protein_coding
PMF1-BGLAP	ENSMMUG00000002620	78.43210864	3.771119623	0.797581	4.728199	2.27E-06	1.47E-05	up-regulated	protein_coding
KAAG1	ENSMMUG00000023131	25.18853651	6.817077878	1.44187	4.727941	2.27E-06	1.48E-05	up-regulated	protein_coding
MS4A15	ENSMMUG00000000017	37.20350991	3.988005129	0.843603	4.727349	2.27E-06	1.48E-05	up-regulated	protein_coding
RNF149	ENSMMUG00000001149	96.89703775	-3.181390476	0.673037	-4.72692	2.28E-06	1.48E-05	down-regulated	protein_coding
SNORA70	ENSMMUG00000025468	7.532673869	5.47724797	1.158726	4.726956	2.28E-06	1.48E-05	up-regulated	snoRNA
C14H11ORF58	ENSMMUG00000017626	83.23625637	-4.318514126	0.913935	-4.72519	2.30E-06	1.49E-05	down-regulated	protein_coding
NDUFS8	ENSMMUG00000023189	153.9090024	-3.306839133	0.699863	-4.72498	2.30E-06	1.49E-05	down-regulated	protein_coding
TCEAL8	ENSMMUG00000004152	115.4992582	-2.183282979	0.462208	-4.72359	2.32E-06	1.50E-05	down-regulated	protein_coding
VDAC1	ENSMMUG00000016235	588.8086039	-1.523395495	0.322545	-4.72304	2.32E-06	1.51E-05	down-regulated	protein_coding
CERK	ENSMMUG00000020950	316.1318432	1.889711611	0.400284	4.720932	2.35E-06	1.52E-05	up-regulated	protein_coding

DHRS11	ENSMMUG00000000620	472.9967784	-1.577847749	0.334519	-4.71676	2.40E-06	1.55E-05	down-regulated	protein_coding
Y_RNA	ENSMMUG00000027714	10.76456894	7.185900187	1.524607	4.71328	2.44E-06	1.58E-05	up-regulated	misc_RNA
STRADB	ENSMMUG00000018141	165.0637435	-2.39729869	0.508651	-4.71306	2.44E-06	1.58E-05	down-regulated	protein_coding
PERP	ENSMMUG00000000997	576.9343758	-1.893954508	0.402017	-4.71113	2.46E-06	1.59E-05	down-regulated	protein_coding
PDCD6	ENSMMUG00000011933	99.28845305	-2.620586004	0.556277	-4.71094	2.47E-06	1.59E-05	down-regulated	protein_coding
PRMT2	ENSMMUG00000015803	86.87973676	-4.355759973	0.925612	-4.70582	2.53E-06	1.63E-05	down-regulated	protein_coding
RNF128	ENSMMUG00000021412	189.260666	-3.324594958	0.706636	-4.70482	2.54E-06	1.64E-05	down-regulated	protein_coding
RNF126	ENSMMUG00000008672	177.6201007	-2.463506766	0.523991	-4.70143	2.58E-06	1.66E-05	down-regulated	protein_coding
DAPK3	ENSMMUG00000022765	142.6435136	-2.489024882	0.529507	-4.70065	2.59E-06	1.67E-05	down-regulated	protein_coding
RAB5C	ENSMMUG00000014761	252.8535864	-1.787434798	0.380329	-4.69971	2.61E-06	1.68E-05	down-regulated	protein_coding
mml-mir-1250	ENSMMUG00000033887	7.128788505	5.509985385	1.172493	4.699376	2.61E-06	1.68E-05	up-regulated	miRNA
PDCL	ENSMMUG00000006896	75.90192624	2.103068707	0.447564	4.698923	2.62E-06	1.68E-05	up-regulated	protein_coding
FZD5	ENSMMUG00000013656	116.1704673	1.950525422	0.415295	4.696727	2.64E-06	1.70E-05	up-regulated	protein_coding
U6	ENSMMUG00000027501	9.632487692	6.77710379	1.44355	4.694747	2.67E-06	1.71E-05	up-regulated	snRNA
NDUFB5	ENSMMUG00000009746	87.49655262	-4.979849378	1.060827	-4.69431	2.68E-06	1.72E-05	down-regulated	protein_coding
HCFC1	ENSMMUG00000000319	160.879824	1.50873932	0.321481	4.693097	2.69E-06	1.72E-05	up-regulated	protein_coding
CCNK	ENSMMUG00000001201	126.1066706	1.637830722	0.348987	4.693098	2.69E-06	1.72E-05	up-regulated	protein_coding
ACTL6A	ENSMMUG00000009742	155.0573483	2.150400626	0.458208	4.693067	2.69E-06	1.72E-05	up-regulated	protein_coding
SELT	ENSMMUG00000017699	82.82578751	-5.764887103	1.229011	-4.69067	2.72E-06	1.74E-05	down-regulated	protein_coding
NPAS1	ENSMMUG00000011330	13.21897209	3.358507986	0.71665	4.686401	2.78E-06	1.78E-05	up-regulated	protein_coding
HADHB	ENSMMUG00000004381	145.7647878	-3.34788676	0.71466	-4.68458	2.81E-06	1.79E-05	down-regulated	protein_coding
CAPG	ENSMMUG00000002675	146.1448161	-2.950026599	0.629779	-4.68423	2.81E-06	1.80E-05	down-regulated	protein_coding
CEBPZ	ENSMMUG00000016433	141.1702249	1.451173256	0.309884	4.682959	2.83E-06	1.81E-05	up-regulated	protein_coding
CEP63	ENSMMUG00000012752	73.00753279	2.120297586	0.452942	4.681164	2.85E-06	1.82E-05	up-regulated	protein_coding
SIRT7	ENSMMUG00000012890	87.19330003	-3.363139588	0.718597	-4.68015	2.87E-06	1.83E-05	down-regulated	protein_coding
HIBADH	ENSMMUG00000023134	76.35545717	-4.182838459	0.894042	-4.67857	2.89E-06	1.84E-05	down-regulated	protein_coding
AHRR	ENSMMUG00000000752	14.26392731	5.497981535	1.175536	4.677001	2.91E-06	1.86E-05	up-regulated	protein_coding
CDH2	ENSMMUG00000015178	8.874090214	6.618044115	1.415012	4.677024	2.91E-06	1.86E-05	up-regulated	protein_coding
snoU13	ENSMMUG00000035880	11.41858001	7.495274482	1.603224	4.675127	2.94E-06	1.87E-05	up-regulated	snoRNA
PKD1L3	ENSMMUG00000016717	48.15639706	5.508111686	1.178738	4.67289	2.97E-06	1.89E-05	up-regulated	protein_coding
VCP	ENSMMUG00000020190	602.8302373	-1.352589584	0.289493	-4.67228	2.98E-06	1.89E-05	down-regulated	protein_coding
TMCO1	ENSMMUG00000005585	87.20030726	-2.855494268	0.61163	-4.66866	3.03E-06	1.93E-05	down-regulated	protein_coding
HMGN1	ENSMMUG00000013547	44.95592282	-5.85856169	1.255382	-4.66676	3.06E-06	1.94E-05	down-regulated	protein_coding
NRAP	ENSMMUG00000001208	7.892053542	7.4837957	1.604705	4.663658	3.11E-06	1.97E-05	up-regulated	protein_coding
RAN	ENSMMUG00000020927	221.5516375	-3.702374043	0.79401	-4.66288	3.12E-06	1.98E-05	down-regulated	protein_coding
GRSF1	ENSMMUG00000000380	84.21383275	-4.923192649	1.055873	-4.66268	3.12E-06	1.98E-05	down-regulated	protein_coding
SAMD8	ENSMMUG00000008690	92.47901853	3.441794052	0.73862	4.659765	3.17E-06	2.01E-05	up-regulated	protein_coding
TMPRSS2	ENSMMUG00000015332	584.8487765	-2.321235449	0.498396	-4.65741	3.20E-06	2.03E-05	down-regulated	protein_coding
MCF2L2	ENSMMUG00000001456	16.68894847	3.801004151	0.816396	4.655835	3.23E-06	2.04E-05	up-regulated	protein_coding
PFN2	ENSMMUG00000023337	201.8987828	-3.053414078	0.656061	-4.65416	3.25E-06	2.06E-05	down-regulated	protein_coding
PRR15L	ENSMMUG00000003324	321.4499202	-1.55122307	0.333337	-4.65361	3.26E-06	2.06E-05	down-regulated	protein_coding
EMC1	ENSMMUG00000006688	208.4779896	1.138825917	0.244744	4.653136	3.27E-06	2.06E-05	up-regulated	protein_coding
WSB2	ENSMMUG00000010236	296.07982	-1.816975419	0.390502	-4.65292	3.27E-06	2.07E-05	down-regulated	protein_coding
STK38L	ENSMMUG00000023248	95.87807043	-3.043642054	0.654178	-4.65262	3.28E-06	2.07E-05	down-regulated	protein_coding
MCOLN3	ENSMMUG00000005361	31.41456048	5.07332559	1.091412	4.648406	3.35E-06	2.11E-05	up-regulated	protein_coding
VIMP	ENSMMUG00000011823	129.1745532	-2.663174966	0.573092	-4.64703	3.37E-06	2.12E-05	down-regulated	protein_coding
DYNC1I2	ENSMMUG00000007301	132.1386894	-1.962158925	0.422362	-4.64568	3.39E-06	2.13E-05	down-regulated	protein_coding
ZNF711	ENSMMUG00000013603	56.09812147	2.873259211	0.619082	4.641159	3.46E-06	2.18E-05	up-regulated	protein_coding
FAM109A	ENSMMUG00000031550	43.22952547	-5.803738835	1.25079	-4.64006	3.48E-06	2.19E-05	down-regulated	protein_coding
NEU1	ENSMMUG00000021707	373.8869233	-2.614656473	0.563535	-4.63974	3.49E-06	2.19E-05	down-regulated	protein_coding
ENTPD6	ENSMMUG00000011874	89.6363832	-3.596138411	0.77516	-4.63922	3.50E-06	2.20E-05	down-regulated	protein_coding
TPMT	ENSMMUG00000016627	79.43619206	-4.819969929	1.039109	-4.63856	3.51E-06	2.20E-05	down-regulated	protein_coding
SPEF2	ENSMMUG00000018739	16.66720473	5.3683808	1.157369	4.638435	3.51E-06	2.20E-05	up-regulated	protein_coding
NCAM1	ENSMMUG00000004688	6.203969189	5.45875046	1.177147	4.637272	3.53E-06	2.22E-05	up-regulated	protein_coding
TUBGCP2	ENSMMUG00000023034	97.71343465	-3.5482932	0.765256	-4.63674	3.54E-06	2.22E-05	down-regulated	protein_coding
PAIP2	ENSMMUG00000005970	76.62408187	-3.436757317	0.741268	-4.63632	3.55E-06	2.22E-05	down-regulated	protein_coding
5S_rRNA	ENSMMUG00000026485	6.021334511	5.700647393	1.229626	4.636084	3.55E-06	2.22E-05	up-regulated	rRNA
ABCA9	ENSMMUG00000018592	17.56586092	6.845118248	1.476812	4.635064	3.57E-06	2.23E-05	up-regulated	protein_coding
SOX10	ENSMMUG00000006986	8.01912961	5.333114871	1.150979	4.633545	3.59E-06	2.25E-05	up-regulated	protein_coding
FOXP2	ENSMMUG00000008005	9.603053082	4.381357882	0.945751	4.632674	3.61E-06	2.26E-05	up-regulated	protein_coding
PPP1R1C	ENSMMUG00000001171	9.990746825	5.238772281	1.131062	4.631727	3.63E-06	2.27E-05	up-regulated	protein_coding
MDH1B	ENSMMUG00000017734	9.451729581	4.395805624	0.949236	4.630889	3.64E-06	2.28E-05	up-regulated	protein_coding
C1ORF112	ENSMMUG00000016582	66.41064965	3.321654324	0.717317	4.630664	3.64E-06	2.28E-05	up-regulated	protein_coding
NAT9	ENSMMUG00000005738	97.29389355	2.530445817	0.546574	4.629647	3.66E-06	2.29E-05	up-regulated	protein_coding
FUS	ENSMMUG00000019637	217.4136177	-3.341142077	0.722312	-4.62562	3.73E-06	2.33E-05	down-regulated	protein_coding
ACO1	ENSMMUG00000008196	234.7301967	-1.541665154	0.333448	-4.62341	3.77E-06	2.35E-05	down-regulated	protein_coding
KIAA1671	ENSMMUG00000005834	116.7046433	2.901021062	0.62839	4.616594	3.90E-06	2.43E-05	up-regulated	protein_coding
KCNN2	ENSMMUG00000020502	9.092384631	8.394135592	1.818943	4.614844	3.93E-06	2.45E-05	up-regulated	protein_coding
FNDC3A	ENSMMUG00000023756	194.2547073	1.852529814	0.401426	4.614873	3.93E-06	2.45E-05	up-regulated	protein_coding
PDIA2	ENSMMUG00000000604	26.40681038	4.111820672	0.891498	4.612261	3.98E-06	2.47E-05	up-regulated	protein_coding
CD59	ENSMMUG00000006062	664.7678477	-1.811843846	0.39287	-4.61181	3.99E-06	2.48E-05	down-regulated	protein_coding
CACNA1S	ENSMMUG00000017414	9.965618198	9.243048454	2.004451	4.611261	4.00E-06	2.48E-05	up-regulated	protein_coding
HIST1H2AH	ENSMMUG00000001438	25.09509736	6.918361125	1.500406	4.610993	4.01E-06	2.48E-05	up-regulated	protein_coding
PRPSAP1	ENSMMUG00000003543	77.37234149	-2.939584452	0.637603	-4.61036	4.02E-06	2.49E-05	down-regulated	protein_coding
GHRH	ENSMMUG00000031295	6.135865514	6.067717811	1.316112	4.610335	4.02E-06	2.49E-05	up-regulated	protein_coding
ETFB	ENSMMUG00000023531	154.2528286	-2.441106026	0.529607	-4.60928	4.04E-06	2.50E-05	down-regulated	protein_coding
5S_rRNA	ENSMMUG00000026104	7.277168248	6.242038233	1.354302	4.609046	4.05E-06	2.50E-05	up-regulated	rRNA
ABRACL	ENSMMUG00000022821	229.1363292	1.665676108	0.361527	4.607335	4.08E-06	2.52E-05	up-regulated	protein_coding
DDIT3	ENSMMUG00000011286	85.80596279	2.177817617	0.472953	4.604727	4.13E-06	2.55E-05	up-regulated	protein_coding
PDLIM2	ENSMMUG00000018520	168.4177346	-3.20528399	0.696115	-4.60453	4.13E-06	2.56E-05	down-regulated	protein_coding
STKLD1	ENSMMUG00000000934	19.7355545	5.583977631	1.213785	4.600465	4.22E-06	2.60E-05	up-regulated	protein_coding
JOSD1	ENSMMUG00000021083	381.0154673	-1.659666726	0.360838	-4.59948	4.24E-06	2.61E-05	down-regulated	protein_coding
ATHL1	ENSMMUG00000004625	91.15180206	-5.903603906	1.283628	-4.59915	4.24E-06	2.62E-05	down-regulated	protein_coding
SNX9	ENSMMUG00000007142	156.4067162	-2.751219389	0.598676	-4.59551	4.32E-06	2.66E-05	down-regulated	protein_coding
WDR5B	ENSMMUG00000016378	23.26850537	3.460190872	0.75298	4.595331	4.32E-06	2.66E-05	up-regulated	protein_coding
EWSR1	ENSMMUG00000021374	355.8662724	-2.286815905	0.497855	-4.59334	4.36E-06	2.69E-05	down-regulated	protein_coding
H2AFV	ENSMMUG00000000962	103.8163575	-6.083256423	1.325454	-4.58956	4.44E-06	2.73E-05	down-regulated	protein_coding
ZNF79	ENSMMUG00000021760	30.68351358	2.894484181	0.630719	4.589184	4.45E-06	2.74E-05	up-regulated	protein_coding
KCNH1	ENSMMUG00000016102	8.230227995	6.931450802	1.510659	4.588361	4.47E-06	2.75E-05	up-regulated	protein_coding
MIF4GD	ENSMMUG00000018023	138.3335605	1.800983881	0.392616	4.587139	4.49E-06	2.76E-05	up-regulated	protein_coding
TST	ENSMMUG00000012349	169.1376535	-2.299136623	0.501403	-4.58541	4.53E-06	2.78E-05	down-regulated	protein_coding
U1	ENSMMUG00000028170	13.54167656	5.908777499	1.288594	4.585444	4.53E-06	2.78E-05	up-regulated	snRNA
DEDD	ENSMMUG00000002010	115.8662682	1.540590457	0.336006	4.585004	4.54E-06	2.79E-05	up-regulated	protein_coding
DAZAP2	ENSMMUG00000001884	248.6976896	-1.764573391	0.385067	-4.58251	4.59E-06	2.82E-05	down-regulated	protein_coding
PITRM1	ENSMMUG00000004938	305.3236145	1.609720694	0.351356	4.581452	4.62E-06	2.83E-05	up-regulated	protein_coding
PINK1	ENSMMUG00000000126	79.81270724	-4.26540677	0.931366	-4.57973	4.66E-06	2.85E-05	down-regulated	protein_coding
HPCAL1	ENSMMUG00000020440	265.5023663	1.695209202	0.370178	4.579439	4.66E-06	2.86E-05	up-regulated	protein_coding
CBX6	ENSMMUG00000004870	33.18029282	4.614050742	1.007867	4.578036	4.69E-06	2.87E-05	up-regulated	protein_coding
C8H8ORF82	ENSMMUG00000015595	257.0047934	1.837055939	0.401296	4.577813	4.70E-06	2.88E-05	up-regulated	protein_coding
ST5	ENSMMUG00000021830	199.7887153	2.122882496	0.463933	4.575841	4.74E-06	2.90E-05	up-regulated	protein_coding

ORMDL3	ENSMMUG00000004586	85.88161906	-3.011567345	0.658257	-4.57506	4.76E-06	2.91E-05	down-regulated	protein_coding
SNX6	ENSMMUG00000012576	76.34245626	-3.352188664	0.732715	-4.57503	4.76E-06	2.91E-05	down-regulated	protein_coding
MAMDC2	ENSMMUG00000008343	8.11717024	5.751860706	1.257256	4.574933	4.76E-06	2.91E-05	up-regulated	protein_coding
GPR3	ENSMMUG00000018325	15.71487048	3.581337847	0.783026	4.573718	4.79E-06	2.93E-05	up-regulated	protein_coding
OTC	ENSMMUG00000013493	125.3227099	-3.965302557	0.867029	-4.57344	4.80E-06	2.93E-05	down-regulated	protein_coding
PCK2	ENSMMUG00000021590	401.9989532	-2.353912463	0.514967	-4.57099	4.85E-06	2.96E-05	down-regulated	protein_coding
TUBA1B	ENSMMUG00000021081	916.4139386	-3.100002545	0.678452	-4.56923	4.90E-06	2.99E-05	down-regulated	protein_coding
ADH4	ENSMMUG00000011059	16.78398259	4.740718944	1.037785	4.568115	4.92E-06	3.00E-05	up-regulated	protein_coding
APOD	ENSMMUG00000031415	7.69141778	4.998842399	1.094535	4.567093	4.95E-06	3.01E-05	up-regulated	protein_coding
CINP	ENSMMUG00000003483	58.90246279	-6.248537806	1.368237	-4.56685	4.95E-06	3.02E-05	down-regulated	protein_coding
FBXL22	ENSMMUG00000012116	7.875377411	6.533601265	1.431334	4.564695	5.00E-06	3.04E-05	up-regulated	protein_coding
CD99	ENSMMUG00000018687	153.2771363	-2.582461667	0.56574	-4.56475	5.00E-06	3.04E-05	down-regulated	protein_coding
FCAMR	ENSMMUG00000011221	17.85302153	3.577634092	0.783845	4.564209	5.01E-06	3.05E-05	up-regulated	protein_coding
CMTM3	ENSMMUG00000020847	45.42336729	2.672840056	0.585667	4.563756	5.02E-06	3.06E-05	up-regulated	protein_coding
RBX1	ENSMMUG00000012642	51.04310168	-6.041840144	1.325299	-4.55885	5.14E-06	3.13E-05	down-regulated	protein_coding
ZDHHC6	ENSMMUG00000001157	86.81710403	-2.656008853	0.582695	-4.55814	5.16E-06	3.13E-05	down-regulated	protein_coding
EGLN2	ENSMMUG00000001197	104.7980155	-2.575367358	0.565053	-4.55775	5.17E-06	3.14E-05	down-regulated	protein_coding
ATP6V1D	ENSMMUG00000021418	84.57995733	-2.946348611	0.646752	-4.55561	5.22E-06	3.17E-05	down-regulated	protein_coding
MANEAL	ENSMMUG00000022040	13.95996162	4.181556496	0.918331	4.553431	5.28E-06	3.20E-05	up-regulated	protein_coding
TSPAN7	ENSMMUG00000006876	97.97522987	-5.160901091	1.134332	-4.54973	5.37E-06	3.25E-05	down-regulated	protein_coding
GATA6	ENSMMUG00000010944	151.1853212	-1.854803141	0.407761	-4.54875	5.40E-06	3.26E-05	down-regulated	protein_coding
NSDHL	ENSMMUG00000013176	188.6896348	-2.319495891	0.510003	-4.548	5.42E-06	3.27E-05	down-regulated	protein_coding
CACNA2D4	ENSMMUG00000017265	30.6989776	3.154245175	0.693637	4.547404	5.43E-06	3.28E-05	up-regulated	protein_coding
TAF9	ENSMMUG00000009140	80.88951414	-4.272874313	0.939783	-4.54666	5.45E-06	3.29E-05	down-regulated	protein_coding
MFSD2B	ENSMMUG00000012337	11.99942373	7.025804483	1.545378	4.546335	5.46E-06	3.30E-05	up-regulated	protein_coding
HERC5	ENSMMUG00000010316	15.84132708	4.753444546	1.045952	4.544612	5.50E-06	3.32E-05	up-regulated	protein_coding
HIC1	ENSMMUG00000030393	13.40032346	5.287424138	1.164337	4.541146	5.59E-06	3.38E-05	up-regulated	protein_coding
SLC30A9	ENSMMUG00000022782	42.96394511	-5.794904813	1.276295	-4.54041	5.61E-06	3.39E-05	down-regulated	protein_coding
SNORD91	ENSMMUG00000032751	15.54344615	3.640912098	0.801954	4.54005	5.62E-06	3.39E-05	up-regulated	snoRNA
FAM108A1	ENSMMUG00000016539	286.7330844	-2.732351034	0.60198	-4.53894	5.65E-06	3.41E-05	down-regulated	protein_coding
SCN8A	ENSMMUG00000000415	11.83486552	5.869010102	1.293088	4.538755	5.66E-06	3.41E-05	up-regulated	protein_coding
TMC1	ENSMMUG00000012503	18.81000342	6.875735243	1.517122	4.532091	5.84E-06	3.52E-05	up-regulated	protein_coding
CCDC109A	ENSMMUG00000020402	47.97119574	-5.953998869	1.313919	-4.53148	5.86E-06	3.53E-05	down-regulated	protein_coding
NDUFAF3	ENSMMUG00000005654	86.43743644	-5.8261731	1.285773	-4.53126	5.86E-06	3.53E-05	down-regulated	protein_coding
ABI1	ENSMMUG00000005078	102.9381031	-2.431448335	0.537044	-4.52747	5.97E-06	3.59E-05	down-regulated	protein_coding
C5orf30	ENSMMUG00000004211	77.53944607	-2.708929125	0.598415	-4.52684	5.99E-06	3.60E-05	down-regulated	protein_coding
CCDC106	ENSMMUG00000004808	38.83783042	3.477275729	0.768154	4.526797	5.99E-06	3.60E-05	up-regulated	protein_coding
RAB25	ENSMMUG00000022306	174.9158596	-2.783818694	0.615324	-4.52415	6.06E-06	3.64E-05	down-regulated	protein_coding
DLD	ENSMMUG00000006564	142.5572531	-1.935773117	0.427979	-4.52305	6.10E-06	3.66E-05	down-regulated	protein_coding
RLBP1	ENSMMUG00000014420	9.493058323	6.609563051	1.461504	4.52244	6.11E-06	3.67E-05	up-regulated	protein_coding
SNCG	ENSMMUG00000011849	7.772145097	6.920648608	1.530629	4.52144	6.14E-06	3.68E-05	up-regulated	protein_coding
DDI1	ENSMMUG00000005354	9.058486486	9.10067596	2.013656	4.519479	6.20E-06	3.72E-05	up-regulated	protein_coding
SLC7A6	ENSMMUG00000002064	18.03730803	3.506290265	0.775945	4.518737	6.22E-06	3.72E-05	up-regulated	protein_coding
ACSS2	ENSMMUG00000017932	517.3317442	-2.396095181	0.530363	-4.51784	6.25E-06	3.74E-05	down-regulated	protein_coding
GPR173	ENSMMUG00000002592	9.052759446	9.106684672	2.016029	4.517139	6.27E-06	3.75E-05	up-regulated	protein_coding
ALX1	ENSMMUG00000016536	9.052759446	9.106684672	2.016029	4.517139	6.27E-06	3.75E-05	up-regulated	protein_coding
ROS1	ENSMMUG00000023738	9.052759446	9.106684672	2.016029	4.517139	6.27E-06	3.75E-05	up-regulated	protein_coding
ATL1	ENSMMUG00000009577	17.88898048	5.245914497	1.161485	4.516559	6.29E-06	3.76E-05	up-regulated	protein_coding
TAF6L	ENSMMUG00000000110	95.16517269	2.554401468	0.565605	4.516232	6.29E-06	3.76E-05	up-regulated	protein_coding
STK10	ENSMMUG00000006811	68.39435443	1.896596517	0.419952	4.516222	6.30E-06	3.76E-05	up-regulated	protein_coding
HIST1H3A	ENSMMUG00000022053	8.179525879	8.244329808	1.825683	4.51575	6.31E-06	3.77E-05	up-regulated	protein_coding
SMC1B	ENSMMUG00000023004	10.34580341	7.754488159	1.717442	4.515138	6.33E-06	3.77E-05	up-regulated	protein_coding
EEF1G	ENSMMUG00000006598	1426.00192	-3.398411787	0.752836	-4.51415	6.36E-06	3.79E-05	down-regulated	protein_coding
HIST1H3A	ENSMMUG00000029920	33.0515556	5.331595081	1.18156	4.512334	6.41E-06	3.82E-05	up-regulated	protein_coding
KIAA0195	ENSMMUG00000022120	184.8649623	1.134646501	0.251666	4.508532	6.53E-06	3.89E-05	up-regulated	protein_coding
NUDT16	ENSMMUG00000001154	43.48584224	-5.81039895	1.289711	-4.50519	6.63E-06	3.95E-05	down-regulated	protein_coding
SLC27A2	ENSMMUG00000002876	153.8910012	-1.730574483	0.384134	-4.50513	6.63E-06	3.95E-05	down-regulated	protein_coding
AMER1	ENSMMUG00000004309	14.13293655	3.234773569	0.718257	4.503645	6.68E-06	3.97E-05	up-regulated	protein_coding
WNT4	ENSMMUG00000006915	15.56999359	3.943555069	0.875641	4.503621	6.68E-06	3.97E-05	up-regulated	protein_coding
RNPS1	ENSMMUG00000019238	175.9035755	-2.442126856	0.542283	-4.50342	6.69E-06	3.98E-05	down-regulated	protein_coding
KCTD3	ENSMMUG00000022639	193.880989	-2.113810449	0.469412	-4.50311	6.70E-06	3.98E-05	down-regulated	protein_coding
ALX3	ENSMMUG00000031857	13.94593653	6.240245644	1.385851	4.502825	6.71E-06	3.98E-05	up-regulated	protein_coding
TFDP2	ENSMMUG00000011134	38.64580982	-5.641884668	1.253543	-4.50075	6.77E-06	4.02E-05	down-regulated	protein_coding
TNFRSF19	ENSMMUG00000004237	9.214827065	4.49053871	0.998013	4.499479	6.81E-06	4.04E-05	up-regulated	protein_coding
SUSD1	ENSMMUG00000021097	123.3593692	2.508393533	0.557606	4.498507	6.84E-06	4.06E-05	up-regulated	protein_coding
RPL26	ENSMMUG00000003448	646.732908	-2.403263123	0.534247	-4.49842	6.85E-06	4.06E-05	down-regulated	protein_coding
THBS2	ENSMMUG00000023631	33.99183746	2.74255647	0.609942	4.496425	6.91E-06	4.09E-05	up-regulated	protein_coding
NAA60	ENSMMUG00000018711	39.91099906	-5.688613206	1.265697	-4.49445	6.98E-06	4.13E-05	down-regulated	protein_coding
ZNF589	ENSMMUG00000021607	27.53498341	2.303539837	0.512573	4.494069	6.99E-06	4.14E-05	up-regulated	protein_coding
TLCD2	ENSMMUG00000002378	13.65819309	4.023539518	0.895379	4.493673	7.00E-06	4.14E-05	up-regulated	protein_coding
HERPUD1	ENSMMUG00000019189	72.99220209	-4.082906717	0.908813	-4.49257	7.04E-06	4.16E-05	down-regulated	protein_coding
DPP7	ENSMMUG00000022750	44.89560494	-5.856778779	1.303772	-4.49218	7.05E-06	4.16E-05	down-regulated	protein_coding
ARMCX1	ENSMMUG00000023604	35.72908853	-5.528371376	1.231489	-4.48918	7.15E-06	4.22E-05	down-regulated	protein_coding
LXN	ENSMMUG00000004963	69.70958467	1.894315375	0.422051	4.488356	7.18E-06	4.23E-05	up-regulated	protein_coding
B3GNT3	ENSMMUG00000012280	87.0967967	-3.924067048	0.874465	-4.48739	7.21E-06	4.25E-05	down-regulated	protein_coding
NQO1	ENSMMUG00000019684	359.0203554	-2.2522216	0.501895	-4.48743	7.21E-06	4.25E-05	down-regulated	protein_coding
ND1	ENSMMUG00000028699	14609.04606	7.148751748	1.593406	4.48646	7.24E-06	4.27E-05	up-regulated	protein_coding
PFDN5	ENSMMUG00000022030	98.95215918	-5.176826951	1.153946	-4.4862	7.25E-06	4.27E-05	down-regulated	protein_coding
XG	ENSMMUG00000018688	12.61134465	7.311522957	1.62996	4.485706	7.27E-06	4.28E-05	up-regulated	protein_coding
SARM1	ENSMMUG00000019150	19.17446046	4.02317719	0.896933	4.48548	7.27E-06	4.28E-05	up-regulated	protein_coding
TMED10	ENSMMUG00000015168	130.0022669	-2.015097852	0.4493	-4.48497	7.29E-06	4.29E-05	down-regulated	protein_coding
MKKS	ENSMMUG00000031166	74.40915316	-5.607551705	1.250539	-4.48411	7.32E-06	4.31E-05	down-regulated	protein_coding
ZC3H15	ENSMMUG00000017248	67.02883657	-5.454083111	1.216517	-4.48336	7.35E-06	4.32E-05	down-regulated	protein_coding
ORAI1	ENSMMUG00000001306	91.37895298	-5.907463866	1.318047	-4.48198	7.40E-06	4.34E-05	down-regulated	protein_coding
NDFIP1	ENSMMUG00000016787	240.7425029	-1.78053906	0.397297	-4.48163	7.41E-06	4.35E-05	down-regulated	protein_coding
COTL1	ENSMMUG00000007039	1062.217428	-1.775004593	0.396072	-4.48152	7.41E-06	4.35E-05	down-regulated	protein_coding
FRMD5	ENSMMUG00000022777	15.63671949	3.572361954	0.797191	4.481185	7.42E-06	4.35E-05	up-regulated	protein_coding
SOD1	ENSMMUG00000001711	417.435548	-2.054001188	0.458402	-4.48079	7.44E-06	4.36E-05	down-regulated	protein_coding
SHROOM4	ENSMMUG00000000378	9.539865737	7.047993559	1.573579	4.478957	7.50E-06	4.39E-05	up-regulated	protein_coding
SPTLC3	ENSMMUG00000000089	18.19076876	5.09688113	1.138089	4.478455	7.52E-06	4.40E-05	up-regulated	protein_coding
ADD3	ENSMMUG00000006233	117.1670997	-3.107787797	0.693999	-4.47808	7.53E-06	4.41E-05	down-regulated	protein_coding
CETN2	ENSMMUG00000020743	77.80588172	-4.189590118	0.935653	-4.47772	7.54E-06	4.41E-05	down-regulated	protein_coding
DIXDC1	ENSMMUG00000017565	25.15415587	3.106501772	0.694591	4.472421	7.73E-06	4.52E-05	up-regulated	protein_coding
FAM92A1	ENSMMUG00000005557	27.37865981	2.300216189	0.514445	4.471259	7.78E-06	4.55E-05	up-regulated	protein_coding
CHMP1A	ENSMMUG00000014909	75.72139942	-3.274244558	0.732308	-4.47113	7.78E-06	4.55E-05	down-regulated	protein_coding
PAOX	ENSMMUG00000006326	122.8601984	-2.575975069	0.576259	-4.47017	7.82E-06	4.56E-05	down-regulated	protein_coding
PSMD2	ENSMMUG00000000800	389.4881578	-1.748921662	0.391377	-4.46864	7.87E-06	4.59E-05	down-regulated	protein_coding
ZNF334	ENSMMUG00000007317	25.35586375	2.37544275	0.531592	4.468549	7.88E-06	4.59E-05	up-regulated	protein_coding
VDAC3	ENSMMUG00000020185	115.6117498	-3.019052129	0.675742	-4.46776	7.90E-06	4.61E-05	down-regulated	protein_coding
NDUFA1	ENSMMUG00000032359	37.68012472	-5.603959996	1.254477	-4.46717	7.93E-06	4.62E-05	down-regulated	protein_coding

F10	ENSMMUG00000001038	25.79279949	3.999983214	0.895559	4.466465	7.95E-06	4.63E-05	up-regulated	protein_coding
SPRED1	ENSMMUG00000010704	152.3497477	2.375972024	0.532349	4.463182	8.08E-06	4.70E-05	up-regulated	protein_coding
MED1	ENSMMUG00000009132	119.4814641	2.00055857	0.448553	4.460032	8.19E-06	4.77E-05	up-regulated	protein_coding
SEPT8	ENSMMUG00000010309	321.638433	1.146655405	0.257161	4.458895	8.24E-06	4.79E-05	up-regulated	protein_coding
CGGBP1	ENSMMUG00000005239	81.96102537	1.498535297	0.336132	4.458178	8.27E-06	4.81E-05	up-regulated	protein_coding
LANCL1	ENSMMUG00000010520	36.18868355	-5.547233925	1.244337	-4.45798	8.27E-06	4.81E-05	down-regulated	protein_coding
EIF4A1	ENSMMUG00000013499	717.6964162	-2.231333019	0.5006	-4.45731	8.30E-06	4.82E-05	down-regulated	protein_coding
SOLH	ENSMMUG00000000615	293.0694463	-1.92227442	0.431447	-4.45541	8.37E-06	4.86E-05	down-regulated	protein_coding
PDHB	ENSMMUG00000015672	91.81098382	-2.588255884	0.580929	-4.45537	8.37E-06	4.86E-05	down-regulated	protein_coding
TMEM154	ENSMMUG00000008262	36.30139026	-5.550771147	1.246044	-4.45472	8.40E-06	4.88E-05	down-regulated	protein_coding
C7orf50	ENSMMUG00000007206	140.9828378	-2.736597697	0.61481	-4.45113	8.54E-06	4.96E-05	down-regulated	protein_coding
HSD17B10	ENSMMUG00000009296	114.1660755	-3.785347742	0.850769	-4.44932	8.61E-06	5.00E-05	down-regulated	protein_coding
ODF3	ENSMMUG00000011502	6.251556828	5.081941554	1.142293	4.448893	8.63E-06	5.00E-05	up-regulated	protein_coding
OR9Q2	ENSMMUG00000005166	12.55484998	8.082137979	1.816788	4.448586	8.64E-06	5.01E-05	up-regulated	protein_coding
KHNYN	ENSMMUG00000009516	114.400421	-2.660202024	0.597999	-4.44851	8.65E-06	5.01E-05	down-regulated	protein_coding
KRI1	ENSMMUG00000011951	90.09275332	2.23881218	0.5033	4.448263	8.66E-06	5.01E-05	up-regulated	protein_coding
AKAP9	ENSMMUG00000002364	440.3105181	1.941271186	0.436647	4.445857	8.75E-06	5.07E-05	up-regulated	protein_coding
SLC13A3	ENSMMUG00000016924	21.73108403	6.720430542	1.51446	4.43751	9.10E-06	5.26E-05	up-regulated	protein_coding
PAIP1	ENSMMUG00000011878	103.9742574	-2.047575829	0.461597	-4.43585	9.17E-06	5.30E-05	down-regulated	protein_coding
MTFR1	ENSMMUG00000011263	34.42552877	-5.475318165	1.234386	-4.43566	9.18E-06	5.30E-05	down-regulated	protein_coding
LDB3	ENSMMUG00000003608	17.59365443	4.296776249	0.968781	4.43524	9.20E-06	5.31E-05	up-regulated	protein_coding
GPER1	ENSMMUG00000007209	34.33548888	3.92981204	0.886692	4.431991	9.34E-06	5.39E-05	up-regulated	protein_coding
MICU2	ENSMMUG00000011252	71.23393159	-2.741795632	0.619072	-4.42888	9.47E-06	5.46E-05	down-regulated	protein_coding
FICD	ENSMMUG00000017123	61.38660002	3.756559926	0.848316	4.428257	9.50E-06	5.48E-05	up-regulated	protein_coding
CNOT7	ENSMMUG00000001873	87.72879689	-2.732576672	0.617396	-4.42597	9.60E-06	5.53E-05	down-regulated	protein_coding
PCGF2	ENSMMUG00000012360	48.09000481	2.176657589	0.4918	4.425904	9.60E-06	5.53E-05	up-regulated	protein_coding
MATN2	ENSMMUG00000020072	9.099782723	5.933002633	1.340857	4.424783	9.65E-06	5.56E-05	up-regulated	protein_coding
SPAG1	ENSMMUG00000004386	71.62349478	2.580458035	0.583216	4.424529	9.67E-06	5.56E-05	up-regulated	protein_coding
NF2	ENSMMUG00000010311	159.3150449	1.878368973	0.424582	4.424039	9.69E-06	5.57E-05	up-regulated	protein_coding
SLIT3	ENSMMUG00000017087	7.894592207	6.320872788	1.428771	4.423994	9.69E-06	5.57E-05	up-regulated	protein_coding
ARFIP1	ENSMMUG00000018490	117.9503426	-2.154699798	0.48715	-4.42308	9.73E-06	5.59E-05	down-regulated	protein_coding
GFPT1	ENSMMUG00000013797	163.1707381	-3.442609555	0.778353	-4.42294	9.74E-06	5.59E-05	down-regulated	protein_coding
TSPAN1	ENSMMUG00000016452	667.9504606	-1.420420166	0.321236	-4.42173	9.79E-06	5.62E-05	down-regulated	protein_coding
CFAP61	ENSMMUG00000011429	9.133897931	7.180249833	1.623987	4.421372	9.81E-06	5.63E-05	up-regulated	protein_coding
SLC11A2	ENSMMUG00000022961	733.5773138	-3.188674136	0.721813	-4.41759	9.98E-06	5.72E-05	down-regulated	protein_coding
SNRNP70	ENSMMUG00000004928	498.0382706	-1.875108926	0.424564	-4.41655	1.00E-05	5.75E-05	down-regulated	protein_coding
FAM83E	ENSMMUG00000022156	132.885543	-1.644157967	0.372378	-4.41529	1.01E-05	5.78E-05	down-regulated	protein_coding
FANCC	ENSMMUG00000008299	48.26709652	1.859557223	0.421186	4.415052	1.01E-05	5.78E-05	up-regulated	protein_coding
MCL1	ENSMMUG00000008221	539.4607988	-1.969850491	0.446226	-4.41447	1.01E-05	5.80E-05	down-regulated	protein_coding
SUMO3	ENSMMUG00000006760	73.98949195	-3.100289548	0.702545	-4.41294	1.02E-05	5.84E-05	down-regulated	protein_coding
GLMP	ENSMMUG00000000092	166.5182398	-1.904674504	0.431727	-4.41176	1.03E-05	5.87E-05	down-regulated	protein_coding
HN1L	ENSMMUG00000000979	121.3393152	-2.366651345	0.536553	-4.41084	1.03E-05	5.89E-05	down-regulated	protein_coding
TMEM120B	ENSMMUG00000031504	42.67321416	3.288149747	0.745809	4.408834	1.04E-05	5.94E-05	up-regulated	protein_coding
CCAR1	ENSMMUG00000016652	68.15358876	-5.464572954	1.240278	-4.40593	1.05E-05	6.02E-05	down-regulated	protein_coding
MMS19	ENSMMUG00000030510	116.5091089	2.13485245	0.484562	4.405734	1.05E-05	6.02E-05	up-regulated	protein_coding
CDK16	ENSMMUG00000014002	235.8671124	-1.186771087	0.269391	-4.40539	1.06E-05	6.03E-05	down-regulated	protein_coding
SPG21	ENSMMUG00000010949	129.2146549	-2.588994381	0.587785	-4.40466	1.06E-05	6.04E-05	down-regulated	protein_coding
snoU13	ENSMMUG00000036287	8.923787848	6.197065683	1.407316	4.403465	1.07E-05	6.08E-05	up-regulated	snoRNA
ETS2	ENSMMUG00000009063	343.6727874	-1.877815815	0.42651	-4.40274	1.07E-05	6.09E-05	down-regulated	protein_coding
CAPN2	ENSMMUG00000011142	419.4705635	-1.492903223	0.339111	-4.4024	1.07E-05	6.10E-05	down-regulated	protein_coding
SLC35C2	ENSMMUG00000018071	82.56437482	-3.050357315	0.692935	-4.40208	1.07E-05	6.11E-05	down-regulated	protein_coding
EGF	ENSMMUG00000010043	7.273005877	4.247194881	0.964899	4.401697	1.07E-05	6.11E-05	up-regulated	protein_coding
GPX2	ENSMMUG00000021987	2094.469588	-3.061728734	0.695579	-4.4017	1.07E-05	6.11E-05	down-regulated	protein_coding
TNFAIP8L2	ENSMMUG00000007897	24.99011862	5.081724376	1.154588	4.401333	1.08E-05	6.12E-05	up-regulated	protein_coding
POR	ENSMMUG00000020086	574.5298061	-1.534341531	0.348662	-4.40066	1.08E-05	6.14E-05	down-regulated	protein_coding
U6	ENSMMUG00000025859	11.72319948	6.813529427	1.548554	4.399929	1.08E-05	6.16E-05	up-regulated	snRNA
ATP5L	ENSMMUG00000017604	127.2863333	-2.80340069	0.637455	-4.3978	1.09E-05	6.21E-05	down-regulated	protein_coding
CREG1	ENSMMUG00000008161	42.40656538	-5.776000487	1.313562	-4.3972	1.10E-05	6.22E-05	down-regulated	protein_coding
AHSA1	ENSMMUG00000013554	82.85206813	-3.04995798	0.693752	-4.39632	1.10E-05	6.25E-05	down-regulated	protein_coding
DAGLB	ENSMMUG00000010292	88.36183545	2.650130992	0.602825	4.396188	1.10E-05	6.25E-05	up-regulated	protein_coding
JTB	ENSMMUG00000005010	122.5125033	-2.726459591	0.620289	-4.39547	1.11E-05	6.27E-05	down-regulated	protein_coding
RAB26	ENSMMUG00000002843	49.59756286	3.89023865	0.885329	4.394116	1.11E-05	6.30E-05	up-regulated	protein_coding
ARHGAP32	ENSMMUG00000002963	365.2104166	2.111403321	0.480705	4.392308	1.12E-05	6.35E-05	up-regulated	protein_coding
CD58	ENSMMUG00000008156	65.34497796	-3.427696797	0.780455	-4.39192	1.12E-05	6.36E-05	down-regulated	protein_coding
FOXJ3	ENSMMUG00000014138	96.59789903	1.986905113	0.452423	4.3917	1.12E-05	6.37E-05	up-regulated	protein_coding
FAM65C	ENSMMUG00000007883	9.693343087	5.631270132	1.282526	4.390765	1.13E-05	6.39E-05	up-regulated	protein_coding
SLC31A1	ENSMMUG00000011197	116.8847371	-2.348810545	0.534968	-4.39056	1.13E-05	6.39E-05	down-regulated	protein_coding
TMEM50A	ENSMMUG00000032217	91.06317875	-2.756715554	0.627914	-4.39028	1.13E-05	6.40E-05	down-regulated	protein_coding
TGIF2	ENSMMUG00000008195	128.6917465	-2.797256677	0.637293	-4.38928	1.14E-05	6.42E-05	down-regulated	protein_coding
MAML2	ENSMMUG00000014806	31.76448113	2.52503183	0.575374	4.388507	1.14E-05	6.44E-05	up-regulated	protein_coding
DUSP28	ENSMMUG00000014836	18.20190743	3.783632212	0.862241	4.388136	1.14E-05	6.45E-05	up-regulated	protein_coding
RAB21	ENSMMUG00000019434	33.81257642	-5.449549928	1.241858	-4.38822	1.14E-05	6.45E-05	down-regulated	protein_coding
RFXANK	ENSMMUG00000010722	184.9108216	2.026992851	0.462045	4.387006	1.15E-05	6.48E-05	up-regulated	protein_coding
NPM1	ENSMMUG00000017463	120.5791713	-4.011742498	0.914865	-4.38507	1.16E-05	6.53E-05	down-regulated	protein_coding
GAA	ENSMMUG00000009589	178.2197947	-2.115934489	0.482562	-4.3848	1.16E-05	6.54E-05	down-regulated	protein_coding
UQCRC1	ENSMMUG00000014705	506.9974412	-1.983047169	0.452323	-4.38414	1.16E-05	6.56E-05	down-regulated	protein_coding
LASS2	ENSMMUG00000023234	544.082369	1.003069178	0.22882	4.383659	1.17E-05	6.57E-05	up-regulated	protein_coding
IL12RB2	ENSMMUG00000000651	11.5079125	5.311216398	1.211855	4.382716	1.17E-05	6.59E-05	up-regulated	protein_coding
CCT6A	ENSMMUG00000015272	134.4386621	-2.272614732	0.518576	-4.38241	1.17E-05	6.60E-05	down-regulated	protein_coding
PSMA4	ENSMMUG00000010740	117.7220477	-2.801214739	0.639222	-4.38222	1.17E-05	6.60E-05	down-regulated	protein_coding
MTRR	ENSMMUG00000018747	95.46575965	1.887502829	0.430887	4.380507	1.18E-05	6.65E-05	up-regulated	protein_coding
AURKAIP1	ENSMMUG00000015830	94.33014238	-4.534237137	1.035353	-4.37941	1.19E-05	6.68E-05	down-regulated	protein_coding
MGST2	ENSMMUG00000021999	70.22455335	-4.65514947	1.062969	-4.37938	1.19E-05	6.68E-05	down-regulated	protein_coding
SLC35D2	ENSMMUG00000007992	70.96947616	-2.804610921	0.640596	-4.37813	1.20E-05	6.72E-05	down-regulated	protein_coding
TRIB1	ENSMMUG00000021759	271.5059284	-1.637709365	0.374121	-4.37748	1.20E-05	6.73E-05	down-regulated	protein_coding
PHC3	ENSMMUG00000022199	86.92308735	2.175822546	0.497045	4.377518	1.20E-05	6.73E-05	up-regulated	protein_coding
COX6B1	ENSMMUG00000016765	238.8873991	-2.091747166	0.477982	-4.3762	1.21E-05	6.77E-05	down-regulated	protein_coding
CORO2A	ENSMMUG00000002853	230.6169893	-2.372699853	0.542302	-4.37524	1.21E-05	6.80E-05	down-regulated	protein_coding
DRAM2	ENSMMUG00000002463	87.24974892	-2.62730478	0.60056	-4.37476	1.22E-05	6.81E-05	down-regulated	protein_coding
CDC34	ENSMMUG00000028950	40.30712548	-5.700950942	1.303254	-4.3744	1.22E-05	6.82E-05	down-regulated	protein_coding
SNX4	ENSMMUG00000011106	68.21637927	-4.560576747	1.043498	-4.37047	1.24E-05	6.94E-05	down-regulated	protein_coding
ACADM	ENSMMUG00000007220	81.77862318	-4.313914927	0.987181	-4.36993	1.24E-05	6.95E-05	down-regulated	protein_coding
YWHAE	ENSMMUG00000019378	99.75194999	-2.205709226	0.504832	-4.36919	1.25E-05	6.97E-05	down-regulated	protein_coding
TMEM175	ENSMMUG00000017221	98.96608999	1.865281307	0.426968	4.36867	1.25E-05	6.99E-05	up-regulated	protein_coding
SLC10A4	ENSMMUG00000006703	6.950731204	4.758827914	1.089567	4.367632	1.26E-05	7.01E-05	up-regulated	protein_coding
MRPS16	ENSMMUG00000011284	74.90375512	-4.766250173	1.091246	-4.36772	1.26E-05	7.01E-05	down-regulated	protein_coding
CXCR5	ENSMMUG00000009316	19.22890656	4.201740786	0.962194	4.366835	1.26E-05	7.03E-05	up-regulated	protein_coding
DDOST	ENSMMUG00000008836	504.1819276	1.531488735	0.350813	4.365543	1.27E-05	7.07E-05	up-regulated	protein_coding
TXN2	ENSMMUG00000031028	34.39607221	-5.472199198	1.253775	-4.36458	1.27E-05	7.10E-05	down-regulated	protein_coding
ACLY	ENSMMUG00000005963	64.00008403	-5.387536111	1.234456	-4.3643	1.28E-05	7.11E-05	down-regulated	protein_coding

TGM1	ENSMMUG00000012452	8.314540809	4.307887937	0.987462	4.362586	1.29E-05	7.16E-05	up-regulated	protein_coding
RAB8A	ENSMMUG00000015784	165.5550265	-2.049866416	0.470057	-4.36089	1.30E-05	7.21E-05	down-regulated	protein_coding
PRPF39	ENSMMUG00000010862	33.16813221	-5.419734957	1.24358	-4.35817	1.31E-05	7.29E-05	down-regulated	protein_coding
SEC61A1	ENSMMUG00000018661	298.3830526	-1.494093486	0.34289	-4.35736	1.32E-05	7.32E-05	down-regulated	protein_coding
SOX4	ENSMMUG00000014454	398.0841791	2.108429146	0.483912	4.357053	1.32E-05	7.32E-05	up-regulated	protein_coding
PDCD4	ENSMMUG00000017204	90.90238027	-2.306063239	0.529261	-4.35714	1.32E-05	7.32E-05	down-regulated	protein_coding
ACPT	ENSMMUG00000003583	16.93953709	4.910731175	1.127133	4.356834	1.32E-05	7.33E-05	up-regulated	protein_coding
FNDC3B	ENSMMUG00000004189	100.2369005	2.424558575	0.55653	4.356567	1.32E-05	7.33E-05	up-regulated	protein_coding
RBM47	ENSMMUG00000012036	450.4595148	-1.728549014	0.396771	-4.35654	1.32E-05	7.33E-05	down-regulated	protein_coding
SLFN12	ENSMMUG00000006042	11.34329912	4.545584062	1.043469	4.356221	1.32E-05	7.34E-05	up-regulated	protein_coding
NTAN1	ENSMMUG00000002989	126.9853936	2.17077181	0.498328	4.356113	1.32E-05	7.34E-05	up-regulated	protein_coding
IDH2	ENSMMUG00000014833	700.8028295	-1.489498635	0.342007	-4.35517	1.33E-05	7.37E-05	down-regulated	protein_coding
CSPG4	ENSMMUG00000016104	14.5845187	6.092092331	1.399316	4.353622	1.34E-05	7.42E-05	up-regulated	protein_coding
GIPC1	ENSMMUG00000005729	169.3385743	-2.474738972	0.568447	-4.35351	1.34E-05	7.42E-05	down-regulated	protein_coding
TPRN	ENSMMUG00000001677	90.68972646	-3.221937708	0.740401	-4.35161	1.35E-05	7.48E-05	down-regulated	protein_coding
FAM149B1	ENSMMUG00000011281	114.8017905	1.626493186	0.373899	4.350082	1.36E-05	7.53E-05	up-regulated	protein_coding
ITPK1	ENSMMUG00000009974	165.8797709	-1.725848145	0.396762	-4.34983	1.36E-05	7.54E-05	down-regulated	protein_coding
BOC	ENSMMUG00000013443	6.828307809	7.303141677	1.680154	4.346709	1.38E-05	7.64E-05	up-regulated	protein_coding
CTNND1	ENSMMUG00000012366	980.1868853	-1.315649579	0.30276	-4.34552	1.39E-05	7.67E-05	down-regulated	protein_coding
PLXNC1	ENSMMUG00000018237	55.62118163	3.529368778	0.812376	4.3445	1.40E-05	7.71E-05	up-regulated	protein_coding
HYLS1	ENSMMUG00000007622	44.32180632	2.743074996	0.631651	4.342705	1.41E-05	7.77E-05	up-regulated	protein_coding
RNF168	ENSMMUG00000004035	23.98986236	2.29535062	0.528636	4.342023	1.41E-05	7.79E-05	up-regulated	protein_coding
ANXA3	ENSMMUG00000022218	160.6341182	-2.270047719	0.522837	-4.34179	1.41E-05	7.79E-05	down-regulated	protein_coding
ATP6V0E2	ENSMMUG00000022273	72.18273181	-3.672789255	0.845936	-4.34169	1.41E-05	7.79E-05	down-regulated	protein_coding
C15H9ORF78	ENSMMUG00000003795	200.9570654	1.603205624	0.369343	4.3407	1.42E-05	7.83E-05	up-regulated	protein_coding
U6	ENSMMUG00000035966	9.752018787	7.201609359	1.661345	4.334805	1.46E-05	8.03E-05	up-regulated	snRNA
PSMD7	ENSMMUG00000011125	102.7294687	-2.913251098	0.672399	-4.33262	1.47E-05	8.11E-05	down-regulated	protein_coding
NRAMP1	ENSMMUG00000013359	37.68530076	2.11893096	0.489125	4.332084	1.48E-05	8.13E-05	up-regulated	protein_coding
IDH3A	ENSMMUG00000011838	117.7132216	-2.565263773	0.592181	-4.33189	1.48E-05	8.13E-05	down-regulated	protein_coding
UQCC1	ENSMMUG00000010403	31.41591552	-5.341776554	1.234772	-4.32612	1.52E-05	8.34E-05	down-regulated	protein_coding
IAH1	ENSMMUG00000018045	80.04050639	-2.527795678	0.584328	-4.32598	1.52E-05	8.34E-05	down-regulated	protein_coding
SNX3	ENSMMUG00000020366	150.5321698	-1.889063657	0.436725	-4.32553	1.52E-05	8.36E-05	down-regulated	protein_coding
WISP1	ENSMMUG00000010199	83.40364459	8.501469386	1.965617	4.325089	1.52E-05	8.37E-05	up-regulated	protein_coding
CHRNE	ENSMMUG00000002409	76.3726613	3.434105355	0.79414	4.324309	1.53E-05	8.40E-05	up-regulated	protein_coding
ARL8B	ENSMMUG00000008810	125.3011373	-2.241773378	0.518468	-4.32384	1.53E-05	8.41E-05	down-regulated	protein_coding
H1FX	ENSMMUG00000013171	221.3216582	-3.197374209	0.739773	-4.3221	1.55E-05	8.48E-05	down-regulated	protein_coding
SLC35G1	ENSMMUG00000001607	191.1579197	-3.766313195	0.871538	-4.32146	1.55E-05	8.49E-05	down-regulated	protein_coding
CACNA1C	ENSMMUG00000012988	15.32731323	3.899652684	0.902391	4.321466	1.55E-05	8.49E-05	up-regulated	protein_coding
TOE1	ENSMMUG00000020081	50.7650852	2.687030793	0.621813	4.321287	1.55E-05	8.50E-05	up-regulated	protein_coding
ALCAM	ENSMMUG00000009107	98.09492289	-2.464138148	0.570372	-4.32023	1.56E-05	8.53E-05	down-regulated	protein_coding
mml-mir-135a-1	ENSMMUG00000027031	22.1554401	3.451204198	0.798969	4.319573	1.56E-05	8.56E-05	up-regulated	miRNA
COX6C	ENSMMUG00000000165	124.9657525	-2.454240476	0.568442	-4.31749	1.58E-05	8.63E-05	down-regulated	protein_coding
SMG7	ENSMMUG00000018458	205.3012085	-2.176557196	0.504131	-4.31744	1.58E-05	8.63E-05	down-regulated	protein_coding
CRB1	ENSMMUG00000012322	8.272638732	5.271364478	1.221669	4.314887	1.60E-05	8.73E-05	up-regulated	protein_coding
ZC3H11A	ENSMMUG00000010539	239.4073748	-1.985263017	0.460143	-4.31444	1.60E-05	8.74E-05	down-regulated	protein_coding
HK1	ENSMMUG00000015302	126.0782468	-2.092259425	0.485088	-4.31315	1.61E-05	8.79E-05	down-regulated	protein_coding
HNRNPL	ENSMMUG00000004877	478.1106876	-2.163735331	0.501671	-4.31306	1.61E-05	8.79E-05	down-regulated	protein_coding
KRTCAP3	ENSMMUG00000005095	74.64718221	-2.676001677	0.62049	-4.31273	1.61E-05	8.80E-05	down-regulated	protein_coding
PRR7	ENSMMUG00000022889	47.59074307	3.315983053	0.769108	4.311464	1.62E-05	8.84E-05	up-regulated	protein_coding
FAR1	ENSMMUG00000011990	69.83688257	-2.964695074	0.687828	-4.31022	1.63E-05	8.89E-05	down-regulated	protein_coding
CTAGE5	ENSMMUG00000021121	82.69917957	-4.866861195	1.12913	-4.31028	1.63E-05	8.89E-05	down-regulated	protein_coding
FBXO7	ENSMMUG00000006728	71.85553551	-2.596495407	0.602666	-4.30835	1.64E-05	8.95E-05	down-regulated	protein_coding
COPB1	ENSMMUG00000016444	155.0361927	-2.39464286	0.555917	-4.30755	1.65E-05	8.98E-05	down-regulated	protein_coding
NFE2L2	ENSMMUG00000001861	307.9602521	-1.372414715	0.318795	-4.305	1.67E-05	9.07E-05	down-regulated	protein_coding
STARD5	ENSMMUG00000011560	104.5405753	-2.538786264	0.589735	-4.30496	1.67E-05	9.07E-05	down-regulated	protein_coding
TMEM8A	ENSMMUG00000000610	434.8218949	-1.492644109	0.346749	-4.30468	1.67E-05	9.08E-05	down-regulated	protein_coding
SLC4A10	ENSMMUG00000008384	11.62234285	5.346682156	1.242107	4.304526	1.67E-05	9.09E-05	up-regulated	protein_coding
CST7	ENSMMUG00000005757	13.57071705	7.365365173	1.71111	4.304436	1.67E-05	9.09E-05	up-regulated	protein_coding
SMEK1	ENSMMUG00000014945	86.1757688	-2.939376996	0.683063	-4.30323	1.68E-05	9.13E-05	down-regulated	protein_coding
FXYD3	ENSMMUG00000022127	128.2850238	-3.310331431	0.769328	-4.30289	1.69E-05	9.14E-05	down-regulated	protein_coding
OSTF1	ENSMMUG00000010401	58.25240595	-5.232674534	1.216497	-4.30143	1.70E-05	9.20E-05	down-regulated	protein_coding
KBTBD4	ENSMMUG00000004289	122.520869	2.631377598	0.611845	4.300727	1.70E-05	9.23E-05	up-regulated	protein_coding
RCN2	ENSMMUG00000008213	63.48231416	-4.514236906	1.049669	-4.30063	1.70E-05	9.23E-05	down-regulated	protein_coding
GLB1L	ENSMMUG00000019624	103.8422592	2.375026518	0.552307	4.300196	1.71E-05	9.24E-05	up-regulated	protein_coding
STK3	ENSMMUG00000028952	31.4805638	-5.345582117	1.24322	-4.29979	1.71E-05	9.25E-05	down-regulated	protein_coding
PIM3	ENSMMUG00000002157	100.5968154	-3.941561053	0.917019	-4.29823	1.72E-05	9.32E-05	down-regulated	protein_coding
MTUS2	ENSMMUG00000008658	12.03689727	5.717183159	1.330722	4.296302	1.74E-05	9.39E-05	up-regulated	protein_coding
ADIPOR1	ENSMMUG00000007046	195.7034362	-1.419930301	0.330622	-4.29472	1.75E-05	9.45E-05	down-regulated	protein_coding
RRNAD1	ENSMMUG00000000038	187.9337747	1.052100644	0.245094	4.292647	1.77E-05	9.53E-05	up-regulated	protein_coding
ATP6V1B2	ENSMMUG00000020000	189.1840791	-1.879233704	0.438042	-4.29007	1.79E-05	9.63E-05	down-regulated	protein_coding
TUBB2C	ENSMMUG00000012164	1173.027282	-2.298547302	0.536155	-4.2871	1.81E-05	9.76E-05	down-regulated	protein_coding
FAM160A2	ENSMMUG00000016882	80.83753269	-2.281285328	0.532147	-4.28695	1.81E-05	9.76E-05	down-regulated	protein_coding
PCDHGA8	ENSMMUG00000029806	18.68305883	3.058214862	0.71367	4.285195	1.83E-05	9.84E-05	up-regulated	protein_coding
SLC35A2	ENSMMUG00000004553	64.94859308	-2.864903558	0.668794	-4.28368	1.84E-05	9.90E-05	down-regulated	protein_coding
AARS	ENSMMUG00000021653	198.3193467	-1.825130725	0.426191	-4.28243	1.85E-05	9.95E-05	down-regulated	protein_coding
SUGP2	ENSMMUG00000019665	192.3378803	1.629199627	0.380714	4.279321	1.87E-05	0.000100815	up-regulated	protein_coding
THEM6	ENSMMUG00000018302	86.32028417	-4.401205242	1.028524	-4.27915	1.88E-05	0.000100856	down-regulated	protein_coding
EHD1	ENSMMUG00000003972	174.6004839	-1.971335135	0.460754	-4.2785	1.88E-05	0.000101112	down-regulated	protein_coding
SDR16C5	ENSMMUG00000014810	7.60903304	6.240545169	1.458971	4.277361	1.89E-05	0.00010159	up-regulated	protein_coding
FDXR	ENSMMUG00000015648	97.75064771	-2.446625904	0.572457	-4.2739	1.92E-05	0.00010314	down-regulated	protein_coding
DENND2D	ENSMMUG00000002457	108.4698043	1.316515955	0.308161	4.272169	1.94E-05	0.000103831	up-regulated	protein_coding
GCDH	ENSMMUG00000007043	71.63504549	-3.065031006	0.717443	-4.27216	1.94E-05	0.000103831	down-regulated	protein_coding
SCYL1	ENSMMUG00000023477	168.6549348	-2.068030177	0.484098	-4.27192	1.94E-05	0.000103904	down-regulated	protein_coding
HEATR9	ENSMMUG00000022233	9.11657994	5.851498003	1.369876	4.271552	1.94E-05	0.000104036	up-regulated	protein_coding
MRP63	ENSMMUG00000016562	38.73764602	-5.643553929	1.321282	-4.27127	1.94E-05	0.000104128	down-regulated	protein_coding
PTPN1	ENSMMUG00000007878	68.76952416	-2.477823466	0.580597	-4.26772	1.97E-05	0.000105746	down-regulated	protein_coding
PFKFB1	ENSMMUG00000008425	8.582771881	6.434829999	1.507811	4.267663	1.98E-05	0.000105746	up-regulated	protein_coding
DUSP1	ENSMMUG00000008844	204.7134252	-2.600727196	0.609465	-4.26723	1.98E-05	0.00010589	down-regulated	protein_coding
OTUD7A	ENSMMUG00000012838	6.093435196	6.614893724	1.550175	4.267191	1.98E-05	0.00010589	up-regulated	protein_coding
GTF2A2	ENSMMUG00000018428	32.33943588	-5.38331241	1.261694	-4.26674	1.98E-05	0.000106066	down-regulated	protein_coding
ND6	ENSMMUG00000028672	64344.4411	9.734255399	2.281613	4.266392	1.99E-05	0.000106189	up-regulated	protein_coding
TRHDE	ENSMMUG00000014775	35.38551783	3.712079968	0.870658	4.263533	2.01E-05	0.000107517	up-regulated	protein_coding
IZUMO4	ENSMMUG00000015660	15.1341691	3.116253994	0.73139	4.260729	2.04E-05	0.000108765	up-regulated	protein_coding
CHRNB2	ENSMMUG00000016089	6.298097774	4.959374777	1.163981	4.260702	2.04E-05	0.000108765	up-regulated	protein_coding
SAP30L	ENSMMUG00000010619	30.47192088	-5.297666939	1.243994	-4.25859	2.06E-05	0.000109755	down-regulated	protein_coding
USP33	ENSMMUG00000018326	72.97588681	-2.975808936	0.698877	-4.25799	2.06E-05	0.00011001	down-regulated	protein_coding
RBM39	ENSMMUG00000031284	67.62869324	-3.881216342	0.912273	-4.25444	2.10E-05	0.000111725	down-regulated	protein_coding
MTPN	ENSMMUG00000017108	238.2776349	-2.120445951	0.498603	-4.25278	2.11E-05	0.000112518	down-regulated	protein_coding
IFT140	ENSMMUG00000000044	49.10372185	2.149783654	0.505693	4.251164	2.13E-05	0.000113258	up-regulated	protein_coding
CLK1	ENSMMUG00000006640	68.7486446	-4.625173709	1.087984	-4.25114	2.13E-05	0.000113258	down-regulated	protein_coding

ENTPD3	ENSMMUG00000013330	9.939599797	4.318922999	1.016143	4.250309	2.13E-05	0.000113637	up-regulated	protein_coding
USP47	ENSMMUG00000022860	101.438855	-2.250986274	0.529672	-4.24977	2.14E-05	0.000113867	down-regulated	protein_coding
ESD	ENSMMUG00000004209	80.18603213	-4.266992902	1.004468	-4.24801	2.16E-05	0.000114722	down-regulated	protein_coding
HSP90B1	ENSMMUG00000023469	678.5243735	-1.583741705	0.372935	-4.2467	2.17E-05	0.000115353	down-regulated	protein_coding
AK3	ENSMMUG00000000143	283.7501566	-2.712865123	0.638954	-4.24579	2.18E-05	0.000115701	down-regulated	protein_coding
CRIPT	ENSMMUG00000005388	82.13693656	-2.170783241	0.511275	-4.24582	2.18E-05	0.000115701	down-regulated	protein_coding
PON2	ENSMMUG00000008014	118.4686336	-2.164320404	0.509759	-4.24577	2.18E-05	0.000115701	down-regulated	protein_coding
MAMLD1	ENSMMUG00000022263	18.71374475	2.592518981	0.610626	4.245676	2.18E-05	0.000115706	up-regulated	protein_coding
FBXO33	ENSMMUG00000006984	68.33764311	1.439961542	0.339254	4.244498	2.19E-05	0.000116272	up-regulated	protein_coding
7SK	ENSMMUG00000023869	9.558423892	6.918911327	1.630677	4.242968	2.21E-05	0.000117024	up-regulated	misc_RNA
PSMB7	ENSMMUG00000007358	593.7001089	1.917407786	0.451943	4.242584	2.21E-05	0.00011718	up-regulated	protein_coding
ATXN7L3	ENSMMUG00000019923	74.72564808	-2.67581886	0.630932	-4.24105	2.22E-05	0.000117938	down-regulated	protein_coding
KPNA2	ENSMMUG00000018256	79.83254857	-5.708892792	1.346788	-4.23889	2.25E-05	0.000119034	down-regulated	protein_coding
CS	ENSMMUG00000002483	595.7181434	-1.334462079	0.314914	-4.23754	2.26E-05	0.000119709	down-regulated	protein_coding
BLMH	ENSMMUG00000001026	79.3810637	-2.666208105	0.629283	-4.2369	2.27E-05	0.000120006	down-regulated	protein_coding
SNRPN	ENSMMUG00000023139	57.44619004	-5.227537498	1.2342	-4.23557	2.28E-05	0.000120674	down-regulated	protein_coding
PTPRR	ENSMMUG00000011075	100.8489536	-3.132899009	0.739994	-4.23368	2.30E-05	0.00012164	down-regulated	protein_coding
IGSF22	ENSMMUG00000007131	7.50056125	4.843641039	1.144195	4.233232	2.30E-05	0.000121798	up-regulated	protein_coding
SNORA12	ENSMMUG00000032802	5.834522178	5.120910047	1.210054	4.23197	2.32E-05	0.000122438	up-regulated	snoRNA
TRIM32	ENSMMUG00000013107	31.41083111	-5.341807306	1.262922	-4.22972	2.34E-05	0.000123622	down-regulated	protein_coding
RPS29	ENSMMUG00000029367	90.68317933	-4.981791221	1.177873	-4.22948	2.34E-05	0.000123708	down-regulated	protein_coding
TXNDC9	ENSMMUG00000018947	33.77277023	-5.445837863	1.287814	-4.22875	2.35E-05	0.000124066	down-regulated	protein_coding
ECM1	ENSMMUG00000006257	148.0878752	-4.72494558	1.117477	-4.22823	2.36E-05	0.000124307	down-regulated	protein_coding
GDF15	ENSMMUG00000021180	611.121274	-2.72015323	0.643569	-4.22667	2.37E-05	0.000125124	down-regulated	protein_coding
SYT16	ENSMMUG00000008766	9.062689768	3.982339658	0.94224	4.22646	2.37E-05	0.000125193	up-regulated	protein_coding
MRPS18A	ENSMMUG00000014749	71.84065584	-3.34304275	0.791503	-4.22366	2.40E-05	0.000126709	down-regulated	protein_coding
ING2	ENSMMUG00000008881	29.11376046	-5.232537188	1.239052	-4.22302	2.41E-05	0.000126999	down-regulated	protein_coding
7SK	ENSMMUG00000034829	6.098083734	6.576933165	1.558128	4.221047	2.43E-05	0.000127999	up-regulated	misc_RNA
WISP3	ENSMMUG00000012582	14.78323352	3.859247013	0.914377	4.22063	2.44E-05	0.000128188	up-regulated	protein_coding
GSTZ1	ENSMMUG00000001097	77.38931584	-3.424763436	0.811608	-4.21973	2.45E-05	0.000128656	down-regulated	protein_coding
SPECC1	ENSMMUG00000018579	151.4739577	1.644076051	0.389765	4.218123	2.46E-05	0.000129525	up-regulated	protein_coding
TMEM87A	ENSMMUG00000014515	124.4468917	-1.700278396	0.403164	-4.21734	2.47E-05	0.000129929	down-regulated	protein_coding
DNM3	ENSMMUG00000007273	12.99359885	3.333532092	0.79046	4.217203	2.47E-05	0.000129958	up-regulated	protein_coding
TMEM106B	ENSMMUG00000005159	32.37850978	-5.387180294	1.277601	-4.21664	2.48E-05	0.000130235	down-regulated	protein_coding
RGS6	ENSMMUG00000001820	10.02042669	7.829085693	1.857835	4.214091	2.51E-05	0.000131568	up-regulated	protein_coding
MRPL13	ENSMMUG00000002599	34.29661727	-5.467701163	1.297601	-4.2137	2.51E-05	0.000131747	down-regulated	protein_coding
TFE3	ENSMMUG00000023494	77.59504546	-2.392350113	0.5679	-4.21263	2.52E-05	0.000132324	down-regulated	protein_coding
SRP9	ENSMMUG00000000134	198.298017	-2.485611584	0.59015	-4.21183	2.53E-05	0.000132716	down-regulated	protein_coding
DNAJB14	ENSMMUG00000006831	48.83586512	2.47515157	0.587672	4.211794	2.53E-05	0.000132716	up-regulated	protein_coding
LMBRD1	ENSMMUG00000005573	116.598223	-2.381713595	0.565653	-4.21055	2.55E-05	0.000133397	down-regulated	protein_coding
EMC3	ENSMMUG00000018290	57.81669674	-3.731652952	0.886283	-4.21045	2.55E-05	0.000133408	down-regulated	protein_coding
C5ORF62	ENSMMUG00000016864	117.0998914	-1.940720551	0.46096	-4.21017	2.55E-05	0.000133523	down-regulated	protein_coding
TMEM106C	ENSMMUG00000011441	131.3478143	-2.243134998	0.533147	-4.20735	2.58E-05	0.00013512	down-regulated	protein_coding
NCKAP1	ENSMMUG00000014397	209.331669	-2.410795596	0.573008	-4.20727	2.58E-05	0.00013512	down-regulated	protein_coding
RPL10A	ENSMMUG00000015907	799.569566	-2.584642585	0.614333	-4.20724	2.59E-05	0.00013512	down-regulated	protein_coding
TRAPPC3	ENSMMUG00000022499	70.4385954	-2.977438285	0.707757	-4.20686	2.59E-05	0.000135294	down-regulated	protein_coding
PLEK2	ENSMMUG00000021420	82.14426866	-3.072774205	0.730796	-4.2047	2.61E-05	0.000136546	down-regulated	protein_coding
PANK3	ENSMMUG00000012521	120.0531541	-2.84957315	0.677827	-4.20398	2.62E-05	0.000136927	down-regulated	protein_coding
PARD6G	ENSMMUG00000013708	16.64616403	4.332119421	1.031395	4.200254	2.67E-05	0.000139149	up-regulated	protein_coding
AMMECR1	ENSMMUG00000023423	22.08057778	2.745198637	0.653684	4.199578	2.67E-05	0.000139514	up-regulated	protein_coding
NCOA4	ENSMMUG00000015182	277.7622017	-1.840441595	0.438472	-4.19739	2.70E-05	0.000140762	down-regulated	protein_coding
KDM2B	ENSMMUG00000021564	114.5519753	1.987310774	0.473693	4.195358	2.72E-05	0.00014198	up-regulated	protein_coding
RRP1B	ENSMMUG00000000104	84.80697651	1.955270949	0.466162	4.1944	2.74E-05	0.000142487	up-regulated	protein_coding
CTNND2	ENSMMUG00000023419	7.138616683	6.236141303	1.486784	4.194383	2.74E-05	0.000142487	up-regulated	protein_coding
ATP5J	ENSMMUG00000008725	81.11075985	-2.703710784	0.644647	-4.1941	2.74E-05	0.000142615	down-regulated	protein_coding
USP35	ENSMMUG00000017476	41.87108554	2.069561184	0.493502	4.193623	2.75E-05	0.00014286	up-regulated	protein_coding
RAB3D	ENSMMUG00000011317	121.0211861	-1.951546823	0.465442	-4.19289	2.75E-05	0.000143257	down-regulated	protein_coding
DSG2	ENSMMUG00000021095	321.0642335	-2.72530837	0.649993	-4.19283	2.75E-05	0.000143257	down-regulated	protein_coding
UBA3	ENSMMUG00000008620	90.41286015	-2.485462046	0.592842	-4.19245	2.76E-05	0.000143443	down-regulated	protein_coding
TBL2	ENSMMUG00000009093	55.88028633	-3.685655159	0.879228	-4.19192	2.77E-05	0.000143724	down-regulated	protein_coding
TP73	ENSMMUG00000016569	11.62697267	3.505019399	0.836289	4.191157	2.78E-05	0.000144157	up-regulated	protein_coding
DDX41	ENSMMUG00000016911	68.41668267	-3.60803331	0.860889	-4.19105	2.78E-05	0.000144171	down-regulated	protein_coding
TOB1	ENSMMUG00000022740	65.35262079	-3.21240673	0.766888	-4.18889	2.80E-05	0.000145501	down-regulated	protein_coding
GLS	ENSMMUG00000005612	120.506136	-2.774284959	0.662398	-4.18824	2.81E-05	0.000145859	down-regulated	protein_coding
DDX1	ENSMMUG00000023437	124.2141736	-1.799936196	0.429813	-4.18772	2.82E-05	0.000146145	down-regulated	protein_coding
OPA1	ENSMMUG00000009765	89.78210064	-2.401730252	0.573568	-4.18735	2.82E-05	0.000146328	down-regulated	protein_coding
U7	ENSMMUG00000035649	17.15635933	2.676044948	0.639606	4.183895	2.87E-05	0.000148516	up-regulated	snRNA
GULP1	ENSMMUG00000008265	110.0649568	1.646211515	0.393505	4.183463	2.87E-05	0.000148744	up-regulated	protein_coding
KCNK1	ENSMMUG00000022429	92.11354598	-2.37772122	0.568387	-4.18328	2.87E-05	0.000148762	down-regulated	protein_coding
SYNCRIP	ENSMMUG00000012575	130.9813347	-1.97311095	0.471712	-4.18287	2.88E-05	0.000148967	down-regulated	protein_coding
SFT2D1	ENSMMUG00000011736	56.92798394	-5.216207988	1.247565	-4.18111	2.90E-05	0.000149963	down-regulated	protein_coding
UNC45B	ENSMMUG00000005170	9.968780121	7.329356828	1.753513	4.179813	2.92E-05	0.000150674	up-regulated	protein_coding
TAGAP	ENSMMUG00000011994	209.5393681	3.994598826	0.955695	4.179785	2.92E-05	0.000150674	up-regulated	protein_coding
KDM6B	ENSMMUG00000008173	200.9942062	-1.417684272	0.339237	-4.17904	2.93E-05	0.000151112	down-regulated	protein_coding
ATF4	ENSMMUG00000004499	622.5965413	-2.113869881	0.505931	-4.17818	2.94E-05	0.000151576	down-regulated	protein_coding
TCTEX1D4	ENSMMUG00000006580	8.947106837	4.095836178	0.98029	4.17819	2.94E-05	0.000151576	up-regulated	protein_coding
SCTR	ENSMMUG00000011128	95.91444514	2.796020373	0.669359	4.177158	2.95E-05	0.000152202	up-regulated	protein_coding
RPL21	ENSMMUG00000029598	5.724401656	5.506067845	1.31945	4.173002	3.01E-05	0.00015495	up-regulated	protein_coding
ANAPC16	ENSMMUG00000015600	56.71408546	-5.208700966	1.249356	-4.16911	3.06E-05	0.000157506	down-regulated	protein_coding
DDX39B	ENSMMUG00000008839	270.1092691	-2.467448193	0.591953	-4.16832	3.07E-05	0.000157997	down-regulated	protein_coding
LGALS2	ENSMMUG00000002866	56.44521834	2.811665589	0.674644	4.167629	3.08E-05	0.000158416	up-regulated	protein_coding
RGS20	ENSMMUG00000015417	10.7985127	5.666919415	1.360011	4.16682	3.09E-05	0.000158921	up-regulated	protein_coding
MSC	ENSMMUG00000000947	12.29504227	7.395134287	1.77534	4.165476	3.11E-05	0.000159802	up-regulated	protein_coding
KMT2D	ENSMMUG00000015718	357.7397102	1.324889143	0.318139	4.164497	3.12E-05	0.000160431	up-regulated	protein_coding
ZNF496	ENSMMUG00000019585	31.23159231	2.553444502	0.613584	4.161522	3.16E-05	0.000162476	up-regulated	protein_coding
HIF3A	ENSMMUG00000000215	13.99655803	4.070433963	0.978206	4.161122	3.17E-05	0.000162643	up-regulated	protein_coding
GPR113	ENSMMUG00000004383	271.3996947	2.126095487	0.510936	4.161178	3.17E-05	0.000162643	up-regulated	protein_coding
U6	ENSMMUG00000028413	12.75785284	7.184627236	1.726717	4.160861	3.17E-05	0.00016277	up-regulated	snRNA
ERCC6L	ENSMMUG00000012957	48.40030537	2.842391781	0.683277	4.159943	3.18E-05	0.000163366	up-regulated	protein_coding
RAF1	ENSMMUG00000007450	235.3827064	1.462436189	0.351574	4.15968	3.19E-05	0.000163495	up-regulated	protein_coding
PLS3	ENSMMUG00000006377	61.22505044	-4.453318836	1.070622	-4.15956	3.19E-05	0.00016352	down-regulated	protein_coding
TANC2	ENSMMUG00000013411	31.44484597	4.339908218	1.043651	4.15839	3.20E-05	0.000164302	up-regulated	protein_coding
EDAR	ENSMMUG00000019574	12.41062917	4.00518635	0.963193	4.158239	3.21E-05	0.000164351	up-regulated	protein_coding
GAS8	ENSMMUG00000000401	93.02853299	1.744688823	0.419676	4.157227	3.22E-05	0.000165021	up-regulated	protein_coding
STAT5	ENSMMUG00000010705	45.55856248	2.274075061	0.547426	4.154122	3.27E-05	0.000167216	up-regulated	protein_coding
DVL2	ENSMMUG00000010542	77.89078306	-3.178760513	0.765246	-4.1539	3.27E-05	0.000167316	down-regulated	protein_coding
APEH	ENSMMUG00000014540	239.403747	-1.836893665	0.442317	-4.15289	3.28E-05	0.000167996	down-regulated	protein_coding
Y_RNA	ENSMMUG00000026490	6.083053525	5.926671059	1.427176	4.152727	3.29E-05	0.000168057	up-regulated	misc_RNA
TRA2B	ENSMMUG00000001185	138.3149405	-2.232717714	0.537852	-4.15117	3.31E-05	0.00016914	down-regulated	protein_coding
SURF4	ENSMMUG00000022348	499.3393541	-1.328386722	0.320025	-4.15089	3.31E-05	0.00016923	down-regulated	protein_coding

ZBTB20	ENSMMUG00000020641	18.66320865	3.59385773	0.866001	4.149944	3.33E-05	0.000169812	up-regulated	protein_coding
ACADS	ENSMMUG00000020648	164.4205033	-2.217526209	0.53468	-4.14739	3.36E-05	0.00017165	down-regulated	protein_coding
B3GNT5	ENSMMUG00000031605	166.1290213	-3.090324841	0.745336	-4.14622	3.38E-05	0.000172437	down-regulated	protein_coding
ZP3	ENSMMUG00000003637	11.7853878	6.271680089	1.513032	4.145108	3.40E-05	0.000173181	up-regulated	protein_coding
AP5M1	ENSMMUG00000018279	60.68838761	-3.337365972	0.805166	-4.14494	3.40E-05	0.000173245	down-regulated	protein_coding
TMCO4	ENSMMUG00000002492	89.46347496	3.380945827	0.81576	4.144533	3.41E-05	0.000173422	up-regulated	protein_coding
GLRX3	ENSMMUG00000018429	55.11103094	-5.166476513	1.246599	-4.14446	3.41E-05	0.000173422	down-regulated	protein_coding
GUK1	ENSMMUG00000023085	166.7194775	-2.674299604	0.645608	-4.1423	3.44E-05	0.000175003	down-regulated	protein_coding
NOTCH1	ENSMMUG00000017022	118.3895493	2.430401961	0.586991	4.140441	3.47E-05	0.000176298	up-regulated	protein_coding
METRNL	ENSMMUG00000001111	82.22992458	-3.392908389	0.819485	-4.14029	3.47E-05	0.000176347	down-regulated	protein_coding
PBX2	ENSMMUG00000018456	89.86304759	1.82121352	0.439945	4.139641	3.48E-05	0.000176786	up-regulated	protein_coding
GNA12	ENSMMUG00000006460	57.68239138	-4.368696737	1.055629	-4.13848	3.50E-05	0.000177622	down-regulated	protein_coding
GTF2E2	ENSMMUG00000000459	58.2280507	-3.738810506	0.903628	-4.13756	3.51E-05	0.000178272	down-regulated	protein_coding
Y_RNA	ENSMMUG00000037187	7.230217669	5.393942357	1.304082	4.136199	3.53E-05	0.000179265	up-regulated	misc_RNA
C8ORF59	ENSMMUG00000006381	31.78600213	-5.358170368	1.295659	-4.13548	3.54E-05	0.000179698	down-regulated	protein_coding
SENP8	ENSMMUG00000000490	26.54184379	2.232322076	0.539886	4.1348	3.55E-05	0.000180166	up-regulated	protein_coding
CDA	ENSMMUG00000001498	65.50731745	-3.490770582	0.844311	-4.13446	3.56E-05	0.000180367	down-regulated	protein_coding
FBXL3	ENSMMUG00000021458	30.26429877	-5.289827606	1.279741	-4.13352	3.57E-05	0.000181047	down-regulated	protein_coding
AZI2	ENSMMUG00000000056	28.22172557	-5.188598288	1.255439	-4.13289	3.58E-05	0.000181407	down-regulated	protein_coding
TRIM31	ENSMMUG00000012125	50.44460569	2.698801153	0.653005	4.132896	3.58E-05	0.000181407	up-regulated	protein_coding
TUB	ENSMMUG00000015012	10.8052575	5.485672953	1.327612	4.131986	3.60E-05	0.000182007	up-regulated	protein_coding
NICN1	ENSMMUG00000031780	68.69937076	2.187028221	0.529313	4.131823	3.60E-05	0.000182059	up-regulated	protein_coding
STAM2	ENSMMUG00000010527	29.76226564	-5.265689216	1.274506	-4.13155	3.60E-05	0.000182208	down-regulated	protein_coding
SLC50A1	ENSMMUG00000013219	120.7336248	-1.656136651	0.400863	-4.13143	3.61E-05	0.00018224	down-regulated	protein_coding
USP4	ENSMMUG00000000822	55.89724388	-3.247660257	0.786128	-4.13121	3.61E-05	0.000182349	down-regulated	protein_coding
FBLN2	ENSMMUG00000017659	8.403167028	4.921506749	1.191983	4.12884	3.65E-05	0.000184173	up-regulated	protein_coding
LYPLA1	ENSMMUG00000013157	67.69619252	-4.614246466	1.118462	-4.12553	3.70E-05	0.000186645	down-regulated	protein_coding
DHX15	ENSMMUG00000009637	152.674876	-1.677428328	0.40668	-4.12469	3.71E-05	0.00018726	down-regulated	protein_coding
C8A	ENSMMUG00000022992	8.934474605	6.728452366	1.631664	4.123676	3.73E-05	0.000188018	up-regulated	protein_coding
CDC5L	ENSMMUG00000014389	64.18888339	-3.151800938	0.764471	-4.12285	3.74E-05	0.000188626	down-regulated	protein_coding
STX8	ENSMMUG00000009398	29.00088272	-5.22585807	1.267788	-4.12203	3.76E-05	0.000189232	down-regulated	protein_coding
MUC13	ENSMMUG00000014184	5589.755448	-2.032701479	0.493143	-4.12193	3.76E-05	0.000189245	down-regulated	protein_coding
C10H22ORF28	ENSMMUG00000006720	162.690093	-1.627549011	0.394874	-4.1217	3.76E-05	0.000189371	down-regulated	protein_coding
RNASET2	ENSMMUG00000005673	64.89159699	-2.610778016	0.633664	-4.12013	3.79E-05	0.000190597	down-regulated	protein_coding
GPS1	ENSMMUG00000012800	420.582922	2.062069943	0.500506	4.119972	3.79E-05	0.000190658	up-regulated	protein_coding
MPZL2	ENSMMUG00000023595	92.24814554	-3.434106411	0.833803	-4.11861	3.81E-05	0.000191724	down-regulated	protein_coding
KMT2E	ENSMMUG00000022727	312.4017336	1.299582619	0.315618	4.117578	3.83E-05	0.000192513	up-regulated	protein_coding
C19orf35	ENSMMUG00000017950	7.278291041	4.771462755	1.15903	4.116773	3.84E-05	0.000193117	up-regulated	protein_coding
SUCLG2	ENSMMUG00000009693	227.5208302	-1.445986283	0.35131	-4.11599	3.86E-05	0.000193709	down-regulated	protein_coding
STUB1	ENSMMUG00000001983	1059.783448	2.042916185	0.496431	4.11521	3.87E-05	0.000194293	up-regulated	protein_coding
SIKE1	ENSMMUG00000001973	27.19366882	-5.13332124	1.247466	-4.115	3.87E-05	0.000194402	down-regulated	protein_coding
DBF4	ENSMMUG00000006771	73.45097254	2.844506373	0.691366	4.114328	3.88E-05	0.000194899	up-regulated	protein_coding
COPS8	ENSMMUG00000021605	63.52331216	-3.778575036	0.91857	-4.11354	3.90E-05	0.000195496	down-regulated	protein_coding
ADCY10	ENSMMUG00000010380	13.25957144	5.205471473	1.265785	4.112444	3.91E-05	0.000196357	up-regulated	protein_coding
LAMP2	ENSMMUG00000001484	66.32922061	-5.441061487	1.323518	-4.11106	3.94E-05	0.000197399	down-regulated	protein_coding
GANAB	ENSMMUG00000018117	623.7109311	-1.15381647	0.28067	-4.11094	3.94E-05	0.000197434	down-regulated	protein_coding
SH3BGRL	ENSMMUG00000001689	83.06565422	-2.688455323	0.654071	-4.11034	3.95E-05	0.000197874	down-regulated	protein_coding
SREBF1	ENSMMUG00000023375	558.2599285	-1.303998317	0.317285	-4.10986	3.96E-05	0.000198215	down-regulated	protein_coding
IL6ST	ENSMMUG00000001893	81.3142656	-4.293339352	1.045287	-4.10733	4.00E-05	0.000200326	down-regulated	protein_coding
EIF3J	ENSMMUG00000014288	61.45715911	-3.405681094	0.829613	-4.10514	4.04E-05	0.000202162	down-regulated	protein_coding
PSME3	ENSMMUG00000012685	63.75849014	-2.79820927	0.681774	-4.10431	4.06E-05	0.000202823	down-regulated	protein_coding
SSB	ENSMMUG00000023051	164.6033018	-1.916747826	0.467023	-4.10418	4.06E-05	0.000202857	down-regulated	protein_coding
UBE2A	ENSMMUG00000022667	77.95474658	-2.223833692	0.541977	-4.10319	4.07E-05	0.000203661	down-regulated	protein_coding
C4ORF52	ENSMMUG00000006680	52.36830164	-5.091658739	1.241157	-4.10235	4.09E-05	0.000204259	down-regulated	protein_coding
KLF10	ENSMMUG00000018815	127.2016615	-2.821078421	0.687854	-4.10127	4.11E-05	0.000205137	down-regulated	protein_coding
PAPLN	ENSMMUG00000020195	63.57202607	1.734067733	0.422989	4.099554	4.14E-05	0.000206521	up-regulated	protein_coding
5S_rRNA	ENSMMUG00000034702	13.59342196	6.634271818	1.618674	4.098585	4.16E-05	0.000207314	up-regulated	rRNA
PAQR8	ENSMMUG00000013715	143.5261473	-2.429765568	0.593061	-4.09699	4.19E-05	0.000208674	down-regulated	protein_coding
TUBA3C	ENSMMUG00000006668	7.145809978	6.004024594	1.466031	4.095427	4.21E-05	0.000210013	up-regulated	protein_coding
LLGL2	ENSMMUG00000001228	152.7058439	-1.967794178	0.480712	-4.0935	4.25E-05	0.000211694	down-regulated	protein_coding
CCNDBP1	ENSMMUG00000022253	52.29162975	-5.090999056	1.2439	-4.09277	4.26E-05	0.000212282	down-regulated	protein_coding
GK	ENSMMUG00000005959	84.48976124	-4.951452908	1.210105	-4.09176	4.28E-05	0.000213066	down-regulated	protein_coding
STK39	ENSMMUG00000006215	246.1144975	-1.353082138	0.330724	-4.09127	4.29E-05	0.000213433	down-regulated	protein_coding
VPS37B	ENSMMUG00000015222	62.6590831	-3.373368908	0.82459	-4.09096	4.30E-05	0.000213644	down-regulated	protein_coding
NDUFA7	ENSMMUG00000016697	79.48601922	-3.872259975	0.946628	-4.09058	4.30E-05	0.000213921	down-regulated	protein_coding
CD72	ENSMMUG00000016318	5.295498599	5.069300027	1.239366	4.090236	4.31E-05	0.000214118	up-regulated	protein_coding
CISD1	ENSMMUG00000019823	65.21061314	-4.547963565	1.111915	-4.09021	4.31E-05	0.000214118	down-regulated	protein_coding
EIF3C	ENSMMUG00000011798	418.13739	-1.631676111	0.399012	-4.08929	4.33E-05	0.00021489	down-regulated	protein_coding
CD9	ENSMMUG00000004470	616.2595763	-1.993421083	0.487525	-4.08886	4.33E-05	0.0002152	down-regulated	protein_coding
DDX3X	ENSMMUG00000017569	181.6588341	-2.937243534	0.718365	-4.08879	4.34E-05	0.0002152	down-regulated	protein_coding
MMU.4022	ENSMMUG00000013766	398.7690656	-1.583479484	0.387397	-4.08748	4.36E-05	0.000216297	down-regulated	protein_coding
TCEA1	ENSMMUG00000016022	64.23593373	-3.141795071	0.76883	-4.08646	4.38E-05	0.000217143	down-regulated	protein_coding
U5	ENSMMUG00000027468	8.751887115	5.607198302	1.372417	4.085638	4.40E-05	0.00021784	up-regulated	snRNA
SMIM15	ENSMMUG00000021815	61.21437978	-2.496740825	0.611152	-4.0853	4.40E-05	0.000218076	down-regulated	protein_coding
MFSD5	ENSMMUG00000022020	27.39994597	-5.143828861	1.259206	-4.08498	4.41E-05	0.000218308	down-regulated	protein_coding
AAGAB	ENSMMUG00000015456	80.15534173	-2.336713579	0.572121	-4.0843	4.42E-05	0.00021887	down-regulated	protein_coding
SCN9A	ENSMMUG00000018510	9.958347723	5.090770001	1.246459	4.084186	4.42E-05	0.000218899	up-regulated	protein_coding
HN1	ENSMMUG00000016654	272.2786417	-2.249214316	0.550752	-4.0839	4.43E-05	0.000219096	down-regulated	protein_coding
C22ORF24	ENSMMUG00000009947	7.586688503	4.42312164	1.083183	4.083447	4.44E-05	0.000219444	up-regulated	protein_coding
ACCN1	ENSMMUG00000011413	16.43501328	5.096245171	1.248062	4.083325	4.44E-05	0.000219481	up-regulated	protein_coding
RER1	ENSMMUG00000012346	196.4929627	-1.782430406	0.436583	-4.08268	4.45E-05	0.000219934	down-regulated	protein_coding
IRF2BP2	ENSMMUG00000020252	200.9383064	-1.297618106	0.317832	-4.08272	4.45E-05	0.000219934	down-regulated	protein_coding
FAM160B2	ENSMMUG00000013017	109.2096061	1.316572393	0.322568	4.081536	4.47E-05	0.000220947	up-regulated	protein_coding
PCBD1	ENSMMUG00000008789	102.9687968	-2.693585942	0.660001	-4.08119	4.48E-05	0.000221202	down-regulated	protein_coding
CCDC109B	ENSMMUG00000008171	55.0628422	2.665544491	0.653178	4.080883	4.49E-05	0.000221414	up-regulated	protein_coding
GIGYF2	ENSMMUG00000012211	212.5062676	1.549199177	0.379649	4.080608	4.49E-05	0.000221599	up-regulated	protein_coding
LSM4	ENSMMUG00000004405	141.868077	-3.002925013	0.735997	-4.08008	4.50E-05	0.000222027	down-regulated	protein_coding
SLC35B1	ENSMMUG00000008870	67.20316884	-4.501924193	1.103511	-4.07964	4.51E-05	0.000222372	down-regulated	protein_coding
TMUB1	ENSMMUG00000020466	85.39880379	-3.030249851	0.743244	-4.07706	4.56E-05	0.000224692	down-regulated	protein_coding
RNF123	ENSMMUG00000014564	322.8341157	1.146593466	0.281319	4.075776	4.59E-05	0.000225857	up-regulated	protein_coding
PAPPA2	ENSMMUG00000023214	14.24599269	6.23825987	1.530803	4.075156	4.60E-05	0.000226303	up-regulated	protein_coding
BRD2	ENSMMUG00000023138	347.368613	-1.217259909	0.298775	-4.07417	4.62E-05	0.000227183	down-regulated	protein_coding
5S_rRNA	ENSMMUG00000036146	9.422224515	6.610866278	1.622746	4.073875	4.62E-05	0.000227393	up-regulated	rRNA
LAT2	ENSMMUG00000003840	21.18707059	3.552126495	0.872109	4.07303	4.64E-05	0.000228062	up-regulated	protein_coding
TMEM37	ENSMMUG00000011127	68.81311187	-2.872640463	0.705282	-4.07304	4.64E-05	0.000228062	down-regulated	protein_coding
MPP4	ENSMMUG00000018151	6.523776098	5.231110828	1.284521	4.07242	4.65E-05	0.000228581	up-regulated	protein_coding
VPS41	ENSMMUG00000010042	72.84520298	-3.316412172	0.814376	-4.07234	4.65E-05	0.000228585	down-regulated	protein_coding
SNRPB	ENSMMUG00000010050	213.161727	-3.419031428	0.839615	-4.07214	4.66E-05	0.000228685	down-regulated	protein_coding
ITGAV	ENSMMUG00000019462	65.75861559	-4.52811337	1.111993	-4.07207	4.66E-05	0.000228685	down-regulated	protein_coding
COPS6	ENSMMUG00000013440	66.84650008	-4.605014737	1.131263	-4.07069	4.69E-05	0.00022997	down-regulated	protein_coding

TFRC	ENSMMUG00000002114	528.4215979	-1.539422609	0.378253	-4.06983	4.70E-05	0.000230741	down-regulated	protein_coding
COX20	ENSMMUG00000031175	84.57871621	2.973336244	0.730892	4.068094	4.74E-05	0.000232381	up-regulated	protein_coding
PLA2G10	ENSMMUG00000009994	12.28929207	4.170292117	1.025222	4.067695	4.75E-05	0.000232699	up-regulated	protein_coding
THOC7	ENSMMUG00000012783	33.05475407	-5.41452824	1.331659	-4.066	4.78E-05	0.000234313	down-regulated	protein_coding
TNPO2	ENSMMUG00000005684	127.1279979	-1.853821768	0.456026	-4.06516	4.80E-05	0.000235075	down-regulated	protein_coding
WDR82	ENSMMUG00000008234	137.6940149	-2.23435794	0.549793	-4.064	4.82E-05	0.00023617	down-regulated	protein_coding
EMC2	ENSMMUG00000019260	47.50097386	-4.94870021	1.218239	-4.06218	4.86E-05	0.000237942	down-regulated	protein_coding
GPAA1	ENSMMUG00000007161	262.5253331	-1.955666003	0.481657	-4.06029	4.90E-05	0.000239793	down-regulated	protein_coding
GTF3C3	ENSMMUG00000008643	74.77014949	-2.432299457	0.599176	-4.05941	4.92E-05	0.000240529	down-regulated	protein_coding
TMEM202	ENSMMUG00000017854	16.39427355	4.330861265	1.067301	4.057768	4.95E-05	0.000242141	up-regulated	protein_coding
BCL2L14	ENSMMUG00000023315	125.1730852	-1.91249571	0.47133	-4.05766	4.96E-05	0.000242173	down-regulated	protein_coding
UBE3C	ENSMMUG00000008733	73.31931029	-3.100066581	0.764135	-4.05696	4.97E-05	0.000242723	down-regulated	protein_coding
MGMT	ENSMMUG00000011446	39.06617258	-5.655837618	1.394359	-4.05623	4.99E-05	0.000243406	down-regulated	protein_coding
ZBED5	ENSMMUG00000006652	48.95399603	-4.995211303	1.23166	-4.05567	5.00E-05	0.00024373	down-regulated	protein_coding
WDR26	ENSMMUG00000012227	166.0992565	-1.820438412	0.448872	-4.05558	5.00E-05	0.000243741	down-regulated	protein_coding
C11orf24	ENSMMUG00000019253	187.6546146	-1.411500629	0.3481	-4.05487	5.02E-05	0.000244401	down-regulated	protein_coding
RDH10	ENSMMUG00000005143	25.76929095	-5.057305702	1.247757	-4.05312	5.05E-05	0.000245987	down-regulated	protein_coding
HSD17B14	ENSMMUG00000028736	27.12834061	2.655563797	0.655244	4.052785	5.06E-05	0.000246253	up-regulated	protein_coding
NDUFS4	ENSMMUG00000004861	60.46018174	-3.747258392	0.924824	-4.05186	5.08E-05	0.000247142	down-regulated	protein_coding
ISCU	ENSMMUG00000017126	61.43509595	-4.458870207	1.100576	-4.0514	5.09E-05	0.000247548	down-regulated	protein_coding
NRG4	ENSMMUG00000020882	8.905990024	6.414078506	1.58331	4.051057	5.10E-05	0.000247823	up-regulated	protein_coding
ZC3HAV1	ENSMMUG00000006550	112.5637767	2.220347142	0.54815	4.050618	5.11E-05	0.000248203	up-regulated	protein_coding
PCCB	ENSMMUG00000003262	105.1749956	-2.729922282	0.674023	-4.05019	5.12E-05	0.000248572	down-regulated	protein_coding
CAPZA1	ENSMMUG00000015668	269.0322399	-2.504810088	0.618549	-4.04949	5.13E-05	0.000249059	down-regulated	protein_coding
KCNK4	ENSMMUG00000017802	20.64998399	2.984551764	0.737002	4.049585	5.13E-05	0.000249059	up-regulated	protein_coding
GNB1	ENSMMUG00000001896	759.7175949	-1.350469409	0.333525	-4.04908	5.14E-05	0.000249416	down-regulated	protein_coding
SEPX1	ENSMMUG00000007936	65.42376479	-4.572972042	1.129637	-4.04818	5.16E-05	0.00025029	down-regulated	protein_coding
ARHGAP29	ENSMMUG00000006369	102.0440626	-2.144715825	0.529973	-4.04684	5.19E-05	0.000251638	down-regulated	protein_coding
PGAP3	ENSMMUG00000001347	49.48900742	-5.009305115	1.23835	-4.04515	5.23E-05	0.000253377	down-regulated	protein_coding
RASSF3	ENSMMUG00000023229	29.07311712	-5.231938433	1.294032	-4.04313	5.27E-05	0.000255478	down-regulated	protein_coding
MLH3	ENSMMUG00000002911	49.90139427	1.814492327	0.44881	4.042899	5.28E-05	0.000255643	up-regulated	protein_coding
CAP2	ENSMMUG00000001990	5.29468473	5.185842223	1.283791	4.039476	5.36E-05	0.000259225	up-regulated	protein_coding
MS4A8B	ENSMMUG00000011166	2566.095985	-2.11645959	0.523935	-4.03954	5.36E-05	0.000259225	down-regulated	protein_coding
BRI3	ENSMMUG00000013275	213.4750373	-2.042359867	0.505622	-4.03931	5.36E-05	0.000259324	down-regulated	protein_coding
COG4	ENSMMUG00000003171	86.26209284	-2.100869571	0.520488	-4.03635	5.43E-05	0.000262434	down-regulated	protein_coding
SPRED2	ENSMMUG00000006012	111.9247733	-2.082248364	0.516103	-4.03456	5.47E-05	0.000264349	down-regulated	protein_coding
CUL2	ENSMMUG00000002469	25.58060565	-5.045571426	1.250931	-4.03345	5.50E-05	0.00026551	down-regulated	protein_coding
SUMO4	ENSMMUG00000007076	12.79621509	3.137005679	0.777824	4.033051	5.51E-05	0.000265873	up-regulated	protein_coding
C11H12ORF73	ENSMMUG00000031591	34.76136616	1.769695471	0.438842	4.032649	5.52E-05	0.000266237	up-regulated	protein_coding
POLR3C	ENSMMUG00000016735	70.37201532	1.834197909	0.454978	4.031397	5.54E-05	0.000267568	up-regulated	protein_coding
RSL24D1	ENSMMUG00000020693	70.76202809	-3.005971417	0.745757	-4.03077	5.56E-05	0.000268197	down-regulated	protein_coding
TMEM206	ENSMMUG00000002141	18.5235209	2.59275334	0.643263	4.030628	5.56E-05	0.000268263	up-regulated	protein_coding
CD46	ENSMMUG00000007129	96.48864279	-2.061373532	0.511521	-4.02989	5.58E-05	0.000269018	down-regulated	protein_coding
MRPL3	ENSMMUG00000000220	71.04968162	-2.796594354	0.69443	-4.02718	5.64E-05	0.000271946	down-regulated	protein_coding
RYBP	ENSMMUG00000007128	87.92849347	-3.377052266	0.838796	-4.02607	5.67E-05	0.000273137	down-regulated	protein_coding
C9orf16	ENSMMUG00000032017	67.20463386	-5.44392752	1.352674	-4.02457	5.71E-05	0.000274796	down-regulated	protein_coding
PHLDA3	ENSMMUG00000031350	68.56270654	2.113266013	0.525162	4.024026	5.72E-05	0.000275336	up-regulated	protein_coding
NUCB1	ENSMMUG00000003904	365.1430862	-1.387844972	0.344944	-4.02339	5.74E-05	0.000275966	down-regulated	protein_coding
MRPS2	ENSMMUG00000021597	87.61466574	-3.474038735	0.863474	-4.02333	5.74E-05	0.000275966	down-regulated	protein_coding
NPHP3	ENSMMUG00000021772	190.6146931	-2.029064283	0.504593	-4.02119	5.79E-05	0.000278385	down-regulated	protein_coding
SEC11A	ENSMMUG00000011486	84.38485154	-3.079827432	0.766038	-4.02046	5.81E-05	0.000279059	down-regulated	protein_coding
COX8A	ENSMMUG00000031143	292.8749974	-2.034008054	0.506015	-4.01966	5.83E-05	0.00027982	down-regulated	protein_coding
YRDC	ENSMMUG00000005372	132.5270504	2.435204158	0.606065	4.018057	5.87E-05	0.000281629	up-regulated	protein_coding
FBXW5	ENSMMUG00000011528	224.722545	-1.968713076	0.489975	-4.01798	5.87E-05	0.000281629	down-regulated	protein_coding
GPR98	ENSMMUG00000018976	44.14673494	2.521188425	0.627499	4.017836	5.87E-05	0.000281709	up-regulated	protein_coding
MRPS28	ENSMMUG00000022368	36.3530762	-5.551869989	1.381936	-4.01746	5.88E-05	0.000282067	down-regulated	protein_coding
PPP1R7	ENSMMUG00000016209	50.77756473	-5.02849996	1.25193	-4.0166	5.90E-05	0.000282999	down-regulated	protein_coding
PLD3	ENSMMUG00000006071	197.1784047	-2.006291182	0.499547	-4.01622	5.91E-05	0.000283176	down-regulated	protein_coding
PHB	ENSMMUG00000000156	393.4226963	-1.985395136	0.494362	-4.01608	5.92E-05	0.000283244	down-regulated	protein_coding
NUP133	ENSMMUG00000022091	57.66644913	-4.29087378	1.068614	-4.01536	5.94E-05	0.000284006	down-regulated	protein_coding
PRDM1	ENSMMUG00000021401	84.47466314	2.067889131	0.51521	4.013686	5.98E-05	0.000285936	up-regulated	protein_coding
PRAP1	ENSMMUG00000006323	280.7406526	-3.411094599	0.850609	-4.01018	6.07E-05	0.000290119	down-regulated	protein_coding
SLC37A4	ENSMMUG00000016890	147.7738163	-3.027252413	0.754915	-4.01006	6.07E-05	0.000290168	down-regulated	protein_coding
RBM12	ENSMMUG00000020964	89.56046053	-1.841257951	0.45924	-4.00936	6.09E-05	0.000290927	down-regulated	protein_coding
C2CD5	ENSMMUG00000006641	53.99969983	-4.233488675	1.05593	-4.00925	6.09E-05	0.000290964	down-regulated	protein_coding
PPIF	ENSMMUG00000003189	81.30963357	-2.82182431	0.704089	-4.00777	6.13E-05	0.000292698	down-regulated	protein_coding
PSMC4	ENSMMUG00000017188	93.60727502	-2.657956294	0.663266	-4.00738	6.14E-05	0.000292889	down-regulated	protein_coding
ABCA8	ENSMMUG00000018587	14.13272033	7.356972424	1.836088	4.006874	6.15E-05	0.000293412	up-regulated	protein_coding
ASB16	ENSMMUG00000016867	10.49056904	5.504425077	1.374059	4.005959	6.18E-05	0.000294386	up-regulated	protein_coding
SLC12A6	ENSMMUG00000020935	62.64274581	-3.485277213	0.870029	-4.00593	6.18E-05	0.000294386	down-regulated	protein_coding
DBI	ENSMMUG00000011126	228.2563559	-2.088811581	0.521504	-4.00536	6.19E-05	0.000295001	down-regulated	protein_coding
UBE2I	ENSMMUG00000003654	86.74664103	-3.686376115	0.920448	-4.00498	6.20E-05	0.000295377	down-regulated	protein_coding
TTYH3	ENSMMUG00000019005	342.1230241	-1.241590118	0.310028	-4.00477	6.21E-05	0.000295533	down-regulated	protein_coding
RAB40C	ENSMMUG00000013198	47.11066372	-4.916874509	1.227842	-4.00449	6.22E-05	0.000295794	down-regulated	protein_coding
WDR11	ENSMMUG00000001118	65.42421831	-2.232047569	0.557469	-4.00389	6.23E-05	0.000296436	down-regulated	protein_coding
SYN3	ENSMMUG00000006732	6.349066358	4.952419341	1.237244	4.002782	6.26E-05	0.000297733	up-regulated	protein_coding
KLHL9	ENSMMUG00000010959	47.95567879	-4.963926891	1.240152	-4.00268	6.26E-05	0.000297767	down-regulated	protein_coding
ARHGAP6	ENSMMUG00000003896	8.379246334	4.163309119	1.040413	4.001592	6.29E-05	0.000299034	up-regulated	protein_coding
RNF213	ENSMMUG00000005455	471.2835338	2.170715712	0.542535	4.001062	6.31E-05	0.000299603	up-regulated	protein_coding
SCARB1	ENSMMUG00000016486	137.5179119	-2.156564624	0.539119	-4.00017	6.33E-05	0.000300637	down-regulated	protein_coding
MACROD1	ENSMMUG00000000479	27.10761644	-5.12827853	1.282623	-3.99828	6.38E-05	0.000302949	down-regulated	protein_coding
LDOC1L	ENSMMUG00000014063	51.86552055	2.673369549	0.66878	3.997382	6.40E-05	0.000303992	up-regulated	protein_coding
ITGA3	ENSMMUG00000000153	381.4624756	-1.185232911	0.296674	-3.99507	6.47E-05	0.000306874	down-regulated	protein_coding
IBTK	ENSMMUG00000010749	77.63520747	-2.668262133	0.667987	-3.99448	6.48E-05	0.000307527	down-regulated	protein_coding
RECQL4	ENSMMUG00000015588	200.6981084	3.10307229	0.777064	3.993327	6.52E-05	0.000308877	up-regulated	protein_coding
SEP15	ENSMMUG00000016554	51.99756451	-4.214117594	1.0553	-3.99329	6.52E-05	0.000308877	down-regulated	protein_coding
GLTPD2	ENSMMUG00000012998	13.10316265	4.213394755	1.055207	3.992957	6.53E-05	0.000309205	up-regulated	protein_coding
PSMB2	ENSMMUG00000013090	100.8907771	-2.837104229	0.710577	-3.99268	6.53E-05	0.000309468	down-regulated	protein_coding
HIST2H2AC	ENSMMUG00000031744	183.8713808	3.08497903	0.772718	3.992372	6.54E-05	0.000309761	up-regulated	protein_coding
RHOU	ENSMMUG00000002924	85.43450744	-2.561586922	0.641652	-3.99217	6.55E-05	0.000309915	down-regulated	protein_coding
ENPP3	ENSMMUG00000017549	6.633425738	5.651111985	1.415575	3.992096	6.55E-05	0.000309915	up-regulated	protein_coding
CELF1	ENSMMUG00000011657	72.15866017	-2.215641188	0.555198	-3.99073	6.59E-05	0.000311606	down-regulated	protein_coding
PARK7	ENSMMUG00000019671	82.10871038	-3.894798492	0.97616	-3.98992	6.61E-05	0.000312566	down-regulated	protein_coding
ENPP7	ENSMMUG00000016414	6.633155014	5.039296449	1.263117	3.989572	6.62E-05	0.000312916	up-regulated	protein_coding
NUTF2	ENSMMUG00000012038	75.57062214	-3.786262615	0.949094	-3.98934	6.63E-05	0.000313115	down-regulated	protein_coding
CLDND1	ENSMMUG00000009549	59.32095999	-3.302539115	0.827875	-3.98918	6.63E-05	0.000313228	down-regulated	protein_coding
CAMK1G	ENSMMUG00000003369	14.89110019	6.118085987	1.534252	3.987668	6.67E-05	0.000315122	up-regulated	protein_coding
RAPGEFL1	ENSMMUG00000015160	121.6513676	-1.625757046	0.407721	-3.98742	6.68E-05	0.000315343	down-regulated	protein_coding
SF3B5	ENSMMUG00000009434	107.9722768	-3.70210868	0.92855	-3.98698	6.69E-05	0.00031583	down-regulated	protein_coding
HIAT1	ENSMMUG00000004238	44.56727325	-4.85731756	1.218491	-3.98634	6.71E-05	0.000316574	down-regulated	protein_coding

CLU	ENSMMUG00000021516	89.42290456	-2.988437539	0.749685	-3.98626	6.71E-05	0.000316575	down-regulated	protein_coding
U2SURP	ENSMMUG00000002775	85.3660875	-2.382959266	0.597899	-3.98556	6.73E-05	0.000317303	down-regulated	protein_coding
SLC25A39	ENSMMUG00000017944	156.7439864	-2.612711985	0.655535	-3.98562	6.73E-05	0.000317303	down-regulated	protein_coding
SLCO5A1	ENSMMUG00000008783	14.37295951	2.599236859	0.652182	3.985446	6.74E-05	0.000317344	up-regulated	protein_coding
ANO9	ENSMMUG00000031293	188.8856226	-1.384927215	0.347505	-3.98535	6.74E-05	0.000317369	down-regulated	protein_coding
GOLGA5	ENSMMUG00000015169	53.84662843	-4.275027777	1.072851	-3.98474	6.76E-05	0.000318084	down-regulated	protein_coding
FAM49B	ENSMMUG00000015348	51.79977981	-4.207424976	1.055997	-3.98431	6.77E-05	0.000318543	down-regulated	protein_coding
GP1BA	ENSMMUG00000002763	16.01665374	5.415838289	1.359578	3.983471	6.79E-05	0.000319372	up-regulated	protein_coding
HIST1H2AH	ENSMMUG00000029905	72.02806969	8.978999598	2.254116	3.98338	6.79E-05	0.000319372	up-regulated	protein_coding
ESYT2	ENSMMUG00000022361	232.0151134	-1.665066022	0.418098	-3.98247	6.82E-05	0.000320485	down-regulated	protein_coding
SFXN1	ENSMMUG00000005148	26.49652965	-5.096528106	1.279774	-3.98236	6.82E-05	0.000320527	down-regulated	protein_coding
SENP6	ENSMMUG00000004491	49.26423499	-3.530223363	0.886537	-3.98204	6.83E-05	0.000320753	down-regulated	protein_coding
SP2	ENSMMUG00000004765	79.10661924	1.527572409	0.383613	3.982066	6.83E-05	0.000320753	up-regulated	protein_coding
RAD51D	ENSMMUG00000000750	108.594433	1.596476329	0.401026	3.98098	6.86E-05	0.000321937	up-regulated	protein_coding
MON2	ENSMMUG00000000956	49.56312011	-5.01241812	1.259108	-3.98093	6.86E-05	0.000321937	down-regulated	protein_coding
STX5	ENSMMUG00000005280	55.93892171	-3.165054703	0.795237	-3.98001	6.89E-05	0.000323071	down-regulated	protein_coding
CSDA	ENSMMUG00000022469	175.7361321	-2.171584415	0.54589	-3.97806	6.95E-05	0.000325625	down-regulated	protein_coding
PLEKHF2	ENSMMUG00000014183	23.9271623	-4.950509764	1.244904	-3.97662	6.99E-05	0.000327499	down-regulated	protein_coding
WFDC2	ENSMMUG00000002751	66.03145398	-4.463741335	1.122522	-3.97653	6.99E-05	0.000327513	down-regulated	protein_coding
MAP6	ENSMMUG00000007676	41.88175147	2.821161831	0.709501	3.97626	7.00E-05	0.000327775	up-regulated	protein_coding
DDC	ENSMMUG00000019991	698.5966528	-2.173168519	0.546553	-3.97613	7.00E-05	0.000327841	down-regulated	protein_coding
ZFP112	ENSMMUG00000012802	13.23195825	2.845701898	0.715802	3.975541	7.02E-05	0.00032855	up-regulated	protein_coding
DHX40	ENSMMUG00000022042	48.92861405	-4.993341379	1.256305	-3.97462	7.05E-05	0.00032971	down-regulated	protein_coding
TTC37	ENSMMUG00000001205	137.6238443	-1.948240502	0.490182	-3.97453	7.05E-05	0.000329737	down-regulated	protein_coding
PRDX6	ENSMMUG00000008776	89.83777857	-2.369734829	0.596486	-3.97283	7.10E-05	0.000331878	down-regulated	protein_coding
AXDND1	ENSMMUG00000003769	10.8737236	6.114482271	1.539278	3.972306	7.12E-05	0.000332496	up-regulated	protein_coding
LIPA	ENSMMUG00000022551	108.3632888	-3.982404134	1.002659	-3.97184	7.13E-05	0.000333033	down-regulated	protein_coding
SERINC3	ENSMMUG00000013969	134.8813193	-2.114179943	0.532313	-3.97169	7.14E-05	0.000333143	down-regulated	protein_coding
SEC61G	ENSMMUG00000008366	31.39567304	-5.340260243	1.344646	-3.9715	7.14E-05	0.000333296	down-regulated	protein_coding
TIMM13	ENSMMUG00000001191	85.7839138	-4.328667089	1.090292	-3.97019	7.18E-05	0.000334907	down-regulated	protein_coding
HTR4	ENSMMUG00000016913	13.69053661	3.873905764	0.975736	3.970242	7.18E-05	0.000334907	up-regulated	protein_coding
WHAMM	ENSMMUG00000003498	85.58531505	2.436972276	0.613882	3.969772	7.19E-05	0.000335376	up-regulated	protein_coding
C11ORF61	ENSMMUG00000008488	38.62774313	2.231334152	0.562091	3.969701	7.20E-05	0.000335376	up-regulated	protein_coding
MAML3	ENSMMUG00000021998	92.69993167	2.233713917	0.562775	3.969109	7.21E-05	0.0003361	up-regulated	protein_coding
TNFRSF1A	ENSMMUG00000000377	277.562551	-1.561352672	0.393466	-3.9682	7.24E-05	0.000337267	down-regulated	protein_coding
CTSV	ENSMMUG00000022971	140.9055367	-3.032563987	0.764315	-3.96769	7.26E-05	0.000337888	down-regulated	protein_coding
GABARAPL1	ENSMMUG00000002197	103.4640424	-2.3388705	0.589796	-3.96556	7.32E-05	0.000340803	down-regulated	protein_coding
VPS4B	ENSMMUG00000021184	124.1183357	-2.282128467	0.575682	-3.96422	7.36E-05	0.000342504	down-regulated	protein_coding
JMJD6	ENSMMUG00000022411	24.04409432	-4.956354574	1.250372	-3.9639	7.37E-05	0.000342726	down-regulated	protein_coding
IL23A	ENSMMUG00000031831	24.2494696	2.736444531	0.690338	3.963919	7.37E-05	0.000342726	up-regulated	protein_coding
TET3	ENSMMUG00000020551	98.08965692	2.079744527	0.52478	3.96308	7.40E-05	0.0003438	up-regulated	protein_coding
CHID1	ENSMMUG00000003618	92.56303015	-2.908415969	0.734262	-3.961	7.46E-05	0.000346689	down-regulated	protein_coding
TMA7	ENSMMUG00000031887	31.88714915	-5.362618326	1.354175	-3.96006	7.49E-05	0.000347942	down-regulated	protein_coding
ARMCX3	ENSMMUG00000015655	93.44983489	-2.575414734	0.650496	-3.95915	7.52E-05	0.000349158	down-regulated	protein_coding
SPINK1	ENSMMUG00000017227	33.49152797	-5.434001505	1.372826	-3.95826	7.55E-05	0.000350237	down-regulated	protein_coding
PSMC5	ENSMMUG00000016769	156.151248	-2.014399985	0.50902	-3.95741	7.58E-05	0.000351251	down-regulated	protein_coding
NECAP2	ENSMMUG00000019940	92.34045515	-1.872165026	0.473099	-3.95724	7.58E-05	0.000351389	down-regulated	protein_coding
WNK4	ENSMMUG00000016616	23.56583915	2.847686724	0.719882	3.955771	7.63E-05	0.000353439	up-regulated	protein_coding
UBE2R2	ENSMMUG00000012594	45.93588342	-4.903223871	1.24016	-3.9537	7.70E-05	0.000356395	down-regulated	protein_coding
SRP68	ENSMMUG00000001795	118.92057	-1.919368867	0.485475	-3.95359	7.70E-05	0.000356439	down-regulated	protein_coding
FKBP1A	ENSMMUG00000015996	332.3659507	-1.624086058	0.410814	-3.95333	7.71E-05	0.000356711	down-regulated	protein_coding
CISD2	ENSMMUG00000011204	45.13872418	-4.873324796	1.23315	-3.95193	7.75E-05	0.000358689	down-regulated	protein_coding
BCAM	ENSMMUG00000003273	142.401284	-2.19761659	0.556109	-3.95177	7.76E-05	0.000358811	down-regulated	protein_coding
TSPAN13	ENSMMUG00000017049	118.325605	-2.042112652	0.516877	-3.95086	7.79E-05	0.00035994	down-regulated	protein_coding
ANAPC4	ENSMMUG00000004458	25.27085962	-5.027022192	1.272539	-3.95039	7.80E-05	0.000360495	down-regulated	protein_coding
TCIRG1	ENSMMUG00000023190	55.32211053	-4.309579058	1.090939	-3.95034	7.80E-05	0.000360495	down-regulated	protein_coding
SNRK	ENSMMUG00000001028	133.3925418	1.633120563	0.413635	3.948212	7.87E-05	0.000363474	up-regulated	protein_coding
INO80E	ENSMMUG00000020276	164.0939449	2.144016475	0.543029	3.948256	7.87E-05	0.000363474	up-regulated	protein_coding
SNRPE	ENSMMUG00000031372	35.38965611	-5.513274254	1.396893	-3.94681	7.92E-05	0.000365485	down-regulated	protein_coding
CCDC153	ENSMMUG00000012191	20.24365309	3.873291318	0.981569	3.946021	7.95E-05	0.000366457	up-regulated	protein_coding
HMGA2	ENSMMUG00000022660	8.656679606	4.134502622	1.047762	3.946032	7.95E-05	0.000366457	up-regulated	protein_coding
IGLON5	ENSMMUG00000023529	5.463791395	5.148448505	1.30488	3.945534	7.96E-05	0.000367082	up-regulated	protein_coding
SH3RF3	ENSMMUG00000017175	24.40944286	2.974010163	0.753853	3.945082	7.98E-05	0.000367656	up-regulated	protein_coding
CSNK1D	ENSMMUG00000018329	143.9180129	-1.366351324	0.346517	-3.94311	8.04E-05	0.00037058	down-regulated	protein_coding
ESYT1	ENSMMUG00000021979	217.1464414	-1.733908014	0.439818	-3.94233	8.07E-05	0.000371658	down-regulated	protein_coding
SPATA5L1	ENSMMUG00000005858	52.55001553	1.833836071	0.465239	3.94171	8.09E-05	0.000372379	up-regulated	protein_coding
PRKG1	ENSMMUG00000010844	7.912193328	5.648296198	1.432952	3.941721	8.09E-05	0.000372379	up-regulated	protein_coding
TRIM36	ENSMMUG00000011130	69.24727558	-2.42350813	0.615086	-3.94011	8.14E-05	0.000374622	down-regulated	protein_coding
DNAJB9	ENSMMUG00000011833	26.48844792	-5.097458221	1.293714	-3.94017	8.14E-05	0.000374622	down-regulated	protein_coding
SEPT11	ENSMMUG00000019901	92.88336035	-1.959829236	0.497455	-3.93971	8.16E-05	0.000375132	down-regulated	protein_coding
C2H3ORF18	ENSMMUG00000010499	16.86382998	2.897762668	0.735633	3.939143	8.18E-05	0.000375896	up-regulated	protein_coding
SLC25A37	ENSMMUG00000016662	59.47069372	-2.528709222	0.641972	-3.93897	8.18E-05	0.000376042	down-regulated	protein_coding
TAX1BP3	ENSMMUG00000023390	467.666396	-1.185721631	0.301084	-3.93817	8.21E-05	0.000377172	down-regulated	protein_coding
FMO5	ENSMMUG00000000476	57.91423434	-5.24090659	1.330918	-3.93781	8.22E-05	0.000377616	down-regulated	protein_coding
SLC35A3	ENSMMUG00000000931	119.2391003	-2.978653979	0.756608	-3.93685	8.26E-05	0.000379009	down-regulated	protein_coding
SNRPF	ENSMMUG00000009302	37.7994969	-5.608250712	1.424683	-3.93649	8.27E-05	0.000379457	down-regulated	protein_coding
DDX58	ENSMMUG00000012442	49.31268867	2.650205845	0.673314	3.936064	8.28E-05	0.000380009	up-regulated	protein_coding
RPL17-C18ORF32	ENSMMUG00000018837	922.7494494	-2.149493185	0.546165	-3.93561	8.30E-05	0.000380601	down-regulated	protein_coding
HEG1	ENSMMUG00000001168	29.15110277	2.56342549	0.651542	3.934395	8.34E-05	0.00038241	up-regulated	protein_coding
SUPT4H1	ENSMMUG00000007579	46.35859823	-4.89304755	1.243726	-3.93418	8.35E-05	0.000382623	down-regulated	protein_coding
SENP5	ENSMMUG00000002259	69.43933635	1.550189256	0.394274	3.931756	8.43E-05	0.000386383	up-regulated	protein_coding
GP6	ENSMMUG00000003117	10.78587829	6.116063025	1.55619	3.930151	8.49E-05	0.000388845	up-regulated	protein_coding
ISCA1	ENSMMUG00000003399	95.8987146	-2.116992074	0.538702	-3.9298	8.50E-05	0.000389283	down-regulated	protein_coding
PACSIN1	ENSMMUG00000007811	7.140564411	4.272659038	1.087326	3.929512	8.51E-05	0.000389629	up-regulated	protein_coding
RREB1	ENSMMUG00000007852	254.3247942	1.86593258	0.475094	3.927503	8.58E-05	0.000392769	up-regulated	protein_coding
PCSK9	ENSMMUG00000005736	246.03841	-2.863438402	0.729115	-3.92728	8.59E-05	0.000393004	down-regulated	protein_coding
C9ORF40	ENSMMUG00000031678	18.8978871	2.806016048	0.714568	3.926869	8.61E-05	0.00039355	up-regulated	protein_coding
TFPT	ENSMMUG00000022296	59.45623836	2.093439245	0.533135	3.926662	8.61E-05	0.000393762	up-regulated	protein_coding
ZAR1	ENSMMUG00000006704	7.621209049	4.054963407	1.032902	3.925796	8.64E-05	0.000395055	up-regulated	protein_coding
MRPL4	ENSMMUG00000001219	70.06474788	-4.684776401	1.193412	-3.92553	8.65E-05	0.000395256	down-regulated	protein_coding
FAM107B	ENSMMUG00000017169	78.3821791	-3.017572408	0.768707	-3.92552	8.65E-05	0.000395256	down-regulated	protein_coding
SAMD14	ENSMMUG00000014687	17.46211061	3.69457511	0.941576	3.923822	8.72E-05	0.000397921	up-regulated	protein_coding
KDELR2	ENSMMUG00000030415	273.4955081	-1.45796588	0.371606	-3.92342	8.73E-05	0.000398453	down-regulated	protein_coding
FA2H	ENSMMUG00000008381	193.5916125	-2.113483092	0.538758	-3.92288	8.75E-05	0.000399224	down-regulated	protein_coding
U6	ENSMMUG00000037376	5.982478486	6.095991926	1.554168	3.922351	8.77E-05	0.000399972	up-regulated	snRNA
EIF3D	ENSMMUG00000012754	258.0468682	-2.214850894	0.564781	-3.92161	8.80E-05	0.000401079	down-regulated	protein_coding
RPL28	ENSMMUG00000015901	420.9268294	-2.616708249	0.667284	-3.92143	8.80E-05	0.000401244	down-regulated	protein_coding
HIST1H3A	ENSMMUG00000001439	7.547443405	4.462043309	1.138467	3.919343	8.88E-05	0.000404605	up-regulated	protein_coding
ERP44	ENSMMUG00000011415	126.6563237	-1.437998195	0.366931	-3.91898	8.89E-05	0.00040508	down-regulated	protein_coding
SH3GLB1	ENSMMUG00000013487	61.20395465	-2.280473423	0.582006	-3.9183	8.92E-05	0.000406096	down-regulated	protein_coding

TMEM68	ENSMMUG00000005477	23.96592488	-4.953136296	1.264964	-3.91563	9.02E-05	0.000410348	down-regulated	protein_coding
TOMM20	ENSMMUG00000007136	128.855668	-2.967408453	0.757836	-3.91564	9.02E-05	0.000410348	down-regulated	protein_coding
HNRNPK	ENSMMUG00000018871	700.1418218	-1.047961835	0.267655	-3.91535	9.03E-05	0.000410699	down-regulated	protein_coding
RPS17L	ENSMMUG00000011327	581.8494678	-2.683215936	0.685464	-3.91445	9.06E-05	0.000412101	down-regulated	protein_coding
FAM188B	ENSMMUG00000011761	22.80015287	3.119819474	0.797071	3.914107	9.07E-05	0.000412555	up-regulated	protein_coding
MYRIP	ENSMMUG00000017701	23.03076613	2.54323112	0.649816	3.913774	9.09E-05	0.000412993	up-regulated	protein_coding
SATL1	ENSMMUG00000004336	11.20090859	5.934896708	1.516564	3.913384	9.10E-05	0.000413526	up-regulated	protein_coding
RTN3	ENSMMUG00000015876	473.714254	1.078831696	0.275739	3.912514	9.13E-05	0.000414887	up-regulated	protein_coding
PPP1R14D	ENSMMUG00000001724	57.16126584	-4.370612104	1.117384	-3.91147	9.17E-05	0.000416552	down-regulated	protein_coding
NDUFAF4	ENSMMUG00000018843	24.59296552	-4.987704425	1.275567	-3.91019	9.22E-05	0.000418637	down-regulated	protein_coding
KHK	ENSMMUG00000016047	327.3663909	-2.795282898	0.714945	-3.90979	9.24E-05	0.000419192	down-regulated	protein_coding
ACIN1	ENSMMUG00000012979	566.8589755	1.392483377	0.356384	3.907254	9.34E-05	0.000423206	up-regulated	protein_coding
MAP3K11	ENSMMUG00000007404	301.3121968	-1.336750049	0.342351	-3.90462	9.44E-05	0.000427572	down-regulated	protein_coding
LGMN	ENSMMUG00000013966	862.9723371	-2.856722596	0.731749	-3.90397	9.46E-05	0.000428589	down-regulated	protein_coding
SESTD1	ENSMMUG00000019690	59.07848794	-2.942039787	0.753637	-3.90379	9.47E-05	0.000428762	down-regulated	protein_coding
ACHE	ENSMMUG00000021257	188.1344454	-1.578710302	0.404516	-3.90272	9.51E-05	0.000430527	down-regulated	protein_coding
GNG12	ENSMMUG00000014807	305.4840443	-2.237446471	0.573418	-3.90194	9.54E-05	0.000431632	down-regulated	protein_coding
RAB6B	ENSMMUG00000008919	42.51593964	-4.78637927	1.227258	-3.90006	9.62E-05	0.000434866	down-regulated	protein_coding
RHBDD1	ENSMMUG00000000722	22.24802051	-4.844324535	1.242568	-3.89864	9.67E-05	0.000437147	down-regulated	protein_coding
LYN	ENSMMUG00000014149	111.130537	-1.792528339	0.459783	-3.89864	9.67E-05	0.000437147	down-regulated	protein_coding
UBA5	ENSMMUG00000021771	23.67521027	-4.933605933	1.265516	-3.89849	9.68E-05	0.000437271	down-regulated	protein_coding
PRC1	ENSMMUG00000005109	103.9060057	2.332393098	0.598445	3.897422	9.72E-05	0.000438912	up-regulated	protein_coding
HEXIM1	ENSMMUG00000010481	308.6847336	1.547506487	0.397066	3.897353	9.72E-05	0.000438912	up-regulated	protein_coding
CCDC36	ENSMMUG00000022607	6.091785139	5.771767902	1.480924	3.897409	9.72E-05	0.000438912	up-regulated	protein_coding
ANO6	ENSMMUG00000011811	292.4904066	-2.473711479	0.635142	-3.89474	9.83E-05	0.00044353	down-regulated	protein_coding
KIFAP3	ENSMMUG00000010081	65.84068121	-3.38941412	0.870331	-3.8944	9.84E-05	0.000444018	down-regulated	protein_coding
POLR2G	ENSMMUG00000014368	68.70857719	-3.006515673	0.772202	-3.89343	9.88E-05	0.000445647	down-regulated	protein_coding
RHOT2	ENSMMUG00000022010	154.7085335	-2.516454247	0.646352	-3.89332	9.89E-05	0.000445707	down-regulated	protein_coding
SYNJ2BP	ENSMMUG00000011086	22.30962966	-4.848896016	1.246083	-3.89131	9.97E-05	0.000449272	down-regulated	protein_coding
OCIAD2	ENSMMUG00000014869	50.74947388	-4.144358298	1.065132	-3.89093	9.99E-05	0.000449825	down-regulated	protein_coding
LTA4H	ENSMMUG00000009312	210.3165137	-2.19394685	0.563952	-3.89031	0.0001001	0.000450844	down-regulated	protein_coding
TMEM120A	ENSMMUG00000020087	223.3331603	-1.691279847	0.434772	-3.89003	0.0001002	0.000451209	down-regulated	protein_coding
HIST1H3A	ENSMMUG00000023731	10.18256309	4.741341451	1.219307	3.888554	0.0001008	0.000453826	up-regulated	protein_coding
DPM2	ENSMMUG00000014374	53.52448804	-5.123725033	1.31775	-3.88824	0.000101	0.000454276	down-regulated	protein_coding
STAR	ENSMMUG00000013676	9.094519495	5.475900333	1.40931	3.885518	0.0001021	0.000459243	up-regulated	protein_coding
TMPRSS15	ENSMMUG00000021860	76.82241279	-5.642178733	1.45221	-3.88523	0.0001022	0.000459633	down-regulated	protein_coding
TBX6	ENSMMUG00000000981	6.697355595	4.077937441	1.049706	3.884838	0.0001024	0.000460121	up-regulated	protein_coding
ONECUT2	ENSMMUG00000023218	44.11904884	2.774126765	0.714093	3.884823	0.0001024	0.000460121	up-regulated	protein_coding
BLOC1S2	ENSMMUG00000010827	45.45759023	-4.88340345	1.257875	-3.88226	0.0001035	0.000464547	down-regulated	protein_coding
NARS	ENSMMUG00000019974	178.5946915	-2.223321519	0.572999	-3.88015	0.0001044	0.000468454	down-regulated	protein_coding
ZNF772	ENSMMUG00000014285	10.38406905	4.089886013	1.054102	3.879973	0.0001045	0.00046865	up-regulated	protein_coding
LBX2	ENSMMUG00000002125	11.15666711	5.797066078	1.495157	3.877229	0.0001057	0.000473664	up-regulated	protein_coding
SMEK2	ENSMMUG00000007001	80.99892039	-2.107629452	0.543649	-3.87682	0.0001058	0.000474309	down-regulated	protein_coding
TMEM184B	ENSMMUG00000019390	206.6185682	1.601825615	0.413222	3.876431	0.000106	0.000474917	up-regulated	protein_coding
RPL17	ENSMMUG00000031768	6.826882008	7.299302794	1.883578	3.875233	0.0001065	0.00047696	up-regulated	protein_coding
FAM43A	ENSMMUG00000010709	107.5424503	1.800533186	0.464843	3.873419	0.0001073	0.000480372	up-regulated	protein_coding
PDK2	ENSMMUG00000014685	23.3568384	-4.914568108	1.268822	-3.87333	0.0001074	0.000480393	down-regulated	protein_coding
MCTP1	ENSMMUG00000001397	6.483395939	4.760673127	1.229136	3.873185	0.0001074	0.00048053	up-regulated	protein_coding
NSL1	ENSMMUG00000019287	21.75912859	-4.812263065	1.243214	-3.87082	0.0001085	0.000485054	down-regulated	protein_coding
TPR	ENSMMUG00000009081	87.49937688	2.102020626	0.543401	3.868266	0.0001096	0.000490016	up-regulated	protein_coding
C1orf174	ENSMMUG00000015176	22.90927179	-4.885750921	1.263117	-3.86801	0.0001097	0.000490372	down-regulated	protein_coding
ATP6AP2	ENSMMUG00000012762	102.9179309	-1.954117412	0.50523	-3.86778	0.0001098	0.000490681	down-regulated	protein_coding
LGALS3BP	ENSMMUG00000019662	271.2189323	-2.00705556	0.518927	-3.8677	0.0001099	0.000490681	down-regulated	protein_coding
POLR2D	ENSMMUG00000001989	25.27997818	-5.028174475	1.300201	-3.86723	0.0001101	0.000491486	down-regulated	protein_coding
LRRC40	ENSMMUG00000021921	48.38957903	-4.958383625	1.282347	-3.86665	0.0001103	0.0004925	down-regulated	protein_coding
RPS3	ENSMMUG00000009019	1031.081915	-2.898508756	0.749656	-3.86645	0.0001104	0.000492741	down-regulated	protein_coding
TMEM223	ENSMMUG00000023094	98.4128653	2.060778526	0.533036	3.866113	0.0001106	0.000493269	up-regulated	protein_coding
N4BP2L2	ENSMMUG00000007200	55.17921303	-2.640411914	0.683216	-3.86468	0.0001112	0.000495928	down-regulated	protein_coding
TSPAN15	ENSMMUG00000015314	111.2626047	-2.101894431	0.54401	-3.8637	0.0001117	0.000497537	down-regulated	protein_coding
MRPS27	ENSMMUG00000017905	22.34967708	-4.8501335	1.255607	-3.86278	0.0001121	0.000499265	down-regulated	protein_coding
GPR89B	ENSMMUG00000019640	86.08988428	1.426162958	0.369365	3.86112	0.0001129	0.000502352	up-regulated	protein_coding
RNF19A	ENSMMUG00000004387	47.31042935	-4.946498219	1.281153	-3.86097	0.0001129	0.000502496	down-regulated	protein_coding
IFI30	ENSMMUG00000003163	241.4815546	-1.698340093	0.439975	-3.86009	0.0001133	0.000504004	down-regulated	protein_coding
POU2F1	ENSMMUG00000008155	20.05507177	2.843055107	0.73673	3.859017	0.0001138	0.000505738	up-regulated	protein_coding
ANP32A	ENSMMUG00000022717	166.4477329	-1.780113422	0.461366	-3.85836	0.0001142	0.000506789	down-regulated	protein_coding
MOCS2	ENSMMUG00000020449	64.96654472	-2.31535358	0.600126	-3.85811	0.0001143	0.000507134	down-regulated	protein_coding
UBL5	ENSMMUG00000007877	70.02499631	-3.864988911	1.001933	-3.85753	0.0001145	0.000508182	down-regulated	protein_coding
GPR137C	ENSMMUG00000006998	8.741035977	4.268743203	1.107254	3.855251	0.0001156	0.000512784	up-regulated	protein_coding
MRPL51	ENSMMUG00000018676	66.10586576	-4.598669722	1.192902	-3.85503	0.0001157	0.000513092	down-regulated	protein_coding
PSMB6	ENSMMUG00000012999	102.9943957	-3.199849684	0.830075	-3.85489	0.0001158	0.000513217	down-regulated	protein_coding
HELZ	ENSMMUG00000002959	95.76046878	1.648020977	0.427529	3.854761	0.0001158	0.000513328	up-regulated	protein_coding
DNAJC21	ENSMMUG00000000446	47.64894226	-4.050458556	1.05079	-3.85468	0.0001159	0.00051334	down-regulated	protein_coding
ATP4B	ENSMMUG00000007425	7.913961734	6.21727223	1.613095	3.85425	0.0001161	0.000514081	up-regulated	protein_coding
DBT	ENSMMUG00000007706	25.45790095	-5.040139101	1.307724	-3.85413	0.0001161	0.000514173	down-regulated	protein_coding
MOBKL3	ENSMMUG00000016547	55.1565114	-2.354713297	0.611064	-3.85347	0.0001165	0.000515408	down-regulated	protein_coding
GLYCTK	ENSMMUG00000016971	94.52905757	-2.449019078	0.635618	-3.85297	0.0001167	0.000516128	down-regulated	protein_coding
ARCN1	ENSMMUG00000002543	117.8131922	-2.478752522	0.643425	-3.85243	0.000117	0.000517105	down-regulated	protein_coding
SIRT6	ENSMMUG00000019955	66.58474465	-3.39010698	0.880218	-3.85144	0.0001174	0.000519038	down-regulated	protein_coding
PPIB	ENSMMUG00000021123	275.9575139	-2.443096364	0.63449	-3.85049	0.0001179	0.000520904	down-regulated	protein_coding
ARSG	ENSMMUG00000007306	8.843738513	4.8969434	1.272383	3.848638	0.0001188	0.000524357	up-regulated	protein_coding
MARK2	ENSMMUG00000014131	165.4915945	-1.296544456	0.336927	-3.84814	0.000119	0.000525258	down-regulated	protein_coding
LAMA3	ENSMMUG00000004321	408.0494805	-1.636903839	0.425521	-3.84682	0.0001197	0.000527764	down-regulated	protein_coding
POLR2B	ENSMMUG00000014008	131.0014668	-1.591098466	0.413635	-3.84663	0.0001198	0.000528022	down-regulated	protein_coding
AP2M1	ENSMMUG00000006557	416.5687967	-1.130045063	0.293784	-3.84652	0.0001198	0.000528095	down-regulated	protein_coding
VPS8	ENSMMUG00000000425	53.00230056	-2.741694192	0.712972	-3.84544	0.0001203	0.000530241	down-regulated	protein_coding
CASP4	ENSMMUG00000009850	53.92589464	-3.241133443	0.843142	-3.84411	0.000121	0.000532965	down-regulated	protein_coding
HSPE1	ENSMMUG00000010491	479.2852037	2.192823944	0.570452	3.844009	0.000121	0.000533022	up-regulated	protein_coding
KDSR	ENSMMUG00000006578	60.54904972	-3.259656186	0.848041	-3.84375	0.0001212	0.000533426	down-regulated	protein_coding
SQRDL	ENSMMUG00000015749	143.4238554	-1.901494034	0.494722	-3.84356	0.0001213	0.000533669	down-regulated	protein_coding
TTC39A	ENSMMUG00000004564	41.28009072	2.152577027	0.560125	3.843028	0.0001215	0.000534659	up-regulated	protein_coding
EFNA2	ENSMMUG00000022105	56.36314507	-2.418607077	0.629381	-3.84284	0.0001216	0.000534914	down-regulated	protein_coding
PPP1R16A	ENSMMUG00000015582	212.2746644	-1.737811812	0.452231	-3.84275	0.0001217	0.000534928	down-regulated	protein_coding
IARS	ENSMMUG00000004628	172.4098884	-1.600720119	0.416572	-3.8426	0.0001217	0.000535099	down-regulated	protein_coding
NT5C3B	ENSMMUG00000013056	26.24395394	-5.081554756	1.322603	-3.84209	0.000122	0.000535884	down-regulated	protein_coding
5S_rRNA	ENSMMUG00000024087	10.23420831	5.616515868	1.461839	3.842089	0.000122	0.000535884	up-regulated	rRNA
HECTD3	ENSMMUG00000012993	310.4503397	-1.20529678	0.313829	-3.84061	0.0001227	0.000538666	down-regulated	protein_coding
SIT1	ENSMMUG00000016319	4.504086611	5.175640366	1.347617	3.840586	0.0001227	0.000538666	up-regulated	protein_coding
NACC2	ENSMMUG00000000276	65.71313684	1.883207119	0.490464	3.839645	0.0001232	0.000540569	up-regulated	protein_coding
EFTUD1	ENSMMUG00000023353	106.830513	-2.096680666	0.546161	-3.83894	0.0001236	0.000541946	down-regulated	protein_coding
CARD10	ENSMMUG00000003007	81.74847694	-2.877460967	0.74961	-3.83861	0.0001237	0.000542516	down-regulated	protein_coding

URM1	ENSMMUG00000018612	76.88009154	-2.228102604	0.580608	-3.83754	0.0001243	0.000544724	down-regulated	protein_coding
RANBP2	ENSMMUG00000021019	203.0776579	-1.571439201	0.409567	-3.83683	0.0001246	0.000546117	down-regulated	protein_coding
ARL2	ENSMMUG00000012320	125.526092	-2.391659891	0.623431	-3.83629	0.0001249	0.000547158	down-regulated	protein_coding
CHST4	ENSMMUG00000004391	9.504185438	5.405022096	1.408967	3.836159	0.000125	0.000547277	up-regulated	protein_coding
MXD4	ENSMMUG00000001160	97.26184356	-2.468023756	0.643434	-3.8357	0.0001252	0.000548119	down-regulated	protein_coding
ZBTB24	ENSMMUG00000003678	42.79774371	2.387302734	0.622508	3.834978	0.0001256	0.000549573	up-regulated	protein_coding
ARF5	ENSMMUG00000015423	199.7471541	-3.342672834	0.871788	-3.83427	0.0001259	0.000550986	down-regulated	protein_coding
MBD6	ENSMMUG00000011289	142.3859642	1.192512525	0.31106	3.833711	0.0001262	0.00055207	up-regulated	protein_coding
BAIAP2L2	ENSMMUG00000002699	192.5001046	-2.34130579	0.610927	-3.83238	0.0001269	0.000554713	down-regulated	protein_coding
NCOA6	ENSMMUG00000010715	214.4449754	1.399446256	0.365171	3.832309	0.0001269	0.000554713	up-regulated	protein_coding
OIP5	ENSMMUG00000012679	26.05739912	3.263020488	0.851433	3.832385	0.0001269	0.000554713	up-regulated	protein_coding
TBX10	ENSMMUG00000018529	19.91660385	2.27757456	0.594334	3.832146	0.000127	0.00055491	up-regulated	protein_coding
NCBP2-AS2	ENSMMUG00000007826	27.06270525	-5.12584876	1.33822	-3.83035	0.000128	0.000558808	down-regulated	protein_coding
FNTB	ENSMMUG00000004340	74.42698556	1.380549476	0.360472	3.829833	0.0001282	0.000559805	up-regulated	protein_coding
AMFR	ENSMMUG00000019902	159.0490148	-1.590144321	0.415251	-3.82936	0.0001285	0.000560534	down-regulated	protein_coding
NICA3	ENSMMUG00000023736	8.55893593	3.58779749	0.936912	3.829384	0.0001285	0.000560534	up-regulated	protein_coding
SRXN1	ENSMMUG00000017353	101.4957437	-2.241698445	0.585554	-3.82834	0.000129	0.0005624	down-regulated	protein_coding
ARPC5L	ENSMMUG00000020628	55.28873115	-3.721883806	0.972199	-3.82832	0.000129	0.0005624	down-regulated	protein_coding
TRAF2	ENSMMUG00000011527	78.13182973	-2.389107501	0.624125	-3.82793	0.0001292	0.000563107	down-regulated	protein_coding
INTS8	ENSMMUG00000005356	60.6759995	-2.669826838	0.697881	-3.82562	0.0001304	0.00056824	down-regulated	protein_coding
CCDC91	ENSMMUG00000011802	22.56936244	-4.865833187	1.271985	-3.82539	0.0001306	0.000568606	down-regulated	protein_coding
ZNF672	ENSMMUG00000000633	63.1429358	1.705015375	0.445791	3.824693	0.0001309	0.000570032	up-regulated	protein_coding
TWF1	ENSMMUG00000006154	71.69852551	-2.539270621	0.66406	-3.82385	0.0001314	0.000571621	down-regulated	protein_coding
CCL23	ENSMMUG00000002467	233.9288677	-3.035945932	0.794057	-3.82334	0.0001317	0.000572649	down-regulated	protein_coding
FAM212A	ENSMMUG00000020979	7.775057412	4.075310948	1.066433	3.821442	0.0001327	0.000576889	up-regulated	protein_coding
SNRPB2	ENSMMUG00000014493	51.48770312	-3.591931289	0.940114	-3.82074	0.0001331	0.000578353	down-regulated	protein_coding
NSA2	ENSMMUG00000011196	128.9657143	-2.281119028	0.597335	-3.81883	0.0001341	0.000582539	down-regulated	protein_coding
ABHD15	ENSMMUG00000019670	45.15231876	2.012936953	0.527111	3.818811	0.0001341	0.000582539	up-regulated	protein_coding
MX1	ENSMMUG00000015329	44.43645756	3.400360726	0.890452	3.818693	0.0001342	0.00058264	up-regulated	protein_coding
FAM173A	ENSMMUG00000011521	30.90339007	-5.317665071	1.392637	-3.81841	0.0001343	0.000583119	down-regulated	protein_coding
PLEKHA1	ENSMMUG00000013040	192.586523	-2.127134433	0.557168	-3.81776	0.0001347	0.000584479	down-regulated	protein_coding
AK7	ENSMMUG00000003397	5.056710464	5.039219197	1.320952	3.81484	0.0001363	0.000590898	up-regulated	protein_coding
ARFRP1	ENSMMUG00000000169	53.10743144	-4.251324706	1.114803	-3.81352	0.000137	0.000593694	down-regulated	protein_coding
LINGO3	ENSMMUG00000017951	8.418242852	5.857480621	1.535963	3.813555	0.000137	0.000593694	up-regulated	protein_coding
NAT1	ENSMMUG00000005920	24.40773165	-4.977372007	1.305385	-3.81295	0.0001373	0.000594885	down-regulated	protein_coding
VMA21	ENSMMUG00000009034	22.94651398	-4.888492966	1.282649	-3.81125	0.0001383	0.000598637	down-regulated	protein_coding
TMEM9B	ENSMMUG00000001998	51.71449408	-4.160267545	1.092293	-3.80875	0.0001397	0.000604353	down-regulated	protein_coding
LMF2	ENSMMUG00000030970	133.938334	-2.66298088	0.699216	-3.80853	0.0001398	0.000604708	down-regulated	protein_coding
SIN3B	ENSMMUG00000018504	87.60341109	1.343133073	0.352678	3.808388	0.0001399	0.000604859	up-regulated	protein_coding
PRTG	ENSMMUG00000003653	8.281683948	7.530814491	1.977856	3.807565	0.0001403	0.000606521	up-regulated	protein_coding
NCBP1	ENSMMUG00000012281	67.62952243	-2.531903912	0.665048	-3.8071	0.0001406	0.000607456	down-regulated	protein_coding
IPO7	ENSMMUG00000023636	203.9725449	-1.837479997	0.482883	-3.80523	0.0001417	0.000611883	down-regulated	protein_coding
FSD2	ENSMMUG00000019138	4.315458425	4.717792465	1.240421	3.803379	0.0001427	0.00061612	up-regulated	protein_coding
JAGN1	ENSMMUG00000018280	42.83988388	-4.799048954	1.261874	-3.80311	0.0001429	0.000616571	down-regulated	protein_coding
DNAJC1	ENSMMUG00000023649	39.66258851	-4.683696184	1.231863	-3.80212	0.0001435	0.000618848	down-regulated	protein_coding
SEH1L	ENSMMUG00000006800	51.79072168	-3.161099211	0.831787	-3.80037	0.0001445	0.000623049	down-regulated	protein_coding
ANKS3	ENSMMUG00000011001	125.7716399	2.583736947	0.680002	3.799602	0.0001449	0.0006248	up-regulated	protein_coding
FHIT	ENSMMUG00000012832	9.352606572	3.867466332	1.017889	3.799497	0.000145	0.000624874	up-regulated	protein_coding
IPO8	ENSMMUG00000001361	121.4851124	-1.375360265	0.362031	-3.79901	0.0001453	0.000625796	down-regulated	protein_coding
FBXW2	ENSMMUG00000001646	45.50958223	-4.014744781	1.056795	-3.79898	0.0001453	0.000625796	down-regulated	protein_coding
GLTSCR2	ENSMMUG00000013348	276.8132977	-2.65375423	0.698757	-3.79782	0.000146	0.000628541	down-regulated	protein_coding
CFTR	ENSMMUG00000011269	232.4500679	-2.233903417	0.588771	-3.79418	0.0001481	0.00063725	down-regulated	protein_coding
UBE2D2	ENSMMUG00000020666	46.2317324	-4.038407569	1.064329	-3.79432	0.000148	0.00063725	down-regulated	protein_coding
DDX18	ENSMMUG00000021343	68.72796791	-1.963512	0.517504	-3.7942	0.0001481	0.00063725	down-regulated	protein_coding
CLDN12	ENSMMUG00000023657	154.6205092	-2.68898752	0.709165	-3.79177	0.0001496	0.000643284	down-regulated	protein_coding
DNAJC10	ENSMMUG00000014394	68.75922444	-2.304715583	0.607932	-3.79107	0.00015	0.000644686	down-regulated	protein_coding
KLHL12	ENSMMUG00000017849	64.68960909	-2.043770981	0.53909	-3.79115	0.00015	0.000644686	down-regulated	protein_coding
SNX10	ENSMMUG00000023230	51.50117121	-3.546607128	0.935795	-3.78994	0.0001507	0.000647439	down-regulated	protein_coding
TSC2	ENSMMUG00000002808	188.4751594	-1.585702935	0.418555	-3.78851	0.0001516	0.000650771	down-regulated	protein_coding
BCL2L15	ENSMMUG00000006787	49.89259407	-3.414456781	0.901249	-3.78858	0.0001515	0.000650771	down-regulated	protein_coding
MAPK12	ENSMMUG00000014106	18.30261652	4.330564992	1.143165	3.788223	0.0001517	0.000651336	up-regulated	protein_coding
OCLN	ENSMMUG00000006216	201.5346878	-2.610441172	0.689154	-3.78789	0.0001519	0.000651809	down-regulated	protein_coding
CCT5	ENSMMUG00000020112	231.7095312	-1.809735101	0.477762	-3.78794	0.0001519	0.000651809	down-regulated	protein_coding
AP1S1	ENSMMUG00000010162	75.09287188	-2.889423649	0.763295	-3.78546	0.0001534	0.000657621	down-regulated	protein_coding
MCM6	ENSMMUG00000017486	59.18668802	-4.419237132	1.167413	-3.7855	0.0001534	0.000657621	down-regulated	protein_coding
MRPL22	ENSMMUG00000021229	27.78258913	-5.163686852	1.364123	-3.78535	0.0001535	0.000657707	down-regulated	protein_coding
CMTM6	ENSMMUG00000001443	135.7468569	-2.227673995	0.588707	-3.78401	0.0001543	0.000661055	down-regulated	protein_coding
COX19	ENSMMUG00000007202	23.09708748	-4.897054588	1.294185	-3.78389	0.0001544	0.000661182	down-regulated	protein_coding
PITPNM1	ENSMMUG00000011821	233.3475609	-1.499973409	0.396526	-3.78278	0.0001551	0.000663729	down-regulated	protein_coding
GINM1	ENSMMUG00000015161	53.68627351	-2.878666298	0.761023	-3.78263	0.0001552	0.00066393	down-regulated	protein_coding
C1H1ORF210	ENSMMUG00000002647	39.12230936	-4.64063486	1.226919	-3.78235	0.0001554	0.000664286	down-regulated	protein_coding
SFXN4	ENSMMUG00000008291	41.69925149	-4.756133119	1.257571	-3.782	0.0001556	0.000665021	down-regulated	protein_coding
ZNF335	ENSMMUG00000013702	179.7701249	1.653195323	0.437132	3.781911	0.0001556	0.000665055	up-regulated	protein_coding
ABHD13	ENSMMUG00000008039	20.63231926	-4.73690483	1.252967	-3.78055	0.0001565	0.000668294	down-regulated	protein_coding
HPGD	ENSMMUG00000020408	171.5128468	-4.24027764	1.121726	-3.78014	0.0001567	0.000669201	down-regulated	protein_coding
PTPN14	ENSMMUG00000015632	18.87877778	2.441854569	0.646144	3.779115	0.0001574	0.000671753	up-regulated	protein_coding
SMARCA5	ENSMMUG00000000829	205.4695496	-1.36934916	0.362359	-3.77898	0.0001575	0.00067184	down-regulated	protein_coding
CACNB2	ENSMMUG00000007445	38.08380173	3.939779087	1.042564	3.778933	0.0001575	0.00067184	up-regulated	protein_coding
USP48	ENSMMUG00000017344	59.24179294	-2.262796426	0.599007	-3.77758	0.0001584	0.000675294	down-regulated	protein_coding
ST6GALNAC2	ENSMMUG00000022273	200.2352311	1.702291798	0.4507	3.776996	0.0001587	0.000676677	up-regulated	protein_coding
ASNSD1	ENSMMUG00000014310	45.51814743	-3.405642269	0.901709	-3.77688	0.0001588	0.000676798	down-regulated	protein_coding
ZCWPW1	ENSMMUG00000012720	10.02079204	6.011319052	1.591772	3.776494	0.0001591	0.000677632	up-regulated	protein_coding
ENY2	ENSMMUG00000006104	65.61120824	-2.935377392	0.777295	-3.7764	0.0001591	0.000677683	down-regulated	protein_coding
MYOT	ENSMMUG00000016356	21.92516895	5.226661102	1.384668	3.774667	0.0001602	0.000682207	up-regulated	protein_coding
NCLN	ENSMMUG00000017036	97.17514196	-1.901608395	0.50383	-3.7743	0.0001605	0.000682995	down-regulated	protein_coding
RSPH1	ENSMMUG00000018084	9.632515076	3.811377325	1.011009	3.769874	0.0001633	0.000694814	up-regulated	protein_coding
LRP11	ENSMMUG00000009828	93.40120595	-1.713326707	0.454526	-3.76948	0.0001636	0.0006957	down-regulated	protein_coding
ZNF664	ENSMMUG00000009213	148.815303	-2.45764707	0.652006	-3.76936	0.0001637	0.00069582	down-regulated	protein_coding
PIGC	ENSMMUG00000007279	21.49314153	-4.7937661	1.271836	-3.76917	0.0001638	0.000696147	down-regulated	protein_coding
TALDO1	ENSMMUG00000007510	392.5966035	-1.4995079	0.397898	-3.76858	0.0001642	0.000697599	down-regulated	protein_coding
mml-mir-631	ENSMMUG00000027217	21.37843842	2.72230028	0.722419	3.768311	0.0001644	0.000698131	up-regulated	miRNA
INSIG1	ENSMMUG00000030017	649.1464516	-1.717481069	0.455781	-3.76822	0.0001644	0.000698177	down-regulated	protein_coding
PDE1B	ENSMMUG00000000111	20.90077649	5.75676222	1.528246	3.766908	0.0001653	0.000701643	up-regulated	protein_coding
ARFGAP1	ENSMMUG00000015911	50.90529238	-2.501205612	0.664136	-3.7661	0.0001658	0.000703693	down-regulated	protein_coding
PTPRS	ENSMMUG00000011962	8.466967506	4.742716565	1.259375	3.765929	0.0001659	0.000703764	up-regulated	protein_coding
EFNB1	ENSMMUG00000020965	253.6776138	-1.367218307	0.363044	-3.76598	0.0001659	0.000703764	down-regulated	protein_coding
MKRN2	ENSMMUG00000009536	87.91284127	1.448787832	0.384742	3.765604	0.0001661	0.000704469	up-regulated	protein_coding
MCPH1	ENSMMUG00000010137	26.58995106	2.108155862	0.560203	3.763201	0.0001678	0.000710661	up-regulated	protein_coding
MROH6	ENSMMUG00000001639	52.44759159	2.368722136	0.62946	3.763103	0.0001678	0.000710701	up-regulated	protein_coding
FRMD4A	ENSMMUG00000017164	70.2616761	1.422695437	0.378081	3.762934	0.0001679	0.000710971	up-regulated	protein_coding

RPS6KA3	ENSMMUG00000002134	76.32634804	-2.513664195	0.66812	-3.7623	0.0001684	0.000712571	down-regulated	protein_coding
RPL23	ENSMMUG00000004502	693.680504	-2.286605475	0.607842	-3.76184	0.0001687	0.00071345	down-regulated	protein_coding
KIAA0146	ENSMMUG00000013149	77.87798083	1.826025675	0.485444	3.761557	0.0001689	0.000714043	up-regulated	protein_coding
MRPL18	ENSMMUG00000017037	45.401115	-4.883977479	1.298799	-3.76038	0.0001697	0.000717199	down-regulated	protein_coding
EMC7	ENSMMUG00000000010	40.47267561	-4.715031501	1.254392	-3.75882	0.0001707	0.000721473	down-regulated	protein_coding
SULT2A1	ENSMMUG00000000047	67.74655477	-5.470092728	1.455472	-3.7583	0.0001711	0.000722549	down-regulated	protein_coding
DGAT2	ENSMMUG00000000185	136.0350867	-3.150169358	0.838368	-3.7575	0.0001716	0.000724629	down-regulated	protein_coding
CHUK	ENSMMUG00000018476	49.76643705	-3.567146672	0.94967	-3.7562	0.0001725	0.000728201	down-regulated	protein_coding
PLRG1	ENSMMUG00000021301	48.25130548	-3.069068768	0.817363	-3.75484	0.0001735	0.000731936	down-regulated	protein_coding
KLHL35	ENSMMUG00000009021	7.11629586	5.554209507	1.479312	3.754591	0.0001736	0.000732444	up-regulated	protein_coding
ECH1	ENSMMUG00000014927	189.7146402	-1.649279502	0.439365	-3.75378	0.0001742	0.00073437	down-regulated	protein_coding
KIR3DL2	ENSMMUG00000001519	9.45916737	6.152728059	1.639205	3.753482	0.0001744	0.000735035	up-regulated	protein_coding
FAM113B	ENSMMUG00000002203	11.30457235	4.458951755	1.188121	3.752943	0.0001748	0.000736181	up-regulated	protein_coding
AIFM2	ENSMMUG00000006392	133.920847	-1.921695844	0.512043	-3.753	0.0001747	0.000736181	down-regulated	protein_coding
CHN2	ENSMMUG00000008329	79.28647303	2.543472082	0.677818	3.752442	0.0001751	0.000737432	up-regulated	protein_coding
RHBDF1	ENSMMUG00000000588	188.7324902	1.841732486	0.490979	3.751142	0.000176	0.000741048	up-regulated	protein_coding
RAB11FIP4	ENSMMUG00000008127	48.71409975	-2.693154088	0.718202	-3.74986	0.0001769	0.00074464	down-regulated	protein_coding
MRPS18C	ENSMMUG00000015353	101.3424483	1.573367086	0.419613	3.749568	0.0001771	0.00074527	up-regulated	protein_coding
ING1	ENSMMUG00000022818	67.65667486	1.852645726	0.494474	3.746697	0.0001792	0.000753625	up-regulated	protein_coding
CCL3	ENSMMUG00000030216	7.050485638	4.391158301	1.172493	3.745146	0.0001803	0.000758069	up-regulated	protein_coding
ABCC1	ENSMMUG00000005914	68.51515785	1.752592396	0.468042	3.744519	0.0001807	0.00075974	up-regulated	protein_coding
CRLS1	ENSMMUG00000013744	110.1214401	2.012318298	0.537458	3.744143	0.000181	0.000760654	up-regulated	protein_coding
GCLC	ENSMMUG00000008684	210.8881229	-1.157218758	0.309167	-3.74303	0.0001818	0.000763817	down-regulated	protein_coding
SH3YL1	ENSMMUG00000017096	61.15944351	-2.844473988	0.760151	-3.74198	0.0001826	0.000766767	down-regulated	protein_coding
SPP1	ENSMMUG00000008793	52.8245929	2.908051674	0.777331	3.741074	0.0001832	0.000769317	up-regulated	protein_coding
SLC2A12	ENSMMUG00000022921	15.53042339	6.105687084	1.63245	3.740198	0.0001839	0.000771776	up-regulated	protein_coding
POGK	ENSMMUG00000017789	111.0730012	1.63132009	0.43637	3.738389	0.0001852	0.000777117	up-regulated	protein_coding
LEPR	ENSMMUG00000003265	13.15543305	5.234696082	1.400298	3.738272	0.0001853	0.000777248	up-regulated	protein_coding
NDUFS7	ENSMMUG00000003045	152.4700807	-2.392273966	0.639975	-3.73807	0.0001854	0.000777636	down-regulated	protein_coding
FAM100B	ENSMMUG00000003537	59.1400689	-3.284106779	0.878882	-3.73669	0.0001865	0.000781699	down-regulated	protein_coding
NAB2	ENSMMUG00000017525	97.17790638	1.447817802	0.387479	3.736502	0.0001866	0.000782043	up-regulated	protein_coding
WIPI2	ENSMMUG00000007045	92.4504303	-2.035101194	0.544892	-3.73487	0.0001878	0.000786642	down-regulated	protein_coding
RABAC1	ENSMMUG00000017426	50.20472939	-4.167486523	1.115852	-3.7348	0.0001879	0.000786642	down-regulated	protein_coding
TSR2	ENSMMUG00000012612	42.30799711	-4.780181372	1.280003	-3.73451	0.0001881	0.000787332	down-regulated	protein_coding
USP15	ENSMMUG00000000955	62.93275433	-2.100483087	0.562475	-3.73436	0.0001882	0.000787572	down-regulated	protein_coding
CHMP7	ENSMMUG00000016653	58.7979778	-2.187441828	0.585883	-3.73358	0.0001888	0.000789764	down-regulated	protein_coding
ZDHHC14	ENSMMUG00000015872	16.79782667	2.638844646	0.706855	3.733217	0.000189	0.000790678	up-regulated	protein_coding
APEX2	ENSMMUG00000008428	25.4097884	-5.034988487	1.348839	-3.73283	0.0001893	0.000791421	down-regulated	protein_coding
C19ORF62	ENSMMUG00000013923	84.08479502	-2.464611806	0.660252	-3.73284	0.0001893	0.000791421	down-regulated	protein_coding
NDUFB7	ENSMMUG00000021612	134.0035715	-3.003028675	0.80459	-3.73237	0.0001897	0.000792641	down-regulated	protein_coding
GALK1	ENSMMUG00000021765	122.3032027	-1.99351171	0.534169	-3.73199	0.00019	0.000793603	down-regulated	protein_coding
GPR137B	ENSMMUG00000014263	69.02891468	1.725884619	0.462637	3.730534	0.0001911	0.000797966	up-regulated	protein_coding
LAMA4	ENSMMUG00000013782	7.998193717	4.296674967	1.15209	3.729462	0.0001919	0.000801132	up-regulated	protein_coding
COX4NB	ENSMMUG00000006762	48.47459238	-3.507769613	0.940662	-3.72904	0.0001922	0.00080223	down-regulated	protein_coding
SNORD63	ENSMMUG00000035047	12.96461787	5.098601033	1.367574	3.728208	0.0001928	0.000804653	up-regulated	snoRNA
TMOD3	ENSMMUG00000002345	68.33999492	-2.796628351	0.750549	-3.72611	0.0001945	0.000811141	down-regulated	protein_coding
SUMF1	ENSMMUG00000004734	54.02991129	-2.57905197	0.692172	-3.72603	0.0001945	0.000811166	down-regulated	protein_coding
MAP4K5	ENSMMUG00000020550	57.69801479	-2.427443148	0.651533	-3.72574	0.0001947	0.000811843	down-regulated	protein_coding
APLP2	ENSMMUG00000018029	1821.892182	-1.177342864	0.316015	-3.72559	0.0001949	0.000812085	down-regulated	protein_coding
MRPL34	ENSMMUG00000009657	44.39268654	-4.848379974	1.30167	-3.72474	0.0001955	0.000814527	down-regulated	protein_coding
SV2A	ENSMMUG00000007257	5.457406735	5.889019621	1.581122	3.724584	0.0001956	0.00081462	up-regulated	protein_coding
COX4I1	ENSMMUG00000006763	720.0588723	-1.632893308	0.43844	-3.72433	0.0001958	0.000815213	down-regulated	protein_coding
SUPT20H	ENSMMUG00000003590	64.49520939	-2.224032624	0.597206	-3.72406	0.000196	0.000815819	down-regulated	protein_coding
KCMF1	ENSMMUG00000001414	123.5182834	-1.365665848	0.366869	-3.72249	0.0001973	0.000820428	down-regulated	protein_coding
CUL1	ENSMMUG00000017541	101.7598183	-1.959681951	0.526507	-3.72205	0.0001976	0.000821643	down-regulated	protein_coding
UBE2D1	ENSMMUG00000019825	20.18120484	-4.705268507	1.264722	-3.7204	0.0001989	0.000826784	down-regulated	protein_coding
HBXIP	ENSMMUG00000015023	62.27410328	-2.46954198	0.664003	-3.71917	0.0001999	0.000830561	down-regulated	protein_coding
RCAN1	ENSMMUG00000004600	231.5135596	-1.993783845	0.536179	-3.7185	0.0002004	0.000832279	down-regulated	protein_coding
C2CD4D	ENSMMUG00000014358	11.32206037	4.280705221	1.151177	3.718546	0.0002004	0.000832279	up-regulated	protein_coding
NCF1	ENSMMUG00000007586	13.14892083	3.922985973	1.055024	3.718387	0.0002005	0.000832412	up-regulated	protein_coding
LAMB2	ENSMMUG00000002952	179.7741408	-1.712897423	0.460949	-3.71602	0.0002024	0.000839741	down-regulated	protein_coding
AMACR	ENSMMUG00000013467	47.27191223	-3.984369522	1.072606	-3.71466	0.0002035	0.000843778	down-regulated	protein_coding
MAP3K5	ENSMMUG00000016376	53.78452728	-3.252708428	0.875679	-3.7145	0.0002036	0.000844071	down-regulated	protein_coding
SCO2	ENSMMUG00000001764	168.4621729	1.808438916	0.4869	3.714187	0.0002039	0.00084487	up-regulated	protein_coding
OXNAD1	ENSMMUG00000022693	21.71529952	-4.808909849	1.295084	-3.7132	0.0002047	0.000847919	down-regulated	protein_coding
TMEM179	ENSMMUG00000029177	8.245633055	8.240312084	2.219514	3.712665	0.0002051	0.000849471	up-regulated	protein_coding
IGLV7-46	ENSMMUG00000007435	8.242058763	8.240307213	2.219696	3.712358	0.0002053	0.000850255	up-regulated	protein_coding
CAT	ENSMMUG00000002429	282.5235486	-1.311746874	0.353394	-3.71185	0.0002058	0.000851714	down-regulated	protein_coding
MRPS18B	ENSMMUG00000006525	161.5888245	1.559388277	0.420153	3.711478	0.0002061	0.000852718	up-regulated	protein_coding
DDX42	ENSMMUG00000016762	107.9083756	-1.794783066	0.483799	-3.70977	0.0002074	0.00085823	down-regulated	protein_coding
POLR2L	ENSMMUG00000020622	91.52035476	-2.969089274	0.800488	-3.7091	0.000208	0.000860268	down-regulated	protein_coding
CHRNA7	ENSMMUG00000022577	10.15183666	6.944124274	1.872307	3.70886	0.0002082	0.000860826	up-regulated	protein_coding
LOX	ENSMMUG00000021007	6.19589835	6.182587265	1.667987	3.706615	0.00021	0.000867981	up-regulated	protein_coding
SLU7	ENSMMUG00000023075	57.79060644	-2.603270932	0.702496	-3.70575	0.0002108	0.000870711	down-regulated	protein_coding
THSD4	ENSMMUG00000006118	16.79930364	2.964686169	0.800104	3.705374	0.0002111	0.000871489	up-regulated	protein_coding
7SK	ENSMMUG00000034510	8.913035164	4.69618754	1.2674	3.705371	0.0002111	0.000871489	up-regulated	misc_RNA
CDC26	ENSMMUG00000005843	88.57352548	1.407799092	0.379981	3.704917	0.0002115	0.000872352	up-regulated	protein_coding
DPY30	ENSMMUG00000014481	40.63246637	-4.717931731	1.273431	-3.7049	0.0002115	0.000872352	down-regulated	protein_coding
TNFRSF14	ENSMMUG00000016329	131.1526957	-1.950652775	0.526506	-3.7049	0.0002115	0.000872352	down-regulated	protein_coding
MAGEE2	ENSMMUG00000023635	21.00902349	3.281269946	0.886068	3.70318	0.0002129	0.000878028	up-regulated	protein_coding
ITGB6	ENSMMUG00000002678	41.37553922	-4.724628165	1.275911	-3.70295	0.0002131	0.000878585	down-regulated	protein_coding
STARD10	ENSMMUG00000015054	599.7183873	-1.862047831	0.50291	-3.70255	0.0002134	0.000879714	down-regulated	protein_coding
MPHOSPH10	ENSMMUG00000008397	116.8757917	1.212700536	0.327557	3.702253	0.0002137	0.000880474	up-regulated	protein_coding
RNF141	ENSMMUG00000015786	55.12085024	-2.842946349	0.767913	-3.70217	0.0002138	0.000880492	down-regulated	protein_coding
PLS1	ENSMMUG00000012403	753.8609629	-2.47591803	0.668977	-3.70105	0.0002147	0.000884132	down-regulated	protein_coding
UACA	ENSMMUG00000007570	101.1977755	-1.611878093	0.435546	-3.70082	0.0002149	0.000884684	down-regulated	protein_coding
NPRL3	ENSMMUG00000000590	76.85014516	1.980501445	0.535358	3.699396	0.0002161	0.00088915	up-regulated	protein_coding
MBD2	ENSMMUG00000003136	119.7570109	-1.610500496	0.435373	-3.69912	0.0002163	0.000889844	down-regulated	protein_coding
BTD	ENSMMUG00000020762	85.417612	-2.901057546	0.784342	-3.69872	0.0002167	0.000891015	down-regulated	protein_coding
C3ORF26	ENSMMUG00000002939	15.65815662	3.12386131	0.844662	3.698359	0.000217	0.000892012	up-regulated	protein_coding
SPATA24	ENSMMUG00000023071	9.828455112	3.297448429	0.891804	3.697502	0.0002177	0.000894765	up-regulated	protein_coding
SRCIN1	ENSMMUG00000005041	16.76019873	4.463953777	1.20771	3.696213	0.0002188	0.000899058	up-regulated	protein_coding
ZFAND1	ENSMMUG00000002626	46.11866663	-3.337176772	0.902891	-3.6961	0.0002189	0.000899191	down-regulated	protein_coding
GNL3	ENSMMUG00000012511	100.0230021	-2.694367588	0.729114	-3.6954	0.0002195	0.000901151	down-regulated	protein_coding
IL18BP	ENSMMUG00000007340	101.0324093	1.79469841	0.485952	3.693163	0.0002215	0.00090886	up-regulated	protein_coding
COMMD7	ENSMMUG00000022721	18.91617291	-4.610355916	1.248421	-3.69295	0.0002217	0.000909363	down-regulated	protein_coding
URI1	ENSMMUG00000018441	52.87897217	-2.562838263	0.69402	-3.69274	0.0002218	0.000909837	down-regulated	protein_coding
LMOD3	ENSMMUG00000008628	5.484326346	4.9819523	1.349504	3.691692	0.0002228	0.00091307	up-regulated	protein_coding
NOS3	ENSMMUG00000020437	7.362266224	6.14621302	1.664857	3.691737	0.0002227	0.00091307	up-regulated	protein_coding
GPBAR1	ENSMMUG00000022811	34.46177375	2.242592531	0.607482	3.69162	0.0002228	0.00091307	up-regulated	protein_coding

CDKN2AIP	ENSMMUG00000002004	36.51853352	-4.565110401	1.237432	-3.68918	0.000225	0.000921389	down-regulated	protein_coding
EIF4E2	ENSMMUG00000022151	69.78057024	-2.828450982	0.766691	-3.68917	0.000225	0.000921389	down-regulated	protein_coding
VRK3	ENSMMUG00000010837	92.77759648	-2.258173455	0.612247	-3.68833	0.0002257	0.000924134	down-regulated	protein_coding
BDH2	ENSMMUG00000019085	64.23310258	-3.466662023	0.940687	-3.68524	0.0002285	0.000935154	down-regulated	protein_coding
RNF7	ENSMMUG00000006648	68.47752552	-2.176013947	0.590527	-3.68487	0.0002288	0.000936268	down-regulated	protein_coding
ZNF462	ENSMMUG00000017151	83.18157983	1.418895098	0.385111	3.684381	0.0002293	0.000937782	up-regulated	protein_coding
MED23	ENSMMUG00000017544	55.48139392	-2.673713739	0.725715	-3.68425	0.0002294	0.000937997	down-regulated	protein_coding
EIF4E	ENSMMUG00000011066	42.52008004	-3.301082507	0.896206	-3.6834	0.0002301	0.000940326	down-regulated	protein_coding
RAB22A	ENSMMUG00000000125	40.34161187	-4.709538289	1.278767	-3.68287	0.0002306	0.000941732	down-regulated	protein_coding
ZNF706	ENSMMUG00000006943	203.0813831	-1.284056224	0.348657	-3.68287	0.0002306	0.000941732	down-regulated	protein_coding
SHFM1	ENSMMUG00000004127	56.82052222	-3.761808265	1.021604	-3.68226	0.0002312	0.000943721	down-regulated	protein_coding
C9H10ORF107	ENSMMUG00000013152	9.095806931	6.829684249	1.854824	3.68212	0.0002313	0.000943958	up-regulated	protein_coding
AGPAT2	ENSMMUG00000020990	370.5207342	-2.492063992	0.676942	-3.68136	0.000232	0.00094652	down-regulated	protein_coding
MAP2K3	ENSMMUG00000010904	215.8513051	-1.43360746	0.389517	-3.68048	0.0002328	0.000949236	down-regulated	protein_coding
HM13	ENSMMUG00000005119	106.2907621	-2.531071983	0.687747	-3.68024	0.000233	0.000949849	down-regulated	protein_coding
SLC35E2B	ENSMMUG00000005578	155.6557846	1.853416999	0.503663	3.679875	0.0002333	0.000950934	up-regulated	protein_coding
EBP	ENSMMUG00000018336	549.2491564	-1.876802672	0.510041	-3.67971	0.0002335	0.000951276	down-regulated	protein_coding
NT5C3	ENSMMUG00000011695	161.931683	-1.631009027	0.443323	-3.67905	0.0002341	0.000953186	down-regulated	protein_coding
RBBP7	ENSMMUG00000016238	100.0044161	-2.280070792	0.619768	-3.67891	0.0002342	0.000953434	down-regulated	protein_coding
GTF2B	ENSMMUG00000002794	128.6678471	1.4964312	0.406773	3.678791	0.0002343	0.000953611	up-regulated	protein_coding
METTL21A	ENSMMUG00000022602	42.76866574	-3.323990693	0.903603	-3.67859	0.0002345	0.000954071	down-regulated	protein_coding
AP1S2	ENSMMUG00000014055	27.22205566	1.986080071	0.540281	3.676012	0.0002369	0.000963223	up-regulated	protein_coding
BAT2L2	ENSMMUG00000013639	654.2598105	1.205022431	0.327865	3.675365	0.0002375	0.000965389	up-regulated	protein_coding
VPS24	ENSMMUG00000013134	44.51421136	-3.389249168	0.922194	-3.6752	0.0002377	0.000965726	down-regulated	protein_coding
MOGS	ENSMMUG00000002726	191.0673704	-1.490715353	0.405679	-3.67462	0.0002382	0.000967654	down-regulated	protein_coding
SPTLC2	ENSMMUG00000013556	47.87677429	-4.108154544	1.118232	-3.67379	0.000239	0.000970236	down-regulated	protein_coding
TCF7	ENSMMUG00000022788	168.7353869	1.656387551	0.450937	3.673217	0.0002395	0.00097215	up-regulated	protein_coding
EPN3	ENSMMUG00000017249	26.75271261	3.184227337	0.866916	3.673053	0.0002397	0.000972492	up-regulated	protein_coding
SELK	ENSMMUG00000019272	37.65773931	-4.581561168	1.247602	-3.67229	0.0002404	0.000975105	down-regulated	protein_coding
RABL6	ENSMMUG00000005042	459.9266775	-1.491145961	0.406078	-3.67207	0.0002406	0.000975513	down-regulated	protein_coding
MRFAP1L1	ENSMMUG00000021886	20.01808247	-4.691005506	1.277728	-3.67136	0.0002413	0.000977822	down-regulated	protein_coding
SEC63	ENSMMUG00000016598	111.9855339	-1.501847492	0.409179	-3.67039	0.0002422	0.00098127	down-regulated	protein_coding
CCDC47	ENSMMUG00000016761	248.5332152	-1.120252753	0.30537	-3.66851	0.000244	0.00098794	down-regulated	protein_coding
C14H11orf31	ENSMMUG00000002566	57.83536838	-3.362263009	0.916936	-3.66685	0.0002456	0.000994118	down-regulated	protein_coding
TRUB2	ENSMMUG00000014146	116.6266653	1.628026392	0.444004	3.666693	0.0002457	0.000994428	up-regulated	protein_coding
MRI1	ENSMMUG00000018068	21.0017503	-4.760626937	1.298757	-3.66553	0.0002468	0.000998685	down-regulated	protein_coding
KBTBD13	ENSMMUG00000004028	9.272160414	4.446190975	1.213022	3.665382	0.000247	0.000998962	up-regulated	protein_coding
STT3B	ENSMMUG00000008608	183.6889723	-1.874322949	0.511695	-3.66297	0.0002493	0.001007567	down-regulated	protein_coding
TMEM165	ENSMMUG00000001918	81.40464033	-2.694009235	0.735844	-3.66111	0.0002511	0.001014304	down-regulated	protein_coding
PPP1R37	ENSMMUG00000022930	78.90412767	-2.601737785	0.710694	-3.66084	0.0002514	0.001015107	down-regulated	protein_coding
PIH1D1	ENSMMUG00000022116	184.8846258	2.015677745	0.550751	3.659872	0.0002523	0.001018654	up-regulated	protein_coding
SRGAP1	ENSMMUG00000001952	28.30680482	1.879607492	0.513642	3.659373	0.0002528	0.001020345	up-regulated	protein_coding
FBLIM1	ENSMMUG00000032243	121.7142101	-1.877479727	0.513081	-3.65923	0.000253	0.001020635	down-regulated	protein_coding
TMEM19	ENSMMUG00000020948	37.24436463	-4.591039207	1.255334	-3.65723	0.000255	0.001028343	down-regulated	protein_coding
KCTD9	ENSMMUG00000010151	42.65377101	-3.838865466	1.049879	-3.65648	0.0002557	0.001031029	down-regulated	protein_coding
PFDN2	ENSMMUG00000002008	43.14048765	-4.808741262	1.31534	-3.65589	0.0002563	0.001033112	down-regulated	protein_coding
FAAH2	ENSMMUG00000016725	19.38330105	-4.646104889	1.271187	-3.65494	0.0002572	0.001036386	down-regulated	protein_coding
INSR	ENSMMUG00000028907	110.6242477	2.007217593	0.54917	3.654999	0.0002572	0.001036386	up-regulated	protein_coding
SYN2	ENSMMUG00000001120	20.41851069	3.919068674	1.072511	3.654105	0.0002581	0.001039451	up-regulated	protein_coding
C10ORF84	ENSMMUG00000012741	20.9916802	-4.759149303	1.303267	-3.65171	0.0002605	0.00104891	down-regulated	protein_coding
CCNC	ENSMMUG00000004061	52.20535438	-2.509265096	0.687202	-3.65142	0.0002608	0.001049768	down-regulated	protein_coding
COG3	ENSMMUG00000018409	47.01803836	-3.02620206	0.828845	-3.65111	0.0002611	0.001050762	down-regulated	protein_coding
TMEM70	ENSMMUG00000003017	38.99866229	-4.660024416	1.276374	-3.65099	0.0002612	0.001050961	down-regulated	protein_coding
CLIP4	ENSMMUG00000010259	14.85553694	3.450885946	0.945224	3.650865	0.0002614	0.001051154	up-regulated	protein_coding
FBXW8	ENSMMUG00000017475	64.60973403	1.897007149	0.519679	3.650346	0.0002619	0.001052981	up-regulated	protein_coding
METTL7B	ENSMMUG00000013225	63.52413484	-3.491341729	0.956941	-3.64844	0.0002638	0.001059622	down-regulated	protein_coding
LINC00998	ENSMMUG00000030198	62.48461385	-3.121618672	0.855724	-3.64793	0.0002644	0.001061436	down-regulated	protein_coding
TMEM230	ENSMMUG00000013258	46.23067422	-3.997383695	1.095833	-3.6478	0.0002645	0.001061647	down-regulated	protein_coding
HIST1H2BM	ENSMMUG00000029924	18.18160092	3.531140866	0.968112	3.64745	0.0002649	0.001062805	up-regulated	protein_coding
AHNAK2	ENSMMUG00000010579	25.88325328	4.339913536	1.190459	3.645579	0.0002668	0.001070263	up-regulated	protein_coding
IRF2BP1	ENSMMUG00000019680	55.03019346	-3.302990954	0.906498	-3.64368	0.0002688	0.001077575	down-regulated	protein_coding
LFNG	ENSMMUG00000016074	133.9724535	-2.863178356	0.785819	-3.64356	0.0002689	0.001077776	down-regulated	protein_coding
RABGGTB	ENSMMUG00000000873	44.29402049	-4.847665495	1.330664	-3.64304	0.0002694	0.001079642	down-regulated	protein_coding
ALL-1	ENSMMUG00000005482	150.6403979	1.76070897	0.483345	3.642755	0.0002697	0.001080545	up-regulated	protein_coding
TBC1D15	ENSMMUG00000007727	37.10568768	-4.589057134	1.260126	-3.64174	0.0002708	0.001084188	down-regulated	protein_coding
SDHAF1	ENSMMUG00000028788	23.89732795	-4.94657073	1.358297	-3.64174	0.0002708	0.001084188	down-regulated	protein_coding
PANK4	ENSMMUG00000014686	53.04343874	-2.685866841	0.737644	-3.64114	0.0002714	0.001086405	down-regulated	protein_coding
NDUFB9	ENSMMUG00000015493	98.35539746	-2.352418032	0.646271	-3.63999	0.0002727	0.001090988	down-regulated	protein_coding
TSPAN6	ENSMMUG00000008209	21.35616736	-4.785774374	1.314923	-3.63958	0.0002731	0.00109239	down-regulated	protein_coding
TIMMDC1	ENSMMUG00000012014	58.85112104	-2.236353264	0.614499	-3.63931	0.0002734	0.001093234	down-regulated	protein_coding
UROS	ENSMMUG00000008539	63.08452826	-3.137302627	0.862195	-3.63874	0.000274	0.00109537	down-regulated	protein_coding
C6orf106	ENSMMUG00000022824	330.0674671	-1.208866235	0.332273	-3.63817	0.0002746	0.00109747	down-regulated	protein_coding
OR10Z1	ENSMMUG00000000727	10.04144627	9.241053904	2.540599	3.637352	0.0002755	0.001099103	up-regulated	protein_coding
RFX3	ENSMMUG00000000802	18.76122511	2.494014753	0.685649	3.637451	0.0002753	0.001099103	up-regulated	protein_coding
PKD1L1	ENSMMUG00000007032	10.04144627	9.241053904	2.540599	3.637352	0.0002755	0.001099103	up-regulated	protein_coding
SDK1	ENSMMUG00000019724	10.04144627	9.241053904	2.540599	3.637352	0.0002755	0.001099103	up-regulated	protein_coding
TMEM38B	ENSMMUG00000002672	37.66574282	-4.608129695	1.266939	-3.63721	0.0002756	0.001099379	down-regulated	protein_coding
C4H6ORF203	ENSMMUG00000007391	20.12900229	-4.69845768	1.292239	-3.63591	0.000277	0.001104668	down-regulated	protein_coding
KIAA1683	ENSMMUG00000016354	8.485652542	3.903133982	1.073702	3.63521	0.0002778	0.001107339	up-regulated	protein_coding
MFAP5	ENSMMUG00000010747	8.055794931	5.075922857	1.396512	3.634715	0.0002783	0.001109154	up-regulated	protein_coding
TGIF1	ENSMMUG00000005086	146.4013438	-2.242714163	0.61704	-3.63463	0.0002784	0.001109198	down-regulated	protein_coding
GPR68	ENSMMUG00000022390	18.30999061	3.949602113	1.086685	3.634542	0.0002785	0.001109272	up-regulated	protein_coding
ZDHHC20	ENSMMUG00000007373	48.31702701	-4.97496252	1.369061	-3.63385	0.0002792	0.001111945	down-regulated	protein_coding
SNRPD3	ENSMMUG00000000264	95.23677466	-2.656726021	0.731171	-3.63352	0.0002796	0.001113054	down-regulated	protein_coding
TAF2	ENSMMUG00000014702	46.82301925	-2.623991836	0.722433	-3.63216	0.0002811	0.00111863	down-regulated	protein_coding
TACO1	ENSMMUG00000014556	47.88365405	-3.502028327	0.964341	-3.63152	0.0002818	0.00112102	down-regulated	protein_coding
AHCTF1	ENSMMUG00000016408	111.1870278	-1.708725119	0.470534	-3.63146	0.0002818	0.00112102	down-regulated	protein_coding
RNFT1	ENSMMUG00000009203	40.91556575	-3.854813184	1.061925	-3.63002	0.0002834	0.001126967	down-regulated	protein_coding
MAGED1	ENSMMUG00000021120	71.58323791	-2.414136778	0.665269	-3.62881	0.0002847	0.001131631	down-regulated	protein_coding
ZFP91	ENSMMUG00000011714	132.9000568	-1.460939001	0.402698	-3.62788	0.0002858	0.001135088	down-regulated	protein_coding
VPS26A	ENSMMUG00000004101	35.01050956	-4.47322731	1.233325	-3.62697	0.0002868	0.001138795	down-regulated	protein_coding
PMAIP1	ENSMMUG00000012622	55.65206877	-3.068273578	0.846002	-3.62679	0.000287	0.001139234	down-regulated	protein_coding
ZNF367	ENSMMUG00000007996	27.47632124	2.349343419	0.647859	3.626319	0.0002875	0.001141009	up-regulated	protein_coding
RNF41	ENSMMUG00000021981	19.32376486	-4.642724082	1.280632	-3.62534	0.0002886	0.001145024	down-regulated	protein_coding
PAK4	ENSMMUG00000013140	106.1857695	-1.617229308	0.44611	-3.62518	0.0002888	0.001145391	down-regulated	protein_coding
PLP2	ENSMMUG00000023022	309.1180225	-2.13187525	0.588111	-3.62495	0.000289	0.001146085	down-regulated	protein_coding
MEIS2	ENSMMUG00000006840	6.126311516	4.441587938	1.225448	3.62446	0.0002896	0.001147958	up-regulated	protein_coding
ANXA2R	ENSMMUG00000019477	32.36502789	4.00922267	1.106554	3.623161	0.000291	0.001153419	up-regulated	protein_coding
SCMH1	ENSMMUG00000017104	49.11696302	-2.701402581	0.746177	-3.62032	0.0002942	0.001165492	down-regulated	protein_coding
ABCD1	ENSMMUG00000012746	75.95866781	-1.888499411	0.521736	-3.61964	0.000295	0.001168226	down-regulated	protein_coding

ATXN10	ENSMMUG00000008955	36.46487521	-4.557782766	1.259469	-3.61881	0.000296	0.001171658	down-regulated	protein_coding
SSBP1	ENSMMUG00000022442	42.51454522	-4.763237027	1.316292	-3.61868	0.0002961	0.001171934	down-regulated	protein_coding
COL15A1	ENSMMUG00000011582	6.78152249	4.058555326	1.122024	3.617174	0.0002978	0.001178438	up-regulated	protein_coding
CHCHD3	ENSMMUG00000001671	122.192941	-1.951512024	0.539697	-3.61594	0.0002993	0.001183552	down-regulated	protein_coding
NDUFA12	ENSMMUG00000018897	46.75840468	-3.454984484	0.955508	-3.61586	0.0002994	0.001183552	down-regulated	protein_coding
TNRC6B	ENSMMUG00000021345	82.93019229	1.64095503	0.453825	3.615836	0.0002994	0.001183552	up-regulated	protein_coding
NAP1L5	ENSMMUG00000010140	8.414081878	4.693901011	1.298673	3.614383	0.0003011	0.001189874	up-regulated	protein_coding
PLEKHG3	ENSMMUG00000019170	336.3458419	-1.549805019	0.428837	-3.61397	0.0003015	0.001191436	down-regulated	protein_coding
GAPVD1	ENSMMUG00000003668	77.35654287	-2.201953129	0.60938	-3.61343	0.0003022	0.001193571	down-regulated	protein_coding
SRC	ENSMMUG00000020131	78.41966518	-1.998533102	0.55325	-3.61235	0.0003034	0.00119823	down-regulated	protein_coding
SEMA3B	ENSMMUG00000020117	439.2159489	-1.411003662	0.390621	-3.61221	0.0003036	0.001198571	down-regulated	protein_coding
SFN	ENSMMUG00000013855	118.4026724	-2.167384894	0.600065	-3.61192	0.0003039	0.001199568	down-regulated	protein_coding
NELFCD	ENSMMUG00000015390	313.5777684	1.361913174	0.377107	3.611479	0.0003045	0.001200931	up-regulated	protein_coding
DAP	ENSMMUG00000001131	194.426858	-1.070547451	0.296457	-3.61113	0.0003049	0.001201965	down-regulated	protein_coding
CEBPD	ENSMMUG00000013151	54.13390092	-3.700363055	1.024758	-3.61096	0.0003051	0.001202327	down-regulated	protein_coding
C20H16ORF80	ENSMMUG00000015891	34.8765776	-4.495548547	1.245057	-3.61072	0.0003054	0.001203123	down-regulated	protein_coding
PSMD14	ENSMMUG00000011906	109.7609133	-1.684864375	0.466657	-3.6105	0.0003056	0.00120373	down-regulated	protein_coding
EML2	ENSMMUG00000019032	41.53356933	-3.218457196	0.891751	-3.60914	0.0003072	0.001209431	down-regulated	protein_coding
ROMO1	ENSMMUG00000005130	67.27315958	-3.866106717	1.071266	-3.60891	0.0003075	0.001210174	down-regulated	protein_coding
TULP4	ENSMMUG00000019448	52.18343408	2.130794699	0.590554	3.608127	0.0003084	0.001213508	up-regulated	protein_coding
C16orf93	ENSMMUG00000020880	19.52192672	3.015093622	0.835692	3.607903	0.0003087	0.001214219	up-regulated	protein_coding
PRMT3	ENSMMUG00000010719	18.89637605	-4.609828183	1.277827	-3.60755	0.0003091	0.001215526	down-regulated	protein_coding
KRT10	ENSMMUG00000001500	84.49100039	1.7130893	0.474906	3.607219	0.0003095	0.001216746	up-regulated	protein_coding
STXBP3	ENSMMUG00000010158	57.78279856	-2.411384525	0.668509	-3.60711	0.0003096	0.001216922	down-regulated	protein_coding
CYP4B1	ENSMMUG00000019873	185.1333665	-2.907907265	0.806251	-3.6067	0.0003101	0.001218498	down-regulated	protein_coding
TMEM134	ENSMMUG00000005493	46.26018442	-4.056110204	1.124775	-3.60615	0.0003108	0.001220052	down-regulated	protein_coding
TBK1	ENSMMUG00000007658	39.43827035	-3.798723062	1.053367	-3.60627	0.0003106	0.001220052	down-regulated	protein_coding
DCTN6	ENSMMUG00000013735	42.89429479	-3.885872894	1.077562	-3.60617	0.0003107	0.001220052	down-regulated	protein_coding
5S_rRNA	ENSMMUG00000037226	8.641580115	6.605310058	1.833277	3.603007	0.0003146	0.001234238	up-regulated	rRNA
SCLY	ENSMMUG00000018483	56.4463595	-2.630734595	0.730301	-3.60226	0.0003155	0.001237452	down-regulated	protein_coding
ACSL4	ENSMMUG00000004188	75.59875582	-1.912694213	0.531097	-3.6014	0.0003165	0.001241207	down-regulated	protein_coding
PLSCR4	ENSMMUG00000007976	68.20885773	-5.47994772	1.521886	-3.60076	0.0003173	0.00124384	down-regulated	protein_coding
DDX54	ENSMMUG00000021795	118.5711089	-1.779040861	0.494171	-3.60005	0.0003182	0.001246626	down-regulated	protein_coding
SNAPC4	ENSMMUG00000003086	41.98989256	-4.744806909	1.318563	-3.59847	0.0003201	0.001253543	down-regulated	protein_coding
ATP5G1	ENSMMUG00000016241	316.3620373	-1.884784454	0.523838	-3.59803	0.0003206	0.001255327	down-regulated	protein_coding
EIF2B5	ENSMMUG00000006562	90.00728427	-2.86974793	0.797685	-3.5976	0.0003212	0.001256709	down-regulated	protein_coding
MRPL20	ENSMMUG00000006905	56.73062575	-4.278241986	1.189188	-3.59762	0.0003211	0.001256709	down-regulated	protein_coding
RAB4A	ENSMMUG00000017720	62.72619864	-2.215892775	0.616037	-3.59701	0.0003219	0.001259186	down-regulated	protein_coding
INA	ENSMMUG00000008872	26.96202586	2.260016735	0.628445	3.596204	0.0003229	0.001262753	up-regulated	protein_coding
UBR5	ENSMMUG00000020766	174.6637372	-1.536523759	0.427328	-3.59566	0.0003236	0.001265053	down-regulated	protein_coding
PPP4R2	ENSMMUG00000016959	58.77590244	-2.937168511	0.816886	-3.59557	0.0003237	0.001265135	down-regulated	protein_coding
PGAM1	ENSMMUG00000010554	143.5533109	-2.021513402	0.562471	-3.59399	0.0003257	0.001272349	down-regulated	protein_coding
CWC15	ENSMMUG00000023374	49.57760263	-3.491018459	0.971361	-3.59395	0.0003257	0.001272349	down-regulated	protein_coding
RING1	ENSMMUG00000003863	55.03895934	-2.95225153	0.821511	-3.59368	0.000326	0.001272919	down-regulated	protein_coding
ZNF786	ENSMMUG00000018549	17.81157447	3.043118182	0.846788	3.59372	0.000326	0.001272919	up-regulated	protein_coding
MSH6	ENSMMUG00000022842	139.1922263	1.157722997	0.322191	3.59328	0.0003265	0.001274545	up-regulated	protein_coding
PEX2	ENSMMUG00000002280	17.74239968	-4.518178038	1.257584	-3.59275	0.0003272	0.001276788	down-regulated	protein_coding
NTRK2	ENSMMUG00000023117	5.307234908	4.541548422	1.264112	3.592679	0.0003273	0.001276788	up-regulated	protein_coding
PBXIP1	ENSMMUG00000006576	254.5636045	-1.358707284	0.378262	-3.59198	0.0003282	0.001279888	down-regulated	protein_coding
NDUFV1	ENSMMUG00000018526	202.0037066	-1.85072354	0.515288	-3.59163	0.0003286	0.00128123	down-regulated	protein_coding
LMO4	ENSMMUG00000017383	55.37887399	-2.629575771	0.73216	-3.59153	0.0003287	0.001281357	down-regulated	protein_coding
DMRTB1	ENSMMUG00000019081	95.11566577	-4.567784227	1.27214	-3.59063	0.0003299	0.001285448	down-regulated	protein_coding
RAPGEF3	ENSMMUG00000000477	20.35668206	2.251674732	0.627206	3.59001	0.0003307	0.001288155	up-regulated	protein_coding
CPNE1	ENSMMUG00000020960	116.5350837	-1.764170639	0.491449	-3.58974	0.000331	0.00128916	down-regulated	protein_coding
MAST2	ENSMMUG00000015371	406.8057648	-1.385517687	0.386015	-3.58928	0.0003316	0.001291034	down-regulated	protein_coding
GRB7	ENSMMUG00000020775	182.0358431	-1.868505727	0.521016	-3.58627	0.0003354	0.001305685	down-regulated	protein_coding
APOBEC1	ENSMMUG00000016169	43.09472304	-3.893812032	1.085823	-3.58605	0.0003357	0.001306448	down-regulated	protein_coding
LARP1	ENSMMUG00000009411	216.3268789	1.397498436	0.389721	3.585894	0.0003359	0.001306849	up-regulated	protein_coding
RANBP6	ENSMMUG00000011603	48.50564367	-2.710111314	0.756146	-3.58411	0.0003382	0.00131544	down-regulated	protein_coding
HHLA2	ENSMMUG00000019978	66.22831201	-3.586028583	1.000615	-3.58383	0.0003386	0.001316525	down-regulated	protein_coding
NCALD	ENSMMUG00000020996	26.83085555	5.793523627	1.616675	3.583604	0.0003389	0.00131728	up-regulated	protein_coding
PHKG1	ENSMMUG00000015276	10.15060093	7.056078614	1.969347	3.582954	0.0003397	0.001320199	up-regulated	protein_coding
RPL4	ENSMMUG00000016844	1593.06919	-2.137601822	0.596698	-3.58238	0.0003405	0.001322726	down-regulated	protein_coding
PCNT	ENSMMUG00000012914	152.9753474	1.32387098	0.369567	3.582225	0.0003407	0.001323164	up-regulated	protein_coding
ISG20L2	ENSMMUG00000000037	94.11425634	1.140896615	0.318522	3.581841	0.0003412	0.001324729	up-regulated	protein_coding
ATP4A	ENSMMUG00000000845	17.57663058	2.442745643	0.681993	3.581773	0.0003413	0.001324729	up-regulated	protein_coding
RNF40	ENSMMUG00000020881	149.5746952	-1.409403183	0.393509	-3.58163	0.0003415	0.001325074	down-regulated	protein_coding
ACP6	ENSMMUG00000013561	168.4400413	1.619596524	0.452432	3.579758	0.0003439	0.001334251	up-regulated	protein_coding
DGAT1	ENSMMUG00000000825	702.7145282	-1.307408816	0.365297	-3.57903	0.0003449	0.00133761	down-regulated	protein_coding
PLEKHG7	ENSMMUG00000002243	13.83916107	7.05622643	1.971739	3.578682	0.0003453	0.001338657	up-regulated	protein_coding
LYPD8	ENSMMUG00000007995	60.95529653	-5.315566659	1.485324	-3.57873	0.0003453	0.001338657	down-regulated	protein_coding
ZNF534	ENSMMUG00000009888	17.26948469	-4.479544037	1.252145	-3.5775	0.0003469	0.001344005	down-regulated	protein_coding
RNF182	ENSMMUG00000002501	17.66431613	-4.511887087	1.261537	-3.5765	0.0003482	0.00134876	down-regulated	protein_coding
UGP2	ENSMMUG00000008770	154.7164508	-1.366998968	0.382306	-3.57567	0.0003493	0.001352312	down-regulated	protein_coding
LSM14A	ENSMMUG00000021614	126.6383851	-1.374272391	0.384396	-3.57515	0.00035	0.001354257	down-regulated	protein_coding
UGT1A1	ENSMMUG00000021216	3804.87112	-2.278916737	0.637512	-3.5747	0.0003506	0.001355467	down-regulated	protein_coding
LRTOMT	ENSMMUG00000021280	16.37634315	2.96528125	0.829486	3.574842	0.0003504	0.001355467	up-regulated	protein_coding
TMEM57	ENSMMUG00000018014	60.01902877	-2.554328145	0.714625	-3.57436	0.0003511	0.001356874	down-regulated	protein_coding
VAMP2	ENSMMUG00000021801	32.80405398	-4.374395672	1.223988	-3.57389	0.0003517	0.001358957	down-regulated	protein_coding
NDUFA8	ENSMMUG00000011450	130.5531848	-1.766457397	0.49449	-3.57228	0.0003539	0.001366587	down-regulated	protein_coding
IP6K2	ENSMMUG00000014730	49.13707752	-4.128679212	1.156013	-3.57148	0.000355	0.001370001	down-regulated	protein_coding
PPARGC1B	ENSMMUG00000004749	47.57636651	1.964368336	0.550172	3.570463	0.0003564	0.00137497	up-regulated	protein_coding
PMM1	ENSMMUG00000001853	67.16820247	-2.165685414	0.606813	-3.56895	0.0003584	0.001382288	down-regulated	protein_coding
MAT2B	ENSMMUG00000013980	65.99688661	-2.524689447	0.707542	-3.56825	0.0003594	0.001385479	down-regulated	protein_coding
TWSG1	ENSMMUG00000010726	34.80789071	-4.491475882	1.258886	-3.56782	0.00036	0.0013874	down-regulated	protein_coding
Y_RNA	ENSMMUG00000025594	6.592763407	4.996293387	1.401188	3.565756	0.0003628	0.001397982	up-regulated	misc_RNA
BRMS1L	ENSMMUG00000010665	24.47611295	2.936550351	0.823881	3.56429	0.0003648	0.001405435	up-regulated	protein_coding
FAM122B	ENSMMUG00000016948	17.36460086	-4.488029899	1.259902	-3.56221	0.0003678	0.001416134	down-regulated	protein_coding
SDSL	ENSMMUG00000020665	35.83987215	-4.532533449	1.272413	-3.56216	0.0003678	0.001416134	down-regulated	protein_coding
EEF1B2	ENSMMUG00000001054	417.9678849	-2.199662193	0.617763	-3.56069	0.0003699	0.001423698	down-regulated	protein_coding
FAM65A	ENSMMUG00000017105	121.5312972	1.134220929	0.318581	3.560226	0.0003705	0.001425752	up-regulated	protein_coding
PRAM1	ENSMMUG00000018808	6.99265841	3.591139738	1.0087	3.560166	0.0003706	0.001425752	up-regulated	protein_coding
IRF9	ENSMMUG00000012474	155.077548	-1.477228779	0.415059	-3.55908	0.0003722	0.001430825	down-regulated	protein_coding
PCYT1B	ENSMMUG00000012821	9.324955002	6.020008905	1.691469	3.559043	0.0003722	0.001430825	up-regulated	protein_coding
ICAM2	ENSMMUG00000014656	6.800903343	4.179442523	1.174324	3.559019	0.0003722	0.001430825	up-regulated	protein_coding
PLAU	ENSMMUG00000009906	75.80514127	-2.167157756	0.609014	-3.55847	0.000373	0.001433422	down-regulated	protein_coding
CYP17A1	ENSMMUG00000017601	32.1600427	3.554487772	0.999268	3.557093	0.000375	0.001440569	up-regulated	protein_coding
DLK2	ENSMMUG00000015574	21.62103273	2.681383407	0.754101	3.555735	0.0003769	0.001447637	up-regulated	protein_coding
CD274	ENSMMUG00000012235	6.518857271	3.775078646	1.061759	3.555495	0.0003773	0.001448564	up-regulated	protein_coding
EIF1AY	ENSMMUG00000000193	44.90643185	-2.946557678	0.828835	-3.55506	0.0003779	0.001450565	down-regulated	protein_coding

GOLPH3	ENSMMUG00000013957	87.61747404	-1.779603181	0.500632	-3.55471	0.0003784	0.001452106	down-regulated	protein_coding
DYNC1LI1	ENSMMUG00000005465	18.23635625	-4.556937528	1.282013	-3.55452	0.0003787	0.001452786	down-regulated	protein_coding
MRPL28	ENSMMUG00000000609	110.5802066	-3.257042604	0.916456	-3.55395	0.0003795	0.001455509	down-regulated	protein_coding
TRUB1	ENSMMUG00000001902	17.49329127	-4.497442279	1.26568	-3.55338	0.0003803	0.001458285	down-regulated	protein_coding
ADAM9	ENSMMUG00000006033	193.2324878	-1.554064246	0.437409	-3.55288	0.000381	0.001460648	down-regulated	protein_coding
ZNF619	ENSMMUG00000009437	15.13171063	2.618941011	0.737226	3.552426	0.0003817	0.001462788	up-regulated	protein_coding
SERPINB5	ENSMMUG00000014182	81.83062186	-1.716845134	0.483316	-3.55222	0.000382	0.001463151	down-regulated	protein_coding
KCNMA1	ENSMMUG00000018390	6.20156032	5.369666272	1.511623	3.552252	0.0003819	0.001463151	up-regulated	protein_coding
ERH	ENSMMUG00000021571	68.28896201	-3.246930236	0.914327	-3.55117	0.0003835	0.001468607	down-regulated	protein_coding
C2orf54	ENSMMUG00000011416	79.90653021	-2.857301547	0.804669	-3.5509	0.0003839	0.001469683	down-regulated	protein_coding
PGP	ENSMMUG00000019234	92.95961707	-2.771722246	0.780601	-3.55075	0.0003841	0.00147013	down-regulated	protein_coding
SPRYD3	ENSMMUG00000000329	108.2440718	-1.719783272	0.48437	-3.55055	0.0003844	0.001470839	down-regulated	protein_coding
GFRA3	ENSMMUG00000013272	9.241056093	6.752056045	1.902083	3.549821	0.0003855	0.001474543	up-regulated	protein_coding
TAPBP	ENSMMUG00000003875	178.1223198	-1.717456044	0.484066	-3.54798	0.0003882	0.001484077	down-regulated	protein_coding
FTSJ3	ENSMMUG00000016768	216.6446406	1.657351456	0.467368	3.546139	0.0003909	0.001493688	up-regulated	protein_coding
BMPR1A	ENSMMUG00000011852	18.94682299	-4.614205555	1.301509	-3.54527	0.0003922	0.00149819	down-regulated	protein_coding
CHKA	ENSMMUG00000023192	45.48437966	-2.630963343	0.742505	-3.54336	0.0003951	0.001508703	down-regulated	protein_coding
YPEL3	ENSMMUG00000000980	178.7681727	-2.202061175	0.621486	-3.54322	0.0003953	0.001509115	down-regulated	protein_coding
ATG5	ENSMMUG00000019679	72.09991945	-1.808663493	0.51061	-3.54216	0.0003969	0.001514766	down-regulated	protein_coding
OR2W1	ENSMMUG00000004377	9.128587516	9.104588622	2.57093	3.54136	0.0003981	0.001515276	up-regulated	protein_coding
ZNF648	ENSMMUG00000006589	9.128587516	9.104588622	2.57093	3.54136	0.0003981	0.001515276	up-regulated	protein_coding
DRAP1	ENSMMUG00000008655	103.7696406	-2.112973057	0.596603	-3.54167	0.0003976	0.001515276	down-regulated	protein_coding
CCL7	ENSMMUG00000009782	9.128587516	9.104588622	2.57093	3.54136	0.0003981	0.001515276	up-regulated	protein_coding
GADL1	ENSMMUG00000010033	9.128587516	9.104588622	2.57093	3.54136	0.0003981	0.001515276	up-regulated	protein_coding
ATP2B3	ENSMMUG00000011478	9.128587516	9.104588622	2.57093	3.54136	0.0003981	0.001515276	up-regulated	protein_coding
NR2E3	ENSMMUG00000019276	9.128587516	9.104588622	2.57093	3.54136	0.0003981	0.001515276	up-regulated	protein_coding
IGLV4-69	ENSMMUG00000031083	9.128587516	9.104588622	2.57093	3.54136	0.0003981	0.001515276	up-regulated	protein_coding
SNORD5	ENSMMUG00000036245	9.128587516	9.104588622	2.57093	3.54136	0.0003981	0.001515276	up-regulated	snoRNA
STRA8	ENSMMUG00000002318	11.41567093	6.643760012	1.876287	3.540909	0.0003988	0.001517465	up-regulated	protein_coding
AMT	ENSMMUG00000005452	89.72981773	2.083364884	0.588411	3.540664	0.0003991	0.001518055	up-regulated	protein_coding
CSE1L	ENSMMUG00000019636	122.6853528	-1.808498122	0.510834	-3.54029	0.0003997	0.001519813	down-regulated	protein_coding
C5H4ORF29	ENSMMUG00000020931	17.75038549	-4.519731379	1.277114	-3.53902	0.0004016	0.001526724	down-regulated	protein_coding
COMMD9	ENSMMUG00000007095	57.89335864	-2.27201001	0.642191	-3.53791	0.0004033	0.001532763	down-regulated	protein_coding
DPP4	ENSMMUG00000008387	287.7873877	-2.335500062	0.660364	-3.53669	0.0004052	0.00153945	down-regulated	protein_coding
SERINC2	ENSMMUG00000013188	762.813938	-1.614974848	0.456777	-3.53559	0.0004069	0.001545455	down-regulated	protein_coding
BBS7	ENSMMUG00000009819	16.80385467	-4.439488114	1.25582	-3.53513	0.0004076	0.001547712	down-regulated	protein_coding
DEPDC7	ENSMMUG00000002535	42.87166904	-3.338942805	0.944937	-3.53351	0.0004101	0.001556821	down-regulated	protein_coding
SLC51B	ENSMMUG00000004030	156.335377	-2.121220332	0.600473	-3.53258	0.0004115	0.001561859	down-regulated	protein_coding
FAM111A	ENSMMUG00000010274	86.20005797	1.230728665	0.348411	3.532404	0.0004118	0.001562498	up-regulated	protein_coding
CLTA	ENSMMUG00000006132	221.4374331	-1.4544949	0.411885	-3.53131	0.0004135	0.001568114	down-regulated	protein_coding
TFCP2L1	ENSMMUG00000008829	4.304663285	4.762062604	1.348884	3.530371	0.000415	0.001572865	up-regulated	protein_coding
GPRC5C	ENSMMUG00000013324	43.26440837	-2.899933321	0.82142	-3.53039	0.0004149	0.001572865	down-regulated	protein_coding
AVPR1B	ENSMMUG00000002255	8.429253733	4.911712576	1.391466	3.529884	0.0004157	0.001575345	up-regulated	protein_coding
MPLKIP	ENSMMUG00000001701	19.85006185	-4.678554252	1.32594	-3.52848	0.000418	0.001582873	down-regulated	protein_coding
KIDINS220	ENSMMUG00000021036	115.0613206	-1.750860425	0.4962	-3.52854	0.0004179	0.001582873	down-regulated	protein_coding
SYNGAP1	ENSMMUG00000010659	13.90231183	3.162418873	0.896402	3.527903	0.0004189	0.001585905	up-regulated	protein_coding
SLC52A2	ENSMMUG00000007374	116.0433398	-2.344061171	0.664466	-3.52773	0.0004191	0.001586493	down-regulated	protein_coding
GTF2I	ENSMMUG00000006823	204.7913589	-1.690459602	0.479358	-3.52651	0.0004211	0.001593418	down-regulated	protein_coding
SNORD42	ENSMMUG00000032856	5.607412819	5.264934979	1.493086	3.52621	0.0004216	0.001594797	up-regulated	snoRNA
TRMT2B	ENSMMUG00000010394	17.52908858	-4.499720947	1.27613	-3.52607	0.0004218	0.001595221	down-regulated	protein_coding
EP400	ENSMMUG00000017729	130.732062	1.353928315	0.384096	3.524976	0.0004235	0.00160139	up-regulated	protein_coding
DAPP1	ENSMMUG00000006829	7.105176931	3.248408794	0.921713	3.524317	0.0004246	0.001604947	up-regulated	protein_coding
HNRNPA1	ENSMMUG00000031613	22.95146139	-4.888400791	1.387965	-3.52199	0.0004283	0.001618233	down-regulated	protein_coding
DERA	ENSMMUG00000013309	44.35884092	-2.918650658	0.828794	-3.52156	0.000429	0.001620423	down-regulated	protein_coding
AREGB	ENSMMUG00000002314	91.46822497	-2.276516164	0.646587	-3.52082	0.0004302	0.001624543	down-regulated	protein_coding
ZUFSP	ENSMMUG00000001806	17.73080231	-4.516144401	1.282812	-3.5205	0.0004307	0.001626029	down-regulated	protein_coding
BCAR1	ENSMMUG00000002630	87.8011356	-1.641933121	0.466524	-3.51951	0.0004324	0.001631291	down-regulated	protein_coding
MDP1	ENSMMUG00000012476	81.41213691	-2.030950294	0.577046	-3.51956	0.0004323	0.001631291	down-regulated	protein_coding
TOX3	ENSMMUG00000013967	44.32242663	-3.973479722	1.129714	-3.51724	0.0004361	0.001644812	down-regulated	protein_coding
RNF8	ENSMMUG00000001287	44.81502236	-2.552595597	0.725871	-3.5166	0.0004371	0.001648124	down-regulated	protein_coding
DYNLT3	ENSMMUG00000013221	32.92088585	-4.408703477	1.253694	-3.51657	0.0004372	0.001648124	down-regulated	protein_coding
RFWD2	ENSMMUG00000002072	47.75773366	-2.333083274	0.663516	-3.51624	0.0004377	0.00164885	down-regulated	protein_coding
UNC5CL	ENSMMUG00000021168	87.83257766	-1.938055981	0.551366	-3.51501	0.0004397	0.001656095	down-regulated	protein_coding
MTIF3	ENSMMUG00000009030	66.3477293	1.901868848	0.541292	3.513571	0.0004421	0.001664618	up-regulated	protein_coding
CDK8	ENSMMUG00000014841	38.63435207	-3.762747672	1.071103	-3.51296	0.0004431	0.001667981	down-regulated	protein_coding
MTTP	ENSMMUG00000009091	724.5559609	-2.491375263	0.709281	-3.51253	0.0004439	0.001670236	down-regulated	protein_coding
NUAK1	ENSMMUG00000020951	11.31204724	3.285903193	0.936276	3.509547	0.0004489	0.001688667	up-regulated	protein_coding
MUM1	ENSMMUG00000003043	51.62191362	-2.450686333	0.698704	-3.50748	0.0004524	0.001701413	down-regulated	protein_coding
UBR3	ENSMMUG00000023062	44.42821082	-3.389971977	0.96712	-3.50522	0.0004562	0.001715422	down-regulated	protein_coding
MARCH9	ENSMMUG00000022143	32.26541113	-4.348656949	1.24115	-3.50373	0.0004588	0.001723672	down-regulated	protein_coding
ANKRD11	ENSMMUG00000017161	437.6190028	1.092502819	0.311845	3.503351	0.0004594	0.001725688	up-regulated	protein_coding
PRKAR1A	ENSMMUG00000002194	226.1411065	-1.083651049	0.309419	-3.50222	0.0004614	0.001732592	down-regulated	protein_coding
F2R	ENSMMUG00000021352	50.89442045	-3.584860448	1.024146	-3.50034	0.0004647	0.001744377	down-regulated	protein_coding
NAPB	ENSMMUG00000022388	16.50785852	-4.415108246	1.261768	-3.49914	0.0004668	0.001751289	down-regulated	protein_coding
C20ORF54	ENSMMUG00000021670	67.5060746	-2.177030391	0.622212	-3.49886	0.0004673	0.001752704	down-regulated	protein_coding
SLC25A18	ENSMMUG00000031121	5.157799521	4.510371374	1.289163	3.498681	0.0004676	0.001753403	up-regulated	protein_coding
PLAGL2	ENSMMUG00000000118	143.8810274	1.22922314	0.351358	3.498488	0.0004679	0.001754207	up-regulated	protein_coding
SLC16A2	ENSMMUG00000013165	13.43151969	4.733202179	1.353498	3.497014	0.0004705	0.001762994	up-regulated	protein_coding
DCAF11	ENSMMUG00000014391	185.9513062	-1.160796916	0.332023	-3.49613	0.0004721	0.001768356	down-regulated	protein_coding
USP36	ENSMMUG00000005352	143.6964373	1.371760078	0.392376	3.496037	0.0004722	0.001768525	up-regulated	protein_coding
SPPL2B	ENSMMUG00000000453	47.5716345	-3.07063215	0.878402	-3.4957	0.0004728	0.001770279	down-regulated	protein_coding
PSME2	ENSMMUG00000012473	103.3556256	-2.45056894	0.701077	-3.49544	0.0004733	0.00177111	down-regulated	protein_coding
CAPN10	ENSMMUG00000015628	70.28176728	-2.146992347	0.614362	-3.49467	0.0004746	0.00177524	down-regulated	protein_coding
SERPINB2	ENSMMUG00000016617	29.96390215	4.510407895	1.290869	3.494088	0.0004757	0.001778664	up-regulated	protein_coding
TTLL13	ENSMMUG00000010181	150.6132421	-1.486314618	0.425445	-3.49356	0.0004766	0.001781737	down-regulated	protein_coding
U2AF2	ENSMMUG00000004810	233.1287149	-1.827593269	0.523697	-3.48979	0.0004834	0.001805733	down-regulated	protein_coding
VWA9	ENSMMUG00000008095	17.16077524	-4.469205978	1.280659	-3.48977	0.0004834	0.001805733	down-regulated	protein_coding
HPRT1	ENSMMUG00000008303	36.89051817	-4.575083304	1.311401	-3.4887	0.0004854	0.001812505	down-regulated	protein_coding
CACNA1B	ENSMMUG00000009187	16.36829763	4.589114745	1.315464	3.488591	0.0004856	0.001812768	up-regulated	protein_coding
FCHSD2	ENSMMUG00000001294	71.16099941	1.749589048	0.501531	3.488493	0.0004858	0.00181295	up-regulated	protein_coding
PRPF40A	ENSMMUG00000010812	113.9807785	-1.685634954	0.483495	-3.48635	0.0004897	0.001827025	down-regulated	protein_coding
TOR1AIP1	ENSMMUG00000014873	63.48882549	1.730614928	0.496431	3.486117	0.0004901	0.001828166	up-regulated	protein_coding
STRAP	ENSMMUG00000013307	60.17546965	-1.960929925	0.562638	-3.48524	0.0004917	0.001833666	down-regulated	protein_coding
CASP16	ENSMMUG00000020971	172.6958832	2.130046153	0.611326	3.484306	0.0004934	0.001839614	up-regulated	protein_coding
EIF3I	ENSMMUG00000006119	142.9270865	-2.195231264	0.630084	-3.48403	0.0004939	0.001841016	down-regulated	protein_coding
GART	ENSMMUG00000022702	108.9073668	1.748133051	0.501814	3.48363	0.0004947	0.001843292	up-regulated	protein_coding
CYB5R4	ENSMMUG00000009585	16.22186704	-4.388977921	1.260059	-3.48315	0.0004955	0.0018461	down-regulated	protein_coding
ATP13A4	ENSMMUG00000009764	5.283584502	5.301073395	1.521958	3.483063	0.0004957	0.001846232	up-regulated	protein_coding
RBM14	ENSMMUG00000007830	84.24244397	-2.265040551	0.650352	-3.48279	0.0004962	0.001847608	down-regulated	protein_coding
MDH1	ENSMMUG00000006163	297.188896	-1.518748662	0.436123	-3.48238	0.000497	0.001849939	down-regulated	protein_coding

SPTLC1	ENSMMUG00000003006	31.04211303	-4.324525659	1.242503	-3.4805	0.0005005	0.001862541	down-regulated	protein_coding
CYHR1	ENSMMUG00000002659	76.43315562	-2.371764853	0.681589	-3.47976	0.0005019	0.00186719	down-regulated	protein_coding
KPNB1	ENSMMUG00000019760	307.3708676	1.062051899	0.305265	3.479113	0.0005031	0.001871193	up-regulated	protein_coding
FREM2	ENSMMUG00000002702	14.83933181	3.59651009	1.034223	3.477501	0.0005061	0.001880114	up-regulated	protein_coding
C5ORF25	ENSMMUG00000003919	47.67310541	1.531725579	0.440451	3.477626	0.0005059	0.001880114	up-regulated	protein_coding
RABGGTA	ENSMMUG00000012447	42.23677931	-3.288590162	0.945747	-3.47724	0.0005066	0.001881332	down-regulated	protein_coding
PSAT1	ENSMMUG00000011868	55.59087185	-2.730182493	0.78518	-3.47714	0.0005068	0.001881531	down-regulated	protein_coding
MECOM	ENSMMUG00000002242	150.3680931	-1.298333988	0.373434	-3.47675	0.0005075	0.001883488	down-regulated	protein_coding
ATP6V0D1	ENSMMUG00000029105	46.29103589	-3.264424886	0.938937	-3.47672	0.0005076	0.001883488	down-regulated	protein_coding
PPP4C	ENSMMUG00000000982	272.8842506	-1.762061588	0.507022	-3.47531	0.0005103	0.001891936	down-regulated	protein_coding
RNF5	ENSMMUG00000018438	157.3540077	-1.408255798	0.405216	-3.47532	0.0005102	0.001891936	down-regulated	protein_coding
HIST1H2BG	ENSMMUG00000029911	7.350699671	4.574375719	1.317115	3.473027	0.0005146	0.001907624	up-regulated	protein_coding
DULLARD	ENSMMUG00000010547	68.21102806	-1.916667801	0.552027	-3.47205	0.0005165	0.001913552	down-regulated	protein_coding
ABCC8	ENSMMUG00000023581	29.56055547	4.586499049	1.320972	3.472065	0.0005165	0.001913552	up-regulated	protein_coding
XRN2	ENSMMUG00000011405	105.9298004	-1.565709629	0.451114	-3.47076	0.000519	0.001921775	down-regulated	protein_coding
ELOF1	ENSMMUG00000001476	17.92635865	-4.531274518	1.306094	-3.46933	0.0005218	0.001931532	down-regulated	protein_coding
CABP4	ENSMMUG00000005491	19.66819541	2.397672758	0.69141	3.467803	0.0005247	0.001942042	up-regulated	protein_coding
STARD3NL	ENSMMUG00000023659	38.10961505	-3.125921194	0.901544	-3.4673	0.0005257	0.001945184	down-regulated	protein_coding
SREK1IP1	ENSMMUG00000015040	20.68728473	-4.737985988	1.366801	-3.46648	0.0005273	0.001950618	down-regulated	protein_coding
PRPF31	ENSMMUG00000022297	200.4934369	1.565381878	0.451636	3.466023	0.0005282	0.001953416	up-regulated	protein_coding
ACE2	ENSMMUG00000014048	599.7174544	-2.87467109	0.82942	-3.46588	0.0005285	0.001953946	down-regulated	protein_coding
CALU	ENSMMUG00000009616	112.2232446	-1.81497408	0.523739	-3.46542	0.0005294	0.001956792	down-regulated	protein_coding
ADAMTSL5	ENSMMUG00000003815	72.6858472	-1.944736251	0.561486	-3.46355	0.0005331	0.001969884	down-regulated	protein_coding
TBCEL	ENSMMUG00000000086	14.10122027	2.275285925	0.657264	3.461753	0.0005367	0.001982079	up-regulated	protein_coding
VAMP7	ENSMMUG00000002015	46.07783355	-2.659771204	0.768569	-3.46068	0.0005388	0.001989488	down-regulated	protein_coding
RBPMS	ENSMMUG00000000458	141.6043533	1.595623267	0.46117	3.45995	0.0005403	0.001994357	up-regulated	protein_coding
GUCA1C	ENSMMUG00000000872	5.771068143	4.526483719	1.308296	3.459832	0.0005405	0.001994707	up-regulated	protein_coding
CENPH	ENSMMUG00000030035	14.85642439	3.255403126	0.941217	3.458719	0.0005428	0.00200245	up-regulated	protein_coding
LACTB2	ENSMMUG00000012513	18.6569273	-4.591282476	1.327522	-3.45854	0.0005431	0.002003284	down-regulated	protein_coding
TTLL9	ENSMMUG00000000971	4.323681257	4.687654791	1.355701	3.457734	0.0005447	0.002008732	up-regulated	protein_coding
TRAPPC2L	ENSMMUG00000012433	52.76164005	-2.299218461	0.66504	-3.45726	0.0005457	0.002011213	down-regulated	protein_coding
RND3	ENSMMUG00000016040	58.34125835	-2.628036898	0.760144	-3.45729	0.0005456	0.002011213	down-regulated	protein_coding
GEMIN7	ENSMMUG00000022929	19.12206018	-4.624441312	1.338843	-3.45406	0.0005522	0.002033131	down-regulated	protein_coding
TRAF5	ENSMMUG00000031427	8.912815921	3.403612019	0.985392	3.45407	0.0005522	0.002033131	up-regulated	protein_coding
RIMS2	ENSMMUG00000019407	9.479224237	3.102300121	0.898473	3.452859	0.0005547	0.002041131	up-regulated	protein_coding
RAB28	ENSMMUG00000022025	15.77076923	-4.347831181	1.259764	-3.45131	0.0005579	0.002052375	down-regulated	protein_coding
RAB14	ENSMMUG00000005595	234.5916313	-1.227850817	0.35579	-3.45105	0.0005584	0.00205361	down-regulated	protein_coding
CCDC101	ENSMMUG00000011370	91.56311855	1.652348808	0.478802	3.451004	0.0005585	0.00205361	up-regulated	protein_coding
NAA38	ENSMMUG00000015822	20.05949656	-4.69339938	1.360093	-3.45079	0.0005589	0.002054679	down-regulated	protein_coding
MYSM1	ENSMMUG00000011800	15.70640226	-4.342840235	1.258923	-3.44965	0.0005613	0.002062345	down-regulated	protein_coding
MAOB	ENSMMUG00000019492	100.9485585	-3.320919128	0.962665	-3.44971	0.0005612	0.002062345	down-regulated	protein_coding
RASSF6	ENSMMUG00000003833	17.95074587	-4.536697627	1.315466	-3.44874	0.0005632	0.002068748	down-regulated	protein_coding
VAMP3	ENSMMUG00000018559	34.52479057	-4.452846408	1.291178	-3.44867	0.0005634	0.002068748	down-regulated	protein_coding
ADGRB1	ENSMMUG00000019877	5.596841934	5.985523583	1.736116	3.447651	0.0005655	0.002076034	up-regulated	protein_coding
ZNF45	ENSMMUG00000021474	20.09508128	1.990342273	0.577361	3.447311	0.0005662	0.002077571	up-regulated	protein_coding
STRBP	ENSMMUG00000022919	93.88901024	-1.442941298	0.418566	-3.44734	0.0005661	0.002077571	down-regulated	protein_coding
SYNPO2L	ENSMMUG00000004597	5.924943938	5.719427633	1.659236	3.447025	0.0005668	0.002079231	up-regulated	protein_coding
FAM57A	ENSMMUG00000023373	163.092347	1.796769768	0.521299	3.446717	0.0005674	0.002080524	up-regulated	protein_coding
ABCD4	ENSMMUG00000004041	102.8397511	-1.932561992	0.560883	-3.44557	0.0005699	0.002088048	down-regulated	protein_coding
REEP4	ENSMMUG00000013027	51.48830066	-3.616153372	1.049498	-3.4456	0.0005698	0.002088048	down-regulated	protein_coding
MAPRE3	ENSMMUG00000019732	100.5770839	-2.028035405	0.588599	-3.44553	0.0005699	0.002088048	down-regulated	protein_coding
CTBP1	ENSMMUG00000008323	206.6047166	-1.901262827	0.551836	-3.44534	0.0005703	0.002088994	down-regulated	protein_coding
SFMBT2	ENSMMUG00000019194	8.031878914	6.519333865	1.892483	3.444858	0.0005714	0.002092177	up-regulated	protein_coding
C5H4ORF27	ENSMMUG00000010232	18.1252657	-4.547125052	1.320189	-3.4443	0.0005725	0.002095975	down-regulated	protein_coding
R3HCC1L	ENSMMUG00000010281	15.60647278	-4.33310235	1.258306	-3.4436	0.000574	0.002100853	down-regulated	protein_coding
TMEM65	ENSMMUG00000015122	116.899379	-2.942762333	0.8546	-3.44344	0.0005744	0.00210157	down-regulated	protein_coding
CCDC171	ENSMMUG00000016731	8.398904529	3.907712828	1.135058	3.442743	0.0005758	0.002106424	up-regulated	protein_coding
SAR1B	ENSMMUG00000004417	73.09500118	-2.551639368	0.74157	-3.44086	0.0005799	0.00212058	down-regulated	protein_coding
SLC3A1	ENSMMUG00000006310	295.8188202	2.611186435	0.759318	3.438858	0.0005842	0.002135782	up-regulated	protein_coding
GLT25D2	ENSMMUG00000016405	15.05719073	4.658846362	1.354803	3.438764	0.0005844	0.002135972	up-regulated	protein_coding
CCDC154	ENSMMUG00000021102	16.24533106	3.405224147	0.990659	3.437331	0.0005875	0.00214675	up-regulated	protein_coding
NDFIP2	ENSMMUG00000007531	210.054172	-1.522155577	0.442889	-3.43688	0.0005885	0.002149571	down-regulated	protein_coding
ASPG	ENSMMUG00000014295	13.87613536	6.890295055	2.004837	3.436835	0.0005886	0.002149571	up-regulated	protein_coding
SH2B1	ENSMMUG00000023524	142.0373861	-1.571606279	0.457335	-3.43645	0.0005894	0.002152095	down-regulated	protein_coding
PSMG3	ENSMMUG00000013083	23.52031864	-4.923269534	1.432761	-3.43621	0.0005899	0.002153418	down-regulated	protein_coding
GID8	ENSMMUG00000005712	41.47741671	-2.812340192	0.818688	-3.43518	0.0005922	0.002161074	down-regulated	protein_coding
ZMAT2	ENSMMUG00000004484	30.34771105	-4.289130864	1.248709	-3.43485	0.0005929	0.002163139	down-regulated	protein_coding
ZNF654	ENSMMUG00000032107	37.41728485	2.265542736	0.659641	3.434506	0.0005936	0.002165335	up-regulated	protein_coding
ZNF768	ENSMMUG00000016879	81.21521123	-1.796072953	0.523028	-3.43399	0.0005948	0.00216835	down-regulated	protein_coding
SMCR7	ENSMMUG00000018359	58.48338942	-2.255457895	0.656911	-3.43343	0.000596	0.002171729	down-regulated	protein_coding
CTSS	ENSMMUG00000010670	51.78293644	-2.728619763	0.794753	-3.43329	0.0005963	0.002171967	down-regulated	protein_coding
ACSL5	ENSMMUG00000001155	550.5769179	-2.087524875	0.608188	-3.43237	0.0005983	0.002177427	down-regulated	protein_coding
MAP3K15	ENSMMUG00000017339	8.416574437	4.106269345	1.196313	3.432438	0.0005982	0.002177427	up-regulated	protein_coding
NFKBIA	ENSMMUG00000020374	79.8370355	-2.152404821	0.627087	-3.43239	0.0005983	0.002177427	down-regulated	protein_coding
PRDX2	ENSMMUG00000005689	295.6088019	-1.873392335	0.545816	-3.43228	0.0005985	0.002177596	down-regulated	protein_coding
CSMD2	ENSMMUG00000002741	29.64623296	2.91200868	0.848455	3.432131	0.0005989	0.002178213	up-regulated	protein_coding
ZNHIT2	ENSMMUG00000018775	115.7601749	2.523135065	0.735296	3.431454	0.0006004	0.002182534	up-regulated	protein_coding
SIM1	ENSMMUG00000022285	5.13572691	5.929790387	1.728405	3.430787	0.0006018	0.002187348	up-regulated	protein_coding
ABCC5	ENSMMUG00000005515	226.8879301	-1.70763713	0.497803	-3.43035	0.0006028	0.002190317	down-regulated	protein_coding
CUL7	ENSMMUG00000002451	180.6424494	1.376910817	0.401437	3.429952	0.0006037	0.002191833	up-regulated	protein_coding
ZBTB1	ENSMMUG00000004748	15.87937919	-4.358188817	1.270605	-3.43001	0.0006036	0.002191833	down-regulated	protein_coding
FAM188A	ENSMMUG00000023766	38.86029513	-2.717681012	0.792326	-3.43	0.0006036	0.002191833	down-regulated	protein_coding
CNTRL	ENSMMUG00000012668	58.02772858	2.283275777	0.665721	3.429778	0.0006041	0.002192676	up-regulated	protein_coding
PCDH9	ENSMMUG00000005620	4.471682274	5.448646896	1.58886	3.42928	0.0006052	0.002196142	up-regulated	protein_coding
P2RX7	ENSMMUG00000006298	14.52313624	4.773019126	1.391914	3.429105	0.0006056	0.002196436	up-regulated	protein_coding
NOL7	ENSMMUG00000008681	66.87147502	-2.164041104	0.631069	-3.42916	0.0006054	0.002196436	down-regulated	protein_coding
FAM89B	ENSMMUG00000031183	61.37146885	-3.163584657	0.922882	-3.42794	0.0006082	0.002204752	down-regulated	protein_coding
KIAA1539	ENSMMUG00000011862	84.42102829	-1.873496151	0.546557	-3.42782	0.0006085	0.002205195	down-regulated	protein_coding
EXOC2	ENSMMUG00000023558	38.50271438	-3.15839134	0.921464	-3.42758	0.000609	0.002205973	down-regulated	protein_coding
C14H11ORF75	ENSMMUG00000030946	22.16624901	-4.837671461	1.411395	-3.42758	0.000609	0.002205973	down-regulated	protein_coding
UFD1L	ENSMMUG00000031106	66.50080466	-2.058855271	0.600759	-3.42709	0.0006101	0.002209377	down-regulated	protein_coding
TIAL1	ENSMMUG00000001472	120.5872029	-1.585293294	0.462673	-3.42638	0.0006117	0.002214595	down-regulated	protein_coding
RAB13	ENSMMUG00000017586	44.04126945	-3.30833675	0.965583	-3.42626	0.000612	0.002215028	down-regulated	protein_coding
USF1	ENSMMUG00000002003	47.67527489	-3.011171446	0.879228	-3.42479	0.0006153	0.002225919	down-regulated	protein_coding
PIGH	ENSMMUG00000021327	42.65722045	1.684905427	0.491966	3.424839	0.0006152	0.002225919	up-regulated	protein_coding
CCHCR1	ENSMMUG00000015679	80.69282547	2.378507914	0.694667	3.423953	0.0006172	0.002231633	up-regulated	protein_coding
PARD3B	ENSMMUG00000014814	56.32261226	2.389988772	0.698271	3.422724	0.00062	0.002240603	up-regulated	protein_coding
ACADSB	ENSMMUG00000000684	51.03060544	-3.150533297	0.920758	-3.42167	0.0006224	0.002248701	down-regulated	protein_coding
NMT1	ENSMMUG00000013586	130.2987343	-1.569945838	0.45897	-3.42058	0.0006249	0.002257163	down-regulated	protein_coding
DDX56	ENSMMUG00000002645	99.91002192	-1.863234106	0.544859	-3.41966	0.000627	0.002263903	down-regulated	protein_coding

SUV420H1	ENSMMUG00000014237	61.08336807	-2.617945705	0.765563	-3.41963	0.0006271	0.002263903	down-regulated	protein_coding
CDK5	ENSMMUG00000020451	18.05190022	-4.541574137	1.328163	-3.41944	0.0006275	0.002264926	down-regulated	protein_coding
TEX261	ENSMMUG00000002093	50.67266257	-2.796784593	0.818096	-3.41865	0.0006293	0.002270925	down-regulated	protein_coding
WDR61	ENSMMUG00000014207	42.50539828	-3.244144311	0.948992	-3.41852	0.0006296	0.00227147	down-regulated	protein_coding
KBTBD2	ENSMMUG00000004944	35.83828945	-3.664250836	1.071953	-3.41829	0.0006301	0.002271758	down-regulated	protein_coding
SLC25A13	ENSMMUG00000005250	84.16514432	-1.833186962	0.53629	-3.41827	0.0006302	0.002271758	down-regulated	protein_coding
B3GNT4	ENSMMUG00000012654	18.80459248	4.724447494	1.382084	3.41835	0.00063	0.002271758	up-regulated	protein_coding
MICAL1	ENSMMUG00000023221	389.4174748	1.380222585	0.40387	3.417492	0.000632	0.002277713	up-regulated	protein_coding
NUCB2	ENSMMUG00000005895	18.65208383	-4.588439251	1.342703	-3.41732	0.0006324	0.002278607	down-regulated	protein_coding
C1GALT1C1	ENSMMUG00000023574	28.67658969	-4.201886203	1.230081	-3.41594	0.0006356	0.002288956	down-regulated	protein_coding
ZSCAN18	ENSMMUG00000020503	50.65265011	-2.463387204	0.721201	-3.41567	0.0006362	0.002290635	down-regulated	protein_coding
KCNH2	ENSMMUG00000020436	11.38314844	3.316169862	0.97128	3.414227	0.0006396	0.002302256	up-regulated	protein_coding
LACTB	ENSMMUG00000003544	39.55360201	-3.137731673	0.919277	-3.41326	0.0006419	0.002309858	down-regulated	protein_coding
LARGE	ENSMMUG00000012113	82.52772577	1.304291602	0.382139	3.413136	0.0006422	0.002310319	up-regulated	protein_coding
TXNL4A	ENSMMUG00000001613	75.30331202	-2.630816376	0.770917	-3.41258	0.0006435	0.002314431	down-regulated	protein_coding
TRO	ENSMMUG00000008422	9.262385997	6.486687869	1.90161	3.411156	0.0006469	0.002325972	up-regulated	protein_coding
RPL37A	ENSMMUG00000019544	327.9689268	-2.15667948	0.632261	-3.41106	0.0006471	0.002326194	down-regulated	protein_coding
TSGA13	ENSMMUG00000004803	10.34822646	6.001404387	1.759781	3.410314	0.0006489	0.002331984	up-regulated	protein_coding
MFSD1	ENSMMUG00000023510	70.29563932	-1.927733272	0.565474	-3.40906	0.0006519	0.002342159	down-regulated	protein_coding
PPP1R13B	ENSMMUG00000018123	148.4591888	1.346654006	0.395038	3.408923	0.0006522	0.002342717	up-regulated	protein_coding
POLB	ENSMMUG00000020793	16.22776993	-4.388081129	1.287507	-3.4082	0.0006539	0.002348331	down-regulated	protein_coding
CALD1	ENSMMUG00000006527	91.74925629	1.473351126	0.432324	3.407977	0.0006545	0.002349662	up-regulated	protein_coding
ZBTB8A	ENSMMUG00000020058	32.43060209	2.837523089	0.832919	3.406721	0.0006575	0.0023587	up-regulated	protein_coding
mml-mir-219-2	ENSMMUG00000026874	6.213714233	4.88432536	1.433682	3.40684	0.0006572	0.0023587	up-regulated	miRNA
PABPC3	ENSMMUG00000017786	1423.618159	-1.307378645	0.383773	-3.40665	0.0006577	0.002358756	down-regulated	protein_coding
UBTD1	ENSMMUG00000020031	30.51929868	-4.293764821	1.260643	-3.40601	0.0006592	0.002363643	down-regulated	protein_coding
MTA1	ENSMMUG00000017313	116.6663361	-1.918106754	0.563314	-3.40504	0.0006615	0.002371489	down-regulated	protein_coding
SLC35B4	ENSMMUG00000000916	34.8927756	1.532768416	0.450273	3.404088	0.0006639	0.002379147	up-regulated	protein_coding
NEBL	ENSMMUG00000010589	166.83023	-2.940619651	0.864404	-3.4019	0.0006692	0.002397633	down-regulated	protein_coding
USP39	ENSMMUG00000017820	66.42607748	-1.831665032	0.538662	-3.4004	0.0006729	0.002410284	down-regulated	protein_coding
VPS51	ENSMMUG00000018772	112.5193235	-1.720733903	0.506121	-3.39985	0.0006742	0.002414524	down-regulated	protein_coding
ICA1L	ENSMMUG00000017364	15.21919314	4.345908656	1.27834	3.39965	0.0006747	0.002415646	up-regulated	protein_coding
PDCD6IP	ENSMMUG00000014069	338.7330223	-1.594778855	0.469124	-3.39948	0.0006751	0.002416497	down-regulated	protein_coding
METTL11A	ENSMMUG00000006057	145.0322657	1.865385456	0.548929	3.398226	0.0006782	0.002427032	up-regulated	protein_coding
INF2	ENSMMUG00000016551	50.72808708	-2.536772903	0.74663	-3.39763	0.0006797	0.002431692	down-regulated	protein_coding
mml-mir-324	ENSMMUG00000032650	12.45388676	3.381690761	0.995561	3.396769	0.0006819	0.002438758	up-regulated	miRNA
KLK1	ENSMMUG00000006371	58.89021177	1.395207878	0.410858	3.395836	0.0006842	0.002446462	up-regulated	protein_coding
TMEM248	ENSMMUG00000015843	72.0401588	-2.30187931	0.678001	-3.3951	0.000686	0.002452448	down-regulated	protein_coding
ADORA2B	ENSMMUG00000020399	57.16295884	-2.244438415	0.661331	-3.39382	0.0006892	0.002463277	down-regulated	protein_coding
FBXW4	ENSMMUG00000006753	126.9891353	1.569348733	0.462569	3.392677	0.0006921	0.002472352	up-regulated	protein_coding
G6PD	ENSMMUG00000017409	108.0796807	-2.555229697	0.753268	-3.39219	0.0006934	0.002476116	down-regulated	protein_coding
NSF	ENSMMUG00000005548	60.30623395	-2.588770178	0.763221	-3.3919	0.0006941	0.00247812	down-regulated	protein_coding
RNF24	ENSMMUG00000004703	17.77321356	2.91913599	0.860745	3.391406	0.0006954	0.002481343	up-regulated	protein_coding
MRFAP1	ENSMMUG00000021695	452.146985	-1.204636787	0.35538	-3.38971	0.0006997	0.002496093	down-regulated	protein_coding
NDUFAB1	ENSMMUG00000020522	117.5466975	-1.888634849	0.557214	-3.38943	0.0007004	0.002498067	down-regulated	protein_coding
MBTPS1	ENSMMUG00000020053	160.6871556	-1.303588761	0.384693	-3.38865	0.0007024	0.002504515	down-regulated	protein_coding
HNRNPH3	ENSMMUG00000009792	119.548053	-2.27386542	0.671465	-3.38642	0.0007081	0.002523664	down-regulated	protein_coding
SLC25A22	ENSMMUG00000007513	42.08529412	-3.908878766	1.154369	-3.38616	0.0007088	0.002525444	down-regulated	protein_coding
XRCC6	ENSMMUG00000001856	411.3823817	-1.412010816	0.417015	-3.38599	0.0007092	0.002526338	down-regulated	protein_coding
SERPINF1	ENSMMUG00000022194	6.715886632	3.220894117	0.951622	3.384638	0.0007127	0.002537573	up-regulated	protein_coding
TTC18	ENSMMUG00000011285	16.84373075	3.047361805	0.900372	3.384558	0.0007129	0.002537671	up-regulated	protein_coding
CTNNAL1	ENSMMUG00000000690	47.95782649	-2.705663075	0.799456	-3.38438	0.0007134	0.00253868	down-regulated	protein_coding
SLC30A4	ENSMMUG00000011510	40.18676927	3.188471108	0.942203	3.384058	0.0007142	0.002541014	up-regulated	protein_coding
MAMU-DQB	ENSMMUG00000029799	81.59572379	-3.278469546	0.969066	-3.38312	0.0007167	0.002549032	down-regulated	protein_coding
KLHL32	ENSMMUG00000005093	9.474794841	3.01857046	0.892821	3.380934	0.0007224	0.002568791	up-regulated	protein_coding
CPEB2	ENSMMUG00000001384	64.80747456	-2.441371739	0.722192	-3.3805	0.0007235	0.002572173	down-regulated	protein_coding
CD1D	ENSMMUG00000020156	19.14840283	-4.629348097	1.369713	-3.3798	0.0007254	0.002577664	down-regulated	protein_coding
PHOSPHO1	ENSMMUG00000002436	116.0718165	-3.082206217	0.91205	-3.37943	0.0007264	0.002580315	down-regulated	protein_coding
FAM47E-STBD1	ENSMMUG00000020915	38.28184784	-3.701221745	1.095431	-3.37878	0.0007281	0.002585735	down-regulated	protein_coding
MED21	ENSMMUG00000003218	34.56213817	-3.607945722	1.068153	-3.37774	0.0007308	0.002594867	down-regulated	protein_coding
GDF11	ENSMMUG00000006288	11.29274408	3.317923556	0.98304	3.375167	0.0007377	0.002618627	up-regulated	protein_coding
MCMBP	ENSMMUG00000009559	90.20570515	-1.900406392	0.563181	-3.37442	0.0007397	0.002625126	down-regulated	protein_coding
SCLT1	ENSMMUG00000002146	15.88548378	-4.359634552	1.292023	-3.37427	0.0007401	0.00262585	down-regulated	protein_coding
RARS	ENSMMUG00000012516	104.2900214	-1.610970497	0.477487	-3.37386	0.0007412	0.002629009	down-regulated	protein_coding
M6PR	ENSMMUG00000017846	143.2890493	-1.681841958	0.498501	-3.3738	0.0007414	0.002629009	down-regulated	protein_coding
EDEM2	ENSMMUG00000010032	41.16533456	-3.261009101	0.966596	-3.37371	0.0007416	0.002629269	down-regulated	protein_coding
KCNQ1	ENSMMUG00000007742	414.4231782	-1.893090574	0.561223	-3.37315	0.0007431	0.002633898	down-regulated	protein_coding
JAK3	ENSMMUG00000010996	18.18119551	3.071387194	0.910615	3.372873	0.0007439	0.002635911	up-regulated	protein_coding
TOLLIP	ENSMMUG00000003104	54.18380704	-2.124498342	0.6299	-3.37275	0.0007442	0.00263639	down-regulated	protein_coding
RBM22	ENSMMUG00000020683	72.25218202	-1.649202544	0.489069	-3.37213	0.0007459	0.002641712	down-regulated	protein_coding
GLRX	ENSMMUG00000005308	98.10727924	-2.725043698	0.808145	-3.37197	0.0007463	0.002642566	down-regulated	protein_coding
SLC27A4	ENSMMUG00000014148	117.5042807	-1.27489319	0.378184	-3.37109	0.0007487	0.002650373	down-regulated	protein_coding
U2AF1	ENSMMUG00000022769	81.0649378	-3.080881407	0.914034	-3.37064	0.0007499	0.002654024	down-regulated	protein_coding
RBMS2	ENSMMUG00000019330	36.702907	-3.638637613	1.079617	-3.3703	0.0007509	0.002656619	down-regulated	protein_coding
DLST	ENSMMUG00000004260	246.5090778	-1.326321376	0.393648	-3.36931	0.0007536	0.002665564	down-regulated	protein_coding
LSM6	ENSMMUG00000005524	18.02758734	-4.539126335	1.347455	-3.36867	0.0007553	0.00267111	down-regulated	protein_coding
EXOC1	ENSMMUG00000023427	63.06853567	-1.80289304	0.535206	-3.3686	0.0007555	0.00267111	down-regulated	protein_coding
TCEB1	ENSMMUG00000005155	50.13190033	-2.725401418	0.809191	-3.36806	0.000757	0.002675683	down-regulated	protein_coding
TMEM161B	ENSMMUG00000009319	31.03496288	-4.321117917	1.283052	-3.36784	0.0007576	0.002677102	down-regulated	protein_coding
SP110	ENSMMUG00000012197	15.53701193	2.794331869	0.829782	3.367551	0.0007584	0.002678596	up-regulated	protein_coding
PHYH	ENSMMUG00000023798	123.2575438	-1.949265652	0.578832	-3.36759	0.0007583	0.002678596	down-regulated	protein_coding
TMEM200B	ENSMMUG00000011378	8.414862103	4.344417483	1.290266	3.36707	0.0007597	0.002682603	up-regulated	protein_coding
KCNK5	ENSMMUG00000008993	121.6448197	-1.849893968	0.549452	-3.3668	0.0007605	0.002683839	down-regulated	protein_coding
HSPH1	ENSMMUG00000013707	61.68386558	-3.160893828	0.93886	-3.36674	0.0007606	0.002683839	down-regulated	protein_coding
FAM100A	ENSMMUG00000029250	38.36434868	-3.763189768	1.117754	-3.36674	0.0007606	0.002683839	down-regulated	protein_coding
CDK7	ENSMMUG00000018621	17.84869884	-4.524935618	1.344302	-3.36601	0.0007626	0.002690243	down-regulated	protein_coding
LDLRAD4	ENSMMUG00000004032	24.30321313	3.023848403	0.898549	3.365255	0.0007647	0.002696945	up-regulated	protein_coding
SERTAD1	ENSMMUG00000016027	48.10322514	-3.498927865	1.039767	-3.36511	0.0007651	0.00269703	down-regulated	protein_coding
SCAMP3	ENSMMUG00000018313	76.956865	-1.917897482	0.569932	-3.36514	0.0007651	0.00269703	down-regulated	protein_coding
FRMPD3	ENSMMUG00000022293	6.474724039	6.650786653	1.976856	3.364326	0.0007673	0.002703353	up-regulated	protein_coding
GAL3ST2	ENSMMUG00000016941	9.188396097	7.627863354	2.267987	3.363274	0.0007702	0.002712995	up-regulated	protein_coding
COX16	ENSMMUG00000011089	73.13029484	-2.793678673	0.830753	-3.36283	0.0007715	0.002715691	down-regulated	protein_coding
PHOSPHO2	ENSMMUG00000020585	58.53325675	-2.268304965	0.674526	-3.36281	0.0007715	0.002715691	down-regulated	protein_coding
SS18L2	ENSMMUG00000031677	32.58955402	-4.391880091	1.306021	-3.36279	0.0007716	0.002715691	down-regulated	protein_coding
CMBL	ENSMMUG00000020113	138.407336	-1.289548531	0.383507	-3.36251	0.0007724	0.002717779	down-regulated	protein_coding
SLC35F5	ENSMMUG00000008875	58.87670117	-3.558161881	1.058929	-3.36015	0.000779	0.002740436	down-regulated	protein_coding
UBA2	ENSMMUG00000005884	86.41342953	-1.898702543	0.565197	-3.35937	0.0007812	0.002747546	down-regulated	protein_coding
GJB2	ENSMMUG00000010522	45.24189868	-2.274000154	0.677086	-3.35851	0.0007836	0.002755387	down-regulated	protein_coding
CLIP3	ENSMMUG00000019701	8.768493097	3.448825297	1.026929	3.358387	0.000784	0.002755932	up-regulated	protein_coding
PAFAH1B2	ENSMMUG00000002050	46.15074025	-3.239408593	0.964818	-3.35753	0.0007864	0.002763762	down-regulated	protein_coding

IL18	ENSMMUG00000015248	46.08040412	-3.98067836	1.185881	-3.35673	0.0007887	0.002771161	down-regulated	protein_coding
NOL11	ENSMMUG00000002414	30.06622265	-4.241009208	1.263581	-3.35634	0.0007898	0.002774336	down-regulated	protein_coding
GOLGA7	ENSMMUG00000016120	68.61838954	-1.649725892	0.491613	-3.35574	0.0007915	0.002779651	down-regulated	protein_coding
MRPL10	ENSMMUG00000021486	73.36833735	-2.06422922	0.61515	-3.35565	0.0007918	0.0027799	down-regulated	protein_coding
FBXL4	ENSMMUG00000001659	16.38093135	-4.404664925	1.312941	-3.35481	0.0007942	0.002786991	down-regulated	protein_coding
HNRNPLL	ENSMMUG00000002338	44.39345151	-2.287844916	0.682009	-3.35456	0.0007949	0.002788751	down-regulated	protein_coding
HID1	ENSMMUG00000023406	305.4597298	-1.671312212	0.498404	-3.35333	0.0007985	0.002800549	down-regulated	protein_coding
SLC25A11	ENSMMUG00000002765	136.6934255	-1.849304751	0.551792	-3.35145	0.0008039	0.002818175	down-regulated	protein_coding
TMEM42	ENSMMUG00000003650	18.59169412	-4.584043788	1.367946	-3.35104	0.0008051	0.002821357	down-regulated	protein_coding
C5ORF15	ENSMMUG00000000489	83.61635229	-2.280954105	0.680742	-3.35069	0.0008061	0.002823873	down-regulated	protein_coding
MRPL15	ENSMMUG00000005539	34.05867447	-4.42971073	1.322171	-3.35033	0.0008071	0.002826811	down-regulated	protein_coding
SETBP1	ENSMMUG00000004997	6.531640342	3.848984441	1.148904	3.350134	0.0008077	0.002828131	up-regulated	protein_coding
AHDC1	ENSMMUG00000009868	167.1754824	1.255648381	0.374859	3.349655	0.0008091	0.002832334	up-regulated	protein_coding
SAT2	ENSMMUG00000013511	88.03850634	-2.278935336	0.680394	-3.34944	0.0008098	0.002833006	down-regulated	protein_coding
KLHDC8A	ENSMMUG00000018585	12.36314594	6.627655177	1.978712	3.34948	0.0008096	0.002833006	up-regulated	protein_coding
SLC25A38	ENSMMUG00000022963	31.37423588	-4.334835623	1.294219	-3.34938	0.0008099	0.002833006	down-regulated	protein_coding
XIRP2	ENSMMUG00000013251	10.82068988	4.031624199	1.203932	3.348715	0.0008119	0.002839146	up-regulated	protein_coding
NFS1	ENSMMUG00000010951	94.22422281	1.370169743	0.409186	3.348526	0.0008124	0.00284038	up-regulated	protein_coding
SNORA16	ENSMMUG00000025267	8.48934513	4.719158392	1.410084	3.346722	0.0008177	0.002858228	up-regulated	snoRNA
C11H12ORF44	ENSMMUG00000015392	55.87288216	-2.439977065	0.729096	-3.34658	0.0008182	0.00285899	down-regulated	protein_coding
MARCH5	ENSMMUG00000004816	41.0488851	-2.465429709	0.73672	-3.34649	0.0008184	0.002859161	down-regulated	protein_coding
KLHDC8B	ENSMMUG00000002833	15.78023629	-4.347943014	1.299708	-3.34532	0.0008219	0.002869836	down-regulated	protein_coding
ADCY3	ENSMMUG00000009600	74.82377777	1.924102731	0.575206	3.34507	0.0008226	0.002871756	up-regulated	protein_coding
NAALAD2	ENSMMUG00000005207	11.09339931	4.788670496	1.431911	3.344252	0.000825	0.002879527	up-regulated	protein_coding
DFFA	ENSMMUG00000032254	43.72752256	-2.46195141	0.736267	-3.34383	0.0008263	0.002883219	down-regulated	protein_coding
FBXL5	ENSMMUG00000009456	55.47392578	-2.222726456	0.664884	-3.34303	0.0008287	0.00289084	down-regulated	protein_coding
VWA5A	ENSMMUG00000020491	145.3745248	-2.070508816	0.619532	-3.34205	0.0008316	0.002899579	down-regulated	protein_coding
TSC22D1	ENSMMUG00000003753	187.0314057	-1.658287255	0.496341	-3.34102	0.0008347	0.002909458	down-regulated	protein_coding
OR2AE1	ENSMMUG00000022501	6.477224086	6.6239497	1.982642	3.340972	0.0008349	0.002909458	up-regulated	protein_coding
ZBTB44	ENSMMUG00000022944	36.48874887	2.1013583	0.629031	3.340625	0.0008359	0.002911653	up-regulated	protein_coding
NDRG1	ENSMMUG00000010200	2694.166065	-2.485477222	0.74415	-3.34002	0.0008377	0.002917251	down-regulated	protein_coding
RIOK2	ENSMMUG00000000307	15.93730237	-4.36156983	1.305975	-3.3397	0.0008387	0.002919905	down-regulated	protein_coding
IFRD1	ENSMMUG00000008033	61.95199609	-2.793024299	0.836571	-3.33866	0.0008418	0.002930209	down-regulated	protein_coding
NARG2	ENSMMUG00000021320	14.78733644	-4.256147653	1.275162	-3.33773	0.0008447	0.00293926	down-regulated	protein_coding
THRSP	ENSMMUG00000017470	6.374236166	4.556911317	1.365475	3.337236	0.0008462	0.002943775	up-regulated	protein_coding
FBXL15	ENSMMUG00000002996	125.6753065	1.712679126	0.513254	3.336906	0.0008472	0.002946542	up-regulated	protein_coding
GABARAPL2	ENSMMUG00000005759	113.0962826	-1.626057284	0.487332	-3.33665	0.0008479	0.002948528	down-regulated	protein_coding
EBPL	ENSMMUG00000020571	19.11392082	-4.623930404	1.386147	-3.33582	0.0008505	0.002956677	down-regulated	protein_coding
ATL2	ENSMMUG00000008463	63.60142498	-2.236077638	0.670368	-3.3356	0.0008512	0.002957754	down-regulated	protein_coding
COPS2	ENSMMUG00000009838	47.49326587	-2.071422563	0.621009	-3.33558	0.0008512	0.002957754	down-regulated	protein_coding
TMEM183A	ENSMMUG00000000801	95.47866699	-1.670962228	0.500993	-3.3353	0.0008521	0.002959967	down-regulated	protein_coding
MEP1B	ENSMMUG00000023019	165.6244673	-3.032767502	0.909433	-3.33479	0.0008536	0.002964708	down-regulated	protein_coding
WBSCR16	ENSMMUG00000001003	28.47290622	-4.194455704	1.258195	-3.33371	0.000857	0.00297476	down-regulated	protein_coding
BTNL8	ENSMMUG00000019858	99.77752712	-2.91518367	0.874457	-3.33371	0.000857	0.00297476	down-regulated	protein_coding
WRB	ENSMMUG00000020654	39.87783532	-2.759248037	0.827698	-3.33364	0.0008572	0.00297476	down-regulated	protein_coding
RNF219	ENSMMUG00000011697	14.9481647	-4.271340141	1.282189	-3.33129	0.0008645	0.002999313	down-regulated	protein_coding
C15H9ORF72	ENSMMUG00000016508	15.65347608	-4.338832645	1.302576	-3.33096	0.0008655	0.00300119	down-regulated	protein_coding
PPP1R21	ENSMMUG00000022853	37.19767715	-2.656785266	0.797616	-3.33091	0.0008656	0.00300119	down-regulated	protein_coding
7SK	ENSMMUG00000034173	7.668956291	4.367729398	1.311265	3.330928	0.0008656	0.00300119	up-regulated	misc_RNA
PRSS37	ENSMMUG00000011844	12.076627	6.396162847	1.921119	3.329395	0.0008703	0.003015318	up-regulated	protein_coding
PDZD11	ENSMMUG00000013938	41.68276799	-3.305882501	0.993316	-3.32813	0.0008743	0.003028343	down-regulated	protein_coding
KLF6	ENSMMUG00000010696	161.7178628	-1.96586673	0.590998	-3.32635	0.0008799	0.003046208	down-regulated	protein_coding
MRPL39	ENSMMUG00000017605	31.46223977	-4.342472093	1.305741	-3.32568	0.000882	0.003052848	down-regulated	protein_coding
SDF4	ENSMMUG00000019518	542.0692639	-1.185755427	0.356573	-3.32542	0.0008828	0.003054136	down-regulated	protein_coding
LPAR6	ENSMMUG00000007894	28.15058159	2.347767045	0.706048	3.325224	0.0008835	0.003055557	up-regulated	protein_coding
CDR2	ENSMMUG00000022035	27.0878982	-4.121989294	1.24033	-3.3233	0.0008896	0.003075962	down-regulated	protein_coding
AP3M1	ENSMMUG00000020825	131.0060458	-1.382589941	0.416127	-3.32252	0.0008921	0.003083824	down-regulated	protein_coding
IGSF5	ENSMMUG00000003427	7.512286112	4.779682258	1.438793	3.322009	0.0008937	0.003088083	up-regulated	protein_coding
GPBP1	ENSMMUG00000014647	121.764148	-1.548988911	0.466282	-3.322	0.0008938	0.003088083	down-regulated	protein_coding
RSL1D1	ENSMMUG00000019632	280.9068197	1.063049536	0.320125	3.320734	0.0008978	0.003101346	up-regulated	protein_coding
KDELR3	ENSMMUG00000004303	85.18311255	1.259148722	0.379361	3.319127	0.000903	0.003116964	up-regulated	protein_coding
ARF5	ENSMMUG00000018617	254.3545723	-1.182681389	0.356317	-3.31919	0.0009028	0.003116964	down-regulated	protein_coding
TUBB2B	ENSMMUG00000030028	231.4616232	-1.373797548	0.414188	-3.31685	0.0009104	0.003141736	down-regulated	protein_coding
CCDC56	ENSMMUG00000012637	91.9343231	-2.618825397	0.789711	-3.31618	0.0009126	0.003148468	down-regulated	protein_coding
AIMP1	ENSMMUG00000015759	50.51970993	-2.68335252	0.809222	-3.31597	0.0009133	0.003150126	down-regulated	protein_coding
NAPSA	ENSMMUG00000013402	10.97991188	3.49678219	1.05596	3.311473	0.0009281	0.003198821	up-regulated	protein_coding
LMO1	ENSMMUG00000015017	13.90566975	6.407963056	1.935451	3.310836	0.0009302	0.003205323	up-regulated	protein_coding
SLC30A1	ENSMMUG00000000868	144.5730187	1.51033393	0.45638	3.30938	0.000935	0.003221265	up-regulated	protein_coding
LNP1	ENSMMUG00000032088	15.7903273	3.714347889	1.123127	3.30715	0.0009425	0.003245438	up-regulated	protein_coding
DHX35	ENSMMUG00000003385	14.81647354	-4.256800095	1.287217	-3.30698	0.0009431	0.003246616	down-regulated	protein_coding
PHF6	ENSMMUG00000007997	36.06610703	-2.993549841	0.905668	-3.30535	0.0009486	0.003264772	down-regulated	protein_coding
PLXNA4	ENSMMUG00000005127	7.13657895	6.093208141	1.843903	3.304517	0.0009514	0.003273685	up-regulated	protein_coding
PIH1D2	ENSMMUG00000019419	6.986616354	3.716586358	1.124813	3.304182	0.0009525	0.003276807	up-regulated	protein_coding
Y_RNA	ENSMMUG00000024526	7.728721363	6.442571554	1.950231	3.303492	0.0009549	0.003284077	up-regulated	misc_RNA
COQ10A	ENSMMUG00000031834	34.18382449	2.239979389	0.678231	3.302679	0.0009577	0.003292818	up-regulated	protein_coding
PCID2	ENSMMUG00000001041	28.35827468	-4.18456067	1.26724	-3.30211	0.0009596	0.00329713	down-regulated	protein_coding
MTX2	ENSMMUG00000010185	35.40290645	-3.036406213	0.919531	-3.30213	0.0009596	0.00329713	down-regulated	protein_coding
RIN3	ENSMMUG00000029233	70.79494104	3.173659383	0.961086	3.302158	0.0009594	0.00329713	up-regulated	protein_coding
U6	ENSMMUG00000036036	5.75004856	4.237209869	1.283311	3.30178	0.0009607	0.003300181	up-regulated	snRNA
TSC22D3	ENSMMUG00000008207	28.24779357	-4.183743793	1.267244	-3.30145	0.0009619	0.003303254	down-regulated	protein_coding
CDPF1	ENSMMUG00000016259	13.32786082	3.3489177	1.014755	3.300223	0.0009661	0.003316935	up-regulated	protein_coding
ANP32E	ENSMMUG00000021531	60.38612711	-1.939996458	0.58792	-3.29976	0.0009677	0.003321561	down-regulated	protein_coding
IFFO1	ENSMMUG00000018681	12.15159097	3.060265685	0.927621	3.299046	0.0009701	0.003329249	up-regulated	protein_coding
MRPS7	ENSMMUG00000018022	157.2821708	1.608146543	0.4875	3.298759	0.0009711	0.003331849	up-regulated	protein_coding
CUL4A	ENSMMUG00000019297	107.7467184	-1.265162924	0.383652	-3.29768	0.0009749	0.003342572	down-regulated	protein_coding
FAM129A	ENSMMUG00000019515	16.69228187	2.208498743	0.669718	3.297653	0.000975	0.003342572	up-regulated	protein_coding
PSENEN	ENSMMUG00000002386	72.7503876	-2.350114034	0.713417	-3.29417	0.0009871	0.003383478	down-regulated	protein_coding
SHC2	ENSMMUG00000000485	192.4373752	-2.292534804	0.69602	-3.29378	0.0009885	0.00338735	down-regulated	protein_coding
PAR14	ENSMMUG00000012956	16.97395616	-4.452315084	1.351774	-3.29368	0.0009888	0.003387681	down-regulated	protein_coding
MCIDAS	ENSMMUG00000030102	13.76765044	3.507772782	1.065193	3.293087	0.0009909	0.003394038	up-regulated	protein_coding
TSC1	ENSMMUG00000009918	62.76683654	1.316804018	0.399912	3.292735	0.0009922	0.003397475	up-regulated	protein_coding
TRIM8	ENSMMUG00000004394	285.3158655	1.038642937	0.315471	3.292358	0.0009935	0.003401206	up-regulated	protein_coding
ABHD17C	ENSMMUG00000012347	77.46253958	-1.580863228	0.480189	-3.29217	0.0009942	0.003402649	down-regulated	protein_coding
HNRNPH1	ENSMMUG00000010843	294.1177128	-2.096586902	0.637031	-3.29118	0.0009977	0.003413798	down-regulated	protein_coding
CHCHD1	ENSMMUG00000004608	43.24971484	-3.883940174	1.18022	-3.29086	0.0009988	0.003416863	down-regulated	protein_coding
PGAM1	ENSMMUG00000020027	657.697494	-1.409183865	0.428295	-3.29021	0.0010011	0.003423914	down-regulated	protein_coding
SCRN2	ENSMMUG00000003012	65.08137018	-2.109444578	0.641154	-3.29008	0.0010016	0.003423927	down-regulated	protein_coding
PXDC1	ENSMMUG00000010926	16.56632734	-4.42091537	1.343688	-3.29014	0.0010014	0.003423927	down-regulated	protein_coding
RASGRP4	ENSMMUG00000020469	9.752098852	3.864466679	1.17483	3.289384	0.0010041	0.003431538	up-regulated	protein_coding
GOLPH3L	ENSMMUG00000010668	36.76707094	-3.647065611	1.109178	-3.28808	0.0010087	0.003446629	down-regulated	protein_coding

ARMC5	ENSMMUG00000000895	55.84743177	1.757270233	0.534528	3.287518	0.0010107	0.003452696	up-regulated	protein_coding
SWAP70	ENSMMUG00000009204	155.1818226	-2.298596223	0.699236	-3.2873	0.0010115	0.00345456	down-regulated	protein_coding
PAQR7	ENSMMUG00000007857	29.49188807	-5.251978031	1.597856	-3.28689	0.001013	0.003458721	down-regulated	protein_coding
EMG1	ENSMMUG00000010207	112.8113591	1.546996069	0.470697	3.286607	0.001014	0.00346138	up-regulated	protein_coding
MRPL24	ENSMMUG00000000039	110.9714505	-1.94893513	0.593055	-3.28626	0.0010153	0.003463104	down-regulated	protein_coding
ELL2	ENSMMUG00000005314	75.57379243	-2.164282751	0.658581	-3.28628	0.0010152	0.003463104	down-regulated	protein_coding
KRT23	ENSMMUG00000001505	276.6951844	1.603214365	0.487909	3.285888	0.0010166	0.003466881	up-regulated	protein_coding
PEX19	ENSMMUG00000021852	122.9964847	-1.118281504	0.340359	-3.28559	0.0010177	0.003469683	down-regulated	protein_coding
TRAF7	ENSMMUG00000019223	317.627703	-1.135823241	0.345728	-3.28531	0.0010187	0.003472358	down-regulated	protein_coding
ICMT	ENSMMUG00000018350	91.90301161	-1.34737008	0.410194	-3.28471	0.0010209	0.003478841	down-regulated	protein_coding
PAF1	ENSMMUG00000005552	81.68741866	1.255741334	0.382326	3.284474	0.0010217	0.003480888	up-regulated	protein_coding
ACCN4	ENSMMUG00000013244	6.357230676	6.310089311	1.921263	3.284345	0.0010222	0.003480888	up-regulated	protein_coding
RSPH4A	ENSMMUG00000001805	5.574219864	4.256265689	1.296351	3.283266	0.0010261	0.003493399	up-regulated	protein_coding
MED30	ENSMMUG00000002068	16.56549556	-4.417878927	1.345858	-3.28258	0.0010286	0.003501123	down-regulated	protein_coding
PGM1	ENSMMUG00000020049	201.9413244	-1.540983837	0.469502	-3.28217	0.0010301	0.003505359	down-regulated	protein_coding
LAMTOR2	ENSMMUG00000012126	43.31712172	-3.347548637	1.020327	-3.28086	0.0010349	0.003520786	down-regulated	protein_coding
GNPTAB	ENSMMUG00000012562	73.81579485	2.486510573	0.758001	3.280352	0.0010368	0.003526292	up-regulated	protein_coding
AEBP1	ENSMMUG00000002422	9.236250449	5.220244313	1.591419	3.280246	0.0010372	0.003526763	up-regulated	protein_coding
CNNM2	ENSMMUG00000003747	78.80200009	1.80296709	0.549724	3.27977	0.0010389	0.003531865	up-regulated	protein_coding
RRP9	ENSMMUG00000003644	19.55653444	-4.65682694	1.419999	-3.27946	0.0010401	0.00353408	down-regulated	protein_coding
SNED1	ENSMMUG00000004968	23.07030807	4.141014155	1.263124	3.27839	0.001044	0.003546623	up-regulated	protein_coding
ACTR10	ENSMMUG00000003508	32.70749686	-3.508509575	1.070236	-3.27826	0.0010445	0.003547437	down-regulated	protein_coding
PIGY	ENSMMUG00000010318	87.44589507	-1.747816245	0.53334	-3.27712	0.0010487	0.003560965	down-regulated	protein_coding
PAFAH1B1	ENSMMUG00000020524	251.5458239	-1.043525298	0.318553	-3.27583	0.0010535	0.003575543	down-regulated	protein_coding
PRDX4	ENSMMUG00000021366	41.2737475	-2.826638378	0.862867	-3.27587	0.0010534	0.003575543	down-regulated	protein_coding
CETN3	ENSMMUG00000020180	16.32213376	-4.396524621	1.342642	-3.27453	0.0010584	0.003591085	down-regulated	protein_coding
TIAM1	ENSMMUG00000019571	13.41400757	2.604620461	0.795468	3.274326	0.0010591	0.003592857	up-regulated	protein_coding
NMRK1	ENSMMUG00000010400	17.50513984	-4.496792073	1.374359	-3.27192	0.0010682	0.003622704	down-regulated	protein_coding
ANKS1B	ENSMMUG00000022406	10.81599375	6.583752866	2.0123	3.271755	0.0010688	0.003623952	up-regulated	protein_coding
HPS6	ENSMMUG00000009374	53.35305962	-2.448104663	0.748517	-3.27061	0.0010732	0.003636955	down-regulated	protein_coding
ZCCHC6	ENSMMUG00000003401	94.33146548	-1.587362766	0.4859	-3.26685	0.0010875	0.003684662	down-regulated	protein_coding
NUP54	ENSMMUG00000020910	27.96522936	-4.167785642	1.276224	-3.26572	0.0010919	0.003697972	down-regulated	protein_coding
MACC1	ENSMMUG00000022683	73.36038486	-3.100706117	0.94952	-3.26555	0.0010925	0.003698981	down-regulated	protein_coding
TERF2IP	ENSMMUG00000005756	49.35393848	-2.121293026	0.649785	-3.26461	0.0010962	0.003710396	down-regulated	protein_coding
TNFRSF12A	ENSMMUG00000007663	290.098001	-1.144362656	0.350607	-3.26395	0.0010987	0.00371817	down-regulated	protein_coding
CNBP	ENSMMUG00000011585	345.2313767	-1.804093747	0.553026	-3.26222	0.0011054	0.003739584	down-regulated	protein_coding
SLC25A27	ENSMMUG00000019250	15.64494957	-4.33576144	1.329097	-3.26219	0.0011056	0.003739584	down-regulated	protein_coding
UBE2J2	ENSMMUG00000010030	48.64295022	-2.34914435	0.720144	-3.26205	0.0011061	0.003740491	down-regulated	protein_coding
CAST	ENSMMUG00000002062	640.5864753	-1.050147698	0.32198	-3.26153	0.0011081	0.003746436	down-regulated	protein_coding
MRPS14	ENSMMUG00000001573	44.13770374	-3.238211171	0.992991	-3.26107	0.0011099	0.003751226	down-regulated	protein_coding
5S_rRNA	ENSMMUG00000023874	6.07266334	4.053079426	1.242882	3.261033	0.0011101	0.003751226	up-regulated	rRNA
BMP6	ENSMMUG00000021600	5.736905366	4.676694584	1.43415	3.260952	0.0011104	0.003751405	up-regulated	protein_coding
MSRA	ENSMMUG00000009382	51.11319588	-2.807973	0.861372	-3.25988	0.0011146	0.003764667	down-regulated	protein_coding
RNF217	ENSMMUG00000003134	42.41960384	1.794834766	0.551378	3.255179	0.0011332	0.003825765	up-regulated	protein_coding
VPS36	ENSMMUG00000011360	175.0607548	-1.633934353	0.502006	-3.25481	0.0011347	0.003828948	down-regulated	protein_coding
CR1	ENSMMUG00000013596	5.590051293	3.371503309	1.035833	3.254873	0.0011344	0.003828948	up-regulated	protein_coding
TRPC3	ENSMMUG00000009820	8.388114306	5.540485455	1.702586	3.254159	0.0011373	0.003836781	up-regulated	protein_coding
ESYT3	ENSMMUG00000007757	37.18418129	1.612315201	0.495604	3.253231	0.001141	0.003848411	up-regulated	protein_coding
KAT5	ENSMMUG00000019839	104.4175996	1.521355994	0.467696	3.252876	0.0011424	0.00385231	up-regulated	protein_coding
MBOAT1	ENSMMUG00000017425	14.66956702	-4.243178732	1.304703	-3.25222	0.0011451	0.003859408	down-regulated	protein_coding
ACTL10	ENSMMUG00000023549	10.12043626	7.362946712	2.263936	3.252277	0.0011448	0.003859408	up-regulated	protein_coding
MARCH8	ENSMMUG00000013437	74.8247742	-2.350502989	0.722806	-3.25192	0.0011463	0.003862594	down-regulated	protein_coding
LRRC23	ENSMMUG00000001781	5.446498098	5.193026963	1.597416	3.250893	0.0011504	0.003874663	up-regulated	protein_coding
ZNF837	ENSMMUG00000009387	13.90675082	3.116026564	0.958496	3.250953	0.0011502	0.003874663	up-regulated	protein_coding
DNAJC2	ENSMMUG00000003430	88.81909951	1.521322057	0.468169	3.249514	0.001156	0.003891643	up-regulated	protein_coding
PLK2	ENSMMUG00000005177	38.86917133	-2.711720642	0.834494	-3.24954	0.0011559	0.003891643	down-regulated	protein_coding
DPCR1	ENSMMUG00000018615	5.400513503	4.554836774	1.402021	3.248765	0.0011591	0.003900054	up-regulated	protein_coding
PDCD10	ENSMMUG00000005938	116.3957973	-1.204461557	0.37078	-3.24845	0.0011603	0.003903405	down-regulated	protein_coding
DCAF10	ENSMMUG00000008667	130.9887608	1.482860319	0.456535	3.248078	0.0011619	0.003907627	up-regulated	protein_coding
EAPP	ENSMMUG00000008824	41.97666624	-2.877497442	0.88593	-3.248	0.0011622	0.003907794	down-regulated	protein_coding
RPL12	ENSMMUG00000000422	839.6540552	-1.877697882	0.578398	-3.24637	0.0011689	0.003929217	down-regulated	protein_coding
GJB3	ENSMMUG00000022677	91.11485938	-2.516881901	0.775362	-3.24607	0.0011701	0.003932436	down-regulated	protein_coding
MAEA	ENSMMUG00000008357	92.71589184	-1.612296622	0.496722	-3.24587	0.0011709	0.003934271	down-regulated	protein_coding
DTX1	ENSMMUG00000021791	20.92763021	5.174821406	1.594363	3.245698	0.0011716	0.003935758	up-regulated	protein_coding
ALDH9A1	ENSMMUG00000015946	85.28131453	-2.39816924	0.738916	-3.24553	0.0011723	0.00393721	down-regulated	protein_coding
CAPN12	ENSMMUG00000006431	46.86446521	2.50809905	0.772834	3.245325	0.0011732	0.003939038	up-regulated	protein_coding
ZMYM2	ENSMMUG00000030812	81.1954462	-1.625874353	0.501062	-3.24486	0.0011751	0.003944583	down-regulated	protein_coding
TEX35	ENSMMUG00000021077	11.12123053	6.445632792	1.98664	3.24449	0.0011766	0.003948739	up-regulated	protein_coding
ABRA	ENSMMUG00000017850	12.38036698	6.50925315	2.006798	3.243602	0.0011803	0.003960131	up-regulated	protein_coding
AIMP2	ENSMMUG00000030420	126.6937934	1.714749374	0.528862	3.242337	0.0011855	0.003976807	up-regulated	protein_coding
FOXK1	ENSMMUG00000011227	52.89844355	1.598265074	0.493087	3.241348	0.0011897	0.003989583	up-regulated	protein_coding
UQCRQ	ENSMMUG00000015635	228.5403653	-3.317255575	1.023444	-3.24127	0.00119	0.003989583	down-regulated	protein_coding
OARD1	ENSMMUG00000021172	55.16286807	-1.94434261	0.59988	-3.24122	0.0011902	0.003989583	down-regulated	protein_coding
PTRF	ENSMMUG00000029994	46.26319627	2.408958759	0.743597	3.239602	0.001197	0.004011343	up-regulated	protein_coding
COQ10B	ENSMMUG00000010488	17.16125109	-4.471643488	1.380725	-3.23862	0.0012011	0.004022344	down-regulated	protein_coding
ABCG1	ENSMMUG00000018058	16.97857546	-4.455881177	1.375813	-3.23873	0.0012007	0.004022344	down-regulated	protein_coding
RNF214	ENSMMUG00000002067	53.67475285	1.895513803	0.585614	3.236798	0.0012088	0.004047131	up-regulated	protein_coding
ZSWIM7	ENSMMUG00000030294	40.23154394	2.55409642	0.789165	3.236454	0.0012102	0.004051055	up-regulated	protein_coding
GLA	ENSMMUG00000019577	37.7404008	-3.755997293	1.160921	-3.23536	0.0012149	0.004064682	down-regulated	protein_coding
SHARPIN	ENSMMUG00000007152	164.6527209	-1.528631903	0.472638	-3.23426	0.0012196	0.004079443	down-regulated	protein_coding
LEMD2	ENSMMUG00000021734	98.32785535	-1.391663001	0.430411	-3.23333	0.0012235	0.004091682	down-regulated	protein_coding
VPS25	ENSMMUG00000002149	96.83666024	-1.747558531	0.540557	-3.23288	0.0012255	0.004095257	down-regulated	protein_coding
BTRC	ENSMMUG00000006741	48.28566245	1.426168824	0.441141	3.232913	0.0012253	0.004095257	up-regulated	protein_coding
MIOX	ENSMMUG00000009088	18.40740471	2.223801888	0.687851	3.232971	0.0012251	0.004095257	up-regulated	protein_coding
GOT1	ENSMMUG00000010356	71.71242912	-1.604859308	0.496573	-3.23187	0.0012298	0.004108854	down-regulated	protein_coding
CCBL1	ENSMMUG00000021848	55.02583078	-1.892951206	0.585784	-3.23148	0.0012315	0.00411342	down-regulated	protein_coding
DYM	ENSMMUG00000001760	32.39131814	-3.50374813	1.084351	-3.23119	0.0012327	0.004115658	down-regulated	protein_coding
PPP2R4	ENSMMUG00000002515	88.06787407	-1.309366668	0.405222	-3.23123	0.0012326	0.004115658	down-regulated	protein_coding
TMEM64	ENSMMUG00000004011	13.94925416	-4.172540477	1.291496	-3.23078	0.0012345	0.00412061	down-regulated	protein_coding
NIT1	ENSMMUG00000002009	98.43240402	-1.637343357	0.507116	-3.22874	0.0012434	0.004149198	down-regulated	protein_coding
HAUS2	ENSMMUG00000001548	13.91644939	-4.165993503	1.290508	-3.22818	0.0012458	0.004156299	down-regulated	protein_coding
MAPK8IP1	ENSMMUG00000002456	91.90404438	1.035716977	0.320991	3.226618	0.0012526	0.004178071	up-regulated	protein_coding
PPM1M	ENSMMUG00000008236	17.3132549	2.772270281	0.859288	3.226239	0.0012543	0.004182623	up-regulated	protein_coding
QDPR	ENSMMUG00000005730	46.98662393	-2.832883808	0.878104	-3.22614	0.0012547	0.004183143	down-regulated	protein_coding
WDR46	ENSMMUG00000003869	146.9708015	1.753639393	0.543661	3.225614	0.001257	0.004189794	up-regulated	protein_coding
PLK1	ENSMMUG00000017306	276.6913303	2.466818201	0.764812	3.225392	0.001258	0.004192058	up-regulated	protein_coding
GNB3	ENSMMUG00000005190	19.34523657	4.534365348	1.406864	3.22303	0.0012684	0.004223801	up-regulated	protein_coding
AFF1	ENSMMUG00000014076	67.19199757	1.93211369	0.599593	3.222378	0.0012713	0.004232429	up-regulated	protein_coding
VBP1	ENSMMUG00000015095	27.61254439	-4.110925578	1.275973	-3.2218	0.0012739	0.004239795	down-regulated	protein_coding
IMMP2L	ENSMMUG00000015535	13.8209165	-4.157010239	1.290298	-3.22174	0.0012741	0.004239795	down-regulated	protein_coding

ADAM28	ENSMMUG00000005317	7.669512065	4.776183146	1.482621	3.221446	0.0012755	0.004242682	up-regulated	protein_coding
FAM213B	ENSMMUG00000012380	15.45524258	-4.317963652	1.340394	-3.22141	0.0012756	0.004242682	down-regulated	protein_coding
VWA1	ENSMMUG00000013280	51.05617418	-2.305885231	0.715996	-3.22053	0.0012795	0.004254827	down-regulated	protein_coding
CRYAB	ENSMMUG00000022133	10.75092645	3.240919841	1.006577	3.219745	0.001283	0.004264467	up-regulated	protein_coding
SCAMP4	ENSMMUG00000013747	33.03008056	-2.917225565	0.906132	-3.21943	0.0012845	0.004268181	down-regulated	protein_coding
SLC41A2	ENSMMUG00000017129	16.60358239	-4.424071703	1.37455	-3.21856	0.0012884	0.004279104	down-regulated	protein_coding
IFRD2	ENSMMUG00000020119	85.08598317	-2.147374412	0.667178	-3.21859	0.0012882	0.004279104	down-regulated	protein_coding
MT-CYB	ENSMMUG00000028670	52865.16519	8.826916384	2.742941	3.218048	0.0012907	0.004285744	up-regulated	protein_coding
TIPRL	ENSMMUG00000021166	25.36057857	-3.981395773	1.237349	-3.21768	0.0012923	0.004290211	down-regulated	protein_coding
TBC1D22A	ENSMMUG00000003764	53.43261794	-1.894051864	0.588687	-3.21742	0.0012935	0.004293145	down-regulated	protein_coding
RCC1	ENSMMUG00000018338	18.6112646	-4.585380786	1.425365	-3.21699	0.0012954	0.004296594	down-regulated	protein_coding
C20orf27	ENSMMUG00000022079	67.53827843	-2.03330289	0.632052	-3.21699	0.0012954	0.004296594	down-regulated	protein_coding
ANKRD13D	ENSMMUG00000015928	74.03630777	1.69797366	0.527842	3.216823	0.0012962	0.004298042	up-regulated	protein_coding
ADGRL2	ENSMMUG00000002516	93.72192181	-1.912268088	0.595077	-3.21348	0.0013114	0.004347376	down-regulated	protein_coding
NUDCD2	ENSMMUG00000019552	17.11455306	-4.464208069	1.389621	-3.21254	0.0013157	0.00436065	down-regulated	protein_coding
ACOX2	ENSMMUG00000006229	42.48702408	-2.829014902	0.880746	-3.21207	0.0013178	0.00436573	down-regulated	protein_coding
WRNIP1	ENSMMUG00000007143	96.47418113	-1.549305538	0.482333	-3.21211	0.0013176	0.00436573	down-regulated	protein_coding
PPHLN1	ENSMMUG00000021735	35.03213026	-2.896010443	0.901935	-3.21089	0.0013233	0.0043827	down-regulated	protein_coding
COG5	ENSMMUG00000015411	83.9203648	1.509430646	0.470155	3.210494	0.0013251	0.004387649	up-regulated	protein_coding
FBN3	ENSMMUG00000010003	26.57999094	3.024030256	0.941963	3.210351	0.0013257	0.004388805	up-regulated	protein_coding
GALNT7	ENSMMUG00000013106	178.1236337	-1.44633202	0.450581	-3.20993	0.0013277	0.004394216	down-regulated	protein_coding
XPO5	ENSMMUG00000015193	129.4712071	1.233748565	0.38443	3.209295	0.0013306	0.004402894	up-regulated	protein_coding
SCFD1	ENSMMUG00000019692	40.62626443	-2.677057792	0.834223	-3.20904	0.0013318	0.004403659	down-regulated	protein_coding
SEL1L3	ENSMMUG00000020349	273.4906484	-1.122772526	0.349873	-3.20908	0.0013316	0.004403659	down-regulated	protein_coding
DNTT	ENSMMUG00000022496	10.27213273	3.536622334	1.102059	3.209104	0.0013315	0.004403659	up-regulated	protein_coding
AIG1	ENSMMUG00000006430	69.82025474	-1.839430621	0.573436	-3.20773	0.0013378	0.004422713	down-regulated	protein_coding
KCNB2	ENSMMUG00000013770	7.355530009	7.404302778	2.308777	3.207024	0.0013412	0.004432625	up-regulated	protein_coding
MTMR10	ENSMMUG00000009428	60.50680686	2.039112225	0.635867	3.206823	0.0013421	0.004434674	up-regulated	protein_coding
PIGG	ENSMMUG00000017457	46.32798474	-1.986221731	0.619606	-3.20562	0.0013477	0.004451194	down-regulated	protein_coding
EIF2A	ENSMMUG00000017696	76.000584	-1.697153315	0.529454	-3.20548	0.0013484	0.004452316	down-regulated	protein_coding
HMGB3	ENSMMUG00000004633	47.74914473	-3.061144639	0.955038	-3.20526	0.0013494	0.00445466	down-regulated	protein_coding
SLC25A43	ENSMMUG00000020282	18.83943884	2.296482973	0.716532	3.204995	0.0013506	0.004457732	up-regulated	protein_coding
TMEM63B	ENSMMUG00000016230	189.2600772	-1.433354132	0.447312	-3.20437	0.0013536	0.004465301	down-regulated	protein_coding
FAM50A	ENSMMUG00000005505	49.83162875	-2.912088572	0.909005	-3.2036	0.0013572	0.004474161	down-regulated	protein_coding
KRT20	ENSMMUG00000001503	2258.342736	-1.659240177	0.518141	-3.20229	0.0013634	0.004493448	down-regulated	protein_coding
SH3GLB2	ENSMMUG00000011759	325.3942156	-1.447692393	0.452136	-3.2019	0.0013653	0.004498633	down-regulated	protein_coding
B4GALT2	ENSMMUG00000015155	27.34683059	-4.096361582	1.279677	-3.20109	0.0013691	0.004509115	down-regulated	protein_coding
ZFP41	ENSMMUG00000018500	71.50169451	1.372881369	0.428907	3.200885	0.0013701	0.004511294	up-regulated	protein_coding
Y_RNA	ENSMMUG00000028414	7.21388039	5.078436316	1.586662	3.200705	0.0013709	0.004513064	up-regulated	misc_RNA
SCML1	ENSMMUG00000012899	15.89328341	-4.358959644	1.362126	-3.20011	0.0013737	0.004521258	down-regulated	protein_coding
EXOSC9	ENSMMUG00000009816	62.98244249	1.991612254	0.622577	3.19898	0.0013791	0.004538043	up-regulated	protein_coding
LEF1	ENSMMUG00000023256	59.34198305	2.106091459	0.658586	3.197898	0.0013843	0.004554046	up-regulated	protein_coding
ANKRD49	ENSMMUG00000004119	13.37688248	-4.110956379	1.285868	-3.19703	0.0013885	0.00456672	down-regulated	protein_coding
ABHD6	ENSMMUG00000006315	103.9564264	-2.361247701	0.738673	-3.19661	0.0013905	0.004572189	down-regulated	protein_coding
HERC3	ENSMMUG00000010138	102.7333704	-2.35253042	0.735975	-3.19648	0.0013911	0.004572189	down-regulated	protein_coding
PHAX	ENSMMUG00000016683	24.59591466	-3.977854124	1.24443	-3.19653	0.0013909	0.004572189	down-regulated	protein_coding
ADSSL1	ENSMMUG00000016556	49.42368993	-2.406646019	0.753028	-3.19596	0.0013937	0.004579427	down-regulated	protein_coding
GMPS	ENSMMUG00000007324	57.3869773	-2.042244397	0.639104	-3.19548	0.001396	0.004584906	down-regulated	protein_coding
KCNJ12	ENSMMUG00000010903	10.05331641	4.3061747	1.347572	3.195507	0.0013959	0.004584906	up-regulated	protein_coding
RNF145	ENSMMUG00000021699	401.8138281	-1.270905285	0.397793	-3.19489	0.0013988	0.004593187	down-regulated	protein_coding
HGD	ENSMMUG00000001593	12.4434065	4.106570703	1.285555	3.194395	0.0014012	0.004600048	up-regulated	protein_coding
NKTR	ENSMMUG00000021242	60.13909455	-1.734552686	0.543556	-3.19112	0.0014172	0.004650374	down-regulated	protein_coding
TYRO3	ENSMMUG00000013803	8.689723206	3.212784578	1.007043	3.190316	0.0014212	0.004662231	up-regulated	protein_coding
MRPS23	ENSMMUG00000001913	30.44650339	-4.293762545	1.346179	-3.18959	0.0014247	0.004672832	down-regulated	protein_coding
C13H2ORF78	ENSMMUG00000010863	7.224907254	6.012759999	1.887093	3.186256	0.0014413	0.00472268	up-regulated	protein_coding
SLC13A1	ENSMMUG00000016770	8.129435891	5.596116631	1.756484	3.185977	0.0014427	0.00472545	up-regulated	protein_coding
ARL5A	ENSMMUG00000010521	39.29681603	-3.221257282	1.011215	-3.18553	0.0014449	0.00473121	down-regulated	protein_coding
TMEM25	ENSMMUG00000005492	24.72482349	-3.984324191	1.250963	-3.18501	0.0014475	0.004738752	down-regulated	protein_coding
RAP2B	ENSMMUG00000015779	35.06906019	-3.030773892	0.951671	-3.18469	0.0014491	0.004742873	down-regulated	protein_coding
ATP8	ENSMMUG00000028684	51.15410201	11.57383666	3.636753	3.182464	0.0014603	0.004777208	up-regulated	protein_coding
RBBP8	ENSMMUG00000012715	26.64210822	-4.098160596	1.288626	-3.18026	0.0014714	0.004812638	down-regulated	protein_coding
ERCC1	ENSMMUG00000000718	36.29683688	-3.671268324	1.154727	-3.17934	0.0014761	0.004826801	down-regulated	protein_coding
TOB2	ENSMMUG00000002554	53.62061103	-2.495118716	0.785098	-3.1781	0.0014824	0.004844151	down-regulated	protein_coding
SLC2A7	ENSMMUG00000019020	36.41904801	-4.532644547	1.426668	-3.17708	0.0014876	0.004859989	down-regulated	protein_coding
TRAIP	ENSMMUG00000016476	39.15108124	2.783165033	0.876055	3.176928	0.0014884	0.004861481	up-regulated	protein_coding
ANK2	ENSMMUG00000008633	8.685553206	6.114902588	1.925716	3.175392	0.0014963	0.004886159	up-regulated	protein_coding
OXSM	ENSMMUG00000014798	14.06312536	-4.180782289	1.316873	-3.17478	0.0014995	0.004895335	down-regulated	protein_coding
SH3KBP1	ENSMMUG00000004744	74.16541877	-1.823712315	0.574612	-3.17382	0.0015045	0.004910421	down-regulated	protein_coding
PLD1	ENSMMUG00000023289	141.9625885	-1.860204224	0.586121	-3.17375	0.0015048	0.004910421	down-regulated	protein_coding
GORASP2	ENSMMUG00000015203	128.2572525	-1.122734441	0.353786	-3.17349	0.0015062	0.004913838	down-regulated	protein_coding
ARPC1A	ENSMMUG00000019342	200.1647454	-1.593539267	0.502277	-3.17263	0.0015107	0.004927235	down-regulated	protein_coding
C4ORF14	ENSMMUG00000022530	56.86192639	-2.292821338	0.722706	-3.17255	0.0015111	0.004927384	down-regulated	protein_coding
TMEM89	ENSMMUG00000014706	8.763580794	4.326941053	1.364114	3.171979	0.001514	0.004935982	up-regulated	protein_coding
RGS9	ENSMMUG00000014521	5.695804871	4.063780811	1.281584	3.170905	0.0015196	0.004953115	up-regulated	protein_coding
FUCA1	ENSMMUG00000004216	386.9006961	-1.978981405	0.624145	-3.17071	0.0015207	0.004955313	down-regulated	protein_coding
SMARCC1	ENSMMUG00000009345	158.4365438	-1.418313715	0.447339	-3.17056	0.0015215	0.004956806	down-regulated	protein_coding
SSR4	ENSMMUG00000003375	395.8304706	1.331218368	0.419948	3.169964	0.0015246	0.004965766	up-regulated	protein_coding
ZFAND4	ENSMMUG00000011030	27.67264774	1.721298851	0.543161	3.169042	0.0015294	0.004980391	up-regulated	protein_coding
PTS	ENSMMUG00000015255	13.96056979	-4.170260444	1.316064	-3.16874	0.001531	0.004984494	down-regulated	protein_coding
KTN1	ENSMMUG00000006650	255.8019503	-1.728226068	0.545412	-3.16866	0.0015314	0.004984594	down-regulated	protein_coding
SLC16A1	ENSMMUG00000006171	87.46133547	-1.836737404	0.579773	-3.16803	0.0015348	0.004994372	down-regulated	protein_coding
SLC39A11	ENSMMUG00000014195	107.8910231	-1.38514446	0.437357	-3.16708	0.0015398	0.005009154	down-regulated	protein_coding
COX15	ENSMMUG00000023762	24.94730602	-3.995072669	1.261456	-3.16703	0.00154	0.005009154	down-regulated	protein_coding
HOOK1	ENSMMUG00000012850	105.478561	1.630020583	0.514802	3.166305	0.0015439	0.005020561	up-regulated	protein_coding
NCK1	ENSMMUG00000007389	13.18607464	-4.090598966	1.291951	-3.16622	0.0015443	0.005020908	down-regulated	protein_coding
GNG10	ENSMMUG00000023304	197.3037928	-1.296785174	0.409623	-3.1658	0.0015466	0.005026955	down-regulated	protein_coding
UBL3	ENSMMUG00000003771	16.24278441	-4.392486104	1.387716	-3.16526	0.0015494	0.005035093	down-regulated	protein_coding
RHOV	ENSMMUG00000003155	29.57778436	-4.215986674	1.333396	-3.16184	0.0015677	0.00509347	down-regulated	protein_coding
FHL1	ENSMMUG00000007922	9.353470189	4.527056749	1.43188	3.161618	0.0015689	0.005096206	up-regulated	protein_coding
CTSZ	ENSMMUG00000015391	624.8491836	-1.272620929	0.402692	-3.16029	0.0015761	0.005118387	down-regulated	protein_coding
ECE2	ENSMMUG00000018955	14.4987047	3.117289371	0.986639	3.159505	0.0015804	0.005129782	up-regulated	protein_coding
TIMELESS	ENSMMUG00000021878	51.34383481	1.864655402	0.590188	3.159429	0.0015808	0.005129947	up-regulated	protein_coding
TNIP2	ENSMMUG00000007966	50.00712865	-2.526827772	0.800217	-3.15768	0.0015903	0.005159638	down-regulated	protein_coding
PDXK	ENSMMUG00000012300	106.346893	-1.411062762	0.446925	-3.15727	0.0015925	0.005165727	down-regulated	protein_coding
TOR2A	ENSMMUG00000016429	104.1368443	1.230577455	0.389803	3.156924	0.0015944	0.005170656	up-regulated	protein_coding
APC2	ENSMMUG00000003053	20.15326938	2.705078082	0.857224	3.155624	0.0016016	0.005191371	up-regulated	protein_coding
PIN1	ENSMMUG00000008245	35.98534806	-3.606566907	1.143009	-3.15533	0.0016032	0.00519547	down-regulated	protein_coding
FBXO3	ENSMMUG00000006063	37.60694631	-2.607740921	0.82654	-3.15501	0.0016049	0.005199947	down-regulated	protein_coding
SLC35E4	ENSMMUG00000019804	17.49991909	3.3280266	1.055284	3.15368	0.0016123	0.005222492	up-regulated	protein_coding
CMC2	ENSMMUG00000013081	14.04155393	-4.178757244	1.325353	-3.15294	0.0016164	0.005234559	down-regulated	protein_coding

SDHB	ENSMMUG00000001614	234.9246915	-1.562709305	0.495961	-3.15087	0.0016279	0.005270608	down-regulated	protein_coding
SEMA4G	ENSMMUG00000010680	392.2078031	-1.153344715	0.366068	-3.15063	0.0016292	0.005273645	down-regulated	protein_coding
DECR1	ENSMMUG00000006663	122.2314912	-1.726452538	0.548098	-3.1499	0.0016333	0.005285691	down-regulated	protein_coding
FZD1	ENSMMUG00000022114	58.46427682	1.823840088	0.579066	3.149622	0.0016348	0.005289522	up-regulated	protein_coding
SLC1A1	ENSMMUG00000000135	122.7322127	1.483492413	0.47103	3.149466	0.0016357	0.005291124	up-regulated	protein_coding
ALS2CL	ENSMMUG00000021247	149.7848539	-1.7716916	0.56263	-3.14895	0.0016386	0.005299348	down-regulated	protein_coding
PSMB8	ENSMMUG00000011301	68.82477905	-1.489675575	0.473112	-3.14868	0.0016401	0.005301798	down-regulated	protein_coding
HRAS	ENSMMUG00000021659	31.43388603	-4.33757468	1.377562	-3.14873	0.0016398	0.005301798	down-regulated	protein_coding
IRF5	ENSMMUG00000018478	68.49651864	1.816017891	0.576976	3.147474	0.0016469	0.005322439	up-regulated	protein_coding
MINOS1	ENSMMUG00000005855	77.34951883	-2.268639975	0.721127	-3.14596	0.0016554	0.005348779	down-regulated	protein_coding
SORCS3	ENSMMUG00000003610	20.4860181	4.482816669	1.425106	3.145603	0.0016574	0.005354157	up-regulated	protein_coding
FASTKD1	ENSMMUG00000020511	13.28738123	-4.101266282	1.303879	-3.14543	0.0016584	0.005356026	down-regulated	protein_coding
MAPKAP1	ENSMMUG00000016826	72.97329079	-1.778476005	0.565453	-3.14523	0.0016596	0.005358618	down-regulated	protein_coding
CPOX	ENSMMUG00000010833	54.88800003	-2.101652891	0.668511	-3.14378	0.0016678	0.005381429	down-regulated	protein_coding
LCA5L	ENSMMUG00000010407	20.786561	3.375067835	1.073749	3.143256	0.0016708	0.005389894	up-regulated	protein_coding
SLC39A1	ENSMMUG00000005007	94.9465124	-2.021735761	0.643219	-3.14315	0.0016714	0.005390538	down-regulated	protein_coding
GCNT3	ENSMMUG00000018426	112.8308895	-1.434105409	0.456274	-3.14308	0.0016718	0.005390657	down-regulated	protein_coding
ZC3H13	ENSMMUG00000017025	134.7584572	1.228012289	0.390765	3.142582	0.0016746	0.005398628	up-regulated	protein_coding
SEC11C	ENSMMUG00000004408	36.49098704	-3.680357061	1.171361	-3.14195	0.0016783	0.005409086	down-regulated	protein_coding
MYDGF	ENSMMUG00000028924	64.05594399	-2.719788392	0.865776	-3.14145	0.0016812	0.005417166	down-regulated	protein_coding
KPTN	ENSMMUG00000014723	19.03436246	-4.617757133	1.470289	-3.14071	0.0016854	0.005429469	down-regulated	protein_coding
PHF5A	ENSMMUG00000011354	13.92513593	-4.166484549	1.326721	-3.14044	0.001687	0.005433356	down-regulated	protein_coding
CYFIP1	ENSMMUG00000003890	271.1081544	-1.04128699	0.331748	-3.13879	0.0016965	0.005462802	down-regulated	protein_coding
AHNAK	ENSMMUG00000022301	3614.247157	1.316037572	0.419292	3.138712	0.0016969	0.005462971	up-regulated	protein_coding
LAMTOR3	ENSMMUG00000006830	30.9574089	-3.425821197	1.091784	-3.13782	0.0017021	0.00547775	down-regulated	protein_coding
CRELD1	ENSMMUG00000018285	71.88180267	-2.001228501	0.637874	-3.13734	0.0017049	0.005484846	down-regulated	protein_coding
ZNF330	ENSMMUG00000010446	30.44553323	-3.417612726	1.089491	-3.13689	0.0017075	0.005489527	down-regulated	protein_coding
MED10	ENSMMUG00000013521	35.41109078	-3.049169103	0.972019	-3.13694	0.0017072	0.005489527	down-regulated	protein_coding
OSBPL7	ENSMMUG00000019039	42.62529795	-2.761918243	0.880846	-3.13553	0.0017154	0.005513807	down-regulated	protein_coding
PITHD1	ENSMMUG00000019554	45.96523911	-2.379322094	0.7589	-3.13522	0.0017172	0.005517046	down-regulated	protein_coding
AP3S1	ENSMMUG00000015288	167.9411417	-1.446176307	0.461368	-3.13454	0.0017212	0.00552617	down-regulated	protein_coding
OSGIN1	ENSMMUG00000014219	162.3000157	-2.966889424	0.946552	-3.13442	0.001722	0.005527218	down-regulated	protein_coding
PPP1R2	ENSMMUG00000019396	80.98658378	-1.83475712	0.585392	-3.13424	0.001723	0.005529322	down-regulated	protein_coding
CCR7	ENSMMUG00000002102	6.586732799	5.398871821	1.722677	3.134002	0.0017244	0.005532541	up-regulated	protein_coding
ZXDC	ENSMMUG00000004195	39.00959828	-2.690953697	0.858831	-3.13327	0.0017287	0.005544446	down-regulated	protein_coding
MED25	ENSMMUG00000020175	138.2572647	-1.503364092	0.479812	-3.13324	0.0017289	0.005544446	down-regulated	protein_coding
MTERFD2	ENSMMUG00000006835	13.84744436	-4.159016496	1.327673	-3.13256	0.0017329	0.005555987	down-regulated	protein_coding
GPSM1	ENSMMUG00000003081	14.6138938	2.922164476	0.932876	3.132425	0.0017337	0.00555681	up-regulated	protein_coding
CSTB	ENSMMUG00000012303	262.4100826	-1.53054239	0.488619	-3.13238	0.0017339	0.00555681	down-regulated	protein_coding
PIGK	ENSMMUG00000020217	40.17406133	-3.016255484	0.963035	-3.13203	0.001736	0.005562225	down-regulated	protein_coding
VPS45	ENSMMUG00000013673	80.80869884	1.039689085	0.331963	3.13194	0.0017366	0.005562703	up-regulated	protein_coding
TCF7L2	ENSMMUG00000022799	207.862071	1.408470292	0.449808	3.131273	0.0017405	0.0055741	up-regulated	protein_coding
DTNBP1	ENSMMUG00000015363	70.05729936	1.500358106	0.479187	3.131052	0.0017418	0.005577043	up-regulated	protein_coding
SIX5	ENSMMUG00000016455	13.70055536	-4.143421324	1.323651	-3.1303	0.0017463	0.005590134	down-regulated	protein_coding
TNS2	ENSMMUG00000000318	148.3170642	1.628099474	0.520352	3.128841	0.001755	0.005616622	up-regulated	protein_coding
CTSD	ENSMMUG00000003874	709.2517364	-1.6303436	0.521179	-3.12818	0.0017589	0.005625364	down-regulated	protein_coding
TBCC	ENSMMUG00000001305	35.49593602	-3.042460216	0.972656	-3.12799	0.0017601	0.005627796	down-regulated	protein_coding
SKI	ENSMMUG00000010280	79.77047744	1.58445544	0.506772	3.126566	0.0017686	0.005652598	up-regulated	protein_coding
AOC1	ENSMMUG00000020435	800.2299349	-1.860731951	0.595132	-3.12659	0.0017685	0.005652598	down-regulated	protein_coding
RAB32	ENSMMUG00000006518	62.83160407	-2.034402266	0.651154	-3.1243	0.0017823	0.005693726	down-regulated	protein_coding
VPS9D1	ENSMMUG00000002505	14.24765649	-4.199878625	1.34438	-3.12403	0.0017839	0.005697767	down-regulated	protein_coding
VTA1	ENSMMUG00000023012	68.79146295	-1.600488952	0.51233	-3.12394	0.0017845	0.005698096	down-regulated	protein_coding
BET1	ENSMMUG00000000007	13.90767024	-4.168719535	1.335176	-3.12222	0.0017949	0.005730157	down-regulated	protein_coding
CARS	ENSMMUG00000000511	125.1453147	-1.452261417	0.465321	-3.12099	0.0018025	0.005752982	down-regulated	protein_coding
MTIF2	ENSMMUG00000030833	79.12854666	-1.84660547	0.591741	-3.12063	0.0018046	0.005758618	down-regulated	protein_coding
SLC9A8	ENSMMUG00000002715	107.2852102	1.25407325	0.401984	3.119711	0.0018103	0.005774067	up-regulated	protein_coding
AP1S3	ENSMMUG00000009710	12.2193003	-3.980405970	1.275879	-3.11974	0.0018101	0.005774067	down-regulated	protein_coding
MPZL1	ENSMMUG00000009184	72.23313818	-1.720630447	0.551561	-3.11956	0.0018112	0.005775662	down-regulated	protein_coding
SCML4	ENSMMUG00000014936	43.98016674	-2.183022183	0.700209	-3.11767	0.0018228	0.005811493	down-regulated	protein_coding
FIS1	ENSMMUG00000007839	184.8162582	-2.051728682	0.658269	-3.11685	0.0018279	0.005826368	down-regulated	protein_coding
Y_RNA	ENSMMUG00000036778	4.487915128	4.334831828	1.391038	3.116258	0.0018316	0.005835532	up-regulated	misc_RNA
LTC4S	ENSMMUG00000012373	20.21779794	2.106925824	0.676171	3.115967	0.0018334	0.005839976	up-regulated	protein_coding
OTX1	ENSMMUG00000016676	7.447391372	6.087438852	1.953756	3.115762	0.0018347	0.005842728	up-regulated	protein_coding
AGFG1	ENSMMUG00000013247	118.1677727	-1.829523168	0.587215	-3.11559	0.0018358	0.005844754	down-regulated	protein_coding
S100A6	ENSMMUG00000008917	1276.770188	-1.53825319	0.49391	-3.11444	0.0018429	0.005864991	down-regulated	protein_coding
RBM15	ENSMMUG00000015020	45.33891815	1.496249519	0.48047	3.114138	0.0018448	0.005869698	up-regulated	protein_coding
PHIP	ENSMMUG00000017186	92.79684378	1.555666877	0.499692	3.11325	0.0018504	0.005884736	up-regulated	protein_coding
GAB2	ENSMMUG00000017477	29.18141003	2.611409053	0.838831	3.113153	0.001851	0.005885344	up-regulated	protein_coding
ACTR8	ENSMMUG00000019269	53.85608847	-2.000099248	0.642526	-3.11287	0.0018528	0.005889669	down-regulated	protein_coding
SIVA1	ENSMMUG00000016557	30.42855263	-4.289142063	1.378042	-3.11249	0.0018552	0.005894594	down-regulated	protein_coding
CCDC158	ENSMMUG00000020916	6.085558807	5.212321284	1.674627	3.112527	0.0018549	0.005894594	up-regulated	protein_coding
STK11IP	ENSMMUG00000002519	80.99606409	-1.670543134	0.536836	-3.11183	0.0018593	0.005906489	down-regulated	protein_coding
RPS15	ENSMMUG00000003050	933.5938984	-2.377658245	0.764221	-3.11122	0.0018632	0.005916069	down-regulated	protein_coding
CSTF1	ENSMMUG00000000072	13.11405329	-4.080467057	1.311575	-3.11112	0.0018638	0.005916715	down-regulated	protein_coding
RNF115	ENSMMUG00000016736	50.05224374	1.373676024	0.441695	3.110012	0.0018708	0.005937626	up-regulated	protein_coding
SET	ENSMMUG00000008532	113.1296188	-1.291853195	0.415464	-3.10943	0.0018745	0.00594808	down-regulated	protein_coding
CECR5	ENSMMUG00000031124	38.38901179	-3.186148831	1.024736	-3.10924	0.0018757	0.005950488	down-regulated	protein_coding
RBP5	ENSMMUG00000008898	4.257925636	4.628492521	1.488899	3.108668	0.0018793	0.005960688	up-regulated	protein_coding
CDC16	ENSMMUG00000007439	41.61923677	-2.703710205	0.869891	-3.1081	0.0018829	0.005970791	down-regulated	protein_coding
RPL21	ENSMMUG00000028889	117.1804261	-2.697725811	0.868347	-3.10674	0.0018916	0.005995729	down-regulated	protein_coding
ZMIZ2	ENSMMUG00000002652	231.6987693	-1.4265823	0.459219	-3.10654	0.0018929	0.005998349	down-regulated	protein_coding
RITA1	ENSMMUG00000001129	14.82329092	-4.257032955	1.370517	-3.10615	0.0018954	0.006004934	down-regulated	protein_coding
PYHIN1	ENSMMUG00000004488	4.985694373	4.964925002	1.598472	3.106044	0.0018961	0.006005751	up-regulated	protein_coding
KIF1BP	ENSMMUG00000008985	34.23882836	-2.512160246	0.808853	-3.10583	0.0018975	0.006007409	down-regulated	protein_coding
TMEM97	ENSMMUG00000019142	23.06721527	-3.876813497	1.248231	-3.10585	0.0018973	0.006007409	down-regulated	protein_coding
SDC3	ENSMMUG00000013519	193.5722066	1.139019368	0.366825	3.105079	0.0019023	0.00602135	up-regulated	protein_coding
N4BP2L1	ENSMMUG00000007198	20.11691723	2.38058358	0.766892	3.104198	0.001908	0.00603659	up-regulated	protein_coding
C2CD3	ENSMMUG00000010321	61.61533182	1.452319951	0.467848	3.104257	0.0019076	0.00603659	up-regulated	protein_coding
TCEB3	ENSMMUG00000003173	88.42373331	1.117140658	0.359935	3.103726	0.001911	0.006043538	up-regulated	protein_coding
FXR1	ENSMMUG00000012105	110.7310475	-1.441888301	0.464563	-3.10375	0.0019108	0.006043538	down-regulated	protein_coding
C1GALT1	ENSMMUG00000016338	28.6690713	-4.171069247	1.344327	-3.10272	0.0019175	0.006061403	down-regulated	protein_coding
EHD4	ENSMMUG00000003846	141.1405219	-1.542147353	0.497089	-3.10235	0.0019199	0.006067542	down-regulated	protein_coding
LRRC66	ENSMMUG00000018939	102.9504398	-1.871965125	0.60343	-3.10221	0.0019208	0.00606923	down-regulated	protein_coding
GALNT3	ENSMMUG00000019177	133.4576492	-1.525051762	0.491639	-3.10198	0.0019223	0.006072593	down-regulated	protein_coding
SDHAF2	ENSMMUG00000015281	57.98628251	-2.111740577	0.680808	-3.10182	0.0019234	0.006074524	down-regulated	protein_coding
GLB1	ENSMMUG00000020109	69.64189395	-2.866722164	0.924286	-3.10155	0.0019251	0.006078524	down-regulated	protein_coding
ATAD1	ENSMMUG00000006414	28.36959746	-3.297989526	1.0636	-3.10078	0.0019301	0.006091251	down-regulated	protein_coding
C19ORF22	ENSMMUG00000009682	106.2498406	-1.842732524	0.594266	-3.10086	0.0019296	0.006091251	down-regulated	protein_coding
MED7	ENSMMUG00000010993	12.32626397	-3.992254657	1.287518	-3.10074	0.0019304	0.006091251	down-regulated	protein_coding
UFSP2	ENSMMUG00000003520	13.57835643	-4.130103645	1.332752	-3.09893	0.0019422	0.006127164	down-regulated	protein_coding

APPL2	ENSMMUG00000009524	161.3964011	1.427744877	0.46093	3.097528	0.0019514	0.006154836	up-regulated	protein_coding
BCO1	ENSMMUG00000000720	170.1516557	-2.399179035	0.774606	-3.09729	0.001953	0.006158435	down-regulated	protein_coding
KBTBD10	ENSMMUG00000020510	5.727315608	3.780183288	1.220663	3.096828	0.001956	0.006166651	up-regulated	protein_coding
ANTXR2	ENSMMUG00000003333	56.14327565	-2.847892074	0.919843	-3.09606	0.0019611	0.006180171	down-regulated	protein_coding
GSTM4	ENSMMUG00000019630	1159.814939	-1.426104847	0.460621	-3.09605	0.0019612	0.006180171	down-regulated	protein_coding
SUCLA2	ENSMMUG00000007539	73.72447983	-1.9782539	0.639165	-3.09506	0.0019677	0.006196647	down-regulated	protein_coding
RXRA	ENSMMUG00000008183	98.08670544	-2.252899772	0.727889	-3.09511	0.0019674	0.006196647	down-regulated	protein_coding
CD302	ENSMMUG00000020720	44.73842155	2.010916407	0.649692	3.095184	0.0019669	0.006196647	up-regulated	protein_coding
SEC61B	ENSMMUG00000010661	67.45023958	-2.538046059	0.820158	-3.09458	0.0019709	0.00620521	down-regulated	protein_coding
FBXO9	ENSMMUG00000005340	61.34432134	-1.651840661	0.533869	-3.09409	0.0019742	0.006214128	down-regulated	protein_coding
ZC3H7A	ENSMMUG00000011242	45.47338664	-1.857103292	0.600231	-3.09398	0.0019749	0.006215079	down-regulated	protein_coding
KDM5B	ENSMMUG00000015233	98.61321882	1.333226631	0.430921	3.093901	0.0019754	0.006215347	up-regulated	protein_coding
RAB2B	ENSMMUG00000014992	16.22839686	-4.390616449	1.419815	-3.09239	0.0019855	0.006245786	down-regulated	protein_coding
CACFD1	ENSMMUG00000010976	13.79249149	-4.156468345	1.344887	-3.09057	0.0019977	0.006281261	down-regulated	protein_coding
CHI3L2	ENSMMUG00000002455	5.584620993	6.073634851	1.965486	3.090144	0.0020006	0.0062876	up-regulated	protein_coding
SRSF1	ENSMMUG00000004725	222.7601066	-1.821224484	0.589366	-3.09014	0.0020006	0.0062876	down-regulated	protein_coding
RPS6KB2	ENSMMUG00000005483	75.47363598	-2.368197177	0.766395	-3.09005	0.0020013	0.006288194	down-regulated	protein_coding
RNF151	ENSMMUG00000007940	3.608097339	4.760073024	1.540706	3.08954	0.0020047	0.006297522	up-regulated	protein_coding
GPCPD1	ENSMMUG00000001809	40.70692751	-2.730796909	0.884207	-3.08841	0.0020123	0.006320069	down-regulated	protein_coding
GOLT1A	ENSMMUG00000017112	42.24986427	-3.073073539	0.995257	-3.08772	0.002017	0.006333433	down-regulated	protein_coding
ZNF217	ENSMMUG00000018358	102.7833621	-2.280581821	0.738867	-3.08659	0.0020246	0.006356021	down-regulated	protein_coding
TANK	ENSMMUG00000001680	23.50015599	-3.865565419	1.252404	-3.08652	0.0020252	0.006356038	down-regulated	protein_coding
HIST1H4K	ENSMMUG00000029921	4.765595409	3.910798403	1.267147	3.086303	0.0020266	0.006358026	up-regulated	protein_coding
DGCR2	ENSMMUG00000019925	185.2809966	1.370194443	0.444088	3.085415	0.0020327	0.006375631	up-regulated	protein_coding
SDCCAG3	ENSMMUG00000003089	46.17173512	-2.553424198	0.827598	-3.08534	0.0020332	0.006375714	down-regulated	protein_coding
JRKL	ENSMMUG00000003821	12.59570176	-4.024027735	1.304417	-3.08492	0.002036	0.006383336	down-regulated	protein_coding
STX10	ENSMMUG00000011427	56.58524954	-2.419723365	0.784486	-3.08447	0.0020391	0.006391629	down-regulated	protein_coding
H1F0	ENSMMUG00000015257	1522.351793	1.079004595	0.349909	3.083668	0.0020447	0.006407495	up-regulated	protein_coding
ARNTL2	ENSMMUG00000023249	36.37667153	-2.643756973	0.857838	-3.08188	0.002057	0.006444607	down-regulated	protein_coding
SLC22A23	ENSMMUG00000001371	74.97543749	1.651194853	0.535883	3.081258	0.0020613	0.00645672	up-regulated	protein_coding
FBXL19	ENSMMUG00000003230	32.29758726	-3.504043664	1.137364	-3.08085	0.0020641	0.006464254	down-regulated	protein_coding
GPR176	ENSMMUG00000006039	18.30829535	3.515136407	1.141321	3.079883	0.0020708	0.006483699	up-regulated	protein_coding
ARIH2	ENSMMUG00000010571	9.962304692	2.39463129	0.777667	3.07925	0.0020752	0.006494656	up-regulated	protein_coding
USP16	ENSMMUG00000003020	92.21551699	-1.60135805	0.520128	-3.07877	0.0020785	0.006501905	down-regulated	protein_coding
TEFM	ENSMMUG00000009073	37.01021994	1.753347987	0.569492	3.078793	0.0020784	0.006501905	up-regulated	protein_coding
BBS12	ENSMMUG00000030171	12.00402643	2.223236239	0.72213	3.07872	0.0020789	0.006501905	up-regulated	protein_coding
SMUG1	ENSMMUG00000011642	34.21203273	-2.461219166	0.799502	-3.07844	0.0020809	0.006506612	down-regulated	protein_coding
SNORD49	ENSMMUG00000026295	10.75855478	3.998861714	1.299175	3.078	0.0020839	0.006514748	up-regulated	snoRNA
UBE2L3	ENSMMUG00000018994	177.910209	-1.228181071	0.399056	-3.07772	0.0020859	0.0065195	down-regulated	protein_coding
BCL7B	ENSMMUG00000023063	133.5171299	-1.338416696	0.434899	-3.07753	0.0020872	0.006521527	down-regulated	protein_coding
ADK	ENSMMUG00000022237	47.66421554	-2.429554143	0.790155	-3.07478	0.0021066	0.006579651	down-regulated	protein_coding
ULK3	ENSMMUG00000012972	51.6515159	-1.879071948	0.611257	-3.07411	0.0021113	0.006592998	down-regulated	protein_coding
COMMD4	ENSMMUG00000021509	66.48382756	-2.504998528	0.814951	-3.0738	0.0021135	0.006598354	down-regulated	protein_coding
TOMM20L	ENSMMUG00000003516	10.78831101	4.597251231	1.496246	3.072524	0.0021226	0.006623777	up-regulated	protein_coding
SETD4	ENSMMUG00000001125	61.30369009	-2.133712618	0.6945	-3.0723	0.0021242	0.006627275	down-regulated	protein_coding
CCDC45	ENSMMUG00000009459	56.2348968	1.65292059	0.538298	3.070642	0.002136	0.006661961	up-regulated	protein_coding
ZFYVE21	ENSMMUG00000018121	46.25235795	-2.349736617	0.765234	-3.07061	0.0021362	0.006661961	down-regulated	protein_coding
ZNF160	ENSMMUG00000001106	14.96218513	-4.273581534	1.392559	-3.06887	0.0021487	0.006699456	down-regulated	protein_coding
GLRX5	ENSMMUG00000017079	94.26989615	-1.785065826	0.581742	-3.06848	0.0021515	0.006706645	down-regulated	protein_coding
IMMT	ENSMMUG00000015824	93.65367039	-1.277631194	0.416384	-3.06839	0.0021521	0.00670716	down-regulated	protein_coding
PAQR5	ENSMMUG00000014886	32.92987285	1.95753048	0.638032	3.068073	0.0021544	0.006712866	up-regulated	protein_coding
ALMS1	ENSMMUG00000002819	33.15076635	1.638409448	0.53407	3.067779	0.0021566	0.006716528	up-regulated	protein_coding
KCNK10	ENSMMUG00000021010	14.61334706	3.47137372	1.131649	3.067536	0.0021583	0.006720513	up-regulated	protein_coding
SNX7	ENSMMUG00000008515	44.91807529	-1.929618683	0.629092	-3.06731	0.00216	0.006724122	down-regulated	protein_coding
NEK10	ENSMMUG00000019324	3.556755861	4.354369421	1.419819	3.066849	0.0021633	0.006733004	up-regulated	protein_coding
AKAP7	ENSMMUG00000000577	14.94416915	-4.271881886	1.393208	-3.06622	0.0021678	0.006744246	down-regulated	protein_coding
C8H8ORF4	ENSMMUG00000022984	28.26094502	-3.284796084	1.071508	-3.06558	0.0021725	0.006757103	down-regulated	protein_coding
PDE4D	ENSMMUG00000012132	25.0012715	2.221709538	0.724904	3.064833	0.0021779	0.006772609	up-regulated	protein_coding
RFX7	ENSMMUG00000017801	58.58441891	1.667136914	0.544034	3.064397	0.0021811	0.006780978	up-regulated	protein_coding
PTER	ENSMMUG00000016063	29.7586003	-3.377522253	1.102255	-3.06419	0.0021826	0.006784151	down-regulated	protein_coding
COL8A1	ENSMMUG00000002935	6.825583506	6.184403866	2.018414	3.063992	0.002184	0.006787181	up-regulated	protein_coding
B3GAT3	ENSMMUG00000031126	46.22511896	-2.926660801	0.95539	-3.06332	0.002189	0.006801057	down-regulated	protein_coding
B3GNT7	ENSMMUG00000011777	221.7655098	-1.274672335	0.41625	-3.06228	0.0021966	0.006823225	down-regulated	protein_coding
METTL2A	ENSMMUG00000009802	23.94870553	-3.892713936	1.271311	-3.06197	0.0021989	0.006828723	down-regulated	protein_coding
CYB5B	ENSMMUG00000012039	59.81506254	-1.801991542	0.58868	-3.06107	0.0022054	0.006847684	down-regulated	protein_coding
TRAK2	ENSMMUG00000023812	136.0144908	-1.407877111	0.459997	-3.06063	0.0022088	0.006856444	down-regulated	protein_coding
KDM6A	ENSMMUG00000003317	41.59658845	-2.490603772	0.813865	-3.06022	0.0022118	0.006864309	down-regulated	protein_coding
FCHO2	ENSMMUG00000015422	75.97499768	-2.171292722	0.709596	-3.0599	0.0022141	0.006870006	down-regulated	protein_coding
MOGAT2	ENSMMUG00000000506	123.2483686	-1.995633217	0.652231	-3.0597	0.0022156	0.006873112	down-regulated	protein_coding
HIF1AN	ENSMMUG00000006637	34.25834021	-2.533007612	0.828022	-3.05911	0.00222	0.006885229	down-regulated	protein_coding
CLNS1A	ENSMMUG00000018945	66.52176587	-2.632676581	0.860766	-3.05853	0.0022243	0.006897079	down-regulated	protein_coding
CA5B	ENSMMUG00000014052	8.401743917	2.80094491	0.915824	3.058387	0.0022253	0.006898782	up-regulated	protein_coding
TM4SF1	ENSMMUG00000017215	212.769761	-2.051331124	0.670813	-3.05798	0.0022283	0.006906651	down-regulated	protein_coding
LUC7L3	ENSMMUG00000010117	132.3297053	-1.786560305	0.584306	-3.05758	0.0022314	0.006914462	down-regulated	protein_coding
COPS7A	ENSMMUG00000009583	64.20283763	-1.713342868	0.560407	-3.05732	0.0022333	0.006918826	down-regulated	protein_coding
C12ORF41	ENSMMUG00000003877	47.69507676	-2.112245144	0.691038	-3.05663	0.0022384	0.006933359	down-regulated	protein_coding
OR10G3	ENSMMUG00000003550	6.398954603	4.589471753	1.501709	3.056165	0.0022419	0.006942507	up-regulated	protein_coding
RFNG	ENSMMUG00000012799	81.70003042	-2.066244956	0.676112	-3.05607	0.0022426	0.006943219	down-regulated	protein_coding
ACOT8	ENSMMUG00000012510	169.4168855	1.582253749	0.517783	3.055823	0.0022444	0.006945873	up-regulated	protein_coding
TCOF1	ENSMMUG00000009111	37.94979655	1.61788574	0.529656	3.054599	0.0022536	0.006972766	up-regulated	protein_coding
MMADHC	ENSMMUG00000005633	85.86425637	-1.85378815	0.607091	-3.05356	0.0022614	0.006995433	down-regulated	protein_coding
LRRC1	ENSMMUG00000011049	131.6529018	-1.278321766	0.418731	-3.05285	0.0022668	0.007010526	down-regulated	protein_coding
GNB2L1	ENSMMUG00000003604	1337.289126	-1.889844174	0.619124	-3.05245	0.0022698	0.007018342	down-regulated	protein_coding
TAPT1	ENSMMUG00000001747	23.39823788	-3.859842039	1.264585	-3.05226	0.0022712	0.007019641	down-regulated	protein_coding
BHLHE41	ENSMMUG00000018629	12.63520158	-4.028901473	1.320511	-3.05102	0.0022807	0.007047231	down-regulated	protein_coding
FBXO25	ENSMMUG00000011105	41.19063827	-2.307882328	0.756634	-3.0502	0.0022869	0.007063414	down-regulated	protein_coding
ANKRD46	ENSMMUG00000009958	59.58738892	-2.25693278	0.740118	-3.04942	0.0022928	0.007080097	down-regulated	protein_coding
AKR1B1	ENSMMUG00000000917	40.04116935	-3.261542199	1.069693	-3.04904	0.0022957	0.007085936	down-regulated	protein_coding
SETDB2	ENSMMUG00000013473	11.70887514	-3.918615269	1.285182	-3.04907	0.0022955	0.007085936	down-regulated	protein_coding
A4GALT	ENSMMUG00000010194	104.8110793	-1.451458723	0.476272	-3.04754	0.0023072	0.007119946	down-regulated	protein_coding
ZBED1	ENSMMUG00000007514	116.2420393	1.211947166	0.39769	3.047466	0.0023078	0.007120153	up-regulated	protein_coding
IFNAR1	ENSMMUG00000003961	42.93191247	-2.746883615	0.901388	-3.04739	0.0023084	0.007120317	down-regulated	protein_coding
BID	ENSMMUG00000020799	137.6827144	-1.33560931	0.438382	-3.04668	0.0023138	0.007135655	down-regulated	protein_coding
SH3RF1	ENSMMUG00000012701	93.98026167	-1.87919698	0.616967	-3.04586	0.0023201	0.007153568	down-regulated	protein_coding
CHPT1	ENSMMUG00000020877	59.47916098	-2.779272283	0.912691	-3.04514	0.0023257	0.007169219	down-regulated	protein_coding
TIA1	ENSMMUG00000001126	67.1314731	-1.752576115	0.575647	-3.04453	0.0023304	0.007178993	down-regulated	protein_coding
KIAA1551	ENSMMUG00000023575	62.0437399	-2.393590204	0.786462	-3.04349	0.0023385	0.007202281	down-regulated	protein_coding
IKBKE	ENSMMUG00000002526	121.153509	1.711305491	0.562297	3.043419	0.0023391	0.007202496	up-regulated	protein_coding
TMEM33	ENSMMUG00000020940	70.54823544	-1.907190335	0.626745	-3.04301	0.0023423	0.0072092	down-regulated	protein_coding
MITD1	ENSMMUG00000000572	13.77890008	-4.151382909	1.365614	-3.03994	0.0023663	0.007278317	down-regulated	protein_coding

UXT	ENSMMUG00000018535	117.6534154	1.424542695	0.468626	3.039827	0.0023671	0.007279425	up-regulated	protein_coding
CLPP	ENSMMUG00000028914	34.05952233	-2.979305051	0.980144	-3.03966	0.0023685	0.007281882	down-regulated	protein_coding
XYLT2	ENSMMUG00000017226	102.9929313	1.036742552	0.34112	3.039235	0.0023718	0.007290577	up-regulated	protein_coding
PPP2R5B	ENSMMUG00000003975	222.2805628	-1.126114003	0.370585	-3.03875	0.0023757	0.007300856	down-regulated	protein_coding
VILL	ENSMMUG00000000984	321.3082346	-1.151058681	0.378886	-3.03801	0.0023815	0.007317223	down-regulated	protein_coding
CXADR	ENSMMUG00000010755	78.82107121	-2.119645988	0.697747	-3.03784	0.0023828	0.007317947	down-regulated	protein_coding
MYO10	ENSMMUG00000012672	411.5356398	1.285851238	0.423273	3.037875	0.0023825	0.007317947	up-regulated	protein_coding
AFTPH	ENSMMUG00000006503	117.1331796	-1.205259277	0.396771	-3.03767	0.0023841	0.007320538	down-regulated	protein_coding
PRELID2	ENSMMUG00000013588	53.50447584	-2.151888471	0.708507	-3.03722	0.0023877	0.007329031	down-regulated	protein_coding
CHAMP1	ENSMMUG00000023599	82.80566042	1.309285498	0.431084	3.037192	0.0023879	0.007329031	up-regulated	protein_coding
DDX39A	ENSMMUG00000005723	96.88963212	-2.535711056	0.834933	-3.03702	0.0023893	0.007331584	down-regulated	protein_coding
ZNF316	ENSMMUG00000010222	12.55605705	-4.018599736	1.323235	-3.03695	0.0023898	0.007331723	down-regulated	protein_coding
PECR	ENSMMUG00000017964	48.05889357	-2.158912115	0.710932	-3.03673	0.0023916	0.007335421	down-regulated	protein_coding
NRAS	ENSMMUG00000001977	35.96233555	-3.508872723	1.155911	-3.03559	0.0024006	0.007361663	down-regulated	protein_coding
MGAT3	ENSMMUG00000004495	71.79983706	-2.526977539	0.832605	-3.03503	0.0024051	0.007373871	down-regulated	protein_coding
ENO2	ENSMMUG00000009443	99.40161739	-3.337725691	1.099975	-3.03437	0.0024104	0.007386836	down-regulated	protein_coding
ELAVL1	ENSMMUG00000006544	127.1496145	-1.736404293	0.572314	-3.03401	0.0024133	0.007394016	down-regulated	protein_coding
COPS4	ENSMMUG00000022312	40.21676902	-2.137980405	0.704784	-3.03352	0.0024172	0.007402672	down-regulated	protein_coding
ATP5G3	ENSMMUG00000029649	23.81623151	-3.923061398	1.293264	-3.03346	0.0024177	0.007402707	down-regulated	protein_coding
SUDS3	ENSMMUG00000020629	80.01565413	1.209973655	0.398909	3.033208	0.0024197	0.007405674	up-regulated	protein_coding
AXL	ENSMMUG00000009145	21.21553382	2.425643155	0.799923	3.032346	0.0024266	0.007425204	up-regulated	protein_coding
SLC30A5	ENSMMUG00000018143	53.07492031	-2.332939655	0.769454	-3.03194	0.0024299	0.007433546	down-regulated	protein_coding
FMO4	ENSMMUG00000014230	40.0765685	-2.805864567	0.925542	-3.03159	0.0024327	0.007440611	down-regulated	protein_coding
LHB	ENSMMUG00000003917	14.30665908	-4.205386013	1.387367	-3.0312	0.0024358	0.0074432	down-regulated	protein_coding
GTF2F1	ENSMMUG00000007904	104.3890522	-1.275845665	0.420883	-3.03136	0.0024346	0.0074432	down-regulated	protein_coding
MMAB	ENSMMUG00000011665	36.4146788	-2.940036515	0.96992	-3.03122	0.0024357	0.0074432	down-regulated	protein_coding
PAPSS1	ENSMMUG00000019907	36.25693393	-2.632143171	0.868354	-3.03119	0.0024359	0.0074432	down-regulated	protein_coding
U1	ENSMMUG00000025581	5.183667205	4.421789615	1.458779	3.031158	0.0024362	0.0074432	up-regulated	snRNA
TRIM7	ENSMMUG00000003598	8.600442841	4.848705586	1.599733	3.030947	0.0024379	0.007446793	up-regulated	protein_coding
PPIH	ENSMMUG00000013129	28.17343571	-4.178593236	1.379042	-3.03007	0.002445	0.007466846	down-regulated	protein_coding
DLAT	ENSMMUG00000017568	94.10384325	-1.492828865	0.492715	-3.0298	0.0024472	0.007471921	down-regulated	protein_coding
NUF2	ENSMMUG00000001152	19.04941349	-4.619115222	1.524708	-3.02951	0.0024495	0.007476025	down-regulated	protein_coding
BOLA3	ENSMMUG00000015207	38.70631113	-3.014440196	0.995028	-3.0295	0.0024496	0.007476025	down-regulated	protein_coding
ZNF33A	ENSMMUG00000015471	11.4813464	-3.890853596	1.284694	-3.02862	0.0024567	0.007495504	down-regulated	protein_coding
ARID3C	ENSMMUG00000020786	21.73095464	-3.786755886	1.250338	-3.02859	0.002457	0.007495504	down-regulated	protein_coding
ALG8	ENSMMUG00000017472	13.56366557	-4.128315453	1.363343	-3.02808	0.0024611	0.007506401	down-regulated	protein_coding
FAM91A1	ENSMMUG00000019646	61.20152417	-2.319854869	0.766453	-3.02674	0.002472	0.007536504	down-regulated	protein_coding
LMBR1L	ENSMMUG00000012769	65.55312427	-1.669777555	0.551721	-3.02631	0.0024756	0.00754575	down-regulated	protein_coding
ALG9	ENSMMUG00000014003	12.43652791	-4.004561334	1.323426	-3.0259	0.0024789	0.007554186	down-regulated	protein_coding
MFSD4	ENSMMUG00000004113	21.23921275	2.182991662	0.721563	3.025366	0.0024833	0.007566016	up-regulated	protein_coding
ILVBL	ENSMMUG00000007729	64.08777704	-1.81119398	0.598715	-3.02513	0.0024852	0.00757021	down-regulated	protein_coding
KLHL21	ENSMMUG00000018181	22.58805003	-3.804160772	1.257609	-3.02491	0.002487	0.007574045	down-regulated	protein_coding
ANKRD44	ENSMMUG00000022425	9.122291753	3.537458673	1.169521	3.024708	0.0024887	0.007577594	up-regulated	protein_coding
INPP4A	ENSMMUG00000022903	36.33996489	2.373479708	0.78502	3.023463	0.002499	0.007603938	up-regulated	protein_coding
S100A13	ENSMMUG00000008144	13.57996028	-4.130404023	1.366242	-3.02319	0.0025013	0.007609238	down-regulated	protein_coding
FPR1	ENSMMUG00000013049	14.00518586	5.930387102	1.962159	3.022378	0.002508	0.007627956	up-regulated	protein_coding
ZNF561	ENSMMUG00000015388	11.84868449	-3.93677555	1.302706	-3.022	0.0025111	0.00763428	down-regulated	protein_coding
LZIC	ENSMMUG00000013292	31.27171249	-2.72061591	0.900298	-3.0219	0.0025119	0.007634971	down-regulated	protein_coding
GPSM2	ENSMMUG00000010161	26.83873578	2.209426258	0.731322	3.021141	0.0025182	0.007649335	up-regulated	protein_coding
MEF2A	ENSMMUG00000018122	51.56875089	-1.967480858	0.651271	-3.02098	0.0025195	0.00765164	down-regulated	protein_coding
DCTD	ENSMMUG00000000284	76.72726809	-2.121426488	0.702429	-3.02013	0.0025267	0.007671589	down-regulated	protein_coding
CEBPB	ENSMMUG00000004874	79.66985691	-2.057045722	0.681174	-3.01985	0.002529	0.007675314	down-regulated	protein_coding
TMEM80	ENSMMUG00000005162	106.2565858	1.501361776	0.497154	3.019912	0.0025285	0.007675314	up-regulated	protein_coding
PPTC7	ENSMMUG00000013156	42.27886693	-2.721917735	0.901499	-3.01932	0.0025334	0.007687087	down-regulated	protein_coding
OXCT1	ENSMMUG00000011027	23.81026846	-3.927797164	1.301038	-3.01897	0.0025363	0.007694389	down-regulated	protein_coding
PRKRIR	ENSMMUG00000022399	57.21563523	-1.617693761	0.535887	-3.01872	0.0025384	0.007699138	down-regulated	protein_coding
NMI	ENSMMUG00000017781	29.41706265	-3.238679585	1.072895	-3.01864	0.0025391	0.007699613	down-regulated	protein_coding
AUTS2	ENSMMUG00000017675	141.8460726	1.149459191	0.3808	3.018536	0.00254	0.007700531	up-regulated	protein_coding
HBS1L	ENSMMUG00000004802	71.02954203	-1.810056729	0.599722	-3.01816	0.0025431	0.007703464	down-regulated	protein_coding
TPM3	ENSMMUG00000010805	508.3197551	-1.632930274	0.54102	-3.01824	0.0025424	0.007703464	down-regulated	protein_coding
WDR5	ENSMMUG00000014159	44.34740855	-2.516601645	0.833796	-3.01825	0.0025424	0.007703464	down-regulated	protein_coding
TSEN15	ENSMMUG00000016409	25.1722914	-4.006328832	1.327676	-3.01755	0.0025483	0.007716293	down-regulated	protein_coding
SIRT2	ENSMMUG00000023640	32.13061896	-2.86358429	0.948984	-3.01753	0.0025485	0.007716293	down-regulated	protein_coding
HCFC2	ENSMMUG00000022571	14.07976388	-4.186033675	1.387345	-3.0173	0.0025504	0.007720433	down-regulated	protein_coding
FAM70B	ENSMMUG00000007432	21.90709091	-3.799274083	1.259435	-3.01665	0.0025558	0.007733668	down-regulated	protein_coding
FAM120C	ENSMMUG00000008760	14.71831607	2.118538786	0.702499	3.015717	0.0025637	0.007755857	up-regulated	protein_coding
CNIH2	ENSMMUG00000006950	7.769372359	2.766859534	0.918026	3.013924	0.0025789	0.007800169	up-regulated	protein_coding
LRRC8D	ENSMMUG00000012828	134.097807	1.197909295	0.397582	3.012986	0.0025869	0.00782264	up-regulated	protein_coding
PCNX	ENSMMUG00000019470	154.1775779	1.571546352	0.521609	3.01288	0.0025878	0.0078237	up-regulated	protein_coding
METTL9	ENSMMUG00000013872	60.24095358	-1.881214072	0.624484	-3.01243	0.0025916	0.007833598	down-regulated	protein_coding
RIC8A	ENSMMUG00000011506	92.1024034	-1.348591506	0.447813	-3.01151	0.0025995	0.007855765	down-regulated	protein_coding
BOLA1	ENSMMUG00000007258	32.11434625	-3.498126695	1.161708	-3.01119	0.0026022	0.007860594	down-regulated	protein_coding
PACS1	ENSMMUG00000022518	131.9135998	1.229911709	0.408519	3.010662	0.0026068	0.007872673	up-regulated	protein_coding
POP7	ENSMMUG00000008581	13.86302086	-4.160154786	1.382172	-3.00987	0.0026136	0.007891614	down-regulated	protein_coding
PDCD5	ENSMMUG00000015395	41.77907355	-2.767429854	0.919473	-3.0098	0.0026142	0.007891658	down-regulated	protein_coding
MRPS35	ENSMMUG00000021741	47.00121653	-2.67683207	0.889491	-3.0094	0.0026177	0.007900495	down-regulated	protein_coding
REPIN1	ENSMMUG00000022633	204.5046133	-1.035235747	0.344031	-3.00914	0.0026199	0.00790554	down-regulated	protein_coding
NLRC5	ENSMMUG00000000769	14.61268824	2.788600818	0.926869	3.008626	0.0026243	0.007916293	up-regulated	protein_coding
C3orf38	ENSMMUG00000021507	11.51926219	-3.89448675	1.294482	-3.00853	0.0026251	0.007916293	down-regulated	protein_coding
LPGAT1	ENSMMUG00000020560	33.70844497	-4.416322953	1.467987	-3.00842	0.0026261	0.007917437	down-regulated	protein_coding
OTOF	ENSMMUG00000014292	21.48153865	4.274471743	1.421296	3.007447	0.0026345	0.007939476	up-regulated	protein_coding
RCE1	ENSMMUG00000004531	164.181168	1.85014651	0.615606	3.005406	0.0026523	0.007991247	up-regulated	protein_coding
TIMP-2	ENSMMUG00000005355	19.95826758	-4.688586474	1.561059	-3.00346	0.0026692	0.008038988	down-regulated	protein_coding
ZEB2	ENSMMUG00000016845	12.87395949	4.53255573	1.509614	3.002459	0.0026781	0.00806387	up-regulated	protein_coding
TKFC	ENSMMUG00000002692	487.6037171	-1.397547138	0.465522	-3.00211	0.0026812	0.008069747	down-regulated	protein_coding
PDCD7	ENSMMUG00000006297	29.97221532	-2.767504737	0.921841	-3.00215	0.0026808	0.008069747	down-regulated	protein_coding
POC1B	ENSMMUG00000003574	116.2965454	-1.429873377	0.476303	-3.00203	0.0026819	0.008070179	down-regulated	protein_coding
COMMD8	ENSMMUG00000021777	11.99835688	-3.952965121	1.316858	-3.00181	0.0026838	0.008074084	down-regulated	protein_coding
SLC34A3	ENSMMUG00000012165	9.853828059	3.952264059	1.317481	2.999864	0.002701	0.008124249	up-regulated	protein_coding
KLHL22	ENSMMUG00000008152	47.73494428	-1.902417376	0.634498	-2.9983	0.0027149	0.008160758	down-regulated	protein_coding
AQP11	ENSMMUG00000018944	24.59147101	-3.936075505	1.31275	-2.99834	0.0027145	0.008160758	down-regulated	protein_coding
LDAH	ENSMMUG00000003355	97.42272636	-2.043222354	0.681599	-2.99769	0.0027203	0.008175482	down-regulated	protein_coding
TEF	ENSMMUG00000005221	165.7776352	1.631096734	0.544162	2.997447	0.0027225	0.008180251	up-regulated	protein_coding
CLYBL	ENSMMUG00000003558	12.58713239	-4.02179601	1.341942	-2.997	0.0027265	0.008190633	down-regulated	protein_coding
TMEM17	ENSMMUG00000021214	12.13286662	2.961650033	0.988249	2.996866	0.0027277	0.00819107	up-regulated	protein_coding
XRCC4	ENSMMUG00000021609	21.37882397	-3.766483878	1.256814	-2.99685	0.0027278	0.00819107	down-regulated	protein_coding
AMD1	ENSMMUG00000000535	67.9823586	-1.521603702	0.507786	-2.99654	0.0027306	0.008195824	down-regulated	protein_coding
HEXDC	ENSMMUG00000019221	35.48250494	-3.481700029	1.161895	-2.99657	0.0027304	0.008195824	down-regulated	protein_coding
HIPK2	ENSMMUG00000002481	145.0863065	1.866282631	0.622922	2.996013	0.0027353	0.008206619	up-regulated	protein_coding
GPRIN3	ENSMMUG00000007840	85.80512996	1.794326338	0.5989	2.996039	0.0027351	0.008206619	up-regulated	protein_coding

HIP14L	ENSMMUG00000007041	58.78979041	-1.725651415	0.576299	-2.99437	0.0027502	0.008247568	down-regulated	protein_coding
TPD52	ENSMMUG00000022369	200.4687183	-1.168601796	0.390259	-2.99442	0.0027496	0.008247568	down-regulated	protein_coding
MED4	ENSMMUG00000017988	53.76772513	-1.76575515	0.589805	-2.9938	0.0027553	0.008259855	down-regulated	protein_coding
MPI	ENSMMUG00000005227	77.97705853	1.463032395	0.488777	2.993253	0.0027602	0.008272435	up-regulated	protein_coding
ADAT3	ENSMMUG00000011928	15.29438383	-4.302137977	1.437494	-2.9928	0.0027643	0.008282868	down-regulated	protein_coding
UFSP1	ENSMMUG00000021256	13.36869928	-4.107760668	1.372582	-2.99272	0.002765	0.008283272	down-regulated	protein_coding
MESDC1	ENSMMUG00000009410	146.6176654	-1.254085021	0.41907	-2.99254	0.0027666	0.00828645	down-regulated	protein_coding
ZNF326	ENSMMUG00000003442	35.06081448	-3.005606664	1.004395	-2.99245	0.0027674	0.008287101	down-regulated	protein_coding
RSAD1	ENSMMUG00000017240	13.146417	-4.084491252	1.365025	-2.99225	0.0027693	0.008289208	down-regulated	protein_coding
ZNF800	ENSMMUG00000002428	33.2472174	-2.806574496	0.938174	-2.99153	0.0027758	0.008306966	down-regulated	protein_coding
TMEM105	ENSMMUG00000007241	11.2867607	-3.866121945	1.292501	-2.9912	0.0027789	0.00831431	down-regulated	protein_coding
RBM5	ENSMMUG00000004921	135.2822346	-1.217699057	0.407187	-2.99052	0.0027851	0.008331076	down-regulated	protein_coding
NUP205	ENSMMUG00000016384	126.3825212	1.154177012	0.386017	2.989962	0.0027901	0.008344416	up-regulated	protein_coding
RTEL1	ENSMMUG00000000167	164.7467916	1.789735713	0.5986	2.989868	0.002791	0.00834522	up-regulated	protein_coding
PARS2	ENSMMUG00000032084	12.24291043	-3.983131816	1.332544	-2.98912	0.0027978	0.008363939	down-regulated	protein_coding
GRIPAP1	ENSMMUG00000020059	61.08023523	-1.463754291	0.489758	-2.98873	0.0028014	0.00837279	down-regulated	protein_coding
TRPV4	ENSMMUG00000003942	8.853882459	3.287775361	1.100241	2.988232	0.002806	0.008384707	up-regulated	protein_coding
BTBD11	ENSMMUG00000015540	32.27361176	2.635201605	0.882477	2.986141	0.0028252	0.00844048	up-regulated	protein_coding
BHLHA15	ENSMMUG00000015478	8.709681387	3.319451647	1.11167	2.986004	0.0028265	0.00844069	up-regulated	protein_coding
TBC1D8B	ENSMMUG00000021413	30.19625768	-4.249820001	1.423229	-2.98604	0.0028262	0.00844069	down-regulated	protein_coding
TIE1	ENSMMUG00000010660	8.349897791	2.867033767	0.960318	2.985504	0.0028311	0.008452516	up-regulated	protein_coding
RRM2B	ENSMMUG00000020765	41.1271999	-2.768166354	0.92722	-2.98545	0.0028316	0.008452516	down-regulated	protein_coding
NIPBL	ENSMMUG00000022154	97.75633375	-1.586892343	0.531638	-2.98491	0.0028366	0.008465492	down-regulated	protein_coding
ESRP1	ENSMMUG00000021112	59.75571584	-1.607311215	0.53885	-2.98285	0.0028558	0.008517322	down-regulated	protein_coding
PSTPIP2	ENSMMUG00000003566	14.23026908	-4.201594751	1.408744	-2.98251	0.0028589	0.008525024	down-regulated	protein_coding
CCDC148	ENSMMUG00000011637	11.08439076	5.054588566	1.695191	2.981722	0.0028663	0.008543515	up-regulated	protein_coding
DCUN1D3	ENSMMUG00000016746	64.49549769	1.551648849	0.520486	2.981154	0.0028716	0.008555685	up-regulated	protein_coding
LRP1B	ENSMMUG00000009885	31.08254678	10.85247235	3.640707	2.980869	0.0028743	0.008561821	up-regulated	protein_coding
ST3GAL5	ENSMMUG00000019559	31.60834835	-2.778851569	0.932283	-2.98069	0.002876	0.008564926	down-regulated	protein_coding
OTUB2	ENSMMUG00000007762	11.6845507	2.298714524	0.771236	2.980558	0.0028772	0.008566914	up-regulated	protein_coding
ZNF611	ENSMMUG00000006472	11.9790178	-3.951284228	1.325802	-2.9803	0.0028797	0.008572423	down-regulated	protein_coding
ZNF473	ENSMMUG00000010838	32.24879	1.582027738	0.530909	2.979845	0.0028839	0.00858328	up-regulated	protein_coding
FSD1L	ENSMMUG00000002665	50.15611105	-2.521128423	0.846153	-2.97952	0.002887	0.008590619	down-regulated	protein_coding
RABGAP1	ENSMMUG00000000670	39.54514471	-2.341429146	0.786234	-2.97803	0.0029011	0.008627865	down-regulated	protein_coding
C10ORF12	ENSMMUG00000010427	32.42301178	1.981878289	0.665507	2.978	0.0029014	0.008627865	up-regulated	protein_coding
ADAMTS9	ENSMMUG00000000265	93.31430724	1.599442474	0.537128	2.977766	0.0029036	0.008631458	up-regulated	protein_coding
FAAH	ENSMMUG00000002714	180.5227239	-1.332145551	0.447545	-2.97656	0.002915	0.008661169	down-regulated	protein_coding
TP53I3	ENSMMUG00000001283	129.9549555	1.347560388	0.452866	2.975624	0.0029239	0.008685847	up-regulated	protein_coding
ZDHHC17	ENSMMUG00000018254	48.14971699	1.335638175	0.448976	2.974855	0.0029313	0.008705818	up-regulated	protein_coding
ALG14	ENSMMUG00000023641	21.17900515	-3.746689214	1.259739	-2.97418	0.0029377	0.008723227	down-regulated	protein_coding
COIL	ENSMMUG00000009280	21.16919573	-3.751621887	1.261886	-2.97303	0.0029488	0.008754159	down-regulated	protein_coding
DNASE2B	ENSMMUG00000000406	10.88926096	5.975750033	2.010379	2.972449	0.0029543	0.008768832	up-regulated	protein_coding
RILPL1	ENSMMUG00000008416	38.31232916	2.11405975	0.711269	2.972235	0.0029564	0.008773105	up-regulated	protein_coding
ABHD8	ENSMMUG00000009656	31.50564938	-2.864792178	0.964051	-2.97162	0.0029624	0.008788915	down-regulated	protein_coding
TP53I13	ENSMMUG00000003866	53.51659108	-2.300258695	0.774303	-2.97075	0.0029708	0.008811991	down-regulated	protein_coding
PCF11	ENSMMUG00000005029	47.71486914	-1.820502601	0.61364	-2.96673	0.0030099	0.008924264	down-regulated	protein_coding
SEPN1	ENSMMUG00000007851	48.75764115	1.66837939	0.56246	2.966219	0.0030149	0.008937185	up-regulated	protein_coding
ZNF232	ENSMMUG00000004779	35.87354862	2.118592788	0.714369	2.965684	0.0030201	0.008947118	up-regulated	protein_coding
MANBAL	ENSMMUG00000012536	33.52621253	-2.945535279	0.993195	-2.96572	0.0030198	0.008947118	down-regulated	protein_coding
ANKRD9	ENSMMUG00000016889	45.00277069	-3.018336837	1.017725	-2.96577	0.0030193	0.008947118	down-regulated	protein_coding
SSH3	ENSMMUG00000015932	169.4602024	-1.223114219	0.412472	-2.96533	0.0030236	0.008953811	down-regulated	protein_coding
THOC5	ENSMMUG00000023552	80.87431024	-1.918979082	0.647132	-2.96536	0.0030233	0.008953811	down-regulated	protein_coding
POLG	ENSMMUG00000011160	396.8202975	1.136219495	0.383214	2.964975	0.0030271	0.008962157	up-regulated	protein_coding
MTPAP	ENSMMUG00000003001	29.52538511	-2.725829447	0.919418	-2.96474	0.0030294	0.008967261	down-regulated	protein_coding
PON3	ENSMMUG00000008013	38.43614168	-3.803436173	1.282984	-2.96452	0.0030315	0.008970946	down-regulated	protein_coding
CNKSR1	ENSMMUG00000032214	228.447498	-1.074305701	0.362393	-2.96448	0.003032	0.008970946	down-regulated	protein_coding
MAMU-DPA1	ENSMMUG00000019371	52.09766309	3.758922955	1.268052	2.964329	0.0030334	0.008973457	up-regulated	protein_coding
DCTN2	ENSMMUG00000011292	217.4639653	-1.102947008	0.37214	-2.96379	0.0030387	0.008987227	down-regulated	protein_coding
ENC1	ENSMMUG00000019996	201.2522923	-1.026358261	0.3464	-2.96293	0.0030473	0.009010597	down-regulated	protein_coding
SLC22A13	ENSMMUG00000006480	10.41871864	5.20457292	1.756917	2.962333	0.0030532	0.009026188	up-regulated	protein_coding
SLC25A33	ENSMMUG00000001958	12.20290233	-3.975889356	1.342234	-2.96214	0.003055	0.009029845	down-regulated	protein_coding
ZNF770	ENSMMUG00000029777	37.22670239	-3.75332517	1.26735	-2.96155	0.0030609	0.009043402	down-regulated	protein_coding
TMPRSS3	ENSMMUG00000018079	7.594094988	5.124525344	1.730491	2.961312	0.0030633	0.009048597	up-regulated	protein_coding
TMPRSS11E	ENSMMUG00000011103	7.381860006	6.910733083	2.334304	2.960512	0.0030713	0.009064584	up-regulated	protein_coding
GRM1	ENSMMUG00000016428	7.381860006	6.910733083	2.334304	2.960512	0.0030713	0.009064584	up-regulated	protein_coding
PSMD3	ENSMMUG00000020382	217.0053508	-1.294939232	0.437547	-2.95954	0.003081	0.009090567	down-regulated	protein_coding
ADAM10	ENSMMUG00000017507	105.7156219	-2.311459599	0.781378	-2.95818	0.0030946	0.009127599	down-regulated	protein_coding
SOAT1	ENSMMUG00000003768	28.84102068	-3.343478184	1.130666	-2.95709	0.0031056	0.009156329	down-regulated	protein_coding
C19orf60	ENSMMUG00000000159	38.58796997	-3.162149637	1.069817	-2.95579	0.0031187	0.009191972	down-regulated	protein_coding
ENGASE	ENSMMUG00000023027	42.54589931	-2.199287322	0.744068	-2.95576	0.003119	0.009191972	down-regulated	protein_coding
CREBBP	ENSMMUG00000002286	193.2476484	1.054825746	0.356961	2.955019	0.0031265	0.009212164	up-regulated	protein_coding
MPDZ	ENSMMUG00000019388	30.36872871	1.502067734	0.508338	2.954862	0.0031281	0.009213012	up-regulated	protein_coding
C16orf13	ENSMMUG00000021968	97.71223494	-1.940508478	0.656704	-2.95492	0.0031275	0.009213012	down-regulated	protein_coding
HSPA13	ENSMMUG00000020091	21.62571066	-3.73796194	1.265399	-2.95398	0.0031371	0.009237512	down-regulated	protein_coding
CMTM1	ENSMMUG00000029108	5.543609061	4.010186149	1.357616	2.953843	0.0031384	0.009239632	up-regulated	protein_coding
KTI12	ENSMMUG00000008822	11.20351833	-3.85429681	1.305218	-2.95299	0.0031471	0.009261318	down-regulated	protein_coding
TNFSF13B	ENSMMUG00000008040	6.124923615	4.947083724	1.675843	2.951997	0.0031573	0.009289264	up-regulated	protein_coding
TNNT3	ENSMMUG00000016589	7.247934402	5.216568558	1.76803	2.950497	0.0031726	0.009332541	up-regulated	protein_coding
HNF4A	ENSMMUG00000006464	599.9591783	1.53472831	0.520176	2.950401	0.0031736	0.009333515	up-regulated	protein_coding
ABCB10	ENSMMUG00000022094	47.68220311	-2.089531503	0.708272	-2.95018	0.0031759	0.009338168	down-regulated	protein_coding
NMD3	ENSMMUG00000009564	32.83446656	-2.448056704	0.829841	-2.95003	0.0031774	0.009340854	down-regulated	protein_coding
HNRNPM	ENSMMUG00000018806	273.090137	-1.640170425	0.556106	-2.94938	0.0031841	0.009358471	down-regulated	protein_coding
QARS	ENSMMUG00000002943	393.1489191	-1.024905251	0.34754	-2.94903	0.0031877	0.009367222	down-regulated	protein_coding
MBIP	ENSMMUG00000004856	11.36937669	-3.874819555	1.314004	-2.94886	0.0031894	0.009368471	down-regulated	protein_coding
SEPHS1	ENSMMUG00000021233	52.25851702	-2.099112303	0.711838	-2.94886	0.0031895	0.009368471	down-regulated	protein_coding
USP9X	ENSMMUG00000013753	618.7392442	1.181952378	0.400929	2.948031	0.0031981	0.009391701	up-regulated	protein_coding
PARD3	ENSMMUG00000019156	72.6042077	-1.535671299	0.520982	-2.94765	0.003202	0.009401438	down-regulated	protein_coding
ECI1	ENSMMUG00000019237	53.40510224	-2.599287756	0.881913	-2.94733	0.0032053	0.009409112	down-regulated	protein_coding
EPC1	ENSMMUG00000012451	11.86481674	-3.939132565	1.336881	-2.94651	0.0032138	0.009432139	down-regulated	protein_coding
NAGA	ENSMMUG00000000005	138.6029322	-1.426486544	0.484152	-2.94636	0.0032154	0.009434675	down-regulated	protein_coding
USP19	ENSMMUG00000002948	181.8969938	-1.02747737	0.348787	-2.94586	0.0032206	0.009448168	down-regulated	protein_coding
OSBPL8	ENSMMUG00000017864	58.90138448	-1.975639266	0.670988	-2.94437	0.0032361	0.009491582	down-regulated	protein_coding
ZHX1	ENSMMUG00000006050	81.14004837	-1.948404146	0.661753	-2.94431	0.0032368	0.00949167	down-regulated	protein_coding
CEBPG	ENSMMUG00000006127	132.1036256	1.333586901	0.453	2.943903	0.003241	0.009500132	up-regulated	protein_coding
ACOT9	ENSMMUG00000018824	82.66754806	-1.408638353	0.478486	-2.94395	0.0032405	0.009500132	down-regulated	protein_coding
PRKCSH	ENSMMUG00000002328	327.355373	-1.516703835	0.515402	-2.94276	0.003253	0.009531372	down-regulated	protein_coding
RCHY1	ENSMMUG00000009177	10.72482272	-3.791923425	1.288767	-2.94229	0.003258	0.009543874	down-regulated	protein_coding
CWF19L1	ENSMMUG00000018472	20.9464534	-3.730106297	1.268028	-2.94166	0.0032646	0.009561312	down-regulated	protein_coding
MYOM1	ENSMMUG00000019892	11.30825421	2.408763744	0.818971	2.941207	0.0032694	0.009573302	up-regulated	protein_coding
PNPT1	ENSMMUG00000007003	61.02348513	-1.988562585	0.67612	-2.94114	0.0032701	0.009573433	down-regulated	protein_coding

ATP2B1	ENSMMUG00000023378	297.5078833	-1.65577291	0.563067	-2.94063	0.0032754	0.009586468	down-regulated	protein_coding
CBLC	ENSMMUG00000003271	449.5901049	1.159667854	0.394417	2.940207	0.0032799	0.009596308	up-regulated	protein_coding
SEC23B	ENSMMUG00000012878	81.19655463	-1.578795355	0.537179	-2.93905	0.0032922	0.009629016	down-regulated	protein_coding
LINC00116	ENSMMUG00000022800	15.7533421	-4.344540681	1.478225	-2.93902	0.0032925	0.009629016	down-regulated	protein_coding
MYD88	ENSMMUG00000000995	40.98738624	-2.198553132	0.748565	-2.93702	0.0033138	0.009689322	down-regulated	protein_coding
ANKRD12	ENSMMUG00000022097	81.4738354	-1.650947814	0.56217	-2.93674	0.0033168	0.009696101	down-regulated	protein_coding
TSPYL2	ENSMMUG00000002593	11.44959497	-3.88470256	1.322936	-2.93643	0.0033202	0.009704042	down-regulated	protein_coding
POU2AF1	ENSMMUG00000003244	9.51059078	5.015965009	1.708642	2.935645	0.0033286	0.009726494	up-regulated	protein_coding
MNAT1	ENSMMUG00000001642	21.38315513	-3.717846539	1.266562	-2.93538	0.0033314	0.009732682	down-regulated	protein_coding
COPRS	ENSMMUG00000020401	13.12988064	-4.081311246	1.390486	-2.93517	0.0033337	0.009737394	down-regulated	protein_coding
DDX19B	ENSMMUG00000020316	65.06035698	-1.679098001	0.572169	-2.93462	0.0033396	0.009752733	down-regulated	protein_coding
SLC25A20	ENSMMUG00000015418	31.29580791	-3.447054208	1.174855	-2.93403	0.003346	0.00976929	down-regulated	protein_coding
MSI2	ENSMMUG00000002069	28.87620063	-3.348004215	1.141191	-2.93378	0.0033486	0.009775028	down-regulated	protein_coding
SACM1L	ENSMMUG00000002130	87.68382121	-1.553312281	0.529479	-2.93366	0.0033499	0.009776775	down-regulated	protein_coding
ZDHHC4	ENSMMUG00000015545	42.81477006	-2.085604042	0.711088	-2.93298	0.0033573	0.009796355	down-regulated	protein_coding
HIST1H4K	ENSMMUG00000029897	15.0708773	2.872531657	0.979829	2.931667	0.0033715	0.009835699	up-regulated	protein_coding
RPF1	ENSMMUG00000022181	43.49802754	-2.219812608	0.757218	-2.93154	0.0033729	0.009837777	down-regulated	protein_coding
PLA2G16	ENSMMUG00000005775	22.30789561	-3.830918899	1.306854	-2.93141	0.0033743	0.009839883	down-regulated	protein_coding
NOX1	ENSMMUG00000004178	7.623154141	4.906692817	1.674518	2.930212	0.0033873	0.009875738	up-regulated	protein_coding
USMG5	ENSMMUG00000014037	84.4167674	-2.392866285	0.816662	-2.93006	0.003389	0.009878654	down-regulated	protein_coding
RDH14	ENSMMUG00000014083	27.56550253	-3.198091114	1.091522	-2.92994	0.0033903	0.009880387	down-regulated	protein_coding
TMEM192	ENSMMUG00000021912	20.03367047	-3.670765011	1.253214	-2.92908	0.0033997	0.00990563	down-regulated	protein_coding
FSCN1	ENSMMUG00000004282	71.63185643	-2.634977192	0.899715	-2.92868	0.003404	0.009916372	down-regulated	protein_coding
KIAA1211	ENSMMUG00000016358	152.2909749	1.904624286	0.650399	2.928392	0.0034072	0.009923541	up-regulated	protein_coding
KRTCAP2	ENSMMUG00000012180	82.13832121	-2.08949132	0.713595	-2.92812	0.0034102	0.00992818	down-regulated	protein_coding
ZBTB9	ENSMMUG00000029765	11.43709947	-3.883561844	1.32646	-2.92776	0.0034141	0.009937429	down-regulated	protein_coding
CXCL10	ENSMMUG00000020903	10.60704815	4.561309829	1.558963	2.925862	0.003435	0.009996351	up-regulated	protein_coding
SUPT16H	ENSMMUG00000015000	66.47973312	-1.677008709	0.573248	-2.92545	0.0034396	0.010003452	down-regulated	protein_coding
TESK2	ENSMMUG00000020083	67.79606587	1.440720748	0.49246	2.92556	0.0034384	0.010003452	up-regulated	protein_coding
SURF1	ENSMMUG00000022344	106.0907126	-1.902572298	0.650349	-2.92547	0.0034394	0.010003452	down-regulated	protein_coding
DSCAM	ENSMMUG00000005174	25.22395218	8.20594461	2.805656	2.924787	0.0034469	0.010022718	up-regulated	protein_coding
ARPC5	ENSMMUG00000031302	71.44573194	-1.868136743	0.638895	-2.92401	0.0034555	0.010045697	down-regulated	protein_coding
NSMCE1	ENSMMUG00000015832	49.43306502	-2.619788955	0.895998	-2.92388	0.003457	0.010047878	down-regulated	protein_coding
TUBB6	ENSMMUG00000032572	246.5086016	1.181037538	0.404022	2.923199	0.0034646	0.010067762	up-regulated	protein_coding
SH2B3	ENSMMUG00000002223	57.70796062	1.821744707	0.623295	2.922763	0.0034694	0.010079785	up-regulated	protein_coding
NCBP2	ENSMMUG00000007825	134.785503	-1.58307797	0.541813	-2.92182	0.00348	0.01010244	down-regulated	protein_coding
DDX28	ENSMMUG00000010836	12.1823141	-3.97390118	1.360023	-2.92194	0.0034786	0.01010244	down-regulated	protein_coding
TMEM180	ENSMMUG00000011948	30.01189749	1.632964405	0.558888	2.921808	0.0034801	0.01010244	up-regulated	protein_coding
IGFL1	ENSMMUG00000020229	98.91561086	-6.997172852	2.394717	-2.92192	0.0034788	0.01010244	down-regulated	protein_coding
FKBP15	ENSMMUG00000000173	99.3562897	-1.203495661	0.412041	-2.92081	0.0034912	0.010132697	down-regulated	protein_coding
SHC1	ENSMMUG00000006581	166.6960808	-1.271138099	0.435258	-2.92043	0.0034955	0.01014324	down-regulated	protein_coding
PRDM15	ENSMMUG00000018046	30.49723031	1.610331918	0.551477	2.920037	0.0034999	0.010153796	up-regulated	protein_coding
PFDN4	ENSMMUG00000017300	13.03561957	-4.071140505	1.39458	-2.91926	0.0035087	0.010177101	down-regulated	protein_coding
BICD2	ENSMMUG00000001061	77.45439723	1.072975742	0.367666	2.918347	0.0035189	0.010202728	up-regulated	protein_coding
HIST1H2AC	ENSMMUG00000029923	149.6829	1.705841391	0.584543	2.918249	0.00352	0.010203824	up-regulated	protein_coding
TPBG	ENSMMUG00000005196	13.09499761	-4.078464133	1.397783	-2.91781	0.003525	0.010215872	down-regulated	protein_coding
GLUL	ENSMMUG00000022884	104.0354765	-1.323493251	0.453797	-2.91649	0.00354	0.010255293	down-regulated	protein_coding
TGFA	ENSMMUG00000010591	14.54393944	-4.233013178	1.451554	-2.9162	0.0035433	0.010262845	down-regulated	protein_coding
ABCF2	ENSMMUG00000006679	37.23885203	-2.265597559	0.777143	-2.91529	0.0035536	0.010290543	down-regulated	protein_coding
PPP1R11	ENSMMUG00000012122	96.6436647	-1.14601994	0.393143	-2.91502	0.0035567	0.010297359	down-regulated	protein_coding
FBXO22	ENSMMUG00000009161	40.10600519	-2.352299509	0.807375	-2.91351	0.0035738	0.010342884	down-regulated	protein_coding
NUP188	ENSMMUG00000011757	215.0446338	1.079695176	0.370575	2.913567	0.0035733	0.010342884	up-regulated	protein_coding
DKK3	ENSMMUG00000022862	13.89341324	5.152135303	1.769136	2.912233	0.0035886	0.010383317	up-regulated	protein_coding
GEM	ENSMMUG00000019759	12.78048431	-4.044953305	1.389541	-2.911	0.0036027	0.010422236	down-regulated	protein_coding
AGPS	ENSMMUG00000008502	35.33414218	-3.066342802	1.053669	-2.91016	0.0036125	0.010448222	down-regulated	protein_coding
INVS	ENSMMUG00000001524	39.39522083	1.474743603	0.507108	2.908144	0.0036358	0.010511467	up-regulated	protein_coding
NAA15	ENSMMUG00000018184	63.55078051	-1.751419953	0.602244	-2.90816	0.0036356	0.010511467	down-regulated	protein_coding
MNP2	ENSMMUG00000017957	6.989694513	4.979204263	1.712508	2.90755	0.0036427	0.010529272	up-regulated	protein_coding
MFHAS1	ENSMMUG00000016708	148.0780166	1.454445593	0.500304	2.907126	0.0036477	0.010541424	up-regulated	protein_coding
CD2AP	ENSMMUG00000017952	319.2801847	-1.975283801	0.679887	-2.90531	0.0036689	0.01060059	down-regulated	protein_coding
MRPS33	ENSMMUG00000008277	34.54647701	-2.908420735	1.001197	-2.90494	0.0036732	0.010610827	down-regulated	protein_coding
ASB13	ENSMMUG00000001290	19.49188048	-3.62931635	1.24958	-2.90443	0.0036792	0.010626121	down-regulated	protein_coding
NR0B2	ENSMMUG00000010291	35.85619589	-2.822165191	0.971842	-2.90394	0.003685	0.010640756	down-regulated	protein_coding
ATP5H	ENSMMUG00000011647	241.9290414	-1.395414938	0.480641	-2.90324	0.0036932	0.010662269	down-regulated	protein_coding
ASTN2	ENSMMUG00000013105	6.152570787	3.215827055	1.107763	2.902991	0.0036962	0.010668539	up-regulated	protein_coding
ZNF562	ENSMMUG00000020452	10.60468583	-3.774980009	1.300447	-2.90283	0.003698	0.010671771	down-regulated	protein_coding
IL8	ENSMMUG00000003836	33.18277961	-3.538482247	1.219339	-2.90197	0.0037083	0.010699053	down-regulated	protein_coding
SNTG1	ENSMMUG00000002811	4.428915182	5.046729145	1.739364	2.90148	0.003714	0.010713573	up-regulated	protein_coding
ADSS	ENSMMUG00000000016	60.21491744	-1.657169688	0.57128	-2.9008	0.0037221	0.010734668	down-regulated	protein_coding
CBX4	ENSMMUG00000016418	37.53714662	-2.25242642	0.776618	-2.9003	0.003728	0.010749534	down-regulated	protein_coding
ACSF3	ENSMMUG00000023245	21.58767077	-3.775494087	1.301929	-2.89992	0.0037325	0.010760347	down-regulated	protein_coding
COL6A3	ENSMMUG00000014049	17.89900476	2.819689946	0.972369	2.899814	0.0037338	0.01076189	up-regulated	protein_coding
CNTNAP2	ENSMMUG00000015772	94.2698005	1.320870354	0.455559	2.899448	0.0037382	0.010772272	up-regulated	protein_coding
AADAC	ENSMMUG00000001664	15.58064293	-4.331974274	1.494148	-2.89929	0.00374	0.01077535	down-regulated	protein_coding
ZNF410	ENSMMUG00000011144	44.29278406	-1.993620059	0.687689	-2.89902	0.0037434	0.010782752	down-regulated	protein_coding
NFAT5	ENSMMUG00000018255	65.72587608	1.321636972	0.455913	2.898877	0.003745	0.010785311	up-regulated	protein_coding
C6orf223	ENSMMUG00000011364	200.6071354	-1.690649433	0.583246	-2.89869	0.0037472	0.010789524	down-regulated	protein_coding
TADA2A	ENSMMUG00000021911	46.73979762	1.597287948	0.551104	2.89834	0.0037514	0.010799418	up-regulated	protein_coding
ATAT1	ENSMMUG00000006526	40.68870388	1.769592198	0.610586	2.898187	0.0037533	0.010800282	up-regulated	protein_coding
ZNF83	ENSMMUG00000023272	33.51200626	-2.126222513	0.73363	-2.89822	0.0037529	0.010800282	down-regulated	protein_coding
AVL9	ENSMMUG00000011770	106.2690361	1.547788618	0.534151	2.89766	0.0037596	0.010816228	up-regulated	protein_coding
LINS	ENSMMUG00000016503	10.58999461	-3.772364912	1.302776	-2.89564	0.0037839	0.010884003	down-regulated	protein_coding
FUBP3	ENSMMUG00000008249	64.41764477	-1.767226707	0.61068	-2.89387	0.0038053	0.010943287	down-regulated	protein_coding
ACSS3	ENSMMUG00000013800	13.35083813	-4.108260407	1.420162	-2.89281	0.0038181	0.010977951	down-regulated	protein_coding
DUSP12	ENSMMUG00000004047	22.37299091	-3.83464025	1.32566	-2.89263	0.0038204	0.010982166	down-regulated	protein_coding
SLC16A11	ENSMMUG00000021176	6.485427357	3.07966046	1.064697	2.892524	0.0038216	0.010983524	up-regulated	protein_coding
CASP7	ENSMMUG00000001217	450.2739689	-1.995959413	0.690107	-2.89225	0.003825	0.010988722	down-regulated	protein_coding
FOXRED1	ENSMMUG00000007944	83.31664448	-2.006948101	0.693904	-2.89226	0.0038248	0.010988722	down-regulated	protein_coding
TTR	ENSMMUG00000016673	82.84026992	-2.114959738	0.73155	-2.89107	0.0038394	0.011027123	down-regulated	protein_coding
GPR160	ENSMMUG00000022196	108.8283137	-1.464892294	0.506704	-2.89102	0.0038399	0.011027123	down-regulated	protein_coding
SLC38A2	ENSMMUG00000022359	77.67843834	-2.45433518	0.849068	-2.89062	0.0038448	0.011038911	down-regulated	protein_coding
CDCP1	ENSMMUG00000005742	99.60175725	-1.506287095	0.521132	-2.89042	0.0038473	0.011043977	down-regulated	protein_coding
DENND6A	ENSMMUG00000018619	72.26293023	-1.566438133	0.542633	-2.88674	0.0038926	0.011167181	down-regulated	protein_coding
GTDC1	ENSMMUG00000011922	10.53733528	-3.764909217	1.30434	-2.88645	0.0038962	0.011172842	down-regulated	protein_coding
APRT	ENSMMUG00000013824	289.1317447	-1.857797874	0.643665	-2.88628	0.0038983	0.011176603	down-regulated	protein_coding
ZNF689	ENSMMUG00000001121	13.4890499	2.295352479	0.795344	2.885987	0.0039019	0.011184725	up-regulated	protein_coding
NUB1	ENSMMUG00000006244	39.42134568	-2.087241976	0.723381	-2.8854	0.0039092	0.011202691	down-regulated	protein_coding
LRRC42	ENSMMUG00000007182	63.47651401	-1.60614924	0.556656	-2.88535	0.0039097	0.011202691	down-regulated	protein_coding
ZNF57	ENSMMUG00000018682	36.42707282	-2.204573761	0.764117	-2.88513	0.0039126	0.0112085	down-regulated	protein_coding
AKT2	ENSMMUG00000003621	76.1188811	-1.415964541	0.490795	-2.88505	0.0039136	0.011209133	down-regulated	protein_coding

SEZ6L2	ENSMMUG00000018986	295.5931027	-1.42986829	0.495682	-2.88465	0.0039185	0.011221029	down-regulated	protein_coding
C18ORF10	ENSMMUG00000006863	101.2385794	1.041211344	0.36099	2.884323	0.0039226	0.011230341	up-regulated	protein_coding
C11H12ORF57	ENSMMUG00000010202	113.4890742	-2.203500698	0.764	-2.88416	0.0039245	0.011233724	down-regulated	protein_coding
FBXL14	ENSMMUG00000019440	19.3282335	-3.61089825	1.25257	-2.88279	0.0039417	0.011278202	down-regulated	protein_coding
YTHDF2	ENSMMUG00000011391	109.0567372	-1.523353336	0.528634	-2.88168	0.0039556	0.011315825	down-regulated	protein_coding
MPV17	ENSMMUG00000010511	26.00573667	-3.156399934	1.095557	-2.88109	0.003963	0.011334635	down-regulated	protein_coding
KCNN4	ENSMMUG00000021267	11.35996419	-3.873612885	1.344658	-2.88074	0.0039674	0.011344928	down-regulated	protein_coding
ZFP30	ENSMMUG00000001933	10.83477203	-3.807417821	1.322223	-2.87956	0.0039823	0.011383025	down-regulated	protein_coding
HMGN2	ENSMMUG00000010443	253.4145778	-2.086288815	0.724508	-2.8796	0.0039819	0.011383025	down-regulated	protein_coding
U5	ENSMMUG00000024178	2.794896609	4.192349475	1.456035	2.879292	0.0039857	0.011390326	up-regulated	snRNA
CCDC23	ENSMMUG00000012401	14.41298083	-4.217086236	1.464667	-2.87921	0.0039867	0.011390949	down-regulated	protein_coding
PET117	ENSMMUG00000023140	36.37448834	-2.283570022	0.793467	-2.87796	0.0040025	0.011431485	down-regulated	protein_coding
ATRAID	ENSMMUG00000010263	58.34898103	-2.034992851	0.707223	-2.87744	0.0040091	0.011448029	down-regulated	protein_coding
DNALI1	ENSMMUG00000008418	6.193450865	3.040856823	1.056957	2.876991	0.0040149	0.011462148	up-regulated	protein_coding
GLRA1	ENSMMUG00000014704	21.07450127	10.29880408	3.581471	2.875579	0.0040329	0.011504256	up-regulated	protein_coding
FAM134C	ENSMMUG00000016796	82.10203483	1.293765432	0.45003	2.874844	0.0040423	0.011528755	up-regulated	protein_coding
POLR2K	ENSMMUG00000004385	10.70245064	-3.789781016	1.318404	-2.87452	0.0040464	0.011538177	down-regulated	protein_coding
DZIP1L	ENSMMUG00000013269	6.974894284	3.172398415	1.10371	2.874304	0.0040492	0.011543829	up-regulated	protein_coding
POLDIP2	ENSMMUG00000019146	157.2878915	-1.271991999	0.442588	-2.87399	0.0040532	0.011552996	down-regulated	protein_coding
CCBL2	ENSMMUG00000018795	40.25392899	-2.031058561	0.706803	-2.87359	0.0040584	0.0115654	down-regulated	protein_coding
CCNG2	ENSMMUG00000000914	52.22924576	-2.331925687	0.81177	-2.87264	0.0040705	0.01159569	down-regulated	protein_coding
CCDC43	ENSMMUG00000015049	34.47845255	-2.110547306	0.734741	-2.87251	0.0040723	0.01159569	down-regulated	protein_coding
SLC24A1	ENSMMUG00000022059	10.10839612	2.982585899	1.038322	2.872507	0.0040723	0.01159569	up-regulated	protein_coding
SLC26A3	ENSMMUG00000017618	162.1991136	-1.98392938	0.69069	-2.87239	0.0040739	0.011597785	down-regulated	protein_coding
NUDT16L1	ENSMMUG00000001890	45.52084864	-2.340062292	0.814757	-2.8721	0.0040776	0.011606032	down-regulated	protein_coding
GPC4	ENSMMUG00000003456	43.54730162	-2.305240948	0.80272	-2.87179	0.0040816	0.011615109	down-regulated	protein_coding
PTCRA	ENSMMUG00000000460	4.558172407	4.755934103	1.6565	2.871075	0.0040908	0.011638958	up-regulated	protein_coding
RC3H2	ENSMMUG00000021134	107.9234187	1.638409148	0.570715	2.870799	0.0040944	0.01164677	up-regulated	protein_coding
SVIP	ENSMMUG00000020820	11.43431116	-3.885398072	1.353824	-2.86994	0.0041055	0.011676013	down-regulated	protein_coding
ZNF286A	ENSMMUG00000002354	15.24717488	2.328019133	0.81124	2.869705	0.0041085	0.011682446	up-regulated	protein_coding
FOXO4	ENSMMUG00000032429	68.04020971	1.658507442	0.578028	2.869252	0.0041144	0.011696838	up-regulated	protein_coding
RBM7	ENSMMUG00000012270	29.02447111	-2.702247419	0.94204	-2.86851	0.0041242	0.011722137	down-regulated	protein_coding
ADRA2A	ENSMMUG00000005316	90.001891	-1.614562181	0.562957	-2.868	0.0041307	0.011738452	down-regulated	protein_coding
ATP6V1H	ENSMMUG00000004064	76.05932447	-1.48434646	0.517596	-2.86777	0.0041337	0.011739883	down-regulated	protein_coding
ENKD1	ENSMMUG00000009808	13.58176407	-4.130820294	1.440402	-2.86782	0.004133	0.011739883	down-regulated	protein_coding
PPP2R5A	ENSMMUG00000019771	266.578118	-1.003877513	0.350049	-2.86782	0.0041331	0.011739883	down-regulated	protein_coding
NEDD1	ENSMMUG00000004093	19.57647887	-3.635833269	1.267989	-2.8674	0.0041386	0.011751335	down-regulated	protein_coding
UBE2G1	ENSMMUG00000005080	73.28145448	-1.691629574	0.589991	-2.86721	0.0041411	0.011755973	down-regulated	protein_coding
C20ORF20	ENSMMUG00000016855	11.73142733	-3.920266817	1.367341	-2.86707	0.0041429	0.011758792	down-regulated	protein_coding
CDC42BPG	ENSMMUG00000003971	240.7228906	-1.001924995	0.349481	-2.86689	0.0041453	0.011760847	down-regulated	protein_coding
NEURL1B	ENSMMUG00000008848	46.86305927	-3.477903129	1.213111	-2.86693	0.0041448	0.011760847	down-regulated	protein_coding
CTTNBP2	ENSMMUG00000010297	20.68525053	2.227802892	0.777179	2.866524	0.0041501	0.011772128	up-regulated	protein_coding
COMMD10	ENSMMUG00000008738	10.75900646	-3.794731885	1.323982	-2.86615	0.004155	0.01178289	down-regulated	protein_coding
THEM5	ENSMMUG00000014359	4.717711272	4.327590657	1.509919	2.866107	0.0041555	0.01178289	up-regulated	protein_coding
F2	ENSMMUG00000019674	8.84810296	4.178996475	1.458807	2.864668	0.0041745	0.011834219	up-regulated	protein_coding
NME4	ENSMMUG00000000612	161.7870255	-2.066449694	0.7217	-2.86331	0.0041924	0.011878009	down-regulated	protein_coding
PKD2	ENSMMUG00000008795	42.83250066	1.92961549	0.674306	2.861631	0.0042147	0.011938611	up-regulated	protein_coding
TBC1D17	ENSMMUG00000011626	304.2438165	1.572811597	0.549972	2.859803	0.004239	0.012005233	up-regulated	protein_coding
CASC5	ENSMMUG00000001288	20.3258824	1.77608413	0.621089	2.85963	0.0042413	0.012009376	up-regulated	protein_coding
NDEL1	ENSMMUG00000021938	64.91051971	-1.331133538	0.465503	-2.85956	0.0042423	0.01200971	down-regulated	protein_coding
ARFGEF1	ENSMMUG00000014424	121.4639752	-1.584080874	0.553973	-2.85949	0.0042432	0.012009938	down-regulated	protein_coding
SUV420H2	ENSMMUG00000003069	20.84006705	-3.723112995	1.302139	-2.85923	0.0042467	0.012017376	down-regulated	protein_coding
TPP2	ENSMMUG00000007247	45.18155822	-1.787743614	0.625361	-2.85874	0.0042533	0.012031081	down-regulated	protein_coding
RP9	ENSMMUG00000002884	32.2072368	-2.368605164	0.828621	-2.85849	0.0042566	0.012038212	down-regulated	protein_coding
C1H1ORF50	ENSMMUG00000016111	11.67256029	-3.913694812	1.369223	-2.85833	0.0042587	0.012041706	down-regulated	protein_coding
GRB2	ENSMMUG00000004756	99.4785951	-1.674589483	0.585995	-2.85768	0.0042675	0.012063984	down-regulated	protein_coding
SEPHS2	ENSMMUG00000020839	163.1065039	-1.152308514	0.403287	-2.85729	0.0042727	0.012076413	down-regulated	protein_coding
PROSAPIP1	ENSMMUG00000010028	250.9390725	-1.01344787	0.354889	-2.85567	0.0042946	0.012135758	down-regulated	protein_coding
SEPT1	ENSMMUG00000021842	14.67717703	2.368893811	0.829632	2.855355	0.0042989	0.012145532	up-regulated	protein_coding
CENPV	ENSMMUG00000004896	130.1955206	1.544858227	0.541068	2.855203	0.0043009	0.012146471	up-regulated	protein_coding
LAMB1	ENSMMUG00000006567	153.1380067	1.754446518	0.614462	2.855259	0.0043002	0.012146471	up-regulated	protein_coding
HNRNPA1	ENSMMUG00000012989	777.4170686	-1.575671965	0.551984	-2.85456	0.0043096	0.012168665	down-regulated	protein_coding
CENPM	ENSMMUG00000003360	43.69259152	-3.421311553	1.198656	-2.85429	0.0043133	0.012176609	down-regulated	protein_coding
LMAN1	ENSMMUG00000007580	99.15010562	-2.03726665	0.713835	-2.85397	0.0043176	0.012185858	down-regulated	protein_coding
GPR35	ENSMMUG00000015419	31.79129725	-2.853593103	1.000073	-2.85338	0.0043256	0.012204045	down-regulated	protein_coding
TMEM79	ENSMMUG00000000091	50.67993175	1.528985558	0.536031	2.852422	0.0043387	0.012238615	up-regulated	protein_coding
TXNDC15	ENSMMUG00000005284	61.53580571	1.335531038	0.468322	2.851739	0.0043481	0.012262513	up-regulated	protein_coding
ARPP19	ENSMMUG00000008344	42.65454391	-2.521844632	0.884365	-2.85159	0.0043501	0.012265845	down-regulated	protein_coding
ZNF460	ENSMMUG00000019285	22.01532044	1.797152658	0.630299	2.851269	0.0043545	0.012275742	up-regulated	protein_coding
GTPBP2	ENSMMUG00000008890	167.5940527	1.032368119	0.362105	2.851015	0.004358	0.012278207	up-regulated	protein_coding
SDF2L1	ENSMMUG00000018798	39.45828661	-3.253560246	1.141189	-2.85103	0.0043578	0.012278207	down-regulated	protein_coding
SCAMP1	ENSMMUG00000016398	12.58153409	-4.024212231	1.4117	-2.85061	0.0043635	0.012290137	down-regulated	protein_coding
GOLGA2	ENSMMUG00000022967	101.6781665	-1.548963978	0.543421	-2.85039	0.0043665	0.012294945	down-regulated	protein_coding
POMK	ENSMMUG00000021272	14.59630546	1.80285139	0.632641	2.849722	0.0043758	0.012318448	up-regulated	protein_coding
CNIH	ENSMMUG00000012325	102.4056069	-1.531354828	0.537502	-2.84902	0.0043854	0.012343096	down-regulated	protein_coding
BAZ2B	ENSMMUG00000006182	100.4465821	1.134805382	0.398429	2.848198	0.0043968	0.012372655	up-regulated	protein_coding
PI4KA	ENSMMUG00000022167	222.5819269	1.058517875	0.37176	2.847313	0.004409	0.012402173	up-regulated	protein_coding
VAMP4	ENSMMUG00000023203	9.983858203	-3.688639882	1.29601	-2.84615	0.0044251	0.012445035	down-regulated	protein_coding
SCN3A	ENSMMUG00000018840	7.555048546	4.327729603	1.520631	2.846009	0.0044271	0.01244814	up-regulated	protein_coding
HSD17B4	ENSMMUG00000017685	244.3444924	-1.077218739	0.378516	-2.8459	0.0044286	0.012449878	down-regulated	protein_coding
EML4	ENSMMUG00000016277	275.1123979	-1.050123605	0.369096	-2.84512	0.0044394	0.01247784	down-regulated	protein_coding
UTP20	ENSMMUG00000012891	55.44892361	1.189635185	0.418155	2.844965	0.0044416	0.012481539	up-regulated	protein_coding
HIVEP2	ENSMMUG00000007998	127.9198936	1.665837162	0.585625	2.844546	0.0044475	0.012495502	up-regulated	protein_coding
MFSD12	ENSMMUG00000010653	87.37204117	-1.874174955	0.658991	-2.84401	0.004455	0.012511744	down-regulated	protein_coding
DHCR7	ENSMMUG00000011976	229.8730877	-1.267392791	0.445636	-2.84401	0.004455	0.012511744	down-regulated	protein_coding
LTB4R	ENSMMUG00000020697	6.969409122	3.397998521	1.195029	2.843444	0.0044629	0.01253131	up-regulated	protein_coding
EME2	ENSMMUG00000018595	32.83368069	2.114231243	0.743676	2.842948	0.0044698	0.012548338	up-regulated	protein_coding
GLIS3	ENSMMUG00000012460	31.60215643	1.809388452	0.636519	2.84263	0.0044743	0.012558375	up-regulated	protein_coding
VTN	ENSMMUG00000019148	7.562078522	3.944467438	1.388064	2.841705	0.0044873	0.012591746	up-regulated	protein_coding
DYNLT1	ENSMMUG00000019454	83.47871749	-2.170209478	0.763712	-2.84166	0.004488	0.012591746	down-regulated	protein_coding
LEMD3	ENSMMUG00000016892	30.0946265	-2.799755788	0.985318	-2.84147	0.0044906	0.012596535	down-regulated	protein_coding
SYF2	ENSMMUG00000002968	40.96243576	-2.137291926	0.752449	-2.84045	0.004505	0.01263223	down-regulated	protein_coding
EDA2R	ENSMMUG00000015031	7.023974894	3.087421806	1.08695	2.840445	0.0045051	0.01263223	up-regulated	protein_coding
CACNA1I	ENSMMUG00000004878	21.93128465	10.35037873	3.644883	2.839701	0.0045156	0.012659186	up-regulated	protein_coding
KIFC2	ENSMMUG00000002661	98.78529993	-1.776102077	0.62547	-2.83963	0.0045166	0.012659638	down-regulated	protein_coding
SLCO2B1	ENSMMUG00000009126	45.93834046	-4.042847199	1.424934	-2.83722	0.0045509	0.012753017	down-regulated	protein_coding
C9orf156	ENSMMUG00000015654	10.34128322	-3.73966493	1.318233	-2.83688	0.0045557	0.012764152	down-regulated	protein_coding
ACER1	ENSMMUG00000005698	4.593495496	3.915172099	1.380365	2.836331	0.0045635	0.012783449	up-regulated	protein_coding
PRSS36	ENSMMUG00000002033	33.49283914	2.167530277	0.76425	2.836154	0.004566	0.012786718	up-regulated	protein_coding
PICK1	ENSMMUG00000002697	50.68058128	-2.200633725	0.77593	-2.83612	0.0045665	0.012786718	down-regulated	protein_coding

GBAS	ENSMMUG00000004679	31.8707594	-2.403855663	0.847701	-2.83573	0.004572	0.012799715	down-regulated	protein_coding
EDN3	ENSMMUG00000016131	49.70766871	2.290516869	0.807839	2.835362	0.0045774	0.012812143	up-regulated	protein_coding
MINPP1	ENSMMUG00000004766	104.2736396	-1.7584785	0.620236	-2.83518	0.00458	0.012817042	down-regulated	protein_coding
SLA/LP	ENSMMUG00000019784	9.955510415	-3.683782767	1.299793	-2.83413	0.0045951	0.012856548	down-regulated	protein_coding
UBXN4	ENSMMUG00000012248	113.5916413	-1.412777366	0.498537	-2.83385	0.0045991	0.012865316	down-regulated	protein_coding
EVI5L	ENSMMUG00000023303	58.48220038	1.447777714	0.510926	2.833632	0.0046022	0.012871491	up-regulated	protein_coding
CDC42BPB	ENSMMUG00000008380	231.7266249	-1.132487841	0.399685	-2.83345	0.0046048	0.012876211	down-regulated	protein_coding
SSX2IP	ENSMMUG00000006203	26.07153182	-3.163641636	1.11694	-2.83242	0.0046197	0.012915349	down-regulated	protein_coding
AKIRIN1	ENSMMUG00000009373	28.12172721	-2.662512161	0.940215	-2.83181	0.0046285	0.012937309	down-regulated	protein_coding
KCNJ13	ENSMMUG00000021831	124.2880096	2.223261196	0.785192	2.831486	0.0046332	0.01294794	up-regulated	protein_coding
WDR64	ENSMMUG00000008763	21.38073545	10.3198503	3.645186	2.83109	0.004639	0.012961444	up-regulated	protein_coding
GXYLT1	ENSMMUG00000010865	11.06936982	-3.839081096	1.356097	-2.83098	0.0046406	0.012963414	down-regulated	protein_coding
ALAD	ENSMMUG00000031965	25.21646818	-3.108233931	1.097979	-2.83087	0.0046422	0.012965281	down-regulated	protein_coding
ATRNL1	ENSMMUG00000001905	9.675279689	4.496609195	1.588903	2.830009	0.0046547	0.012992477	up-regulated	protein_coding
NINJ1	ENSMMUG00000014398	90.73472878	-1.615365693	0.57078	-2.8301	0.0046533	0.012992477	down-regulated	protein_coding
LPCAT4	ENSMMUG00000020939	204.3514543	-1.347131533	0.476016	-2.83001	0.0046546	0.012992477	down-regulated	protein_coding
MCTP2	ENSMMUG00000004787	32.26687107	1.618794514	0.572238	2.828882	0.0046711	0.013033168	up-regulated	protein_coding
P2RY2	ENSMMUG00000019186	63.11427109	1.016927159	0.359474	2.828933	0.0046703	0.013033168	up-regulated	protein_coding
REEP3	ENSMMUG00000016850	189.7859258	-1.675604981	0.592502	-2.82802	0.0046837	0.013063299	down-regulated	protein_coding
LLPH	ENSMMUG00000016620	33.23924848	-2.872262092	1.016159	-2.82659	0.0047047	0.013119218	down-regulated	protein_coding
SAFB	ENSMMUG00000017970	168.9440072	-1.481172304	0.524195	-2.82561	0.004719	0.013156607	down-regulated	protein_coding
NDUFS2	ENSMMUG00000004520	265.829117	-1.202548896	0.425756	-2.8245	0.0047354	0.013197575	down-regulated	protein_coding
RIOK1	ENSMMUG00000015577	20.74077003	-3.720395721	1.317239	-2.82439	0.0047371	0.013197575	down-regulated	protein_coding
FAH	ENSMMUG00000017060	94.47188011	-2.039829242	0.722226	-2.82436	0.0047375	0.013197575	down-regulated	protein_coding
HMGA1	ENSMMUG00000019529	1086.96603	-2.069791195	0.732853	-2.82429	0.0047385	0.013197818	down-regulated	protein_coding
UNK	ENSMMUG00000013646	58.57356467	1.120816918	0.396953	2.82355	0.0047495	0.013223267	up-regulated	protein_coding
SCN10A	ENSMMUG00000015827	21.00708859	10.2906711	3.645482	2.822856	0.0047598	0.013249339	up-regulated	protein_coding
RALY	ENSMMUG00000018093	415.2221602	-1.433289646	0.507757	-2.82279	0.0047608	0.013249494	down-regulated	protein_coding
FZD3	ENSMMUG00000007486	9.175192424	2.441419935	0.865149	2.821964	0.0047731	0.013281027	up-regulated	protein_coding
TOMM70A	ENSMMUG00000020025	129.584632	-1.097399096	0.3889	-2.8218	0.0047754	0.013285011	down-regulated	protein_coding
FAM208A	ENSMMUG00000002820	147.8571628	1.052338199	0.373026	2.821087	0.0047861	0.01330953	up-regulated	protein_coding
FAM135A	ENSMMUG00000009060	106.331011	-1.602698262	0.56817	-2.82081	0.0047903	0.013317672	down-regulated	protein_coding
CIAPIN1	ENSMMUG00000020472	66.34375671	-1.917941917	0.68007	-2.82021	0.0047992	0.013337902	down-regulated	protein_coding
FLJ35808	ENSMMUG00000003436	36.26044774	2.018362879	0.715953	2.819129	0.0048154	0.013377824	up-regulated	protein_coding
PHF20L1	ENSMMUG00000010191	35.17312931	-2.16352034	0.767426	-2.81919	0.0048145	0.013377824	down-regulated	protein_coding
PEX13	ENSMMUG00000005687	31.2581866	-2.259250769	0.801947	-2.81721	0.0048443	0.013450093	down-regulated	protein_coding
TRIM47	ENSMMUG00000009377	21.02878573	-3.742026964	1.328326	-2.8171	0.0048459	0.013450093	down-regulated	protein_coding
SAMM50	ENSMMUG00000015446	98.55270109	-1.718977654	0.610197	-2.81708	0.0048462	0.013450093	down-regulated	protein_coding
AP1B1	ENSMMUG00000021380	236.5790173	-1.155749219	0.410254	-2.81715	0.0048451	0.013450093	down-regulated	protein_coding
UBL4A	ENSMMUG00000009015	72.29741984	-1.983564812	0.704231	-2.81664	0.0048529	0.013463493	down-regulated	protein_coding
MMP1	ENSMMUG00000002037	241.814737	-2.197812781	0.780481	-2.81597	0.004863	0.013486157	down-regulated	protein_coding
PGD	ENSMMUG00000023029	417.4953809	-1.230232106	0.436872	-2.816	0.0048625	0.013486157	down-regulated	protein_coding
APBA3	ENSMMUG00000009672	78.95781457	1.268159268	0.450404	2.815606	0.0048685	0.013496754	up-regulated	protein_coding
ADIPOR2	ENSMMUG00000017262	567.884812	-1.234589354	0.438599	-2.81485	0.0048801	0.013522895	down-regulated	protein_coding
DEGS1	ENSMMUG00000002400	87.48580076	-2.068218533	0.734844	-2.8145	0.0048853	0.013534867	down-regulated	protein_coding
FCMD	ENSMMUG00000002669	23.83619458	-3.039929393	1.080122	-2.81443	0.0048864	0.013535106	down-regulated	protein_coding
NGDN	ENSMMUG00000014766	29.52417744	-3.357908834	1.19342	-2.81369	0.0048977	0.013558556	down-regulated	protein_coding
EGR2	ENSMMUG00000010256	13.7688813	3.19691978	1.136352	2.813319	0.0049033	0.013571354	up-regulated	protein_coding
SCAND1	ENSMMUG00000009306	90.66644753	-2.146609356	0.763048	-2.8132	0.0049051	0.013573587	down-regulated	protein_coding
BAZ1B	ENSMMUG00000014270	95.79957698	-1.15311814	0.410066	-2.81203	0.004923	0.013620603	down-regulated	protein_coding
SH3PXD2A	ENSMMUG00000007067	77.3294633	1.568332074	0.557761	2.811835	0.004926	0.013626115	up-regulated	protein_coding
PYCR2	ENSMMUG00000011434	92.88093673	-1.559791282	0.554814	-2.81137	0.004933	0.013642978	down-regulated	protein_coding
SMAD4	ENSMMUG00000018334	52.24285877	-2.175219643	0.774119	-2.80993	0.0049553	0.013701789	down-regulated	protein_coding
DYNLRB1	ENSMMUG00000031253	34.84185904	-2.59205914	0.922505	-2.8098	0.0049572	0.013704349	down-regulated	protein_coding
C8ORF30A	ENSMMUG00000014717	11.2606074	-3.860460804	1.373985	-2.80968	0.0049591	0.013706903	down-regulated	protein_coding
ERGIC2	ENSMMUG00000015811	50.70125971	-1.554818592	0.55341	-2.80952	0.0049615	0.013711013	down-regulated	protein_coding
OSBPL2	ENSMMUG00000012623	101.022386	-1.397698574	0.497595	-2.80891	0.004971	0.013731849	down-regulated	protein_coding
TTF1	ENSMMUG00000006833	18.7228955	-3.562057246	1.268317	-2.80849	0.0049774	0.013746959	down-regulated	protein_coding
RBM42	ENSMMUG00000018954	141.7111291	-1.652384798	0.588471	-2.80793	0.0049861	0.013768287	down-regulated	protein_coding
ANKRD39	ENSMMUG00000030736	13.92368252	-4.166128938	1.484316	-2.80677	0.0050042	0.013815368	down-regulated	protein_coding
IL12RB1	ENSMMUG00000012096	4.633344937	4.053712891	1.444331	2.806637	0.0050062	0.013818199	up-regulated	protein_coding
AP1AR	ENSMMUG00000015591	37.45964818	-3.029281325	1.079837	-2.80531	0.0050268	0.013872399	down-regulated	protein_coding
SCAF1	ENSMMUG00000012938	233.0473026	-1.065307732	0.379792	-2.80497	0.005032	0.013884258	down-regulated	protein_coding
COX7A2L	ENSMMUG00000016278	35.84283154	-2.22417559	0.793061	-2.80455	0.0050387	0.013899994	down-regulated	protein_coding
ZNF322A	ENSMMUG00000017480	18.77784412	-3.517078396	1.254176	-2.80429	0.0050427	0.013908228	down-regulated	protein_coding
C19orf66	ENSMMUG00000018138	26.85703184	-3.236211915	1.154164	-2.80395	0.0050481	0.013920537	down-regulated	protein_coding
TOM1L1	ENSMMUG00000010550	223.9025112	-1.051492304	0.375023	-2.80381	0.0050503	0.013923668	down-regulated	protein_coding
LCMT1	ENSMMUG00000005942	28.76475755	-3.255297042	1.161165	-2.80347	0.0050555	0.013935455	down-regulated	protein_coding
Y_RNA	ENSMMUG00000027736	13.23791285	9.631043746	3.436149	2.802859	0.0050652	0.013956601	up-regulated	misc_RNA
GCHFR	ENSMMUG00000013159	59.10079902	-1.758265726	0.627332	-2.80277	0.0050666	0.013957854	down-regulated	protein_coding
EIF3H	ENSMMUG00000020283	226.6040771	-1.714970296	0.612058	-2.80197	0.0050791	0.013989578	down-regulated	protein_coding
ARHGAP12	ENSMMUG00000004778	114.1341252	-1.594110566	0.568967	-2.80177	0.0050824	0.01399313	down-regulated	protein_coding
RAP1GDS1	ENSMMUG00000022417	50.53446132	-2.08677243	0.744791	-2.80182	0.0050815	0.01399313	down-regulated	protein_coding
U6	ENSMMUG00000025259	14.1507716	9.726558119	3.47238	2.801121	0.0050925	0.014018352	up-regulated	snRNA
MCCC1	ENSMMUG00000010553	40.70149139	-1.982046793	0.707727	-2.80058	0.0051011	0.014039107	down-regulated	protein_coding
CUL3	ENSMMUG00000022259	134.5372556	-1.139720454	0.407079	-2.79975	0.0051142	0.014072504	down-regulated	protein_coding
CUTC	ENSMMUG00000023763	19.25099296	-3.60257875	1.286801	-2.79964	0.005116	0.014074634	down-regulated	protein_coding
CD97	ENSMMUG00000005722	67.32050242	-1.426373019	0.509572	-2.79916	0.0051236	0.014083458	down-regulated	protein_coding
PVRL3	ENSMMUG00000009998	70.03484581	-1.47620718	0.527382	-2.79912	0.0051242	0.014083458	down-regulated	protein_coding
NPR2	ENSMMUG00000016334	6.911412764	3.152678931	1.126297	2.799154	0.0051237	0.014083458	up-regulated	protein_coding
MFAP1	ENSMMUG00000022773	43.25134743	-2.007068708	0.717019	-2.79918	0.0051232	0.014083458	down-regulated	protein_coding
Y_RNA	ENSMMUG00000027344	5.670319771	3.86563229	1.381264	2.798618	0.0051322	0.014102762	up-regulated	misc_RNA
TOMM40	ENSMMUG00000014303	138.2891118	-2.715271567	0.970511	-2.79778	0.0051456	0.014135536	down-regulated	protein_coding
CYP4F22	ENSMMUG00000016996	8.903072427	4.585769565	1.639096	2.797743	0.0051461	0.014135536	up-regulated	protein_coding
C15orf61	ENSMMUG00000020012	12.83557788	-4.048943398	1.447399	-2.79739	0.0051517	0.014145385	down-regulated	protein_coding
MCTS1	ENSMMUG00000009043	35.61696836	-2.251965931	0.805047	-2.79731	0.005153	0.014146292	down-regulated	protein_coding
PUSL1	ENSMMUG00000016279	25.52575129	1.69029217	0.604355	2.796851	0.0051603	0.014163643	up-regulated	protein_coding
ANKAR	ENSMMUG00000014314	11.34565989	2.763663473	0.988232	2.796575	0.0051647	0.014173005	up-regulated	protein_coding
CSAD	ENSMMUG00000015153	65.8364705	1.493902899	0.534258	2.796222	0.0051704	0.014185531	up-regulated	protein_coding
SLC6A17	ENSMMUG00000021544	5.563540595	4.915397435	1.757908	2.796164	0.0051713	0.014185531	up-regulated	protein_coding
U4atac	ENSMMUG00000035767	3.724788281	4.446807439	1.590751	2.795414	0.0051833	0.01421574	up-regulated	snRNA
FXC1	ENSMMUG00000007732	57.44417008	-1.723764447	0.616754	-2.7949	0.0051916	0.014232982	down-regulated	protein_coding
VANGL2	ENSMMUG00000020295	6.06288261	3.70476782	1.325522	2.794951	0.0051908	0.014232982	up-regulated	protein_coding
EIF5A2	ENSMMUG00000022880	13.57344486	-4.13294631	1.478868	-2.79467	0.0051953	0.014240213	down-regulated	protein_coding
LSM12	ENSMMUG00000006804	71.24670689	-1.488900106	0.532845	-2.79425	0.0052021	0.01425321	down-regulated	protein_coding
ADH1A	ENSMMUG00000018738	135.3003448	-2.079097747	0.74407	-2.79422	0.0052024	0.01425321	down-regulated	protein_coding
SPAST	ENSMMUG00000021691	34.15502954	2.272232329	0.8132	2.794187	0.005203	0.01425321	up-regulated	protein_coding
UBE2L6	ENSMMUG00000001332	10.73373418	-3.7940072	1.35803	-2.79376	0.0052099	0.014269358	down-regulated	protein_coding
CSNK1G3	ENSMMUG00000006646	67.7549394	-1.953841892	0.699603	-2.79279	0.0052256	0.014306675	down-regulated	protein_coding
TTC13	ENSMMUG00000005888	32.17387806	-2.366639684	0.847687	-2.79188	0.0052403	0.014344161	down-regulated	protein_coding

LRRC19	ENSMMUG00000006079	82.78576438	-1.673853255	0.59962	-2.79153	0.005246	0.014357059	down-regulated	protein_coding
CLCN1	ENSMMUG00000012632	11.82258917	3.992101619	1.430318	2.79106	0.0052536	0.014374956	up-regulated	protein_coding
KCTD2	ENSMMUG00000011648	52.35294832	-1.491653346	0.534491	-2.79079	0.0052579	0.014384055	down-regulated	protein_coding
mml-mir-675	ENSMMUG00000032643	261.6139492	-3.588119151	1.285908	-2.79034	0.0052653	0.014401464	down-regulated	miRNA
SPCS1	ENSMMUG00000017370	208.7586805	1.169206341	0.419068	2.790013	0.0052706	0.014413149	up-regulated	protein_coding
CTSB	ENSMMUG00000019212	461.6026555	-1.203014171	0.431305	-2.78924	0.0052832	0.014444707	down-regulated	protein_coding
ZNF823	ENSMMUG00000008815	9.783564204	-3.659780251	1.31225	-2.78894	0.0052882	0.014455604	down-regulated	protein_coding
WIBG	ENSMMUG00000011002	37.2019752	-2.256462883	0.809242	-2.78836	0.0052975	0.014478147	down-regulated	protein_coding
ST3GAL3	ENSMMUG00000015555	14.94502203	2.349328228	0.842565	2.788305	0.0052985	0.014478147	up-regulated	protein_coding
VPS37C	ENSMMUG00000015019	57.33565917	-1.889483267	0.677799	-2.78767	0.0053088	0.014503559	down-regulated	protein_coding
ATXN2	ENSMMUG00000002226	66.91482124	-1.327328891	0.476164	-2.78755	0.0053109	0.014506423	down-regulated	protein_coding
HAPLN4	ENSMMUG00000008578	9.733632199	-3.651903671	1.310179	-2.78733	0.0053144	0.014510482	down-regulated	protein_coding
WRN	ENSMMUG00000022655	19.10119695	-3.590914483	1.288284	-2.78736	0.0053139	0.014510482	down-regulated	protein_coding
WASF3	ENSMMUG00000005055	9.682801213	-3.645471691	1.308156	-2.78673	0.0053243	0.014534861	down-regulated	protein_coding
DNAH6	ENSMMUG00000019116	9.321256798	2.935390657	1.05338	2.786641	0.0053258	0.014535889	up-regulated	protein_coding
Y_RNA	ENSMMUG00000025050	12.93460057	9.598482078	3.444698	2.786451	0.0053289	0.0145388	up-regulated	misc_RNA
HMOX2	ENSMMUG00000009901	99.62606353	-1.815608109	0.65162	-2.7863	0.0053314	0.014542765	down-regulated	protein_coding
RASSF4	ENSMMUG00000017489	68.08427614	-1.649827762	0.592163	-2.78611	0.0053345	0.014548676	down-regulated	protein_coding
DCTN5	ENSMMUG00000017305	11.56327093	-3.902453808	1.401372	-2.78474	0.0053571	0.014607352	down-regulated	protein_coding
IREB2	ENSMMUG00000023474	107.6571382	-1.471531201	0.528487	-2.78443	0.0053623	0.014618647	down-regulated	protein_coding
TBC1D22B	ENSMMUG00000022008	46.45620568	-1.689112619	0.606969	-2.78286	0.0053882	0.014686368	down-regulated	protein_coding
TECPR1	ENSMMUG00000015479	32.22871439	-2.371347114	0.852316	-2.78224	0.0053985	0.014708944	down-regulated	protein_coding
IL17RB	ENSMMUG00000019267	43.67798313	1.340304502	0.481726	2.782294	0.0053976	0.014708944	up-regulated	protein_coding
EMP2	ENSMMUG00000008468	12.44162336	-4.004907723	1.439592	-2.78197	0.0054029	0.014718128	down-regulated	protein_coding
RALB	ENSMMUG00000000462	79.92960895	-1.201393531	0.432057	-2.78064	0.0054252	0.014773175	down-regulated	protein_coding
TMEM167B	ENSMMUG00000031889	18.04296627	-3.513489845	1.263612	-2.78051	0.0054273	0.014775993	down-regulated	protein_coding
ROD1	ENSMMUG00000000464	156.2882488	-1.770891833	0.636935	-2.78033	0.0054303	0.01478143	down-regulated	protein_coding
CLK4	ENSMMUG00000004002	19.02721181	-3.535871026	1.271978	-2.77982	0.0054389	0.014801833	down-regulated	protein_coding
TMEM98	ENSMMUG00000022356	88.38186602	-1.44145543	0.518561	-2.77972	0.0054406	0.014803545	down-regulated	protein_coding
SNRPA1	ENSMMUG00000011826	75.88164347	-1.428793482	0.514107	-2.77918	0.0054497	0.014825496	down-regulated	protein_coding
CDC25C	ENSMMUG00000009025	49.00246909	2.199176883	0.791419	2.778778	0.0054564	0.014840899	up-regulated	protein_coding
ACTR6	ENSMMUG00000023603	18.78482874	-3.567912311	1.28442	-2.77784	0.0054722	0.014880942	down-regulated	protein_coding
N6AMT2	ENSMMUG00000013370	21.492262	-3.724992437	1.341064	-2.77764	0.0054755	0.014887298	down-regulated	protein_coding
WBSCR22	ENSMMUG00000011367	92.57160115	-1.716863374	0.618174	-2.77731	0.005481	0.014899295	down-regulated	protein_coding
TRIP13	ENSMMUG00000014740	15.11761785	-4.285142301	1.542983	-2.77718	0.0054833	0.014902602	down-regulated	protein_coding
CCDC151	ENSMMUG00000016505	8.99218745	2.930100556	1.055658	2.775616	0.0055097	0.014971607	up-regulated	protein_coding
MAP2K4	ENSMMUG00000019842	57.13124069	-1.994584231	0.718744	-2.7751	0.0055185	0.014992553	down-regulated	protein_coding
NR1H4	ENSMMUG00000003571	39.75417976	-2.370776537	0.854383	-2.77484	0.0055229	0.015001606	down-regulated	protein_coding
ATL3	ENSMMUG00000005776	94.97871741	1.381209237	0.497807	2.774586	0.0055272	0.01500792	up-regulated	protein_coding
MME	ENSMMUG00000006662	365.3141909	-2.003155801	0.721968	-2.77458	0.0055273	0.01500792	down-regulated	protein_coding
CCDC132	ENSMMUG00000017311	30.94142017	-2.232436699	0.804793	-2.77393	0.0055384	0.015035146	down-regulated	protein_coding
SHMT2	ENSMMUG00000021785	142.730985	-1.444714517	0.520982	-2.77306	0.0055531	0.015072196	down-regulated	protein_coding
Y_RNA	ENSMMUG00000035859	4.385909053	4.864830091	1.754648	2.772539	0.0055621	0.015093586	up-regulated	misc_RNA
POU2F3	ENSMMUG00000012251	11.27475278	3.488519618	1.258654	2.771628	0.0055777	0.015132982	up-regulated	protein_coding
FBXO42	ENSMMUG00000012733	37.29642758	-2.306423868	0.832211	-2.77144	0.0055809	0.015138787	down-regulated	protein_coding
U6atac	ENSMMUG00000033802	4.469569903	4.900957662	1.768673	2.77098	0.0055888	0.015154417	up-regulated	snRNA
CD101	ENSMMUG00000016904	5.938204762	6.611319103	2.386291	2.770541	0.0055963	0.015171932	up-regulated	protein_coding
TSTA3	ENSMMUG00000014664	117.4719272	-2.090047934	0.754744	-2.76922	0.0056191	0.015230904	down-regulated	protein_coding
ATP5SL	ENSMMUG00000014348	242.3916688	1.028447162	0.37157	2.76784	0.0056429	0.015289474	up-regulated	protein_coding
KIAA1239	ENSMMUG00000003676	7.15012981	2.622473694	0.947841	2.766786	0.0056612	0.015333114	up-regulated	protein_coding
NDUFB1	ENSMMUG00000022428	34.0894123	-2.984190749	1.07866	-2.76657	0.0056649	0.015340229	down-regulated	protein_coding
Y_RNA	ENSMMUG00000028257	4.445812215	5.09852381	1.843121	2.766245	0.0056706	0.015352674	up-regulated	misc_RNA
BRPF3	ENSMMUG00000015416	110.4208663	1.475160247	0.533424	2.765454	0.0056844	0.015387022	up-regulated	protein_coding
EYA2	ENSMMUG00000010769	4.454481255	4.329102127	1.565475	2.765361	0.005686	0.015388488	up-regulated	protein_coding
ITGB1BP2	ENSMMUG00000020479	6.347346675	3.042474475	1.100245	2.76527	0.0056876	0.015389808	up-regulated	protein_coding
MAPKAPK3	ENSMMUG00000010495	50.61403803	-1.685367839	0.609633	-2.76456	0.0056999	0.01541738	down-regulated	protein_coding
MSL3	ENSMMUG00000002689	10.16677894	-3.713044601	1.343288	-2.76415	0.0057072	0.015434094	down-regulated	protein_coding
MAPK15	ENSMMUG00000001187	6.864153805	3.801273657	1.375273	2.764013	0.0057095	0.015434484	up-regulated	protein_coding
MAGOH	ENSMMUG00000007611	22.34032358	-3.826948811	1.384552	-2.76403	0.0057091	0.015434484	down-regulated	protein_coding
NAGS	ENSMMUG00000009713	42.36262043	1.809398833	0.654878	2.762957	0.005728	0.015481066	up-regulated	protein_coding
FAM63B	ENSMMUG00000018109	44.99954332	1.622290063	0.587168	2.762905	0.0057289	0.015481066	up-regulated	protein_coding
ADAMTS8	ENSMMUG00000003300	4.296866626	4.349482951	1.574402	2.762625	0.0057339	0.015491369	up-regulated	protein_coding
LEPROT	ENSMMUG00000003264	24.75710883	-3.083454988	1.116464	-2.7618	0.0057483	0.0155245	down-regulated	protein_coding
TMEM150B	ENSMMUG00000028716	112.1869362	-1.21263941	0.439097	-2.76167	0.0057507	0.01552808	down-regulated	protein_coding
TSPAN5	ENSMMUG00000011702	10.16926428	3.591582469	1.300825	2.761004	0.0057624	0.015556607	up-regulated	protein_coding
OCEL1	ENSMMUG00000011931	65.50198055	-1.771491008	0.641645	-2.76086	0.0057649	0.015560523	down-regulated	protein_coding
PDRG1	ENSMMUG00000000973	54.37954569	-2.018586967	0.73144	-2.75974	0.0057847	0.015610758	down-regulated	protein_coding
ZNF506	ENSMMUG00000028833	30.4002933	-2.824946741	1.024164	-2.75829	0.0058104	0.015674208	down-regulated	protein_coding
DLL3	ENSMMUG00000019093	8.33460432	6.632953603	2.405757	2.757117	0.0058314	0.015727762	up-regulated	protein_coding
EDF1	ENSMMUG00000005051	593.9527898	-1.057079451	0.383572	-2.75588	0.0058534	0.015784296	down-regulated	protein_coding
BTG3	ENSMMUG00000002116	73.53664663	1.195956988	0.434015	2.755566	0.0058591	0.015796507	up-regulated	protein_coding
CCR4	ENSMMUG00000022661	4.197856678	5.010449969	1.818474	2.755305	0.0058637	0.015806088	up-regulated	protein_coding
ACAA2	ENSMMUG00000003040	167.3466147	-1.400436728	0.508517	-2.75396	0.0058879	0.015865028	down-regulated	protein_coding
5S_rRNA	ENSMMUG00000028160	5.317063086	5.031493976	1.827136	2.75376	0.0058915	0.015871807	up-regulated	rRNA
MCFD2	ENSMMUG00000006254	35.08444814	-2.764260039	1.003931	-2.75344	0.0058973	0.015884557	down-regulated	protein_coding
LRCH1	ENSMMUG00000016293	34.49398688	-2.544007429	0.924252	-2.7525	0.0059142	0.015926802	down-regulated	protein_coding
RALA	ENSMMUG00000016059	64.24003409	-1.410581543	0.512548	-2.7521	0.0059215	0.015943603	down-regulated	protein_coding
AOC3	ENSMMUG00000001871	7.026893524	3.11003986	1.130129	2.751932	0.0059245	0.01594852	up-regulated	protein_coding
METTL10	ENSMMUG00000015217	9.551581827	-3.623771924	1.317231	-2.75105	0.0059404	0.015988383	down-regulated	protein_coding
MFSD8	ENSMMUG00000022751	9.535148655	-3.622512681	1.316882	-2.75083	0.0059445	0.015996431	down-regulated	protein_coding
UNKL	ENSMMUG00000006693	10.44582712	3.385466352	1.230802	2.750619	0.0059483	0.016003453	up-regulated	protein_coding
snoU13	ENSMMUG00000035987	4.575302264	4.719052014	1.715836	2.750293	0.0059542	0.016016333	up-regulated	snoRNA
COXI	ENSMMUG00000028689	10657.29201	3.909693144	1.421649	2.750111	0.0059575	0.016022211	up-regulated	protein_coding
DAZAP1	ENSMMUG00000003047	171.2568852	-1.768637069	0.643251	-2.74953	0.0059681	0.016038588	down-regulated	protein_coding
SPTBN1	ENSMMUG00000013541	2185.912113	1.049965151	0.38188	2.749463	0.0059693	0.016038678	up-regulated	protein_coding
EDC4	ENSMMUG00000011888	101.9029824	-1.563293099	0.568611	-2.74932	0.0059719	0.016042593	down-regulated	protein_coding
EPT1	ENSMMUG00000014287	23.74806846	-3.921637965	1.426999	-2.74817	0.0059929	0.016093987	down-regulated	protein_coding
LINC00493	ENSMMUG00000031145	26.42735472	-3.124676307	1.137012	-2.74815	0.0059933	0.016093987	down-regulated	protein_coding
EBNA1BP2	ENSMMUG00000002640	98.40485215	-1.831843526	0.666739	-2.74747	0.0060057	0.016121251	down-regulated	protein_coding
NKIRAS1	ENSMMUG00000006352	7.57477572	2.755111785	1.002867	2.747235	0.00601	0.016129653	up-regulated	protein_coding
TMX1	ENSMMUG00000001901	46.44804331	-2.18174463	0.794188	-2.74714	0.0060117	0.016131275	down-regulated	protein_coding
SLCO4C1	ENSMMUG00000007773	3.845042068	4.685385986	1.705813	2.746717	0.0060195	0.01614291	up-regulated	protein_coding
PNRC1	ENSMMUG00000014940	144.5642336	-1.165289045	0.424247	-2.74672	0.0060194	0.01614291	down-regulated	protein_coding
SEC14L2	ENSMMUG00000013404	114.4161031	-1.103038395	0.401725	-2.74576	0.0060371	0.0161871	down-regulated	protein_coding
POLH	ENSMMUG00000008903	65.03893039	1.467377227	0.534492	2.745369	0.0060443	0.016197504	up-regulated	protein_coding
BNIP3	ENSMMUG00000022445	228.3422448	-1.820916939	0.663255	-2.74543	0.0060433	0.016197504	down-regulated	protein_coding
LIN7C	ENSMMUG00000023119	19.02965916	-3.58828917	1.307037	-2.74536	0.0060444	0.016197504	down-regulated	protein_coding
VMO1	ENSMMUG00000012997	5.104936265	3.576230985	1.302693	2.745259	0.0060463	0.016199441	up-regulated	protein_coding
KAT6B	ENSMMUG00000023488	56.09407698	1.251018946	0.455784	2.744764	0.0060555	0.01622086	up-regulated	protein_coding
C12orf45	ENSMMUG00000017133	12.728952	-4.037204585	1.471293	-2.74398	0.0060699	0.016256387	down-regulated	protein_coding

LARP4B	ENSMMUG00000005279	203.5267426	-1.078413616	0.393066	-2.7436	0.006077	0.016272522	down-regulated	protein_coding
C1H1ORF123	ENSMMUG00000007608	33.26309768	-2.491737375	0.908421	-2.74293	0.0060893	0.016300984	down-regulated	protein_coding
ZNF207	ENSMMUG00000016875	142.5265106	-1.605865685	0.585463	-2.7429	0.00609	0.016300984	down-regulated	protein_coding
Y_RNA	ENSMMUG00000037200	5.029592827	3.797097817	1.384813	2.741957	0.0061074	0.016341565	up-regulated	misc_RNA
LRRC8C	ENSMMUG00000008003	9.452335754	4.417337964	1.611307	2.741463	0.0061166	0.016363019	up-regulated	protein_coding
STARD7	ENSMMUG00000023260	253.4494646	-1.006309784	0.367141	-2.74093	0.0061265	0.016383193	down-regulated	protein_coding
EHBP1L1	ENSMMUG00000014169	185.4639583	-1.282679527	0.467983	-2.74087	0.0061277	0.016383306	down-regulated	protein_coding
FABP2	ENSMMUG00000009031	189.1376867	-1.659161382	0.605409	-2.74056	0.0061334	0.016392432	down-regulated	protein_coding
RIPK3	ENSMMUG00000020701	70.80392042	-2.141345073	0.781349	-2.74057	0.0061332	0.016392432	down-regulated	protein_coding
SH2D2A	ENSMMUG00000010917	7.683348042	4.680943826	1.708155	2.74035	0.0061374	0.016399947	up-regulated	protein_coding
CYP4F11	ENSMMUG00000006873	259.0280283	-1.937605022	0.70745	-2.73886	0.0061653	0.016468331	down-regulated	protein_coding
GPD1	ENSMMUG00000006532	145.4895757	-1.992511942	0.72785	-2.73753	0.0061902	0.016525554	down-regulated	protein_coding
MRPL46	ENSMMUG00000013658	37.1583099	-3.044834056	1.112249	-2.73755	0.0061899	0.016525554	down-regulated	protein_coding
FGFR4	ENSMMUG00000004919	95.98474361	-2.083433339	0.761187	-2.73708	0.0061986	0.016541807	down-regulated	protein_coding
ANKRD13C	ENSMMUG00000013853	56.93556647	-1.585122024	0.57912	-2.73712	0.0061979	0.016541807	down-regulated	protein_coding
C10ORF54	ENSMMUG00000012967	151.9183797	-1.871546218	0.683846	-2.73679	0.0062041	0.016550137	down-regulated	protein_coding
ASXL2	ENSMMUG00000001377	59.27341361	-1.922025938	0.702495	-2.736	0.0062191	0.016583911	down-regulated	protein_coding
RPRD1B	ENSMMUG00000018924	47.19117094	-1.502643261	0.549259	-2.73576	0.0062236	0.016592674	down-regulated	protein_coding
KCTD15	ENSMMUG00000011319	12.35159956	-3.995090975	1.460444	-2.73553	0.006228	0.016598122	down-regulated	protein_coding
VOPP1	ENSMMUG00000013015	53.73934778	-2.200511329	0.804447	-2.73543	0.0062298	0.016599968	down-regulated	protein_coding
YTHDC2	ENSMMUG00000001376	27.53770763	-3.097206795	1.13233	-2.73525	0.0062333	0.016604947	down-regulated	protein_coding
PRKCD	ENSMMUG00000007230	122.9833928	-1.009335014	0.36923	-2.73362	0.0062642	0.016675854	down-regulated	protein_coding
MRPL33	ENSMMUG00000022650	21.20541304	-3.748806083	1.371357	-2.73365	0.0062637	0.016675854	down-regulated	protein_coding
SLC5A9	ENSMMUG00000007558	362.0210927	1.550628043	0.567291	2.733392	0.0062686	0.016684351	up-regulated	protein_coding
ENTPD4	ENSMMUG00000016659	58.31447849	-1.960835256	0.717399	-2.73326	0.0062711	0.016688065	down-regulated	protein_coding
ANKRA2	ENSMMUG00000023513	24.64378167	-3.020896412	1.105313	-2.73307	0.0062747	0.016694451	down-regulated	protein_coding
CFAP97	ENSMMUG00000015473	11.27620048	-3.866059209	1.41461	-2.73295	0.006277	0.016697288	down-regulated	protein_coding
ZNF276	ENSMMUG00000002506	122.5898541	1.94407278	0.711553	2.732156	0.0062921	0.016734472	up-regulated	protein_coding
GCH1	ENSMMUG00000004084	48.34346093	-2.271720765	0.831542	-2.73194	0.0062963	0.01673664	down-regulated	protein_coding
KCNAB2	ENSMMUG00000016837	12.29883864	-3.990874107	1.460799	-2.73198	0.0062955	0.01673664	down-regulated	protein_coding
PRICKLE3	ENSMMUG00000023024	34.06065673	1.630214432	0.596727	2.731928	0.0062965	0.01673664	up-regulated	protein_coding
RNF138	ENSMMUG00000012849	9.475091881	-3.612108008	1.322687	-2.73089	0.0063164	0.016786426	down-regulated	protein_coding
PRKAB1	ENSMMUG00000019860	78.53680683	-1.239897466	0.454214	-2.72976	0.006338	0.01684062	down-regulated	protein_coding
BRSK1	ENSMMUG00000002965	6.118503987	3.657414928	1.339987	2.72944	0.0063442	0.016853939	up-regulated	protein_coding
FTH1	ENSMMUG00000032350	101.90339	-1.651643519	0.605156	-2.72929	0.0063472	0.016858618	down-regulated	protein_coding
SCAND3	ENSMMUG00000004619	9.982658292	-3.689877298	1.352691	-2.7278	0.0063757	0.016924995	down-regulated	protein_coding
FCRLA	ENSMMUG00000004042	16.77731391	9.954345244	3.649362	2.727694	0.0063779	0.016927483	up-regulated	protein_coding
GTPBP10	ENSMMUG00000023656	9.898015525	-3.674898509	1.347415	-2.72737	0.0063842	0.016941005	down-regulated	protein_coding
NDUFA11	ENSMMUG00000016665	138.186694	-2.186245669	0.801682	-2.72707	0.0063899	0.016953101	down-regulated	protein_coding
ZNF524	ENSMMUG00000006774	28.12965972	-3.21722618	1.179875	-2.72675	0.0063961	0.01696634	down-regulated	protein_coding
CNOT8	ENSMMUG00000008318	54.26153228	-1.52924396	0.560887	-2.72647	0.0064015	0.016977581	down-regulated	protein_coding
CXorf23	ENSMMUG00000004745	9.296573492	-3.587061363	1.31571	-2.72633	0.0064043	0.016978468	down-regulated	protein_coding
ZHX2	ENSMMUG00000005363	19.80045836	1.782125314	0.653764	2.725947	0.0064117	0.016995022	up-regulated	protein_coding
STAU2	ENSMMUG00000021588	36.3479613	-2.471377207	0.906637	-2.72587	0.0064132	0.016995738	down-regulated	protein_coding
UPP1	ENSMMUG00000007037	86.54854236	-1.630138206	0.598163	-2.72524	0.0064255	0.017025157	down-regulated	protein_coding
UBE2M	ENSMMUG00000022621	88.67860437	-1.618052867	0.59395	-2.72422	0.0064453	0.017074477	down-regulated	protein_coding
BRD7	ENSMMUG00000003119	159.6156261	-1.083332399	0.397683	-2.72411	0.0064475	0.017077041	down-regulated	protein_coding
BATF2	ENSMMUG00000006568	9.353704096	-3.595258284	1.320208	-2.72325	0.0064643	0.017118264	down-regulated	protein_coding
NUPL1	ENSMMUG00000018420	27.80232871	-3.107186234	1.141142	-2.72288	0.0064717	0.017134615	down-regulated	protein_coding
TLN2	ENSMMUG00000011503	86.19906114	1.350186712	0.495903	2.722682	0.0064754	0.017141425	up-regulated	protein_coding
SGSM1	ENSMMUG00000010839	14.27013713	2.49966008	0.918162	2.722461	0.0064798	0.01714969	up-regulated	protein_coding
PLA2G4C	ENSMMUG00000014293	62.46633098	-1.589160825	0.583753	-2.72232	0.0064826	0.01715387	down-regulated	protein_coding
PHACTR4	ENSMMUG00000019216	52.38774659	1.638307599	0.60186	2.722075	0.0064873	0.017163319	up-regulated	protein_coding
DLGAP1	ENSMMUG00000001471	4.566301758	3.560414548	1.308155	2.721708	0.0064946	0.017177258	up-regulated	protein_coding
SMN1	ENSMMUG00000020214	33.90550735	-2.109351894	0.775472	-2.72009	0.0065265	0.017257151	down-regulated	protein_coding
MALSU1	ENSMMUG00000004841	29.91072655	-2.769733685	1.018554	-2.71928	0.0065424	0.017296118	down-regulated	protein_coding
ECT2	ENSMMUG00000017014	40.50193034	-2.567940491	0.944377	-2.71919	0.0065442	0.017297539	down-regulated	protein_coding
CPM	ENSMMUG00000000946	11.58805728	-3.90559209	1.436356	-2.7191	0.006546	0.0172992	down-regulated	protein_coding
PAK1	ENSMMUG00000001387	167.7397103	-1.733539344	0.637563	-2.71901	0.0065478	0.017300587	down-regulated	protein_coding
STK36	ENSMMUG00000006744	20.93517339	1.860275367	0.684208	2.718872	0.0065505	0.017304484	up-regulated	protein_coding
STRIP2	ENSMMUG00000019359	11.27478655	3.490870481	1.28399	2.718767	0.0065526	0.017306793	up-regulated	protein_coding
ARSB	ENSMMUG00000006792	19.92380963	2.892800197	1.064808	2.716734	0.006593	0.01740371	up-regulated	protein_coding
SIM2	ENSMMUG00000015564	4.838416047	4.312923364	1.587514	2.716778	0.0065921	0.01740371	up-regulated	protein_coding
LYAR	ENSMMUG00000023393	19.00150854	-3.588091215	1.320689	-2.71683	0.006591	0.01740371	down-regulated	protein_coding
SNX22	ENSMMUG00000021122	4.59967536	4.063342331	1.49572	2.716647	0.0065947	0.017405009	up-regulated	protein_coding
RTFDC1	ENSMMUG00000000074	107.222929	-1.221793854	0.449939	-2.71547	0.0066182	0.01745916	down-regulated	protein_coding
CUTA	ENSMMUG00000010658	71.65217446	-2.128470772	0.783842	-2.71543	0.0066189	0.01745916	down-regulated	protein_coding
ENG	ENSMMUG00000002248	54.69259203	2.882372882	1.061517	2.715333	0.0066209	0.017461178	up-regulated	protein_coding
KCTD21	ENSMMUG00000017474	25.1028863	2.033117569	0.748775	2.71526	0.0066224	0.017461794	up-regulated	protein_coding
INCENP	ENSMMUG00000004420	90.50173469	1.494417282	0.550438	2.714962	0.0066283	0.01747426	up-regulated	protein_coding
DYNLRB2	ENSMMUG00000001907	10.39905382	-3.745297866	1.37965	-2.71467	0.0066341	0.017484721	down-regulated	protein_coding
LPA	ENSMMUG00000016201	16.14228769	9.90834544	3.649966	2.71464	0.0066348	0.017484721	up-regulated	protein_coding
TNFRSF1B	ENSMMUG00000021447	92.67179928	-1.309471435	0.482389	-2.71456	0.0066364	0.017485861	down-regulated	protein_coding
MMD	ENSMMUG00000022628	26.32996429	-2.493017727	0.9185	-2.71423	0.0066431	0.017500092	down-regulated	protein_coding
COL5A1	ENSMMUG00000012147	4.230062554	4.139542377	1.525381	2.713776	0.0066521	0.017520633	up-regulated	protein_coding
MOSPD3	ENSMMUG00000002949	27.04471485	-2.600451937	0.95852	-2.71299	0.006668	0.017555857	down-regulated	protein_coding
LIG4	ENSMMUG00000008874	11.23837386	-3.861292809	1.423497	-2.71254	0.006677	0.017576288	down-regulated	protein_coding
COMMD3	ENSMMUG00000000878	43.13057455	-2.568528465	0.946948	-2.71243	0.0066792	0.01757896	down-regulated	protein_coding
UCHL3	ENSMMUG00000003560	36.49678321	-2.303060164	0.849367	-2.7115	0.0066979	0.017621633	down-regulated	protein_coding
C4H6orf15	ENSMMUG00000015674	14.26235118	-4.203864372	1.550555	-2.7112	0.006704	0.017631068	down-regulated	protein_coding
DRG1	ENSMMUG00000019099	53.73231357	-1.79866477	0.663468	-2.711	0.006708	0.017638184	down-regulated	protein_coding
MRM1	ENSMMUG00000000622	24.45305427	-3.088939631	1.139831	-2.71	0.0067284	0.017688542	down-regulated	protein_coding
SLC43A2	ENSMMUG00000017561	121.1677443	-1.510092718	0.557298	-2.70967	0.006735	0.017702797	down-regulated	protein_coding
TMEM203	ENSMMUG00000018106	16.84192678	-3.407847756	1.257726	-2.70953	0.0067378	0.0177068	down-regulated	protein_coding
PIK3C2A	ENSMMUG00000022548	64.27403434	-2.159119257	0.796884	-2.70945	0.0067394	0.017707742	down-regulated	protein_coding
HAGH	ENSMMUG00000000737	147.6488695	-1.037117217	0.38287	-2.7088	0.0067527	0.017739308	down-regulated	protein_coding
ULK1	ENSMMUG00000015773	78.98616996	-1.74827133	0.645432	-2.70868	0.0067551	0.017742335	down-regulated	protein_coding
TESC	ENSMMUG00000017478	92.16590578	-2.459281311	0.907999	-2.70846	0.0067596	0.017750775	down-regulated	protein_coding
WDR85	ENSMMUG00000019609	38.68905358	1.559518951	0.575998	2.707507	0.0067791	0.017795382	up-regulated	protein_coding
C1QBP	ENSMMUG00000006592	222.1472765	-1.755459784	0.648457	-2.70713	0.0067867	0.017812038	down-regulated	protein_coding
CAB39	ENSMMUG00000009793	141.2417657	-1.704569047	0.62988	-2.70618	0.0068062	0.017860083	down-regulated	protein_coding
LARP7	ENSMMUG00000017911	33.03836604	-2.094402189	0.774005	-2.70593	0.0068114	0.017870305	down-regulated	protein_coding
THOC1	ENSMMUG00000004662	83.48651115	2.076097999	0.767379	2.705441	0.0068214	0.01789317	up-regulated	protein_coding
SLIRP	ENSMMUG00000029256	34.82802754	-2.838381403	1.049409	-2.70474	0.0068357	0.01792751	down-regulated	protein_coding
ZDHHC23	ENSMMUG00000001946	11.96308925	-3.951070597	1.460959	-2.70444	0.006842	0.017940691	down-regulated	protein_coding
CYTIP	ENSMMUG00000014176	38.28926359	-2.402461058	0.888488	-2.70399	0.0068513	0.017961527	down-regulated	protein_coding
STAG2	ENSMMUG00000005825	10.29384369	-3.734645368	1.38133	-2.70366	0.0068581	0.017976102	down-regulated	protein_coding
PPPDE2	ENSMMUG00000001855	34.59258611	-2.079027565	0.769026	-2.70346	0.0068623	0.017983737	down-regulated	protein_coding
NSUN7	ENSMMUG00000012561	17.10926181	2.155836226	0.79781	2.702193	0.0068884	0.018048799	up-regulated	protein_coding
VAMP5	ENSMMUG00000030784	11.38076599	-3.875763165	1.434492	-2.70184	0.0068958	0.018061454	down-regulated	protein_coding

LRRC41	ENSMMUG00000032149	120.2780811	1.316441792	0.487266	2.701688	0.0068988	0.018066205	up-regulated	protein_coding
CELF6	ENSMMUG00000010687	250.0181538	-1.09078471	0.403809	-2.70124	0.0069081	0.018087128	down-regulated	protein_coding
SEPP1	ENSMMUG00000030144	400.9167216	-2.104627852	0.779157	-2.70116	0.0069098	0.018088275	down-regulated	protein_coding
SRPX2	ENSMMUG00000008210	7.095752777	4.53515134	1.679437	2.7004	0.0069256	0.018122924	up-regulated	protein_coding
TTC27	ENSMMUG00000009372	18.54646731	-3.545277123	1.313271	-2.69958	0.0069428	0.018164413	down-regulated	protein_coding
ALDOA	ENSMMUG00000000983	3899.403022	-1.575071354	0.58357	-2.69903	0.0069543	0.018191128	down-regulated	protein_coding
GCK	ENSMMUG00000002427	9.595442376	2.976021896	1.102867	2.698442	0.0069665	0.018216401	up-regulated	protein_coding
PIK3CB	ENSMMUG00000008666	70.57512414	-2.078739842	0.770346	-2.69845	0.0069663	0.018216401	down-regulated	protein_coding
TMIGD1	ENSMMUG00000013279	156.6651265	-2.76934712	1.026486	-2.69789	0.006978	0.018232324	down-regulated	protein_coding
DNM1	ENSMMUG00000013717	17.74169408	2.368190624	0.877804	2.697859	0.0069787	0.018232324	up-regulated	protein_coding
SNAPC3	ENSMMUG00000016189	30.72580365	-2.232065552	0.827302	-2.69801	0.0069756	0.018232324	down-regulated	protein_coding
ZNF213	ENSMMUG00000020970	62.10132676	1.112592506	0.412743	2.695609	0.007026	0.018351694	up-regulated	protein_coding
ADAMTS15	ENSMMUG00000003301	10.59026891	2.8216251	1.04712	2.694652	0.0070462	0.018401045	up-regulated	protein_coding
GBE1	ENSMMUG00000015877	147.1126354	-1.551887052	0.576186	-2.69338	0.0070732	0.018464761	down-regulated	protein_coding
ARL14	ENSMMUG00000031560	37.66108137	-1.922404482	0.713813	-2.69315	0.007078	0.018473929	down-regulated	protein_coding
ELOVL7	ENSMMUG00000014211	14.02757894	-4.180608985	1.553374	-2.69131	0.0071172	0.018565986	down-regulated	protein_coding
PDHA1	ENSMMUG00000017336	98.2757196	-1.460276252	0.542667	-2.69093	0.0071254	0.018583807	down-regulated	protein_coding
LRRC48	ENSMMUG00000002942	7.718863084	3.645827809	1.35504	2.690568	0.0071331	0.018600357	up-regulated	protein_coding
SLC39A5	ENSMMUG00000008674	55.97390841	1.924339058	0.715541	2.689349	0.0071592	0.018665005	up-regulated	protein_coding
7SK	ENSMMUG00000036369	15.06655226	9.817329306	3.651219	2.688781	0.0071713	0.018693299	up-regulated	misc_RNA
CCDC126	ENSMMUG00000014987	9.308665064	-3.588884189	1.334964	-2.68837	0.0071801	0.018712661	down-regulated	protein_coding
NSMAF	ENSMMUG00000017297	56.10540157	1.143603083	0.425543	2.687396	0.0072011	0.018764093	up-regulated	protein_coding
RPS11	ENSMMUG00000007549	677.2955913	-1.771710508	0.659336	-2.68711	0.0072072	0.018776449	down-regulated	protein_coding
CHRAC1	ENSMMUG00000008043	8.993428143	-3.538046502	1.317868	-2.68468	0.00726	0.018907091	down-regulated	protein_coding
L3MBTL4	ENSMMUG00000016302	4.003542808	3.899742179	1.453206	2.683544	0.0072846	0.018967656	up-regulated	protein_coding
SLC8A3	ENSMMUG00000017433	14.92892179	9.793195405	3.651564	2.681918	0.0073201	0.019053111	up-regulated	protein_coding
CTBS	ENSMMUG00000010127	28.61077846	-2.635051853	0.982611	-2.68168	0.0073253	0.01905951	down-regulated	protein_coding
DCTPP1	ENSMMUG00000015520	34.83367395	-3.007806198	1.121598	-2.68171	0.0073246	0.01905951	down-regulated	protein_coding
CLDN1	ENSMMUG00000001915	9.34277516	-3.593899383	1.3403	-2.68141	0.0073312	0.019071288	down-regulated	protein_coding
AACS	ENSMMUG00000011117	351.1133495	-1.110064626	0.414133	-2.68046	0.0073522	0.019118952	down-regulated	protein_coding
SRRD	ENSMMUG00000023443	10.41411756	-3.746842614	1.39866	-2.67888	0.0073869	0.019205598	down-regulated	protein_coding
MRPS25	ENSMMUG00000005157	29.10918822	-3.265639208	1.219152	-2.67862	0.0073927	0.019217247	down-regulated	protein_coding
SNORA20	ENSMMUG00000025943	5.94892764	6.475727512	2.4182	2.677913	0.0074083	0.019243514	up-regulated	snoRNA
SIAH1	ENSMMUG00000007400	63.13590844	1.188316872	0.443772	2.677764	0.0074116	0.019248538	up-regulated	protein_coding
CUEDC1	ENSMMUG00000009228	73.28516054	1.379184507	0.5152	2.67699	0.0074287	0.019289516	up-regulated	protein_coding
TGDS	ENSMMUG00000013063	16.18965891	-3.347861468	1.250801	-2.67657	0.0074379	0.019309909	down-regulated	protein_coding
UFM1	ENSMMUG00000014990	23.60407994	-2.877507417	1.075553	-2.67538	0.0074645	0.019375506	down-regulated	protein_coding
FMN2	ENSMMUG00000004206	14.71277488	9.766821219	3.651948	2.674414	0.007486	0.019427633	up-regulated	protein_coding
GRK6	ENSMMUG00000019817	34.06727893	-2.551110804	0.954128	-2.67376	0.0075006	0.019461895	down-regulated	protein_coding
RINL	ENSMMUG00000023639	10.06657757	-3.700656766	1.384166	-2.67356	0.007505	0.019469807	down-regulated	protein_coding
C6H5ORF54	ENSMMUG00000023074	9.15425136	-3.561794891	1.332318	-2.67338	0.0075091	0.01947319	down-regulated	protein_coding
C11orf30	ENSMMUG00000022401	39.09919055	1.704887649	0.637746	2.673304	0.0075108	0.019474193	up-regulated	protein_coding
RPUSD1	ENSMMUG00000000842	9.89467899	-3.673180778	1.374272	-2.67282	0.0075217	0.019498772	down-regulated	protein_coding
MCF2L	ENSMMUG00000001030	9.511412307	-3.617915633	1.353988	-2.67204	0.0075391	0.019533869	down-regulated	protein_coding
C1QL3	ENSMMUG00000016064	3.737161215	4.908024456	1.836749	2.672125	0.0075372	0.019533869	up-regulated	protein_coding
KCND3	ENSMMUG00000014490	14.45700579	9.75761807	3.652084	2.671794	0.0075447	0.019544079	up-regulated	protein_coding
TBL1XR1	ENSMMUG00000019597	77.63206362	-2.056538988	0.769869	-2.67128	0.0075562	0.019570301	down-regulated	protein_coding
ZNF480	ENSMMUG00000020234	12.35328882	1.890539957	0.707981	2.670327	0.0075777	0.019618941	up-regulated	protein_coding
TEP1	ENSMMUG00000016255	475.2451462	-1.031234214	0.38624	-2.66993	0.0075868	0.019638676	down-regulated	protein_coding
DKK1	ENSMMUG00000010847	9.887890342	-3.676015444	1.377074	-2.66944	0.0075978	0.019663678	down-regulated	protein_coding
DNASE2	ENSMMUG00000028866	9.012036842	-3.539633889	1.32629	-2.66882	0.0076117	0.019696128	down-regulated	protein_coding
CHD9	ENSMMUG00000016310	101.6534043	1.098116075	0.411483	2.668678	0.007615	0.019701086	up-regulated	protein_coding
BEX4	ENSMMUG00000004150	38.26034976	-2.218433877	0.831351	-2.66847	0.0076198	0.019706224	down-regulated	protein_coding
RAB5B	ENSMMUG00000031853	41.75170946	-2.146994872	0.804574	-2.66849	0.0076194	0.019706224	down-regulated	protein_coding
ECHDC3	ENSMMUG00000001668	27.68410822	-2.503338528	0.938144	-2.6684	0.0076215	0.019706863	down-regulated	protein_coding
TFPI2	ENSMMUG00000020581	5.856874082	4.161033143	1.559455	2.668262	0.0076245	0.019711097	up-regulated	protein_coding
DENND5B	ENSMMUG00000003221	35.74665234	-3.547901269	1.329885	-2.66782	0.0076344	0.019730465	down-regulated	protein_coding
FAM3B	ENSMMUG00000004258	158.8574277	-1.460578531	0.547482	-2.66781	0.0076348	0.019730465	down-regulated	protein_coding
AEBP2	ENSMMUG00000016243	34.97390558	1.427112984	0.53504	2.6673	0.0076463	0.019756794	up-regulated	protein_coding
EED	ENSMMUG00000019621	25.12548012	-2.487769025	0.932761	-2.6671	0.0076508	0.019764699	down-regulated	protein_coding
EFNB2	ENSMMUG00000009892	136.8853039	-1.455572627	0.545907	-2.66634	0.0076682	0.019802444	down-regulated	protein_coding
SLC45A4	ENSMMUG00000013579	8.905823934	-3.524148952	1.322185	-2.6654	0.0076897	0.019847121	down-regulated	protein_coding
GLTSCR1L	ENSMMUG00000029682	31.18086658	1.88259781	0.706295	2.665457	0.0076884	0.019847121	up-regulated	protein_coding
COL13A1	ENSMMUG00000005716	14.31657018	9.734146038	3.652434	2.665112	0.0076963	0.019858812	up-regulated	protein_coding
PRPF38A	ENSMMUG00000014333	138.6429548	1.157784573	0.434428	2.665077	0.0076971	0.019858812	up-regulated	protein_coding
ATG10	ENSMMUG00000003577	14.28401359	1.678147534	0.629911	2.664101	0.0077194	0.019912943	up-regulated	protein_coding
ARPC4	ENSMMUG00000031972	105.4644269	-1.097211079	0.411905	-2.66375	0.0077275	0.019930178	down-regulated	protein_coding
TREH	ENSMMUG00000002549	289.3460701	-2.019302669	0.758445	-2.66243	0.007758	0.020005048	down-regulated	protein_coding
KIAA1949	ENSMMUG00000019961	12.72107152	1.926287722	0.723527	2.662358	0.0077595	0.020005391	up-regulated	protein_coding
FMN1	ENSMMUG00000001811	35.11052091	1.57957274	0.593413	2.661845	0.0077714	0.020032262	up-regulated	protein_coding
PTCD3	ENSMMUG00000015820	109.6554647	-1.251183887	0.470105	-2.6615	0.0077794	0.020049329	down-regulated	protein_coding
SCCPDH	ENSMMUG00000022208	44.23430447	-2.028270731	0.762159	-2.66122	0.0077859	0.020062361	down-regulated	protein_coding
SNRPC	ENSMMUG00000000869	87.04760227	-2.107670424	0.792057	-2.66101	0.0077907	0.020071019	down-regulated	protein_coding
RBM27	ENSMMUG00000004476	124.3215789	1.003163745	0.377248	2.659166	0.0078334	0.020173881	up-regulated	protein_coding
SRD5A3	ENSMMUG00000010113	32.12456653	-2.412320804	0.907194	-2.6591	0.007835	0.020174124	down-regulated	protein_coding
SNORA1	ENSMMUG00000024091	5.24753361	5.552862334	2.088805	2.658392	0.0078515	0.020212927	up-regulated	snoRNA
SIK3	ENSMMUG00000016128	119.1328411	1.405633289	0.528773	2.658294	0.0078537	0.020215121	up-regulated	protein_coding
CPSF7	ENSMMUG00000015280	101.0707883	-1.166799469	0.439128	-2.65708	0.0078821	0.020280951	down-regulated	protein_coding
PDE6D	ENSMMUG00000019565	25.59482127	-2.510037805	0.944662	-2.65708	0.0078822	0.020280951	down-regulated	protein_coding
CWC22	ENSMMUG00000020642	34.74880157	-2.011675312	0.757136	-2.65695	0.007885	0.020284553	down-regulated	protein_coding
WBP11	ENSMMUG00000019299	107.0542348	-1.15053914	0.433106	-2.65649	0.007896	0.020309056	down-regulated	protein_coding
RBM18	ENSMMUG00000011455	28.35248209	-2.239677481	0.843257	-2.65598	0.0079077	0.020335637	down-regulated	protein_coding
KCTD11	ENSMMUG00000006212	41.284693	-2.076382543	0.781862	-2.65569	0.0079147	0.020349766	down-regulated	protein_coding
CTPS2	ENSMMUG00000004797	26.32032873	-2.500423075	0.941848	-2.6548	0.0079354	0.020399442	down-regulated	protein_coding
IKBKB	ENSMMUG00000008351	66.68729321	-1.420374846	0.535438	-2.65274	0.0079842	0.02052118	down-regulated	protein_coding
MYO1B	ENSMMUG00000001761	163.6411747	-1.216857302	0.458798	-2.65227	0.0079952	0.020541848	down-regulated	protein_coding
TMEM50B	ENSMMUG00000005509	16.67961489	-3.393524702	1.279455	-2.65232	0.007994	0.020541848	down-regulated	protein_coding
GUSB	ENSMMUG00000021236	70.38999061	-1.803561689	0.680042	-2.65213	0.0079985	0.020546639	down-regulated	protein_coding
OXSR1	ENSMMUG00000000996	28.02242465	-3.192049922	1.203907	-2.65141	0.0080157	0.020582241	down-regulated	protein_coding
RNF146	ENSMMUG00000015354	78.34781339	1.07463982	0.405316	2.651364	0.0080167	0.020582241	up-regulated	protein_coding
CDKN2AIPNL	ENSMMUG00000015938	10.31205239	-3.734189005	1.408394	-2.65138	0.0080163	0.020582241	down-regulated	protein_coding
NCOR1	ENSMMUG00000002523	457.5534185	1.024127713	0.386315	2.651017	0.008025	0.020599692	up-regulated	protein_coding
TIMM9	ENSMMUG00000005788	10.62195529	-3.775494013	1.424529	-2.65035	0.008041	0.020636946	down-regulated	protein_coding
7SK	ENSMMUG00000035300	13.8757442	9.680707636	3.653252	2.649887	0.0080519	0.020661183	up-regulated	misc_RNA
MED8	ENSMMUG00000023671	24.84513989	-3.119627167	1.177298	-2.64982	0.0080535	0.020661607	down-regulated	protein_coding
CSF3R	ENSMMUG00000020434	13.69288127	9.677764617	3.653298	2.649049	0.0080719	0.02070503	up-regulated	protein_coding
SNORA1	ENSMMUG00000024245	4.601591028	4.165079024	1.572373	2.648913	0.0080751	0.0207096	up-regulated	snoRNA
ZFC3H1	ENSMMUG00000020943	208.8378894	1.137241008	0.429341	2.648806	0.0080777	0.020712404	up-regulated	protein_coding
G3BP2	ENSMMUG00000002153	163.7327115	-1.838292276	0.694551	-2.64673	0.0081273	0.020828383	down-regulated	protein_coding
F8A1	ENSMMUG00000009476	30.1545861	-2.249502752	0.849994	-2.64649	0.0081331	0.020839414	down-regulated	protein_coding

SLC22A5	ENSMMUG00000007087	38.96872916	-1.969940516	0.744464	-2.64612	0.0081421	0.020854844	down-regulated	protein_coding
CCKBR	ENSMMUG00000016884	13.71555589	9.667597426	3.653458	2.64615	0.0081414	0.020854844	up-regulated	protein_coding
STRC	ENSMMUG00000021840	107.822727	1.509680474	0.570599	2.645782	0.0081502	0.020871941	up-regulated	protein_coding
KCNJ3	ENSMMUG00000007096	40.49719782	1.524854946	0.576361	2.645661	0.0081531	0.020874499	up-regulated	protein_coding
TRIM45	ENSMMUG00000015976	16.03446657	1.899094305	0.717826	2.645619	0.0081542	0.020874499	up-regulated	protein_coding
JAK2	ENSMMUG00000014558	28.03793061	-2.577556813	0.974649	-2.6446	0.0081787	0.020925995	down-regulated	protein_coding
FGFR2	ENSMMUG00000009594	7.337155312	3.026873612	1.144889	2.643814	0.0081978	0.020970909	up-regulated	protein_coding
TMEM171	ENSMMUG00000015433	32.63821599	-2.466245897	0.932887	-2.64367	0.0082012	0.02097601	down-regulated	protein_coding
RUNX1	ENSMMUG00000001649	77.03905024	1.581684366	0.598329	2.643503	0.0082053	0.020982652	up-regulated	protein_coding
C14H11ORF83	ENSMMUG00000013950	81.63825309	-1.849153269	0.699842	-2.64224	0.0082359	0.02105313	down-regulated	protein_coding
MRPL55	ENSMMUG00000002790	28.63159602	-3.238686052	1.226529	-2.64053	0.0082776	0.021156126	down-regulated	protein_coding
MCAT	ENSMMUG00000004740	45.9530622	-2.330642613	0.883144	-2.63903	0.0083144	0.021238616	down-regulated	protein_coding
ANKRD26	ENSMMUG00000016403	16.54069005	-3.374237892	1.27858	-2.63905	0.0083138	0.021238616	down-regulated	protein_coding
KDM3B	ENSMMUG00000000455	160.287442	-1.032217858	0.391194	-2.63864	0.008324	0.021259363	down-regulated	protein_coding
GLCE	ENSMMUG00000021364	80.06020778	-2.127137371	0.806304	-2.63813	0.0083364	0.021286982	down-regulated	protein_coding
SOAT2	ENSMMUG00000020339	57.57199053	-1.678403889	0.636232	-2.63804	0.0083388	0.021289322	down-regulated	protein_coding
CAPN3	ENSMMUG00000001534	46.07575059	2.062889987	0.782112	2.637588	0.0083498	0.021309778	up-regulated	protein_coding
MPZL3	ENSMMUG00000023594	53.8417004	-1.496508276	0.567392	-2.63752	0.0083515	0.021310197	down-regulated	protein_coding
RPS19BP1	ENSMMUG00000004501	44.43074086	-2.569360211	0.974184	-2.63745	0.0083532	0.021310751	down-regulated	protein_coding
TIMM17B	ENSMMUG00000004547	133.2438169	1.317190137	0.499478	2.637132	0.008361	0.021316862	up-regulated	protein_coding
ALOX5	ENSMMUG00000013434	10.41234856	3.773917445	1.431082	2.637108	0.0083616	0.021316862	up-regulated	protein_coding
RELB	ENSMMUG00000014153	67.01644706	-1.664118131	0.631016	-2.63721	0.0083592	0.021316862	down-regulated	protein_coding
U7	ENSMMUG00000035482	3.833167569	3.916250383	1.485033	2.637147	0.0083607	0.021316862	up-regulated	snRNA
PLA2G7	ENSMMUG00000019254	4.779770393	4.87640548	1.849509	2.636594	0.0083743	0.021345309	up-regulated	protein_coding
PDGFD	ENSMMUG00000005353	3.810278787	3.68543921	1.398099	2.636036	0.0083881	0.021376618	up-regulated	protein_coding
ASCL2	ENSMMUG00000001322	12.91694741	-4.059066659	1.540534	-2.63484	0.0084176	0.021440244	down-regulated	protein_coding
CHPF	ENSMMUG00000013242	260.8997772	-1.269756057	0.481907	-2.63486	0.0084172	0.021440244	down-regulated	protein_coding
GUCY2C	ENSMMUG00000009870	114.6284855	-1.776090044	0.674134	-2.63463	0.008423	0.021450209	down-regulated	protein_coding
NXT2	ENSMMUG00000013204	25.61990796	-2.503009478	0.950124	-2.6344	0.0084285	0.021460346	down-regulated	protein_coding
RSRP1	ENSMMUG00000002970	82.08309844	-1.503900868	0.570961	-2.63398	0.008439	0.02147929	down-regulated	protein_coding
SORT1	ENSMMUG00000005812	185.3074448	-1.56397858	0.593758	-2.63403	0.0084377	0.02147929	down-regulated	protein_coding
CSNK2B	ENSMMUG00000005294	132.1119575	-1.580507298	0.600282	-2.63294	0.0084649	0.021541304	down-regulated	protein_coding
GMFB	ENSMMUG00000012326	9.193314814	-3.570815268	1.356709	-2.63197	0.0084892	0.02159534	down-regulated	protein_coding
ZNF629	ENSMMUG00000020891	41.19371962	1.921265211	0.730047	2.631699	0.0084959	0.021608593	up-regulated	protein_coding
C10H22ORF32	ENSMMUG00000013061	106.6592794	-1.79255813	0.68125	-2.63128	0.0085065	0.021631617	down-regulated	protein_coding
MPV17L2	ENSMMUG00000005025	150.5078916	1.25763871	0.477969	2.631214	0.008508	0.021631685	up-regulated	protein_coding
DNAJC13	ENSMMUG00000016005	94.43516419	-1.606424768	0.610648	-2.63069	0.0085212	0.021661213	down-regulated	protein_coding
HDAC7	ENSMMUG00000021655	8.741244494	-3.498049314	1.330015	-2.63008	0.0085364	0.021696068	down-regulated	protein_coding
GSTA4	ENSMMUG00000014899	17.26012768	-3.436445566	1.306644	-2.62998	0.008539	0.02169878	down-regulated	protein_coding
FOXN3	ENSMMUG00000019898	19.89053456	-3.660787637	1.392743	-2.62847	0.0085769	0.021791061	down-regulated	protein_coding
SGPL1	ENSMMUG00000009800	102.5312046	-2.12577771	0.808807	-2.62829	0.0085816	0.021799082	down-regulated	protein_coding
SLC16A8	ENSMMUG00000002698	4.692239833	3.709301071	1.411922	2.627128	0.0086109	0.02186525	up-regulated	protein_coding
SPATA5	ENSMMUG00000007422	9.123389807	2.048951049	0.779937	2.627074	0.0086123	0.02186525	up-regulated	protein_coding
FILIP1L	ENSMMUG00000002945	335.468067	-1.054689561	0.401583	-2.62633	0.0086312	0.021909262	down-regulated	protein_coding
C1ORF162	ENSMMUG00000003579	10.32742086	5.601137768	2.132842	2.626139	0.008636	0.021917525	up-regulated	protein_coding
NDUFA1	ENSMMUG00000032194	12.35207204	-3.993808533	1.521305	-2.62525	0.0086585	0.021970771	down-regulated	protein_coding
VWA5B2	ENSMMUG00000018949	22.30318766	2.725872894	1.039008	2.623534	0.0087023	0.022077896	up-regulated	protein_coding
FGF12	ENSMMUG00000000949	9.163486665	-3.563401759	1.358491	-2.62306	0.0087144	0.022100805	down-regulated	protein_coding
CHDH	ENSMMUG00000019266	263.3909335	-1.098739743	0.418951	-2.6226	0.0087262	0.022126657	down-regulated	protein_coding
TMEM109	ENSMMUG00000014531	60.43190761	-1.563946679	0.596439	-2.62214	0.0087379	0.022152428	down-regulated	protein_coding
UBE2Q1	ENSMMUG00000019547	95.20670814	-1.872723754	0.714376	-2.62148	0.0087549	0.022187423	down-regulated	protein_coding
FBXO21	ENSMMUG00000017479	90.52895337	-1.335194952	0.509554	-2.62032	0.0087847	0.022251169	down-regulated	protein_coding
SLC26A11	ENSMMUG00000002850	21.83376888	-2.885875042	1.102615	-2.6173	0.0088628	0.022440892	down-regulated	protein_coding
NTRK1	ENSMMUG00000012424	4.963600544	4.614320384	1.763182	2.617042	0.0088695	0.022453945	up-regulated	protein_coding
CALCOCO1	ENSMMUG00000009457	75.93920477	-1.333888904	0.509756	-2.61672	0.008878	0.022467213	down-regulated	protein_coding
U2	ENSMMUG00000035242	12.63128829	9.564121294	3.655145	2.616619	0.0088805	0.022469757	up-regulated	snRNA
IFT52	ENSMMUG00000000396	21.46541175	-2.867721268	1.09639	-2.6156	0.008907	0.022529572	down-regulated	protein_coding
KIF17	ENSMMUG00000008837	21.83321635	2.868841809	1.096824	2.615589	0.0089074	0.022529572	up-regulated	protein_coding
ZNF17	ENSMMUG00000014282	8.439649223	-3.446385582	1.31788	-2.6151	0.0089202	0.02255778	down-regulated	protein_coding
TMX3	ENSMMUG00000017728	30.0852821	-2.351869387	0.899363	-2.61504	0.0089217	0.02255778	down-regulated	protein_coding
YPEL5	ENSMMUG00000006615	73.72877137	-1.499155808	0.573323	-2.61485	0.0089266	0.022566116	down-regulated	protein_coding
SOX9	ENSMMUG00000002412	736.2417982	1.09970297	0.420687	2.614066	0.0089472	0.022614073	up-regulated	protein_coding
CHD6	ENSMMUG00000020458	63.69076544	1.38501125	0.529989	2.613284	0.0089677	0.022657771	up-regulated	protein_coding
CIDEC	ENSMMUG00000010350	9.375031322	-3.596316062	1.376378	-2.61288	0.0089782	0.022676279	down-regulated	protein_coding
SNTB2	ENSMMUG00000016640	22.77065527	1.594214584	0.610131	2.612904	0.0089777	0.022676279	up-regulated	protein_coding
USP49	ENSMMUG00000012421	5.329547114	2.684340472	1.027728	2.611918	0.0090036	0.022728231	up-regulated	protein_coding
KALRN	ENSMMUG00000016672	212.0096994	1.110571692	0.425329	2.611091	0.0090254	0.022779199	up-regulated	protein_coding
CAAP1	ENSMMUG00000006073	22.50235288	-2.874208843	1.100823	-2.61096	0.0090288	0.022783636	down-regulated	protein_coding
ZNF131	ENSMMUG00000014251	16.25652017	-3.345694796	1.281484	-2.6108	0.0090332	0.022790718	down-regulated	protein_coding
ASNA1	ENSMMUG00000006275	78.89474343	-1.809808101	0.693224	-2.61071	0.0090354	0.022792239	down-regulated	protein_coding
ATP13A2	ENSMMUG00000001217	646.4563806	-1.284001217	0.49198	-2.60987	0.0090578	0.022844634	down-regulated	protein_coding
SHOX2	ENSMMUG00000019030	11.57799393	9.424899649	3.612046	2.609297	0.0090729	0.022878605	up-regulated	protein_coding
SLC25A35	ENSMMUG00000013544	15.07250411	2.448502267	0.938591	2.6087	0.0090887	0.022914466	up-regulated	protein_coding
PYCRL	ENSMMUG00000014666	37.75991004	-2.359700539	0.904574	-2.60863	0.0090905	0.022914989	down-regulated	protein_coding
PPCS	ENSMMUG00000013126	92.14182963	-1.325382315	0.508157	-2.60822	0.0091016	0.022934889	down-regulated	protein_coding
KLK10	ENSMMUG00000015451	9.333200996	2.145854055	0.82273	2.608212	0.0091017	0.022934889	up-regulated	protein_coding
MAP7	ENSMMUG00000016373	212.295215	1.043799471	0.400389	2.606966	0.0091348	0.023014396	up-regulated	protein_coding
MYH1	ENSMMUG00000009774	6.175148289	3.969657527	1.522824	2.606773	0.00914	0.023023272	up-regulated	protein_coding
VPS13B	ENSMMUG00000022186	105.5350098	1.383548367	0.530958	2.605757	0.0091671	0.023087541	up-regulated	protein_coding
SAAL1	ENSMMUG00000014377	24.00943407	-3.035330753	1.165019	-2.60539	0.0091769	0.023108077	down-regulated	protein_coding
AANAT	ENSMMUG00000018970	6.0743272	3.805109303	1.460698	2.604994	0.0091876	0.023130792	up-regulated	protein_coding
KCNJ11	ENSMMUG00000023580	6.184792788	3.435860775	1.31903	2.604839	0.0091917	0.023133044	up-regulated	protein_coding
PYCR1	ENSMMUG00000012895	18.07046706	-3.512489949	1.348678	-2.60439	0.0092037	0.023158992	down-regulated	protein_coding
KCND1	ENSMMUG00000004559	16.07849009	2.320846813	0.891221	2.604121	0.009211	0.023173317	up-regulated	protein_coding
TXLNA	ENSMMUG00000015580	55.38953886	1.146950292	0.440474	2.603902	0.0092169	0.023179859	up-regulated	protein_coding
NRBF2	ENSMMUG00000017726	45.94651628	-1.813054753	0.696274	-2.60394	0.0092159	0.023179859	down-regulated	protein_coding
PHEX	ENSMMUG00000023589	10.84780667	2.814110042	1.0808	2.603729	0.0092216	0.023187445	up-regulated	protein_coding
CARS2	ENSMMUG00000012666	132.1106842	1.367558195	0.525421	2.602785	0.009247	0.02324317	up-regulated	protein_coding
FH	ENSMMUG00000005667	107.7360605	-1.409687731	0.541649	-2.60259	0.0092524	0.023252499	down-regulated	protein_coding
ALDH1A3	ENSMMUG00000006659	107.6486194	-2.400226436	0.922346	-2.60231	0.0092599	0.023267287	down-regulated	protein_coding
PTGS1	ENSMMUG00000012886	113.2529222	-1.971708107	0.75775	-2.60206	0.0092666	0.023280148	down-regulated	protein_coding
EIF1B	ENSMMUG00000017704	41.52153654	-1.709911233	0.657388	-2.60107	0.0092934	0.023343146	down-regulated	protein_coding
ARTN	ENSMMUG00000015565	27.10246174	-2.639230876	1.014971	-2.6003	0.0093142	0.023391268	down-regulated	protein_coding
KNTC1	ENSMMUG00000007556	9.850464011	-3.667184283	1.410399	-2.6001	0.0093196	0.023399768	down-regulated	protein_coding
C15orf39	ENSMMUG00000008529	84.74464639	-1.310746769	0.504123	-2.60005	0.0093209	0.023399768	down-regulated	protein_coding
IPMK	ENSMMUG00000019822	18.06602614	-3.509360458	1.349757	-2.59999	0.0093225	0.023399768	down-regulated	protein_coding
TSNARE1	ENSMMUG00000001432	8.049266746	2.983394136	1.147603	2.599674	0.0093312	0.02341749	up-regulated	protein_coding
RPS21	ENSMMUG00000005006	194.4067772	-1.918806511	0.738512	-2.59821	0.0093712	0.023513606	down-regulated	protein_coding
NAPRT	ENSMMUG00000001641	169.0272886	-1.463028927	0.563174	-2.59783	0.0093816	0.023535546	down-regulated	protein_coding
ATF7IP	ENSMMUG00000003664	33.29879242	-2.11609992	0.814637	-2.5976	0.0093879	0.023542859	down-regulated	protein_coding

MTHFSD	ENSMMUG00000019500	10.00267265	-3.689132028	1.420195	-2.59762	0.0093871	0.023542859	down-regulated	protein_coding
C17ORF85	ENSMMUG00000008555	26.78092997	-2.130260715	0.820138	-2.59744	0.0093921	0.023549285	down-regulated	protein_coding
RCN1	ENSMMUG00000018360	17.22517429	-3.439504714	1.324286	-2.59725	0.0093973	0.023558116	down-regulated	protein_coding
C7ORF47	ENSMMUG00000012728	42.31964156	-2.168599692	0.835006	-2.59711	0.0094013	0.023563971	down-regulated	protein_coding
ZNF585A	ENSMMUG00000000107	8.465959278	-3.451972132	1.329615	-2.59622	0.0094256	0.023620708	down-regulated	protein_coding
MON1A	ENSMMUG00000031811	107.1083778	1.277352042	0.492106	2.595683	0.0094403	0.023653439	up-regulated	protein_coding
NSUN2	ENSMMUG00000002561	63.23165477	-1.638604191	0.631347	-2.59541	0.0094479	0.023668156	down-regulated	protein_coding
ABCA7	ENSMMUG00000015303	69.8632213	-1.704891424	0.65697	-2.59508	0.0094568	0.023686452	down-regulated	protein_coding
DROSHA	ENSMMUG00000012193	134.3077388	1.618386316	0.623719	2.594735	0.0094664	0.023706199	up-regulated	protein_coding
TOR3A	ENSMMUG00000006261	91.59666259	1.192823607	0.460014	2.593018	0.0095138	0.023819498	up-regulated	protein_coding
TAPBPL	ENSMMUG00000014266	111.399422	1.260060056	0.486019	2.592614	0.009525	0.023840206	up-regulated	protein_coding
CYB561A3	ENSMMUG00000022372	45.66178808	1.447332295	0.558311	2.592342	0.0095325	0.023854815	up-regulated	protein_coding
TNFSF10	ENSMMUG00000019302	104.3915799	-1.012868549	0.390773	-2.59196	0.0095431	0.023873009	down-regulated	protein_coding
EARS2	ENSMMUG00000020525	8.713674798	-3.490088458	1.346506	-2.59196	0.0095431	0.023873009	down-regulated	protein_coding
POLR2I	ENSMMUG00000000522	61.98685147	1.645049344	0.634695	2.591873	0.0095455	0.023874692	up-regulated	protein_coding
KCTD20	ENSMMUG00000014508	65.04885342	1.287996696	0.497095	2.591046	0.0095685	0.023919475	up-regulated	protein_coding
SSSCA1	ENSMMUG00000017162	21.46674931	-3.723115643	1.43689	-2.59109	0.0095671	0.023919475	down-regulated	protein_coding
ANAPC2	ENSMMUG00000001669	85.75771399	-1.695016018	0.654263	-2.59073	0.0095773	0.023937402	down-regulated	protein_coding
IGSF1	ENSMMUG00000011152	11.86716377	9.47211999	3.656749	2.590311	0.0095889	0.023953662	up-regulated	protein_coding
SH3TC2	ENSMMUG00000019820	13.20775999	2.022476978	0.780757	2.590404	0.0095863	0.023953662	up-regulated	protein_coding
5S_rRNA	ENSMMUG00000033157	11.86716377	9.47211999	3.656749	2.590311	0.0095889	0.023953662	up-regulated	rRNA
RNF4	ENSMMUG00000007948	24.72534614	-2.480968788	0.95795	-2.58987	0.0096012	0.023980019	down-regulated	protein_coding
PNPLA8	ENSMMUG00000011831	33.24515179	-2.114192453	0.816757	-2.58852	0.0096389	0.024065887	down-regulated	protein_coding
CMTM4	ENSMMUG00000020848	79.91095992	1.686714303	0.651789	2.587821	0.0096585	0.024110499	up-regulated	protein_coding
DVL3	ENSMMUG00000006553	102.5206873	-1.12943321	0.436532	-2.58728	0.0096736	0.024143831	down-regulated	protein_coding
PPFIA4	ENSMMUG00000000063	9.994177283	2.496610737	0.965116	2.586852	0.0096857	0.024169912	up-regulated	protein_coding
RHBDD3	ENSMMUG00000021373	39.69329534	-2.435710866	0.941828	-2.58615	0.0097054	0.024209954	down-regulated	protein_coding
LYRM4	ENSMMUG00000005467	11.85550161	-3.93400561	1.52138	-2.58581	0.009715	0.024225753	down-regulated	protein_coding
SSFA2	ENSMMUG00000014277	575.7943465	-1.14644776	0.443422	-2.58546	0.009725	0.024239354	down-regulated	protein_coding
CREM	ENSMMUG00000022508	8.595635361	-3.471799026	1.342812	-2.58547	0.0097246	0.024239354	down-regulated	protein_coding
BNIP3L	ENSMMUG00000023646	225.3861707	-1.584279217	0.61277	-2.58544	0.0097256	0.024239354	down-regulated	protein_coding
HOXA3	ENSMMUG00000012813	12.58094624	2.377904693	0.920588	2.583028	0.0097937	0.024396436	up-regulated	protein_coding
FAM3C	ENSMMUG00000002440	94.75760416	-1.774690556	0.687123	-2.58278	0.0098007	0.024409446	down-regulated	protein_coding
ARL14EP	ENSMMUG00000017590	15.16459673	-3.240481068	1.254983	-2.58209	0.0098204	0.024454153	down-regulated	protein_coding
CDH13	ENSMMUG00000020114	6.562801235	3.720464112	1.441128	2.581634	0.0098334	0.024482241	up-regulated	protein_coding
NHLRC3	ENSMMUG00000002113	55.06161605	1.009113447	0.390934	2.58129	0.0098432	0.024498031	up-regulated	protein_coding
ERGIC3	ENSMMUG00000023456	329.0964875	-1.162058878	0.450177	-2.58134	0.0098419	0.024498031	down-regulated	protein_coding
PFDN1	ENSMMUG00000003279	99.57022424	-1.595135354	0.618166	-2.58043	0.0098677	0.024550311	down-regulated	protein_coding
TMEM261	ENSMMUG00000016624	71.68982087	-2.242491321	0.869359	-2.57948	0.009895	0.024614091	down-regulated	protein_coding
C10ORF10	ENSMMUG00000017490	49.73459588	2.229686388	0.86443	2.579373	0.009898	0.024617072	up-regulated	protein_coding
NUCKS1	ENSMMUG00000005645	202.6855601	1.045592857	0.405383	2.579269	0.009901	0.02462017	up-regulated	protein_coding
ADCY6	ENSMMUG00000006253	198.5431436	-1.12363703	0.435844	-2.57807	0.0099354	0.024688374	down-regulated	protein_coding
GNAO1	ENSMMUG00000019900	7.726558846	4.548971412	1.765098	2.577177	0.0099611	0.024743569	up-regulated	protein_coding
JPH1	ENSMMUG00000022325	21.12770526	2.144066784	0.831942	2.577182	0.0099609	0.024743569	up-regulated	protein_coding
MRPL50	ENSMMUG00000023794	26.13082355	-2.991350022	1.160864	-2.57683	0.009971	0.024763966	down-regulated	protein_coding
KIAA0586	ENSMMUG00000001084	8.289074133	-3.418935956	1.326863	-2.57671	0.0099747	0.024764272	down-regulated	protein_coding
THTPA	ENSMMUG00000004142	79.9568639	1.100061683	0.426926	2.576706	0.0099747	0.024764272	up-regulated	protein_coding
CSRP2	ENSMMUG00000018257	21.06525463	1.57729058	0.612265	2.576158	0.0099905	0.024794895	up-regulated	protein_coding
KLHDC5	ENSMMUG00000021742	44.3582628	1.755392746	0.681388	2.576201	0.0099893	0.024794895	up-regulated	protein_coding
SEPT10	ENSMMUG00000017177	27.24567775	-2.152935272	0.835816	-2.57585	0.0099995	0.024812838	down-regulated	protein_coding
C15orf43	ENSMMUG00000017130	9.803028102	3.864187286	1.500299	2.575611	0.0100063	0.024825424	up-regulated	protein_coding
SLK	ENSMMUG00000015324	214.6197535	-1.056475785	0.410234	-2.5753	0.0100153	0.024843284	down-regulated	protein_coding
AFAP1L2	ENSMMUG00000022523	3.750894023	3.916337991	1.520858	2.575084	0.0100216	0.024854566	up-regulated	protein_coding
CHORDC1	ENSMMUG00000005209	24.68162996	-2.478437492	0.962575	-2.5748	0.0100298	0.024867508	down-regulated	protein_coding
RALGAPA1	ENSMMUG00000016487	47.37876863	-1.963284013	0.762505	-2.57478	0.0100303	0.024867508	down-regulated	protein_coding
CHP1	ENSMMUG00000017092	131.3929787	-1.539079665	0.597766	-2.57472	0.0100321	0.02486768	down-regulated	protein_coding
ZNF169	ENSMMUG00000015805	8.892732226	2.142928302	0.832432	2.574299	0.0100444	0.024893595	up-regulated	protein_coding
GPR65	ENSMMUG00000017260	7.837766003	5.485947538	2.131198	2.574114	0.0100497	0.024902494	up-regulated	protein_coding
FERMT2	ENSMMUG00000010689	16.27666856	2.962646727	1.151203	2.573522	0.0100669	0.02494079	up-regulated	protein_coding
AKAP13	ENSMMUG00000011251	163.420326	1.291323039	0.501795	2.573408	0.0100702	0.024944643	up-regulated	protein_coding
KIF13B	ENSMMUG00000023565	395.7038419	-1.093999167	0.425162	-2.57313	0.0100783	0.024960182	down-regulated	protein_coding
BIK	ENSMMUG00000004739	48.10492583	1.640844943	0.63771	2.573026	0.0100814	0.024963458	up-regulated	protein_coding
C1ORF27	ENSMMUG00000009085	14.99376192	-3.230486502	1.255717	-2.57262	0.0100931	0.024988144	down-regulated	protein_coding
OFD1	ENSMMUG00000019410	42.18996893	-1.753099528	0.681521	-2.57233	0.0101015	0.025004663	down-regulated	protein_coding
SLC12A8	ENSMMUG00000001169	25.42252507	-3.13041707	1.217091	-2.57205	0.0101099	0.025020876	down-regulated	protein_coding
SLC9B2	ENSMMUG00000019083	3.672826283	3.938224929	1.531413	2.571628	0.0101222	0.025044633	up-regulated	protein_coding
MRPL44	ENSMMUG00000006160	33.95065335	-1.850127161	0.719474	-2.5715	0.0101259	0.025047522	down-regulated	protein_coding
ARHGAP23	ENSMMUG00000020020	18.87143593	2.219760341	0.86331	2.571222	0.010134	0.025063192	up-regulated	protein_coding
PAICS	ENSMMUG00000016366	37.41133003	-2.257350427	0.877976	-2.57109	0.010138	0.025068698	down-regulated	protein_coding
FOXA1	ENSMMUG00000009653	63.81986386	-1.394247013	0.542542	-2.56984	0.0101745	0.025154478	down-regulated	protein_coding
ZNF267	ENSMMUG00000008253	8.333554887	-3.426675929	1.334111	-2.56851	0.0102137	0.025247059	down-regulated	protein_coding
KLHL10	ENSMMUG00000005961	3.885979558	4.004763832	1.559252	2.568387	0.0102173	0.025251448	up-regulated	protein_coding
GFM2	ENSMMUG00000019998	44.94221919	1.070235612	0.416781	2.567862	0.0102328	0.025285289	up-regulated	protein_coding
KIRREL2	ENSMMUG00000017051	4.573543824	4.670777257	1.819043	2.567711	0.0102372	0.025291897	up-regulated	protein_coding
PROC	ENSMMUG00000023563	3.973305878	3.986550805	1.553441	2.566271	0.0102799	0.025388328	up-regulated	protein_coding
ZNRF2	ENSMMUG00000012541	33.54999503	-1.949971442	0.75998	-2.56582	0.0102932	0.025415268	down-regulated	protein_coding
SSTR3	ENSMMUG00000018853	18.3299285	9.359451698	3.647797	2.565782	0.0102944	0.025415268	up-regulated	protein_coding
NTN1	ENSMMUG00000009397	50.14596367	1.457237366	0.567968	2.565702	0.0102967	0.02541669	up-regulated	protein_coding
CCDC94	ENSMMUG00000010100	8.434413787	-3.444315611	1.34252	-2.56556	0.0103009	0.025422604	down-regulated	protein_coding
DNTTIP2	ENSMMUG00000008435	54.42442013	-1.40937689	0.549848	-2.56321	0.0103709	0.025568605	down-regulated	protein_coding
FERMT3	ENSMMUG00000012939	18.74018254	1.782659687	0.695469	2.563248	0.0103698	0.025568605	up-regulated	protein_coding
C9orf89	ENSMMUG00000013692	32.56817707	-1.963248088	0.765885	-2.56337	0.0103661	0.025568605	down-regulated	protein_coding
AGPAT3	ENSMMUG00000014892	165.4996231	-1.482160042	0.578229	-2.56327	0.010369	0.025568605	down-regulated	protein_coding
XYLT1	ENSMMUG00000017131	36.65467768	2.263694601	0.883078	2.563415	0.0103648	0.025568605	up-regulated	protein_coding
PIGP	ENSMMUG00000016211	8.304944668	-3.423877101	1.335925	-2.56293	0.0103794	0.025577499	down-regulated	protein_coding
RPS27L	ENSMMUG00000016350	171.0073138	-1.459782835	0.569581	-2.56291	0.01038	0.025577499	down-regulated	protein_coding
EPB41L2	ENSMMUG00000007758	168.5981917	-1.084968913	0.423412	-2.56244	0.0103939	0.025602849	down-regulated	protein_coding
PNPLA3	ENSMMUG00000016698	19.11304193	-3.593955123	1.402714	-2.56214	0.0104028	0.025620434	down-regulated	protein_coding
MED9	ENSMMUG00000006491	20.82720497	-2.833687806	1.106182	-2.56168	0.0104167	0.025645602	down-regulated	protein_coding
NAA30	ENSMMUG00000021311	36.68644993	-1.733784424	0.676878	-2.56144	0.0104239	0.025658941	down-regulated	protein_coding
CPEB3	ENSMMUG00000015050	15.98465093	2.854047237	1.114588	2.56063	0.0104482	0.025714417	up-regulated	protein_coding
TMEM110	ENSMMUG00000014526	31.55024381	1.45114744	0.566809	2.560203	0.0104611	0.025741558	up-regulated	protein_coding
AP2B1	ENSMMUG00000006048	139.2985161	-1.858100752	0.725781	-2.56014	0.010463	0.025741788	down-regulated	protein_coding
DDIT4	ENSMMUG00000012299	199.8970947	-1.250536549	0.488604	-2.55941	0.0104851	0.025791585	down-regulated	protein_coding
NMRK2	ENSMMUG00000015273	10.95430502	9.357142086	3.658902	2.557363	0.0105469	0.025930155	up-regulated	protein_coding
U6	ENSMMUG00000027811	10.95430502	9.357142086	3.658902	2.557363	0.0105469	0.025930155	up-regulated	snRNA
MANEA	ENSMMUG00000012202	18.1044217	-3.512584975	1.373964	-2.55653	0.0105721	0.025987549	down-regulated	protein_coding
DBNDD2	ENSMMUG00000003409	55.8752121	-1.609637881	0.629811	-2.55575	0.010596	0.026041728	down-regulated	protein_coding
ENDOG	ENSMMUG00000003294	112.3473336	1.937376029	0.758145	2.555417	0.0106061	0.026062066	up-regulated	protein_coding
PTPN9	ENSMMUG00000003783	21.9292444	-2.821031677	1.104509	-2.5541	0.0106462	0.026151461	down-regulated	protein_coding

CDC42EP2	ENSMMUG00000012316	39.75711641	-2.108497831	0.825639	-2.55378	0.0106561	0.026171457	down-regulated	protein_coding
CLIC3	ENSMMUG00000021431	17.17540374	2.505925926	0.981395	2.553433	0.0106667	0.026192786	up-regulated	protein_coding
THUMPD1	ENSMMUG00000004225	31.14870861	1.32571896	0.519585	2.551498	0.0107261	0.026334127	up-regulated	protein_coding
PKIA	ENSMMUG00000018611	8.643008891	-3.482598362	1.365096	-2.55117	0.0107361	0.026353988	down-regulated	protein_coding
TMEM159	ENSMMUG00000016758	9.71269004	-3.647969824	1.430027	-2.55098	0.0107421	0.02636416	down-regulated	protein_coding
NCOA7	ENSMMUG00000004290	48.81558042	-1.948675134	0.764016	-2.55057	0.0107547	0.026386133	down-regulated	protein_coding
ZCCHC9	ENSMMUG00000020655	44.16657516	-2.026894162	0.794827	-2.55011	0.010769	0.026416417	down-regulated	protein_coding
CD44	ENSMMUG00000004327	597.6123555	1.732138553	0.679793	2.548038	0.0108331	0.026564449	up-regulated	protein_coding
EFCAB6	ENSMMUG00000005769	6.283470219	2.814608215	1.105054	2.547032	0.0108643	0.026631947	up-regulated	protein_coding
SCARB2	ENSMMUG00000020913	248.2046666	-1.352737081	0.531103	-2.54703	0.0108643	0.026631947	down-regulated	protein_coding
EVA1B	ENSMMUG00000011689	60.48081449	1.450150155	0.569501	2.546351	0.0108856	0.026674969	up-regulated	protein_coding
IL34	ENSMMUG00000020415	27.73819819	-3.30964779	1.299763	-2.54635	0.0108857	0.026674969	down-regulated	protein_coding
CEP164	ENSMMUG00000007791	38.6511627	1.27225283	0.499651	2.546283	0.0108877	0.026675304	up-regulated	protein_coding
ACAD9	ENSMMUG00000006338	60.8439804	-1.852138529	0.727412	-2.5462	0.0108902	0.026676724	down-regulated	protein_coding
ATP9B	ENSMMUG00000021792	16.80889584	-3.398353095	1.335149	-2.5453	0.0109184	0.026741402	down-regulated	protein_coding
TMUB2	ENSMMUG00000019920	44.65976754	-1.485698826	0.583767	-2.54502	0.0109272	0.026758213	down-regulated	protein_coding
FBXL6	ENSMMUG00000007370	77.85161944	-1.925804958	0.757034	-2.54388	0.0109628	0.026836021	down-regulated	protein_coding
MRPS21	ENSMMUG00000021391	47.32734991	-2.708954246	1.065004	-2.54361	0.0109714	0.02685249	down-regulated	protein_coding
ITGA4	ENSMMUG00000000300	11.41635841	3.577803181	1.408037	2.540987	0.011054	0.027049974	up-regulated	protein_coding
MIER1	ENSMMUG00000000643	39.04158554	-2.134975295	0.840262	-2.54085	0.0110585	0.027056227	down-regulated	protein_coding
TLE4	ENSMMUG00000023365	24.34309454	1.351245448	0.531855	2.540625	0.0110654	0.02706864	up-regulated	protein_coding
PNKD	ENSMMUG00000022819	63.99239881	-1.577295006	0.620845	-2.54056	0.0110675	0.027068885	down-regulated	protein_coding
C6orf132	ENSMMUG00000007771	47.81735226	1.413216771	0.556276	2.540497	0.0110695	0.027069185	up-regulated	protein_coding
PKHD1	ENSMMUG00000016993	7.420746275	3.125360153	1.230334	2.540254	0.0110772	0.027081216	up-regulated	protein_coding
CDK2	ENSMMUG00000018817	43.99649267	-2.298378736	0.904795	-2.54022	0.0110782	0.027081216	down-regulated	protein_coding
TIMM17A	ENSMMUG00000001924	77.8681829	-1.647720966	0.648749	-2.53984	0.0110903	0.027105911	down-regulated	protein_coding
HDHD3	ENSMMUG00000007187	88.69925979	-1.419119404	0.558869	-2.53927	0.0111084	0.027131536	down-regulated	protein_coding
RAB11B	ENSMMUG00000010225	161.0144468	-1.136856865	0.447814	-2.53868	0.0111272	0.027172611	down-regulated	protein_coding
FGD3	ENSMMUG00000020795	75.67534992	1.625451599	0.64029	2.538619	0.0111291	0.027172661	up-regulated	protein_coding
GCC1	ENSMMUG00000015421	29.49640036	-1.908920784	0.751995	-2.53848	0.0111337	0.027179129	down-regulated	protein_coding
TDP1	ENSMMUG00000004460	9.083014485	-3.549765738	1.399418	-2.5366	0.0111935	0.027315663	down-regulated	protein_coding
PUM2	ENSMMUG00000023181	207.3053865	-1.097438553	0.432673	-2.53642	0.0111993	0.027325247	down-regulated	protein_coding
COG7	ENSMMUG00000000525	50.47529296	-1.371142584	0.54064	-2.53615	0.0112079	0.027336853	down-regulated	protein_coding
EIF4EBP1	ENSMMUG00000002342	26.77864134	-2.593743068	1.02271	-2.53615	0.0112079	0.027336853	down-regulated	protein_coding
BDH1	ENSMMUG00000002021	36.15112672	-2.047974303	0.808019	-2.53456	0.0112588	0.027456254	down-regulated	protein_coding
CASP10	ENSMMUG00000031297	22.4158982	-2.930656812	1.156395	-2.5343	0.0112671	0.027471625	down-regulated	protein_coding
APOOL	ENSMMUG00000004335	40.17589704	-1.702013973	0.67184	-2.53336	0.0112974	0.027531323	down-regulated	protein_coding
TMCC2	ENSMMUG00000010480	4.070572237	3.647935516	1.440995	2.531539	0.0113563	0.027660593	up-regulated	protein_coding
PLXNB3	ENSMMUG00000003368	52.89197275	-1.850718509	0.731172	-2.53117	0.0113684	0.02768527	down-regulated	protein_coding
CASP8	ENSMMUG00000031296	33.30938178	-1.799893869	0.711123	-2.53106	0.0113718	0.027688891	down-regulated	protein_coding
GRTP1	ENSMMUG00000009791	42.52506009	-1.793571694	0.709297	-2.52866	0.0114499	0.027863237	down-regulated	protein_coding
TMCC1	ENSMMUG00000016793	26.0066134	-2.533571305	1.00196	-2.52862	0.0114513	0.027863237	down-regulated	protein_coding
FANCF	ENSMMUG00000020817	48.9407474	1.565414207	0.619197	2.528136	0.011467	0.027896554	up-regulated	protein_coding
LZTR1	ENSMMUG00000020482	55.11136353	-1.274371734	0.504155	-2.52774	0.01148	0.027923514	down-regulated	protein_coding
RNF169	ENSMMUG00000021038	19.77859418	1.775240572	0.702411	2.527352	0.0114926	0.027949333	up-regulated	protein_coding
C11H12ORF10	ENSMMUG00000022034	17.55457212	-3.462250917	1.370318	-2.5266	0.0115171	0.028004147	down-regulated	protein_coding
PSMD6	ENSMMUG00000001509	102.6169438	-1.189824371	0.471175	-2.52523	0.0115624	0.028104516	down-regulated	protein_coding
HPGDS	ENSMMUG00000022244	14.6251717	4.577599115	1.812986	2.524895	0.0115733	0.028126119	up-regulated	protein_coding
FAM54A	ENSMMUG00000003709	8.948897549	-3.528101196	1.397641	-2.52433	0.011592	0.028166862	down-regulated	protein_coding
MMS19	ENSMMUG00000020030	35.27627246	1.511590503	0.599	2.523522	0.0116186	0.028221667	up-regulated	protein_coding
GCLM	ENSMMUG00000013857	68.56534583	-1.39538102	0.553023	-2.52319	0.0116296	0.028243549	down-regulated	protein_coding
USP10	ENSMMUG00000011244	133.4739441	-1.079798654	0.427971	-2.52306	0.0116338	0.028248869	down-regulated	protein_coding
PPIG	ENSMMUG00000020512	75.00657884	-1.051813096	0.416905	-2.52291	0.0116388	0.028256204	down-regulated	protein_coding
SPATA6L	ENSMMUG00000000136	9.438524563	2.432980015	0.964507	2.522511	0.011652	0.02828016	up-regulated	protein_coding
ALS2CR11	ENSMMUG00000018144	3.988561443	3.908576033	1.54949	2.522492	0.0116526	0.02828016	up-regulated	protein_coding
U6	ENSMMUG00000027459	8.233510216	-3.408413731	1.351484	-2.52198	0.0116697	0.028316658	down-regulated	snRNA
KATNBL1	ENSMMUG00000000013	35.07684879	-1.946518345	0.771847	-2.5219	0.0116724	0.028318498	down-regulated	protein_coding
COPS7B	ENSMMUG00000022831	92.95291969	1.386071601	0.549699	2.521512	0.0116852	0.028344464	up-regulated	protein_coding
SPATA20	ENSMMUG00000017252	30.24502051	-2.193724456	0.871032	-2.51853	0.0117845	0.028575558	down-regulated	protein_coding
WIPI1	ENSMMUG00000007309	66.78647174	-1.655358572	0.657436	-2.5179	0.0118057	0.028617275	down-regulated	protein_coding
CCDC24	ENSMMUG00000015156	11.88291689	2.337362272	0.92837	2.517707	0.0118122	0.028628016	up-regulated	protein_coding
PDP1	ENSMMUG00000004568	51.53142867	-1.929657758	0.766546	-2.51734	0.0118245	0.028644254	down-regulated	protein_coding
CASP9	ENSMMUG00000018163	45.1129781	-1.792368532	0.712013	-2.51733	0.0118249	0.028644254	down-regulated	protein_coding
MVD	ENSMMUG00000014326	332.4534093	-1.744523438	0.693099	-2.51699	0.0118362	0.028656705	down-regulated	protein_coding
FADD	ENSMMUG00000019735	41.6370048	-1.659501859	0.659308	-2.51704	0.0118346	0.028656705	down-regulated	protein_coding
IFNAR2	ENSMMUG00000003956	53.82927682	-1.448781792	0.575701	-2.51655	0.0118509	0.028687383	down-regulated	protein_coding
ZNF850	ENSMMUG00000028781	10.2513039	2.744275532	1.090844	2.515736	0.0118784	0.028749136	up-regulated	protein_coding
CNOT2	ENSMMUG00000014853	52.71591822	-1.355925257	0.539197	-2.51471	0.011913	0.028823112	down-regulated	protein_coding
MARCH2	ENSMMUG00000002252	23.73523035	-2.388485238	0.949966	-2.51428	0.0119274	0.028848039	down-regulated	protein_coding
ANK3	ENSMMUG00000003589	71.31929174	-1.441990384	0.573754	-2.51326	0.0119622	0.028922329	down-regulated	protein_coding
SLC15A2	ENSMMUG00000001049	7.056558655	3.549293852	1.412301	2.513128	0.0119666	0.028927923	up-regulated	protein_coding
STRA13	ENSMMUG00000010890	19.63378323	-3.637568771	1.447479	-2.51304	0.0119697	0.028930503	down-regulated	protein_coding
RTN4R	ENSMMUG00000011547	16.32587889	-3.292988833	1.310862	-2.51208	0.0120022	0.029004202	down-regulated	protein_coding
DCP2	ENSMMUG00000013553	28.04773687	1.44764082	0.576288	2.51201	0.0120046	0.029004875	up-regulated	protein_coding
ALG3	ENSMMUG00000018951	38.19524242	-1.874238176	0.746156	-2.51186	0.0120098	0.02901253	down-regulated	protein_coding
ZNF627	ENSMMUG00000001715	8.011194973	-3.372641518	1.342764	-2.51172	0.0120146	0.029018199	down-regulated	protein_coding
NUDT14	ENSMMUG00000004687	42.27755662	-2.254366407	0.897558	-2.51167	0.0120162	0.029018199	down-regulated	protein_coding
JDP2	ENSMMUG00000002797	21.65488072	1.729036966	0.688658	2.510735	0.012048	0.029088968	up-regulated	protein_coding
AIDA	ENSMMUG00000015025	44.41360115	-1.798368368	0.716285	-2.51069	0.0120496	0.029088968	down-regulated	protein_coding
FGFBP1	ENSMMUG00000008162	73.36301082	-1.63846334	0.652679	-2.51037	0.0120606	0.029110516	down-regulated	protein_coding
UGT8	ENSMMUG00000014456	114.5017369	-1.361727121	0.542468	-2.51024	0.0120648	0.029110701	down-regulated	protein_coding
MICALCL	ENSMMUG00000020955	21.98904916	1.750591184	0.697373	2.510265	0.0120641	0.029110701	up-regulated	protein_coding
EMC4	ENSMMUG00000012335	33.01655711	-1.917601313	0.763972	-2.51004	0.0120717	0.029122349	down-regulated	protein_coding
SPOPL	ENSMMUG00000002560	26.40916327	-2.477430135	0.987137	-2.50971	0.0120829	0.029144476	down-regulated	protein_coding
SLC23A1	ENSMMUG00000001002	33.39821048	1.653253599	0.658982	2.508798	0.0121143	0.029214612	up-regulated	protein_coding
IPO5	ENSMMUG00000013531	118.6400621	-1.964641669	0.783118	-2.50874	0.0121161	0.029214612	down-regulated	protein_coding
RRP7A	ENSMMUG00000009716	53.04922827	-1.667445564	0.664872	-2.50792	0.0121444	0.029272864	down-regulated	protein_coding
ZNF559	ENSMMUG00000018884	8.377019833	-3.433773414	1.369244	-2.50779	0.012149	0.029278802	down-regulated	protein_coding
APPBP2	ENSMMUG00000016566	22.22397651	-2.762374591	1.101808	-2.50713	0.0121717	0.029328458	down-regulated	protein_coding
METTL6	ENSMMUG00000004933	14.75031408	-3.20415081	1.278089	-2.50699	0.0121766	0.029335309	down-regulated	protein_coding
POLD4	ENSMMUG00000004523	194.2705415	-1.053354134	0.420249	-2.5065	0.0121934	0.029360821	down-regulated	protein_coding
PDZD3	ENSMMUG00000012190	235.3712893	-1.14817833	0.458074	-2.50654	0.012192	0.029360821	down-regulated	protein_coding
EPHX2	ENSMMUG00000021515	205.3590723	-1.889874709	0.754705	-2.50412	0.0122756	0.029548629	down-regulated	protein_coding
SLC22A17	ENSMMUG00000014748	8.536257636	-3.463989508	1.383353	-2.50405	0.0122779	0.029549347	down-regulated	protein_coding
FKBP11	ENSMMUG00000006971	36.36042437	-1.981806847	0.791532	-2.50376	0.0122881	0.029563667	down-regulated	protein_coding
PUS7L	ENSMMUG00000000203	7.930086854	-3.356339031	1.34092	-2.50301	0.0123142	0.029621389	down-regulated	protein_coding
STC1	ENSMMUG00000008858	25.51269099	-2.422486181	0.967885	-2.50287	0.0123192	0.029623422	down-regulated	protein_coding
CLK3	ENSMMUG00000011069	206.9511636	1.25206599	0.500242	2.502921	0.0123173	0.029623422	up-regulated	protein_coding
SMPDL3A	ENSMMUG00000021905	113.7034564	-1.608376344	0.642748	-2.50234	0.0123374	0.029657215	down-regulated	protein_coding
SLC29A1	ENSMMUG00000022685	32.5284305	-2.09099066	0.835603	-2.50237	0.0123364	0.029657215	down-regulated	protein_coding

CDK9	ENSMMUG00000013195	89.46378703	-1.255429614	0.501756	-2.50207	0.0123468	0.029674787	down-regulated	protein_coding
GFM1	ENSMMUG00000004961	79.45887781	-1.34594569	0.537961	-2.50194	0.0123515	0.029680827	down-regulated	protein_coding
ILF3	ENSMMUG00000014673	323.1810971	-1.372206903	0.5487	-2.50083	0.0123901	0.029768623	down-regulated	protein_coding
TRAPPC4	ENSMMUG00000016946	175.2201355	1.31962691	0.52773	2.500574	0.0123992	0.029785474	up-regulated	protein_coding
PSD3	ENSMMUG00000016385	45.69662737	1.623453068	0.649489	2.499585	0.0124339	0.029863662	up-regulated	protein_coding
PIK3R3	ENSMMUG00000015080	13.09174841	2.413888214	0.965771	2.499443	0.0124389	0.029870625	up-regulated	protein_coding
NDUFB2	ENSMMUG00000015284	263.5161105	1.062097332	0.424984	2.499149	0.0124492	0.029888235	up-regulated	protein_coding
PGBD2	ENSMMUG00000023296	8.623849179	-3.477466238	1.39148	-2.49911	0.0124504	0.029888235	down-regulated	protein_coding
UPF3A	ENSMMUG00000007449	27.68994076	-2.162960016	0.865542	-2.49897	0.0124557	0.029895676	down-regulated	protein_coding
PPP2R3A	ENSMMUG00000000932	77.97758945	1.60578785	0.642616	2.498828	0.0124605	0.029902156	up-regulated	protein_coding
ZNF773	ENSMMUG00000010429	4.084420916	3.56555437	1.427127	2.498414	0.012475	0.029932057	up-regulated	protein_coding
RIOK3	ENSMMUG00000021478	51.43629589	-2.115289406	0.846898	-2.49769	0.0125005	0.029987981	down-regulated	protein_coding
NKAP	ENSMMUG00000012382	33.16336553	-1.819572712	0.728813	-2.49663	0.0125381	0.030073236	down-regulated	protein_coding
TRERF1	ENSMMUG00000005575	33.91804626	2.202272236	0.883029	2.493997	0.0126313	0.030286552	up-regulated	protein_coding
TMED1	ENSMMUG00000012041	52.83113075	-1.39424489	0.559128	-2.4936	0.0126453	0.030314935	down-regulated	protein_coding
KIF4B	ENSMMUG00000011463	2.754045185	3.9422615	1.581676	2.492458	0.0126862	0.030407627	up-regulated	protein_coding
DACT2	ENSMMUG00000004230	9.656539857	3.619332422	1.452648	2.491541	0.012719	0.030481321	up-regulated	protein_coding
EFR3B	ENSMMUG00000016461	4.009254722	3.299138832	1.32429	2.491251	0.0127294	0.030501018	up-regulated	protein_coding
TICAM1	ENSMMUG00000014163	185.9918029	1.189455145	0.477595	2.49051	0.012756	0.030549147	up-regulated	protein_coding
RBSN	ENSMMUG00000005158	41.5561966	1.685336355	0.676967	2.48954	0.0127908	0.030625996	up-regulated	protein_coding
ELMO3	ENSMMUG00000020259	180.5375366	-1.376460323	0.552907	-2.4895	0.0127924	0.030625996	down-regulated	protein_coding
DHX30	ENSMMUG00000003820	132.4235766	-1.153001277	0.463196	-2.48923	0.012802	0.030643811	down-regulated	protein_coding
CCT4	ENSMMUG00000013973	148.4319117	-1.498511641	0.602213	-2.48834	0.0128341	0.030710131	down-regulated	protein_coding
FAM195B	ENSMMUG00000000258	45.92737778	-2.148133503	0.86332	-2.48823	0.0128382	0.030714956	down-regulated	protein_coding
WAS	ENSMMUG00000019767	9.652875539	4.553587684	1.830187	2.488045	0.0128447	0.030725333	up-regulated	protein_coding
TMEM82	ENSMMUG00000016284	148.6972143	-1.155972994	0.464693	-2.48761	0.0128606	0.030758107	down-regulated	protein_coding
ZNF639	ENSMMUG00000023078	8.441827841	-3.445249672	1.385416	-2.4868	0.0128898	0.03082277	down-regulated	protein_coding
MTMR3	ENSMMUG00000022236	106.8160816	1.262277777	0.507935	2.485118	0.0129509	0.030958235	up-regulated	protein_coding
EXOSC7	ENSMMUG00000019130	78.41441813	1.287145687	0.51803	2.484695	0.0129662	0.030989749	up-regulated	protein_coding
YIPF6	ENSMMUG00000016986	25.01395527	-3.126718527	1.258431	-2.48462	0.0129691	0.030991425	down-regulated	protein_coding
RIPK2	ENSMMUG00000020725	28.00823534	-2.2231763	0.895363	-2.48299	0.0130285	0.031112233	down-regulated	protein_coding
CLN5	ENSMMUG00000021457	83.20324321	1.464997672	0.589999	2.483049	0.0130263	0.031112233	up-regulated	protein_coding
CTSK	ENSMMUG00000000537	9.403390509	-3.602538997	1.451575	-2.48181	0.0130715	0.031207929	down-regulated	protein_coding
AP5Z1	ENSMMUG00000011228	120.9306227	-1.677763625	0.676034	-2.48178	0.013073	0.031207929	down-regulated	protein_coding
DIS3	ENSMMUG00000000787	52.80387276	-1.39482426	0.56213	-2.48132	0.0130897	0.031242716	down-regulated	protein_coding
PSRC1	ENSMMUG00000015380	10.51672447	-3.760867425	1.515841	-2.48104	0.0130998	0.031261478	down-regulated	protein_coding
APOPT1	ENSMMUG00000017006	27.27298985	-2.137332786	0.861509	-2.48092	0.0131045	0.031267337	down-regulated	protein_coding
Y_RNA	ENSMMUG00000028053	14.41813574	-3.162412973	1.275284	-2.47977	0.0131466	0.031357311	down-regulated	misc_RNA
SPC25	ENSMMUG00000013827	12.74706461	-4.039179642	1.629256	-2.47916	0.0131694	0.031395647	down-regulated	protein_coding
C14H11ORF52	ENSMMUG00000030912	15.15903683	-3.237505124	1.305885	-2.47917	0.013169	0.031395647	down-regulated	protein_coding
UBXN2B	ENSMMUG00000001100	8.049085299	-3.379990944	1.36346	-2.47898	0.0131758	0.031404071	down-regulated	protein_coding
BSN	ENSMMUG00000014537	8.011968953	3.554811068	1.434039	2.478881	0.0131795	0.031404071	up-regulated	protein_coding
ZBTB33	ENSMMUG00000011076	35.13649824	-2.131167204	0.85984	-2.47856	0.0131913	0.031426718	down-regulated	protein_coding
NTPCR	ENSMMUG00000012614	31.8948672	-1.682703856	0.67933	-2.47701	0.013249	0.031553563	down-regulated	protein_coding
UFL1	ENSMMUG00000012616	66.74536463	-1.478914761	0.597206	-2.47639	0.0132719	0.03160282	down-regulated	protein_coding
RPS6KB1	ENSMMUG00000019305	52.5866595	1.152738176	0.465541	2.476124	0.0132817	0.031620949	up-regulated	protein_coding
SMAD6	ENSMMUG00000019018	25.97803972	1.565259556	0.632355	2.475285	0.013313	0.03168471	up-regulated	protein_coding
FAM118B	ENSMMUG00000007935	68.94145831	-1.079850048	0.43627	-2.47519	0.0133166	0.031688021	down-regulated	protein_coding
FAM45A	ENSMMUG00000008289	29.01195151	-1.903578699	0.769097	-2.47508	0.0133205	0.031691243	down-regulated	protein_coding
FAM168B	ENSMMUG00000011318	48.89771107	-1.318235895	0.532614	-2.47503	0.0133225	0.031691243	down-regulated	protein_coding
SLC2A10	ENSMMUG00000016927	10.5241817	1.866028643	0.754093	2.474534	0.013341	0.031730021	up-regulated	protein_coding
SLC4A7	ENSMMUG00000019327	87.03334334	-1.912350309	0.773267	-2.47308	0.0133954	0.031854054	down-regulated	protein_coding
C11ORF68	ENSMMUG00000008654	47.39835311	-1.563365137	0.632178	-2.47298	0.0133991	0.031857538	down-regulated	protein_coding
C1R	ENSMMUG00000006470	5.715083797	3.373947063	1.365476	2.470894	0.0134776	0.032038699	up-regulated	protein_coding
LCOR	ENSMMUG00000010425	53.94559934	1.910554524	0.773459	2.470143	0.0135059	0.03209521	up-regulated	protein_coding
SRSF3	ENSMMUG00000014306	244.8739766	-1.256702723	0.508922	-2.46934	0.0135361	0.032161552	down-regulated	protein_coding
PACSIN3	ENSMMUG00000006359	64.96594561	-1.640286407	0.664444	-2.46866	0.013562	0.032217768	down-regulated	protein_coding
HEATR3	ENSMMUG00000020065	32.50175115	-1.776801136	0.71979	-2.4685	0.0135681	0.032226794	down-regulated	protein_coding
ITGAD	ENSMMUG00000014349	5.582472501	6.099601143	2.471232	2.468243	0.0135778	0.032233661	up-regulated	protein_coding
HS3ST3B1	ENSMMUG00000016015	4.723347991	5.25973794	2.130916	2.468299	0.0135757	0.032233661	up-regulated	protein_coding
DUS2L	ENSMMUG00000021841	22.12796593	-2.924593378	1.184843	-2.46834	0.0135742	0.032233661	down-regulated	protein_coding
FOXP1	ENSMMUG00000002889	67.2671324	1.184661212	0.480092	2.46757	0.0136034	0.032279455	up-regulated	protein_coding
RGS17	ENSMMUG00000019078	6.472673015	2.537964675	1.028488	2.467666	0.0135997	0.032279455	up-regulated	protein_coding
E2F6	ENSMMUG00000023225	14.66215706	-3.195999217	1.295332	-2.46732	0.0136128	0.032295101	down-regulated	protein_coding
COXIII	ENSMMUG00000028682	2423.927159	5.139891764	2.083471	2.466985	0.0136256	0.032319973	up-regulated	protein_coding
FBXO34	ENSMMUG00000023258	52.86847654	-1.614614373	0.654616	-2.46651	0.0136438	0.032357827	down-regulated	protein_coding
RAB3GAP1	ENSMMUG00000014630	65.65580553	-1.398688771	0.567114	-2.46633	0.0136507	0.032368633	down-regulated	protein_coding
NUDT3	ENSMMUG00000019532	41.32942593	-1.582637266	0.642099	-2.46479	0.0137095	0.032495422	down-regulated	protein_coding
PLA2G12B	ENSMMUG00000020405	45.24209522	-1.650226693	0.669532	-2.46475	0.0137111	0.032495422	down-regulated	protein_coding
ZNF740	ENSMMUG00000005246	7.546523352	-3.285475178	1.333826	-2.4632	0.0137705	0.032630818	down-regulated	protein_coding
FN3KRP	ENSMMUG00000014660	57.63283302	-1.323909838	0.537594	-2.46266	0.0137912	0.032674323	down-regulated	protein_coding
mml-mir-590	ENSMMUG00000027207	3.709691044	3.756580147	1.525533	2.46247	0.0137984	0.03268596	up-regulated	miRNA
CCDC146	ENSMMUG00000006696	8.13471858	3.608343471	1.465397	2.462365	0.0138024	0.032690038	up-regulated	protein_coding
CCDC64	ENSMMUG00000011209	31.03721774	-2.149114548	0.873154	-2.46132	0.0138425	0.032774041	down-regulated	protein_coding
EREG	ENSMMUG00000002313	13.58693782	-4.134452474	1.680216	-2.46067	0.0138679	0.032828581	down-regulated	protein_coding
SHKBP1	ENSMMUG00000020797	84.62921603	-1.620710417	0.658831	-2.45998	0.0138945	0.032886171	down-regulated	protein_coding
TAF12	ENSMMUG00000005748	15.1337787	-3.242565117	1.318355	-2.45955	0.013911	0.032914081	down-regulated	protein_coding
OGFOD3	ENSMMUG00000019220	120.0455119	1.551983564	0.630993	2.45959	0.0139096	0.032914081	up-regulated	protein_coding
DGKG	ENSMMUG00000021972	4.916658534	3.861345016	1.570278	2.45902	0.0139317	0.03295761	up-regulated	protein_coding
GRAMD1C	ENSMMUG00000001943	23.99989229	-2.32453574	0.945624	-2.4582	0.0139634	0.03302707	down-regulated	protein_coding
THAP7	ENSMMUG00000020483	36.13317367	-2.266396551	0.922321	-2.45728	0.0139995	0.033106981	down-regulated	protein_coding
DHX33	ENSMMUG00000006594	22.68358076	1.2891514	0.5247	2.456932	0.0140129	0.033133122	up-regulated	protein_coding
UHRF1BP1L	ENSMMUG00000023601	99.19923647	-1.588434376	0.646554	-2.45677	0.0140192	0.033142549	down-regulated	protein_coding
GRIN1	ENSMMUG00000001661	30.89115879	2.144401817	0.873083	2.456126	0.0140444	0.03319089	up-regulated	protein_coding
ANKRD43	ENSMMUG00000010310	86.29040428	-1.160622385	0.472714	-2.45523	0.0140793	0.033262355	down-regulated	protein_coding
CRIP1	ENSMMUG00000007158	189.980684	-1.102290398	0.449224	-2.45376	0.014137	0.033387486	down-regulated	protein_coding
RABL3	ENSMMUG00000001594	28.7624875	-1.857864881	0.757324	-2.4532	0.0141593	0.033434553	down-regulated	protein_coding
OGFOD2	ENSMMUG00000015926	52.3727215	-1.712727836	0.698229	-2.45296	0.0141687	0.033451087	down-regulated	protein_coding
SLC7A5	ENSMMUG00000001595	16.2307028	1.522708899	0.62083	2.452699	0.0141789	0.033469653	up-regulated	protein_coding
OPLAH	ENSMMUG00000004009	47.14226209	-1.672853079	0.682082	-2.45257	0.014184	0.033476218	down-regulated	protein_coding
HPS1	ENSMMUG00000020604	62.25193448	-1.343703481	0.548031	-2.45188	0.0142114	0.033535133	down-regulated	protein_coding
ABHD11	ENSMMUG00000014557	265.0864364	1.343088697	0.547895	2.45136	0.0142318	0.033572073	up-regulated	protein_coding
DTX4	ENSMMUG00000010277	115.940175	1.184013346	0.48306	2.451067	0.0142433	0.033587452	up-regulated	protein_coding
THOC3	ENSMMUG00000018737	35.74112423	-2.247663975	0.917032	-2.45102	0.0142451	0.033587452	down-regulated	protein_coding
ABHD3	ENSMMUG00000004382	52.60817931	-1.731490239	0.706685	-2.45016	0.0142793	0.033661791	down-regulated	protein_coding
FLT3LG	ENSMMUG00000006825	8.611347641	3.198706882	1.306601	2.448112	0.0143607	0.033842319	up-regulated	protein_coding
BLOC1S3	ENSMMUG00000022934	7.682646922	-3.312620202	1.353372	-2.44768	0.014378	0.033877438	down-regulated	protein_coding
TSNAXIP1	ENSMMUG00000007803	3.048796884	3.327157819	1.359582	2.447191	0.0143974	0.03390637	up-regulated	protein_coding
CHIA	ENSMMUG00000008857	13.75322319	8.94108795	3.653539	2.44724	0.0143955	0.03390637	up-regulated	protein_coding
GLE1	ENSMMUG00000005989	69.55882471	-1.230426099	0.502809	-2.44711	0.0144009	0.033908785	down-regulated	protein_coding

VARS	ENSMMUG00000006828	141.9601747	-1.4479957	0.59181	-2.44672	0.0144161	0.033938967	down-regulated	protein_coding
TAF13	ENSMMUG00000001010	7.684171047	-3.309831759	1.352814	-2.44663	0.01442	0.033939746	down-regulated	protein_coding
LGR4	ENSMMUG00000023491	296.9033725	-1.280836293	0.523518	-2.4466	0.0144212	0.033939746	down-regulated	protein_coding
EFCAB7	ENSMMUG00000020048	22.95998073	2.719521469	1.111747	2.44617	0.0144383	0.033974324	up-regulated	protein_coding
MYNN	ENSMMUG00000021875	43.05038591	1.100481723	0.449985	2.445595	0.0144613	0.03401152	up-regulated	protein_coding
TMEM60	ENSMMUG00000009007	22.34288788	1.574050287	0.643736	2.445179	0.014478	0.034045114	up-regulated	protein_coding
CCDC12	ENSMMUG00000019810	45.62102031	-2.176009288	0.89005	-2.44482	0.0144926	0.034073656	down-regulated	protein_coding
CPB1	ENSMMUG00000005518	8.376317377	4.475842453	1.83081	2.444734	0.0144959	0.034075851	up-regulated	protein_coding
SLC44A3	ENSMMUG00000001686	73.88619968	-1.11121852	0.454584	-2.44447	0.0145064	0.034094822	down-regulated	protein_coding
PSKH1	ENSMMUG00000011895	74.66881724	-1.153557813	0.472052	-2.44371	0.014537	0.034155529	down-regulated	protein_coding
IFI35	ENSMMUG00000015999	43.95619358	-1.850854072	0.757545	-2.44323	0.0145565	0.034189995	down-regulated	protein_coding
THG1L	ENSMMUG00000001785	8.761740334	-3.498728715	1.432143	-2.443	0.0145657	0.03420575	down-regulated	protein_coding
ANKLE2	ENSMMUG00000015467	112.2277409	-1.024405713	0.419367	-2.44274	0.014576	0.034224432	down-regulated	protein_coding
TMEM5	ENSMMUG00000011561	7.868761055	-3.344460524	1.369263	-2.44253	0.0145849	0.03423956	down-regulated	protein_coding
UBFD1	ENSMMUG00000020523	28.27631541	-1.855645348	0.759801	-2.44228	0.0145949	0.034251728	down-regulated	protein_coding
LONRF2	ENSMMUG00000017455	4.75983996	3.284994757	1.345095	2.442202	0.014598	0.034253181	up-regulated	protein_coding
PIAS1	ENSMMUG00000011699	102.5239208	1.086528539	0.444959	2.441863	0.0146117	0.034279715	up-regulated	protein_coding
NPDC1	ENSMMUG00000017860	343.0555052	1.182464205	0.484269	2.441751	0.0146162	0.034284639	up-regulated	protein_coding
ARL8A	ENSMMUG00000031357	21.95722423	-2.73218861	1.119161	-2.44128	0.0146352	0.034323495	down-regulated	protein_coding
ZNF675	ENSMMUG00000003689	7.921849599	-3.354753961	1.374238	-2.44117	0.0146396	0.034328039	down-regulated	protein_coding
COL4A3BP	ENSMMUG00000006141	24.26066046	-3.059545696	1.253371	-2.44105	0.0146445	0.034333815	down-regulated	protein_coding
NGLY1	ENSMMUG00000014790	51.31429375	-1.314798804	0.538912	-2.43973	0.0146983	0.034454373	down-regulated	protein_coding
TP53INP1	ENSMMUG00000005360	8.786324209	-3.506718224	1.437716	-2.43909	0.0147243	0.034503934	down-regulated	protein_coding
EFCAB9	ENSMMUG00000006817	5.586769485	6.053674842	2.482786	2.438259	0.0147582	0.034572499	up-regulated	protein_coding
HIST1H4K	ENSMMUG00000032119	5.196479839	3.357704804	1.377095	2.438252	0.0147585	0.034572499	up-regulated	protein_coding
ATP6V1C1	ENSMMUG00000001078	37.72308023	-1.769375624	0.725738	-2.43804	0.0147673	0.034587375	down-regulated	protein_coding
TAF7	ENSMMUG00000021944	76.12238874	-1.339228636	0.549351	-2.43784	0.0147754	0.03460072	down-regulated	protein_coding
PCYOX1	ENSMMUG00000007959	81.71055408	-1.861606992	0.763846	-2.43715	0.0148036	0.034655178	down-regulated	protein_coding
MANSC1	ENSMMUG00000018577	34.32914351	-1.768560151	0.725821	-2.43663	0.0148246	0.034698737	down-regulated	protein_coding
TBC1D10C	ENSMMUG00000017280	8.559921211	3.604262766	1.47931	2.436448	0.0148323	0.034710897	up-regulated	protein_coding
HMGN3	ENSMMUG00000004351	29.59763772	-3.11818483	1.280212	-2.43568	0.0148639	0.034778474	down-regulated	protein_coding
FOXC1	ENSMMUG00000005778	49.444906	1.870109674	0.767815	2.435625	0.0148661	0.034778474	up-regulated	protein_coding
MTA2	ENSMMUG00000018110	152.4418948	-1.007745676	0.413927	-2.4346	0.0149084	0.034871671	down-regulated	protein_coding
ATP8A1	ENSMMUG00000017733	20.40815369	2.348453436	0.964643	2.434532	0.0149111	0.034872169	up-regulated	protein_coding
NOSTRIN	ENSMMUG00000019968	58.42136217	1.200277063	0.493192	2.433691	0.0149457	0.034947491	up-regulated	protein_coding
CKLF	ENSMMUG00000000030	11.19926946	-3.852044325	1.582854	-2.43361	0.0149492	0.034949797	down-regulated	protein_coding
ETV6	ENSMMUG00000014370	61.93218384	1.436461404	0.59034	2.433276	0.0149629	0.034975997	up-regulated	protein_coding
HIST3H2BB	ENSMMUG00000031510	3.972237968	3.203055696	1.316637	2.432755	0.0149844	0.035020639	up-regulated	protein_coding
FUK	ENSMMUG00000003174	92.35107499	-1.294770819	0.532515	-2.43143	0.0150395	0.035131824	down-regulated	protein_coding
CARKD	ENSMMUG00000012663	42.18109305	-1.545824784	0.635764	-2.43144	0.0150387	0.035131824	down-regulated	protein_coding
C13H2ORF49	ENSMMUG00000008436	7.554867127	-3.286226467	1.351837	-2.43093	0.01506	0.035173881	down-regulated	protein_coding
ZW10	ENSMMUG00000023772	27.2381435	-2.187348099	0.900057	-2.43023	0.0150891	0.035236135	down-regulated	protein_coding
SLC9A6	ENSMMUG00000011271	39.62838083	-1.883004401	0.774937	-2.42988	0.0151038	0.035258759	down-regulated	protein_coding
TMEM199	ENSMMUG00000021351	33.17285245	-1.796345828	0.73926	-2.42992	0.0151019	0.035258759	down-regulated	protein_coding
CCM2	ENSMMUG00000001799	55.92039773	-1.615778895	0.665217	-2.42895	0.0151426	0.035331862	down-regulated	protein_coding
COG6	ENSMMUG00000004486	52.02762368	-1.528438369	0.629312	-2.42875	0.0151511	0.03534012	down-regulated	protein_coding
ARRDC3	ENSMMUG00000003569	62.86063893	-1.606176317	0.661727	-2.42725	0.0152138	0.035468888	down-regulated	protein_coding
ABHD5	ENSMMUG00000001031	16.58408491	-3.378379124	1.391946	-2.42709	0.0152205	0.035478525	down-regulated	protein_coding
TMEM55B	ENSMMUG00000019124	81.16767751	-1.29620466	0.534201	-2.42643	0.015248	0.035536863	down-regulated	protein_coding
RBMS1	ENSMMUG00000016825	9.629338757	-3.638369982	1.499603	-2.42622	0.0152569	0.035551698	down-regulated	protein_coding
COL18A1	ENSMMUG00000030502	11.15900264	-3.84716781	1.585899	-2.42586	0.0152722	0.035581497	down-regulated	protein_coding
PSMB5	ENSMMUG00000006007	60.67410949	-1.702353315	0.701892	-2.42538	0.0152924	0.035619416	down-regulated	protein_coding
SUGT1	ENSMMUG00000016401	41.48918798	-1.455779784	0.600234	-2.42535	0.0152935	0.035619416	down-regulated	protein_coding
EDRF1	ENSMMUG00000008535	41.47015406	1.063562335	0.438612	2.424839	0.0153152	0.035658247	up-regulated	protein_coding
TMCO6	ENSMMUG00000018246	50.51994037	1.329553485	0.548306	2.424838	0.0153152	0.035658247	up-regulated	protein_coding
EDN1	ENSMMUG00000007263	73.17996262	-1.412781919	0.582721	-2.42446	0.0153313	0.035683901	down-regulated	protein_coding
C18orf21	ENSMMUG00000018703	14.35996404	-3.154484821	1.301101	-2.42447	0.0153306	0.035683901	down-regulated	protein_coding
HES2	ENSMMUG00000022882	35.15903252	-2.207395895	0.91087	-2.42339	0.0153763	0.035770959	down-regulated	protein_coding
FIBCD1	ENSMMUG00000012713	6.4620771	4.354494094	1.797091	2.423079	0.0153896	0.035784229	up-regulated	protein_coding
UGT2B15	ENSMMUG00000023715	467.800187	-2.071525909	0.85495	-2.42298	0.0153938	0.035788085	down-regulated	protein_coding
CDC40	ENSMMUG00000008182	20.62286072	-2.731397057	1.127323	-2.42291	0.0153969	0.035789573	down-regulated	protein_coding
C15H9ORF85	ENSMMUG00000018881	7.684332433	-3.31279716	1.367323	-2.42284	0.0153999	0.035790629	down-regulated	protein_coding
WBP4	ENSMMUG00000007881	31.99958246	-1.63126617	0.673518	-2.42201	0.0154349	0.035866136	down-regulated	protein_coding
MAD2L1BP	ENSMMUG00000006363	38.42115775	-2.059310575	0.850299	-2.42187	0.015441	0.035874404	down-regulated	protein_coding
THADA	ENSMMUG00000000642	48.52472418	1.287723802	0.531776	2.421553	0.0154544	0.035899495	up-regulated	protein_coding
PTGS2	ENSMMUG00000016857	9.994032545	3.202295427	1.322539	2.421323	0.0154641	0.03591627	up-regulated	protein_coding
NR2F2	ENSMMUG00000005430	32.79037434	-1.785376079	0.737384	-2.42123	0.0154681	0.035919671	down-regulated	protein_coding
EIF2D	ENSMMUG00000002528	93.61238656	-1.042753165	0.430721	-2.42095	0.01548	0.03592958	down-regulated	protein_coding
ZNF320	ENSMMUG00000012085	8.427931705	-3.446579696	1.423614	-2.42101	0.0154776	0.03592958	down-regulated	protein_coding
MBNL2	ENSMMUG00000022744	62.08486543	-1.639829674	0.677328	-2.42103	0.0154768	0.03592958	down-regulated	protein_coding
ELOVL5	ENSMMUG00000006422	32.68046619	1.421688151	0.587438	2.420151	0.0155141	0.036001709	up-regulated	protein_coding
PAWR	ENSMMUG00000015179	75.57849408	1.107553465	0.457648	2.420101	0.0155162	0.036001709	up-regulated	protein_coding
C4orf33	ENSMMUG00000002148	26.99465493	-2.345442862	0.969268	-2.41981	0.0155286	0.036015519	down-regulated	protein_coding
BCL2L13	ENSMMUG00000021476	51.769476	1.399629871	0.578411	2.419783	0.0155298	0.036015519	up-regulated	protein_coding
IDUA	ENSMMUG00000032299	80.40256201	-1.751657449	0.724122	-2.41901	0.0155629	0.036080564	down-regulated	protein_coding
SLC1A5	ENSMMUG00000022163	127.409134	-1.140526879	0.471658	-2.41812	0.0156009	0.036162632	down-regulated	protein_coding
TUBE1	ENSMMUG00000012581	14.07661434	-3.123927632	1.292068	-2.41777	0.0156158	0.036191389	down-regulated	protein_coding
SHTN1	ENSMMUG00000007578	94.85927386	-1.660778602	0.687058	-2.41723	0.015639	0.036228833	down-regulated	protein_coding
RNLS	ENSMMUG00000014341	24.83461977	1.879727838	0.777614	2.417303	0.015636	0.036228833	up-regulated	protein_coding
LHPP	ENSMMUG00000015215	35.71045165	-1.691302964	0.69969	-2.41722	0.0156397	0.036228833	down-regulated	protein_coding
ANAPC10	ENSMMUG00000004432	7.623689122	-3.297563498	1.364313	-2.41701	0.0156484	0.036243145	down-regulated	protein_coding
RRM2	ENSMMUG00000002057	79.52558575	-2.400119602	0.993146	-2.41668	0.0156626	0.036263518	down-regulated	protein_coding
ZNF426	ENSMMUG00000005694	7.691722786	-3.31433383	1.371454	-2.41666	0.0156638	0.036263518	down-regulated	protein_coding
SLC41A3	ENSMMUG00000012847	55.14516	-1.272669011	0.526629	-2.41663	0.0156649	0.036263518	down-regulated	protein_coding
FBL	ENSMMUG00000022725	109.3694068	-2.00425678	0.829426	-2.41644	0.0156732	0.036276798	down-regulated	protein_coding
DEF8	ENSMMUG00000000391	77.66115896	-1.204483205	0.498589	-2.41578	0.0157014	0.036324276	down-regulated	protein_coding
CDS1	ENSMMUG00000021162	55.52871136	-2.087647785	0.864154	-2.41583	0.0156995	0.036324276	down-regulated	protein_coding
RHPN2	ENSMMUG00000028798	66.94998001	-1.527230332	0.632182	-2.41581	0.0157003	0.036324276	down-regulated	protein_coding
C5ORF36	ENSMMUG00000002507	5.083869235	3.312867341	1.371849	2.414892	0.0157399	0.036401379	up-regulated	protein_coding
C15ORF57	ENSMMUG00000001482	24.31104387	-2.421787487	1.003025	-2.41448	0.0157576	0.036436284	down-regulated	protein_coding
CTU1	ENSMMUG00000018162	8.758981298	-3.497269344	1.448763	-2.41397	0.0157798	0.03648182	down-regulated	protein_coding
SNIP1	ENSMMUG00000008421	19.59510504	-2.651539205	1.099073	-2.41252	0.0158425	0.036615531	down-regulated	protein_coding
LTB	ENSMMUG00000008849	32.19239296	-2.9120333	1.207082	-2.41246	0.0158454	0.036615531	down-regulated	protein_coding
RASA2	ENSMMUG00000009418	14.68645728	-3.193097565	1.32358	-2.41247	0.0158448	0.036615531	down-regulated	protein_coding
ANKRD10	ENSMMUG00000018929	100.092607	-1.682211924	0.697447	-2.41196	0.0158672	0.036659767	down-regulated	protein_coding
PPT1	ENSMMUG00000009189	26.03209439	-2.295825412	0.951922	-2.41178	0.0158749	0.036671731	down-regulated	protein_coding
SHB	ENSMMUG00000008670	107.4874675	-1.11906988	0.464029	-2.41164	0.0158811	0.036679962	down-regulated	protein_coding
SRFBP1	ENSMMUG00000021009	7.26261143	-3.228931537	1.339073	-2.41132	0.015895	0.036706065	down-regulated	protein_coding
FNDC5	ENSMMUG00000000310	5.919274554	3.956952707	1.641073	2.411198	0.0159002	0.036712119	up-regulated	protein_coding
C7ORF59	ENSMMUG00000018401	73.35094768	-1.838892574	0.762698	-2.41104	0.0159072	0.036722399	down-regulated	protein_coding

XIAP	ENSMMUG00000032353	9.565486413	-3.629017857	1.505294	-2.41084	0.015916	0.036736597	down-regulated	protein_coding
CTSL	ENSMMUG00000017609	137.1093601	-1.226298218	0.508766	-2.41034	0.0159377	0.036780805	down-regulated	protein_coding
ZNF787	ENSMMUG00000022338	126.8481488	-1.356169219	0.562905	-2.40923	0.0159862	0.036886573	down-regulated	protein_coding
ACAD10	ENSMMUG00000015754	42.16755213	-1.532076547	0.636022	-2.40884	0.0160032	0.036919886	down-regulated	protein_coding
PPP1R15B	ENSMMUG00000016561	265.9311161	1.028864433	0.427152	2.408659	0.0160112	0.036932344	up-regulated	protein_coding
NDRG3	ENSMMUG00000008201	14.40095066	-3.162302449	1.313088	-2.40833	0.0160256	0.036959563	down-regulated	protein_coding
SOCS5	ENSMMUG00000005384	27.99360288	-2.201877282	0.91442	-2.40795	0.0160424	0.036992013	down-regulated	protein_coding
NFXL1	ENSMMUG00000021780	37.80217224	1.736509912	0.721174	2.407891	0.0160449	0.036992013	up-regulated	protein_coding
PLA2R1	ENSMMUG00000020722	65.960229	-1.699831383	0.705989	-2.40773	0.016052	0.037002158	down-regulated	protein_coding
TMEM41B	ENSMMUG00000005639	55.40648267	-1.70217996	0.707056	-2.40742	0.0160658	0.037021918	down-regulated	protein_coding
BRCC3	ENSMMUG00000010253	14.9126727	-3.152184621	1.309354	-2.40743	0.016065	0.037021918	down-regulated	protein_coding
SLCO4A1	ENSMMUG00000016848	91.57795527	-1.49725638	0.621995	-2.40718	0.016076	0.037039562	down-regulated	protein_coding
RAP2C	ENSMMUG00000016588	51.53842931	-1.381567734	0.573973	-2.40703	0.016083	0.037049646	down-regulated	protein_coding
APMAP	ENSMMUG00000005755	199.5031759	-1.115420073	0.463522	-2.4064	0.0161106	0.03710102	down-regulated	protein_coding
ACER3	ENSMMUG00000018168	92.02060565	-1.376684883	0.572092	-2.40641	0.0161104	0.03710102	down-regulated	protein_coding
PIK3R1	ENSMMUG00000011532	81.64000627	1.119546732	0.46528	2.406181	0.0161203	0.037117303	up-regulated	protein_coding
SLC27A1	ENSMMUG00000000194	87.57266891	1.284763309	0.534048	2.405709	0.0161411	0.03715553	up-regulated	protein_coding
KCNIP2	ENSMMUG00000015776	6.474791524	2.842535767	1.181591	2.405686	0.0161421	0.03715553	up-regulated	protein_coding
C9ORF140	ENSMMUG00000007112	34.10243747	1.835591089	0.763311	2.404774	0.0161825	0.037242279	up-regulated	protein_coding
RBM8A	ENSMMUG00000016051	143.4660237	1.29709545	0.539448	2.404485	0.0161953	0.037265755	up-regulated	protein_coding
HPDL	ENSMMUG00000020078	13.99348393	-4.173825017	1.736066	-2.40419	0.0162085	0.037290163	down-regulated	protein_coding
C20H16ORF72	ENSMMUG00000001944	110.0635024	-1.05984711	0.4409	-2.40382	0.0162246	0.03732099	down-regulated	protein_coding
ZBTB4	ENSMMUG00000023616	54.97887053	-2.079798636	0.865412	-2.40325	0.0162503	0.037374008	down-regulated	protein_coding
RNF122	ENSMMUG00000029010	7.239898648	-3.224495014	1.342239	-2.40233	0.0162912	0.037455917	down-regulated	protein_coding
KCNJ2	ENSMMUG00000014051	45.17692153	2.466511064	1.026938	2.401811	0.0163141	0.037502646	up-regulated	protein_coding
TOPBP1	ENSMMUG00000000059	93.53076091	-1.174924742	0.489199	-2.40173	0.0163177	0.037504658	down-regulated	protein_coding
OSGEP	ENSMMUG00000006848	22.87747485	-2.885107888	1.201329	-2.4016	0.0163237	0.037512504	down-regulated	protein_coding
ZNF616	ENSMMUG00000001768	28.27606131	1.426212714	0.593977	2.401126	0.0163447	0.03754894	up-regulated	protein_coding
CAMK2D	ENSMMUG00000008587	154.2465612	-1.525898018	0.635494	-2.40112	0.0163449	0.03754894	down-regulated	protein_coding
SLC15A1	ENSMMUG00000001402	177.6760279	-1.8761955	0.781564	-2.40057	0.0163697	0.037599821	down-regulated	protein_coding
TTC32	ENSMMUG00000001570	8.235672958	-3.409414895	1.420294	-2.4005	0.0163727	0.037600707	down-regulated	protein_coding
ZNF644	ENSMMUG00000007635	43.47308067	-1.410723098	0.587716	-2.40035	0.0163796	0.03761027	down-regulated	protein_coding
FRG1	ENSMMUG00000001824	23.68348266	-2.405932767	1.002574	-2.39975	0.0164061	0.037658883	down-regulated	protein_coding
FMR1	ENSMMUG00000015814	14.30288054	-3.160205656	1.316978	-2.39959	0.0164135	0.037669788	down-regulated	protein_coding
SACS	ENSMMUG00000001292	10.5877906	3.118384669	1.300186	2.398414	0.0164663	0.037772564	up-regulated	protein_coding
EFR3A	ENSMMUG00000012559	156.7345167	-1.227904657	0.511959	-2.39844	0.0164649	0.037772564	down-regulated	protein_coding
ZDHHC16	ENSMMUG00000020029	80.06860515	1.386578242	0.578118	2.398433	0.0164654	0.037772564	up-regulated	protein_coding
PLXNB1	ENSMMUG00000003074	358.6223753	-1.232365453	0.51389	-2.39811	0.0164798	0.03779745	down-regulated	protein_coding
PRKAG1	ENSMMUG00000015713	76.86327184	-1.2080557	0.504342	-2.39531	0.0166062	0.038075088	down-regulated	protein_coding
IFT122	ENSMMUG00000012727	50.17689387	1.205575557	0.503414	2.394798	0.0166295	0.038122376	up-regulated	protein_coding
ANO8	ENSMMUG00000016232	46.49636962	-1.972353409	0.823714	-2.39446	0.0166447	0.038144767	down-regulated	protein_coding
RASSF2	ENSMMUG00000020004	7.959112106	-3.362196109	1.404266	-2.39427	0.0166534	0.038150359	down-regulated	protein_coding
USP28	ENSMMUG00000023773	23.59676869	-2.374322661	0.991772	-2.39402	0.0166649	0.038166237	down-regulated	protein_coding
CEP128	ENSMMUG00000019710	13.3011786	1.842223889	0.769669	2.393527	0.0166873	0.038211369	up-regulated	protein_coding
LCORL	ENSMMUG00000018073	8.650614338	-3.484065601	1.456492	-2.39209	0.0167526	0.038348506	down-regulated	protein_coding
TAGLN3	ENSMMUG00000000697	4.755752328	5.005188249	2.092727	2.391706	0.0167703	0.038382813	up-regulated	protein_coding
DTX3L	ENSMMUG00000016383	53.94333146	1.599886942	0.669073	2.391199	0.0167934	0.038429608	up-regulated	protein_coding
SCARA3	ENSMMUG00000021517	9.758407165	3.485229184	1.457605	2.391065	0.0167996	0.038437471	up-regulated	protein_coding
ITM2C	ENSMMUG00000006222	145.3916955	-1.386202167	0.579994	-2.39003	0.0168472	0.038536208	down-regulated	protein_coding
RRAGC	ENSMMUG00000023634	51.74467795	-1.594158604	0.667011	-2.39	0.0168482	0.038536208	down-regulated	protein_coding
TAF4	ENSMMUG00000008696	31.63879104	-1.711967971	0.716575	-2.3891	0.0168898	0.038612742	down-regulated	protein_coding
PSD	ENSMMUG00000002993	6.813300326	2.820952426	1.18108	2.388452	0.0169195	0.038674345	up-regulated	protein_coding
CDKN2C	ENSMMUG00000019057	30.8342063	1.846469568	0.773231	2.387991	0.0169408	0.038716667	up-regulated	protein_coding
COX5A	ENSMMUG00000011677	196.6995717	-1.225354208	0.513163	-2.38785	0.0169474	0.038725533	down-regulated	protein_coding
UBE2G2	ENSMMUG00000006759	28.92211801	-1.884049469	0.789211	-2.38726	0.0169747	0.038781653	down-regulated	protein_coding
TMEM178	ENSMMUG00000011840	3.893241639	4.392505962	1.840394	2.38672	0.0169994	0.038831923	up-regulated	protein_coding
DNAI1	ENSMMUG00000020777	5.065925509	3.369120569	1.41238	2.38542	0.0170596	0.038956818	up-regulated	protein_coding
DCST1	ENSMMUG00000023339	4.220567451	2.994868792	1.255748	2.384929	0.0170824	0.039002601	up-regulated	protein_coding
CCDC58	ENSMMUG00000011919	28.81432782	-2.181691007	0.915128	-2.38403	0.0171243	0.039091932	down-regulated	protein_coding
ELMSAN1	ENSMMUG00000004913	75.06954032	1.190515724	0.499501	2.383408	0.0171532	0.03915148	up-regulated	protein_coding
ZNF146	ENSMMUG00000028785	51.18341256	-1.369150331	0.574467	-2.38334	0.0171562	0.039152194	down-regulated	protein_coding
HIST1H2AH	ENSMMUG00000029902	4.139229404	3.392571044	1.423901	2.382589	0.0171914	0.039219733	up-regulated	protein_coding
RASIP1	ENSMMUG00000018415	9.039353768	2.373370417	0.996393	2.381961	0.0172207	0.039280333	up-regulated	protein_coding
SNX13	ENSMMUG00000021915	41.39955269	-1.704089603	0.715505	-2.38166	0.0172348	0.039306182	down-regulated	protein_coding
NT5DC1	ENSMMUG00000014638	77.03081531	-1.148634704	0.482365	-2.38125	0.0172538	0.039343103	down-regulated	protein_coding
C19ORF50	ENSMMUG00000016078	115.4580304	-1.214896118	0.51021	-2.38117	0.0172578	0.039345871	down-regulated	protein_coding
SLC25A17	ENSMMUG00000008106	22.67745011	-2.304809112	0.968285	-2.3803	0.0172985	0.039425969	down-regulated	protein_coding
SEMA5B	ENSMMUG00000010034	52.68267769	1.58380771	0.665399	2.380237	0.0173015	0.039426501	up-regulated	protein_coding
MAP3K13	ENSMMUG00000023814	43.91295484	1.716397483	0.721145	2.380099	0.017308	0.039434885	up-regulated	protein_coding
YTHDF1	ENSMMUG00000009227	94.74876782	-1.186384893	0.498476	-2.38002	0.0173116	0.039436753	down-regulated	protein_coding
PYROXD1	ENSMMUG00000010854	119.2766913	1.165003785	0.48972	2.37892	0.0173635	0.039542184	up-regulated	protein_coding
TMEM205	ENSMMUG00000011313	64.46443462	-2.048734284	0.861459	-2.37822	0.0173967	0.039605052	down-regulated	protein_coding
TRABD	ENSMMUG00000014087	114.2625953	-1.478569083	0.621981	-2.37719	0.017445	0.039708707	down-regulated	protein_coding
TUBGCP5	ENSMMUG00000005533	27.56779557	-1.965213214	0.826734	-2.37708	0.0174503	0.039714344	down-regulated	protein_coding
ANK1	ENSMMUG00000014726	12.88257015	2.752018134	1.157888	2.376758	0.0174656	0.039742717	up-regulated	protein_coding
SPATS2	ENSMMUG00000003762	40.29723795	1.147282409	0.482921	2.375714	0.017515	0.039842433	up-regulated	protein_coding
RAD51C	ENSMMUG00000009133	14.27423922	-3.145255221	1.32415	-2.3753	0.0175347	0.039880697	down-regulated	protein_coding
HINT3	ENSMMUG00000004291	22.73943244	-2.258244956	0.951042	-2.3745	0.0175729	0.039948492	down-regulated	protein_coding
DDX50	ENSMMUG00000001006	42.96956339	-1.461659409	0.615706	-2.37396	0.0175986	0.040000379	down-regulated	protein_coding
DUSP22	ENSMMUG00000018514	13.91679863	-3.106975348	1.309125	-2.37332	0.0176289	0.040056465	down-regulated	protein_coding
PCGF6	ENSMMUG00000008873	7.580828042	-3.288798301	1.386446	-2.37211	0.0176869	0.040181846	down-regulated	protein_coding
RABGAP1L	ENSMMUG00000001565	59.38793906	1.200335323	0.506301	2.370793	0.01775	0.040318578	up-regulated	protein_coding
ASL	ENSMMUG00000011871	59.41498746	-1.550696871	0.654167	-2.37049	0.0177645	0.040344978	down-regulated	protein_coding
ZNF555	ENSMMUG00000007485	7.135523228	-3.20273373	1.351429	-2.36989	0.0177935	0.040404507	down-regulated	protein_coding
CYBRD1	ENSMMUG00000007295	563.184297	-1.398565454	0.590233	-2.36951	0.0178116	0.040432478	down-regulated	protein_coding
NOTCH4	ENSMMUG00000018461	8.374655087	2.789144679	1.177068	2.369569	0.0178088	0.040432478	up-regulated	protein_coding
TACSTD2	ENSMMUG00000022763	14.07524807	-3.1324961	1.322148	-2.36925	0.0178243	0.040454872	down-regulated	protein_coding
CLUAP1	ENSMMUG00000007569	7.506312963	-3.27498445	1.382355	-2.36913	0.0178298	0.040460931	down-regulated	protein_coding
ARHGDIG	ENSMMUG00000000603	11.04445148	-3.832645749	1.618044	-2.36869	0.0178512	0.040503003	down-regulated	protein_coding
DOLPP1	ENSMMUG00000032030	7.357977796	-3.24989391	1.372197	-2.36839	0.0178658	0.040529631	down-regulated	protein_coding
PIGL	ENSMMUG00000030296	13.02240731	-3.005467646	1.26918	-2.36804	0.0178826	0.040561287	down-regulated	protein_coding
HARS	ENSMMUG00000004481	36.31543755	-1.718492461	0.725834	-2.36761	0.0179034	0.040601796	down-regulated	protein_coding
POLR2J	ENSMMUG00000012019	74.56334761	-1.704604048	0.720042	-2.36737	0.0179152	0.040622095	down-regulated	protein_coding
DHRS7	ENSMMUG00000002540	214.1701905	-1.437907697	0.607527	-2.36682	0.0179416	0.040668894	down-regulated	protein_coding
SYNGR2	ENSMMUG00000021864	588.3290847	1.255672251	0.530778	2.36572	0.0179951	0.04078361	up-regulated	protein_coding
ARL4A	ENSMMUG00000010846	117.7723862	-1.171997411	0.495506	-2.36526	0.0180176	0.040828249	down-regulated	protein_coding
CDC42EP4	ENSMMUG00000018148	93.60841677	-1.072410008	0.453416	-2.36518	0.0180214	0.040830176	down-regulated	protein_coding
ADPRH	ENSMMUG00000000900	54.40361049	-1.346682095	0.569579	-2.36435	0.0180619	0.040908802	down-regulated	protein_coding
HIST1H3A	ENSMMUG00000029912	11.5847683	2.155888154	0.912049	2.363787	0.0180892	0.04096423	up-regulated	protein_coding
ARMC7	ENSMMUG00000029833	62.69213766	1.324991997	0.560557	2.363707	0.0180931	0.040966522	up-regulated	protein_coding

MOCS1	ENSMMUG00000021315	8.798099795	-3.5057908	1.483722	-2.36284	0.0181357	0.04105639	down-regulated	protein_coding
SFMBT1	ENSMMUG00000014527	7.062350836	-3.190099565	1.350698	-2.36182	0.0181857	0.04115633	down-regulated	protein_coding
NTHL1	ENSMMUG00000002807	15.97659572	-3.324950061	1.407922	-2.3616	0.0181962	0.041173481	down-regulated	protein_coding
CCDC28A	ENSMMUG00000017643	21.94869104	-2.250328833	0.953561	-2.35992	0.0182788	0.0413472	down-regulated	protein_coding
DYNC2LI1	ENSMMUG00000008077	20.32944568	-2.774121489	1.17604	-2.35887	0.0183309	0.041451688	down-regulated	protein_coding
COPE	ENSMMUG00000023291	310.2720262	-1.195553206	0.506833	-2.35887	0.0183307	0.041451688	down-regulated	protein_coding
TMEM242	ENSMMUG00000011814	22.47898109	-2.331738923	0.988647	-2.35851	0.0183483	0.041471186	down-regulated	protein_coding
THAP5	ENSMMUG00000011834	7.997802423	-3.370586406	1.429054	-2.35861	0.0183434	0.041471186	down-regulated	protein_coding
PACRGL	ENSMMUG00000017670	13.20626339	-3.036020205	1.287228	-2.35857	0.0183454	0.041471186	down-regulated	protein_coding
TTPAL	ENSMMUG00000013968	51.29667686	1.378823379	0.5847	2.358171	0.0183652	0.041502905	up-regulated	protein_coding
PPP6C	ENSMMUG00000020549	58.1743723	-1.365216461	0.579557	-2.35562	0.0184918	0.041775627	down-regulated	protein_coding
MT1X	ENSMMUG00000013684	57.81828633	1.47346725	0.625573	2.355388	0.0185034	0.041795108	up-regulated	protein_coding
FGD2	ENSMMUG00000022193	29.76528226	1.477084049	0.627237	2.354905	0.0185274	0.041842694	up-regulated	protein_coding
MYO1C	ENSMMUG00000002371	275.0165101	-1.023491748	0.434746	-2.35423	0.0185613	0.041912435	down-regulated	protein_coding
NQO2	ENSMMUG00000008487	67.15538328	-1.800719381	0.765012	-2.35384	0.0185804	0.041948976	down-regulated	protein_coding
FGD6	ENSMMUG00000018901	96.08068769	-1.608397526	0.683325	-2.35378	0.0185836	0.041949462	down-regulated	protein_coding
MRPL21	ENSMMUG00000010124	31.67551354	-2.063788776	0.876999	-2.35324	0.0186106	0.041997103	down-regulated	protein_coding
TRMT13	ENSMMUG00000004243	18.40168233	1.965071377	0.835213	2.352779	0.0186337	0.04204252	up-regulated	protein_coding
OBSL1	ENSMMUG00000007481	8.477819219	3.480740446	1.479533	2.352594	0.018643	0.042056696	up-regulated	protein_coding
ICE1	ENSMMUG00000001063	63.60252811	-1.026011472	0.436154	-2.35241	0.0186522	0.042070887	down-regulated	protein_coding
Y_RNA	ENSMMUG00000026107	3.287227249	3.79062363	1.611529	2.352191	0.0186632	0.042088861	up-regulated	misc_RNA
UCK2	ENSMMUG00000000565	42.64123155	-1.759853249	0.748279	-2.35187	0.0186794	0.042118653	down-regulated	protein_coding
MFN1	ENSMMUG00000004079	103.0761606	-1.229538317	0.522954	-2.35114	0.018716	0.042194396	down-regulated	protein_coding
CEP85	ENSMMUG00000000644	77.79611145	1.337519767	0.56895	2.350855	0.0187303	0.042220066	up-regulated	protein_coding
DHX58	ENSMMUG00000014755	24.71917564	-1.981717571	0.843245	-2.35011	0.018768	0.042298179	down-regulated	protein_coding
WDR16	ENSMMUG00000009399	2.842835803	3.85053926	1.638582	2.349921	0.0187774	0.042312695	up-regulated	protein_coding
VAMP8	ENSMMUG00000030785	156.2654109	-1.060072994	0.451139	-2.34977	0.0187851	0.042323355	down-regulated	protein_coding
PTPRE	ENSMMUG00000005383	107.7801925	1.675699943	0.713161	2.349678	0.0187896	0.042326816	up-regulated	protein_coding
MFI2	ENSMMUG00000016584	28.91077789	2.040493099	0.86858	2.349228	0.0188124	0.042357825	up-regulated	protein_coding
FGA	ENSMMUG00000021303	4.421766596	5.118638399	2.17884	2.34925	0.0188113	0.042357825	up-regulated	protein_coding
ARL5B	ENSMMUG00000007453	24.65727247	-2.862562327	1.218932	-2.34842	0.0188534	0.042443297	down-regulated	protein_coding
SOS2	ENSMMUG00000007464	65.02129413	-1.707421723	0.727226	-2.34786	0.0188818	0.042500517	down-regulated	protein_coding
NAPA	ENSMMUG00000014727	130.4214697	-1.071569035	0.45646	-2.34756	0.0188967	0.042527331	down-regulated	protein_coding
UGCG	ENSMMUG00000021526	109.0035028	-1.821728588	0.776173	-2.34706	0.018922	0.042577553	down-regulated	protein_coding
SSC4D	ENSMMUG00000021065	2.768414618	3.871637619	1.650014	2.346428	0.0189544	0.042637546	up-regulated	protein_coding
ZNF234	ENSMMUG00000021473	32.11830721	1.277640908	0.544506	2.346421	0.0189547	0.042637546	up-regulated	protein_coding
STX18	ENSMMUG00000020204	39.09382276	-1.555236055	0.663255	-2.34485	0.0190346	0.042810441	down-regulated	protein_coding
CNTN2	ENSMMUG00000018797	14.20417188	2.186135992	0.932537	2.344288	0.0190635	0.042868553	up-regulated	protein_coding
GLRX2	ENSMMUG00000022645	15.29241796	-3.258204176	1.390322	-2.34349	0.0191043	0.04294684	down-regulated	protein_coding
CDH24	ENSMMUG00000006009	8.902911028	-3.520570442	1.502338	-2.34339	0.0191092	0.042950919	down-regulated	protein_coding
GCFC1	ENSMMUG00000012291	58.85051939	-1.481401069	0.632273	-2.34298	0.0191306	0.042992307	down-regulated	protein_coding
PSME1	ENSMMUG00000012462	180.9470852	-1.573055756	0.67143	-2.34284	0.0191374	0.043000656	down-regulated	protein_coding
MYL9	ENSMMUG00000016447	15.75756934	-3.304609241	1.411283	-2.34156	0.0192031	0.043134671	down-regulated	protein_coding
NKRF	ENSMMUG00000022670	18.128049	-2.616931357	1.117802	-2.34114	0.0192249	0.043176741	down-regulated	protein_coding
KCNH4	ENSMMUG00000006401	5.871487788	3.554208033	1.519101	2.339679	0.0193003	0.043332337	up-regulated	protein_coding
TRAM2	ENSMMUG00000013718	35.03914465	1.4944617	0.639196	2.338032	0.0193856	0.043516865	up-regulated	protein_coding
TSEN54	ENSMMUG00000022123	137.4054733	1.32059569	0.564912	2.337703	0.0194027	0.043534457	up-regulated	protein_coding
ERO1L	ENSMMUG00000029350	262.6591538	-1.637838738	0.700603	-2.33776	0.0193999	0.043534457	down-regulated	protein_coding
FAM101A	ENSMMUG00000023105	89.03011015	-1.135456942	0.486038	-2.33615	0.0194835	0.04370891	down-regulated	protein_coding
WDR45	ENSMMUG00000010620	74.66354424	-1.202192396	0.514681	-2.3358	0.0195016	0.043742541	down-regulated	protein_coding
ASF1B	ENSMMUG00000014135	33.5852281	-2.555619874	1.094218	-2.33557	0.0195138	0.043762973	down-regulated	protein_coding
NPM3	ENSMMUG00000015774	10.30536755	-3.731878429	1.59795	-2.33542	0.0195217	0.043773802	down-regulated	protein_coding
C20orf195	ENSMMUG00000006069	23.10732278	-2.370768243	1.016006	-2.33342	0.0196261	0.043987026	down-regulated	protein_coding
EXOC7	ENSMMUG00000008885	95.89401354	-1.354549393	0.580575	-2.33312	0.0196421	0.044015747	down-regulated	protein_coding
CIT	ENSMMUG00000019861	80.31485254	1.659679097	0.71149	2.332682	0.0196648	0.044059799	up-regulated	protein_coding
PBRM1	ENSMMUG00000017361	77.37209887	-1.096161286	0.470105	-2.33174	0.0197145	0.044164007	down-regulated	protein_coding
USP2	ENSMMUG00000008921	46.72819029	1.328340216	0.569752	2.331437	0.0197304	0.044192621	up-regulated	protein_coding
PDZD7	ENSMMUG00000022617	14.3594289	-3.088236267	1.324896	-2.33093	0.0197572	0.044245652	down-regulated	protein_coding
IL17REL	ENSMMUG00000005555	26.6902857	2.287603684	0.981621	2.330434	0.0197832	0.04428301	up-regulated	protein_coding
CCDC174	ENSMMUG00000014777	24.02634285	-1.954151801	0.838517	-2.33049	0.0197805	0.04428301	down-regulated	protein_coding
HOXA2	ENSMMUG00000012812	6.979232012	5.042009409	2.1638	2.330165	0.0197975	0.044307838	up-regulated	protein_coding
OBFC2A	ENSMMUG00000009460	21.27082552	-2.663254369	1.143288	-2.32947	0.0198342	0.04437599	down-regulated	protein_coding
ADGRB2	ENSMMUG00000023401	15.73135219	2.009443718	0.862708	2.329228	0.019847	0.044397589	up-regulated	protein_coding
FAM199X	ENSMMUG00000022281	19.52067803	-2.712693056	1.164674	-2.32914	0.0198515	0.044400711	down-regulated	protein_coding
TRPM7	ENSMMUG00000008575	49.34486173	-1.873905668	0.804602	-2.32899	0.0198598	0.044412283	down-regulated	protein_coding
MTFR1L	ENSMMUG00000007854	145.3002321	-1.041745944	0.447363	-2.32864	0.0198782	0.044439313	down-regulated	protein_coding
NUBP2	ENSMMUG00000018597	55.36918672	-2.260938872	0.971015	-2.32843	0.0198893	0.04445716	down-regulated	protein_coding
TTYH1	ENSMMUG00000006027	22.97758251	1.257887406	0.540384	2.327766	0.0199245	0.044528824	up-regulated	protein_coding
DUSP14	ENSMMUG00000002975	34.62832617	1.472093324	0.632466	2.327547	0.0199362	0.044547847	up-regulated	protein_coding
PCNXL4	ENSMMUG00000015443	21.61126186	1.653829151	0.710821	2.326646	0.0199841	0.044647953	up-regulated	protein_coding
LGI3	ENSMMUG00000013028	6.690364115	4.839081179	2.079981	2.326503	0.0199918	0.044657926	up-regulated	protein_coding
PSMB3	ENSMMUG00000012363	116.5812317	-1.720453014	0.739652	-2.32603	0.020017	0.044707239	down-regulated	protein_coding
FAM207A	ENSMMUG00000000953	14.34471562	-3.152351224	1.355344	-2.32587	0.0200256	0.044712368	down-regulated	protein_coding
PRR12	ENSMMUG00000012937	40.30501306	-1.439363141	0.618932	-2.32556	0.0200421	0.044742183	down-regulated	protein_coding
THUMPD2	ENSMMUG00000011841	24.32442491	-2.254744401	0.96982	-2.32491	0.0200767	0.044812312	down-regulated	protein_coding
PSMD10	ENSMMUG00000016981	22.00662489	-2.256326224	0.970938	-2.32386	0.0201329	0.044930607	down-regulated	protein_coding
SCNM1	ENSMMUG00000007900	63.06361901	1.224101077	0.52679	2.323699	0.0201416	0.044943005	up-regulated	protein_coding
C19orf38	ENSMMUG00000018981	4.356343668	3.279881852	1.411945	2.322954	0.0201816	0.04502522	up-regulated	protein_coding
AAK1	ENSMMUG00000007868	41.88420677	1.599434628	0.688666	2.322512	0.0202054	0.045063963	up-regulated	protein_coding
TSPO2	ENSMMUG00000021169	18.70868137	-3.511578745	1.511945	-2.32256	0.0202029	0.045063963	down-regulated	protein_coding
SERPINB10	ENSMMUG00000016618	7.066813147	3.6341454	1.564835	2.322382	0.0202124	0.045072504	up-regulated	protein_coding
SMIM8	ENSMMUG00000022464	6.999817717	-3.174323939	1.367431	-2.32138	0.0202665	0.045171798	down-regulated	protein_coding
ERLIN2	ENSMMUG00000000943	139.6615346	-1.327947244	0.572202	-2.32077	0.0202995	0.045238157	down-regulated	protein_coding
WDR18	ENSMMUG00000002638	43.89641449	-2.363979393	1.018798	-2.32036	0.0203213	0.045272621	down-regulated	protein_coding
AMPD3	ENSMMUG00000015785	44.49208655	-1.486526325	0.64075	-2.31998	0.020342	0.04530443	down-regulated	protein_coding
SOX6	ENSMMUG00000002753	40.21610009	2.587609919	1.11569	2.31929	0.0203793	0.045380338	up-regulated	protein_coding
FAM164A	ENSMMUG00000001653	11.31633254	1.772742546	0.764617	2.318472	0.0204237	0.045472068	up-regulated	protein_coding
NUDT22	ENSMMUG00000012941	64.56051441	-1.552184497	0.669567	-2.31819	0.0204389	0.045498807	down-regulated	protein_coding
CYP39A1	ENSMMUG00000019247	19.33609989	-2.531366258	1.09252	-2.317	0.0205038	0.045636087	down-regulated	protein_coding
DNAJC30	ENSMMUG00000011368	14.1127576	-3.060091136	1.321035	-2.31643	0.0205346	0.045697318	down-regulated	protein_coding
TMEM238	ENSMMUG00000028715	82.5291931	-2.000411821	0.863674	-2.31616	0.0205493	0.045722947	down-regulated	protein_coding
FLRT3	ENSMMUG00000002916	109.1725314	-1.231304301	0.531663	-2.31595	0.020561	0.04574186	down-regulated	protein_coding
JAG2	ENSMMUG00000001276	27.02089476	-2.157136458	0.931585	-2.31555	0.0205826	0.045775484	down-regulated	protein_coding
SPIRE2	ENSMMUG00000029062	86.42384646	1.139298963	0.492012	2.31559	0.0205807	0.045775484	up-regulated	protein_coding
ACOX3	ENSMMUG00000006234	18.1395709	-2.590166184	1.118661	-2.31542	0.0205902	0.045777882	down-regulated	protein_coding
ZNHIT1	ENSMMUG00000006951	220.762414	-1.179307671	0.509378	-2.31519	0.0206025	0.045798147	down-regulated	protein_coding
B3GALT6	ENSMMUG00000019519	26.91857772	-2.656268282	1.147464	-2.3149	0.0206182	0.04582573	down-regulated	protein_coding
NAV2	ENSMMUG00000016942	70.57672084	1.416256934	0.611898	2.314531	0.0206386	0.045863899	up-regulated	protein_coding
NHEJ1	ENSMMUG00000000786	19.97406942	1.6244964	0.701926	2.314341	0.020649	0.04586896	up-regulated	protein_coding
TMEM170A	ENSMMUG00000001559	50.26958496	-1.361366304	0.588214	-2.3144	0.0206455	0.04586896	down-regulated	protein_coding

VAMP1	ENSMMUG00000014262	37.91382687	1.763578226	0.762031	2.314312	0.0206506	0.04586896	up-regulated	protein_coding
SLC25A15	ENSMMUG00000022585	34.66333138	-2.11944438	0.916549	-2.31242	0.0207547	0.046078504	down-regulated	protein_coding
CTDSPL	ENSMMUG00000002907	41.02431645	1.214152549	0.525206	2.311763	0.0207907	0.046144043	up-regulated	protein_coding
PIWIL4	ENSMMUG00000008965	16.96497187	3.501850545	1.514793	2.311768	0.0207905	0.046144043	up-regulated	protein_coding
FAM76B	ENSMMUG00000014792	13.19838179	-2.952859212	1.277365	-2.31168	0.0207954	0.046147029	down-regulated	protein_coding
BLM	ENSMMUG00000019526	30.93223094	-1.795499603	0.777046	-2.31067	0.0208509	0.046255686	down-regulated	protein_coding
RWDD3	ENSMMUG00000004759	7.880454205	-3.345295202	1.448157	-2.31004	0.0208861	0.046326668	down-regulated	protein_coding
HTATSF1	ENSMMUG00000006450	94.13249882	-1.001938603	0.43386	-2.30936	0.0209236	0.046402486	down-regulated	protein_coding
HSD17B6	ENSMMUG00000000542	8.371714802	-3.433531673	1.487007	-2.30902	0.0209424	0.046418563	down-regulated	protein_coding
CNR1	ENSMMUG00000000763	2.930757516	4.28706796	1.856646	2.309039	0.0209414	0.046418563	up-regulated	protein_coding
ABCB11	ENSMMUG00000013832	2.930757516	4.28706796	1.856646	2.309039	0.0209414	0.046418563	up-regulated	protein_coding
ATP6V1A	ENSMMUG00000020740	30.03312041	-2.315792154	1.002945	-2.30899	0.020944	0.046418563	down-regulated	protein_coding
COX14	ENSMMUG00000016470	43.54278715	-1.537116727	0.665793	-2.3087	0.0209602	0.046447328	down-regulated	protein_coding
PARP14	ENSMMUG00000022569	16.75862261	-3.394916515	1.470915	-2.30803	0.0209975	0.046522604	down-regulated	protein_coding
CCDC51	ENSMMUG00000003077	41.14638063	-1.997654224	0.865566	-2.30792	0.0210037	0.04652918	down-regulated	protein_coding
PDGFC	ENSMMUG00000022900	35.27443635	-1.837232879	0.796287	-2.30725	0.0210409	0.046604105	down-regulated	protein_coding
GALK2	ENSMMUG00000009839	33.77774714	-1.861072323	0.806874	-2.30652	0.0210815	0.046679608	down-regulated	protein_coding
IGFBP6	ENSMMUG00000020338	13.66988009	-4.141655775	1.79573	-2.30639	0.0210888	0.046688332	down-regulated	protein_coding
SNORD63	ENSMMUG00000024424	6.655166615	5.076252715	2.201783	2.305519	0.0211375	0.046781548	up-regulated	snoRNA
SOWAHD	ENSMMUG00000010476	13.44028093	-2.980687384	1.293095	-2.30508	0.0211621	0.046821269	down-regulated	protein_coding
MARCH1	ENSMMUG00000010258	7.277000149	5.069858179	2.200244	2.304226	0.02121	0.046919931	up-regulated	protein_coding
DIS3L	ENSMMUG00000003693	69.34197262	1.56336703	0.678815	2.303084	0.0212741	0.047044667	up-regulated	protein_coding
RBM48	ENSMMUG00000015534	6.702468694	-3.114397259	1.352238	-2.30314	0.0212708	0.047044667	down-regulated	protein_coding
AXIN2	ENSMMUG00000016595	132.7623093	-1.591549916	0.691148	-2.30276	0.0212921	0.047072245	down-regulated	protein_coding
NUDT8	ENSMMUG00000018528	37.91792179	-2.019918433	0.877255	-2.30254	0.0213045	0.047092332	down-regulated	protein_coding
RBM4	ENSMMUG00000020363	69.60645102	-1.700197467	0.738484	-2.30228	0.0213194	0.047117754	down-regulated	protein_coding
TUBAL3	ENSMMUG00000017442	25.79780543	-2.429309622	1.055843	-2.30083	0.0214015	0.047291868	down-regulated	protein_coding
PTTG1	ENSMMUG00000023077	31.93240782	-2.928287162	1.273686	-2.29907	0.0215012	0.047497406	down-regulated	protein_coding
PRR3	ENSMMUG00000013893	30.4650544	1.887992441	0.821593	2.297966	0.0215637	0.047613114	up-regulated	protein_coding
MINA	ENSMMUG00000017872	46.19294769	2.024195426	0.880994	2.297627	0.021583	0.047648394	up-regulated	protein_coding
DZIP3	ENSMMUG00000000898	27.53052504	-2.030921737	0.884002	-2.29742	0.021595	0.047667386	down-regulated	protein_coding
PMVK	ENSMMUG00000002027	56.05810234	-2.134669022	0.92932	-2.29702	0.0216175	0.047709613	down-regulated	protein_coding
TMEM144	ENSMMUG00000004448	93.93959704	-1.657913878	0.721809	-2.29689	0.0216252	0.047719022	down-regulated	protein_coding
TBC1D4	ENSMMUG00000021197	63.35557071	1.193692964	0.519742	2.296701	0.0216358	0.047735084	up-regulated	protein_coding
FRK	ENSMMUG00000014635	216.406659	-1.346395345	0.586266	-2.29656	0.0216438	0.047745304	down-regulated	protein_coding
RWDD4A	ENSMMUG00000023650	22.94004268	-2.32823211	1.013898	-2.29632	0.0216577	0.047768544	down-regulated	protein_coding
SLC25A42	ENSMMUG00000019669	14.90512013	1.901718583	0.828269	2.296017	0.0216749	0.047791593	up-regulated	protein_coding
TBC1D14	ENSMMUG00000017000	69.99655161	-1.659126395	0.722668	-2.29584	0.0216853	0.047807035	down-regulated	protein_coding
PLLP	ENSMMUG00000008924	19.00153857	-2.668637201	1.162722	-2.29516	0.0217237	0.047876796	down-regulated	protein_coding
KALRN	ENSMMUG00000003851	8.108088996	2.328182332	1.014499	2.294908	0.0217384	0.047900505	up-regulated	protein_coding
SNTB1	ENSMMUG00000002607	39.81746929	1.682439428	0.733266	2.294445	0.0217649	0.047937773	up-regulated	protein_coding
COQ6	ENSMMUG00000011148	108.2870154	1.747772929	0.762006	2.293647	0.0218108	0.048031258	up-regulated	protein_coding
SLC25A30	ENSMMUG00000015667	20.90569518	-2.19571791	0.95755	-2.29306	0.0218446	0.048098215	down-regulated	protein_coding
RNF14	ENSMMUG00000009768	55.20623108	-1.819269043	0.793439	-2.29289	0.0218543	0.04811219	down-regulated	protein_coding
TIMM22	ENSMMUG00000008129	26.28246352	1.539733808	0.671558	2.292777	0.0218608	0.048119035	up-regulated	protein_coding
UBE2Z	ENSMMUG00000021085	19.15174396	-2.717592373	1.185456	-2.29245	0.02188	0.048153624	down-regulated	protein_coding
PDF	ENSMMUG00000016636	82.69370522	-1.184305357	0.516712	-2.292	0.0219054	0.048202053	down-regulated	protein_coding
RBBP5	ENSMMUG00000018803	12.94292384	-2.997703099	1.307967	-2.29188	0.0219126	0.048210466	down-regulated	protein_coding
HMGB2	ENSMMUG00000003187	128.2551183	-1.815309283	0.792144	-2.29164	0.0219263	0.048233183	down-regulated	protein_coding
SLC2A4RG	ENSMMUG00000020711	97.18812663	-1.178570485	0.514354	-2.29136	0.0219427	0.048258021	down-regulated	protein_coding
SLC24A3	ENSMMUG00000002321	6.547991248	5.746006412	2.508039	2.291036	0.0219613	0.048287637	up-regulated	protein_coding
CCL28	ENSMMUG00000000179	9.468336146	-3.614081688	1.578009	-2.29028	0.0220052	0.04837646	down-regulated	protein_coding
FOXA3	ENSMMUG00000015438	68.00335376	-1.52595864	0.666298	-2.2902	0.0220096	0.048378614	down-regulated	protein_coding
NOB1	ENSMMUG00000006506	126.909812	1.459683564	0.637378	2.29014	0.0220132	0.048379175	up-regulated	protein_coding
ZNF446	ENSMMUG00000014693	24.5783903	1.994763513	0.87108	2.289988	0.022022	0.048390936	up-regulated	protein_coding
ZCCHC7	ENSMMUG00000007421	23.9049558	-1.924418405	0.840419	-2.28983	0.0220311	0.048395892	down-regulated	protein_coding
PABPC4	ENSMMUG00000007497	190.5957346	-1.234728785	0.53922	-2.28984	0.0220304	0.048395892	down-regulated	protein_coding
NAAA	ENSMMUG00000017501	127.6233979	-1.831348788	0.799889	-2.2895	0.0220502	0.048422728	down-regulated	protein_coding
NMNAT3	ENSMMUG00000000287	19.69398706	1.849110724	0.807672	2.289433	0.0220542	0.048424076	up-regulated	protein_coding
SLCO1A2	ENSMMUG00000008584	4.285440639	4.408820757	1.926388	2.288647	0.0220999	0.048516908	up-regulated	protein_coding
FBXO11	ENSMMUG00000022843	88.88130118	-1.006449803	0.439796	-2.28845	0.0221116	0.048534095	down-regulated	protein_coding
PGAP2	ENSMMUG00000003955	131.954649	1.001930696	0.437936	2.287849	0.0221463	0.048594422	up-regulated	protein_coding
VSIG1	ENSMMUG00000007189	5.771242943	3.170207496	1.385699	2.287804	0.0221489	0.048594422	up-regulated	protein_coding
TP53I11	ENSMMUG00000000369	42.70291357	1.117868238	0.488682	2.287518	0.0221656	0.048623377	up-regulated	protein_coding
RFX2	ENSMMUG00000011967	13.55313538	2.234190315	0.976753	2.287364	0.0221746	0.048635604	up-regulated	protein_coding
GPD2	ENSMMUG00000004897	83.30790634	-1.924174834	0.841379	-2.28693	0.0221998	0.048683442	down-regulated	protein_coding
MRPS30	ENSMMUG00000016180	66.58942377	-1.324518137	0.579302	-2.2864	0.0222306	0.048738136	down-regulated	protein_coding
TTC33	ENSMMUG00000023805	27.68100489	-1.732678631	0.758254	-2.28509	0.0223076	0.04888944	down-regulated	protein_coding
MIA3	ENSMMUG00000001021	100.1337441	-1.143305867	0.500645	-2.28367	0.0223911	0.049057134	down-regulated	protein_coding
SLC9A2	ENSMMUG00000004165	581.9615791	1.529416449	0.669842	2.283251	0.0224156	0.049103328	up-regulated	protein_coding
TMEM184C	ENSMMUG00000004977	53.35818021	-1.223824182	0.536221	-2.28231	0.0224709	0.049209192	down-regulated	protein_coding
NENF	ENSMMUG00000002142	79.45847326	-1.304220321	0.571575	-2.2818	0.0225012	0.049267866	down-regulated	protein_coding
POSTN	ENSMMUG00000012307	4.97982689	3.124943479	1.369654	2.281557	0.0225155	0.049291666	up-regulated	protein_coding
SLC38A9	ENSMMUG00000010466	12.72105961	-2.966836733	1.300552	-2.28121	0.0225359	0.049328554	down-regulated	protein_coding
CAMSAP1	ENSMMUG00000021497	22.09072855	-2.308861873	1.012307	-2.28079	0.0225607	0.049375327	down-regulated	protein_coding
MTF2	ENSMMUG00000016808	38.46139295	-1.739488089	0.76279	-2.28043	0.0225824	0.049415072	down-regulated	protein_coding
SLC16A10	ENSMMUG00000021534	14.3482725	-3.088768212	1.3547	-2.28004	0.0226054	0.049450201	down-regulated	protein_coding
PEX12	ENSMMUG00000006047	25.16052272	1.261103425	0.55315	2.279858	0.0226161	0.049465953	up-regulated	protein_coding
SUGP1	ENSMMUG00000028834	58.74030411	-1.519735988	0.666651	-2.27966	0.0226279	0.049484119	down-regulated	protein_coding
ZEB1	ENSMMUG00000015453	5.01768634	4.341806844	1.904806	2.279396	0.0226435	0.049510664	up-regulated	protein_coding
OGFR	ENSMMUG00000016856	82.53152043	-1.31229303	0.575754	-2.27926	0.0226516	0.049520596	down-regulated	protein_coding
ALDH1A2	ENSMMUG00000019504	5.814443468	3.45410258	1.516254	2.27805	0.0227236	0.049670319	up-regulated	protein_coding
GPR180	ENSMMUG00000013059	12.13134791	-2.895715262	1.271296	-2.27777	0.0227405	0.049693204	down-regulated	protein_coding
SNAI3	ENSMMUG00000008716	8.952248072	2.227451458	0.978005	2.277546	0.0227537	0.049698217	up-regulated	protein_coding
SLC46A1	ENSMMUG00000019151	22.25655178	1.773864583	0.778871	2.277483	0.0227574	0.049698217	up-regulated	protein_coding
GSDMA	ENSMMUG00000020381	12.56256713	2.370279145	1.040728	2.27752	0.0227552	0.049698217	up-regulated	protein_coding
TPST2	ENSMMUG00000023450	43.05183955	-1.243032795	0.545789	-2.2775	0.0227566	0.049698217	down-regulated	protein_coding
TCAF1	ENSMMUG00000005136	36.33560964	1.544062389	0.678012	2.277339	0.022766	0.049707358	up-regulated	protein_coding
CDKN3	ENSMMUG00000012323	15.59249697	-3.280407825	1.440692	-2.27697	0.0227882	0.049734749	down-regulated	protein_coding
ESPNL	ENSMMUG00000018486	17.05445576	3.438554491	1.510112	2.277019	0.0227851	0.049734749	up-regulated	protein_coding
THAP2	ENSMMUG00000020946	6.61845836	-3.096482968	1.360012	-2.27681	0.0227978	0.049748027	down-regulated	protein_coding
CPO	ENSMMUG00000014059	43.12158533	-3.587075452	1.575585	-2.27666	0.0228064	0.04975908	down-regulated	protein_coding
SBF2	ENSMMUG00000009205	168.4478673	1.016618881	0.446787	2.275401	0.0228819	0.049916109	up-regulated	protein_coding
UBN2	ENSMMUG00000002277	54.42372612	1.51870085	0.667587	2.274911	0.0229113	0.049964802	up-regulated	protein_coding
PPPDE1	ENSMMUG00000022310	34.53443742	1.193687709	0.524757	2.274744	0.0229213	0.049978922	up-regulated	protein_coding
