# Supplementary material for: Identification, Characterization, and Transcriptional Reprogramming of Epithelial Stem Cells and Intestinal Enteroids in Simian Immunodeficiency Virus Infected Rhesus Macaques
Source: Front Immunol. 2021 Nov 23;12:769990. doi: 10.3389/fimmu.2021.769990 (PMC8650114; doi:10.3389/fimmu.2021.769990)
Supplement: Supplementary file 10 [file Table_4.pdf]

Supplementary Table 4: The 284 enriched KEGG pathways for all DEGs

ID	pathway (#overlap genes)	pvalue	padj	gene list
mcc01100	Metabolic pathways (n=398)	7.69E-08	2.22E-05	CDA, XYLT2, ATP5C1, XYLT1, EPRS, ENO2, GLS, NSDHL, SCP2, PLCE1, EARS2, ACOT8, GLCE, UQCR, UPB1, UQCRC1, HPRT1, UQCRC2, LIPT2, TSTA3, MTMR3, SHMT2, ADH7, SDSL, PLA2G7, ACMSD, MTMR7, UGT8, ADH4, LDHA, RDH10, PNPO, ST3GAL5, LTA4H, ST3GAL3, PMM1, MCAT, PRDX6, DHFR, CS, UCK2, QARS, PPT1, PPT2, MOCS2, ADK, CSAD, PIGY, B3GALT2, ARG2, SPHK2, PGAM1, ITPK1, PIGC, HADHB, HADHA, PIGB, PLCB3, BDH2, BDH1, AGPS, ADSSL1, PIGK, ACSBG1, PIGL, DLD, ACSBG2, GART, PAFAH1B2, PIGH, PAFAH1B1, ASAH1, FH, OAT, GCNT1, ATP5A1, DLST, PTGS2, PAPSS1, PTGS1, FAM213B, EXTL1, ALOX5, GCNT3, CEPT1, GPAT2, AOC3, AOC1, MDH1, GK, AGK, LPCAT4, ETNK2, PLCD4, PLCD1, DGKG, CHPF, DGKA, SAT2, SAT1, TM7SF2, HPGDS, DGAT2, PCYT2, DGAT1, IPMK, MINPP1, ACLY, PGP, SMS, GAPDHS, ND1, ND3, ND2, ND5, ND4, ND6, ATP8, ATP6, AK2, GMPS, MRI1, NTPCR, AK5, AK7, UQCRH, AK8, TKFC, PMVK, PGK1, KDSR, FUK, UROS, GFPT1, ASNS, B3GALT6, UQCRQ, ACO1, COX4I1, AMD1, GADL1, TCIRG1, PIK3C2A, ALAD, UPP1, PGM1, IDH3A, PLA2G12B, HMGCS1, UGT1A1, AMT, NDUFC2, NT5C3B, TST, DAD1, IMPAD1, PCCB, DCTPP1, GAPDH, ALDH9A1, BCAT2, NDUFB9, NDUFB7, ACSS2, NDUFB6, NDUFB5, GLS2, NDUFB4, NDUFB2, PDHB, HSD17B10, CKMT2, NFS1, GANAB, POLR2B, POLR2D, POLR2E, POLR2G, BCO1, ACSS1, POLR2I, ADSS, POLR2K, POLR2L, NDUFA8, NDUFA7, NDUFA4, MMAB, NDUFA1, MBOAT1, GSTZ1, PFKL, SUCLA2, POLR3C, PSAT1, MGAT4A, POLR3H, NAGS, PFKM, PANK4, CMAS, PANK3, ACSM3, ACSM4, C1GALT1C1, ATP6V1E1, SEPHS1, GLUL, SEPHS2, ATP6V1G1, ALG8, ATP6V0E1, ALG9, OXSM, MCCC1, ALG3, TALDO1, MOGS, ACSL4, CMBL, INPP4A, BTD, SUCLG2, SUCLG1, LAP3, ATP6V0D1, CDS1, GCDH, MAOB, ATP5J, ATP5H, ATP5L, UGCG, SGPL1, PPCS, ATP5D, ASL, ARSB, ATP6V1C1, PCK2, PYCR1, PYCR2, ACADSB, ACER1, ALDH6A1, LDHAL6B, COX7B, HIBADH, ACY1, GPAA1, GBE1, AKR1B1, KHK, THTPA, SPTLC1, SPTLC2, SPTLC3, NMRK2, GUK1, NMRK1, DLAT, ACADM, ACADS, COX8A, PCYT1B, ATP6V0B, GAA, NME4, AMPD3, APRT, UGDH, NME7, B3GNT5, B3GNT4, B3GNT3, B3GNT2, ALDOC, ALDOB, ALDOA, ADH1A, HDC, HSD17B3, HSD17B4, HSD17B6, HSD17B8, COX5A, PTS, TRAK2, CYP17A1, MGAT3, MGAT1, ATP6V0E2, CBR3, GCH1, C1GALT1, FAH, GCK, NDUFAB1, PNPLA3, OTC, DPAGT1, ACAA2, NDUFA12, NDUFA10, CPOX, ENPP3, HADH, ENPP7, G6PD, HGD, ALG14, SDHC, SDHA, SDHB, COX6B1, ALDH3A2, DCTD, PLA2G16, DPM2, PHOSPHO1, PHOSPHO2, PLA2G10, ATP6V1A, IDI1, ALOX12, PLD1, PLD3, EBP, AANAT, ATP6V1H, COX11, ATP6V1D, NDUFV1, PDHA1, EPHX2, IDH1, IDH2, CRLS1, COQ6, QDPR, GCLC, AMACR, PI4KA, CRYL1, DEGS1, DEGS2, STT3B, GALK1, B4GALT2, PNLIPRP1, PYCRL, TREH, ATP5G3, ATP5G1, SGSH, HK1, PLCZ1, NAPRT, DBT, A4GALT, DCXR, PLA2G4C, CTPS2, ATP5F1, ALDH1A3, NDUFS8, ACOX2, NDUFS7, CHDH, ALDH1A2, ALDH1A1, NDUFS4, ATP6V1B2, NDUFS2, MVD, ACOX3, ATP6V1B1, DDC, PDXK, MAT2B, HMGCR, LTC4S, AGPAT2, AGPAT3, AGPAT4, POLD4, UGP2, MAT2A, CDIPT, GALNT7, RRM2, GALNT3, NOS3, B3GAT3, GNPDA2, GLB1, CHPT1, SCLY
mcc04142	Lysosome (n=51)	2.84E-05	2.91E-03	SCARB2, CLTA, CTSW, CTSV, TCIRG1, LIPA, CTSS, SGSH, AP4M1, MFSD8, LAPTM4A, GNPTAB, CTSL, CTSK, LAMP2, AP1S2, CTSH, AP1S1, AP3S1, AP1S3, CTSD, CTSC, CTSB, ARSA, ATP6V0B, ENTPD4, GAA, SLC11A2, AP1B1, SUMF1, NPC2, ATP6V0D1, ASAH1, CLN5, GGA1, AP3M1, NEU1, ATP6V1H, ARSB, CD164, NAPSA, DNASE2B, SORT1, M6PR, CTNS, NAGA, GLB1, PPT1, PPT2, GLA, LGMN
mcc01130	Biosynthesis of antibiotics (n=88)	3.02E-05	2.91E-03	ACAA2, PYCRL, ACY1, ENO2, TM7SF2, HK1, NSDHL, FNTB, DBT, DLAT, ACADM, HADH, PGM1, IDH3A, G6PD, ARG2, HMGCS1, PGAM1, AMT, TALDO1, SDHC, NME4, CMBL, AMPD3, SDHA, SDHB, HADHB, ALDH3A2, ACLY, HADHA, NME7, PCCB, CAT, PGP, SUCLG2, MVD, ALDOC, SUCLG1, ALDOB, ALDOA, GART, GAPDH, DLD, PCYOX1, ALDH9A1, BCAT2, GCDH, IDI1, FH, ACSS2, OAT, SHMT2, AK2, AK3, DLST, PDHB, AK5, HMGCR, SDSL, PLA2G7, AK7, HSD17B10, AK8, PAPSS1, LDHA, UGP2, PGK1, ASL, RCE1, ACSS1, PCK2, PDHA1, MDH1, TGDS, IDH1, GFPT1, IDH2, PYCR1, PYCR2, GCK, CS, PFKL, SUCLA2, PSAT1, ACO1, PFKM, OTC, LDHAL6B
mcc03050	Proteasome (n=25)	1.08E-04	7.82E-03	PSMD12, PSMD14, POMP, PSMB6, PSMB7, PSMB4, PSMD6, PSMB5, PSMD7, PSMB2, PSMB3, PSMD2, PSMB1, PSMD3, SHFM1, PSMB8, PSMC5, PSMA4, PSMA1, PSMC4, PSMA2, PSME3, PSME4, PSME1, PSME2
mcc00190	Oxidative phosphorylation (n=68)	2.79E-04	1.61E-02	COX7B, NDUFA12, COX4I1, NDUFA10, ATP5C1, ATP5G3, TCIRG1, ATP5G1, ATP6V1E1, COX8A, ATP6V0B, ATP6V1G1, ATP6V0E1, NDUFC2, SDHC, UQCR, SDHA, ATP5F1, SDHB, COX6B1, COX7A2L, NDUFS8, NDUFS7, PPA1, NDUFS4, ATP6V1B2, UQCRC1, NDUFS2, ND1, UQCRC2, ND3, ATP6V0D1, ND2, ND5, ND4, ATP6V1B1, ATP6V1A, ND6, NDUFB9, NDUFB7, NDUFB6, ATP8, NDUFB5, NDUFB4, ATP6, ATP5A1, NDUFB2, ATP5J, ATP5H, COX5A, UQCRH, ATP5L, ATP5D, COX11, ATP6V1H, ATP6V0E2, ATP6V1D, NDUFV1, ATP6V1C1, LHPP, NDUFA8, NDUFA7, NDUFA4, NDUFA1, ATP4B, ATP4A, UQCRQ, NDUFAB1
mcc00020	Citrate cycle (TCA cycle) (n=20)	6.68E-04	3.22E-02	FH, PDHA1, MDH1, IDH1, IDH2, DLST, SDHC, PDHB, SDHA, SDHB, CS, ACLY, SUCLA2, SUCLG2, SUCLG1, ACO1, DLAT, DLD, PCK2, IDH3A
mcc03060	Protein export (n=15)	9.39E-04	3.88E-02	HSPA5, SRP54, SRP68, SRP14, SEC61A2, IMMP1L, SEC61A1, IMMP2L, SPCS1, SEC61G, SEC61B, SEC62, SEC11A, SEC63, SEC11C

mcc03040	Spliceosome (n=53)	0.001281455	0.04355957	DDX42, HNRNPU, USP39, SNRNP70, MAGOH, DHX15, SNRPD3, TXNL4A, NCBP1, NCBP2, THOC1, THOC3, PRPF40A, PLRG1, WBP11, CDC40, DDX39B, SYF2, SRSF3, PPIH, SNRPA1, SNRPE, SRSF5, SNRPF, SNRPC, SLU7, RBM22, SNRPB, SF3B5, DDX5, SF3B3, RBM8A, SRSF1, U2AF1, ZMAT2, U2AF2, TRA2B, PCBP1, SNRPB2, HNRNPA1, SF3B1, PRPF38A, CCDC12, CDC5L, CWC15, LSM4, U2SURP, HNRNPM, PHF5A, LSM6, HNRNPK, ACIN1, PRPF31
mcc05010	Alzheimer's disease (n=77)	0.001356526	0.04355957	COX7B, NDUFA12, COX4I1, NDUFA10, ATP5C1, ATP2A2, ATP5G3, ATP5G1, RYR3, CASP9, CASP7, CASP8, CASP3, CAPN2, CAPN1, PSENEN, COX8A, MME, ADAM10, NDUFC2, SDHC, UQCR, SDHA, ATP5F1, SDHB, COX6B1, TNFRSF1A, BACE1, BACE2, ADAM17, COX7A2L, PLCB3, NDUFS8, NDUFS7, NDUFS4, UQCRC1, NDUFS2, UQCRC2, MAPT, GAPDH, NDUFB9, NDUFB7, NDUFB6, ATP8, NDUFB5, NDUFB4, ATP6, ATP5A1, NDUFB2, ATP5J, CACNA1C, ATP5H, COX5A, HSD17B10, UQCRH, APH1A, NCSTN, PPP3R1, ATP5D, NAE1, CACNA1S, FADD, BID, NDUFV1, MAPK3, NDUFA8, NDUFA7, NDUFA4, NDUFA1, GRIN2C, GRIN1, CDK5, UQCRQ, NDUFAB1, CYCS, CALM1, CDK5R1
mcc05016	Huntington's disease (n=81)	0.002069783	-	COX7B, NDUFA12, COX4I1, NDUFA10, CLTA, ATP5C1, ATP5G3, ATP5G1, DNALI1, CASP9, CASP8, CREB3L4, CASP3, AP2M1, COX8A, NDUFC2, SDHC, UQCR, SDHA, ATP5F1, SDHB, COX6B1, CREB3, COX7A2L, PLCB3, NDUFS8, NDUFS7, NDUFS4, PPIF, VDAC3, UQCRC1, VDAC2, NDUFS2, VDAC1, UQCRC2, SLC25A5, SLC25A4, NDUFB9, NDUFB7, DNAH2, DCTN2, NDUFB6, DNAH7, ATP8, NDUFB5, DNAH5, NDUFB4, ATP6, ATP5A1, DNAH6, NDUFB2, ATP5J, DNAH9, ATP5H, COX5A, UQCRH, POLR2B, ATP5D, POLR2D, POLR2E, POLR2G, POLR2I, DNAI1, NDUFV1, POLR2K, POLR2L, NDUFA8, NDUFA7, CREBBP, DNAH11, DNAH17, BDNF, NDUFA4, NDUFA1, SOD1, GRIN1, UQCRQ, NDUFAB1, BAX, CYCS, TAF4
mcc05012	Parkinson's disease (n=68)	0.002217667	-	COX7B, NDUFA12, COX4I1, NDUFA10, ATP5C1, PARK7, ATP5G3, ATP5G1, UBE2L3, CASP9, CASP3, SLC18A2, COX8A, NDUFC2, SDHC, UQCR, SDHA, ATP5F1, SDHB, COX6B1, COX7A2L, NDUFS8, NDUFS7, NDUFS4, PPIF, VDAC3, UQCRC1, VDAC2, NDUFS2, ND1, VDAC1, UQCRC2, ND3, SLC25A5, ND2, SLC25A4, ND5, ND4, ND6, NDUFB9, NDUFB7, NDUFB6, ATP8, NDUFB5, NDUFB4, ATP6, ATP5A1, NDUFB2, ATP5J, HTRA2, ATP5H, UBE2J2, COX5A, UQCRH, UBB, ATP5D, NDUFV1, NDUFA8, NDUFA7, NDUFA4, NDUFA1, UBE2G1, UBE2G2, PINK1, UQCRQ, NDUFAB1, CYCS, UBA1
mcc05215	Prostate cancer (n=35)	0.00418705	-	CDKN1A, TCF7, LEF1, PDGFA, PIK3R3, TGFA, PIK3CB, PIK3R1, HSP90B1, CASP9, IKBKB, NRAS, CREB3L4, AKT2, PDGFD, PDGFC, AKT3, HRAS, MAPK3, PDGFRB, TCF7L2, CREBBP, CHUK, EGF, PDPK1, NFKBIA, CREB3, CCNE2, CDK2, CTNNB1, GRB2, RAF1, SOS2, FGFR2, ATF4
mcc04141	Protein processing in endoplasmic reticulum (n=59)	0.004230868	-	UFD1L, TRAM1, PRKCSH, UBE2D2, NGLY1, UBE2D3, UBE2D1, HERPUD1, SEC61A2, SEC61A1, SEC61G, CAPN2, CAPN1, SEC61B, SEC62, MAP3K5, SEC63, PDIA3, SSR4, SSR2, TRAF2, MOGS, RAD23B, RBX1, DNAJC1, DAD1, DDIT3, CRYAB, ATF4, SAR1B, DERL1, CUL1, UBE2J2, RNF5, HSP90B1, LMAN1, GANAB, HSPH1, OS9, SPATA5L1, SSR1, SEC23B, SEC31A, BCAP31, MBTPS1, HSPA5, AMFR, EDEM1, EDEM2, UBE2G1, UBE2G2, SVIP, DNAJA1, DNAJC10, BAX, STUB1, STT3B, MXD3, NFE2L2
mcc04210	Apoptosis (n=29)	0.004361069	-	XIAP, PIK3R3, PIK3CB, PIK3R1, CASP9, IKBKB, CASP7, CASP8, CASP10, CASP3, AKT2, AKT3, CAPN2, TNFSF10, CAPN1, FADD, BID, NTRK1, DFFA, ENDOG, CHUK, TRAF2, TNFRSF1A, NFKBIA, BAX, CYCS, BIRC2, BCL2L1
mcc00071	Fatty acid degradation (n=20)	0.004675068	-	GCDH, ACAA2, ADH1A, ECI1, ECI2, ACSL4, ADH7, CPT1B, ACADSB, HADHB, ALDH3A2, ADH4, HADHA, ACSBG1, ACOX3, ACADM, HADH, ACSBG2, ACADS, ALDH9A1
mcc03013	RNA transport (n=59)	0.004931888	-	POP7, NUP188, FMR1, PHAX, NXT2, XPO1, SUMO1, SUMO3, MAGOH, XPO5, EIF2B1, NUP133, EIF1AY, NCBP1, NCBP2, PABPC4, THOC1, THOC3, THOC5, THOC7, THOC6, SRRM1, EEF1A1, NUP210L, DDX39B, CLNS1A, PABPC3, STRAP, NUP54, GEMIN7, EIF4E2, EIF1B, KPNB1, NUP205, RBM8A, NMD3, FXR1, TPR, EIF4EBP1, SMN1, EIF4E, PAIP1, RANBP2, EIF2B5, UBE2I, UPF3A, EIF1, EIF2S3, EIF5, EIF3I, EIF3J, ACIN1, EIF3H, RNPS1, EIF3E, EIF3F, EIF3D, RAN, EIF4G2
mcc04722	Neurotrophin signaling pathway (n=46)	0.00800133	-	YWHAE, CAMK2D, SHC2, SHC1, MAGED1, ARHGDIG, PIK3R3, PIK3CB, PIK3R1, NTF4, RAP1B, IKBKB, RPS6KA3, NRAS, RAP1A, ARHGDIA, AKT2, AKT3, RPS6KA1, RAC1, HRAS, SH2B3, MAPK3, SH2B1, MAP3K5, NTRK1, NTRK2, RIPK2, SORT1, KIDINS220, BDNF, PDPK1, NTRK3, PRKCD, RHOA, MAPK12, NFKBIA, CAMK4, BAX, GRB2, RAF1, CALM1, SOS2, ATF4, TP73
mcc04120	Ubiquitin mediated proteolysis (n=50)	0.010998472	-	UBE3C, UBE2D2, UBE2D3, CBLC, UBE2D1, UBE2Z, UBE2L3, HERC3, UBE2Q1, CDC26, PIAS4, FBXW8, PIAS1, RBX1, CDC34, UBE2R2, ANAPC4, BIRC2, ANAPC2, CUL7, CUL3, CUL2, CUL1, XIAP, RNF7, RCHY1, UBE2J2, ANAPC10, FZR1, UBR5, TCEB1, UBE2I, UBE2B, FANCL, SIAH1, UBE2G1, UBE2G2, UBE2A, KLHL9, CUL4A, RFWD2, CDC16, UBA3, UBA2, STUB1, UBA1, TRIM32, UBE2K, UBE2M
mcc04330	Notch signaling pathway (n=20)	0.011671739	-	PSENEN, PTCRA, JAG2, CREBBP, NOTCH1, MAML2, DTX3L, CTBP1, NOTCH4, DTX1, RFNG, DTX4, DLL3, APH1A, LFNG, ADAM17, NCSTN, DVL2, DVL3, MAML3
mcc01200	Carbon metabolism (n=46)	0.012886665	-	FH, ACSS2, SHMT2, DLST, PDHB, ENO2, SDSL, HK1, TKFC, ESD, PGK1, DLAT, ACADM, ACSS1, ACADS, IDH3A, G6PD, PDHA1, MDH1, IDH1, PGAM1, IDH2, AMT, TALDO1, SDHC, SDHA, SDHB, GCK, CS, HADHA, ALDH6A1, PFKL, SUCLA2, PSAT1, CAT, PCCB, PGP, SUCLG2, ALDOC, SUCLG1, ACO1, ALDOB, ALDOA, DLD, PFKM, GAPDH
mcc01230	Biosynthesis of amino acids (n=30)	0.013294338	-	ACY1, PYCRL, SHMT2, MAT2B, ENO2, SDSL, MAT2A, PGK1, ASL, GLUL, IDH3A, ARG2, IDH1, PGAM1, IDH2, PYCR1, TALDO1, PYCR2, CS, PFKL, PSAT1, ALDOC, ACO1, ALDOB, NAGS, ALDOA, PFKM, GAPDH, BCAT2, OTC
mcc05221	Acute myeloid leukemia (n=23)	0.015151837	-	CEBPA, TCF7L2, CHUK, JUP, LEF1, TCF7, PIK3R3, PIK3CB, PIK3R1, RUNX1, IKBKB, NRAS, RPS6KB1, AKT2, RPS6KB2, AKT3, EIF4EBP1, PIM1, GRB2, RAF1, HRAS, SOS2, MAPK3

mcc05231	Choline metabolism in cancer (n=36)	0.015993827	-	DGKG, SLC44A3, SLC22A5, DGKA, WAS, PDGFA, PIK3R3, PIK3CB, PIK3R1, PLD1, NRAS, AKT2, PDGFD, PDGFC, AKT3, EIF4EBP1, RAC1, HRAS, WASF3, MAPK3, PDGFRB, PCYT1B, LYPLA1, EGF, PDPK1, PLA2G4C, TSC2, TSC1, GPCPD1, RPS6KB1, RHEB, RPS6KB2, GRB2, CHPT1, RAF1, SOS2
mcc04146	Peroxisome (n=31)	0.018972412	-	ABCD4, PECR, ECI2, HSD17B4, MPV17L2, SCP2, PMVK, MPV17, ABCD1, NUDT13, PHYH, ACOT8, IDH1, EPHX2, IDH2, ECH1, ACSL4, PEX2, PEX13, SOD1, SLC25A17, AMACR, ACOX2, PEX6, CAT, AGPS, FAR1, ACOX3, FAR2, SLC27A2, PAOX
mcc05222	Small cell lung cancer (n=35)	0.019654751	-	ITGB1, LAMA2, LAMA4, LAMA3, XIAP, PIK3R3, LAMC2, PIK3CB, PIK3R1, PTGS2, FHIT, CASP9, IKBKB, RXRA, AKT2, AKT3, ITGAV, CHUK, ITGA3, LAMB2, FN1, LAMB1, TRAF2, NFKBIA, TRAF4, COL4A2, CCNE2, COL4A1, TRAF5, CDK2, RARB, CYCS, BIRC2, BCL2L1
mcc00600	Sphingolipid metabolism (n=19)	0.019824769	-	ARSA, ASAH1, CERK, SPHK2, UGCG, UGT8, ACER1, SGPL1, SPTLC1, SPTLC2, GLB1, SPTLC3, ACER3, NEU1, DEGS1, DEGS2, KDSR, GLA, ENPP7
mcc04932	Non-alcoholic fatty liver disease (NAFLD) (n=61)	0.019857187	-	COX7B, NDUFA12, COX4I1, NDUFA10, PRKAG1, PIK3CB, IKBKB, CASP7, CASP8, AKT2, CASP3, AKT3, LEPR, RAC1, MAP3K5, COX8A, SREBF1, NDUFC2, TRAF2, SDHC, UQCR, SDHA, SDHB, PRKAB1, COX6B1, TNFRSF1A, COX7A2L, NDUFS8, NDUFS7, DDIT3, NDUFS4, UQCRC1, NDUFS2, UQCRC2, ATF4, NDUFB9, CEBPA, NDUFB7, NDUFB6, NDUFB5, NDUFB4, NDUFB2, PIK3R3, PIK3R1, ADIPOR1, ADIPOR2, COX5A, UQCRH, RXRA, BID, NDUFV1, NDUFA8, NDUFA7, NDUFA4, INSR, NDUFA1, UQCRQ, NDUFAB1, BAX, CYCS, MAP3K11
mcc04114	Oocyte meiosis (n=41)	0.021296686	-	YWHAE, CAMK2D, YWHAB, CUL1, ADCY3, SMC3, PKMYT1, ADCY8, ADCY7, ADCY6, ANAPC10, PPP2CB, PPP1CC, RPS6KA3, PPP3R1, SLK, PTTG1, PLCZ1, CDC26, RPS6KA1, YWHAG, MAPK3, YWHAH, PLK1, PPP2R5B, PPP2R5A, PPP2R5D, CDC25C, YWHAZ, REC8, SMC1B, RBX1, CCNE2, CDK2, CDC16, ANAPC4, CPEB3, CALM1, CPEB2, ANAPC2
mcc05213	Endometrial cancer (n=21)	0.030912016	-	APC2, TCF7L2, EGF, PDPK1, LEF1, TCF7, PIK3R3, PIK3CB, AXIN2, PIK3R1, CASP9, NRAS, AKT2, AKT3, CTNNA1, CTNNB1, GRB2, RAF1, HRAS, SOS2, MAPK3
mcc05230	Central carbon metabolism in cancer (n=26)	0.032399814	-	GLS2, PIK3R3, PIK3CB, PDHB, SLC1A5, PIK3R1, GLS, HK1, NRAS, AKT2, AKT3, HRAS, MAPK3, PDGFRB, NTRK1, G6PD, PDHA1, NTRK3, PGAM1, SIRT6, GCK, SLC7A5, PFKL, RAF1, PFKM, FGFR2
mcc04966	Collecting duct acid secretion (n=13)	0.032600005	-	ATP6V1A, ATP6V1G1, ATP6V0E1, TCIRG1, ATP4B, ATP4A, ATP6V1B2, ATP6V1E1, ATP6V0D1, ATP6V0E2, ATP6V1D, ATP6V1C1, ATP6V1B1
mcc04152	AMPK signaling pathway (n=41)	0.032645588	-	PFKFB1, CAB39, PRKAG1, PIK3R3, PIK3CB, HMGCR, PIK3R1, ADIPOR1, ELAVL1, ADIPOR2, PPP2CB, CREB3L4, HNF4A, AKT2, AKT3, EIF4EBP1, LEPR, RAB8A, PCK2, RAB2A, SREBF1, PDPK1, INSR, STRADB, PPP2R5B, PPP2R5A, TSC2, PPP2R5D, TSC1, PPP2R3A, PRKAB1, CPT1B, RAB11B, CREB3, PFKL, RPS6KB1, RAB14, RHEB, RPS6KB2, PFKM, CFTR
mcc04150	mTOR signaling pathway (n=22)	0.036983056	-	CAB39, PDPK1, PIK3R3, TSC2, TSC1, PIK3CB, PIK3R1, IKBKB, RPS6KA3, RPS6KB1, RRAGC, RHEB, AKT2, RPS6KA1, DDIT4, RPS6KB2, AKT3, EIF4EBP1, ULK1, EIF4E2, EIF4E, MAPK3
mcc00280	Valine, leucine and isoleucine degradation (n=20)	0.039213902	-	ACAA2, HIBADH, HMGCS1, MCCC1, ACADSB, HSD17B10, AACS, HADHB, ALDH3A2, HADHA, ALDH6A1, OXCT1, PCCB, DBT, ACADM, HADH, DLD, ACADS, BCAT2, ALDH9A1
mcc01212	Fatty acid metabolism (n=18)	0.041248652	-	PECR, ACAA2, ELOVL5, OXSM, ACSL4, MCAT, CPT1B, ACADSB, HADHB, HADHA, PPT1, ACSBG1, ACOX3, PPT2, ACADM, HADH, ACSBG2, ACADS
mcc04962	Vasopressin-regulated water reabsorption (n=18)	0.041248652	-	NSF, DCTN6, DCTN5, DYNC1I2, RAB5B, RAB5C, DCTN2, ARHGDIG, ADCY3, ADCY6, RAB11A, RAB11B, DYNC2LI1, DYNC1LI1, CREB3, CREB3L4, ARHGDIA, VAMP2
mcc04130	SNARE interactions in vesicular transport (n=15)	0.043431496	-	STX8, STX18, STX10, VAMP8, VAMP7, STX5, VAMP1, SEC22B, VAMP4, VAMP5, YKT6, VAMP2, VTI1B, BET1, VAMP3
mcc05160	Hepatitis C (n=44)	0.04747011	-	SCARB1, CDKN1A, CD81, PIK3R3, PIK3CB, PIK3R1, CLDN1, IKBKB, CLDN22, PPP2CB, NRAS, CLDN20, RXRA, TBK1, AKT2, AKT3, CLDN23, HRAS, IKBKE, MAPK3, IFNAR2, CHUK, EGF, DDX58, PDPK1, TRAF2, TICAM1, MAPK12, PIAS1, TNFRSF1A, NFKBIA, CLDN4, OCLN, CLDN3, CLDN15, IRF3, PSME3, CLDN7, GRB2, EIF3E, RAF1, SOS2, IRF9, IFNAR1
mcc05216	Thyroid cancer (n=13)	0.057164506	-	NTRK1, TCF7L2, TPM3, LEF1, NCOA4, TCF7, NRAS, RXRA, TFG, TPR, CTNNB1, HRAS, MAPK3
mcc04320	Dorso-ventral axis formation (n=12)	0.057543263	-	PIWIL4, NOTCH1, NOTCH4, SPIRE2, GRB2, FMN2, CPEB3, CPEB2, SOS2, ETS2, MAPK3, ETV6
mcc04725	Cholinergic synapse (n=37)	0.065411415	-	ACHE, CAMK2D, CHRNA7, CACNA1B, ADCY3, CACNA1A, PIK3R3, PIK3CB, CACNA1C, PIK3R1, ADCY8, ADCY7, ADCY6, GNG10, NRAS, CREB3L4, AKT2, AKT3, CACNA1S, JAK2, HRAS, KCNJ2, MAPK3, KCNJ3, CHRNB2, KCNJ12, KCNJ14, GNG12, GNAO1, CREB3, PLCB3, CAMK4, GNB2, KCNQ1, GNB1, GNB3, ATF4
mcc04919	Thyroid hormone signaling pathway (n=38)	0.070327052	-	NOTCH1, THRA, SRC, NOTCH4, PIK3R3, PIK3CB, PIK3R1, ACTB, CASP9, NRAS, RXRA, PLCZ1, MED30, AKT2, AKT3, PLCE1, ITGAV, SLC16A2, HRAS, MAPK3, WNT4, MED1, CREBBP, PDPK1, NCOA3, TSC2, ATP1B3, SLC16A10, MED4, RCAN1, PLCB3, NCOR1, TBC1D4, RHEB, CTNNB1, RAF1, PLCD4, PLCD1
mcc04721	Synaptic vesicle cycle (n=23)	0.072444539	-	NAPA, NSF, ATP6V1A, ATP6V0B, ATP6V1G1, ATP6V0E1, CACNA1B, CLTA, CACNA1A, TCIRG1, DNM1, DNM3, ATP6V1B2, ATP6V1H, ATP6V1E1, ATP6V0D1, ATP6V0E2, ATP6V1D, SLC18A2, AP2M1, ATP6V1C1, VAMP2, ATP6V1B1
mcc04370	VEGF signaling pathway (n=22)	0.075770679	-	SHC2, SPHK2, SRC, NOS3, SH2D2A, PLA2G4C, PIK3R3, PIK3CB, PIK3R1, PTGS2, MAPK12, CASP9, NRAS, PPP3R1, MAPKAPK3, AKT2, AKT3, KDR, RAC1, RAF1, HRAS, MAPK3
mcc04915	Estrogen signaling pathway (n=32)	0.082718382	-	SHC2, SRC, SHC1, ADCY3, PIK3R3, PIK3CB, PIK3R1, ADCY8, ADCY7, ADCY6, GRM1, HSP90B1, NRAS, CREB3L4, AKT2, GPER1, AKT3, HRAS, MAPK3, KCNJ3, KCNJ9, NOS3, PRKCD, ESR2, GNAO1, CREB3, PLCB3, GRB2, RAF1, CALM1, SOS2, ATF4

mcc04066	HIF-1 signaling pathway (n=32)	0.082718382	-	CDKN1A, CAMK2D, TFRC, CUL2, PIK3R3, PIK3CB, PDHB, PIK3R1, ENO2, HK1, AKT2, MKNK2, AKT3, EIF4EBP1, TCEB1, TIMP1, EIF4E, MAPK3, CREBBP, EDN1, ANGPT2, PDHA1, EGLN2, EGF, NOS3, INSR, IFNGR2, RBX1, RPS6KB1, RPS6KB2, EIF4E2, GAPDH
mcc05220	Chronic myeloid leukemia (n=25)	0.088950553	-	CDKN1A, SHC2, SHC1, CTBP1, CBLC, PIK3R3, PIK3CB, PIK3R1, IKBKB, NRAS, MECOM, AKT2, AKT3, HRAS, MAPK3, SMAD4, CHUK, GAB2, RUNX1, BCR, NFKBIA, GRB2, RAF1, SOS2, BCL2L1
mcc00564	Glycerophospholipid metabolism (n=30)	0.09102221	-	CDS1, DGKG, ACHE, LPGAT1, DGKA, PLD1, AGPAT2, PLD3, AGPAT3, AGPAT4, GPAT2, CEPT1, CDIPT, PCYT1B, LYPLA1, PCYT2, PLA2G12B, PLA2G4C, MBOAT1, GPCPD1, CRLS1, PLA2G16, PHOSPHO1, ETNK2, LPCAT4, GPD2, PLA2G10, GPD1, CHPT1, PNPLA6
mcc00010	Glycolysis / Gluconeogenesis (n=29)	0.095524456	-	ACSS2, ADH1A, PDHB, ADH7, ENO2, HK1, ADH4, LDHA, PGK1, DLAT, ACSS1, PGM1, PCK2, PDHA1, PGAM1, GCK, ALDH3A2, ALDH1A3, MINPP1, PFKL, GAPDHS, ALDOC, ALDOB, ALDOA, DLD, PFKM, GAPDH, LDHAL6B, ALDH9A1
mcc04115	p53 signaling pathway (n=26)	0.097162531	-	CDKN1A, CD82, RCHY1, CASP9, CASP8, CASP3, PERP, PMAIP1, SFN, BID, RRM2, GADD45A, SIAH1, SHISA5, TSC2, SERPINB5, TP53I3, CCNE2, RFWD2, CCNG2, CCNG1, CDK2, ADGRB1, BAX, CYCS, TP73
mcc04012	ErbB signaling pathway (n=28)	0.100282202	-	CDKN1A, CAMK2D, SHC2, SRC, SHC1, CBLC, PIK3R3, TGFA, PIK3CB, PIK3R1, PAK1, NRAS, AKT2, AKT3, EIF4EBP1, HRAS, MAPK3, NCK1, PAK4, MAP2K4, EGF, EREG, RPS6KB1, NRG4, RPS6KB2, GRB2, RAF1, SOS2
mcc01210	2-Oxocarboxylic acid metabolism (n=8)	0.102112105	-	CS, ACY1, IDH1, IDH2, ACO1, NAGS, IDH3A, BCAT2
mcc04071	Sphingolipid signaling pathway (n=38)	0.108757898	-	ASAH1, PIK3R3, PIK3CB, PIK3R1, PLD1, PPP2CB, SGPL1, NRAS, SPTLC1, SPTLC2, SPTLC3, AKT2, AKT3, RAC1, BID, HRAS, CTSD, MAPK3, MAP3K5, ABCC1, SPHK2, PDPK1, NOS3, PPP2R5B, PPP2R5A, PPP2R5D, TRAF2, PPP2R3A, GAB2, RHOA, MAPK12, TNFRSF1A, ACER1, PLCB3, BAX, DEGS1, DEGS2, RAF1
mcc04010	MAPK signaling pathway (n=73)	0.123544535	-	PTPRR, DUSP16, ELK4, IKBKB, RPS6KA3, MECOM, AKT2, CASP3, RPS6KA1, AKT3, RAC1, HRAS, MAP3K5, MAP2K3, PDGFRB, MAP2K4, DUSP5, CHUK, DUSP1, PLA2G4C, RRAS2, CACNA2D2, TRAF2, CACNA2D4, DUSP6, MAPK8IP1, TNFRSF1A, PPM1A, CACNB2, MAPKAPK3, DDIT3, RASA1, RASA2, MAPT, RAF1, SOS2, ATF4, CACNA1B, PDGFA, CACNA1A, CACNA1C, CACNA1E, RASGRP1, RASGRP4, RELB, STK3, NTF4, RAP1B, CACNA1I, PAK1, NRAS, PPP3R1, RAP1A, MKNK2, CACNA1S, MAPK3, NTRK1, NTRK2, JUND, BDNF, EGF, GADD45A, NFATC3, GNG12, MAPK12, GRB2, MAP3K13, FGFR4, LAMTOR3, FGF12, MAP3K11, FGFR2, MAP3K12
mcc04666	Fc gamma R-mediated phagocytosis (n=28)	0.127519068	-	NCF1, ARPC1B, ARPC1A, ARPC5L, WAS, PIK3R3, PIK3CB, PIK3R1, PLD1, PAK1, AKT2, CFL1, AKT3, RAC1, WASF3, MAPK3, LYN, GSN, MYO10, SPHK2, PRKCD, ARPC5, GAB2, RPS6KB1, ARPC2, ARPC3, RPS6KB2, RAF1
mcc05211	Renal cell carcinoma (n=23)	0.128621633	-	CREBBP, FH, EGLN2, CUL2, PIK3R3, TGFA, PIK3CB, PIK3R1, RBX1, RAP1B, NRAS, PAK1, RAP1A, AKT2, AKT3, TCEB1, GRB2, RAC1, RAF1, HRAS, SOS2, MAPK3, PAK4
mcc04144	Endocytosis (n=72)	0.137153744	-	ARF3, VPS29, ARF1, TFRC, SH3KBP1, ARPC1B, ARPC1A, ARPC5L, CLTA, CBLC, RAB22A, CAPZB, PSD3, CHMP1A, VPS36, HRAS, RAB8A, AP2M1, SH3GLB1, PSD, SH3GLB2, PDCD6IP, VPS37C, VPS37B, RBSN, DNM1, RHOA, ARFGAP1, RNF41, DNM3, ACAP3, ACAP1, PARD3, CHMP4C, CHMP7, STAM2, ARF5, CHMP5, RAB5B, TSG101, RAB5C, SRC, VPS4B, WAS, AGAP1, PARD6G, VPS26A, SNX32, IL2RG, PLD1, SNX3, SNX4, SNX1, SNX2, GRK6, RAB11FIP4, ARFGEF1, RAB4A, VTA1, ARPC5, RAB11A, RAB11B, EHD1, EHD4, ARPC2, ARPC3, CAPZA1, CAPZA2, VPS45, FGFR4, FGFR2, SPG21
mcc04668	TNF signaling pathway (n=34)	0.145098513	-	CEBPB, PIK3R3, PIK3CB, PIK3R1, PTGS2, IKBKB, CASP7, CASP8, CREB3L4, CASP10, CASP3, AKT2, AKT3, FADD, DNM1L, MAPK3, MAP3K5, MAP2K3, MAP2K4, EDN1, CHUK, RIPK3, CCL20, TRAF2, TNFRSF1B, MAPK12, TNFRSF1A, NFKBIA, CREB3, CXCL10, MMP14, TRAF5, BIRC2, ATF4
mcc00230	Purine metabolism (n=53)	0.145302251	-	ADK, GUK1, NUDT16, ENPP3, PDE8B, PGM1, ENTPD3, ENTPD4, PDE4D, ENTPD6, NME4, AMPD3, APRT, NT5C3B, NME7, ADSSL1, HPRT1, GART, GUCY2C, PDE1B, NPR2, AK2, GMPS, ADCY3, AK3, AK5, NTPCR, ADCY8, FHIT, ADCY7, AK7, ADCY6, AK8, PAPSS1, POLD4, POLR2B, POLR2D, POLR2E, PDE6D, PDE6C, POLR2G, PDE6A, POLR2I, ADSS, POLR2K, POLR2L, RRM2, PNPT1, ADCY10, POLR3C, POLR3H, PDE7B, PDE7A
mcc03022	Basal transcription factors (n=15)	0.146502452	-	GTF2A2, TAF12, GTF2B, TAF9, GTF2F1, GTF2E2, TAF6L, CDK7, TAF7, TAF5, TAF4, TAF9B, MNAT1, TAF2, GTF2I
mcc04974	Protein digestion and absorption (n=28)	0.158838107	-	KCNK5, COL18A1, COL15A1, CPB1, COL13A1, COL14A1, SLC1A1, SLC3A1, SLC1A5, DPP4, COL10A1, KCNN4, SLC38A2, PRSS2, SLC15A1, MME, KCNJ13, ATP1B3, SLC16A10, SLC8A3, ACE2, COL4A2, MEP1B, CTRL, COL4A1, KCNQ1, COL6A3
mcc03015	mRNA surveillance pathway (n=30)	0.159448851	-	HBS1L, DAZAP1, RBM8A, NXT2, SMG7, PPP2CB, PPP1CC, PABPN1, FIP1L1, PCF11, MAGOH, CSTF1, PAPOLA, CPSF7, NCBP1, NCBP2, PABPC4, PPP2R5B, PPP2R5A, PPP2R5D, UPF3A, PPP2R3A, SRRM1, NUDT21, DDX39B, WDR82, PABPC3, ACIN1, RNPS1, ETF1
mcc00051	Fructose and mannose metabolism (n=13)	0.162924463	-	PFKFB1, TSTA3, PMM1, AKR1B1, KHK, HK1, PFKL, ENOSF1, ALDOC, ALDOB, ALDOA, FUK, PFKM
mcc00220	Arginine biosynthesis (n=9)	0.16586941	-	ARG2, ACY1, GLS2, NOS3, ASL, NAGS, GLUL, OTC, GLS
mcc04622	RIG-I-like receptor signaling pathway (n=22)	0.173215319	-	DDX3X, CHUK, DDX58, TRAF2, TANK, MAPK12, IKBKB, NFKBIA, CXCL10, SIKE1, TKFC, CASP8, TBK1, IRF3, CASP10, DHX58, IFNK, PIN1, FADD, AZI2, IKBKE, ATG5
mcc05100	Bacterial invasion of epithelial cells (n=26)	0.175633243	-	ITGB1, SHC2, SRC, SHC1, ARPC1B, ARPC1A, ARPC5L, WAS, CLTA, CBLC, PIK3R3, PIK3CB, PIK3R1, ACTB, CTNNA1, RAC1, FN1, ARPC5, DNM1, RHOA, CD2AP, DNM3, ARPC2, ARPC3, CTNNB1, ELMO3

mcc03018	RNA degradation (n=27)	0.184992393	-	ZCCHC7, BTG3, DIS3L, WDR61, TOB2, ENO2, TOB1, HSPD1, EXOSC7, EDC4, EXOSC9, NUDT16, TTC37, PNPT1, DIS3, PABPC4, LSM4, PFKL, LSM6, CNOT7, CNOT2, XRN2, PABPC3, CNOT8, PFKM, DCP2, SKIV2L2
mcc00563	Glycosylphosphatidylinositol(GPI)-anchor biosynthesis (n=10)	0.192252022	-	PIGC, PIGB, DPM2, GPAA1, PIGK, PIGL, PIGG, PIGY, GPLD1, PIGH
mcc00790	Folate biosynthesis (n=5)	0.193312786	-	QDPR, DHFR, MOCS2, GCH1, PTS
mcc04122	Sulfur relay system (n=5)	0.193312786	-	MOCS2, NFS1, CTU1, TST, URM1
mcc05412	Arrhythmogenic right ventricular cardiomyopathy (ARVC) (n=22)	0.194025705	-	ITGB1, RYR2, TCF7L2, ITGA4, JUP, ITGA3, LEF1, TCF7, CACNA2D2, CACNA2D4, CACNA1C, ACTB, CACNB2, CDH2, CTNNA1, ITGA7, CTNNB1, ITGAV, DSG2, ITGB7, CACNA1S, ITGB6
mcc00330	Arginine and proline metabolism (n=17)	0.201338923	-	AOC1, ARG2, OAT, PYCRL, MAOB, NOS3, AMD1, PYCR1, SAT2, PYCR2, SAT1, CKMT2, ALDH3A2, CARNS1, SMS, LAP3, ALDH9A1
mcc05223	Non-small cell lung cancer (n=19)	0.203535159	-	EGF, PDPK1, PIK3R3, TGFA, PIK3CB, PIK3R1, FHIT, EML4, CASP9, NRAS, RXRA, AKT2, AKT3, RARB, GRB2, RAF1, HRAS, SOS2, MAPK3
mcc04720	Long-term potentiation (n=22)	0.216008319	-	CREBBP, CAMK2D, CACNA1C, GRIN2C, ADCY8, GRM1, GRIN1, RAP1B, PPP1CC, RPS6KA3, NRAS, PLCB3, PPP3R1, RAP1A, CAMK4, RPS6KA1, RAF1, CALM1, HRAS, RAPGEF3, MAPK3, ATF4
mcc04662	B cell receptor signaling pathway (n=22)	0.216008319	-	LYN, CD72, CHUK, CD81, NFATC3, PIK3R3, DAPP1, PIK3CB, PIK3R1, IKBKB, NFKBIA, NRAS, PPP3R1, AKT2, AKT3, BLNK, GRB2, RAC1, RAF1, HRAS, SOS2, MAPK3
mcc03320	PPAR signaling pathway (n=21)	0.227812704	-	SLC27A1, GK, MMP1, PDPK1, APOC3, ACSL4, DBI, APOA5, CPT1B, FABP2, RXRA, ACOX2, SCP2, UBC, ACSBG1, ACOX3, ACADM, SLC27A2, ACSBG2, PCK2, SLC27A4
mcc04931	Insulin resistance (n=32)	0.227963438	-	SLC27A1, PRKAG1, PIK3R3, PIK3CB, PIK3R1, IKBKB, PPP1CC, RPS6KA3, CREB3L4, AKT2, AKT3, RPS6KA1, PPARGC1B, PCK2, PTPN1, SREBF1, PDPK1, NOS3, INSR, PRKCD, GFPT1, PRKAB1, CPT1B, TNFRSF1A, NFKBIA, CREB3, TBC1D4, RPS6KB1, RPS6KB2, PRKCQ, SLC27A2, SLC27A4
mcc00650	Butanoate metabolism (n=10)	0.229158543	-	HADHA, BDH2, HMGCS1, ACSM3, BDH1, OXCT1, ACSM4, HADH, ACADS, AACS
mcc00565	Ether lipid metabolism (n=14)	0.239628809	-	PLA2G12B, PLA2G4C, PLD1, PLA2G7, PLD3, PLA2G16, UGT8, LPCAT4, PLA2G10, AGPS, CHPT1, CEPT1, PAFAH1B2, PAFAH1B1
mcc04972	Pancreatic secretion (n=29)	0.241752134	-	PNLIPRP1, RYR2, CPB1, RAB3D, ADCY3, ATP2A2, ADCY8, ADCY7, ADCY6, RAP1B, RAP1A, RAC1, PRSS2, RAB8A, PLA2G12B, SCTR, ATP2B3, ATP1B3, ATP2B1, RHOA, RAB11A, PLCB3, CTRL, KCNQ1, PLA2G10, KCNMA1, SLC26A3, CFTR
mcc00062	Fatty acid elongation (n=9)	0.244238756	-	HADHB, ELOVL1, HADHA, ACAA2, ELOVL5, PPT1, ELOVL7, PPT2, HADH
mcc04151	PI3K-Akt signaling pathway (n=97)	0.247648913	-	YWHAE, ITGB1, CDKN1A, CSF3, CSF3R, YWHAB, LAMC2, PIK3CB, CASP9, IKBKB, CREB3L4, AKT2, AKT3, KDR, ITGB7, ITGAV, ITGB6, RAC1, JAK2, JAK3, HRAS, YWHAG, YWHAH, IFNAR2, PDGFRB, CHUK, ITGA4, ITGA3, PDPK1, F2R, PPP2R5B, PPP2R5A, TSC2, PPP2R5D, TSC1, YWHAZ, CREB3, COL4A2, CCNE2, COL4A1, DDIT4, COL6A3, ITGA7, EIF4E2, RAF1, SOS2, ATF4, IFNAR1, PHLPP2, LAMA2, LAMA4, LAMA3, PDGFA, PIK3R3, PIK3R1, IL2RG, THBS2, HSP90B1, PPP2CB, VTN, GNG10, NRAS, RXRA, PDGFD, PDGFC, CHAD, EIF4EBP1, SPP1, EIF4E, MAPK3, PCK2, MCL1, ANGPT2, LAMB2, EGF, NOS3, INSR, FN1, LAMB1, PPP2R3A, GNG12, EFNA2, RPS6KB1, LPAR6, RHEB, GNB2, RPS6KB2, CDK2, GNB1, GNB3, GRB2, PKN1, FGFR4, FGF12, FGFR2, BCL2L1
mcc04920	Adipocytokine signaling pathway (n=21)	0.252078208	-	CHUK, PRKAG1, ACSL4, TRAF2, TNFRSF1B, ADIPOR1, ADIPOR2, PRKAB1, CPT1B, TNFRSF1A, IKBKB, NFKBIA, RXRA, AKT2, AKT3, LEPR, ACSBG1, PRKCQ, JAK2, ACSBG2, PCK2
mcc04930	Type II diabetes mellitus (n=15)	0.255629784	-	KCNJ11, ABCC8, INSR, PRKCD, CACNA1B, PIK3R3, CACNA1A, PIK3CB, CACNA1C, PIK3R1, CACNA1E, GCK, HK1, IKBKB, MAPK3
mcc04914	Progesterone-mediated oocyte maturation (n=26)	0.256891661	-	ADCY3, PIK3R3, PIK3CB, PIK3R1, PKMYT1, ADCY8, ADCY7, ADCY6, ANAPC10, RPS6KA3, FZR1, AKT2, CDC26, AKT3, RPS6KA1, MAPK3, PLK1, CDC25C, MAPK12, CDK2, CDC16, ANAPC4, RAF1, CPEB3, CPEB2, ANAPC2
mcc00630	Glyoxylate and dicarboxylate metabolism (n=10)	0.268395035	-	CS, MDH1, SHMT2, CAT, PCCB, AMT, PGP, ACO1, DLD, GLUL
mcc00052	Galactose metabolism (n=12)	0.27113917	-	B4GALT2, PFKL, UGP2, GLB1, GAA, AKR1B1, GLA, PFKM, PGM1, GCK, HK1, GALK1
mcc00750	Vitamin B6 metabolism (n=4)	0.27375513	-	PDXK, PHOSPHO2, PSAT1, PNPO
mcc00561	Glycerolipid metabolism (n=19)	0.28081196	-	PNLIPRP1, DGKG, DGAT2, GK, DGAT1, MOGAT2, AGK, DGKA, MBOAT1, AKR1B1, AGPAT2, AGPAT3, AGPAT4, ALDH3A2, TKFC, PNPLA3, GPAT2, GLA, ALDH9A1
mcc00730	Thiamine metabolism (n=3)	0.286041929	-	THTPA, NFS1, NTPCR
mcc00510	N-Glycan biosynthesis (n=15)	0.286765942	-	B4GALT2, DPAGT1, ALG8, ALG9, ALG14, ALG3, MOGS, DPM2, GANAB, DAD1, DOLPP1, MGAT4A, MGAT3, MGAT1, STT3B
mcc04512	ECM-receptor interaction (n=27)	0.288350707	-	ITGB1, LAMA2, SDC4, LAMA4, LAMA3, LAMC2, THBS2, VTN, SV2A, CHAD, SPP1, ITGB7, ITGAV, ITGB6, ITGA4, ITGA3, LAMB2, FN1, LAMB1, GP1BA, COL4A2, COL4A1, ITGA7, SDC1, COL6A3, CD47, CD44
mcc05161	Hepatitis B (n=44)	0.293078629	-	CDKN1A, DDX3X, SRC, YWHAB, PIK3R3, PIK3CB, PIK3R1, CASP9, IKBKB, NRAS, CASP8, TBK1, CREB3L4, CASP10, CASP3, AKT2, AKT3, FADD, HRAS, IKBKE, MAPK3, MAP2K4, CREBBP, EGR2, SMAD4, CHUK, DDX58, NFATC3, TICAM1, YWHAZ, TIRAP, NFKBIA, CREB3, CCNE2, IRF3, CDK2, VDAC3, BAX, CYCS, GRB2, RAF1, MYD88, ATF4, IFNAR1
mcc04530	Tight junction (n=25)	0.293268039	-	SRC, PARD6G, F11R, CLDN1, ACTB, MYL12A, MYL12B, CLDN22, PPP2CB, CLDN20, CLDN23, MYH11, MPDZ, MYH10, MYH7B, MYH15, RHOA, CLDN4, OCLN, CLDN3, CLDN15, PARD3, CLDN7, RAB13, LLGL2
mcc03450	Non-homologous end-joining (n=6)	0.301432322	-	XRCC6, DCLRE1C, XRCC4, LIG4, DNTT, NHEJ1

mcc05200	Pathways in cancer (n=110)	0.301585356	-	CSF3R, CBLC, TFG, AKT2, AKT3, PDGFRB, RALBP1, TPM3, F2R, DAPK3, FLT3LG, RBX1, RUNX1, BCR, MSH6, COL4A2, CCNE2, COL4A1, RAF1, CTBP1, CUL2, TCF7, PDGFA, TGFA, PIK3R3, PIK3R1, RASGRP1, RASGRP4, HSP90B1, GNG10, TPR, DVL2, DVL3, FADD, WNT4, FZD1, CREBBP, SMAD4, FZD3, FZD5, JUP, FN1, GNG12, NFKBIA, LPAR6, GNB2, CDK2, GNB1, GNB3, CYCS, GRB2, FGF12, FGFR2, BCL2L1, ITGB1, CDKN1A, LAMC2, PIK3CB, CASP9, IKBKB, CASP8, MECOM, SUFU, CASP3, ITGAV, RAC1, HRAS, APC2, CHUK, ITGA3, MMP1, NCOA4, TRAF2, AXIN2, RHOA, PLCB3, TRAF4, TRAF5, RARB, SOS2, BIRC2, CEBPA, RALA, FH, RALB, LAMA2, LAMA4, LAMA3, LEF1, ADCY3, XIAP, PTGS2, ADCY8, ADCY7, ADCY6, NRAS, RXRA, CTNNA1, TCEB1, BID, MAPK3, NTRK1, TCF7L2, EGLN2, LAMB2, EGF, LAMB1, CTNNB1, BAX
mcc04540	Gap junction (n=26)	0.302432346	-	TUBAL3, SRC, HTR2B, PDGFA, ADCY3, ADCY8, ADCY7, ADCY6, GRM1, TUBA1B, NRAS, TUBA3C, PDGFD, PDGFC, TUBB1, HRAS, PRKG1, MAPK3, PDGFRB, EGF, CSNK1D, TUBA4A, PLCB3, GRB2, RAF1, SOS2
mcc04064	NF-kappa B signaling pathway (n=26)	0.302432346	-	XIAP, PTGS2, TNFSF13B, RELB, IKBKB, PLAU, BLNK, PIAS4, LYN, UBE2I, CHUK, CSNK2A1, DDX58, TRAF2, TICAM1, TIRAP, TNFRSF1A, NFKBIA, TRAF5, CSNK2B, PRKCQ, LTB, BIRC2, MYD88, BCL2L1
mcc02010	ABC transporters (n=14)	0.304570642	-	ABCD4, ABCC1, ABCC8, ABCC5, ABCA4, ABCB5, ABCA9, ABCA7, ABCA8, ABCB11, ABCA13, ABCB10, ABCD1, CFTR
mcc00900	Terpenoid backbone biosynthesis (n=8)	0.308575003	-	IDI1, HMGCS1, FNTB, PMVK, MVD, RCE1, HMGCR, PCYOX1
mcc04977	Vitamin digestion and absorption (n=8)	0.308575003	-	SCARB1, ABCC1, LMBRD1, BTD, RBP2, GIF, APOA4, MMACHC
mcc05014	Amyotrophic lateral sclerosis (ALS) (n=19)	0.308596506	-	MAP2K3, TOMM40, DERL1, TNFRSF1B, GRIN2C, MAPK12, GRIN1, SOD1, TNFRSF1A, CASP9, PPP3R1, CASP3, CAT, BAX, CYCS, RAC1, BID, BCL2L1, MAP3K5
mcc04520	Adherens junction (n=22)	0.313775792	-	FARP2, PTPN1, TCF7L2, SMAD4, CREBBP, CSNK2A1, SRC, INSR, LEF1, TCF7, CTNND1, WAS, ACTB, RHOA, PARD3, CSNK2B, CTNNA1, CTNNB1, RAC1, SSX2IP, WASF3, MAPK3
mcc00480	Glutathione metabolism (n=17)	0.31381621	-	GSTM4, GPX2, G6PD, RRM2, GPX4, GSTO1, MGST3, GSTP1, IDH1, IDH2, MGST1, MGST2, GCLC, GSTA4, GSTA3, SMS, LAP3
mcc05214	Glioma (n=20)	0.319730819	-	PDGFRB, CAMK2D, CDKN1A, SHC2, SHC1, EGF, PIK3R3, PDGFA, TGFA, PIK3CB, PIK3R1, NRAS, AKT2, AKT3, GRB2, RAF1, CALM1, HRAS, SOS2, MAPK3
mcc04921	Oxytocin signaling pathway (n=43)	0.322636131	-	RYR2, CDKN1A, CAMK2D, SRC, NPR2, PRKAG1, ADCY3, CACNA1C, PTGS2, ADCY8, ADCY7, ADCY6, ACTB, RYR3, PPP1CC, NRAS, PPP3R1, CACNA1S, HRAS, KCNJ2, MAPK3, KCNJ3, KCNJ12, KCNJ9, NOS3, PLA2G4C, KCNJ14, NFATC3, CACNA2D2, CACNA2D4, PRKAB1, RHOA, GNAO1, RCAN1, CACNB2, PLCB3, MYL6, CAMK4, CAMK1, RAF1, CALM1, CAMK1G
mcc00061	Fatty acid biosynthesis (n=5)	0.326672808	-	OXSM, ACSL4, ACSBG1, ACSBG2, MCAT
mcc04810	Regulation of actin cytoskeleton (n=57)	0.330200431	-	ITGB1, NCKAP1, ARPC1B, ARPC1A, ARPC5L, PIK3CB, ACTB, PPP1CC, TMSB4X, CFL1, ITGB7, ITGAV, ITGB6, RAC1, HRAS, APC2, PDGFRB, ITGA4, ITGA3, F2R, RRAS2, F2, RHOA, TIAM1, ITGAD, ITGA7, RAF1, PFN1, SOS2, PFN2, SRC, WAS, PDGFA, PIK3R3, PIK3R1, MYL12A, FGD1, MYL12B, FGD3, PAK1, NRAS, PDGFD, PDGFC, MAPK3, PAK4, GSN, EGF, FN1, ARPC5, GNG12, SSH3, ABI2, ARPC2, ARPC3, FGFR4, FGF12, FGFR2
mcc05210	Colorectal cancer (n=21)	0.330211832	-	APC2, TCF7L2, SMAD4, LEF1, TCF7, PIK3R3, PIK3CB, AXIN2, PIK3R1, RHOA, CASP9, MSH6, CASP3, AKT2, AKT3, BAX, CTNNB1, CYCS, RAC1, RAF1, MAPK3
mcc00620	Pyruvate metabolism (n=16)	0.332208715	-	FH, ACSS2, PDHA1, MDH1, GLO1, PDHB, ALDH3A2, LDHA, LDHD, HAGH, DLAT, ACSS1, DLD, PCK2, LDHAL6B, ALDH9A1
mcc00770	Pantothenate and CoA biosynthesis (n=7)	0.33295331	-	PANK4, PANK3, PPCS, GADL1, ENPP3, UPB1, BCAT2
mcc04620	Toll-like receptor signaling pathway (n=30)	0.335543752	-	CD80, PIK3R3, PIK3CB, PIK3R1, IKBKB, CASP8, TBK1, AKT2, CTSK, AKT3, SPP1, CCL3, RAC1, FADD, IKBKE, MAPK3, IFNAR2, MAP2K3, MAP2K4, CHUK, TICAM1, TIRAP, MAPK12, NFKBIA, CXCL10, IRF3, TOLLIP, IRF5, MYD88, IFNAR1
mcc04910	Insulin signaling pathway (n=39)	0.335605782	-	SHC2, SHC1, PRKAG1, CBLC, PIK3R3, PIK3CB, PIK3R1, HK1, IKBKB, PPP1CC, NRAS, PHKG1, AKT2, MKNK2, AKT3, EIF4EBP1, FLOT1, HRAS, EIF4E, MAPK3, PCK2, PTPN1, SREBF1, EXOC7, PDPK1, INSR, TSC2, TSC1, PRKAB1, GCK, RPS6KB1, PRKAR1A, RHEB, RPS6KB2, GRB2, EIF4E2, RAF1, CALM1, SOS2
mcc05169	Epstein-Barr virus infection (n=34)	0.342944752	-	CDKN1A, PSMD12, PSMD14, PIK3R3, PIK3CB, PIK3R1, RELB, IKBKB, PSMD6, PSMD7, TBK1, PSMD2, AKT2, PSMD3, AKT3, CD58, JAK3, MAP2K3, LYN, MAP2K4, USP7, CHUK, ENTPD3, DDX58, SHFM1, TRAF2, MAPK12, NFKBIA, PSMC5, IRF3, PSMC4, TRAF5, CDK2, CD44
mcc04014	Ras signaling pathway (n=64)	0.347245097	-	PIK3CB, ETS2, IKBKB, SYNGAP1, TBK1, AKT2, AKT3, KDR, PLCE1, RAC1, HRAS, PDGFRB, RALBP1, PLA2G12B, CHUK, PLA2G4C, RRAS2, GAB2, RHOA, PLA2G16, TIAM1, RASA1, PLA2G10, RASA2, RAF1, SOS2, EXOC2, RALA, RAB5B, SHC2, RALB, RAB5C, SHC1, PDGFA, PIK3R3, FOXO4, PIK3R1, PLD1, RASGRP1, RASGRP4, RAP1B, PAK1, GNG10, NRAS, RAP1A, PDGFD, PDGFC, MAPK3, PAK4, ANGPT2, EGF, INSR, GNG12, GRIN1, EFNA2, GNB2, GNB1, GNB3, GRB2, CALM1, FGFR4, FGF12, FGFR2, BCL2L1
mcc00920	Sulfur metabolism (n=4)	0.357356603	-	TST, IMPAD1, SQRDL, PAPSS1
mcc04923	Regulation of lipolysis in adipocytes (n=16)	0.364286103	-	INSR, PIK3R3, ADCY3, PIK3CB, PIK3R1, ABHD5, PTGS2, ADCY8, ADCY7, ADCY6, TSHR, PTGS1, PLA2G16, AKT2, AKT3, PRKG1
mcc05134	Legionellosis (n=19)	0.366246282	-	RAB1A, HBS1L, ARF1, SAR1B, IL18, HSPD1, EEF1A1, EEF1G, NFKBIA, CASP9, BCL2L13, CASP7, CASP8, SPATA5L1, CASP3, CYCS, SEC22B, MYD88, NAIP
mcc00640	Propanoate metabolism (n=11)	0.368688502	-	HADHA, ALDH6A1, LDHA, ACSS2, SUCLA2, PCCB, SUCLG2, SUCLG1, ACADM, ACSS1, LDHAL6B
mcc00410	beta-Alanine metabolism (n=11)	0.368688502	-	AOC3, ALDH3A2, ALDH1A3, HADHA, ALDH6A1, CARNS1, GADL1, SMS, ACADM, UPB1, ALDH9A1

mcc00030	Pentose phosphate pathway (n=9)	0.377706137	-	PFKL, G6PD, TALDO1, ALDOC, ALDOB, ALDOA, PFKM, PGM1, DERA
mcc04664	Fc epsilon RI signaling pathway (n=19)	0.395767743	-	MAP2K3, LYN, MAP2K4, PDPK1, PLA2G4C, PIK3R3, PIK3CB, GAB2, PIK3R1, MAPK12, NRAS, AKT2, AKT3, GRB2, RAC1, RAF1, HRAS, SOS2, MAPK3
mcc04917	Prolactin signaling pathway (n=20)	0.404896343	-	SHC2, SRC, SHC1, PIK3R3, PIK3CB, PIK3R1, MAPK12, ESR2, GCK, CYP17A1, NRAS, AKT2, AKT3, GRB2, JAK2, RAF1, HRAS, SOS2, MAPK3, SOCS5
mcc04062	Chemokine signaling pathway (n=52)	0.40604744	-	NCF1, PIK3CB, CXCL16, IKBKB, AKT2, AKT3, CCR7, RAC1, JAK2, JAK3, CCR4, HRAS, CHUK, RHOA, TIAM1, PLCB3, PARD3, RAF1, SOS2, SHC2, SRC, SHC1, WAS, CXCR5, ADCY3, PIK3R3, CXCR6, PIK3R1, ADCY8, ADCY7, ADCY6, RAP1B, PAK1, GNG10, NRAS, RAP1A, CCL7, GRK6, CCL3, MAPK3, CCL25, LYN, CCL23, CCL20, GNG12, NFKBIA, CXCL10, GNB2, GNB1, GNB3, GRB2, CCL28
mcc00534	Glycosaminoglycan biosynthesis - heparan sulfate / heparin (n=8)	0.40717233	-	HS3ST3B1, EXTL1, B3GAT3, GLCE, XYLT2, XYLT1, B3GALT6, HS6ST1
mcc04068	FoxO signaling pathway (n=36)	0.419020661	-	CDKN1A, FBXO25, PRKAG1, PIK3R3, PIK3CB, FOXO4, PIK3R1, GRM1, IKBKB, NRAS, AKT2, AKT3, TNFSF10, HRAS, MAPK3, PCK2, GABARAPL2, CREBBP, GABARAPL1, USP7, SMAD4, CHUK, EGF, GADD45A, PDPK1, INSR, PLK2, PLK1, PRKAB1, MAPK12, CCNG2, CDK2, CAT, GRB2, RAF1, SOS2
mcc00120	Primary bile acid biosynthesis (n=6)	0.421684098	-	CYP39A1, ACOT8, AMACR, ACOX2, SCP2, HSD17B4
mcc04020	Calcium signaling pathway (n=48)	0.422795453	-	RYR2, CAMK2D, PDE1B, CHRNA7, HTR2B, CACNA1B, ADCY3, CACNA1A, ATP2A2, CACNA1C, HTR4, ADCY8, ADCY7, CACNA1E, RYR3, GRM1, CACNA1I, PPP3R1, PLCZ1, PHKG1, PLCE1, CACNA1S, PDGFRB, AVPR1B, SPHK2, NOS3, F2R, TACR2, ATP2B3, ATP2B1, GRIN2C, GRIN1, SLC8A3, P2RX7, PLCB3, CCKBR, ADORA2B, CAMK4, VDAC3, PPIF, VDAC2, ORAI1, VDAC1, CALM1, SLC25A5, SLC25A4, PLCD4, PLCD1
mcc04510	Focal adhesion (n=57)	0.445124021	-	ITGB1, LAMC2, PIK3CB, ACTB, PPP1CC, AKT2, AKT3, KDR, CAPN2, ITGB7, ITGAV, ITGB6, RAC1, HRAS, PDGFRB, ITGA4, ITGA3, PDPK1, RHOA, COL4A2, COL4A1, COL6A3, ITGA7, TLN2, RAF1, SOS2, BIRC2, SHC2, LAMA2, SRC, SHC1, LAMA4, LAMA3, PDGFA, XIAP, PIK3R3, PIK3R1, THBS2, MYL12A, MYL12B, RAP1B, VTN, PAK1, RAP1A, PDGFD, CHAD, PDGFC, SPP1, MAPK3, PAK4, LAMB2, EGF, FN1, LAMB1, CTNNB1, GRB2
mcc03020	RNA polymerase (n=9)	0.469452256	-	POLR2B, POLR3C, POLR2D, POLR2E, POLR3H, POLR2G, POLR2I, POLR2K, POLR2L
mcc04110	Cell cycle (n=35)	0.499212566	-	YWHAE, CDKN1A, YWHAB, CUL1, SMC3, PKMYT1, ANAPC10, FZR1, PTTG1, CDC26, SFN, BUB3, E2F5, YWHAG, YWHAH, CREBBP, SMAD4, CDKN2C, GADD45A, PLK1, CDC25C, YWHAZ, SMC1B, RBX1, CDK7, STAG2, DBF4, CCNE2, TFDP2, CDK2, CDC16, ANAPC4, MCM6, ANAPC2
mcc05145	Toxoplasmosis (n=31)	0.502184616	-	ITGB1, LAMA2, LAMA4, LAMA3, XIAP, LAMC2, CASP9, IKBKB, CASP8, CASP3, ALOX5, AKT2, AKT3, JAK2, MAPK3, MAP2K3, CHUK, PDPK1, LAMB2, IFNGR2, LAMB1, MAPK12, TNFRSF1A, NFKBIA, GNAO1, PPIF, CYCS, BIRC2, MYD88, BCL2L1
mcc00072	Synthesis and degradation of ketone bodies (n=4)	0.51697257	-	BDH2, HMGCS1, BDH1, OXCT1
mcc04975	Fat digestion and absorption (n=11)	0.529826562	-	PNLIPRP1, FABP2, SCARB1, PLA2G12B, DGAT2, DGAT1, MOGAT2, PLA2G10, MTTP, APOA4, AGPAT2
mcc04145	Phagosome (n=43)	0.532571822	-	ATP6V1A, ITGB1, DYNC1I2, SCARB1, RAB5B, RAB5C, TUBAL3, TFRC, NCF1, C1R, STX18, TCIRG1, THBS2, ACTB, CTSS, SEC61A2, SEC61A1, TUBA1B, TUBA3C, CTSL, SEC61G, TUBB1, LAMP2, ATP6V1H, ITGAV, RAC1, SEC61B, ATP6V0E2, ATP6V1E1, ATP6V1D, ATP6V1C1, ATP6V0B, ATP6V1G1, ATP6V0E1, M6PR, TUBA4A, DYNC1LI1, ATP6V1B2, SEC22B, PLA2R1, ATP6V0D1, VAMP3, ATP6V1B1
mcc04912	GnRH signaling pathway (n=24)	0.540701464	-	MAP2K3, MAP2K4, CAMK2D, GNRHR2, SRC, PLA2G4C, ADCY3, CACNA1C, PLD1, ADCY8, ADCY7, ADCY6, MAPK12, MMP14, NRAS, PLCB3, GRB2, CACNA1S, RAF1, CALM1, HRAS, SOS2, MAPK3, ATF4
mcc04726	Serotonergic synapse (n=30)	0.543170319	-	DDC, MAOB, HTR2B, CACNA1B, CACNA1A, ALOX12, CACNA1C, HTR4, PTGS2, PTGS1, GNG10, NRAS, CASP3, ALOX5, KCNN2, CACNA1S, HRAS, SLC18A2, MAPK3, KCNJ3, KCNJ9, PLA2G4C, GNG12, GNAO1, PLCB3, GNB2, GNB1, GNB3, RAF1, RAPGEF3
mcc04727	GABAergic synapse (n=23)	0.561888554	-	NSF, GABARAPL2, GABARAPL1, GLS2, SRC, CACNA1B, ADCY3, CACNA1A, CACNA1C, GNG12, ADCY8, ADCY7, ADCY6, TRAK2, GLS, GNAO1, GNG10, GNB2, GNB1, GNB3, CACNA1S, SLC38A2, GLUL
mcc00980	Metabolism of xenobiotics by cytochrome P450 (n=17)	0.563252829	-	GSTM4, UGT1A1, GSTO1, MGST3, GSTP1, ADH1A, MGST1, MGST2, ADH7, ADH4, ALDH1A3, GSTA4, CYP2A24, GSTA3, CYP2F1, SULT2A1, CBR3
mcc05212	Pancreatic cancer (n=18)	0.567722367	-	SMAD4, RALBP1, RALA, RALB, CHUK, EGF, PIK3R3, TGFA, PIK3CB, PIK3R1, IKBKB, CASP9, AKT2, AKT3, RAC1, RAF1, MAPK3, BCL2L1
mcc00970	Aminoacyl-tRNA biosynthesis (n=12)	0.572939235	-	NARS, CARS, QARS, VARS, PARS2, RARS, IARS, HARS, EPRS, CARS2, EARS2, AARS
mcc05030	Cocaine addiction (n=13)	0.57734659	-	DDC, MAOB, BDNF, GRIN2C, GRIN1, CREB3, GRIN3A, CDK5, CREB3L4, RGS9, SLC18A2, CDK5R1, ATF4
mcc00533	Glycosaminoglycan biosynthesis - keratan sulfate (n=5)	0.589339368	-	B4GALT2, B3GNT7, B3GNT2, CHST4, ST3GAL3
mcc00100	Steroid biosynthesis (n=6)	0.591408477	-	EBP, NSDHL, SOAT1, SOAT2, LIPA, TM7SF2
mcc00340	Histidine metabolism (n=7)	0.593948399	-	ALDH3A2, ALDH1A3, AOC1, MAOB, HDC, CARNS1, ALDH9A1
mcc04976	Bile secretion (n=18)	0.596407314	-	SCARB1, SLC10A1, SCTR, NR1H4, ADCY3, ATP1B3, HMGCR, SLC51B, ABCB11, ADCY8, ADCY7, NR0B2, ADCY6, RXRA, SLCO1A2, KCNN2, CFTR, SULT2A1
mcc00780	Biotin metabolism (n=2)	0.601865044	-	BTD, OXSM

mcc03008	Ribosome biogenesis in eukaryotes (n=21)	0.606184189	-	POP7, CSNK2A1, IMP3, HEATR1, NMD3, SPATA5, NXT2, GNL3, RRP7A, FBL, EMG1, XPO1, NOB1, TCOF1, XRN2, CSNK2B, MPHOSPH10, DROSHA, RIOK2, RIOK1, RAN
mcc00520	Amino sugar and nucleotide sugar metabolism (n=13)	0.610945357	-	TSTA3, CMAS, PMM1, GFPT1, GCK, HK1, UGDH, CYB5R4, UGP2, GNPDA2, FUK, PGM1, GALK1
mcc00982	Drug metabolism - cytochrome P450 (n=16)	0.619080369	-	GSTM4, UGT1A1, MAOB, GSTO1, MGST3, GSTP1, ADH1A, MGST1, MGST2, FMO4, ADH7, FMO5, ADH4, ALDH1A3, GSTA4, GSTA3
mcc04916	Melanogenesis (n=26)	0.620661011	-	CAMK2D, TCF7, LEF1, ADCY3, ADCY8, ADCY7, ADCY6, NRAS, CREB3L4, DVL2, DVL3, HRAS, MAPK3, WNT4, FZD1, TCF7L2, CREBBP, EDN1, FZD3, FZD5, GNAO1, CREB3, PLCB3, CTNNB1, RAF1, CALM1
mcc04024	cAMP signaling pathway (n=50)	0.62500372	-	RYR2, PIK3CB, HTR4, PPP1CC, CREB3L4, AKT2, AKT3, PLCE1, SOX9, RAC1, PDE4D, F2R, RRAS2, ATP1B3, SSTR2, RHOA, CREB3, TIAM1, ORAI1, ACOX3, RAF1, CFTR, RAPGEF3, CAMK2D, ADCY3, PIK3R3, CACNA1C, PIK3R1, PLD1, ADCY8, ADCY7, ADCY6, RAP1B, PAK1, RAP1A, CACNA1S, MAPK3, CREBBP, BDNF, ATP2B3, ATP2B1, GRIN2C, TSHR, GRIN1, NFKBIA, ADCY10, GRIN3A, CAMK4, GHRL, CALM1
mcc04670	Leukocyte transendothelial migration (n=29)	0.628521691	-	ITGB1, NCF1, CTNND1, PIK3R3, PIK3CB, PIK3R1, F11R, CLDN1, ACTB, MYL12A, MYL12B, RAP1B, CLDN22, CLDN20, RAP1A, CLDN23, CTNNA1, RAC1, CD99, ITGA4, RHOA, MAPK12, CLDN4, OCLN, CLDN3, CLDN15, CLDN7, CTNNB1, RAPGEF3
mcc04724	Glutamatergic synapse (n=29)	0.628521691	-	GLS2, SLC1A1, ADCY3, CACNA1A, GRIK1, CACNA1C, PLD1, ADCY8, ADCY7, ADCY6, GRM1, GLS, GNG10, PPP3R1, DLGAP1, SLC38A2, GLUL, MAPK3, KCNJ3, PLA2G4C, GRIN2C, GNG12, GRIN1, GNAO1, PLCB3, GRIN3A, GNB2, GNB1, GNB3
mcc04911	Insulin secretion (n=21)	0.631683543	-	RYR2, CAMK2D, KCNJ11, ABCC8, ADCY3, ATP1B3, CACNA1C, ADCY8, ADCY7, ADCY6, GCK, RIMS2, CREB3, PLCB3, CREB3L4, KCNMA1, KCNN2, CACNA1S, KCNN4, VAMP2, ATF4
mcc05323	Rheumatoid arthritis (n=23)	0.636415104	-	ATP6V1A, ATP6V0B, ATP6V1G1, ATP6V0E1, CCL20, MMP1, CD80, IL18, TCIRG1, TNFSF13B, CTSL, IL23A, CTSK, ATP6V1B2, CCL3, ATP6V1H, LTB, ATP6V1E1, ATP6V0D1, ATP6V0E2, ATP6V1D, ATP6V1C1, ATP6V1B1
mcc04015	Rap1 signaling pathway (n=55)	0.636572508	-	ITGB1, CTNND1, PIK3CB, ACTB, AKT2, AKT3, KDR, PLCE1, RAC1, HRAS, MAP2K3, PDGFRB, F2R, RHOA, TIAM1, PLCB3, ADORA2B, PARD3, TLN2, RAF1, PFN1, RAPGEF3, PFN2, RALA, RALB, SRC, FPR1, PDGFA, ADCY3, PARD6G, PIK3R3, PIK3R1, ADCY8, ADCY7, ADCY6, RAP1B, NRAS, RAP1A, CNR1, PDGFD, PDGFC, MAPK3, FARP2, ANGPT2, EGF, INSR, MAPK12, GRIN1, GNAO1, EFNA2, CTNNB1, CALM1, FGFR4, FGF12, FGFR2
mcc00601	Glycosphingolipid biosynthesis - lacto and neolacto series (n=7)	0.639751292	-	B4GALT2, B3GNT5, B3GALT2, B3GNT4, B3GNT3, B3GNT2, ST3GAL3
mcc04913	Ovarian steroidogenesis (n=12)	0.641884954	-	SCARB1, STAR, INSR, ALOX5, PLA2G4C, ADCY3, PTGS2, ADCY8, ADCY7, ADCY6, BMP6, CYP17A1
mcc00860	Porphyrin and chlorophyll metabolism (n=9)	0.676572267	-	ALAD, UGT1A1, MMAB, UROS, BLVRB, CPOX, EPRS, EARS2, HMOX2
mcc00240	Pyrimidine metabolism (n=25)	0.685035244	-	CDA, POLD4, POLR2B, POLR2D, POLR2E, POLR2G, UPP1, POLR2I, POLR2K, POLR2L, RRM2, PNPT1, ENTPD3, ENTPD4, ENTPD6, CTPS2, NME4, UPB1, DCTD, NT5C3B, UCK2, POLR3C, NME7, POLR3H, DCTPP1
mcc04611	Platelet activation (n=33)	0.695568333	-	ITGB1, SRC, ADCY3, PIK3R3, PIK3CB, PIK3R1, ADCY8, ADCY7, RASGRP1, ADCY6, ACTB, MYL12A, MYL12B, PTGS1, RAP1B, PPP1CC, RAP1A, AKT2, AKT3, PRKG1, MAPK3, LYN, NOS3, F2R, PLA2G4C, GP1BA, RHOA, MAPK12, VAMP8, PLCB3, ORAI1, TLN2, FERMT3
mcc00053	Ascorbate and aldarate metabolism (n=5)	0.695824678	-	ALDH3A2, UGDH, MIOX, UGT1A1, ALDH9A1
mcc05166	HTLV-I infection (n=65)	0.698153233	-	CDKN1A, PIK3CB, ETS2, ELK4, IKBKB, POLB, XPO1, PTTG1, KAT5, AKT2, CDC26, AKT3, TSPO, JAK3, HRAS, APC2, PDGFRB, MAP2K4, CHUK, RRAS2, TNFRSF1A, VDAC3, VDAC2, ANAPC4, VDAC1, TLN2, SLC25A5, SLC25A4, ATF4, ANAPC2, CREM, PDGFA, ADCY3, XIAP, PIK3R3, PIK3R1, IL2RG, ADCY8, ADCY7, ADCY6, ANAPC10, RELB, POLD4, NRAS, PPP3R1, TP53INP1, DVL2, DVL3, BUB3, WNT4, FZD1, EGR2, CREBBP, SMAD4, FZD3, CDKN2C, FZD5, NFATC3, NFKBIA, CDC16, CTNNB1, BAX, RAN, BCL2L1
mcc00562	Inositol phosphate metabolism (n=18)	0.701861899	-	MIOX, MTMR3, IPMK, ITPK1, PIK3CB, PIK3C2A, MTMR7, INPP4A, MINPP1, ALDH6A1, PLCB3, PLCZ1, IMPAD1, PI4KA, PLCE1, CDIPT, PLCD4, PLCD1
mcc05203	Viral carcinogenesis (n=51)	0.704829316	-	YWHAE, CDKN1A, DDX3X, YWHAB, GTF2B, PIK3CB, POLB, CASP8, CREB3L4, CASP3, HIST3H2BB, RAC1, JAK3, CCR4, HRAS, YWHAG, YWHAH, USP7, MRPS18B, TRAF2, YWHAZ, RHOA, CREB3, CCNE2, IRF3, RASA2, TRAF5, VDAC3, IL6ST, ATP6V0D1, IRF9, ATF4, HIST1H2BM, GTF2A2, SRC, PIK3R3, PIK3R1, GTF2E2, NRAS, PMAIP1, MAPK3, LYN, EGR2, CREBBP, GSN, NFKBIA, HNRNPK, CDK2, BAX, GRB2
mcc00290	Valine, leucine and isoleucine biosynthesis (n=2)	0.707139416	-	SDSL, BCAT2
mcc00350	Tyrosine metabolism (n=10)	0.708333023	-	GSTZ1, AOC3, ADH4, ALDH1A3, DDC, HGD, MAOB, ADH1A, ADH7, FAH
mcc04550	Signaling pathways regulating pluripotency of stem cells (n=34)	0.714571915	-	PIK3R3, PIK3CB, PIK3R1, NRAS, AKT2, AKT3, DVL2, DVL3, OTX1, JAK2, HRAS, JAK3, MAPK3, WNT4, FZD1, APC2, PCGF6, ZFHX3, SMAD4, FZD3, FZD5, PCGF5, PCGF2, COMMD3, AXIN2, POU5F1, MAPK12, CTNNB1, GRB2, RAF1, IL6ST, FGFR4, FGFR2, BMPR1A
mcc03440	Homologous recombination (n=8)	0.715670295	-	RAD52, POLD4, RAD51D, BLM, RAD51C, SHFM1, XRCC3, SSBP1
mcc04710	Circadian rhythm (n=8)	0.715670295	-	BHLHE41, PRKAG1, CUL1, RORA, CSNK1D, FBXL3, PRKAB1, RBX1
mcc04925	Aldosterone synthesis and secretion (n=19)	0.72518017	-	SCARB1, CAMK2D, ADCY3, CACNA1C, ADCY8, ADCY7, ADCY6, CREB3, CACNA1I, PLCB3, STAR, CREB3L4, CAMK4, ORAI1, CACNA1S, CAMK1, CALM1, CAMK1G, ATF4

mcc04621	NOD-like receptor signaling pathway (n=13)	0.730756304	-	CHUK, RIPK2, IL18, MEFV, MAPK12, IKBKB, NFKBIA, PSTPIP1, CASP8, SUGT1, BIRC2, NAIP, MAPK3
mcc00380	Tryptophan metabolism (n=11)	0.735714145	-	ALDH3A2, GCDH, HADHA, DDC, AOC1, AANAT, MAOB, CAT, HADH, ALDH9A1, ACMSD
mcc05164	Influenza A (n=43)	0.739348416	-	TMPRSS2, PIK3R3, PIK3CB, PIK3R1, NXT2, ACTB, CASP9, IKBKB, XPO1, TBK1, PABPN1, AKT2, AKT3, TNFSF10, KPNA2, JAK2, PRSS2, IKBKE, MAPK3, AGFG1, IFNAR2, MAP2K3, MAP2K4, CREBBP, DDX58, IFNGR2, MX1, IL18, TICAM1, MAPK12, TNFRSF1A, NFKBIA, CXCL10, DDX39B, IRF3, AXL, CYCS, VDAC1, RAF1, IRF9, MYD88, IFNAR1
mcc05414	Dilated cardiomyopathy (n=21)	0.74590251	-	ITGB1, MYBPC3, RYR2, ITGA4, ITGA3, TPM3, CACNA2D2, ADCY3, CACNA2D4, CACNA1C, ADCY8, ADCY7, ADCY6, ACTB, TTN, CACNB2, ITGA7, ITGAV, ITGB7, CACNA1S, ITGB6
mcc04623	Cytosolic DNA-sensing pathway (n=15)	0.75205517	-	RIPK3, CHUK, DDX58, IL18, IKBKB, NFKBIA, CXCL10, TBK1, IRF3, POLR3C, POLR2E, POLR3H, IKBKE, POLR2K, POLR2L
mcc00590	Arachidonic acid metabolism (n=15)	0.75205517	-	GPX2, PLA2G12B, EPHX2, PLA2G4C, ALOX12, PTGS2, LTC4S, PTGS1, PLA2G16, HPGDS, FAM213B, ALOX5, PLA2G10, LTA4H, CBR3
mcc05217	Basal cell carcinoma (n=13)	0.756582922	-	FZD1, APC2, TCF7L2, FZD3, FZD5, LEF1, TCF7, AXIN2, SUFU, DVL2, DVL3, CTNNB1, WNT4
mcc04660	T cell receptor signaling pathway (n=24)	0.763910807	-	CHUK, PDPK1, NFATC3, PIK3R3, PIK3CB, PIK3R1, RASGRP1, MAPK12, RHOA, IKBKB, NFKBIA, NRAS, PAK1, PPP3R1, AKT2, AKT3, GRB2, PRKCQ, RAF1, HRAS, SOS2, MAPK3, NCK1, PAK4
mcc04713	Circadian entrainment (n=24)	0.763910807	-	RYR2, CAMK2D, KCNJ9, ADCY3, CACNA1C, GNG12, GRIN2C, ADCY8, ADCY7, ADCY6, RYR3, GRIN1, GNAO1, ADCY10, CACNA1I, GNG10, PLCB3, GNB2, GNB1, GNB3, CALM1, PRKG1, MAPK3, KCNJ3
mcc04022	cGMP-PKG signaling pathway (n=38)	0.765354206	-	NPR2, ADCY3, ATP2A2, CACNA1C, ADCY8, ADCY7, ADCY6, PPP1CC, PPP3R1, CREB3L4, AKT2, AKT3, CACNA1S, PRKG1, MAPK3, GTF2I, MEF2A, NOS3, INSR, NFATC3, ATP2B3, ATP1B3, ATP2B1, ADRA2A, RHOA, SLC8A3, CREB3, PLCB3, KCNMA1, VDAC3, PPIF, VDAC2, VDAC1, RAF1, CALM1, SLC25A5, SLC25A4, ATF4
mcc04964	Proximal tubule bicarbonate reclamation (n=6)	0.767141389	-	MDH1, GLS2, ATP1B3, SLC25A10, PCK2, GLS
mcc05204	Chemical carcinogenesis (n=17)	0.770753894	-	GSTM4, UGT1A1, GSTO1, MGST3, GSTP1, ADH1A, CHRNA7, MGST1, MGST2, ADH7, PTGS2, ADH4, ALDH1A3, GSTA4, CYP2A24, GSTA3, SULT2A1
mcc05205	Proteoglycans in cancer (n=50)	0.772051069	-	ITGB1, CDKN1A, CBLC, PIK3CB, ACTB, PPP1CC, CTSL, PLAU, AKT2, CASP3, AKT3, KDR, PLCE1, ITGAV, RAC1, HRAS, PDPK1, RRAS2, ANK2, ANK3, NUDT16L1, ANK1, RHOA, TIAM1, RAF1, SOS2, CD44, CAMK2D, DDX5, SDC4, SRC, PIK3R3, PIK3R1, VTN, PAK1, NRAS, DROSHA, WNT4, MAPK3, FZD1, FZD3, FZD5, FN1, MAPK12, RPS6KB1, RPS6KB2, PDCD4, CTNNB1, SDC1, GRB2
mcc00500	Starch and sucrose metabolism (n=8)	0.780426644	-	UGP2, TREH, GAA, GBE1, ENPP3, PGM1, GCK, HK1
mcc05132	Salmonella infection (n=21)	0.784570142	-	DYNC1I2, ARPC1B, IFNGR2, ARPC1A, WAS, IL18, ARPC5L, ARPC5, ACTB, MAPK12, DYNC1LI1, KLC4, ARPC2, ARPC3, CCL3, RAC1, PKN1, PFN1, MYD88, MAPK3, PFN2
mcc00524	Butirosin and neomycin biosynthesis (n=2)	0.784588717	-	GCK, HK1
mcc00471	D-Glutamine and D-glutamate metabolism (n=2)	0.784588717	-	GLS2, GLS
mcc00260	Glycine, serine and threonine metabolism (n=11)	0.788826249	-	AOC3, SRR, MAOB, PSAT1, SHMT2, CHDH, PGAM1, AMT, SDSL, DLD, GNMT
mcc00270	Cysteine and methionine metabolism (n=10)	0.794139051	-	LDHA, MDH1, MAT2A, TST, AMD1, SMS, MRI1, MAT2B, SDSL, LDHAL6B
mcc00040	Pentose and glucuronate interconversions (n=6)	0.800665099	-	UGDH, UGP2, UGT1A1, DCXR, CRYL1, AKR1B1
mcc04140	Regulation of autophagy (n=6)	0.800665099	-	GABARAPL2, GABARAPL1, ATG10, ULK1, ATG14, ATG5
mcc00670	One carbon pool by folate (n=4)	0.80066954	-	DHFR, SHMT2, AMT, GART
mcc05410	Hypertrophic cardiomyopathy (HCM) (n=19)	0.805786835	-	ITGB1, MYBPC3, RYR2, ITGA4, ITGA3, TPM3, PRKAG1, CACNA2D2, CACNA2D4, CACNA1C, PRKAB1, ACTB, TTN, CACNB2, ITGA7, ITGAV, ITGB7, CACNA1S, ITGB6
mcc04261	Adrenergic signaling in cardiomyocytes (n=33)	0.807759841	-	RYR2, CAMK2D, CREM, ADCY3, CACNA1C, ADCY8, ADCY7, ADCY6, PPP2CB, PPP1CC, CREB3L4, AKT2, AKT3, CACNA1S, MAPK3, TPM3, PPP2R5B, PPP2R5A, CACNA2D2, ATP2B3, ATP1B3, PPP2R5D, PPP2R3A, ATP2B1, CACNA2D4, MAPK12, CREB3, CACNB2, PLCB3, KCNQ1, CALM1, RAPGEF3, ATF4
mcc04310	Wnt signaling pathway (n=32)	0.808154497	-	CAMK2D, CTBP1, TCF7, LEF1, CUL1, PRICKLE1, PORCN, PPP3R1, RUVBL1, DVL2, DVL3, GPC4, RAC1, WNT4, FZD1, APC2, TCF7L2, CREBBP, SMAD4, FZD3, FZD5, CSNK2A1, SIAH1, NFATC3, AXIN2, DKK1, RHOA, RBX1, PLCB3, VANGL2, CSNK2B, CTNNB1
mcc00532	Glycosaminoglycan biosynthesis - chondroitin sulfate / dermatan sulfate (n=5)	0.815770101	-	CHPF, B3GAT3, XYLT2, XYLT1, B3GALT6
mcc04750	Inflammatory mediator regulation of TRP channels (n=23)	0.815835001	-	MAP2K3, NTRK1, CAMK2D, SRC, PLA2G4C, PRKCD, HTR2B, TRPV2, PIK3R3, ADCY3, ALOX12, PIK3CB, PIK3R1, ADCY8, ADCY7, ADCY6, MAPK12, PPP1CC, PLCB3, P2RY2, TRPV4, PRKCQ, CALM1
mcc04744	Phototransduction (n=7)	0.818071209	-	SLC24A1, GNAT2, GNB1, PDE6A, GUCA1C, CALM1, RGS9
mcc04723	Retrograde endocannabinoid signaling (n=23)	0.831066098	-	KCNJ9, CACNA1B, ADCY3, CACNA1A, ABHD6, CACNA1C, GNG12, PTGS2, ADCY8, ADCY7, ADCY6, MAPK12, GRM1, GNAO1, GNG10, PLCB3, CNR1, GNB2, GNB1, GNB3, CACNA1S, MAPK3, KCNJ3
mcc04070	Phosphatidylinositol signaling system (n=23)	0.831066098	-	CDS1, DGKG, TMEM55B, MTMR3, DGKA, IPMK, ITPK1, PIK3R3, PIK3CB, PIK3R1, PIK3C2A, MTMR7, INPP4A, PLCB3, PLCZ1, IMPAD1, PI4KA, PLCE1, CDIPT, CALM1, PLCD4, IP6K3, PLCD1

mcc04630	Jak-STAT signaling pathway (n=32)	0.834539592	-	CSF3, CSF3R, MPL, PIK3R3, PIK3CB, PIK3R1, IL2RG, AKT2, AKT3, PIM1, LEPR, JAK2, IL12RB1, JAK3, IL12RB2, SOCS5, IFNAR2, PIAS4, CREBBP, TSLP, IFNGR2, PIAS1, IL22RA1, IL23A, IFNK, GRB2, IL6ST, SOS2, STAM2, IRF9, BCL2L1, IFNAR1
mcc05162	Measles (n=30)	0.836193354	-	PIK3R3, PIK3CB, PIK3R1, RCHY1, IL2RG, TBK1, AKT2, AKT3, TNFSF10, JAK2, JAK3, IKBKE, IFNAR2, CHUK, CSNK2A1, DDX58, IFNGR2, MX1, NFKBIA, CCNE2, IRF3, CDK2, CSNK2B, EIF3H, PRKCQ, CD46, IRF9, MYD88, IFNAR1, TP73
mcc00450	Selenocompound metabolism (n=4)	0.836541482	-	SCLY, SEPHS1, SEPHS2, PAPSS1
mcc00511	Other glycan degradation (n=4)	0.836541482	-	GLB1, FUCA1, NEU1, HEXDC
mcc00514	Other types of O-glycan biosynthesis (n=5)	0.845924952	-	LFNG, B4GALT2, GXYLT1, RFNG, ST3GAL3
mcc00592	alpha-Linolenic acid metabolism (n=5)	0.845924952	-	PLA2G16, PLA2G12B, PLA2G4C, PLA2G10, ACOX3
mcc04728	Dopaminergic synapse (n=30)	0.848620196	-	CAMK2D, DDC, MAOB, CACNA1B, CACNA1A, CACNA1C, PPP2CB, PPP1CC, GNG10, CREB3L4, AKT2, AKT3, SLC18A2, KCNJ3, SCN1A, KCNJ9, PPP2R5B, PPP2R5A, PPP2R5D, PPP2R3A, GNG12, MAPK12, GNAO1, CREB3, PLCB3, GNB2, GNB1, GNB3, CALM1, ATF4
mcc05031	Amphetamine addiction (n=15)	0.851448035	-	CAMK2D, DDC, MAOB, CACNA1C, GRIN2C, GRIN1, PPP1CC, CREB3, PPP3R1, GRIN3A, CREB3L4, CAMK4, CALM1, SLC18A2, ATF4
mcc04390	Hippo signaling pathway (n=35)	0.855607357	-	CRB2, YWHAE, CRB1, YWHAB, TCF7, LEF1, PARD6G, ACTB, STK3, PPP2CB, PPP1CC, RASSF6, DVL2, CTNNA1, DVL3, YWHAG, WNT4, YWHAH, FZD1, APC2, TCF7L2, SMAD4, FZD3, FZD5, CSNK1D, AXIN2, YWHAZ, BMP6, PARD3, CTNNB1, NF2, BMPR1A, LLGL2, TP73
mcc00250	Alanine, aspartate and glutamate metabolism (n=8)	0.855964407	-	GLS2, GFPT1, ADSSL1, ASNS, ASL, ADSS, GLUL, GLS
mcc05218	Melanoma (n=16)	0.863302234	-	PDGFRB, CDKN1A, EGF, PIK3R3, PDGFA, PIK3CB, PIK3R1, NRAS, AKT2, PDGFD, PDGFC, AKT3, RAF1, HRAS, FGF12, MAPK3
mcc05032	Morphine addiction (n=20)	0.865355849	-	KCNJ9, PDE1B, PDE4D, CACNA1B, ADCY3, CACNA1A, GNG12, ADCY8, ADCY7, ADCY6, GNAO1, GNG10, GNB2, GRK6, GNB1, GNB3, PDE7B, PDE8B, PDE7A, KCNJ3
mcc00360	Phenylalanine metabolism (n=4)	0.866695082	-	AOC3, ALDH1A3, DDC, MAOB
mcc00603	Glycosphingolipid biosynthesis - globo series (n=3)	0.86669917	-	NAGA, A4GALT, GLA
mcc04514	Cell adhesion molecules (CAMs) (n=32)	0.869049921	-	ITGB1, CD274, CNTNAP2, CNTNAP1, SDC4, CD80, SDC3, ICAM2, NRXN3, LRRC4, ICAM3, F11R, CLDN1, CLDN22, CLDN20, ALCAM, CDH2, CLDN23, ITGB7, NCAM1, ITGAV, CD58, MPZL1, CD99, ITGA4, CLDN4, OCLN, CLDN3, CLDN15, CLDN7, SDC1, CNTN2
mcc04340	Hedgehog signaling pathway (n=5)	0.871799222	-	CSNK1G3, SUFU, IHH, CSNK1D, LRP2
mcc04360	Axon guidance (n=29)	0.872652484	-	ITGB1, SEMA5B, SEMA3B, LRRC4, NTN1, NTN3, EFNB2, EFNB1, PAK1, NRAS, PPP3R1, CFL1, SLIT1, SLIT3, PLXNC1, RAC1, HRAS, SRGAP1, PLXNA4, MAPK3, PAK4, NFATC3, SEMA4G, RHOA, EFNA2, CDK5, RASA1, PLXNB3, PLXNB1
mcc00983	Drug metabolism - other enzymes (n=8)	0.875883402	-	CDA, UCK2, UGT1A1, TPMT, GMPS, HPRT1, UPP1, UPB1
mcc04960	Aldosterone-regulated sodium reabsorption (n=8)	0.875883402	-	PDPK1, INSR, PIK3R3, ATP1B3, SFN, PIK3CB, PIK3R1, MAPK3
mcc05152	Tuberculosis (n=42)	0.880057518	-	CAMK2D, CEBPB, RAB5B, RAB5C, SRC, CEBPG, TCIRG1, CTSS, HSPD1, CASP9, PPP3R1, CASP8, CASP10, CASP3, AKT2, AKT3, LAMP2, ATP6V1H, FADD, BID, JAK2, CTSD, MAPK3, CREBBP, ATP6V0B, SPHK2, RIPK2, IFNGR2, IL18, RFXANK, TIRAP, RHOA, MAPK12, TNFRSF1A, IL23A, BAX, CYCS, RAF1, PLA2R1, ATP6V0D1, CALM1, MYD88
mcc04260	Cardiac muscle contraction (n=19)	0.880829853	-	COX8A, RYR2, COX7B, TPM3, COX4I1, CACNA2D2, ATP1B3, CACNA2D4, CACNA1C, UQCR, COX5A, UQCRH, COX6B1, CACNB2, COX7A2L, UQCRQ, UQCRC1, UQCRC2, CACNA1S
mcc04380	Osteoclast differentiation (n=29)	0.883069378	-	NCF1, FHL2, PIK3R3, PIK3CB, PIK3R1, RELB, IKBKB, PPP3R1, AKT2, CTSK, AKT3, BLNK, RAC1, MAPK3, IFNAR2, JUND, CHUK, IFNGR2, TRAF2, GAB2, MAPK12, TNFRSF1A, NFKBIA, CAMK4, GRB2, SQSTM1, NOX1, IRF9, IFNAR1
mcc05219	Bladder cancer (n=9)	0.884895532	-	CDKN1A, NRAS, EGF, SRC, MMP1, DAPK3, RAF1, HRAS, MAPK3
mcc04270	Vascular smooth muscle contraction (n=26)	0.887773697	-	NPR2, ADCY3, CACNA1C, ADCY8, ADCY7, ADCY6, PPP1CC, CALD1, CACNA1S, PRKG1, MAPK3, PLA2G12B, AVPR1B, PRKCD, PLA2G4C, RHOA, ACTA2, PLCB3, MYL6, ADORA2B, PLA2G10, KCNMA1, PRKCQ, RAF1, CALM1
mcc04978	Mineral absorption (n=10)	0.893273103	-	MT2A, SLC31A1, FTH1, CYBRD1, ATP1B3, SLC30A1, MT1X, SLC26A3, HMOX2, FTL
mcc03430	Mismatch repair (n=5)	0.893832449	-	RFC5, MSH6, POLD4, SSBP1, MLH3
mcc04918	Thyroid hormone synthesis (n=15)	0.89435173	-	GPX2, HSPA5, ADCY3, ATP1B3, LRP2, TTF1, ADCY8, ADCY7, ADCY6, TSHR, HSP90B1, CREB3, PLCB3, CREB3L4, ATF4
mcc00512	Mucin type O-Glycan biosynthesis (n=6)	0.898111345	-	GALNT7, C1GALT1C1, GALNT3, GCNT1, C1GALT1, GCNT3
mcc04961	Endocrine and other factor-regulated calcium reabsorption (n=9)	0.900596971	-	DNM3, PLCB3, CALB1, CLTA, ATP1B3, ADCY6, AP2M1, RAB11A, DNM1
mcc05020	Prion diseases (n=7)	0.903335237	-	PRNP, HSPA5, BAX, NCAM1, C8A, MAPK3, SOD1
mcc04973	Carbohydrate digestion and absorption (n=8)	0.908884668	-	AKT2, AKT3, PIK3R3, ATP1B3, PIK3CB, PIK3R1, SLC37A4, HK1
mcc04971	Gastric acid secretion (n=16)	0.913180196	-	CAMK2D, KCNK10, ADCY3, ATP1B3, SSTR2, ADCY8, ADCY7, ADCY6, ATP4B, ATP4A, PLCB3, CCKBR, KCNQ1, CALM1, CFTR, KCNJ2
mcc00310	Lysine degradation (n=11)	0.913755619	-	ALDH3A2, KMT2E, KMT2D, GCDH, HADHA, EHMT2, KMT2C, SETDB2, DLST, HADH, ALDH9A1
mcc03410	Base excision repair (n=7)	0.9182955	-	POLB, SMUG1, POLD4, NTHL1, APEX2, LIG3, HMGB1

mcc04730	Long-term depression (n=12)	0.919725497	-	GNAO1, LYN, PPP2CB, NRAS, PLCB3, PLA2G4C, CACNA1A, RAF1, HRAS, PRKG1, GRM1, MAPK3
mcc05168	Herpes simplex infection (n=45)	0.921928196	-	SRSF1, CUL1, IKBKB, PPP1CC, CASP8, TBK1, CASP3, C1QBP, TNFRSF14, FADD, TAF9B, JAK2, IKBKE, HMGN1, GTF2I, IFNAR2, CREBBP, USP7, CHUK, CSNK2A1, DDX58, IFNGR2, MED8, TRAF2, HCFC2, TICAM1, GLTSCR2, TNFRSF1A, NFKBIA, TAF6L, CDC34, HNRNPK, UBE2R2, IRF3, TRAF5, CDK2, CSNK2B, SRSF3, CYCS, SRSF5, TAF5, TAF4, IRF9, MYD88, IFNAR1
mcc03420	Nucleotide excision repair (n=9)	0.926615288	-	CUL4A, RFC5, POLD4, CDK7, ERCC1, CETN2, MNAT1, RAD23B, RBX1
mcc01040	Biosynthesis of unsaturated fatty acids (n=4)	0.929716584	-	PECR, HADHA, ELOVL5, ACOX3
mcc03460	Fanconi anemia pathway (n=10)	0.93089509	-	FANCI, BLM, RAD51C, FANCL, EME2, ERCC1, FANCC, POLH, FAN1, FANCF
mcc00830	Retinol metabolism (n=11)	0.935084574	-	SDR16C5, ADH4, UGT1A1, DGAT1, ALDH1A2, RDH10, ADH1A, ALDH1A1, HSD17B6, ADH7, BCO1
mcc04922	Glucagon signaling pathway (n=23)	0.936135778	-	PFKFB1, CREBBP, CAMK2D, PDHA1, PGAM1, PRKAG1, PDHB, PRKAB1, CPT1B, GCK, CREB3, PLCB3, LDHA, PPP3R1, PPP4C, PHKG1, CREB3L4, AKT2, AKT3, CALM1, PCK2, LDHAL6B, ATF4
mcc05034	Alcoholism (n=40)	0.937265781	-	HIST1H2BM, DDC, SHC2, MAOB, SHC1, HIST2H2AB, HIST2H2AC, PPP1CC, GNG10, NRAS, CREB3L4, HIST3H2BB, HRAS, SLC18A2, HIST1H2AC, MAPK3, NTRK2, H2AFY, H2AFZ, BDNF, HIST1H2AJ, GRIN2C, GNG12, GRIN1, GNAO1, CREB3, GRIN3A, H2AFY2, ADORA2B, PKIA, CAMK4, GNB2, GNB1, GNB3, GRB2, HIST2H3D, RAF1, CALM1, SOS2, ATF4
mcc00760	Nicotinate and nicotinamide metabolism (n=6)	0.941075874	-	NT5C3B, NMRK2, NMRK1, NAPRT, ENPP3, NUDT13
mcc04950	Maturity onset diabetes of the young (n=4)	0.943681856	-	HNF4A, PAX4, FOXA3, GCK
mcc04612	Antigen processing and presentation (n=14)	0.946637243	-	PDIA3, HSPA4, RFXANK, KIR3DL2, IFI30, CTSS, TAPBP, CTSL, PSME3, PSME1, PSME2, B2M, CTSB, LGMN
mcc05142	Chagas disease (American trypanosomiasis) (n=21)	0.946819664	-	MAP2K4, CHUK, IFNGR2, PIK3R3, PIK3CB, PIK3R1, TICAM1, MAPK12, TNFRSF1A, IKBKB, NFKBIA, GNAO1, PPP2CB, PLCB3, CASP8, AKT2, AKT3, CCL3, FADD, MYD88, MAPK3
mcc04970	Salivary secretion (n=16)	0.94693284	-	ATP2B3, ADCY3, ATP1B3, ATP2B1, BEST2, ADCY8, ADCY7, ADCY6, RYR3, CST3, PLCB3, KCNMA1, CALM1, KCNN4, PRKG1, VAMP2
mcc04640	Hematopoietic cell lineage (n=15)	0.956316233	-	CSF3, CSF3R, ITGA4, MME, TFRC, ITGA3, FLT3LG, DNTT, GP1BA, CD1D, CD9, CD59, CD37, CD33, CD44
mcc05033	Nicotine addiction (n=7)	0.959724466	-	CHRNB2, GRIN3A, CHRNA7, CACNA1B, CACNA1A, GRIN2C, GRIN1
mcc05146	Amoebiasis (n=21)	0.962540955	-	SERPINB10, SERPINB1, RAB5B, ARG2, SERPINB2, RAB5C, LAMA2, LAMB2, LAMA4, LAMA3, FN1, PIK3R3, LAMC2, LAMB1, PIK3CB, PIK3R1, C8A, PLCB3, COL4A2, COL4A1, CASP3
mcc04350	TGF-beta signaling pathway (n=16)	0.964163455	-	TGIF1, SMAD4, CREBBP, TGIF2, AMHR2, CUL1, SMAD6, BMP6, RHOA, RBX1, PPP2CB, RPS6KB1, RPS6KB2, E2F5, MAPK3, BMPR1A
mcc00130	Ubiquinone and other terpenoid-quinone biosynthesis (n=2)	0.9659256	-	NQO1, COQ6
mcc00591	Linoleic acid metabolism (n=4)	0.971603886	-	PLA2G16, PLA2G12B, PLA2G4C, PLA2G10
mcc05133	Pertussis (n=14)	0.973557677	-	ITGB1, C1R, TICAM1, TIRAP, MAPK12, RHOA, CASP7, IRF3, IL23A, CASP3, CFL1, CALM1, MYD88, MAPK3
mcc00910	Nitrogen metabolism (n=3)	0.977183652	-	CA5B, GLUL, CA14
mcc00531	Glycosaminoglycan degradation (n=3)	0.977183652	-	GLB1, ARSB, SGSH
mcc04060	Cytokine-cytokine receptor interaction (n=42)	0.980040064	-	CSF3, CSF3R, AMHR2, CXCR5, MPL, CXCR6, IL2RG, EDA2R, CXCL16, TNFSF13B, CCL7, LEPR, CCL3, TNFSF10, TNFRSF17, TNFRSF14, CCR7, IL12RB1, CCR4, IL12RB2, CCL25, IFNAR2, TNFRSF12A, TSLP, CCL20, IFNGR2, TNFRSF19, IL18, TNFRSF1B, IL17RB, TNFRSF1A, EDAR, IL22RA1, CXCL10, IL23A, CLCF1, IFNK, LTB, IL6ST, CCL28, BMPR1A, IFNAR1
mcc00430	Taurine and hypotaurine metabolism (n=2)	0.981579713	-	GADL1, CSAD
mcc04650	Natural killer cell mediated cytotoxicity (n=21)	0.98228223	-	IFNAR2, SHC2, SHC1, IFNGR2, ICAM2, PIK3R3, PIK3CB, PIK3R1, NRAS, PAK1, PPP3R1, CASP3, TNFSF10, GRB2, RAC1, RAF1, BID, HRAS, SOS2, MAPK3, IFNAR1
mcc04924	Renin secretion (n=11)	0.986588442	-	PPP3R1, PLCB3, PDE1B, KCNMA1, ORAI1, CACNA1C, CACNA1S, CALM1, ADCY6, KCNJ2, CTSB
mcc05340	Primary immunodeficiency (n=5)	0.986836431	-	DCLRE1C, BLNK, ORAI1, RFXANK, IL2RG
mcc04614	Renin-angiotensin system (n=3)	0.989690216	-	ACE2, MME, ATP6AP2
mcc00604	Glycosphingolipid biosynthesis - ganglio series (n=2)	0.990044298	-	GLB1, ST3GAL5
mcc04742	Taste transduction (n=6)	0.990400604	-	TAS2R40, GNB3, CACNA1A, TAS1R1, ADCY8, ADCY6
mcc04610	Complement and coagulation cascades (n=11)	0.990480816	-	PROC, SERPIND1, F10, PLAU, C1R, F2R, CD59, F2, CD46, C8A, F5
mcc04672	Intestinal immune network for IgA production (n=7)	0.991477895	-	CCL25, ITGA4, CD80, TNFRSF17, ITGB7, CCL28, TNFSF13B
mcc00140	Steroid hormone biosynthesis (n=6)	0.993761884	-	UGT1A1, SRD5A3, HSD17B3, HSD17B6, HSD17B8, CYP17A1

mcc03010	Ribosome (n=62)	0.993954356	-	RPL4, MRPS16, RPL3, MRPS14, RPL34, RPLP0, RPS17L, MRPL34, RPL10A, MRPL33, RPL7, RPS15, RPL7A, MRPL3, RPS14, RPS19, RPL35, RPS11, RPL39, RPS13, RPS12, RPS9, SRRM5, RPL21, RPS7, RPS8, RPL23, RPS5, MRPS18A, RPL13A, MRPS2, MRPS21, MRPS7, MRPS18C, RPL37A, RPL27, RPL26, RPL28, RPS4Y2, MRPL18, RPL12, RPS27L, MRPL15, MRPL13, MRPL10, MRPL20, RPS3, RPL15, RPL17, MRPL28, MRPL24, MRPL22, RPS26, RPS25, RPS29, RPL27A, RPL22L1, RPS20, FAU, RSL24D1, RPS21, RPS23
mcc05143	African trypanosomiasis (n=4)	0.994755871	-	PLCB3, LAMA4, IL18, MYD88
mcc05144	Malaria (n=6)	0.995979603	-	CSF3, CD81, IL18, SDC1, THBS2, MYD88
mcc05202	Transcriptional misregulation in cancer (n=30)	0.996732371	-	CEBPA, CDKN1A, CEBPB, DDX5, HPGD, PDGFA, TMPRSS2, AFF1, ELK4, RXRA, PLAU, TSPAN7, ITGB7, PTCRA, NTRK1, CDKN2C, JUP, FUS, TFE3, PBX3, COMMD3, HMGA2, RUNX1, ETV6, CDK9, NCOR1, EWSR1, DDIT3, HIST2H3D, BCL2L1
mcc05140	Leishmaniasis (n=10)	0.99687259	-	ITGB1, NFKBIA, ITGA4, NCF1, IFNGR2, JAK2, PTGS2, MYD88, MAPK12, MAPK3
mcc05321	Inflammatory bowel disease (IBD) (n=8)	0.999049731	-	IL23A, IFNGR2, IL18, RORA, IL2RG, IL12RB1, FOXP3, IL12RB2
mcc03030	DNA replication (n=4)	0.999115303	-	RFC5, POLD4, SSBP1, MCM6
mcc05416	Viral myocarditis (n=10)	0.999209734	-	CASP9, CXADR, CASP8, CASP3, CD80, CYCS, RAC1, BID, ACTB, EIF4G2
mcc05322	Systemic lupus erythematosus (n=16)	0.999990632	-	HIST1H2BM, SSB, H2AFY, H2AFZ, C1R, CD80, HIST1H2AJ, HIST2H2AB, C8A, HIST2H2AC, H2AFY2, HIST3H2BB, SNRPD3, HIST2H3D, HIST1H2AC, SNRPB
mcc04080	Neuroactive ligand-receptor interaction (n=42)	0.99999336	-	THRA, CHRNA7, MLNR, RXFP4, HTR2B, FPR1, GRIK1, HTR4, LTB4R, GRM1, CHRND, GLRA1, CHRNG, CNR1, P2RY2, CHRNE, LEPR, TSPO, PRSS2, CHRNB2, AVPR1B, GNRHR2, GPR35, GRID1, F2R, SCTR, TACR2, F2, GRIN2C, SSTR2, SSTR3, ADRA2A, TSHR, GRIN1, P2RX7, GRIN3A, CCKBR, ADORA2B, LPAR6, PARD3, F2RL2
mcc05206	MicroRNAs in cancer (n=39)	0.999998541	-	CDKN1A, NOTCH1, GLS2, SHC1, NOTCH4, PDGFA, PTGS2, GLS, IKBKB, NRAS, PLAU, CASP3, PIM1, HRAS, MCL1, PAK4, APC2, PDGFRB, CREBBP, ST14, FZD3, ABCC1, UBE2I, COMMD3, HMGA2, CDC25C, RHOA, SERPINB5, ZEB2, ZEB1, HNRNPK, CCNE2, CCNG1, DDIT4, PDCD4, GRB2, RAF1, SOS2, CD44
mcc05150	Staphylococcus aureus infection (n=2)	0.999999487	-	C1R, FPR1
mcc04940	Type I diabetes mellitus (n=2)	0.999999624	-	CD80, HSPD1
mcc05320	Autoimmune thyroid disease (n=2)	0.999999983	-	CD80, TSHR
mcc04740	Olfactory transduction (n=10)	1	-	OR2D3, OR51G2, CAMK2D, OR2H2, OR2W1, OR10G3, OR4D11, ADCY3, CALM1, PRKG1
