# Supplementary material for: Identification, Characterization, and Transcriptional Reprogramming of Epithelial Stem Cells and Intestinal Enteroids in Simian Immunodeficiency Virus Infected Rhesus Macaques
Source: Front Immunol. 2021 Nov 23;12:769990. doi: 10.3389/fimmu.2021.769990 (PMC8650114; doi:10.3389/fimmu.2021.769990)
Supplement: Supplementary file 11 [file Table_5.pdf]

Supplementary Table 5: List of selected upregulated genes involved in different metabolic pathway of SIV infected enteroids compared to uninfected enteroids

Pathway	Gene	Protein	Log2 fold chang	Biological function
Glycolysis	PFKM	Phosphofructokinase, Muscle	4.1	Conversion of fructose-6-phosphate to fructose-1,6-bisphosphate
	GCK	Glucokinase	3	Phosphorylates glucose to glucose-6-phosphate
Pyruvate metabolism	LDHAL6B	Lactate Dehydrogenase A Like 6B	9.3	Synthesizes (S)-lactate from pyruvate
Fatty acid metabolism	ELOVL5	Elongation of very long chain fatty acids protein 5	1.4	Long-chain fatty acids elongation cycle
	ACSBG 1	Long-chain-fatty-acid--CoA ligase ACSBG1	5.5	Conversion of fatty acids to their active form acyl-CoAs
	ACSBG 2	Long-chain-fatty-acid--CoA ligase ACSBG2	7.4	Conversion of fatty acids to their active form acyl-CoAs
	CPT1B	Carnitine O-palmitoyltransferase 1, muscle isoform	5.2	Fatty acid beta-oxidation
