# Supplementary material for: Identification, Characterization, and Transcriptional Reprogramming of Epithelial Stem Cells and Intestinal Enteroids in Simian Immunodeficiency Virus Infected Rhesus Macaques
Source: Front Immunol. 2021 Nov 23;12:769990. doi: 10.3389/fimmu.2021.769990 (PMC8650114; doi:10.3389/fimmu.2021.769990)
Supplement: Supplementary file 12 [file Table_6.pdf]

Supplementary Table 6: List of selected downregulated genes involved in different metabolic pathway of SIV infected enteroids compared to uninfected enteroids

Pathway	Gene	Protein	Log2 fold change	Biological function
Glycolysis	PDHA1	Pyruvate Dehydrogenase E1 Subunit Alpha 1	1.5	Converts pyruvate to acetyl-coA
	PDHB	Pyruvate Dehydrogenase E1 Subunit Beta	2.6	Converts pyruvate to acetyl-coA
	PGAM1	Phosphoglycerate Mutase 1	2	Conversion of 3-phosphoglycerate to 2-phosphoglycerate
	ENO2	Enolase 2	3.3	Dehydratation of 2-phosphoglycerate to phosphoenolpyruvate
	HK1	Hexokinase 1	2.1	Catalyzes the phosphorylation of various hexoses
	PFKL	Phosphofructokinase, Liver Type	2.3	Conversion of fructose-6-phosphate to fructose-1,6-bisphosphate
	PGK1	Phosphoglycerate Kinase 1	3.8	Transfer of a phosphate group from 1,3-bisphosphoglycerate (1,3-BPG) to ADP
	ALDO A	Aldolase A	1.6	Conversion of fructose 1-6-diphosphate to glyceraldehyde 3-phosphate and dihydroxy-acetone phosphate
	ALDO B	Aldolase B	3.3	Conversion of fructose 1-6-diphosphate to glyceraldehyde 3-phosphate and dihydroxy-acetone phosphate
	ALDO C	Aldolase C	3.2	Conversion of fructose 1-6-diphosphate to glyceraldehyde 3-phosphate and dihydroxy-acetone phosphate
	PGM1	Phosphoglucomutase 1	1.5	Transfer of phosphate from position 1 to 6 on glucose
Pyruvate metabolism	ACSS 1	Acyl-CoA Synthetase Short Chain Family Member 1	1.9	Synthesis of acetyl-CoA
	ACSS 2	Acyl-CoA Synthetase Short Chain Family Member 2	2.4	Synthesis of acetyl-CoA
	PDHA1	Pyruvate Dehydrogenase E1 Subunit Alpha 1	1.5	Conversion of pyruvate to acetyl-CoA
	PDHB	Pyruvate Dehydrogenase E1 Subunit Beta	2.6	Conversion of pyruvate to acetyl-CoA
	LDH A	Lactate Dehydrogenase A	2.6	Synthesizes (S)-lactate from pyruvate
	LDH D	Lactate Dehydrogenase D	1.9	Synthesizes (S)-lactate from pyruvate
	DLAT	Dihydrolipoamide S-Acetyltransferase	1.5	Conversion of pyruvate to acetyl-CoA
	PCK2	Phosphoenolpyruvate Carboxykinase	2.3	Converts oxaloacetate into phosphoenolpyruvate and carbon dioxide
Fatty acid metabolism	PECR	Peroxisomal trans-2-enoyl-CoA reductase	2.1	Chain elongation of fatty acids
	ACAA2	3-ketoacyl-CoA thiolase, mitocondrial	1.4	Production of energy from fats
	OXSM	3-oxoacyl-[acyl-carrier-protein] synthase, mitocondrial	4.2	Biosynthesis of lipoic acid as well as longer chain fatty acids
	ACSL4	Long-chain-fatty-acid--CoA ligase 4	1.9	Conversion of long-chain fatty acids to their active form acyl-CoA
	MCAT	Malonyl-CoA-acyl carrier protein transacylase,	2.3	Transfer of a malonyl moiety from malonyl-CoA
	ACADSB	Short/branched chain specific acyl-CoA dehydrogenase, mitochondrial	3.1	Formation of trans-2-enoyl-CoA
	HADH A	Trifunctional enzyme subunit alpha, mitochondrial	2	Mitochondrial beta-oxidation pathway
	HADH B	Trifunctional enzyme subunit alpha, mitochondrial	3.3	Mitochondrial beta-oxidation pathway
	ACOX3	Peroxisomal acyl-coenzyme A oxidase 3	2.6	Oxidizes the CoA-esters of 2-methyl-branched fatty acids
	ACADM	Medium-chain specific acyl-CoA dehydrogenase, mitocondrial	4.3	Mitochondrial fatty acid beta-oxidation
	HADH	Hydroxyacyl-CoA Dehydrogenase	2.6	Beta-oxidation cycle for medium and short-chain 3-hydroxy fatty acyl-CoAs
	ACADS	Short-chain specific acyl-	2.2	Mitochondrial fatty acid beta-oxidation
Ketone bodies metabolism	BDH1	D-beta-hydroxybutyrate dehydrogenase	2	Conversion of 3-hydroxybutanoate to acetoacetate and NADH
	BDH2	3-hydroxybutyrate dehydrogenase type 2	3.5	Conversion of 3-hydroxybutanoate to acetoacetate and NADH
	HMGCS1	Hydroxymethylglutaryl-CoA synthase, cytoplasmic Succinyl-CoA:3-ketoacid coenzyme A transferase 1	3.4	Condensation of acetyl-CoA with acetoacetyl-CoA to form HMG-CoA
	OXCT1	3-Oxoacid CoA-Transferase 1	3.9	Key enzyme for ketone body catabolism
