# Supplementary material for: Identification, Characterization, and Transcriptional Reprogramming of Epithelial Stem Cells and Intestinal Enteroids in Simian Immunodeficiency Virus Infected Rhesus Macaques
Source: Front Immunol. 2021 Nov 23;12:769990. doi: 10.3389/fimmu.2021.769990 (PMC8650114; doi:10.3389/fimmu.2021.769990)
Supplement: Supplementary file 13 [file Table_7.pdf]

Supplementary Table 7: The 19 significantly enriched GO terms in the Molecular Function Category among upregulated DEGs

Category	GO:number	Term	Count	PValue	FDR	Genes
GOTERM_MF_DIRECT	GO:0005509	calcium ion binding	97	6.21E-05	0.045704	DGKG, RASEF, CBLC, GUCA1C, PKD2L2, CELSR3, CLGN, RPH3A, HPGDS, CDH2, GNPTAB, CAPN3, SVEP1, HMCN1, HPCAL1, SYTL3, PLA2G10, MASP2, FBN2, FBN3, TTYH1, NOTCH4, CABP4, HPCA, CACNA1B, PROZ, LTBP3, CACNA1E, NID2, RASGRP1, FBLN2, FBLN5, RASGRP4, SCUBE3, PPP3R1, OTOF, MCC, LRP1B, PROC, CABYR, FAT2, FBN1, RYR2, SNED1, MCTP1, DOC2A, STAB2, EFCAB6, PKD2, EFCAB7, EFCAB9, MMP21, MMP20, MMP24, CALB1, PLCZ1, ANXA9, OIT3, HEG1, F2, C2CD4D, CDH10, MMP19, CDH13, ITGB1BP2, MCTP2, MATN2, CRB2, CRB1, SLC24A1, SLC24A3, DNAH7, C1R, LRP2, THBS2, NCALD, DLL3, DUOX1, SRR, SLIT1, SLIT3, AOC3, PCDH9, F10, EGF, KCNIP2, KCNIP4, SUSD1, SYT16, PKDREJ, PPP2R3A, GRIN1, DLK2, CAPN12, PLCD4, SLC24A5, PLCD1
GOTERM_MF_DIRECT	GO:0005262	calcium channel activity	13	1.21E-04	-	SLC24A1, SLC24A3, TRPC3, TRPV2, PKDREJ, PKD1L3, PKD2L2, PKD2, GRIN1, TRPM1, GRIN3A, TRPV4, SLC24A5
GOTERM_MF_DIRECT	GO:0004252	serine-type endopeptidase activity	29	0.001096	-	TMPRSS9, TMPRSS7, KLK1, TMPRSS6, C1R, TMPRSS3, PRSS48, PROZ, PRSS27, HTRA2, PRSS50, PRSS2, RHBDF1, F10, MST1, OVCH2, PRSS37, PRSS36, F2, KLK10, PRSS53, PRSS54, IMMP1L, PREPL, PROC, CTRL, MMP19, MASP2, LPA
GOTERM_MF_DIRECT	GO:0008270	zinc ion binding	140	0.001917	-	ZFAND4, EHMT2, GTF2B, MPI, CBLC, RORA, ZDBF2, MT2A, RNF115, FNTB, PIAS4, LMO1, MORC4, KDM2B, LIMCH1, RC3H1, LIG3, RC3H2, PIAS1, TRAIP, RNF123, TRIM17, KAT6B, TRIM16, KMT2E, ZNF276, KMT2D, CPB1, KMT2C, DTX1, BAZ2B, AEBP1, ADH7, MEFV, DTX4, RASGRP1, TRHDE, ACMSD, ADH4, ADAMTS15, ADAMTS14, RNF214, RNF213, PITRM1, ADAMTS18, MICAL1, LONRF2, CPA5, ZFHX3, CREBBP, ZRANB1, PHC1, PHF10, BRPF3, SIAH1, NFXL1, PHC3, ST18, ESR2, THAP11, MEX3A, TP53I3, MKRN2, LHX4, ZCCHC3, KDM5B, THRA, TGFB1I1, FHL1, OTUD7A, LDB3, ING1, NR2E3, MMP21, LIMD2, MMP20, TRIM8, ZCWPW1, CSRP2, MMP24, CALB1, TRIM7, CA5B, HNF4A, ARIH2, RNF151, RNF150, ADAMTS8, UNKL, ADAMTS9, TRIM67, TRIM62, APOBEC2, USP49, DTX3L, DDX58, APOBEC4, ZDHHC16, ZDHHC17, TOPORS, ZDHHC14, RNF168, RNF167, RNF169, DBF4, TRAF5, RARB, MMP19, ITGB1BP2, L3MBTL4, PAPPA2, RNF32, MGRN1, NR1I3, PAPLN, PRICKLE3, PRICKLE1, NR2C2, SAMHD1, DCST1, GLRA1, NRAP, RNF39, ZSWIM8, TRIM46, XIRP2, ZSWIM7, ZSWIM4, POLR2I, TRIM45, RNF24, PCGF2, PEX12, SKI, AGBL5, RNF146, ZNFX1, LNX1, TRIM31, LIMS2
GOTERM_MF_DIRECT	GO:0005242	inward rectifier potassium channel activity	7	0.002959	-	KCNH2, KCNJ12, KCNJ9, KCNJ13, KCNJ14, KCNJ2, KCNJ3
GOTERM_MF_DIRECT	GO:0005245	voltage-gated calcium channel activity	10	0.002991	-	TMC1, CACNB2, CACNA1B, CACNA2D2, CACNA1A, CACNA1C, CACNA1S, CACNA2D4, PKD2, CACNA1E
GOTERM_MF_DIRECT	GO:0005248	voltage-gated sodium channel activity	7	0.004459	-	HCN3, SCN10A, SCN8A, SCN9A, PKD2, SCN3A, SCN1A
GOTERM_MF_DIRECT	GO:0008331	high voltage-gated calcium channel activity	6	0.004977	-	CACNB2, CACNA1B, CACNA1A, CACNA1C, CACNA1S, CACNA1E
GOTERM_MF_DIRECT	GO:0005543	phospholipid binding	12	0.007638	-	PSD, SPTBN4, APOM, F10, PSD3, SYT16, AGAP1, PLA2G7, SPTB, APOA5, SPTBN1, PACSIN1
GOTERM_MF_DIRECT	GO:0005267	potassium channel activity	6	0.016093	-	KCNK7, KCNK10, ABCC8, PKD2, TMEM175, KCNK4
GOTERM_MF_DIRECT	GO:0003700	transcription factor activity, sequence-specific DNA binding	82	0.025792	-	ZNF496, NRL, ZNF45, TFCP2L1, PRDM1, ELK4, CREB3L4, HNF4A, HOXA3, SOX9, ZNF446, SOX6, ZNF367, ZHX2, ZNF480, ZFP2, RFX2, RFX3, FOXP3, RUNX3, POU5F1, HIC1, FOXP2, RUNX1, ETV6, FOXP1, CREB3, ZNF837, RFX7, DDIT3, IRF2, ZSCAN23, ZNF711, IRF5, ATF5, MZF1, L3MBTL4, ZNF232, HOMEZ, NFAT5, NR1I3, FOXK1, FOXO4, ZNF3, NR2C2, ZNF629, ELMSAN1, ZNF789, ZBED1, PLAGL2, HIVEP3, HIVEP2, E2F5, RREB1, ZNF189, ZNF286A, TCF7L2, POU2F1, FOXJ3, PBX3, NFATC3, NFXL1, PBX2, TBX6, ZNF79, SMAD6, TRERF1, ESR2, ST18, ZNFX1, TADA2A, NFIA, TEF, TBX10, ZNF616, NFIC, TAF5, SIM1, ZNF334, ZNF213, ZNF772, TP73
GOTERM_MF_DIRECT	GO:0008201	heparin binding	17	0.027402	-	POSTN, COL13A1, WISP3, ELSPBP1, NAV2, THBS2, APOA5, AGER, CYR61, WISP1, CXCL10, ADAMTS15, ABI3BP, OGN, SLIT1, RSPO2, SLIT3
GOTERM_MF_DIRECT	GO:0016594	glycine binding	5	0.029127	-	GLRA1, SRR, GRIN3A, GNMT, GRIN1
GOTERM_MF_DIRECT	GO:0004222	metalloendopeptidase activity	19	0.030989	-	PAPLN, MMEL1, PHEX, MMP21, MMP20, ADAMTS15, ADAMTS14, ADAM28, ADAM17, MMP24, PITRM1, ADAMTSL4, ADAMTS18, ADAM12, MMP19, RCE1, ADAMTS8, ADAMTS9, PAPPA2
GOTERM_MF_DIRECT	GO:0003677	DNA binding	91	0.032025	-	ZNF496, KDM5B, ONECUT2, ZBTB25, BNC2, CHD9, POGK, CHD6, HIST2H2AB, OTUD7A, ZBTB20, RORA, PRDM2, HIF3A, HIST2H2AC, EME2, SPATA24, HIST3H2BB, SOX6, HIST1H2AC, POLG, PIAS4, ZHX2, KDM2B, ZBTB37, HIST1H2AJ, RECQL, RFX2, RFX3, LIG3, DNTT, TFIP11, RUNX3, POU5F1, TOPORS, RC3H2, FOXP2, SMC1B, RUNX1, DNAJC2, ZEB2, RAD51D, NCOR1, ZEB1, SETBP1, DMTF1, KAT6B, RFX7, AKAP9, ZNF711, FAN1, AHDC1, HOMEZ, HIST1H2BM, RTEL1, NR1I3, BAZ2B, RECQL4, RECQL5, ELMSAN1, TCEB3, E2F5, DEDD, NHEJ1, ZFHX3, EGR2, MBD6, XRCC3, NFATC3, RFXANK, TBX6, TRERF1, MEIS2, ESR2, THAP11, HIPK2, RAD52, LBX2, TADA2A, GON4L, NFIA, H2AFY2, CSDC2, POLR3C, TBX10, NFIC, POU2AF1, NOSTRIN, POLR3H, SIM1, MXD3
GOTERM_MF_DIRECT	GO:0003774	motor activity	9	0.034702	-	MYH1, MYH7B, MYO3B, MYO10, MYH15, MYO18B, MYH11, MYH10, MYO16
GOTERM_MF_DIRECT	GO:0005030	neurotrophin receptor activity	3	0.036289	-	NTRK1, NTRK2, NTRK3
GOTERM_MF_DIRECT	GO:0008307	structural constituent of muscle	5	0.039822	-	MYBPC3, CAPN3, NEXN, MYH11, JPH1
GOTERM_MF_DIRECT	GO:0005089	Rho guanyl-nucleotide exchange factor activity	14	0.047424	-	FARP2, FARP1, PLEKHG7, ARHGEF39, KALRN, FGD1, FGD2, BCR, FGD3, TIAM2, AKAP13, OBSCN, ARHGEF4, MCF2L2
