# Supplementary material for: Identification, Characterization, and Transcriptional Reprogramming of Epithelial Stem Cells and Intestinal Enteroids in Simian Immunodeficiency Virus Infected Rhesus Macaques
Source: Front Immunol. 2021 Nov 23;12:769990. doi: 10.3389/fimmu.2021.769990 (PMC8650114; doi:10.3389/fimmu.2021.769990)
Supplement: Supplementary file 14 [file Table_8.pdf]

Supplementary Table 8: The 38 significantly enriched GO terms in the Molecular Function Category among downregulated DEGs

Category	GO:number	Term	Count	PValue	FDR	Genes
GOTERM_MF_DIRECT	GO:0044822	poly(A) RNA binding	259	1.13E-27	1.36E-24	EIF4A1, POP7, TES, TFRC, ATP5C1, CISD2, HNRNPU, HNRNPR, RRP9, RPL7, CCAR1, TIAL1, LGALS3, RPS19, MAGOH, CPNE3, SNRPD3, RPS11, RPS12, SEC63, DDX18, MBNL2, RPL23, NGDN, DNTTIP2, CDC40, DHX40, DDX39A, DDX39B, EWSR1, CLNS1A, XRN2, SUCLG1, SRFBP1, PFN1, EIF1B, RPL28, ANP32A, DHX9, NMD3, MRPL15, RTF1, HTATSF1, MRPL10, DHX30, MRPL20, C7H14ORF166, DHX35, WBSCR22, CSTF1, LTA4H, RBM12, LYAR, XRCC6, MRPL28, LARP7, MCAT, EIF1, MRPL22, HNRNPL, CCT6A, CS, RPS25, HNRNPM, EIF5, TBL2, HNRNPK, FUBP3, SPATS2L, GRB2, NOL11, RPS21, DZIP3, RPS23, SUGP1, DAZAP1, GFM1, FASTKD1, MRPS14, RPLP0, SRP54, NKAP, ADK, MRPL39, PCSK9, PHF6, SYNCRIP, NKRF, TMSB4X, DHX15, ANXA7, MRPS28, ANXA2, STRBP, NSUN2, PABPC4, MRPS23, PRPF40A, PPHLN1, NUDT16L1, GLTSCR2, UCHL5, GNL3, MRPL44, HADHB, NSA2, HNRNPH1, CSDE1, CDC42EP4, FRG1, STRAP, HNRNPH3, MAPRE1, ALDOA, CSTB, GRN, MRPS35, RPL11, USO1, ATP5A1, NOL7, RPS27L, MRPS30, CTNNA1, RPL15, CCT4, NQO1, NPM1, YTHDF1, YTHDF2, SRP68, NPM3, LRPPRC, KTN1, ARCN1, PHF5A, FAU, ACAA2, PEBP1, MSI2, APEH, SRP14, RPL10A, IPO5, PPP1CC, FBL, ZNF207, SNIP1, ZNF326, MTPAP, CHCHD1, CAST, PDIA3, RPS9, SUPT16H, RPS7, LARP4B, WBP11, GTF2F1, ILF3, FSCN1, SRSF3, ERH, SRSF5, PPIG, PPIB, SNRPB, ZCCHC7, DDX5, VCP, ZCCHC6, RPS19BP1, YTHDC2, DDX1, UBFD1, NTPCR, RTN4, GTF2E2, RPF1, ATXN2, DNAJC21, H1FX, LLPH, ZC3H15, MARK2, TIA1, CPSF7, UBE2I, PNPT1, FUS, LSM4, LSM6, MKRN1, UBA1, GRSF1, CCDC47, DDX42, HMGB2, HMGB3, YBX1, DDX41, MRPL4, MRPL3, SUMO1, ZC3H7A, ZNF768, GLRX3, NCBP1, DDX56, DDX54, SAFB, YWHAZ, DDX50, SYF2, RPL37A, RBMS1, RP9, SNRPA1, ETF1, SLC25A5, EIF4E2, SLC25A11, RBMS2, RBM22, DDX28, RBM47, USP10, GTPBP10, MTIF2, HSD17B10, MRM1, TBRG4, REPIN1, MTHFSD, GANAB, U2AF2, POLR2B, RALY, HNRNPA1, EIF4E, PTCD3, PTPN1, SSB, CNBP, CDC5L, IMMT, U2SURP, MFAP1, EIF3L, EIF3H, RBM42, EIF3E, SLIRP, SSBP1, FAM98A, NAA15, EIF3D, RAN
GOTERM_MF_DIRECT	GO:0019003	GDP binding	21	7.79E-08	4.71E-05	RAB2A, RAB5B, RAB4A, RALA, RALB, RAB5C, SRP54, GEM, RAB22A, RAB11B, RAP2C, RAP1B, RAB21, RAP2B, RRAGC, RAB14, RAB18, RAB28, RAB8A, RAN, ARL8B
GOTERM_MF_DIRECT	GO:0005525	GTP binding	83	1.68E-06	6.78E-04	ARF3, HBS1L, GFM1, ARF1, RAB3D, SRP54, OLA1, RND3, RAB22A, THG1L, TUBA1B, ARL5B, OPA1, ANXA6, RAC1, ARL5A, HRAS, RAB8A, RAB2A, RAB2B, ARL14, RRAS2, RHOA, GEM, TUBA4A, GNL3, RAP2C, RAB32, EEF1A1, ARL4C, ARL4A, RAP2A, RAP2B, RRAGC, ADSSL1, RABL3, RHOU, RHOV, ARF5, ARL8A, PAOX, ARL8B, RABL6, RAB1A, RALA, RAB5B, RALB, RAB5C, TUBAL3, SAR1B, ATL2, GTPBP10, AK3, ARL2, MTIF2, RAP1B, RAB21, ARFRP1, RHOT2, NRAS, RAP1A, RAB40C, RAB25, GNA12, MFN1, RAB28, ADSS, RAB6B, PCK2, RAB4A, GCH1, TUBE1, RAB11A, RAB11B, EHD1, EIF2S3, EHD4, RAB14, RHEB, RAB13, RAB18, DRG1, RAN
GOTERM_MF_DIRECT	GO:0003743	translation initiation factor activity	30	6.18E-06	0.001869	EIF4A1, GTF2A2, EIF5A2, MTIF2, GTF2E2, EIF4EBP1, EIF2D, EIF4E, EIF2A, EIF5A, EIF2B5, TAF12, EIF1AY, TAF13, EIF1, EIF3M, EIF2S3, EIF5, EIF3L, EIF3I, EIF3J, TAF7, EIF3H, EIF3E, EIF3F, EIF4E2, TAF4, EIF3D, EIF1B, TAF2
GOTERM_MF_DIRECT	GO:0003735	structural constituent of ribosome	82	2.22E-05	0.005375	RPL4, SLC25A1, SLC25A3, MRPS16, RPL3, MRPS14, RPL34, RPLP0, RPS17L, MRPL34, RPL10A, MRPL33, RPL7, RPS15, MRPL4, MRPL3, RPS14, RPS19, RPL35, RPS11, RPS13, RPS12, RPS9, RPL21, RPS7, MRPS25, RPS8, RPL23, RPS5, MRPS23, MRPS18A, RPL13A, MRPS2, MRPS21, MRPL46, SLC25A15, SLC25A17, MRPL51, RPL37A, RPL27, SLC25A10, RPL26, SLC25A5, SLC25A11, RPL28, SLC25A4, SLC25A13, RPS4Y2, MRPS35, MRPL18, RPL12, RPL11, RPS27L, MRPL15, MRPS30, MRPL13, SLC25A27, MRPL20, RPS3, SLC25A20, RPL15, SLC25A22, SLC25A24, MRPL28, MRPL24, MRPL22, RPS26, SLC25A38, SLC25A37, SLC25A39, RPS29, RPL27A, SLC25A30, RPL22L1, RPS20, FAU, RSL24D1, RPS21, SLC25A33, RPS23
GOTERM_MF_DIRECT	GO:0003924	GTPase activity	45	2.73E-05	0.005417	RAB1A, HBS1L, GFM1, RALA, RAB5B, DDX3X, RALB, RAB5C, TUBAL3, ATL2, SRP54, GTPBP10, MTIF2, RAB22A, RAP1B, RAB21, TUBA1B, RHOT2, RAP1A, OPA1, GNA12, MFN1, RAC1, HRAS, RAB8A, RAB2A, RAB4A, TBCC, TUBE1, RHOA, RAB11A, GEM, TUBA4A, RAB11B, GNL3, RAB32, EEF1A1, RAP2A, EIF2S3, RRAGC, RAB14, GNB1, RAB13, RHOU, RAN
GOTERM_MF_DIRECT	GO:0004197	cysteine-type endopeptidase activity	20	3.13E-05	0.005417	USP7, USP15, USP16, USP10, USP33, CTSZ, CTSV, CTSS, CASP9, CASP7, CASP8, CTSL, CASP3, CTSK, CASP4, CTSH, BLMH, CTSC, CTSB, LGMN
GOTERM_MF_DIRECT	GO:0000166	nucleotide binding	71	1.08E-04	0.016379	DAZAP1, CELF1, MRPL39, HNRNPR, CELF6, MSI2, ELAVL1, TIAL1, SYNCRIP, RAVER1, SNRNP70, RBM7, RBM5, NCBP2, PABPC4, RBM18, LARP4B, LEMD3, SAFB, NT5C3B, EWSR1, HNRNPH1, ESRP1, PABPC3, SRSF3, RBMS1, SRSF5, HNRNPH3, R3HCC1L, RBMS2, RBM22, RBM47, RBM48, TMEM63B, SRSF1, U2AF1, HNRNPLL, HTATSF1, ADCY6, MTHFSD, RRP7A, PABPN1, U2AF2, TRA2B, G3BP1, POLR2D, G3BP2, RDH14, SNRPB2, RALY, HNRNPA1, RBM12, RBM39, TIA1, HNRNPA3, SSB, FUS, LARP7, UPF3A, NMI, U2SURP, HNRNPL, HNRNPM, RCAN1, HNRNPA2B1, RBM42, RNPS1, ATP13A2, SLIRP, GRSF1, CPEB2
GOTERM_MF_DIRECT	GO:0003729	mRNA binding	29	1.40E-04	0.018816	CELF1, FMR1, SRSF1, PARK7, HNRNPLL, MRPL13, RPL7, FXR1, PCF11, SNRNP70, TRA2B, G3BP1, RPS3, G3BP2, RPL35, PAIP2, SF3B1, RPS13, RBM5, NCBP2, RPS5, MBD2, RPL13A, TDRD7, THOC5, RPS26, NUDT21, ESRP1, LUC7L3
GOTERM_MF_DIRECT	GO:0009055	electron carrier activity	19	2.26E-04	0.027385	GCDH, GLRX3, NDUFA12, GLRX5, ACAD9, SDHC, ETFA, GLRX, GLRX2, ETFB, UQCR, SDHB, ACADSB, ACOX2, FDX1, CIAPIN1, ACADM, ACAD10, ACADS
GOTERM_MF_DIRECT	GO:0000062	fatty-acyl-CoA binding	12	2.49E-04	0.027385	GCDH, SOAT1, ACOX2, SOAT2, ECI2, ACAD9, DBI, ETFA, ACADM, ACAD10, ACADSB, ACADS

GOTERM_MF_DIRECT	GO:0003723	RNA binding	74	5.34E-04	-	PRKCSH, RPL34, FMR1, PARS2, THUMPD2, MRPL39, RPL10A, EPRS, TIAL1, RPS15, MRPL4, FBL, CWC22, PAPOLA, EIF2D, SNRPD3, EARS2, DDX18, KHNYN, PABPC4, STRBP, RNASET2, DDX54, PRPF40A, EWSR1, RPUSD1, TEP1, RBMS1, SNRPF, RBMS2, RBM22, SNRPB, TRUB1, SF3B3, YTHDC2, U2AF1, HNRNPLL, HTATSF1, MRM1, HSP90B1, FXR1, ZMAT2, U2AF2, UBR5, SMN1, RBBP7, HNRNPA1, RBM12, PAIP1, MCTS1, RBM39, TIA1, YTHDF1, YTHDF2, SSB, PNPT1, LARP7, PUS7L, LRPPRC, U2SURP, PUM2, HNRNPL, HNRNPM, LSM6, HNRNPK, QARS, FUBP3, RPS29, RPS20, GRSF1, TRIM32, DZIP3, EIF4G2, SUGP1
GOTERM_MF_DIRECT	GO:0003995	acyl-CoA dehydrogenase activity	9	5.71E-04	-	GCDH, ACOX2, ACAD9, NPHP3, ETFA, ACADM, ACAD10, ACADSB, ACADS
GOTERM_MF_DIRECT	GO:0046961	proton-transporting ATPase activity, ro	11	7.83E-04	-	ATP6V1A, ATP6V0B, ATP6V0E1, ATP5D, ATP5A1, ATP6V1H, ATP5C1, TCIRG1, ATP6V1E1, ATP6V0E2, ATP6V0C
GOTERM_MF_DIRECT	GO:0003824	catalytic activity	33	8.28E-04	-	CDA, ACSS3, ACSS2, HPGD, ECI2, SPATA20, CLYBL, AACS, PLD3, SPTLC1, HINT3, PMPCB, ACSS1, PCYT2, PPTC7, OXSM, LANCL1, ECH1, ACSL5, ACSL4, GTF2F1, ADAT3, PIGC, AMACR, SUCLA2, ILVBL, CWF19L1, UQCRC1, SUCLG2, ECHDC3, SCLY, PIGG, MOCS1
GOTERM_MF_DIRECT	GO:0004004	ATP-dependent RNA helicase activity	20	0.001154	-	DDX28, DDX18, EIF4A1, DDX5, DDX3X, DHX9, DDX1, YTHDC2, DDX56, DDX42, DDX54, DDX41, DDX50, DHX40, DHX30, DDX19B, DDX39A, DDX39B, DHX35, DHX15
GOTERM_MF_DIRECT	GO:0061631	ubiquitin conjugating enzyme activity	10	0.001509	-	UBE2Q1, UBE2R2, UBE2B, UBE2D2, UBE2V2, UBE2D1, UBE2G1, UBE2G2, UBE2A, UBE2J2
GOTERM_MF_DIRECT	GO:0008137	NADH dehydrogenase (ubiquinone) ac	13	0.001601	-	NDUFB9, NDUFA8, NDUFA7, NDUFB7, NDUFA12, NDUFA4, NDUFB4, NDUFA10, NDUFC2, NDUFS8, NDUFS7, NDUFS4, NDUFV1
GOTERM_MF_DIRECT	GO:0015485	cholesterol binding	13	0.002231	-	OSBPL8, OSBPL7, STARD5, OSBPL2, ERLIN1, APOA4, ERLIN2, TSPO2, SOAT1, SCP2, NPC2, SOAT2, ANXA6
GOTERM_MF_DIRECT	GO:0008565	protein transporter activity	20	0.002277	-	RAB4A, TIMM9, COG3, USO1, AP1B1, VPS26A, AP2B1, RANBP6, IPO7, IPO5, IPO8, RAP1A, AP1S1, KPNA4, AP3S1, AP1S3, KPNA2, SEC62, TNPO2, CHMP7
GOTERM_MF_DIRECT	GO:0051287	NAD binding	13	0.003049	-	HIBADH, HPGD, CTBP1, IDH1, IDH2, UGDH, BDH2, GPD1, NDUFS2, GAPDH, NDUFV1, IDH3A, HTATIP2
GOTERM_MF_DIRECT	GO:0004298	threonine-type endopeptidase activity	10	0.003527	-	PSMB6, PSMB4, PSMA4, PSMB5, PSMB2, PSMA1, PSMB3, PSMA2, PSMB1, PSMB8
GOTERM_MF_DIRECT	GO:0015078	hydrogen ion transmembrane transport	12	0.004063	-	ATP6V0B, SLC11A2, ATP5J, TCIRG1, ATP5G3, ATP5H, ATP5F1, ATP6V0D1, ATP6V0E2, ATP5G1, ATP6V0C, ATP5L
GOTERM_MF_DIRECT	GO:0000287	magnesium ion binding	39	0.005	-	PDXK, MAST2, GTPBP10, ENO2, CLYBL, NUDT3, THG1L, STK3, PRPSAP1, RPS6KA3, WRN, RPS6KA1, STK38L, RDH14, MTPAP, ADSS, ATP9B, PGM1, MARK2, IDH3A, MAP3K5, TESC, IDH1, EPHX2, CDC42BPG, ITPK1, IDH2, OXSR1, GEM, PPM1A, NT5C3B, GCLC, ILVBL, PPA1, ADPRH, ADSSL1, PGP, HPRT1, DCTPP1
GOTERM_MF_DIRECT	GO:0050660	flavin adenine dinucleotide binding	18	0.005005	-	GCDH, ACAD9, ETFA, FMO4, SDHA, FMO5, ACADSB, POR, ACOX2, AIFM2, CHDH, AGPS, LDHD, NPHP3, ACADM, ACAD10, DLD, ACADS
GOTERM_MF_DIRECT	GO:0052890	oxidoreductase activity, acting on the C	7	0.006948	-	GCDH, ACOX2, ACAD9, ETFA, ACADM, ACAD10, ACADSB
GOTERM_MF_DIRECT	GO:0070403	NAD+ binding	7	0.006948	-	ALDH1A3, HPGD, CRYL1, SIRT6, SIRT7, HADH, SIRT2
GOTERM_MF_DIRECT	GO:0016787	hydrolase activity	32	0.007855	-	IDI1, VCP, ABHD3, ABHD8, OPLAH, USP19, HDAC7, ABHD13, NUDT16, ACAD10, LYPLA1, ENTPD4, EPHX2, ENTPD6, FAAH2, CMBL, NUDT16L1, MRPL46, HDHD3, GCHFR, NUDT22, NUDT21, PSMC5, PNKD, NUDT8, XPNPEP1, ADPRH, HAGH, SMPDL3A, PAFAH1B2, PNPLA2, PAFAH1B1
GOTERM_MF_DIRECT	GO:0003857	3-hydroxyacyl-CoA dehydrogenase ac	5	0.007908	-	HADHB, HSD17B4, CRYL1, HADH, HSD17B10
GOTERM_MF_DIRECT	GO:0051539	4 iron, 4 sulfur cluster binding	10	0.00991	-	ISCA1, NDUFS8, NDUFS7, NTHL1, ACO1, MOCS1, SDHB, NDUFV1, ISCU, NUBP2
GOTERM_MF_DIRECT	GO:0004364	glutathione transferase activity	10	0.00991	-	GSTZ1, EEF1G, GSTM4, GSTA4, GSTA3, GSTO1, MGST3, GSTP1, MGST1, MGST2
GOTERM_MF_DIRECT	GO:0031625	ubiquitin protein ligase binding	14	0.023467	-	UBE2B, CUL3, CUL2, CUL1, UBE2G1, UBE2G2, UBE2A, UBE2J2, CUL4A, UBE2R2, UBE2V2, SPOPL, UBE2K, ANAPC2
GOTERM_MF_DIRECT	GO:0051537	2 iron, 2 sulfur cluster binding	8	0.024601	-	ISCA1, GLRX5, FDX1, CISD1, CISD2, CIAPIN1, SDHB, ISCU
GOTERM_MF_DIRECT	GO:0030165	PDZ domain binding	5	0.028077	-	CXADR, PLEKHA1, KIDINS220, CRIPT, ATP2B1
GOTERM_MF_DIRECT	GO:0005484	SNAP receptor activity	11	0.032231	-	VAMP8, VAMP7, STX8, STX5, VAMP4, SEC22B, VAMP5, YKT6, VAMP2, STX10, VAMP3
GOTERM_MF_DIRECT	GO:0031369	translation initiation factor binding	4	0.033099	-	GLE1, POLR2D, POLR2G, EIF3F
GOTERM_MF_DIRECT	GO:0008234	cysteine-type peptidase activity	7	0.033687	-	SENP6, CTSK, CTSZ, CTSH, BLMH, CTSC, CTSS
GOTERM_MF_DIRECT	GO:0051015	actin filament binding	9	0.0486	-	CAPZB, ARPC1B, ARPC1A, FRG1, FSCN1, PLS3, CORO2A, CORO1B, PLS1
