# Supplementary material for: Identification, Characterization, and Transcriptional Reprogramming of Epithelial Stem Cells and Intestinal Enteroids in Simian Immunodeficiency Virus Infected Rhesus Macaques
Source: Front Immunol. 2021 Nov 23;12:769990. doi: 10.3389/fimmu.2021.769990 (PMC8650114; doi:10.3389/fimmu.2021.769990)
Supplement: Supplementary file 15 [file Table_9.pdf]

Supplementary Table 9: The 15 significantly enriched GO terms in the Cellular Components Category among upregulated DEGs						
**Category**	**GO:number**	**Term**	**Count**	**PValue**	**FDR**	**Genes**
GOTERM_CC_DIRECT	GO:0005887	integral component of plasma membrane	118	3.31E-05	0.0193	KLB, RXFP4, ICAM2, ICAM3, BEST2, HTR4, BEST1, KDR, SLC39A5, SLC16A8, PLXNC1, SLC16A2, SLC16A4, KCNH1, KCNH2, KCNH4, DSCAM, KCNK10, SLC6A17, ADAM17, SLC5A9, SLCO2A1, PMEL, SLC26A1, HVCN1, ABCB5, FPR1, SLCO5A1, APCDD1, SLC5A2, FLRT1, TSPAN5, PLXNA4, SLC10A4, SLC14A2, CADM4, SLC10A1, ABCA4, LRRN4, TRPV2, SLC4A10, SLC16A11, ATP4A, IL17REL, TRPV4, SLCO1A2, SSPN, SLC26A8, CSPG4, FGFR2, SLC26A5, KCNK7, GPR22, STAB2, HTR2B, PKD2, LTB4R, EDA2R, GRM1, SLC22A13, UPK1B, MMP24, BOC, CD37, SLC18A2, SLC13A1, SLC13A3, TRPC5, TMEM150A, SLC13A4, TRPC3, AVPR1B, GNRHR2, SLC2A10, SLC2A12, TACR2, IL17RD, SSTR2, SSTR3, IL17RB, BACE1, SLC7A5, SLC7A6, DDR2, HCN3, SLC24A1, SLC24A3, SAMD8, SLC5A11, SLC5A10, GLRA1, LRRC8C, HAS3, LRRC8D, KCNJ2, KCNJ3, NTRK1, NTRK2, SLC12A5, CD72, KCNJ11, KCNJ12, KCNJ9, NTRK3, KCNJ13, KCNJ14, TSHR, XG, P2RX7, SLC8A3, SLC4A8, OPN1SW, SLC4A9, TSPAN18, SLCO4C1, ATP13A4, RHAG, KCNK4
GOTERM_CC_DIRECT	GO:0005891	voltage-gated calcium channel complex	10	1.16E-04	0.03381	CACNB2, CACNA1I, PDE4D, CACNA1B, CACNA2D2, CACNA1A, CACNA1C, CACNA1S, CACNA2D4, CACNA1E
GOTERM_CC_DIRECT	GO:0005578	proteinaceous extracellular matrix	32	0.0024946	-	FBN2, FBN3, COL15A1, PAPLN, WISP3, CYR61, WISP1, COL19A1, COCH, HAPLN2, ADAMTS15, VTN, ADAMTS14, ADAMTS18, SLIT1, COL10A1, SLIT3, EMILIN1, ADAMTS8, ADAMTS9, WNT4, POSTN, OMD, TFIP11, BCAN, COL4A2, LOX, COL4A1, OGN, COL6A3, GPLD1, FBN1
GOTERM_CC_DIRECT	GO:0031012	extracellular matrix	22	0.0033059	-	FBN2, FBN3, SPON1, COL14A1, TGFB1I1, AEBP1, LTBP3, THBS2, FBLN2, COCH, MMP21, MMP20, MMP24, ADAMTSL4, EMILIN2, COL8A1, MMP19, IGFBP7, ZP3, TGM4, FGFR2, FBN1
GOTERM_CC_DIRECT	GO:0008076	voltage-gated potassium channel complex	15	0.0039701	-	KCNH2, CNTNAP1, KCND1, KCNJ11, ABCC8, KCND3, KCNIP2, KCNB2, KCNIP4, AKAP9, KCNMA1, CNTN2, KCNA10, KCNJ2, KCNH1
GOTERM_CC_DIRECT	GO:0030018	Z disc	14	0.0120749	-	RYR2, NEXN, LDB3, JPH1, SYNPO2L, MYOT, BAG3, MYO18B, XIRP2, KCNN2, STUB1, ITGB1BP2, CRYAB, SCN1A
GOTERM_CC_DIRECT	GO:0005856	cytoskeleton	20	0.0165599	-	FARP2, FARP1, FRMPD3, MYO10, NR1I3, HTRA2, FRMD4A, LDB3, PTPN14, PPL, PREPL, FRMD5, FRMD7, PRC1, SNTG1, EVL, NF2, TLN2, JAK3, PLEKHH1
GOTERM_CC_DIRECT	GO:0005930	axoneme	11	0.022857	-	SPTBN5, RSPH4A, IFT140, IFT172, TCTEX1D4, AMBRA1, MAPT, ATG14, CCDC151, AK8, DNALI1
GOTERM_CC_DIRECT	GO:0030688	preribosome, small subunit precursor	4	0.0237898	-	NOB1, TSR1, RRP1B, FTSJ3

GOTERM_CC_DIRECT	GO:0005737	cytoplasm	332	0.0246707	-	SLC46A1, MTRR, SLC23A1, THUMPD3, MT1X, WDR83, HTR4, HERC5, ELK4, ENDOU, SYNGAP1, SPATA24, FAM65C, HIST3H2BB, BTRC, SLC34A3, PIH1D1, RECQL, WDR72, ARMC7, RUNX3, ANK1, MIF4GD, RC3H2, RUNX1, TIAM2, INPP4A, RNF123, KATNAL2, DDIT3, SIK3, PIH1D2, PRKCQ, BCORL1, ATF5, NAIP, SPTBN5, NUP205, MTMR3, COPS7B, MEFV, OAZ3, STRIP2, NUAK1, DHX32, LARP1, DHX33, XRRA1, MICAL1, FLRT1, PNPO, ZBED1, TP53BP1, SCN3A, RREB1, TOE1, HEXIM1, HEXIM2, FZD3, AFAP1L2, ZRANB1, EGR2, CREBBP, DNASE2B, INCA1, PLK1, SIAH1, NFATC3, BTBD11, NTAN1, THAP11, FAM111A, ALS2CR12, CABYR, PPP1R1C, PAF1, CPEB3, FERMT2, BTG3, CLIC3, SETD6, STAB2, LRRC4, OTUD7A, TFCP2L1, ARHGAP6, M1AP, FCRLA, THTPA, TRIM7, HNF4A, SUFU, XPO5, CNTRL, ARIH2, TNFAIP8L2, CEP68, MIOX, ZHX2, SERPINB2, ZNF480, SLC2A10, NCOA3, GPBAR1, PAWR, IL16, GP1BA, DNTT, GAB2, GFRA3, POU5F1, IL17RB, RNF168, RNF167, SLC7A5, ZEB1, RILPL1, TCP1, ACSBG1, MAPT, GART, PHLPP2, TRUB2, FOXC1, NR1I3, FAM129A, ADCY3, CCDC106, RECQL4, PSMB7, TUBA3C, SRPX2, RECQL5, TCOF1, CHN2, HAS3, APBB3, DEDD, FARP2, FARP1, HIPK4, CDKN2C, MPP4, CRBN, RFTN1, YLPM1, KRT10, PRSS36, ELP5, GNMT, HIPK2, SKI, ATAT1, KBTBD4, TMCO6, ABI2, ABI3, CAMK4, HYLS1, GHRL, TACC2, PLCD4, ERCC6L, ANKRD13D, ZBTB25, NUCKS1, PRDM1, SYDE1, STK10, TUBB1, CAPN3, OIP5, PTRF, RGS6, PIAS4, THOC1, VASH2, CDC25C, FOXP3, SRCIN1, HIC1, PRSS54, GLTPD2, ADAM17, FRMD5, TXLNA, DMTF1, TSSK3, SNRNP25, FSCN2, TSSK4, CARS2, TSSK2, KPNB1, HOMEZ, BRSK1, RBM8A, ANKRD11, GSTCD, KLHL10, CACNA1A, AK5, CACNA1C, SPATA5, PKMYT1, TRHDE, NPAS3, STRA8, FAM71D, PDZD2, STK36, SNTG1, CHAD, CASKIN1, TSNAXIP1, SUDS3, SRGAP1, IP6K3, MFAP3L, WNT4, FANCI, ZFHX3, RBPMS, BDNF, ANKRD23, IFT122, FANCC, RFXANK, SMAD6, MCC, POU2F3, YRDC, PTPRE, PDCL, AAK1, PI4KA, C15H9ORF78, NF2, RAD9A, KDM5B, PPP1R13B, DOCK3, BNC2, CHD9, FHL1, HTR2B, SYNE4, MEA1, DUSP16, DNALI1, WISP1, SPRED1, DNMT3L, SIN3B, GPER1, GDAP1L1, EMILIN3, DACT2, BLNK, POLH, TRIM67, TRIM62, POU1F1, DTX3L, DDX58, AKTIP, SPATS2, KCNAB1, RTN4RL2, TFIP11, TIRAP, SMC1B, ZAR1, ETV6, ACTA2, LRTOMT, TBC1D4, IMPAD1, ALDH1A2, RASA1, IRF2, CDH13, EVL, IRF5, FAN1, NFAT5, DNAH7, LEF1, CRMP1, NTN1, ASB16, SRR, KCTD20, NRIP2, NNAT, ANKAR, RNF39, CCL3, LRRC8D, SNCG, MICALCL, E2F5, MDH1B, JAZF1, TRIM45, TCF7L2, MCOLN3, MYO10, CMTM3, KCNIP2, NOS3, NTRK3, KCNIP4, MX1, DTNBP1, FBXL15, HSPE1, PTPN14, MSMP, MAPK12, CRYGC, DBNDD1, SNRK, CAPN12, GNPDA2, RPS6KB1, PRC1, LNX1, SPAG1, ACIN1, PFKM, SNTB2
GOTERM_CC_DIRECT	GO:0005581	collagen trimer	13	0.0249156	-	COL15A1, COL14A1, ELSPBP1, C1QL3, COL19A1, MFRP, COL4A2, LOX, COL4A1, COL8A1, COL6A3, COL10A1, EMILIN1
GOTERM_CC_DIRECT	GO:0043025	neuronal cell body	22	0.0252052	-	SNAP47, FZD3, SLC12A5, ELOVL5, TMPRSS3, SLC4A10, CACNA1B, CACNA1A, ERMN, CYP17A1, P2RX7, TANC1, GLRA1, SRR, CALB1, GRIN3A, FRMD7, CNTN2, SNCG, KCNN2, APOD, SCN1A
GOTERM_CC_DIRECT	GO:0005604	basement membrane	10	0.0365967	-	COL15A1, FRAS1, HMCN1, RELL2, THBS2, NID2, NTN1, FREM2, MATN2, FBN1
GOTERM_CC_DIRECT	GO:0014069	postsynaptic density	12	0.0383614	-	GPER1, ADGRB1, DTNBP1, RGS20, DLGAP1, CACNA1C, MAPT, CNIH2, BSN, GRIN2C, GRM1, GRIN1
GOTERM_CC_DIRECT	GO:0048786	presynaptic active zone	6	0.0492658	-	RIMS2, FZD3, SV2A, GPER1, BSN, PPFIA4
