# Supplementary material for: Identification, Characterization, and Transcriptional Reprogramming of Epithelial Stem Cells and Intestinal Enteroids in Simian Immunodeficiency Virus Infected Rhesus Macaques
Source: Front Immunol. 2021 Nov 23;12:769990. doi: 10.3389/fimmu.2021.769990 (PMC8650114; doi:10.3389/fimmu.2021.769990)
Supplement: Supplementary file 16 [file Table_10.pdf]

Supplementary Table 10: The 79 significantly enriched GO terms in the Cellular Components Category among downregulated DEGs

Category	GO:number	Term	Count	PValue	FDR	Genes
GOTERM_CC_DIRECT	GO:0070062	extracellular exosome	623	1.27E-57	1.01E-54	NCKAP1, CYFIP1, TFRC, ARPC5L, ATP5C1, GHITM, ENO2, F11R, LIPA, RPL7, PSMD6, PSMD7, ALCAM, TFG, SCP2, PSMD2, PSMD3, AP1S1, VPS36, TMEM8A, CHID1, IAH1, RPL23, PRKCD, RNASE4, SCAMP3, SCAMP2, CD2AP, ACE2, MYL6, BCAM, CLDN3, PSME1, PSME2, HPRT1, UQCRC2, RPL28, VPS25, TSTA3, TTYH3, SHMT2, VPS26A, SDSL, PRDX2, RHOT2, NCSTN, HSPH1, PRDX4, EPB41L2, PDGFC, SFN, LTA4H, S100A10, LYN, ST14, JUP, NEBL, FUCA1, TMEM192, ERLIN2, PRDX6, HNRNPL, CS, HNRNPM, HNRNPK, EHD4, GPR180, FXYD3, TOLLIP, GIPC1, PPT1, PAM, LGMN, ARF3, ARPC1B, ARPC1A, RPLP0, PRKAG1, TUBA1B, NIPBL, UBL3, RARS, ANXA6, TSPO, ANXA7, TIMP1, CTBS, LYPLA1, SERPINB1, ANXA1, ANXA2, PLLP, ANXA3, ANXA4, ANXA5, RRAS2, NUTF2, SERPINB5, SRP9, VAMP8, HADHB, PLSCR1, BDH2, VAMP7, PSMA4, PSMA1, TMEM33, PLSCR4, TMEM106B, VAMP5, ARF5, PAFAH1B2, PCYOX1, PAFAH1B1, GRN, ASAH1, FH, IDUA, URM1, GSTP1, RPL11, ATP5A1, DLST, HSPA13, FAM129B, PTGS1, PSMB6, LMAN1, PSMB4, NRAS, PSMB5, PSMB2, PSMB3, PPP1R7, ASNA1, PSMB1, RPS3, GCNT3, RPL15, BID, AOC1, SLC16A1, MDH1, GK, PSMB8, TM9SF2, TMEM59, ERP44, PSMC5, RNF149, ABI1, SERBP1, NUCB1, LAMTOR2, MITD1, PLP2, LAMTOR3, CCL28, FRK, FAM49B, TPMT, UBE2D2, RAB3D, CSE1L, CTNND1, PDCD5, SAT2, PARK7, APEH, METRNL, RPL10A, CLU, HID1, NPEPPS, SLK, APMAP, LAMP2, CFL1, CHP1, KPNA4, SLC26A11, NUDT14, AP2M1, PLS1, RAB2A, RAB2B, RPS9, RPS7, TPM3, RPS5, PGD, SDCBP2, TUBA4A, TPST2, TOM1L1, MINPP1, SYPL1, NAAA, S100A6, FSCN1, SMS, ADAM9, SNRPE, IL6ST, PLA2R1, SLC27A2, SQSTM1, ARL8A, FTL, ARL8B, SNRPB, RTN4R, RAB5B, RAB5C, GLO1, CUL3, AK2, ABHD8, TACSTD2, ABHD6, NTPCR, PHB, LIN7C, CORO1B, HSP90B1, RAP1B, TKFC, RAP1A, PMVK, ESD, PGK1, MPDU1, RAB4A, GOT1, GFPT1, CYBRD1, ARPC4, COPS4, COPS6, LSM6, ARPC2, RHEB, SERINC1, ACO1, IARS, NIT1, COPS8, SERINC2, SLC25A1, CPM, SLC25A3, COX4I1, ETFA, ETFB, PIK3C2A, UFSP1, ALAD, SCPEP1, PTER, SCRN2, ITGAV, ITGB6, PGM1, ACTR3, GPX2, PDCD6IP, ITGA3, LANCL1, VPS37C, AHSA1, APOA4, VPS37B, RRM2B, TST, DAD1, NPC2, RPL37A, TMBIM1, DSG2, SLC25A5, GAPDH, SMPDL3A, ALDH9A1, MTPN, RAB1A, NDUFB9, SRC, VPS4B, NDUFB4, SRSF1, NDUFB1, GLRX, SRI, PDHB, RAB21, GANAB, TTR, RAB25, MAL2, DNAJB9, PCBD1, HNRNPA1, EIF4E, SEC11A, ADSS, WASF3, CYB5A, NAGA, APOC3, CDC42BPB, DNAJA1, TSPAN15, MYO1B, MYO1C, SUCLA2, RAB14, MGAT4A, EIF3I, RAB13, RAB18, EIF3H, TRIM36, EIF3E, EIF4A1, ECI1, CISD1, NUDT3, ACTB, TIAL1, RPS14, LGALS3, C1GALT1C1, EFR3A, RPS19, BSG, ARL6IP5, TNFSF10, CPNE3, SNRPD3, ATP6V1E1, KIFAP3, RPS11, GLUL, GOLGA7, B2M, RPS13, SLC12A9, ATP6V1G1, ECH1, ENTPD6, TALDO1, MOGS, ACSL4, CMBL, TWSG1, BTD, PPA1, CSNK2B, VDAC3, VDAC2, GINM1, TAGLN2, SUCLG1, KDELR2, LAP3, PFN1, CFTR, PFN2, CUTA, SLC22A5, SDC4, STXBP3, MAOB, MGST3, MYL12B, SEC14L2, SNX3, SNX2, PDCD10, PPCS, TSPAN6, NEU1, SNX9, TSPAN3, TSPAN1, ATP6V1C1, PCK2, DECR1, GNG12, ACADSB, DERA, CCT6A, RPS25, MSRA, TF, RPS29, SDC1, CYSTM1, GRB2, ITM2C, AARS, HBS1L, AHCTF1, ECM1, CLIC4, GBE1, CTSZ, OLA1, AKR1B1, CTSV, N4BP2L2, WFDC2, RAB22A, KHK, MYDGF, NUDCD2, HINT3, CTSL, CHMP1A, SDF4, MUC13, CTSH, RAC1, CTSD, CTSC, RAB8A, CTSB, CCT2, MME, GAA, IL18, USMG5, ANO6, UCHL3, COMMD7, ITFG1, RHOA, APRT, RAP2C, UGDH, RAP2A, RAP2B, TAX1BP1, PABPC3, B3GNT2, VWA1, TAX1BP3, ALDOC, ALDOB, CD47, CD46, ALDOA, ATP6V0C, NAPA, CSTB, TOMM40, CAB39, TMPRSS2, CAPG, NEDD8, RFNG, COX5A, CST3, PPP2CB, SH3BGRL, SAMM50, CREG1, CD59, MGAT1, CD58, GPC4, CCT8, CCT5, CCT4, NQO1, MON2, NQO2, PLEKHA1, GDF15, UBE2G1, FAH, SVIP, RAB11A, RAB11B, CAPZA1, CAPZA2, HNRNPA2B1, CTNNB1, RAB3GAP1, SCARB2, SCARB1, ACAA2, CD82, CD81, PEBP1, SRP14, NARS, FBL, PLAU, FAM162A, CAPN2, FAM3B, COTL1, OSTF1, CAPN1, FAM3C, PDIA3, NSF, G6PD, SPINK1, APLP2, ANXA13, SHISA5, ADAM10, ATP1B3, ALDH3A2, DCTD, GPD1, UBE2V2, UBE2V1, PPIB, VCP, DDX5, PSMD12, TMED10, TSG101, NDRG3, PSMD14, SLC1A5, NDRG1, PLD3, RTN4, DPP4, SDCBP, EFNB1, UBB, ATP6V1H, PACSIN3, ATP6V1D, TBC1D15, PRNP, HSPA5, HSPA4, VTA1, EPHX2, IDH1, IDH2, DSTN, DNAJC13, QDPR, GNB1, CRYL1, BLMH, UBA1, UBE2K, GALK1, UBE2M, LGALS3BP, DDX3X, YWHAB, TREH, DBI, YBX1, C14H11ORF52, RND3, HEBP1, LASP1, YWHAQ, NAPRT, FLOT1, TMEM109, A4GALT, YWHAH, H2AFY, GLRX3, H2AFZ, DCXR, LSR, OXSR1, YWHAZ, EEF1G, ALDH1A3, ALDH1A1, CAT, ATP6V1B2, BLVRB, PFDN2, DNASE2, HAGH, PON3, DDC, PDXK, RALA, RALB, DCTN2
GOTERM_CC_DIRECT	GO:0005739	mitochondrion	217	1.10E-14	4.37E-12	VARS, PARS2, ECI1, ECI2, GHITM, MTFR1L, LACTB, SCP2, ZMIZ2, BSG, FDXR, ATP6V1E1, FBXO7, GLUL, ACAD10, EARS2, GABARAPL1, STARD5, TSTD1, OXSM, CMC2, ECH1, RSAD1, BOLA1, ACSL4, HDHD3, LACTB2, DDIT4, FDX1, JTB, LAP3, AURKAIP1, GCDH, URI1, MRPL18, TRMT2B, TXN, SDSL, MRPL10, PRDX4, NTHL1, BLOC1S2, LDHD, PCK2, DECR1, NDFIP2, PYCR1, GBAS, MRPL24, MCAT, ACADSB, MSRA, QARS, SFXN4, ECHDC3, COX7B, CLIC4, GFM1, FASTKD1, HIBADH, MRPL39, MRPL34, CLYBL, SPTLC2, RARS, ANXA6, TSPO, ACADM, ACADS, MRPS27, MRPS25, ACAD9, ATPIF1, MRPS23, NME4, MRPL44, HADHB, CTU1, MRPL50, RAD51C, SDHAF1, KIF1BP, AGPS, ALDOC, DLD, FH, OAT, GSTP1, MRPS33, TIMM13, NOL7, HSD17B4, DLST, MRPS30, MALSU1, PTS, MRPL55, TMEM65, PSMB3, EMC2, AGR2, RANBP2, BCAP31, SLC16A1, TIMMDC1, MDH1, GK, APEX2, SYNJ2BP, RAB11A, RAB11B, NUDT8, OTC, PECR, MTCH1, OXNAD1, ACAA2, TACO1, RAB3D, CPOX, CLU, THG1L, NARS, COL4A3BP, FAM162A, MACROD1, CAPN1, MTPAP, CHCHD1, AP2M1, SH3GLB1, PHYH, DGAT2, PPTC7, RAB32, ILF3, SLC9A6, DNAJC30, GPD1, SLC27A2, ABCG1, IREB2, ABHD6, UBB, PMAIP1, TBC1D15, MPDU1, UROS, IDH1, QDPR, CDK7, SCCPDH, AMACR, UQCRQ, ACO1, UBA1, NIT1, TCIRG1, HK1, MRPL4, CASP8, OPA1, CASP4, ABCD1, CCDC51, IDH3A, USP48, NCBP1, CTPS2, GLRX2, YWHAZ, TNFRSF1A, SLC25A17, ACOX2, RRM2B, CAT, PCCB, PFDN2, MTFR1, PFDN4, SLC25A13, DDX28, ACSS3, NDUFB4, C11H12ORF10, RAP1GDS1, GTPBP10, GLRX, MTIF2, MAT2B, PDHB, HSD17B10, UACA, MRM1, TBRG4, SLC25A27, CECR5, OXCT1, ROMO1, APOPT1, ABCF2, PTCD3, CYB5A, MMAB, CCDC58, MACC1, SOD1, GSTZ1, GOLPH3, PINK1, SLC25A39, SUCLA2, CAPN10, OCIAD1, BAX, SLIRP

GOTERM_CC_DIRECT	GO:0016020	membrane	230	2.42E-12	6.39E-10	EIF4A1, CMAS, ECI2, HNRNPU, EPRS, RPL7, RPS14, PSMD7, RPS19, SCP2, PSMD2, PSMD3, TNFSF10, RPL35, RPS11, RPS13, CDK5RAP3, RPS12, CHID1, DDX18, ATP6V0E1, RPL23, ECH1, COG3, MED4, EML4, MYL6, DDX39A, ZW10, PSME3, XRN2, PSME2, ORAI1, YME1L1, PFN1, ANKRA2, RPL28, CUTA, DHX9, NMD3, MST1R, HNRNPLL, FXR1, UGCG, PDGFC, NEU1, XRCC6, PPP1R21, EDEM1, EDEM2, PARP14, PRDX6, HNRNPL, AIMP1, HNRNPM, HNRNPK, FUBP3, CALU, MAGED2, PAM, RPS23, AARS, OGFR, HBS1L, FKBP15, CCDC126, RPLP0, PRKAG1, OLA1, MED15, COIL, LFNG, SYNCRIP, RARS, C1QBP, NUF2, SDF4, BBS7, JAK2, HRAS, CTSC, HTATIP2, OFD1, SREBF1, SERPINB1, SLC39A11, ANO8, LRRC40, RRAS2, PRPF40A, JOSD1, GNL3, MLF2, RAP2A, RAP2B, HNRNPH1, TMEM106C, AIDA, ALDOA, ERGIC2, SLU7, EXOC2, GCNT1, RPL11, DLST, STX10, ARFRP1, NRAS, PGRMC2, PSMB2, MGAT3, ATP6V0E2, UBL4A, NPM1, PLEKHA1, MGMT, RAB11A, LRPPRC, PSMC5, DHRS7, PSMC4, CAPZA2, SERBP1, HNRNPA2B1, PNPLA3, NUCB1, PNPLA2, MTCH1, DENND5B, FMR1, CSE1L, ARHGDIG, CPOX, RPL10A, IPO7, HID1, CXCL16, EDC4, FBL, LAPTM4A, APMAP, CFL1, TMEM38B, GTF2I, CAST, SH3GLB1, UNC5CL, RPS9, RPS7, RPS5, SLC30A5, TSC2, SDHC, WRNIP1, LARP4B, GEM, RNF40, RAB32, ILF3, ILVBL, EHBP1L1, PPIB, FTL, ARL8B, DDX5, PSMD12, TUBGCP2, DDX1, NTPCR, RAP1B, EFNB1, ATXN2, RAP1A, UBR5, GRK6, PMVK, KDSR, SDF2L1, CDK9, CNOT7, RHEB, CNOT2, IARS, DYNLRB1, TMEM19, MAP3K11, GALK1, LGALS3BP, CELF1, YWHAB, CALCOCO2, CCDC47, DDX42, NENF, DDX41, ELAVL1, SPRED2, CAPZB, YWHAQ, PSTPIP2, ACTR3, DIS3, DDX56, PPP2R5A, DDX54, TAPBP, EEF1G, SFT2D1, MVP, IL2RG, GANAB, CAND1, VPS51, POLR2B, HNRNPA1, ABCF2, MTA2, B3GAT3, CDC5L, DNAJA1, NECAP2, EIF3L, MGAT4A, OCIAD1, DRG1, EIF3H, EIF3E, NAA15, EIF3D, RAN
GOTERM_CC_DIRECT	GO:0005925	focal adhesion	96	4.37E-12	8.67E-10	SCARB2, CYFIP1, NCKAP1, TES, CD81, YWHAB, USP33, ARPC1B, RPLP0, FHL2, ARPC5L, RPL10A, RND3, ACTB, RPL7, PPP1CC, RPS14, LASP1, ALCAM, YWHAQ, RPS19, PLAU, BSG, CFL1, FLOT1, ANXA6, CAPN2, CHP1, ITGAV, CPNE3, RAC1, CAPN1, RPS11, B2M, RPS13, ACTR3, PDIA3, RPS9, PDCD6IP, ANXA1, MME, RPS7, ITGA3, RPL23, RPS5, ANXA5, ADAM10, LIG4, RRAS2, YWHAZ, RHOA, MMP14, RPL37A, CAT, ADAM9, LAP3, PPIB, CD46, PFN1, ATP6V0C, GRB7, RALA, CD151, FBLIM1, TWF1, CORO1B, HSP90B1, DPP4, RAB21, EFNB2, NCSTN, FLRT3, EPB41L2, G3BP1, GNA12, CTNNA1, RPS3, CD59, CD99, MPZL1, PAK4, NPM1, GSN, JUP, HSPA5, SRP68, HNRNPK, ARPC2, RPS29, RFWD2, CTNNB1, CD9, SDC1, LAMTOR3, BCAR1
GOTERM_CC_DIRECT	GO:0005829	cytosol	217	1.36E-11	2.16E-09	VPS29, CDA, CMAS, ACTB, BAIAP2L2, AP1S1, ARFIP1, CPNE3, SNRPD3, ATP6V1E1, FBXO7, VPS36, GABARAPL2, GABARAPL1, PRKCD, AP1AR, GAPVD1, JMJD6, RNF126, CLNS1A, PSME4, ULK1, HPRT1, ANKRA2, VPS25, CFTR, GRB7, STXBP3, VPS26A, OAZ1, SNX3, SNX1, HSPH1, SNX2, AP5M1, PRDX4, PDCD10, LHPP, XRCC4, JUP, UCK2, FAM160A2, QARS, GIPC1, FBXL3, AARS, TOP2B, CLIC4, MOCS2, CDKN1A, ADK, WDR45, SH3RF1, AIFM2, C1QBP, ANXA7, MUC13, CTSH, DFFA, ANXA2, RIPK3, PRMT2, PGAM1, PRMT3, SIRT2, UCHL5, RHOA, VAMP8, RAP2C, UGDH, PLSCR1, AFTPH, RAP2A, RAP2B, STRAP, VAMP3, FH, RABGAP1, URM1, USO1, AKAP7, FAM129B, STX10, RELB, ARFRP1, FAM213B, CTNNA1, STX5, BID, LCMT1, PEX19, YTHDF2, GCH1, MDH1, SORT1, UBE2G2, ADAT3, CTNNB1, PNPLA2, UBXN2B, CSE1L, DGKA, CTNND1, PDCD5, PARK7, SAT1, IPO7, IPO8, COL4A3BP, CAPN2, KPNA4, CAPN1, KPNA2, CAST, G6PD, USP7, STX8, LARP4B, RBX1, TOM1L1, PGP, S100A6, SNRPE, SNRPF, GEMIN7, PPIA, ISCU, SNRPB, VCP, UBA5, IREB2, GMPS, ABHD5, CORO1B, HSP90B1, RAP1B, SHARPIN, G3BP1, PMVK, EIF4EBP1, DVL2, G3BP2, TBC1D14, PMAIP1, DVL3, NAE1, RAB6B, BCL2L14, MCL1, BCL2L15, TRAPPC3, OSBPL7, GOT1, HSPA4, VTA1, EPHX2, IDH1, IDH2, GLMP, ASNS, NFKBIA, COPS4, GCLC, UBA3, UBA2, ACO1, UBA1, IARS, MYD88, NFE2L2, SRXN1, ASNSD1, AMD1, NGLY1, OPLAH, ELAVL1, HK1, ALAD, PPP6C, PSTPIP2, NAPRT, PGM1, PDCD6IP, WIPI1, TRAF2, WIPI2, DNAJC1, PSRC1, PEX6, ATP6V1B2, BLVRB, MVD, DCTPP1, GAPDH, PDXK, SRC, VPS4B, RAP1GDS1, SRI, USP19, VPS51, MAT2A, TP53INP1, PDE6D, CTNNAL1, MAPK3, ARFGEF1, CNBP, MID1IP1, SOD1, DNAJA1, GOLPH3, RAB14, CAPN10, PDCD4, BAX, SPG21
GOTERM_CC_DIRECT	GO:0005743	mitochondrial inner membrane	57	3.67E-09	4.85E-07	SLC25A1, SLC25A3, ACAA2, ECI1, SQRDL, LGALS3, FDXR, MPV17, HADH, MCCC1, NDUFC2, ACSL5, UQCR, SDHA, SDHB, COX6B1, HADHB, ALDH3A2, HADHA, SLC25A15, TST, BDH1, CHDH, CSDE1, VDAC2, YME1L1, SUCLG1, SLC25A10, SLC25A5, SLC25A11, SLC25A4, SLC25A13, PAOX, MAOB, SRC, SHMT2, MGST1, AK2, PHB, COX5A, UQCRH, SAMM50, RPS3, SLC25A20, SLC25A22, SLC25A24, NDUFA8, NDUFA7, SURF1, IDH2, IMMT, SLC25A38, ABCB10, NDUFAF3, SFXN1, OCIAD2, OTC
GOTERM_CC_DIRECT	GO:0005840	ribosome	48	8.44E-09	9.56E-07	RPL4, RPS4Y2, RPL3, MRPS35, MRPS14, MRPL18, RPL12, MRPS33, RPL11, RPLP0, RPS27L, MRPL39, MRPS30, MRPL34, MRPL13, MRPL10, RPL7, MRPL55, MRPL33, MRPL4, RPL7A, RPS14, RPL35, RPL15, RPS11, RPS12, NCK1, MRPS27, RPS7, MRPS25, RPS5, RPL23, PRMT3, MRPS23, MRPL28, MRPS18A, MRPS21, MRPL46, MRPL24, MRPL44, RPS25, MRPL50, MRPL51, RPL27A, RPL22L1, FAU, RPS21

GOTERM_CC_DIRECT	GO:0005654	nucleoplasm	281	6.71E-08	6.65E-06	ECI2, HNRNPU, HNRNPR, SMC3, PSMD7, CCDC91, PPP4R2, SCP2, ZMIZ2, GPBP1, PSMD3, FBXO3, RCC1, CCNL2, SNRPD3, PDK2, MEF2A, CXADR, SFMBT1, COG3, NSMCE1, HCFC2, SCAMP2, JMJD6, DDX39B, CLNS1A, PSME3, YME1L1, UQCRC2, LAP3, AURKAIP1, ASF1B, SET, DHX9, CTBP1, MAPKAP1, NMD3, COPS7A, HTATSF1, CDKN2AIP, MRPL10, HSPH1, C7H14ORF166, EPB41L2, MIER1, MCMBP, LTA4H, RBM12, ZBED5, DECR1, LHPP, PRELID1, XRCC6, XRCC4, SIVA1, GADD45A, IRF2BP1, LARP7, TMEM192, UPF3A, NMI, HNRNPL, MSRA, RNPS1, GRB2, FBXL4, SLC2A4RG, TOP2B, DAZAP1, NKAP, ADK, AKR1B1, PHF6, ZMYM2, NKRF, RARS, HTATIP2, PBRM1, ANXA1, PRMT2, TFE3, VWA5A, SIRT6, PRPF40A, PPHLN1, MRPL46, ZHX1, GNL3, APRT, UGDH, AFTPH, PLCB3, RAD51C, PSMA1, BDH1, HNRNPH1, HNRNPH3, DLD, TOMM40, WDR26, AP5Z1, CAPG, CCNDBP1, TMEM70, FAM129B, RELB, PSMB6, WRN, PSMB5, PSMB2, PSMB3, STX5, CCT8, SF3B1, CCT4, NQO2, NPM1, PEX19, TIMMDC1, PLEKHA1, GCH1, CDX2, MGMT, TMUB1, NUDT22, NEDD1, RYBP, RFWD2, PSMC4, GID8, HNRNPA2B1, MPLKIP, CIAPIN1, FMR1, CSE1L, IPO7, ETS2, IPO5, EDC4, COL4A3BP, PAPOLA, SNIP1, KPNA4, SLC26A11, ZNF326, KPNA2, HADH, GTF2I, IPMK, ANXA13, LIG4, ZBTB33, WBP11, THOC5, RNF40, ILF3, MAPKAPK3, AAGAB, PPIG, STAM2, ABCG1, DDX5, VCP, RPS19BP1, TUBGCP2, CUL2, PHB, SEL1L3, FAM188A, ATXN2, UBR5, EIF4EBP1, PACSIN3, RHPN2, AKIRIN1, TIA1, FUS, FBXO34, COPS4, COPS6, C15H9ORF72, IBTK, CNOT7, UBA2, IARS, FGFR4, COPS8, COPE, CELF1, USP33, DDX42, NENF, NXT2, ELAVL1, MFSD8, SENP6, CASP8, OPA1, SIX5, SNRNP70, TIMM17A, HIF1AN, CYTIP, RBM5, CCDC51, TTC37, USP48, TGIF2, HMGCS1, CHUK, NCBP1, NCBP2, DIS3, PLA2G4C, PLRG1, GLRX2, AXIN2, WIPI2, SAFB, RAD23B, HAUS2, RRM2B, ZNF639, ESRP1, FAM96B, BLVRB, NDUFS2, LUC7L3, PFDN2, SNRPA1, MCM6, RBM22, DCTN5, PDXK, CEBPB, ACSS2, RBM48, HPGD, NDUFB6, TM2D1, USP10, NDUFB5, NDUFB4, C11H12ORF10, POMP, MTIF2, SRI, RNF7, PDHB, RCHY1, ADD3, FZR1, DNPEP, CAND1, MTA1, MKNK2, SNRPB2, PCBD1, BUB3, HNRNPA1, DCAF11, SLC25A22, MAPK3, ARFGEF1, ATF7IP, CYHR1, USP28, FBXL14, U2SURP, USF1, SOD1, GOLPH3, MYO1C, CDKN2AIPNL, EIF3L, TNIP2, ASXL2, PDCD4, CCDC174, CLUAP1, RAN
GOTERM_CC_DIRECT	GO:0005764	lysosome	42	2.92E-07	2.58E-05	SCARB2, ASAH1, SRC, CTSZ, PCSK9, CTSV, TMEM97, IFI30, CTSS, AP5M1, CTSL, CTSK, NEU1, CAPN2, CTSH, SLC38A9, CAPN1, DRAM2, VPS36, CTSD, CTSC, CTSB, CHID1, CD164, TMEM150B, GLMP, TSC2, TMEM59, C15H9ORF72, LMBRD1, RRAGC, NPC2, VMA21, KCNQ1, SNX14, NAAA, PPT1, GLA, ITM2C, ENGASE, LGMN, PCYOX1
GOTERM_CC_DIRECT	GO:0048471	perinuclear region of cytoplasm	86	3.48E-07	2.76E-05	ANKRD13C, ACHE, CYFIP1, CLIC4, TFRC, YWHAB, CALCOCO2, USP33, FMR1, HMGB2, PCSK9, NXT2, ATRAID, CLU, COL4A3BP, ANXA6, HIF1AN, ABCD1, CTSB, TPD52, DGAT2, STX8, ANXA4, PRKCD, SLC11A2, TSC2, WRNIP1, CSNK1D, SIRT2, VAMP8, PLA2G16, VAMP7, RAD51C, S100A6, PPIB, ZNF675, VAMP5, VAMP2, PAFAH1B1, VCP, SET, ANP32A, SRC, MVP, USO1, TWF1, NDRG1, CORO1B, STX10, HSP90B1, FXR1, CYB5R4, ATXN2, RAP1A, HECTD3, EIF4E, SEC23B, TSPAN1, ATP9B, RAB11FIP4, SEC31A, LYN, ARFGEF1, RAB4A, NDFIP2, NDFIP1, GALNT3, SORT1, AMFR, M6PR, TMEM192, RAB11A, LRPPRC, PUM2, EHD1, MYO1B, TF, EHD4, CTNNB1, SLIRP, ATXN10, FBXL5, COPS8, ITM2C, CDKN3, PICALM
GOTERM_CC_DIRECT	GO:0005783	endoplasmic reticulum	116	1.09E-06	7.85E-05	ANKRD13C, ZDHHC6, PARK7, EEF1B2, NSDHL, APMAP, ARL6IP5, SLC26A11, KIFAP3, SEC62, PDIA3, CAST, TPD52, BNIP3L, ALG9, PRKCD, SHISA5, ALG3, TPST2, ALDH3A2, PLA2G16, TMX3, RNF128, TMX1, KCNQ1, ZW10, KDELR2, CDS1, RTN4R, SET, ANP32A, SAR1B, YTHDC2, MGST3, MTTP, MGST2, SEZ6L2, APH1A, CYB5R4, NCSTN, SGPL1, PRDX4, SSR1, SLC38A9, KDSR, MPDU1, PRNP, FIS1, NDFIP2, TMEM50A, EDEM1, TMEM50B, SRD5A3, EDEM2, ERLIN1, HPS6, ERLIN2, FA2H, EHD4, TBL2, TMEM98, REEP4, CALU, EBPL, ACO1, FGFR4, MOGAT2, CCDC47, CTSZ, PCSK9, UFSP2, SNX10, DDX41, NKRF, CASP4, TMED2, UBXN4, NCK1, SSR2, IFNGR2, SUMF1, EEF1G, HADHB, DNAJC1, RCN1, RCN2, TMEM33, STX18, USO1, RAP1GDS1, ATL2, AGPAT2, AGPAT3, FAM213B, TMEM203, ASNA1, AGR2, AGR3, DNAJB9, BCAP31, SLC35A2, CNBP, BNIP3, TPBG, SRP68, UBE2G2, FMO5, KTN1, ARCN1, ERP44, TSPO2, LPCAT4, YIPF6, AQP11, PNPLA6, NUCB2
GOTERM_CC_DIRECT	GO:0071013	catalytic step 2 spliceosome	31	6.33E-06	3.98E-04	DDX5, SF3B3, SRSF1, HNRNPU, HNRNPR, DDX41, SYNCRIP, DHX35, CWC22, MAGOH, SNRPB2, RALY, SNRPD3, HNRNPA1, SF3B1, CDC5L, CWC15, PLRG1, CDC40, HNRNPM, PHF5A, HNRNPK, SYF2, HNRNPH1, HNRNPA2B1, FRG1, SNRPA1, SNRPE, SNRPF, SLU7, SNRPB
GOTERM_CC_DIRECT	GO:0005765	lysosomal membrane	45	6.52E-06	3.98E-04	SCARB2, SCARB1, TMEM55B, RAB5C, HM13, TCIRG1, PLD1, ATRAID, TMEM165, DPP4, MFSD8, AP3M1, NCSTN, LAMP2, FLOT1, ANXA6, ATP6V1H, SLC26A11, ATP6V1E1, ATP6V1D, ATP6V1C1, TMEM8A, NSF, RAB2A, ATP6V1G1, ANXA2, STARD3NL, STK11IP, GAA, SLC11A2, TMEM192, DNAJC13, MFSD12, WDR11, RAB14, VPS41, TMBIM1, GNB1, ATP6V1B2, TMEM106B, LAMTOR2, UBA1, ATP6V0C, ARL8A, TECPR1
GOTERM_CC_DIRECT	GO:0005730	nucleolus	131	1.11E-05	6.26E-04	FOXA1, ZNF330, FMR1, RPL10A, TTF1, RPL7, PPP1CC, RPS14, RPS19, ZNF207, RPS11, CHCHD1, RPS13, TSPYL2, DDX18, RPS9, COG7, RPS7, RPL23, SLC30A5, IPMK, NGDN, ACSL5, DNTTIP2, LARP4B, ZBTB33, FBXO11, THAP2, JMJD6, CD2AP, DHX40, EBNA1BP2, ILF3, C8H8ORF4, EWSR1, TMX1, XRN2, SRSF5, VPS25, ZCCHC7, DDX5, BLM, TSG101, RPS19BP1, DHX9, CUL2, MRI1, NMD3, RTF1, CDKN2AIP, RPF1, MBIP, H1FX, LLPH, LYAR, ZNF146, ZC3H15, SMARCA5, RPS25, LSM6, CRYL1, GRB2, RSL24D1, CUTC, RPS23, SETD4, SLC25A3, SRP54, OLA1, HMGB2, PIK3CB, CTSV, PHF6, COIL, SUMO1, NKRF, RAB8A, CTSB, H2AFY, EED, NSUN2, DDX56, DDX54, PLRG1, DDX50, GLTSCR2, GNL3, PLSCR1, NSA2, RCN2, FRG1, AGPS, PFDN2, ERGIC2, PAFAH1B2, DDX28, CSTB, GTF3C3, SF3B3, USO1, RPL11, TIMM13, NOL7, CAPG, ARNTL2, FAM129B, WRN, RBBP5, SAP30L, ASNA1, MKNK2, RPS3, SNRPB2, CCT5, ARFGEF1, NPM1, PCGF5, CRIPT, CDC5L, APEX2, HN1, NPM3, USP28, SMUG1, NEDD1, CDKN2AIPNL, KATNBL1, TCEA1, CIAPIN1, RAN, BCAR1
GOTERM_CC_DIRECT	GO:0030529	intracellular ribonucleoprotein complex	30	1.92E-05	0.001013	DHX9, HNRNPU, HNRNPR, EPRS, HNRNPLL, SYNCRIP, XPO1, RAVER1, SNRNP70, G3BP1, G3BP2, HNRNPA1, NPM1, HNRNPA3, SSB, NCBP1, LARP7, LARP4B, LRPPRC, JMJD6, HNRNPL, HNRNPM, ILF3, HNRNPK, HNRNPH1, HNRNPA2B1, HNRNPH3, SLIRP, GAPDH, SNRPB

GOTERM_CC_DIRECT	GO:0005789	endoplasmic reticulum membrane	62	3.87E-05	0.001917	FKBP11, ABCD4, CISD2, MIA3, HERPUD1, UFL1, ANXA7, TMED1, ELOVL1, ALG8, JAGN1, ALG9, DGAT1, SSR2, MOGS, SLC35G1, ELOVL7, TMEM170A, PIGC, CYP39A1, DPM2, POR, TMX3, SLC9A6, SOAT1, SOAT2, TMX1, FKBP8, KDELR2, SEC22B, SLC27A4, CDS1, RAB1A, TMED10, INSIG1, SRI, HMGCR, MOSPD3, RTN4, LMF2, USP19, HSP90B1, LMAN1, NCLN, SSR1, SEC11A, SEC11C, BCAP31, SDF2L1, ICMT, ERLIN1, ERLIN2, FMO4, FMO5, FKBP1A, FA2H, ERP44, VAPB, FXYD3, VMA21, BAX, SERINC1
GOTERM_CC_DIRECT	GO:0005811	lipid particle	20	4.69E-05	0.002189	BCAP31, VCP, DGAT2, RAB5C, ANXA2, ACSL4, ABHD5, HSD17B11, CIDEC, RAP1B, REPIN1, EHD1, NSDHL, SCCPDH, AIFM2, PNPLA3, LDAH, GAPDH, RAB3GAP1, PNPLA2
GOTERM_CC_DIRECT	GO:0043209	myelin sheath	37	2.07E-04	0.009125	NAPA, NAPB, VCP, RALA, SLC25A3, GDI1, NDUFA10, DLST, ATP5C1, ENO2, NDRG1, COX5A, ACTB, TUBA1B, RAP1A, FAM213B, GLUL, CCT5, IDH3A, NSF, PDIA3, CCT2, PDCD6IP, HSPA5, MDH1, IMMT, SOD1, EHD1, GNB1, ATP6V1B2, UQCRC1, FSCN1, VDAC2, UQCRC2, SLC25A5, DLD, SLC25A4
GOTERM_CC_DIRECT	GO:0005885	Arp2/3 protein complex	8	2.70E-04	0.011275	ACTR3, ARPC2, ARPC3, ARPC1B, ARPC1A, ARPC5L, ARPC4, ARPC5
GOTERM_CC_DIRECT	GO:0005635	nuclear envelope	23	4.48E-04	0.017746	BNIP3L, SUN1, MGST3, CSE1L, BNIP3, PLA2G4C, SIGMAR1, SHISA5, MGST2, TMEM170A, ATRAID, IPO7, RTN4, IPO8, MTA1, S100A6, BAX, ANXA7, TERF2IP, CHMP7, MAPK3, PAFAH1B1, HTATIP2
GOTERM_CC_DIRECT	GO:0030496	midbody	28	5.63E-04	0.021276	ZNF330, CLIC4, SDCCAG3, RALB, TXNDC9, GDI1, CTNND1, HSP90B1, ECT2, RAB11FIP4, ANXA2, HSPA5, C4H6ORF89, VPS37B, SIRT2, GEM, KLHL9, PSRC1, SCCPDH, JTB, PIN1, MPLKIP, MAPRE3, ERH, MITD1, RAN, ARL8A, ARL8B
GOTERM_CC_DIRECT	GO:0005852	eukaryotic translation initiation factor 3 complex	9	6.94E-04	0.025019	EIF3M, DDX3X, EIF3L, EIF3I, EIF3J, EIF3H, EIF3E, EIF3F, EIF3D
GOTERM_CC_DIRECT	GO:0005681	spliceosomal complex	17	9.94E-04	0.034287	WBP4, U2AF1, CWC15, WBP11, DDX39A, LSM6, DDX39B, RHEB, DHX35, DHX15, SNRPE, ZNF326, SNRPF, HNRNPA1, TXNL4A, SLU7, RBM5
GOTERM_CC_DIRECT	GO:0019013	viral nucleocapsid	21	0.001241	0.040989	HNRNPA3, HNRNPU, HNRNPR, LARP4B, HNRNPLL, HNRNPL, HNRNPM, SYNCRIP, HNRNPK, RAVER1, SNRNP70, HNRNPH1, HNRNPA2B1, SNRPE, SNRPB2, SNRPA1, HNRNPH3, SNRPD3, SNRPF, HNRNPA1, SNRPB
GOTERM_CC_DIRECT	GO:0022627	cytosolic small ribosomal subunit	23	0.001409	0.044681	MCTS1, RPS9, DDX3X, RPS7, RPS8, RPS5, RPS27L, MRPS18A, RPS17L, RPS15, RPS26, RPS25, RPS14, RPS19, RPS29, RPS3, EIF2D, FAU, RPS11, RPS21, RPS13, RPS23, RPS12
GOTERM_CC_DIRECT	GO:0005839	proteasome core complex	8	0.002421	-	PSMB6, PSMB4, PSMA4, PSMB5, PSMB2, PSMA1, PSMB1, PSMB8
GOTERM_CC_DIRECT	GO:0016282	eukaryotic 43S preinitiation complex	8	0.002421	-	EIF3M, EIF3L, EIF3I, EIF3J, EIF3H, EIF3E, EIF3F, EIF3D
GOTERM_CC_DIRECT	GO:0033290	eukaryotic 48S preinitiation complex	8	0.002421	-	EIF3M, EIF3L, EIF3I, EIF3J, EIF3H, EIF3E, EIF3F, EIF3D
GOTERM_CC_DIRECT	GO:0000502	proteasome complex	12	0.002638	-	PSMD10, VCP, PSMD7, PSMD2, PSME3, SHFM1, PSMD3, POMP, PSME4, PSME1, PSMG3, PSME2
GOTERM_CC_DIRECT	GO:0005747	mitochondrial respiratory chain complex I	15	0.002994	-	NDUFB9, NDUFA8, NDUFB7, NDUFB6, NDUFA12, NDUFB5, NDUFB4, NDUFA10, NDUFB1, FOXRED1, NDUFS8, NDUFS7, NDUFS4, NDUFS2, NDUFV1
GOTERM_CC_DIRECT	GO:0031965	nuclear membrane	34	0.003008	-	DCTN5, GTF3C3, AHCTF1, MRPS14, NDUFB4, YBX1, TMEM97, APEH, IPO5, REPIN1, FAM188A, FZR1, XPO1, SUMO1, RCC1, EPC1, TSPO, TNPO2, RANBP2, OSBPL8, AKIRIN1, NUP133, GCH1, ANXA4, MRPS23, HN1, PLRG1, RANBP6, PUM2, GCHFR, TMEM57, FAM76B, GAPDH, PAFAH1B1
GOTERM_CC_DIRECT	GO:0055038	recycling endosome membrane	10	0.004287	-	RAP2C, RAP2A, RAP2B, EHD4, SLC9A6, NDRG1, RAB11FIP4, RAB8A, SCAMP2, RAB11B
GOTERM_CC_DIRECT	GO:0016272	prefoldin complex	6	0.004648	-	VBP1, PFDN1, PFDN2, PFDN4, PFDN5, PDRG1
GOTERM_CC_DIRECT	GO:0010494	cytoplasmic stress granule	11	0.004792	-	TIAL1, GRB7, TIA1, ATXN2, DDX3X, PABPC4, DDX1, LARP4B, YBX1, EIF4E, PUM2
GOTERM_CC_DIRECT	GO:0030176	integral component of endoplasmic reticulum membrane	20	0.005749	-	SLC35B3, DGAT2, HSPA5, RHBDD1, EDEM1, AMFR, SLC35B1, DERL1, TEX261, RTN4, TM7SF2, TMCO1, DPM2, TMEM33, DOLPP1, ACER3, FKBP8, ESYT1, PIGG, SLC27A2

GOTERM_CC_DIRECT	GO:0005737	cytoplasm	479	0.005943	-	TES, SPINT2, EPRS, SMC3, GLE1, TBK1, PSMD3, EARS2, CDK5RAP3, SULT2A1, MEF2A, TSPYL2, CXADR, HCFC2, CD2AP, ACE2, RNF126, DDX39A, DDX39B, PRKAR1A, TEP1, PSME3, DDIT4, PSME1, TXNIP, PSME2, HPRT1, ATF4, ANAPC16, MAPKAP1, COPS7A, SDSL, OAZ1, RIC8A, LDHA, PPP4C, C7H14ORF166, FLRT3, EPB41L2, RDH10, PDGFC, MCMBP, RDH14, SFN, LTA4H, NRBF2, ZNF146, ATG5, MCTS1, GSTM4, JUP, PMM1, MAD2L1BP, UPF3A, ELL2, NR0B2, PRDX6, EIF1, EIF5, GJB3, TDP1, PKIA, NMT1, CPEB2, DZIP3, SMG7, IKBKB, UBL5, TUBA1B, TMSB4X, RARS, JAK2, PCMTD1, BRD2, LYPLA1, RING1, RIPK2, GSTO1, DUSP1, TESC, NSUN2, EMP2, HEXDC, DUSP6, SERPINB5, AFTPH, BDH2, PSMA1, CDC42EP4, MTF2, ADSSL1, CDC42EP2, PICK1, ERGIC2, ARF5, PAFAH1B2, PAFAH1B1, WDR26, OAT, LRRC19, ASCL2, TANK, SSFA2, PSMB6, PSMB4, WRN, PSMB5, FAM213B, PSMB2, PSMB1, METTL7B, HARS, PAIP2, BID, BRD7, GLYCTK, MBD2, PSMB8, PSMC5, RFWD2, ABI1, SERBP1, CIAPIN1, FRK, CDKN3, FMR1, UBE2D1, MSI2, APEH, FBXO22, IPO5, NPEPPS, SLK, MAEA, RNF19A, CFL1, PIM1, PAPOLA, PLS3, KPNA4, PIM3, KPNA2, PLS1, GTF2I, MAP2K3, SH3GLB1, SH3GLB2, CSNK1G3, MAP2K4, FBXW5, RALGAPA1, ZBTB33, VRK3, SDCBP2, FBXO11, TUBA4A, THOC7, ACLY, MAPKAPK3, FSCN1, AAGAB, SRSF5, FTL, ARL8B, SNRPB, RTN4R, BLM, TXNDC9, TUBGCP2, IREB2, CREM, STK39, MRI1, ABHD5, LIN7C, GTF2E2, ESD, UBR5, EIF4EBP1, MBIP, SMN1, FUK, SOCS5, NEK9, FUS, RITA1, FBXO34, ARPC4, LRRC66, COPRS, RCAN1, IBTK, ARPC3, TUBGCP5, RNF182, TPP2, IARS, MNAT1, MYD88, NFE2L2, CALCOCO1, CELF1, RNF14, PIK3C2A, CAPZB, UPP1, TNPO2, EIF5A, HMGCS1, CHUK, VBP1, AHSA1, KCNAB2, RAD23B, ADRA2A, ARL4C, NT5C3B, TRAF4, ETF1, EIF4E2, BIRC2, CEBPB, ACSS2, USH1C, SRI, RCHY1, HSD17B11, MTHFSD, SH3BP1, HECTD3, TRIM47, PCBD1, HNRNPA1, EIF4E, ADSS, HN1L, MACC1, NAGA, CDC5L, MYO1B, PFKL, DBNDD2, MYO1C, PTPN9, CCM2, BCAR1, EIF4A1, PANK3, LPGAT1, FAM89B, PARS2, NUDT3, NUBP2, RPS6KA3, HERC3, RASSF2, RASSF3, RPS6KA1, DHX58, FBXO3, RCC1, ARFIP1, FBXO9, RSAD1, IFRD1, TALDO1, CSNK1D, ANK3, DHX40, PPA1, CSNK2B, KCTD11, LAP3, PFN1, TRIB1, PFN2, BEX4, DHX9, STC1, NMD3, CD1D, UBE2J2, FXR1, DHX30, DHX35, STK38L, ZBED5, CDR2, GADD45A, KLHDC8B, NMI, PARP14, DERA, CCT6A, FUBP3, CDC16, SPATS2L, SDC1, RNPS1, CUTC, AARS, AHCTF1, DAZAP1, PTPRR, ACY1, GDI1, NKAP, OLA1, PCSK9, AKR1B1, KRT20, KHK, XPO1, ZMYM2, NUDCD2, PCF11, ENC1, DHX15, KIF13B, BBS7, CLIC1, HTATIP2, APOBEC1, CCT2, SREBF1, EDN1, MME, TSC22D1, TSC22D3, PABPC4, STRADB, TFE3, PRPF40A, UCHL3, UCHL5, APRT, PITPNM1, HNRNPH1, PABPC3, TAX1BP3, MAPRE3, AIDA, SLU7, CSTB, ADH1A, CRIP1, ARNTL2, RELB, PPP2CB, PAK1, SERTAD1, CCT8, MAP4K5, PAK4, CCT4, NQO1, NQO2, NPM1, PLEKHA1, GCH1, TMUB1, UBE2G1, UBE2G2, RANBP6, HNRNPA2B1, PNPLA3, CTNNB1, RAB3GAP1, PNPLA2, ENGASE, CARS, UBE3C, ARHGDIG, TTF1, ETS2, NARS, PTTG1, ARHGDIA, CAPN2, SNIP1, KNTC1, CAPN1, AZI2, TRAPPC2L, LIG4, KCTD2, WBP11, EEF1A1, KCTD9, C8H8ORF4, TIPRL, UBE2V2, PPIH, UBE2V1, PPIG, STAM2, PSMD10, ZCCHC7, PSMD12, NDRG3, RPS19BP1, YTHDC2, NDRG1, STK3, SDCBP, EFNB1, RPRD1B, RHPN2, ECT2, ZC3H15, KLHDC3, SMAD4, UBE2I, UBE2B, TBCC, KLHL22, UBE2A, SSH3, BLMH, UBE2K, GALK2, GALK1, UBE2M, MAST2, NGLY1, DDX42, HMGB2, CASP9, POLB, SENP6, CASP7, LASP1, CASP8, YWHAQ, CASP3, YWHAH, USP47, NCBP2, OXSR1, GPCPD1, EEF1G, ALDH1A3, UBE2R2, IRF3, RASA2, FAM96B, TERF2IP, SPOPL, PFDN4, PFDN5, RBM22, DDC, USP15, DCTN2, HPGD, TUBAL3, MVP, POMP, RNF8, RNF7, TOB2, RNF4, TOB1, HSPD1, MTA1, DNPEP, MKNK2, EPS8L2, ATF7IP, ARID3C, USP28, SOD1, UFM1, TNIP2, CAPN10, ERCC1, RPS6KB2,
GOTERM_CC_DIRECT	GO:0005689	U12-type spliceosomal complex	11	0.006639	-	SF3B5, SF3B3, PHF5A, PDCD7, DHX15, SNRPE, YBX1, SNRPD3, SNRPF, SF3B1, SNRPB
GOTERM_CC_DIRECT	GO:0005665	DNA-directed RNA polymerase II, core complex	8	0.006667	-	URI1, POLR2B, POLR2D, POLR2E, ZNF768, POLR2G, POLR2K, POLR2L
GOTERM_CC_DIRECT	GO:0012506	vesicle membrane	8	0.006667	-	RAB21, BAIAP2L2, GIPC1, ANXA4, GRB2, ATP13A2, TRAF2, NCK1
GOTERM_CC_DIRECT	GO:0070937	CRD-mediated mRNA stability complex	5	0.008766	-	SYNCRIP, DHX9, CSDE1, HNRNPU, YBX1
GOTERM_CC_DIRECT	GO:0071541	eukaryotic translation initiation factor 3 complex, eIF3m	5	0.008766	-	EIF3M, EIF3I, EIF3H, EIF3F, EIF3D
GOTERM_CC_DIRECT	GO:0043234	protein complex	38	0.008844	-	SET, TES, MAGED1, HMGB2, CISD2, CLU, STK3, POLB, UFL1, PAK1, ZNRF2, RASSF2, YWHAQ, SCP2, EPS8L2, CDK5RAP3, SH3GLB1, FIS1, PRELID1, CXADR, PEX19, GCH1, RIPK2, ERLIN1, ERLIN2, WIPI2, SLC51B, USP28, RAB11A, SOD1, PLCB3, SPATS2L, ORAI1, CCM2, SUGT1, SSX2IP, RAB3GAP1, ASF1B
GOTERM_CC_DIRECT	GO:0033179	proton-transporting V-type ATPase, V0 domain	6	0.009024	-	ATP6V0B, ATP6V0E1, TCIRG1, ATP6V0D1, ATP6V0E2, ATP6V0C
GOTERM_CC_DIRECT	GO:0035770	ribonucleoprotein granule	6	0.009024	-	FXR1, DDX28, HNRNPL, DHX30, TDRD7, GRSF1
GOTERM_CC_DIRECT	GO:0005770	late endosome	21	0.010601	-	CHID1, TSG101, SLC31A1, SRC, STX8, F2R, DERL1, M6PR, TMEM192, PCSK9, TMEM59, TF, AP5M1, RNF128, SLC9A6, KCNQ1, SNX14, VPS41, SDF4, VAMP5, LGMN
GOTERM_CC_DIRECT	GO:0005741	mitochondrial outer membrane	17	0.010659	-	BNIP3L, TOMM40, MAOB, MTX2, BNIP3, CISD1, CISD2, HADHB, PPP1CC, OPA1, AIFM2, MFN1, VDAC3, VDAC2, BAX, VDAC1, MCL1
GOTERM_CC_DIRECT	GO:0005903	brush border	14	0.01069	-	SLC15A1, MME, DCXR, USH1C, ADD3, MYL12B, MYO1B, PDZD3, MYL6, MYO1C, CAPZB, SOAT2, CAPZA2, PLS1
GOTERM_CC_DIRECT	GO:0030139	endocytic vesicle	11	0.015546	-	DPP4, EHD1, RAB5B, OCLN, TF, RALA, RAB5C, SH3KBP1, RAB13, PLD1, RAB11FIP4
GOTERM_CC_DIRECT	GO:0036513	Derlin-1 retrotranslocation complex	6	0.015588	-	VCP, HM13, AMFR, DERL1, SVIP, RNF5
GOTERM_CC_DIRECT	GO:0034709	methylosome	6	0.015588	-	CLNS1A, SNRPE, ERH, SNRPD3, SNRPF, SNRPB
GOTERM_CC_DIRECT	GO:0032592	integral component of mitochondrial membrane	4	0.016303	-	TOMM40, ABCB10, BID, TMEM70
GOTERM_CC_DIRECT	GO:0031461	cullin-RING ubiquitin ligase complex	5	0.01776	-	CUL4A, CAND1, CUL3, CUL2, CUL1
GOTERM_CC_DIRECT	GO:0005832	chaperonin-containing T-complex	5	0.01776	-	CCT6A, CCT2, CCT8, CCT5, CCT4
GOTERM_CC_DIRECT	GO:0031201	SNARE complex	15	0.01838	-	NAPA, NAPB, STX8, STX18, STX10, VAMP8, SNX4, VAMP7, STX5, SEC22B, VAMP4, VAMP5, YKT6, VAMP2, VAMP3
GOTERM_CC_DIRECT	GO:0005777	peroxisome	16	0.020104	-	ABCD4, PECR, IDI1, PHYH, PEX19, IDH1, EPHX2, IDH2, SOD1, AMACR, ACOX2, SCP2, PEX6, CAT, PMVK, FAR1

GOTERM_CC_DIRECT	GO:0005794	Golgi apparatus	96	0.020844	-	ARF3, ACHE, USP33, PCSK9, GCC1, MANEA, ZDHHC4, IPO5, GOLGA2, ING2, COL4A3BP, LAPTM4A, CCDC91, MECOM, SIX5, NAPRT, CWC22, CAPN2, TMED2, AP1S1, TMED1, SLC26A11, TOPBP1, FAM3C, KIFAP3, HRAS, B2M, RAB2A, MMD, GLCE, SLC30A5, SACM1L, SLC35C1, SLC39A11, TSC2, AP1AR, CSNK1D, PPHLN1, AXIN2, HAUS2, TPST2, BACE2, PLSCR1, RNF128, GORASP2, CSDE1, TMEM5, TMBIM1, VAMP4, MAPRE1, EAPP, ABCG1, SAR1B, MAPKAP1, SLC1A5, PLD1, PTGS1, PSMB6, APH1A, PAK1, NCSTN, NRAS, CAND1, PSMB3, PDCD10, CTNNA1, DYM, CDIPT, DRAM2, RABAC1, RAB6B, LYN, PRNP, NDFIP2, NDFIP1, B3GAT3, SORT1, LARP7, ABCA7, SLC50A1, FBXL14, RAB14, ARPC2, VAPB, PPT1, TAF7, NUCB1, MPLKIP, CRYL1, ACO1, LZTR1, SERINC3, FGFR4, GLA, RAB3GAP1, ITM2C
GOTERM_CC_DIRECT	GO:0014704	intercalated disc	9	0.021163	-	CAMK2D, CXADR, JUP, TMEM65, CAPZB, CTNNA1, DSG2, ANK3, VAMP5
GOTERM_CC_DIRECT	GO:0005686	U2 snRNP	9	0.021163	-	SF3B5, PHF5A, SNRPE, SNRPB2, SNRPA1, SNRPD3, HTATSF1, SF3B1, SNRPB
GOTERM_CC_DIRECT	GO:0005758	mitochondrial intermembrane space	12	0.023428	-	NDUFA8, COX19, PRELID2, PRELID1, NDUFB7, PNPT1, OPA1, AK2, CIAPIN1, MICU2, FBXL4, COX6B1
GOTERM_CC_DIRECT	GO:0016363	nuclear matrix	14	0.02418	-	CEBPB, BLM, AHCTF1, CLIC4, ANP32A, PRKCD, PRPF40A, SMC3, HNRNPM, PHF5A, MAEA, TEP1, CHMP1A, ZNF326
GOTERM_CC_DIRECT	GO:0015934	large ribosomal subunit	6	0.024702	-	RPL27A, RPL13A, RPL26, MRPL15, RPL10A, MRPL22
GOTERM_CC_DIRECT	GO:0008180	COP9 signalosome	10	0.026633	-	COPS4, COPS6, HSPA5, EPB41L2, COPS2, ATP5A1, FLOT1, GRB2, COPS7A, COPS8
GOTERM_CC_DIRECT	GO:0030134	ER to Golgi transport vesicle	7	0.027631	-	GOLGA2, LMAN1, YIPF6, PCSK9, KLHL12, TEX261, SEC31A
GOTERM_CC_DIRECT	GO:0031902	late endosome membrane	8	0.028335	-	VAMP8, TMEM55B, ANXA2, VPS41, SLC11A2, LAMP2, ANXA6, VPS36
GOTERM_CC_DIRECT	GO:0005769	early endosome	30	0.030741	-	SDCCAG3, RAB5B, TSG101, RAB5C, USP10, DERL1, PCSK9, SNX13, VPS26A, PHB, RAB22A, RAB21, NEURL1B, CHMP1A, VPS8, FLOT1, PTPN1, ANXA2, SORT1, STX8, F2R, SLC11A2, RAB32, EHD1, MYO1B, TF, RAB14, KCNQ1, VPS41, CFTR
GOTERM_CC_DIRECT	GO:0034663	endoplasmic reticulum chaperone complex	5	0.030872	-	SDF2L1, HSPA5, DNAJC10, PPIB, HSP90B1
GOTERM_CC_DIRECT	GO:0000139	Golgi membrane	31	0.033681	-	RAB1A, NAA60, TMED10, CUL3, COPB1, USO1, KLHL12, LMAN1, GOLGA5, STX5, GALNT7, BCAP31, SLC35A2, ARFGEF1, TRAPPC3, GABARAPL2, SLC35A4, SLC35A3, GALNT3, C4H6ORF89, B3GALT6, SCAMP3, TNFRSF1A, GOLPH3, B3GNT7, VAPB, B3GNT5, B3GNT3, B3GNT2, SEC22B, BET1
GOTERM_CC_DIRECT	GO:0005802	trans-Golgi network	24	0.034226	-	ARFGEF1, CHID1, SCOC, STX8, GCNT1, M6PR, DPY30, WIPI1, RAB11A, STX10, RAB21, RAB32, ARFRP1, GOLPH3, ATXN2, VAMP7, RAB14, RAB13, YIPF6, SNX9, RAC1, ATP9B, VAMP2, NCK1
GOTERM_CC_DIRECT	GO:0005768	endosome	28	0.034281	-	VPS29, SCOC, VPS26A, SNX10, PLD1, SNX3, SNX4, FLOT1, PACSIN3, SNX9, ATP6V1E1, ATP9B, RAB8A, ARRDC4, CD164, RAB4A, ATP6V0B, ANXA1, ARRDC3, AP1AR, RHOA, TOM1L1, C15H9ORF72, ARPC2, CDK2, VOPP1, GRB2, KCNK1
GOTERM_CC_DIRECT	GO:0042645	mitochondrial nucleoid	12	0.034973	-	DDX28, HADHB, DHX30, HADHA, POLDIP2, SHMT2, DBT, VDAC2, SSBP1, SLC25A5, GRSF1, LRPPRC
GOTERM_CC_DIRECT	GO:0008541	proteasome regulatory particle, lid subcomplex	4	0.035704	-	PSMD12, PSMD14, SHFM1, PSMD3
GOTERM_CC_DIRECT	GO:0005868	cytoplasmic dynein complex	6	0.036634	-	DYNC1LI1, DYNC1I2, SNX4, DYNLT3, DYNLRB2, DYNLRB1
GOTERM_CC_DIRECT	GO:0045121	membrane raft	24	0.038259	-	LYN, PRNP, CXADR, ANXA2, EMP2, TRAF2, ERLIN2, ARID3C, TNFRSF1B, HK1, TNFRSF1A, SDCBP, EFNB1, PLSCR1, RAP2B, MYO1C, KCNQ1, PPT1, CAPN2, FLOT1, PGK1, MAL2, CTSD, S100A10
GOTERM_CC_DIRECT	GO:0016328	lateral plasma membrane	11	0.038281	-	ANXA1, CLDN3, MYO1C, CLDN12, RAB13, CLDN7, TACSTD2, CTNNB1, ANK3, CLDN1, MARK2
GOTERM_CC_DIRECT	GO:0055037	recycling endosome	17	0.039445	-	SDCCAG3, TFRC, SLC31A1, STX8, SLC11A2, RAB11A, RAB11B, VAMP8, TF, RAB14, RAB13, TBC1D14, DENND6A, CFTR, ABCG1, RAN, VAMP3
GOTERM_CC_DIRECT	GO:0005844	polysome	10	0.041718	-	FXR1, NAA30, ATXN2, VBP1, FMR1, DRG1, RPS3, NAA38, MSI2, RPL7
GOTERM_CC_DIRECT	GO:0005685	U1 snRNP	8	0.048349	-	PRPF39, SNRNP70, LUC7L3, SNRPE, PRPF40A, SNRPD3, SNRPF, SNRPB
GOTERM_CC_DIRECT	GO:0036464	cytoplasmic ribonucleoprotein granule	8	0.048349	-	DDX3X, DHX9, FMR1, RPLP0, HNRNPU, RAC1, RPL28, ACTB
GOTERM_CC_DIRECT	GO:0005845	mRNA cap binding complex	5	0.048351	-	CYFIP1, NCBP1, NCBP2, EIF4E2, EIF4E
