# Supplementary material for: Identification, Characterization, and Transcriptional Reprogramming of Epithelial Stem Cells and Intestinal Enteroids in Simian Immunodeficiency Virus Infected Rhesus Macaques
Source: Front Immunol. 2021 Nov 23;12:769990. doi: 10.3389/fimmu.2021.769990 (PMC8650114; doi:10.3389/fimmu.2021.769990)
Supplement: Supplementary file 17 [file Table_11.pdf]

Supplementary Table 11: The 45 significantly enriched GO terms in the Biological Processes Category among upregulated DEGs

Category	GO:number	Term	Count	PValue	FDR	Genes
GOTERM_BP_DIRECT	GO:0086010	membrane depolarization during action potential	12	1.53E-04	-	KCNH2, CACNA1I, SCN10A, SCN8A, SCN9A, CACNA1B, CACNA1A, CACNA1C, CACNA1S, SCN3A, CACNA1E, SCN1A
GOTERM_BP_DIRECT	GO:0007165	signal transduction	60	0.001158904	-	WISP3, ARR3, ARHGAP6, WISP1, CYR61, SYDE1, SYNGAP1, SIT1, SUFU, TAGAP, SOX9, PDE8B, IKBKE, OR51G2, RALBP1, PDE4D, THOC1, PPP2R5D, ANK2, IL17RD, TICAM1, ANK1, TIRAP, INPP4A, RASA1, TRAF5, AKAP9, RIN3, PDE1B, IGSF1, CHRNA7, CABP4, TRHDE, RASIP1, CHRND, CHRNG, CHRNE, CASKIN1, SLIT1, TNFRSF17, PDE6C, HIVEP3, HIVEP2, PDE6A, SH2B3, RREB1, SRGAP1, CAP2, MYO10, ARHGAP23, ARHGAP32, RPS6KB1, PPP1R1C, NOSTRIN, SARM1, GNB3, CAMK1, PDE7B, PKN1, PDE7A
GOTERM_BP_DIRECT	GO:0000122	negative regulation of transcription from RNA polymerase II promoter	64	0.001459936	-	ZNF496, EHMT2, TFCP2L1, PRDM1, SLA2, NR2E3, ELK4, SIN3B, KAT5, SUFU, SOX9, SOX6, HIST1H1B, LMO1, PIAS4, MED1, ZHX2, RBM15, KDM2B, USP2, PAWR, HFE2, FOXP3, POU5F1, HIC1, ETV6, FOXP1, PIAS1, ZEB2, NCOR1, DDIT3, TIMELESS, RARB, TAGLN3, HIST2H3D, HOMEZ, TMPRSS6, SHOX2, LEF1, NPAS1, DNAJB5, ALX1, SUDS3, TAF9B, CRYM, RREB1, JAZF1, HEXIM1, HEXIM2, TCF7L2, ZFHX3, CBX6, PCGF2, PLK1, ESR2, BMP6, NFIA, H2AFY2, NFIC, TRPV4, BRMS1L, PAF1, CPEB3, TP73
GOTERM_BP_DIRECT	GO:0035556	intracellular signal transduction	50	0.001960633	-	DGKG, MAST4, AKAP13, PLCZ1, GPER1, AKT3, BLNK, JAK3, IKBKE, RGS9, TNS2, RGS6, PLEKHG7, PDPK1, MASTL, CIT, BCR, TIAM2, NRG4, ARHGEF4, TSSK3, PRKCQ, TSSK4, RAF1, TSSK2, BRSK1, NPR2, WNK4, ADCY3, ASB14, ADCY8, ADCY7, ASB16, NUAK1, CHN2, SH2B3, DCLK2, BMX, SPSB3, ADCY10, SNRK, RPS6KB1, CAMK4, PPP1R1C, GPR182, WNK3, CSPG4, PKN1, PLCD4, PLCD1
GOTERM_BP_DIRECT	GO:0007616	long-term memory	7	0.003074303	-	NTF4, CALB1, CAMK4, LRRN4, CTNS, ADCY8, GRIN1
GOTERM_BP_DIRECT	GO:0009653	anatomical structure morphogenesis	5	0.004621553	-	FBN2, FBN3, ANKRD11, FSCN2, FBN1
GOTERM_BP_DIRECT	GO:0007155	cell adhesion	29	0.00501397	-	COL15A1, LAMA2, LAMA4, STAB2, ICAM2, WISP3, ICAM3, THBS2, CYR61, WISP1, HAPLN2, SPP1, HAS3, NCAM1, IGFBP7, MPDZ, AOC3, FARP2, POSTN, TOR1A, ITGA4, DSCAM, GP1BA, BCAN, NINJ2, ITGA7, CNTN2, TLN2, CD44
GOTERM_BP_DIRECT	GO:0042490	mechanoreceptor differentiation	4	0.005593819	-	NTF4, NTRK1, NTRK2, NTRK3
GOTERM_BP_DIRECT	GO:0007420	brain development	18	0.005923926	-	MED1, FOXC1, SLC6A17, C2CD3, SPHK2, SLC23A1, IFT172, ROGDI, CTNS, SPATA5, SHROOM4, SRR, H2AFY2, STK36, COL4A1, NNAT, POMK, APOD
GOTERM_BP_DIRECT	GO:0009791	post-embryonic development	16	0.006160292	-	KDM5B, SLC4A10, PRDM1, GIGYF2, RC3H2, FOXP2, ETNK2, STK36, IMPAD1, SCN9A, TBCE, PLAGL2, ALX4, ATF5, SOX6, SLC18A2
GOTERM_BP_DIRECT	GO:0035904	aorta development	7	0.006692512	-	LOX, SUFU, LRP2, PRICKLE1, PRDM1, SMAD6, PKD2
GOTERM_BP_DIRECT	GO:0051965	positive regulation of synapse assembly	13	0.008355414	-	NTRK1, NTRK2, AMIGO3, BDNF, NTRK3, NRXN3, SRPX2, ADGRB2, LRRTM2, ADGRB1, FLRT1, ADGRL1, GHRL
GOTERM_BP_DIRECT	GO:0042391	regulation of membrane potential	14	0.009872663	-	KCNH2, HCN3, KCNH4, SLC26A1, KCNJ11, CHRNA7, ABCB5, RIMS2, CHRND, CHRNE, KCNMA1, SLC26A8, SLC26A5, KCNH1
GOTERM_BP_DIRECT	GO:0003170	heart valve development	4	0.01278482	-	SHOX2, SOX9, PRDM1, SMAD6
GOTERM_BP_DIRECT	GO:0030183	B cell differentiation	11	0.013333764	-	NTRK1, POU1F1, GON4L, DCLRE1C, CLCF1, CEBPG, IFNK, PIK3R1, JAK3, TSHR, NHEJ1
GOTERM_BP_DIRECT	GO:0007596	blood coagulation	11	0.013333764	-	PROC, SERPIND1, F10, PDGFD, HNF4A, DTNBP1, PROZ, PDGFA, GP1BA, F2, F2RL2
GOTERM_BP_DIRECT	GO:0006468	protein phosphorylation	16	0.01408627	-	BRSK1, WNK4, PIK3R1, RUNX3, P2RX7, SNRK, NUAK1, WNK3, AAK1, NEK10, SIK3, CCL3, TSSK3, TSSK4, TSSK2, IP6K3
GOTERM_BP_DIRECT	GO:0010107	potassium ion import	8	0.015758662	-	ATP4A, KCNJ11, KCNJ12, KCNJ9, KCNJ13, KCNJ14, KCNJ2, KCNJ3
GOTERM_BP_DIRECT	GO:0007601	visual perception	19	0.018507685	-	CHRNB2, SLC24A1, CABP4, ABCA4, CRYBA1, GUCA1C, RLBP1, BEST1, CRYGC, TRPM1, CACNB2, GLRA1, OPN1SW, MFRP, NOB1, OPA3, PDCL, PDE6C, PDE6A
GOTERM_BP_DIRECT	GO:0006936	muscle contraction	8	0.019762875	-	TMOD1, GLRA1, MYOM1, CALD1, TMOD4, CACNA1S, MYOM2, LMOD3
GOTERM_BP_DIRECT	GO:0006310	DNA recombination	8	0.019762875	-	RECQL4, RAD52, RTEL1, RECQL5, RUVBL1, RECQL, LIG3, NHEJ1
GOTERM_BP_DIRECT	GO:0034446	substrate adhesion-dependent cell spreading	9	0.021344689	-	ITGA4, AXL, TYRO3, FN1, LAMB1, ITGB7, SRCIN1, FERMT2, FERMT3
GOTERM_BP_DIRECT	GO:0003281	ventricular septum development	7	0.021663128	-	SUFU, NPRL3, XIRP2, LRP2, PRDM1, SMAD6, CYR61
GOTERM_BP_DIRECT	GO:0006811	ion transport	7	0.021663128	-	CHRNE, CHRNA7, SLCO1A2, SLCO4C1, SLCO2A1, SLC12A1, SLCO5A1
GOTERM_BP_DIRECT	GO:0051480	regulation of cytosolic calcium ion concentration	6	0.022565376	-	TRPC5, CALB1, TRPC3, GPER1, PDE6A, F2
GOTERM_BP_DIRECT	GO:0007628	adult walking behavior	9	0.025668406	-	SPTBN4, GLRA1, CNTN2, HTRA2, CACNA1A, CTNS, MAPT, HIPK2, SCN1A
GOTERM_BP_DIRECT	GO:0001942	hair follicle development	9	0.025668406	-	EDAR, FZD3, VANGL2, TNFRSF19, ALX4, SOX9, LDB2, APCDD1, DNASE1L2
GOTERM_BP_DIRECT	GO:0051453	regulation of intracellular pH	7	0.027447305	-	SLC4A8, SLC4A9, SLC26A1, SLC9A8, SLC26A8, SLC4A10, SLC26A5
GOTERM_BP_DIRECT	GO:0098779	mitophagy in response to mitochondrial depolarization	21	0.028027359	-	SERPINB10, MYOM1, FANCC, ATG14, TEX38, NR2C2, PHYHIP, FANCF, TXLNA, EVA1B, SNTG1, BOC, SLCO1A2, MAP1A, PNPO, ACIN1, MYH11, PRKG1, CLVS1, MAP3K12, ZNF189
GOTERM_BP_DIRECT	GO:0006813	potassium ion transport	6	0.029780314	-	ATP4B, ABCC8, KCNIP2, KCNAB1, TSC1, KCNJ2
GOTERM_BP_DIRECT	GO:0015701	bicarbonate transport	6	0.029780314	-	SLC4A8, SLC4A9, SLC26A1, SLC26A8, SLC4A10, SLC26A5

GOTERM_BP_DIRECT	GO:0032024	positive regulation of insulin secretion	6	0.029780314	-	TCF7L2, GPER1, NNAT, PFKM, SOX4, GCK
GOTERM_BP_DIRECT	GO:0042113	B cell activation	5	0.029850557	-	CHRNB2, LAT2, TXLNA, CHRNA7, CXCR5
GOTERM_BP_DIRECT	GO:0016337	single organismal cell-cell adhesion	13	0.030734001	-	IGSF5, KIRREL2, COL13A1, TTYH1, CTNND2, ICAM2, ICAM3, FNDC3A, NTN1, SRPX2, SOX9, ANXA9, LIMS2
GOTERM_BP_DIRECT	GO:0007612	learning	7	0.034165758	-	SLC8A3, NTRK2, SLC12A5, ATP8A1, NRXN3, CNTN2, SORCS3
GOTERM_BP_DIRECT	GO:0019228	neuronal action potential	7	0.034165758	-	CACNA1I, SCN10A, GPER1, SCN8A, SCN9A, SCN3A, SCN1A
GOTERM_BP_DIRECT	GO:0036514	dopaminergic neuron axon guidance	3	0.036806824	-	FZD3, VANGL2, CELSR3
GOTERM_BP_DIRECT	GO:0061470	T follicular helper cell differentiation	3	0.036806824	-	RC3H1, RC3H2, FOXP1
GOTERM_BP_DIRECT	GO:0090287	regulation of cellular response to growth factor stimulus	3	0.036806824	-	FBN2, FBN3, FBN1
GOTERM_BP_DIRECT	GO:0036515	serotonergic neuron axon guidance	3	0.036806824	-	FZD3, VANGL2, CELSR3
GOTERM_BP_DIRECT	GO:0035082	axoneme assembly	4	0.037466478	-	RSPH1, RSPH4A, RSPH9, CC2D2A
GOTERM_BP_DIRECT	GO:0042462	eye photoreceptor cell development	5	0.040783081	-	CRB1, MFRP, FSCN2, PRDM1, NR2E3
GOTERM_BP_DIRECT	GO:0019722	calcium-mediated signaling	7	0.041860022	-	LAT2, PDPK1, ALMS1, CCL3, HTR2B, BHLHA15, NCALD
GOTERM_BP_DIRECT	GO:0035023	regulation of Rho protein signal transduction	15	0.045951628	-	FARP2, FARP1, PLEKHG7, ARHGEF39, KALRN, FGD1, FGD2, BCR, FGD3, TIAM2, AKAP13, TIAM1, OBSCN, ARHGEF4, MCF2L2
GOTERM_BP_DIRECT	GO:0006302	double-strand break repair	10	0.046819644	-	RECQL4, RNF168, KAT5, DTX3L, EME2, EYA3, LIG3, PARP9, NHEJ1, REC8
