# Supplementary material for: Identification, Characterization, and Transcriptional Reprogramming of Epithelial Stem Cells and Intestinal Enteroids in Simian Immunodeficiency Virus Infected Rhesus Macaques
Source: Front Immunol. 2021 Nov 23;12:769990. doi: 10.3389/fimmu.2021.769990 (PMC8650114; doi:10.3389/fimmu.2021.769990)
Supplement: Supplementary file 18 [file Table_12.pdf]

Supplementary Table 12: The 80 significantly enriched GO terms in the Biological Processes Category among downregulated DEGs						
**Category**	**GO:number**	**Term**	**Count**	**PValue**	**FDR**	**Genes**
GOTERM_BP_DIRECT	GO:0051603	proteolysis involved in cellular protein catabolic process	22	7.43E-08	2.91E-04	HSPA5, CTSZ, CTSV, PSMB8, CTSS, PSMB6, SCPEP1, PSMB4, CASP8, PSMA4, PSMB5, PSMB2, PSMA1, CTSL, CLPP, CTSK, PSMB1, CAPN2, CTSH, CTSC, CTSB, LGMN
GOTERM_BP_DIRECT	GO:0050821	protein stabilization	33	3.43E-05	-	USP33, PHB, PARK7, IFI30, CLU, USP19, STK3, RASSF2, SUMO1, PDCD10, LAMP2, ZNF207, DVL3, CCT8, GOLGA7, TSPAN1, CCT5, CCT4, CCT2, USP7, COG7, PEX19, UBE2B, TESC, COG3, ATP1B3, CCT6A, PEX6, PIN1, NAA15, PPIB, PFN1, PFN2
GOTERM_BP_DIRECT	GO:0006412	translation	77	5.74E-05	-	RPL4, SLC25A1, HBS1L, SLC25A3, RPL3, MRPS14, RPL34, RPS17L, MRPL34, RPL10A, MRPL33, RPS15, MRPL4, MRPL3, RPS14, RPS19, RPL35, RPS11, RPS13, RPS12, RPS9, RPL21, RPS7, RPS8, RPL23, RPS5, MRPS23, MRPS18A, RPL13A, MRPS2, MRPS21, SLC25A15, SLC25A17, MRPL51, RPL37A, RPL27, SLC25A10, RPL26, SLC25A5, SLC25A11, RPL28, SLC25A4, SLC25A13, RPS4Y2, MRPL18, RPL12, RPL11, RPS27L, MRPL15, MRPS30, MRPL13, SLC25A27, MRPL20, PDF, RPS3, SLC25A20, RPL15, SLC25A22, SLC25A24, MRPL24, MRPL22, RPS26, SLC25A38, SLC25A37, SLC25A39, RPS29, RPL27A, SLC25A30, RPL22L1, RPS20, FAU, RSL24D1, RPS21, SLC25A33, RPS23
GOTERM_BP_DIRECT	GO:0017148	negative regulation of translation	17	6.10E-05	-	GRB7, TIA1, DDX3X, FMR1, DAPK3, MALSU1, EPRS, TOB1, FXR1, SYNCRIP, ILF3, CNOT2, ENC1, RPS3, EIF4E2, EIF4E, GAPDH
GOTERM_BP_DIRECT	GO:0006446	regulation of translational initiation	13	1.05E-04	-	EIF4A1, NCBP1, NCBP2, EIF1, GLE1, EIF3M, EIF5, EIF3L, EIF3I, EIF3J, EIF3E, EIF3F, EIF3D
GOTERM_BP_DIRECT	GO:0008152	metabolic process	40	1.26E-04	-	ACSS3, ACSS2, ACAA2, LPGAT1, GSTP1, ECI1, ECI2, IREB2, DLST, AGPAT2, AACS, AGPAT3, UGT8, SCP2, TMEM68, DBT, ACSS1, ACAD10, GSTM4, UGT1A1, GSTO1, EPHX2, OXSM, EDEM1, EDEM2, ECH1, ACSL5, ACSL4, MCAT, HDHD3, SUCLA2, GSTA4, LPCAT4, GSTA3, SUCLG2, ECHDC3, SUCLG1, ACO1, PNPLA6, PIGG
GOTERM_BP_DIRECT	GO:0000398	mRNA splicing, via spliceosome	29	1.57E-04	-	SF3B5, SF3B3, U2AF1, HTATSF1, UBL5, ZMAT2, RAVER1, U2AF2, SNRNP70, TRA2B, CWC22, DHX15, SNRPB2, TXNL4A, NCBP2, PRPF40A, CWC15, PLRG1, HNRNPM, PHF5A, DDX39A, LSM6, DDX39B, SYF2, HNRNPA2B1, SNRPA1, SNRPE, SNRPF, SNRPB
GOTERM_BP_DIRECT	GO:0006979	response to oxidative stress	22	2.14E-04	-	PRNP, NQO1, GPX2, GPX4, SRXN1, NDUFA12, IDH1, NDUFB4, OXSR1, PTGS1, MSRA, GCLC, WRN, NDUFS8, PSMB5, RRM2B, NAPRT, ERCC1, CAT, TXNIP, NDUFS2, GCLM
GOTERM_BP_DIRECT	GO:0006890	retrograde vesicle-mediated transport, Golgi to ER	13	2.91E-04	-	ARF3, COG7, COG4, COG3, STX18, ARCN1, RER1, ATP9B, RAB6B, ERGIC2, COPE, ARF5, SCYL1
GOTERM_BP_DIRECT	GO:0055088	lipid homeostasis	13	2.91E-04	-	CEBPA, GCDH, ACAD9, ETFA, ACADSB, USF1, COL4A3BP, ACOX2, PNPLA3, ACADM, ACAD10, ACADS, PNPLA2
GOTERM_BP_DIRECT	GO:0030433	ER-associated ubiquitin-dependent protein catabolic process	18	4.40E-04	-	EDEM1, AMFR, CCDC47, EDEM2, TMUB1, ERLIN1, ERLIN2, UBE2G2, UBE2J2, USP19, HSP90B1, PSMC5, OS9, PSMC4, DNAJC10, DNAJB9, STT3B, UBXN4
GOTERM_BP_DIRECT	GO:0006099	tricarboxylic acid cycle	12	5.43E-04	-	CS, FH, MDH1, IDH1, IDH2, DLST, SDHC, SUCLG1, PDHB, SDHA, SDHB, IDH3A
GOTERM_BP_DIRECT	GO:0006886	intracellular protein transport	51	6.25E-04	-	VPS29, CLTA, SNX13, IPO7, HID1, IPO5, XPO1, TIMM17A, AP1S1, ARFIP1, AP3S1, AP1S3, SEC61B, TNPO2, TBC1D22A, AP2M1, TBC1D22B, YWHAH, NSF, COG7, STX8, COG3, AP1B1, COPZ1, TOM1L1, SLU7, STAM2, NAPA, NAPB, RABGAP1, TMED10, SAR1B, COPB1, STX18, USO1, VPS26A, STX10, AP3M1, SNX1, SNX2, TBC1D14, STX5, SNX9, SEC23B, TBC1D15, BCAP31, TBC1D8B, GRTP1, AP2B1, VPS41, RAN
GOTERM_BP_DIRECT	GO:0001731	formation of translation preinitiation complex	11	6.26E-04	-	MCTS1, EIF3M, EIF2S3, EIF3L, EIF3I, EIF3J, EIF3H, EIF2D, EIF3E, EIF3F, EIF3D
GOTERM_BP_DIRECT	GO:0008637	apoptotic mitochondrial changes	9	7.15E-04	-	PPP2CB, GCLC, AIFM2, PPIF, JTB, BAX, GCLM, SLC25A4, MCL1
GOTERM_BP_DIRECT	GO:0033539	fatty acid beta-oxidation using acyl-CoA dehydrogenase	9	0.001307806	-	GCDH, ACOX2, ACAD9, ETFA, ACADM, ETFB, ACAD10, ACADSB, ACADS
GOTERM_BP_DIRECT	GO:0006406	mRNA export from nucleus	13	0.001506395	-	ENY2, NUP133, NCBP1, SHFM1, THOC3, SMG7, THOC5, THOC7, GLE1, DDX19B, DDX39A, DDX39B, HNRNPA2B1
GOTERM_BP_DIRECT	GO:0043161	proteasome-mediated ubiquitin-dependent protein catabolic process	32	0.001573166	-	PSMD12, UBXN2B, RNF4, PPP2CB, PSMD6, MTA1, PSMB5, PSMD7, SHARPIN, MAEA, HECTD3, PSMB3, PSMD2, FBXW5, UBE2B, FBXL19, KCTD2, UBE2A, RAD23B, SIRT2, CUL4A, RNF145, RNF126, UBE2R2, TBL1XR1, SPOPL, RNF181, UBE2K, BIRC2, DZIP3, RNF122, NFE2L2
GOTERM_BP_DIRECT	GO:1903608	protein localization to cytoplasmic stress granule	6	0.002062698	-	TIA1, DDX3X, SSB, DHX9, DDX1, YBX1
GOTERM_BP_DIRECT	GO:0006457	protein folding	32	0.002157041	-	FKBP15, TXN, HSP90B1, NUDCD2, CCT8, CCT5, NKTR, CCT4, RANBP2, PDIA3, CCT2, MKKS, VBP1, TBCC, PDRG1, CCT6A, DNAJA1, ERP44, DNAJC1, TMX3, TMX1, FKBP8, PPIF, PFDN1, PFDN2, PPIH, PPIG, PPIB, PFDN4, PFDN5, PPIA, AARS
GOTERM_BP_DIRECT	GO:0007264	small GTPase mediated signal transduction	55	0.002396543	-	ARF3, ARF1, GDI1, RAB3D, RND3, RAB22A, RAPGEFL1, ARL5B, YWHAQ, RAC1, ARL5A, HRAS, RAB8A, RAB2A, RAB2B, ARL14, RRAS2, RHOA, GEM, RAB32, ARL4C, ARL4A, RAP2A, RAP2B, RABL3, RHOU, RHOV, SOS2, ARF5, ARL8A, ARL8B, RABL6, RAB1A, RAB5B, RAB5C, RAP1GDS1, ARL2, RAB21, ARFRP1, RHOT2, NRAS, RAP1A, RAB40C, RAB25, GNA12, RAB28, RAB6B, RAB4A, RAB11A, RAB11B, RAB14, RHEB, RAB13, RAB18, RAN
GOTERM_BP_DIRECT	GO:0034314	Arp2/3 complex-mediated actin nucleation	8	0.002484701	-	ACTR3, ARPC2, ARPC3, ARPC1B, ARPC1A, ARPC5L, ARPC4, ARPC5
GOTERM_BP_DIRECT	GO:1901998	toxin transport	12	0.002731567	-	DNAJA1, CCT2, SCFD1, HSPA5, MEP1B, BNIP3, CCT8, RAB28, ANTXR2, COPZ1, CCT5, CCT4
GOTERM_BP_DIRECT	GO:0006396	RNA processing	19	0.002816681	-	SSB, PNPT1, DHX9, YTHDC2, DDX54, LARP7, TRMT2B, WBP11, LSM4, MRM1, U2SURP, MRPL44, DHX40, DHX30, PRPF39, HNRNPK, DHX35, GRSF1, SUGP1
GOTERM_BP_DIRECT	GO:0015991	ATP hydrolysis coupled proton transport	14	0.003075474	-	ATP6V1A, ATP6V0B, ATP6V0E1, ATP5A1, ATP5G3, TCIRG1, ATP5G1, ATP6V1B2, ATP6V1H, ATP6V1E1, ATP6V0D1, ATP6V0E2, ATP6V0C, ATP6V1C1
GOTERM_BP_DIRECT	GO:0043001	Golgi to plasma membrane protein transport	9	0.003602303	-	NSF, ARFRP1, GOLPH3, VAMP7, ANK3, VAMP5, GOLGA7, VAMP2, VAMP3
GOTERM_BP_DIRECT	GO:0006635	fatty acid beta-oxidation	12	0.003825901	-	HADHB, HADHA, SLC25A17, ACAA2, BDH2, ACOX2, ECI1, HSD17B4, PEX2, ABCD1, SLC27A2, DECR1
GOTERM_BP_DIRECT	GO:0050892	intestinal absorption	6	0.004739935	-	SCARB1, MOGAT2, IREB2, GCNT3, ACO1, F11R
GOTERM_BP_DIRECT	GO:0006511	ubiquitin-dependent protein catabolic process	29	0.005998237	-	UFD1L, USP15, USP16, USP10, CUL3, USP33, CUL2, RNF8, FBXO25, CUL1, UBE2D1, USP19, HERPUD1, PSMD3, TCEB1, USP47, USP7, USP48, USP22, UBE2G1, UBE2G2, USP28, UCHL3, UCHL5, CUL4A, PSMA4, PSMA1, PSMA2, SQSTM1
GOTERM_BP_DIRECT	GO:0006897	endocytosis	21	0.007951542	-	RAB1A, CSNK1G3, SORT1, CSNK1D, SNX10, PIK3C2A, RAB22A, SNX3, EHD1, SNX4, C15H9ORF72, NECAP2, SNX1, VAMP7, SNX2, RINL, ATP6V1H, SNX9, HRAS, ATP9B, PICALM
GOTERM_BP_DIRECT	GO:1903543	positive regulation of exosomal secretion	7	0.008172602	-	SDCBP, TSG101, PDCD6IP, SDC4, VPS4B, SDC1, ATP13A2
GOTERM_BP_DIRECT	GO:0006605	protein targeting	9	0.008181135	-	YWHAQ, YWHAB, GIPC1, SEC61G, RPL11, AP1S3, SYNJ2BP, YWHAZ, TOMM20
GOTERM_BP_DIRECT	GO:1904871	positive regulation of protein localization to Cajal body	5	0.008908992	-	CCT6A, CCT2, CCT8, CCT5, CCT4
GOTERM_BP_DIRECT	GO:0000209	protein polyubiquitination	23	0.009196681	-	UBE2B, UBE3C, RNF14, AMFR, CUL3, UBE2G1, UBE2G2, UBE2A, FBXO22, RNF41, RNF145, RNF126, RNF114, RNF138, RNF19A, UBR5, MKRN1, RNF181, TRIM32, UBE2K, BIRC2, DZIP3, RNF122
GOTERM_BP_DIRECT	GO:0006783	heme biosynthetic process	6	0.009197393	-	ALAD, SLC25A38, UROS, SLC11A2, ATPIF1, CPOX
GOTERM_BP_DIRECT	GO:0006810	transport	24	0.009737701	-	MTCH1, TMEM184C, SLC25A3, SORT1, SLC35F5, NUTF2, NXT2, SLC25A38, SEC14L2, SLC25A27, SLC25A15, SLC25A37, SLC25A39, PITPNM1, ASNA1, G3BP1, G3BP2, TMED2, TMED1, SLC25A20, SLC25A11, SLC25A33, ABCG1, PAFAH1B1
GOTERM_BP_DIRECT	GO:0015031	protein transport	16	0.00979566	-	VPS29, ENY2, TSG101, GDI1, TIMM13, MIA3, SCAMP1, SCAMP3, ARCN1, SCAMP2, SCAMP4, SEC61A1, SNX4, PPT1, KDELR2, BBS7
GOTERM_BP_DIRECT	GO:0010501	RNA secondary structure unwinding	14	0.011073385	-	DDX28, DDX18, EIF4A1, DDX5, DDX3X, DDX1, DDX56, DDX42, DDX54, DDX41, DDX50, DDX19B, DDX39A, DDX39B
GOTERM_BP_DIRECT	GO:0034976	response to endoplasmic reticulum stress	13	0.011627875	-	EIF2B5, PDIA3, UBA5, PPP2CB, ERP44, UFL1, COL4A3BP, UFM1, TMX3, TMEM33, TMX1, FLOT1, NRBF2
GOTERM_BP_DIRECT	GO:0007030	Golgi organization	18	0.012277731	-	ARFGEF1, RAB2A, RAB1A, COG7, TMED10, UBXN2B, COG4, COG3, SURF4, USO1, ATL2, GOLPH3L, CSNK1D, LMAN1, GOLGA5, ZW10, TMED2, DYM
GOTERM_BP_DIRECT	GO:0006887	exocytosis	15	0.012882295	-	EXOC7, MIA3, LIN7C, RAB11A, VAMP8, VAMP7, PDZD11, SCRN2, VAMP4, VAMP5, YKT6, VAMP2, CDK16, VAMP3, EXOC1
GOTERM_BP_DIRECT	GO:0034612	response to tumor necrosis factor	7	0.013047946	-	CASP8, GCH1, YTHDC2, ADAM10, ADAM9, TRIM32, CXCL16
GOTERM_BP_DIRECT	GO:0006749	glutathione metabolic process	11	0.016004459	-	GSTZ1, EEF1G, GSTM4, G6PD, GSTA4, GSTO1, GSTP1, IDH1, MGST1, OPLAH, SOD1
GOTERM_BP_DIRECT	GO:0090200	positive regulation of release of cytochrome c from mitochondria	9	0.016120643	-	FAM162A, BNIP3, PDCD5, PPIF, TNFSF10, BAX, PMAIP1, APOPT1, BID
GOTERM_BP_DIRECT	GO:0015986	ATP synthesis coupled proton transport	10	0.016303296	-	ATP5D, ATP5A1, ATP5J, ATP5C1, TCIRG1, ATP5G3, ATP5H, ATP5F1, ATP5G1, ATP5L

GOTERM_BP_DIRECT	GO:1903553	positive regulation of extracellular exosome assembly	4	0.016508437	-	SDCBP, PDCD6IP, SDC4, SDC1
GOTERM_BP_DIRECT	GO:0010950	positive regulation of endopeptidase activity	4	0.016508437	-	PRELID1, PSME3, PSME1, PSME2
GOTERM_BP_DIRECT	GO:1904851	positive regulation of establishment of protein localization to telomere	5	0.018037384	-	CCT6A, CCT2, CCT8, CCT5, CCT4
GOTERM_BP_DIRECT	GO:0009615	response to virus	14	0.020687323	-	URI1, IFITM1, DDX3X, DDX1, MST1R, GTF2F1, CLU, EEF1G, NPC2, CFL1, DHX58, AP1S1, MYD88, CCT5
GOTERM_BP_DIRECT	GO:0032436	positive regulation of proteasomal ubiquitin-dependent protein catabolic process	16	0.020844468	-	PSMD10, VCP, RNF14, CSNK1D, RCHY1, CLU, FBXO22, GCLC, RNF114, SUMO1, RNF138, RFWD2, RNF19A, BBS7, TRIB1, SOCS5
GOTERM_BP_DIRECT	GO:0009060	aerobic respiration	8	0.021305361	-	COX19, CAT, UQCRC1, MTFR1, UQCRC2, MTFR1L, SDHB, NDUFV1
GOTERM_BP_DIRECT	GO:0045454	cell redox homeostasis	18	0.023765504	-	PDIA3, TXNDC9, GLRX3, GLRX5, GLRX, TXN, GLRX2, PRDX6, TXN2, ERP44, PRDX2, GCLC, PRDX4, TMX3, TMX1, DNAJC10, DLD, NFE2L2
GOTERM_BP_DIRECT	GO:0045116	protein neddylation	6	0.02514335	-	UBA3, NEDD8, NAE1, RNF7, UBE2M, RBX1
GOTERM_BP_DIRECT	GO:0043085	positive regulation of catalytic activity	6	0.02514335	-	APH1A, NCSTN, YWHAB, FGFR4, SLC37A4, SOD1
GOTERM_BP_DIRECT	GO:0051016	barbed-end actin filament capping	6	0.02514335	-	GSN, CAPZB, CAPZA1, CAPZA2, TWF1, CAPG
GOTERM_BP_DIRECT	GO:0006614	SRP-dependent cotranslational protein targeting to membrane	6	0.02514335	-	SEC61A1, SRP54, SRP68, SRP14, SRP9, SEC63
GOTERM_BP_DIRECT	GO:0061136	regulation of proteasomal protein catabolic process	6	0.02514335	-	UBB, PSMD14, PSME3, PSME1, PSME2, UCHL5
GOTERM_BP_DIRECT	GO:0021762	substantia nigra development	11	0.025812503	-	G6PD, HSPA5, YWHAQ, MAOB, MAPKAP1, ATP5J, ACTB, SIRT2, RHOA, YWHAH, COX6B1
GOTERM_BP_DIRECT	GO:2000379	positive regulation of reactive oxygen species metabolic process	8	0.02896116	-	CDKN1A, RIPK3, GADD45A, TSPO, GRB2, ROMO1, RNF41, NFE2L2
GOTERM_BP_DIRECT	GO:0097345	mitochondrial outer membrane permeabilization	5	0.031334833	-	BNIP3L, RHOT2, BNIP3, BLOC1S2, BID
GOTERM_BP_DIRECT	GO:0033146	regulation of intracellular estrogen receptor signaling pathway	5	0.031334833	-	UFL1, UFM1, SRC, UBA5, UFSP2
GOTERM_BP_DIRECT	GO:2000146	negative regulation of cell motility	5	0.031334833	-	TACSTD2, CTNNA1, PIN1, AP1AR, SPINT2
GOTERM_BP_DIRECT	GO:0042787	protein ubiquitination involved in ubiquitin-dependent protein catabolic process	28	0.032439793	-	UBE3C, RNF14, CUL3, CUL2, CUL1, RNF7, RCHY1, RNF5, HERC3, MAEA, OS9, HECTD3, RNF19A, UBR5, TCEB1, AMFR, KLHL21, FBXL14, RBX1, CUL4A, RNF145, RNF126, SPOPL, RNF181, TRIM32, DZIP3, RNF122, ANAPC2
GOTERM_BP_DIRECT	GO:0007266	Rho protein signal transduction	10	0.034450618	-	CNKSR1, CDC42EP4, ARHGDIA, GNA12, CDC42EP2, EPS8L2, TAX1BP3, SYNJ2BP, CTNNAL1, RHOA
GOTERM_BP_DIRECT	GO:0070934	CRD-mediated mRNA stabilization	4	0.036132163	-	SYNCRIP, DHX9, HNRNPU, YBX1
GOTERM_BP_DIRECT	GO:0006750	glutathione biosynthetic process	4	0.036132163	-	GCLC, MGST2, HAGH, GCLM
GOTERM_BP_DIRECT	GO:0031340	positive regulation of vesicle fusion	4	0.036132163	-	C2CD5, ANXA1, ANXA2, AKT2
GOTERM_BP_DIRECT	GO:0034379	very-low-density lipoprotein particle assembly	4	0.036132163	-	SOAT1, DGAT1, SOAT2, APOC3
GOTERM_BP_DIRECT	GO:0000387	spliceosomal snRNP assembly	9	0.036623281	-	CLNS1A, STRAP, SNRPE, SNRPD3, GEMIN7, SNRPF, LSM4, RBM22, SNRPB
GOTERM_BP_DIRECT	GO:0031954	positive regulation of protein autophosphorylation	6	0.037265312	-	RAP2C, TOM1L1, RAP2A, RAP2B, RASSF2, PDGFC
GOTERM_BP_DIRECT	GO:0030837	negative regulation of actin filament polymerization	6	0.037265312	-	ARFGEF1, MKKS, PRKCD, TWF1, PFN1, PFN2
GOTERM_BP_DIRECT	GO:0006607	NLS-bearing protein import into nucleus	7	0.038848571	-	RANBP2, NUP54, KPNA4, KPNA2, RANBP6, TNPO2, IPO5
GOTERM_BP_DIRECT	GO:0032981	mitochondrial respiratory chain complex I assembly	7	0.038848571	-	NDUFS8, NDUFS7, NDUFAF4, NDUFS4, ACAD9, NDUFAF3, FOXRED1
GOTERM_BP_DIRECT	GO:0043123	positive regulation of I-kappaB kinase/NF-kappaB signaling	29	0.039484127	-	ECM1, SHARPIN, CASP8, TBK1, TFG, TSPAN6, MIER1, TNFSF10, FADD, ECT2, NDFIP2, UNC5CL, UBE2I, NDFIP1, RIPK2, PLK2, F2R, SHISA5, RHOA, TNFRSF1A, PPM1A, TNIP2, TERF2IP, CTNNB1, UBE2V1, TRIM32, BIRC2, TMEM9B, MYD88
GOTERM_BP_DIRECT	GO:0042632	cholesterol homeostasis	14	0.04176101	-	SCARB1, DGAT2, EPHX2, MTTP, PCSK9, APOC3, APOA4, TMEM97, EHD1, SOAT1, NPC2, SOAT2, SLC37A4, ABCG1
GOTERM_BP_DIRECT	GO:0070301	cellular response to hydrogen peroxide	9	0.046185627	-	KDM6B, PLEKHA1, PRKCD, PPIF, RPS3, PARK7, ECT2, MAP3K5, NFE2L2
GOTERM_BP_DIRECT	GO:0010388	cullin deneddylation	5	0.049044785	-	COPS4, COPS6, COPS2, COPS7A, COPS8
GOTERM_BP_DIRECT	GO:0032486	Rap protein signal transduction	5	0.049044785	-	RAP1B, RAP2C, RAP2A, RAP2B, RAP1A
GOTERM_BP_DIRECT	GO:0045948	positive regulation of translational initiation	5	0.049044785	-	YTHDF1, DDX3X, POLR2D, RPS6KB2, POLR2G
GOTERM_BP_DIRECT	GO:0033077	T cell differentiation in thymus	8	0.049353754	-	RIPK3, NKAP, ZBTB1, CTNNB1, LIG4, FADD, B2M, JMJD6
